# Systematic Parameter Determination Aimed at a Catalyst-Controlled Asymmetric Rh(I)-Catalyzed Pauson–Khand Reaction

**DOI:** 10.1021/acscatal.4c04490

**Published:** 2024-11-05

**Authors:** Yifan Qi, Luke T. Jesikiewicz, Grace E. Scofield, Peng Liu, Kay M. Brummond

**Affiliations:** Department of Chemistry, University of Pittsburgh, Pittsburgh, Pennsylvania 15260, United States

**Keywords:** catalyst control, asymmetric Pauson**−**Khand reaction, chiral quaternary carbon centers, solvent effect, counterion effect, bisphosphine ligand effect, enyne effect, descriptor selection

## Abstract

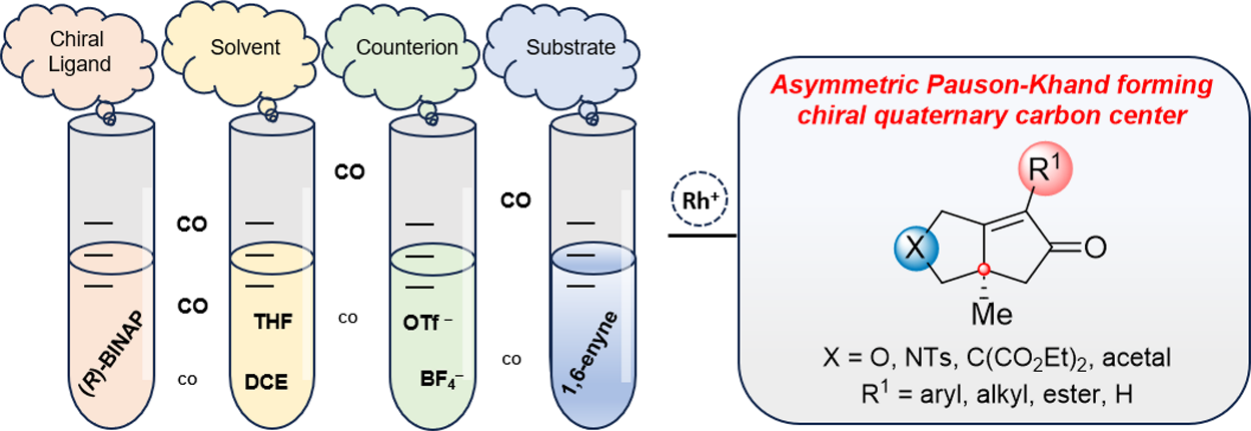

Transition metal-catalyzed carbocyclization reactions have revolutionized the synthesis of complex cyclic organic compounds. Yet, subtle substrate changes can significantly alter reaction pathways. The asymmetric Rh(I)-catalyzed Pauson**–**Khand reaction (PKR) exemplifies such a reaction, hindered by a narrow substrate scope and competing reactivity modes. In this study, we identified parameters predictive of the yield and enantioselectivity in the catalyst-controlled asymmetric PKR, using 1,6-enynes with a 2,2-disubstituted alkene. In this way, ring-fused cyclopentenones can be formed with chiral quaternary carbon centers. Using bisphosphine ligand parameters from palladium complexes, including the energy of the Pd lone pair orbital and the angle formed by the phosphorus aryl groups on the ligand, we established strong correlations with experimental ln(*er*) (*R*^2^ = 0.99 and 0.91) for two distinct precursors. Solvent dipole moments correlated with ln(*er*) for high-dipole-moment precursors (*R*^2^ = 0.94), while Abraham’s hydrogen bond basicity is more relevant for low-dipole-moment precursors (*R*^2^ = 0.93). Additionally, counterions were found to have a significant impact on the PKR reactivity and selectivity, as does the steric demand of the alkyne substituent of the enyne precursor. In the latter case, ln(*er*) correlates with Sterimol B_1_ values for products from different alkyne substituents (*R*^2^ = 0.99). Furthermore, the computed C≡C wavenumber of the enyne precursor can be directly aligned with the yield of asymmetric PKRs.

## Introduction

**Figure 1:**
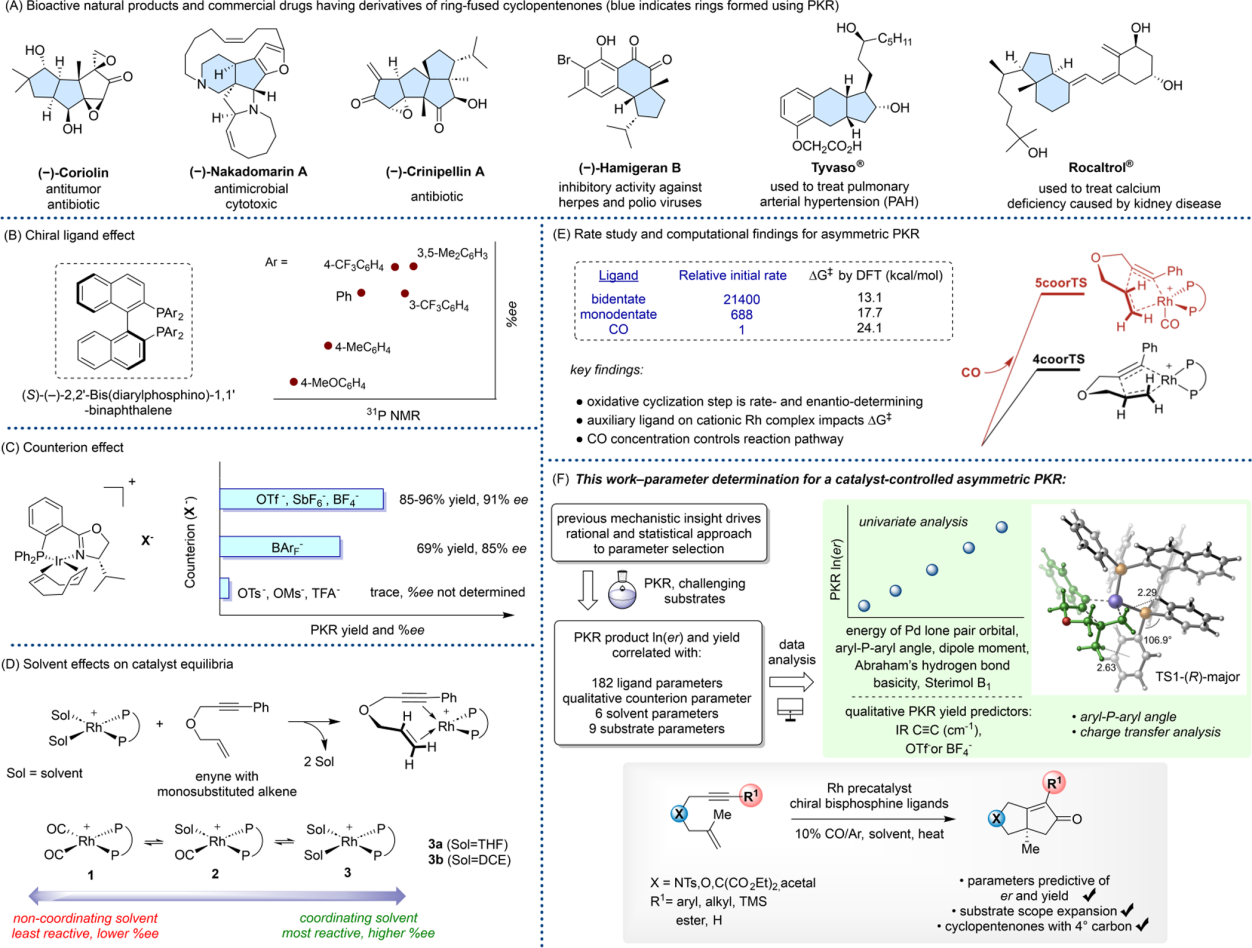
(A) Bioactive natural products and representative drugs accessed through PKR. (B) Impact of the electronic character of bisphosphine ligands on the PKR % ee (Jeong and co-workers).^13^ (C) Impact of counterions on PKR reactivity and selectivity (Pfaltz and co-workers).^14^ (D) Previous independent studies showing the impact of ancillary ligands on enyne binding and catalyst equilibria (Brummond and Liu/Jeong).^16,17^ (E) Previous rate study and computational findings for asymmetric PKR (Brummond and Liu).^16^ (F) This work.

The Pauson**–**Khand reaction (PKR) is a powerful method for synthesizing ring-fused cyclopentenones^1−4^—products equipped for building up complexity to provide compound structures in bioactive natural products and commercial drugs (Figure 1A).^5−11^ Relying on the structural features of the substrate (e.g., rigidity, sterics, and electronics), racemic PKRs have utilized midsynthetic sequences to provide good yields and high diastereoselectivities of key synthetic targets. However, the *asymmetric PKR* has the potential to generate chiral nonracemic 5,5-, 5,6-, and 5,7-ring systems directly without these limitations. Rh(I)-catalyzed PKRs offer the most versatile asymmetric PKR protocol to date, and yet, the substrate structure remains a critical hindrance to a generalized enantioselective PKR. As such, empirical catalyst—and reaction—optimization studies are often required for each substrate.

With the goal of realizing a fully catalyst-controlled asymmetric Rh(I)-catalyzed PKR, we and others have focused on gaining an understanding of the key elementary steps of this transformation. For example, using a combination of experiment and theory, we identified that the oxidative cyclization step is stereodetermining and utilized the corresponding transition state structures and activation barriers to establish our catalyst design. This approach has allowed us to extend the asymmetric PKR to allene-ynes.^12^

Despite these successes, reported studies have generally focused on PKRs utilizing simple enyne systems and have been obtained largely in isolation; data (e.g., yields, ees, reaction rates, and side-products) cannot be reliably used for determining reactivity and selectivity factors. For instance, Jeong and coworkers showed that electron-withdrawing aryl groups on the bisphosphine ligand of the Rh(I) catalyst generally provided higher enantiomeric excesses (ees), albeit with variable yields and significant sensitivity to the substrate structure (Figure 1B).^13^

Similarly, the role that the counterion plays in PKR reactivity and selectivity has been evaluated but remains poorly understood. Pfaltz and coworkers performed an extensive study on counterion effects using Ir-Phox catalysts and showed that the counterion had a strong influence on the PKR yield and ees of three different enynes. In these cases, small weakly coordinating counterions (OTf^–^, SbF_6_^–^, and BF_4_^–^) gave higher yields and ees compared to larger noncoordinating anions such as BArF^–^. When OTs^–^, OMs^–^, and TFA^–^ were used as counterions, only trace amounts of PKR products were observed (Figure 1C).^14^ This is likely due to the electronic requirements of the reaction center, which differs between the mechanistic steps. For example, an electron-deficient reaction center lowers the barriers for complexation of the enyne to the Rh(I) metal and the reductive elimination step, whereas an electron-rich reaction center lowers the barrier for the oxidative cyclization step. The analysis is complicated further, as the solvent impacts the coordinating ability of the counterion. In less polar solvents, the counterion is predicted to be closer to the reaction center, existing as a contact ion-pair with the catalyst. Conversely, in more polar solvents, the counterion is isolated from the reaction center, leading to a dissociated ion pair.^15^

In yet other efforts, computed Gibbs free energy values for the complexation of the enyne to **1**, **3a**, and **3b** shows that the auxiliary ligand may greatly impact the catalyst equilibria (Figure 1D). For example, the complexation of the nonsterically demanding ether-tethered enyne with a monosubstituted alkene to Rh having two COs, **1**, is highly endergonic (Δ*G* = 27.6 kcal/mol), while it is slightly endergonic for **3a** (Δ*G* = 0.8 kcal/mol) and exergonic for **3b** (Δ*G* = −6.8 kcal/mol).^16^ Jeong and co-workers demonstrated that the asymmetric Rh(I)-catalyzed PKR yields higher ees in the presence of coordinating solvents such as tetrahydrofuran (THF) and postulated that these solvents compete with CO as ligands for Rh(I). Solvent coordination shifts the enyne complexation equilibria toward the more reactive catalytic species **2** and **3**. On the other hand, noncoordinating solvents, such as toluene, promote the formation of the less reactive CO-saturated **1**; reactions in noncoordinating solvents require higher reaction temperatures (Figure 1D).^17^

Recently, DFT calculations have been performed to establish the oxidative cyclization step as the rate- and enantio-determining step for the asymmetric PKR for an ether-tethered enyne with a monosubstituted alkene.^16^ Initial rate studies showed that the auxiliary ligand on the Rh(I)–enyne complex is an important reactivity driver. When using the bidentate bisphosphine (*R*)-BINAP as a chiral ligand, the PKR was 21,400-fold faster than when using CO-only conditions (Figure 1E). The monodentate phosphoramidite ligand (*S*)-Monophos showed an intermediate level of reactivity. Another important finding included two distinct reaction pathways that produced enantiomeric PKR products. The TSs for the 4-coordinated pathway are favored over the 5-coordinated pathway by 3.5 kcal/mol under a low CO concentration (Figure 1E).^16^

While these studies have contributed greatly to our understanding of the asymmetric Rh(I)-catalyzed PKR mechanism, it has done more to reveal the complex interrelated factors that control PKR selectivity and reactivity (e.g., CO concentration, ligand identity, solvent, and substrate structure) than to produce significant advances in the asymmetric PKR reaction scope. If anything, the growing appreciation of the mechanistic complexity of the asymmetric PKR has hindered its application to complex molecular target synthesis.

Given that these studies have evaluated reaction parameters largely in isolation, it remains difficult to discern their inter-relationships. As such, catalyst and PKR condition optimization have largely been arbitrary and empirical. To address this gap in the asymmetric PKR methodology, we set out to combine previous experimental and computational mechanistic insights (Figures 1B–E) with a systematic evaluation of a variety of reaction conditions to correlate the product yield and enantioselectivity with parameters for an array of enyne precursors (Figure 1F). Our initial studies utilized the synthetically useful but challenging 1,6-enyne precursors possessing a 2,2-disubstituted alkene for which there are few data (Figure 1F). (Prior to 2023, only two enynes featuring a 2,2-disubstituted alkene were successfully shown to undergo an asymmetric Ir(I) or Rh(I) *catalyst-controlled* PKR.^14,18−25^ In 2023, Baik and co-workers showed that an asymmetric PKR using 2,2-disubstituted alkenes could be effected so long as the enyne precursors bore a chloroacetylene group.^26^) Upon PKR, these substrates provide chiral nonracemic 5,5-ring systems having a quaternary (4°) carbon at the ring fusion.^27−29^ Despite their value, these precursors are also prone to undergoing reactions other than the PKR.^30−33^

Herein, we report parameters for the chiral ligand,^13,16,26^ solvent,^17,34−36^ and substrate that show excellent correlation with the product ln(*er*) (Figure 1F). We also identified qualitative descriptors for the substrate and the Rh precatalyst counterion that are predictive of the reactivity trends and yield (Figure 1F). Efforts to identify parameters predictive of ln(*er*) and yield led to 13 novel chiral nonracemic 5,5-ring systems bearing a 4° carbon.

## Methods and Materials

### Identifying Chiral Bisphosphine Ligand Descriptors for Rh(I)-Catalyzed PKR Yield and Enantioselectivity

To identify potential chiral ligand parameters that correlate with the PKR yield and enantioselectivity, five bisphosphine ligands were evaluated, including a combination of a binaphthyl or biphenyl backbone with tolyl, mesityl, or 3,5-di-*tert*-butyl-4-methoxy-aryl groups on the donor phosphine atoms (e.g., (*R*)-BINAP (**L1**), (*R*)-Tol-BINAP (**L2**), (*R*)-DM-BINAP (**L3**), (*R*)-DM-SEGPHOS (**L4**), and (*R*)-DTBM-SEGPHOS (**L5**)). These relatively electron-rich bisphosphines were selected in order to take advantage of their predicted favorable Gibbs free energy barrier for oxidative cyclization and because they have provided some enantioselectivity in the asymmetric Rh(I)-catalyzed PKR.^13,16,26,37−39^ In this case, the Rh(cod)_2_OTf precatalyst was reacted with each ligand in the presence of enyne **4a** or **6a**, in either THF or 1,2-dichloroethane (DCE) under a 10% concentration of CO in argon. The overall efficiency and selectivity of each ligand was determined by ^1^H NMR spectroscopy of the crude reaction mixture and HPLC of the purified product. Rh(cod)_2_OTf was chosen as the Rh(I) source for these reactions as it afforded the PKR products in the highest yields and % *ee* for nearly all substrates (see the counterion section below), with the exception of all-carbon tethered precursors. The concentration of CO was chosen to maximize the enantioselectivity of the PKR products.^16^ Both −NTs- and ether-tethered precursors were utilized in the ligand study given their distinct calculated dipole moments (5.47 and 1.1 D, respectively, EDF2/6-31G*) and their difference in size.

Reaction of enyne **4a** in the presence of ligands **L1**, **L2**, **L3**, and **L4**, provided product **5a** in high yields (96–99%) and good to excellent enantioselectivity (70–90% ee), with reaction times varying from 19 to 27 h (Table 1, entries 1–4). Utilizing ligand **L5**, the PKR of enyne **4a** was more sluggish, affording only a 39% yield of compound **5a** and 56% recovered starting material after 40 h (entry 5). In the latter case, steric repulsion between the *tert*-butyl groups of the chiral ligand and the enyne could be responsible for the low reaction efficiency based on the computed oxidative cyclization TS structures.

**Table 1:**
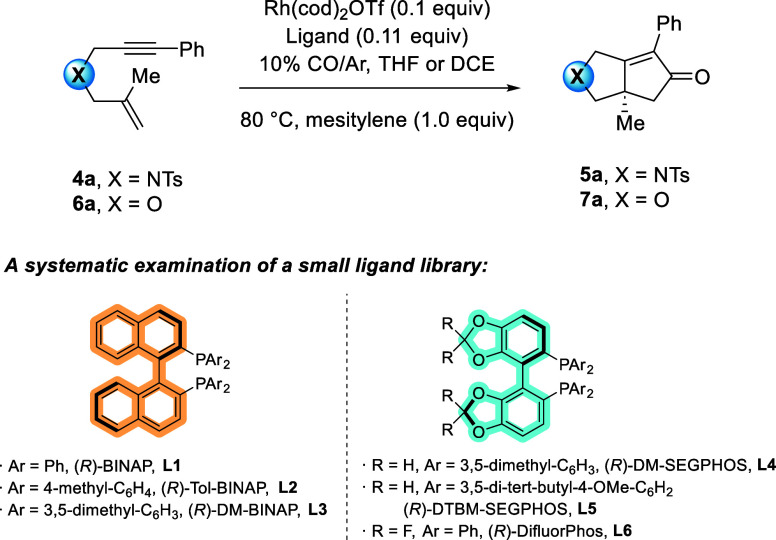
Evaluation of Ligands in the Asymmetric PKR of Precursors **4a** and **6a**

entry	ligand	time (h)	yield^a^ (%)	*er*^b^ (% ee)
X = NTs (**4a**), in THF			**5a**	
1	**L1**	22	99	95:5 (90)
2	**L2**	21	96	91:9 (82)
3	**L3**	19	96	89:11 (78)
4	**L4**	27	98	85:15 (70)
5	**L5**	40	39(56)^c^	93:7 (86)
6	**L6**	26	78 (22)^c^	97:3 (93)
X= O (**6a**), in DCE			**7a**	
7^d^	**L1**	23	84	94:6 (89)
8	**L2**	29	79 (5)^c^	83:17 (66)
9	**L3**	20	74	70:30 (40)
10	**L4**	17	62	74:26 (47)
11	**L5**	44	69 (24)^c^	89:11 (78)
12	**L6**	15	47 (23)^c^	93:7 (87)

a Yield determined by ^1^H NMR spectroscopy of the crude reaction in comparison to an internal standard (mesitylene, s, 6.78 ppm) to the product peak (d, 4.62 ppm of **5a**; d, 4.61 ppm of **7a**).

b ees determined by HPLC.

c Number in parentheses represents the yield of the recovered starting material, as determined by ^1^H NMR spectroscopy of the crude material.

d Reaction performed at 85 °C.

In the conversion of ether-containing precursor **6a**, ligands **L1**–**L4** gave moderate yields of bicyclic compound **7a** (62–84%) with a broader range of selectivity observed (40–89% ee) (Table 1, entries 7–10). Ligand **L5** still resulted in a slower reaction, now providing a 69% yield of product **7a** and 24% recovered starting material after 44 h (entry 11). It is important to note that the *er* of **5a** did not change over the course of the reaction, showing an *er* of 94:6 at 6 h and 95:5 at 21 h (Table S1). Similarly, for **7a**, the *ers* at 3 and 15 h were the same (93:7) (Table S2).

To identify parameters that are predictive of this experimentally observed enantioselectivity, we correlated the ln(*er*) observed for the reaction of enyne **4a** with ligands **L1**–**L5** with 181 DFT-computed descriptors for bisphosphine ligands complexed to Pd(II)Cl_2_ previously developed by Mack and Sigman.^40^ Although the computed TS structures of the Rh(I) bisphosphine enyne complex during the oxidative cyclization step show a twisted tetrahedral geometry, compared to the descriptor data representing a computed ground-state, square planar PdCl_2_/bisphosphine complex, these ligand parameters were successfully applied to a linear regression modeling of the Rh(I)-catalyzed hydroformylation reaction.^40^ The data was processed using MATLAB with the threshold set to *R*^2^ = 0.8 (see Supporting Information, Figure S3).

**Figure 2:**
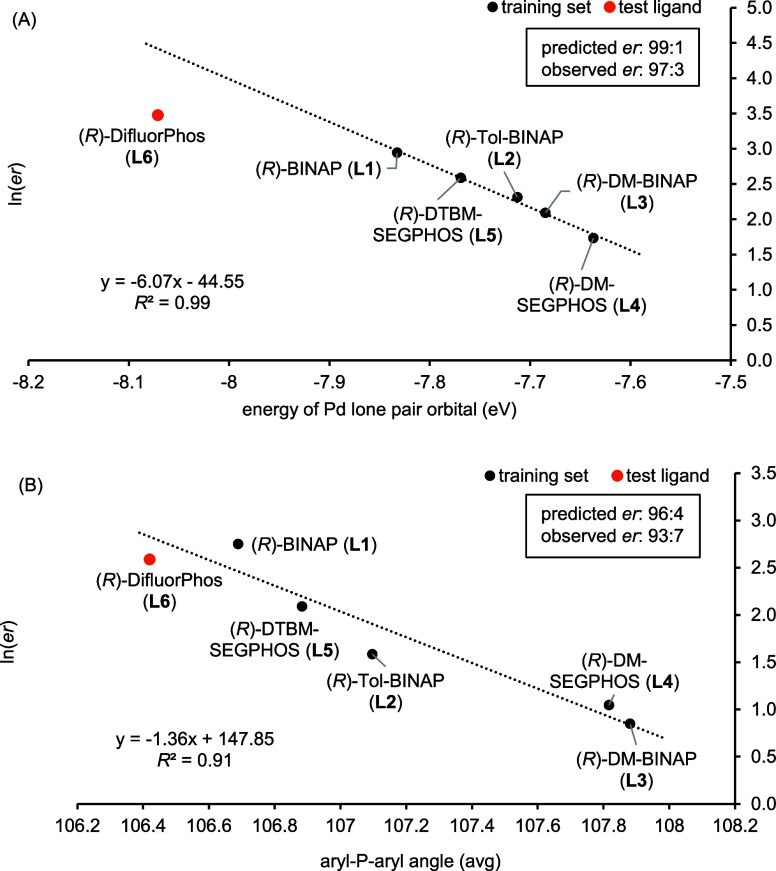
Correlation of ln(*er*) with the best-performing parameter. (A) ln(*er*) of **5a** with the energy of the Pd lone pair orbital (eV). (B) ln(*er*) of **7a** with an average aryl-*P*-aryl angle of the bisphosphine ligands.

For −NTs-tethered **4a**, seven parameters met this threshold, with the energy of the Pd lone pair orbital showing the strongest correlation for the training set (*R*^2^ = 0.99, Figures 2A and S3 in Supporting Information). The findings for ligand **L5** further support the observed electronic influence on PKR *er*; developing steric repulsions greatly decreases the rate of the PKR but has no impact on the observed enantioselectivity (entry 5). To evaluate the linear regression model, we tested (*R*)-DifluorPhos (**L6**), which has a lower energy of the Pd lone pair orbital compared to **L1**–**L5** (see Figure S5 in Supporting Information). As predicted by the model, the (*R*)-DifluorPhos (**L6**) ligand yielded an improved *er* (97:3) for **5a** (entry 6, Table 1) (Figure 2A).^38^ Similarly, the (*R*)-H8-BINAP (**LS1**) ligand has a predicted *er* of 89:11, and we observed an *er* of 90:10 (see Figures S7 and S8). To test a ligand that should afford a lower *er* for **4a**, we selected (*R*)-Xyl-GarPhos (**LS2**) as this ligand shows a higher energy of the Pd lone pair orbital and a larger aryl-P-aryl angle; however, this ligand yielded **5a** with an *er* of 91:9, which was higher than the predicted *er* of 83:17 (Figures S9 and S10). We also reacted **4a** with (*R*)-Xyl-MeOBIPHEP (**LS3**) as it shows a higher energy of the Pd lone pair orbital when compared to **L1**–**L5** (Figure S11). The experiment also showed a higher *er* than what the model predicted (*er*_predicted_ = 83:17, *er*_exp_ = 90:10, Figure S12). Thus, the single-parameter linear regression model cannot be extended to predicting *er*s for bisphosphine ligands having BIPHEP backbones. However, these findings inform our next steps in developing a comprehensive MLR model once we perform a broader systematic ligand study.

For enyne **6a,** the same seven parameters showed good correlations with ln(*er*) for the training set (Figure S14). In this case, the aryl-P-aryl angle (average) descriptor afforded the second highest correlation, featuring an *R*^2^ = 0.91 (Figure 2B). The energy of the Pd lone pair orbital descriptor also showed excellent linearity (*R*^2^ = 0.94; Figure S16). To test the model, we reacted **6a** with the (*R*)-DifluorPhos (**L6**) ligand, which afforded **7a** with an *er* of 93:7, which closely aligned with the predicted *er* of 96:4 (entry 12, Table 1) (Figure 2B). Although we did not observe a higher *er* using (*R*)-DifluorPhos (**L6**), the model shows the general predictability as the smaller aryl-P-aryl angle featured in (*R*)-DifluorPhos (**L6**) gives a better *er* when compared to four of the five ligands in our training set (**L2**–**L5**), which all have larger aryl-P-aryl angles.

The fact that the set of parameters is the same, but the fit changes between substrate classes is likely related to the varying interactions between the electronic and steric properties of the ligands and substrates. For electron-poor *N*-tosylated enynes, such as compound **4a**, the enantioselectivity is more sensitive to the electronic properties of the ligand. In contrast, for less electron-poor substrates such as ether **6a**, the steric nature of the ligand also exerts influence on the diastereomeric transition states, resulting in wider range of enantioselectivity being observed.^38^

**Figure 3:**
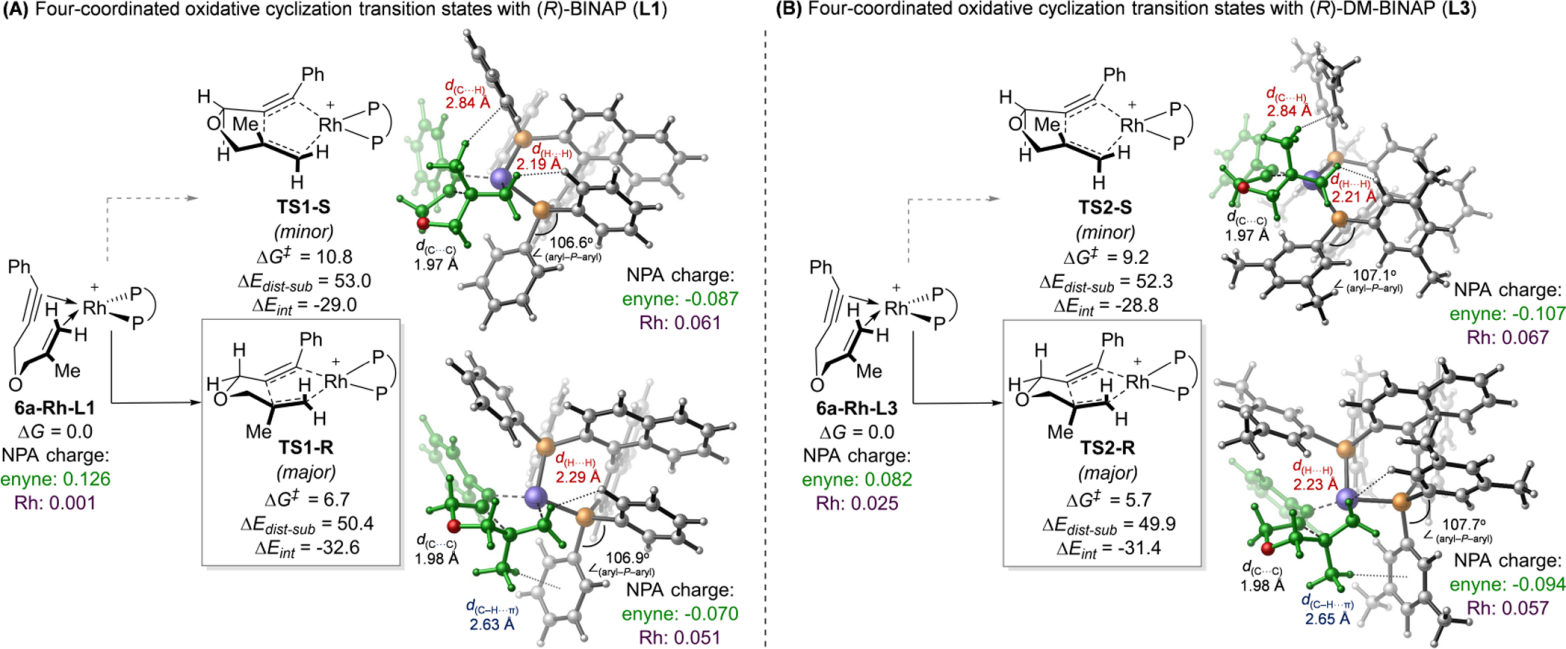
Lowest-energy transition state structures of oxidative cyclization of enyne **6a** with (*R*)-BINAP (**L1**) and (*R*)-DM-BINAP (**L3**) ligands. All energies are given in kcal/mol.

Given the high performance of this five-data-point model, DFT calculations were performed to understand the enantio-determining oxidative cyclization of ether **6a** with the (*R*)-BINAP (**L1**) and (*R*)-DM-BINAP (**L3**) ligands, as they produced the most significant difference in the % ee in product **7a** (89% vs 40% ee, respectively).^41^ With the (*R*)-BINAP-supported Rh catalyst, the oxidative cyclization TS leading to the (*R*) product (**TS1-R**) is favored over the TS leading to the minor (*S*) product (**TS1-S**) by 4.1 kcal/mol (Figure 3A). Similar to previous studies,^16^ the selectivity between π faces of the alkene directly impacts the steric interactions of the terminal alkene hydrogens with the chiral ligand. In **TS1-S**, the shorter H···H distance between a terminal alkenyl hydrogen and a hydrogen atom on the *P*-Ph group on the ligand (2.19 Å), relative to **TS1-R** (2.29 Å), causes more substrate distortion (Δ*E*_dist-sub_ = 53.0 vs 50.4 kcal/mol).^42^ Additionally, **TS1-R** is stabilized through a C–H/π interaction between the alkene methyl hydrogens and one of the (*R*)-BINAP (**L1**) phenyl groups (2.63 Å, Figure 3A). This additional stabilization is reflected in a lower substrate–catalyst interaction energy (Δ*E*_int_) in **TS1-R** relative to **TS1-S** (−32.6 vs–29.0 kcal/mol).

The computed transition states with (*R*)-DM-BINAP (**L3**) share many of the structural features of those with (*R*)-BINAP (**L1**) (Figure 3B). For example, **TS2-S** is 3.5 kcal/mol less stable than **TS2-R** due to the steric clash between the terminal alkene hydrogens and the chiral ligand (2.21 vs 2.23 Å in **TS2-R**), leading to an increase in substrate distortion (52.3 vs 49.9 kcal/mol in **TS2-R**). (*R*)-DM-BINAP also undergoes a stabilizing C–H/π interaction between the alkene methyl group and the phosphine aryl group in **TS2-R** (2.65 Å), leading to a lower Δ*E*_int_ (−31.4 vs −28.8 kcal/mol).

Although the computed enantioselectivity (ΔΔ*G*^‡^) is higher than experimental observations with both ligands, an overestimation also observed in previous computational studies,^16^ computations predicted that the reaction with (*R*)-DM-BINAP is less enantioselective than that with (*R*)-BINAP (ΔΔ*G*^‡^ = 4.1 and 3.5 kcal/mol), which is consistent with the experimental ligand effect trend. The enantioselectivity difference could be attributed to both steric and electronic effects. Sterically, the larger aryl-P-aryl angle between the two *P*-aryl groups on (*R*)-DM-BINAP (107.7° in **TS2-R** vs 106.9° in **TS1-R**) effectively positions the *P*-aryl groups further away from the terminal alkenyl group on the substrate, minimizing ligand–substrate steric repulsions, as evidenced by the longer H···H distance in **TS2-S** relative to **TS1-S** (2.21 vs 2.19 Å). This explains the somewhat surprising observation that the ligand aryl-P-aryl angle descriptor strongly correlates with the ln(*er*) of the reaction (Figure 2B). Electronically, both TSs leading to the minor enantiomer (**TS1-S** and **TS2-S**) undergo a higher degree of metal-to-substrate charge transfer relative to the major TSs. Therefore, stronger donor ligands, such as (*R*)-DM-BINAP, are expected to better stabilize the minor TS, leading to a decrease in the % ee compared to more electron-deficient ligands. This further demonstrates why electronic descriptors, such as the energy of the Pd lone pair orbital, also correlate well with the experimentally observed ln(*er*).

**Figure 4:**
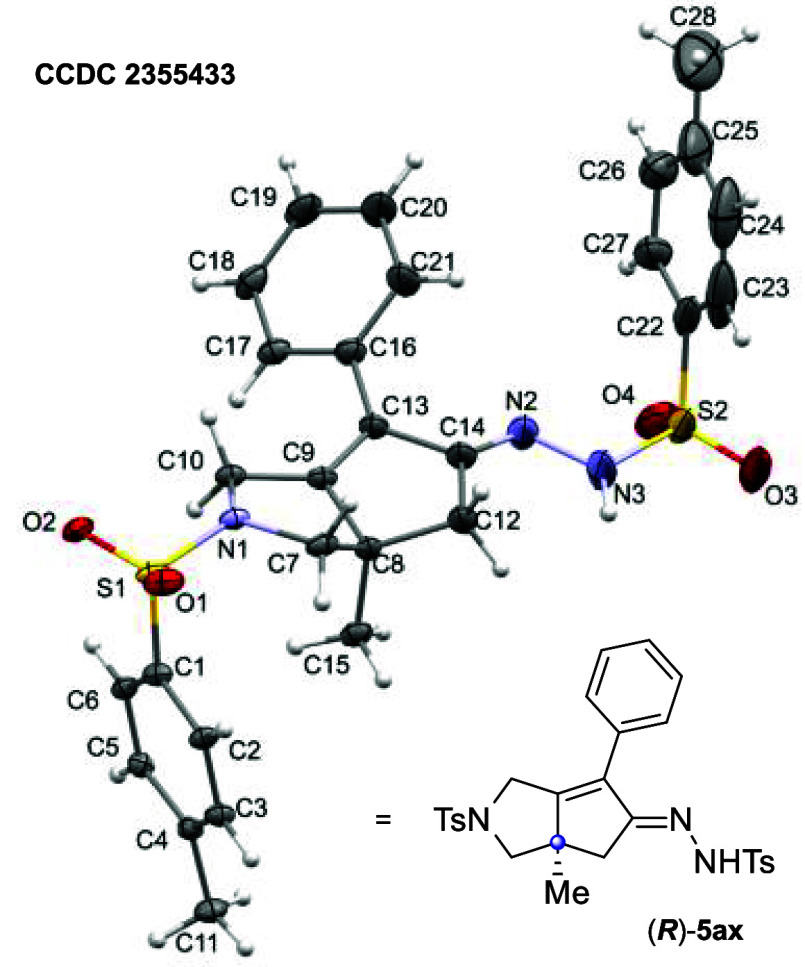
Single-crystal X-ray structure of hydrazone (*R*)-**5ax**.

The absolute configuration of (*R*)-**5ax**, using single-crystal X-ray crystallography, provides further support for our computational predictions (Figure 4). Ketone **5a** (93:7 *er*) was initially converted to the hydrazone (*R*)-**5ax** using *p*-toluenesulfonyl hydrazide under acidic conditions (see Supporting Information). Slow vapor diffusion of methanol into deuterated chloroform provided an X-ray-quality crystal of **5ax**, from which X-ray analysis confirmed that the bridgehead carbon bears the (*R*) configuration, matching the major enantiomer predicted by DFT.

### Identification of Solvent Parameters Correlated to the PKR Yield and Enantioselectivity

NTs- and ether-tethered precursors **4a** and **6a** were evaluated for the solvent study, given the differences in yield and side-product formation observed in an initial solvent study using Rh(cod)_2_BF_4_ (see Supporting Information). Ether-tethered **6a** is a model substrate for the asymmetric PKR,^18,20,24^ whereas the asymmetric PKR of enyne **4a** had not yet been realized prior to this work. Our solvent selection was guided by previous mechanistic work. We focused on solvents that had been successfully used previously in the Rh(I)-catalyzed PKR and that offered a range of coordinating abilities, based on the dipole moment (see Table S4 in Supporting Information). Eleven different solvents with dipole moments ranging from 0 to 2.86 D were selected: THF, DCE, chloroform, chlorobenzene, trifluorotoluene, ethyl acetate, toluene, ethanol, 1,4-dioxane, trifluoroethanol, and dimethyl carbonate. These solvents also showed a range of Abraham’s hydrogen bond basicity values (0.02–0.64), another measure of the coordinating ability,^43^ and a range of dielectric constants (2.219–27.68). Solvents previously used in PKRs—dibutyl ether, xylenes, and acetone—were not included due to technical difficulties associated with high and low boiling points.

Enyne **4a** was reacted under PKR conditions with each solvent twice and the average yield and % ee of product **5a** were used for correlations. Enyne **6a** was reacted with each solvent only once. Each reaction was conducted with Rh(cod)_2_OTf (10 mol %), (*R*)-BINAP (11 mol %), 10% CO atmosphere/argon, 80 °C, solvent (0.05 M), and mesitylene (1.0 equiv) as an internal standard. An initial time point measurement was performed by ^1^H NMR spectroscopy to establish the concentration of enyne **4a** (s, 4.25 ppm) or **6a** (s, 4.36 ppm), relative to the mesitylene (s, 6.78 ppm). A second measurement was performed at 24 h to determine the overall product yield (d, 4.62 ppm for **5a** and d, 4.61 ppm for **7a**), the yield based on the recovered starting material (brsm), and the % ee (purified by prep TLC then % ee by HPLC) (see Tables S5 and S6 in Supporting Information).

The results for the −NTs-tethered precursor **4a** are reported in Table 2. Solvent dipole moments and dielectric constants are plotted against yields, brsm, and ln(*er*) (Figures S27–S32). In doing so, the solvents with the highest dipole moment were observed to afford the highest selectivity.

**Table 2:**
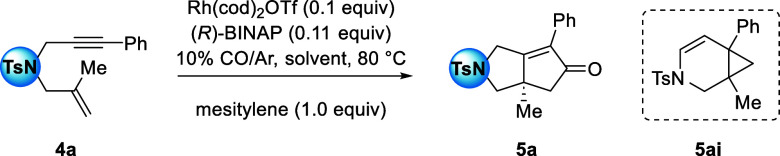
Solvent Study Performed on Enyne Precursor **4a** Using Rh(cod)_2_OTf

entry	solvent	yield^a^ (**5ai**)^b^ %	brsm	%ee^a^^,^^c^**5a**
1	THF	99	99	90
2	DCE	77 (6)	93	86
3	ethyl acetate	23 (trace)	94	88
4	trifluorotoluene	46 (trace)	82	82
5	trifluoroethanol	25 (4)	72	89
6	toluene	28 (5)	66	78
7	ethanol	35	71	89
8	chlorobenzene	32 (2)	73	66
9	1,4-dioxane	31 (2)	77	76
10	chloroform	65	89	94
11	dimethyl carbonate	48 (2)	90	83

a Average of two experiments; determined by comparing an internal standard (mesitylene: s, 6.78 ppm) to the product peak (d, 4.62 ppm) by ^1^H NMR spectroscopy of the crude material.

b Cycloisomerization side-product yield.

c ee determined by HPLC.

**Figure 5:**
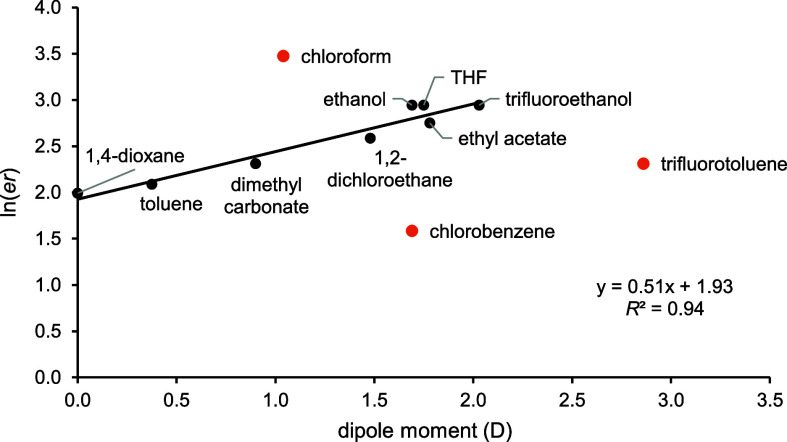
Correlation of ln(*er*) of product **5a** with solvent dipole moments (D). Outliers are indicated with red dots.

The dipole moment showed a positive linear correlation with ln(*er*) of the product **5a** having an *R*^2^ = 0.94 for eight of the 11 solvents (Figure 5). The outlier solvents include trifluorotoluene, chlorobenzene, and chloroform. For halogenated aromatic solvents, arene complexes of the cationic Rh may occur, altering the energy difference between the diastereomeric oxidative cyclization TSs. Similarly, with the lowest boiling point of the set, PKRs in chloroform occur at a lower temperature (61.2 °C) than other solvents (all other reactions were heated to 80 °C), resulting in a higher kinetic selectivity.^44^ Regarding chlorobenzene, analysis of the experimental solvent π* and Taft β values, in relation to the PKR % ee*,* revealed a cluster of solvents associated with low enantioselectivity (1,4-dioxane, toluene, chlorobenzene) (see Figure S38 in the Supporting Information).

In comparison, ether **6a** reacted to yield product **7a** with higher selectivity in halogenated solvents, with DCE and trifluorotoluene affording the highest enantioselectivity (87–89% ee) (Table 3). Conversely, THF, dimethyl carbonate, ethyl acetate, dioxane, and ethanol yielded a lower selectivity.

**Table 3:**
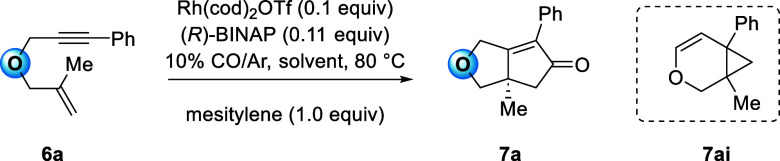
Solvent Study Performed on Enyne Precursor **6a** Using Rh(cod)_2_OTf

entry	solvent	yield^a^ (**7ai**)^b^ %	brsm	%ee^c^**7a**
1	THF	52	78	69
2	DCE^d^	84 (13)	85	89
3	ethyl acetate	40	53	69
4	trifluorotoluene	26 (15)	55	87
5	trifluoroethanol	80	81	85
6	toluene	0 (4)	0	N/A
7	ethanol	52	53	69
8	chlorobenzene	22 (3)	67	84
9	1,4-dioxane	19 (4)	36	59
10	chloroform	trace	0	N/A
11	dimethyl carbonate	32	39	56

a Yield determined by comparing an internal standard (mesitylene: s, 6.78 ppm) to the product peak (d, 4.61 ppm) by ^1^H NMR spectroscopy of the crude material.

b Cycloisomerized side-product yield.

c ee determined by HPLC.

d Reaction performed at 85 °C.

**Figure 6:**
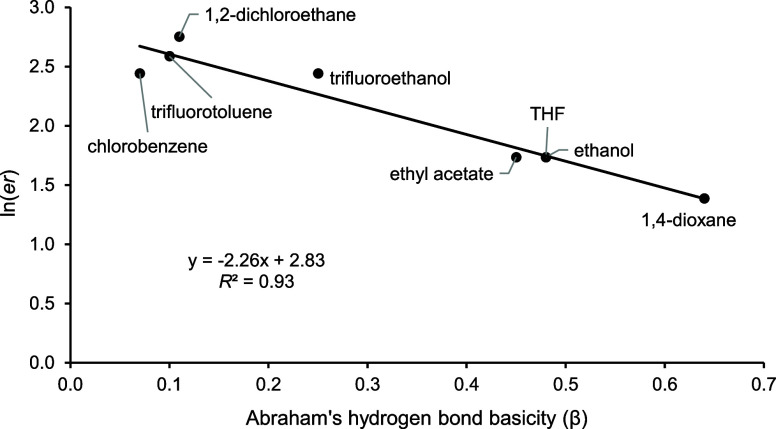
Correlation of ln(*er*) of ether product **7a** with Abraham’s hydrogen bond basicity of the solvents.

Abraham’s hydrogen bond basicity (β), a solvent parameter that describes the ability of a solvent to act as a hydrogen bond acceptor,^45^ shows a positive correlation with PKR ln(*er*) of ether product **7a** for 8 of 11 solvents tested (*R*^2^ = 0.93) (Figure 6). Toluene and chloroform were not included in the regression analysis, as no PKR product was afforded after 24 h. Dimethyl carbonate was also excluded as no β value was identified. Solvent π* values, another measure of solvent polarity, were plotted against the ln(*er*) of product **7a** and found to exhibit a moderate correlation (*R*^2^ = 0.80) for 8 of the 11 solvents (π* was not identified for dimethyl carbonate; Table S4 and Figure S50).

This study shows that solvents strongly influence PKR outcomes (e.g., % ee and yield) and, to a lesser extent, side-product formation. We attribute the variable solvent effectiveness for these two substrate classes to favorable electrostatic interactions between the enyne (*D* = 5.47 for **4a** and 1.1 for **6a**) and the solvent in the oxidative cyclization step. While these data support the fact that a single solvent parameter can be used to predict enantioselectivity for a distinct PKR substrate, the multifaceted nature of PKR as indicated by the solvent outliers and substrate dependency on solvent selection supports future studies directed toward using multivariate regression models (MLRs) for the selection of parameters. Correlating selectivity data for **5a** with Katritzky’s solvent database showed low linearity (Figure S39).^46^

### Identifying Counterion Descriptors That Correlate to the Rh(I)-Catalyzed PKR Yield and Enantioselectivity

We then briefly examined the effect of three different Rh(I) precatalysts (e.g., Rh(cod)_2_BF_4_,^12,47^ Rh(cod)_2_SbF_6_,^16^ and Rh(cod)_2_OTf^48^) to determine their impact on the enantioselectivity and yield for six enyne precursors featuring different tethers and alkyne substituents. These precatalysts have been successfully used in the asymmetric PKR previously and differ only in their counterion, which is known to be a key factor in controlling the PKR catalyst efficiency.^14^ In all cases, (*R*)-BINAP was used as the chiral ligand, as it afforded overall the highest yield and high ee% among the six bisphosphine ligands examined above. The reactions were performed using a 10% CO/argon atmosphere with mesitylene as an internal standard, and the reaction temperature (60–100 °C) was selected based upon the reaction rate. The solvent used for these reactions was chosen based on our solvent study. All reactions were allowed to proceed to completion or stopped when there was no visible progress.

Our experiments show that the counterion has a strong influence on the yield and %ee of the product. For example, reaction of **4a** in THF gives **5a** in >90% yield and no side-product **5ai**, regardless of the counterion (Table 4, entries 1–3). Conversely, the reaction of **4a** in DCE gives **5a** in 63 and 68% yields and **5ai** in 7 and 17%, respectively (entries 4–5). For **4b**, counterions BF_4_^–^ and OTf^–^ gave lower yields (44 and 71%, respectively) due to the reaction not proceeding to completion (entries 6–7). The reaction of ether **6a** was more sensitive to the catalyst counterion, giving **7a** in >84% yield for OTf^–^ and SbF_6_^–^, but only 54% yield for BF_4_^–^. Regardless of the counterion, reactions of ether **6a** resulted in the formation of side-product **7ai** (entries 8–10). When the reaction is performed in THF, instead of DCE, the reaction is sluggish, giving **7a** in low to moderate yields (entries 11–12). Reacting precursor **6b** in DCE using BF_4_^–^ afforded **7b** in 35% yield along with a 55% yield of **7bi** (entry 13). However, counterion OTf^–^ gave a 66% yield of **7b** and 25% yield of **7bi** (entry 14). Reaction of **8b** provided **9b** in 93% yield with the BF_4_^–^ counterion and 67% yield for OTf^–^ (entries 15–16). Similarly, acetal **10a** gives 81% yield with BF_4_^–^ but only 34% yield for OTf^–^ (entries 17–18).

**Table 4:**
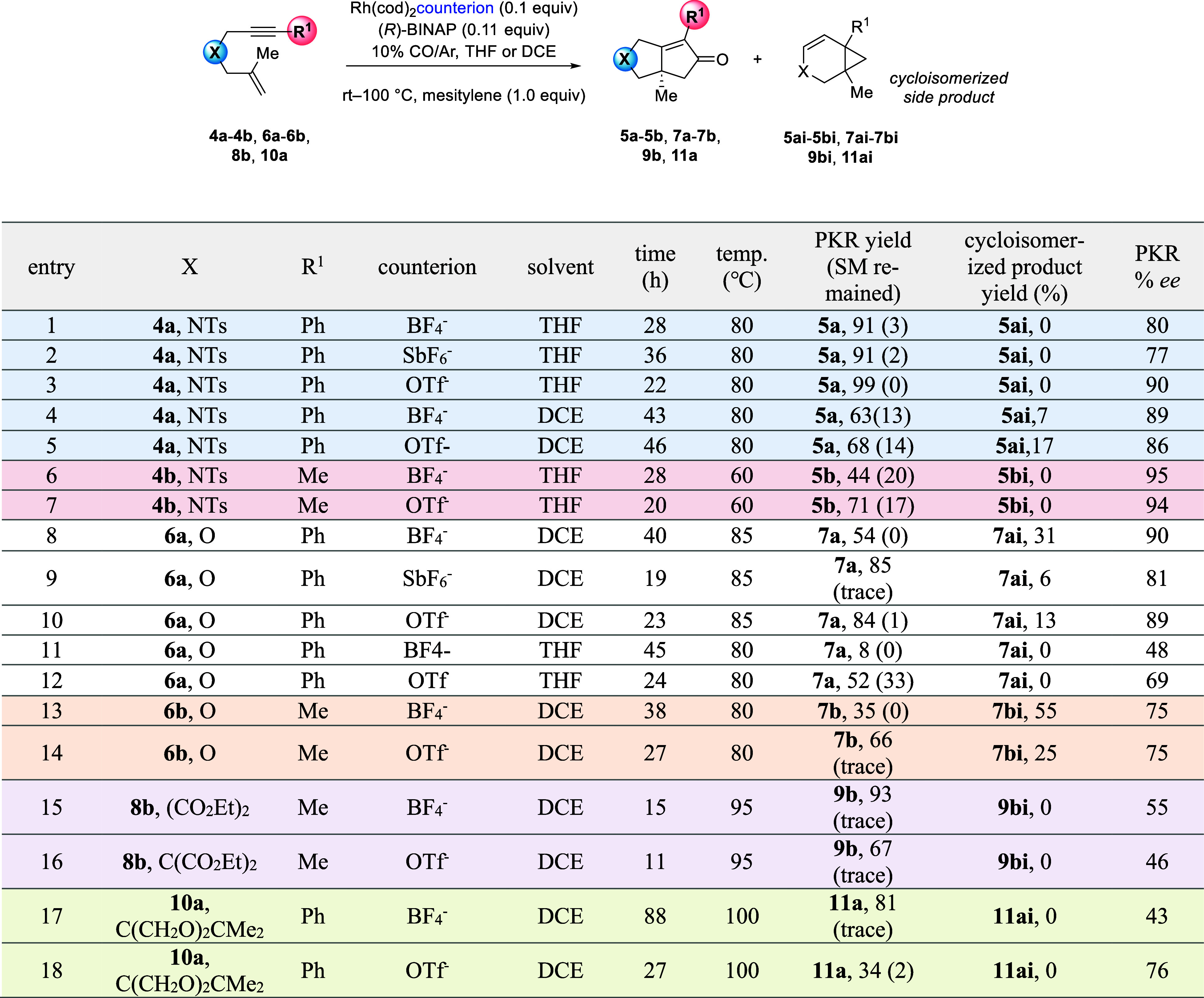
Counterion Effect on Selected Enyne Precursors Featuring Different Tether and Alkyne Substituents

For all enynes, the PKR was faster when using Rh(cod)_2_OTf. Consiglio and Schmid have shown that the degree of ion-pairing for [Rh(1,5-cod)Biphemp]counterion, where counterion = BF_4_^–^, PF_6_^–^, and OTf^–^, is inversely correlated with the catalytic activity for the asymmetric PKR. For example, they showed that using [Rh(1,5-cod)Biphemp]OTf, which has the lowest ion-pairing, has the highest catalytic activity. Our findings, using six different enynes and two solvents, show that this is a general principle of the asymmetric PKR.^49^

The strong influence that the counterion has on the yield is dependent upon the substrate and solvent. For example, when using DCE, we observed lower yields for ether tethers (entries 8–10, 13–14), which can be attributed to the formation of side-products **7ai** and **7bi** in 6–55% yield. We observe the formation of this side-product only in the more polar solvent, DCE (ε = 10.42), which favors a dissociated ion pair and a more electron-deficient Rh(I)–alkyne complex, relative to the complex in a less polar solvent THF (ε = 7.52).^15^ This leads to a reaction energy profile that favors a [1,2]-hydrogen shift (**II** → **III**, Figures S52 and S53)-promoted cycloisomerization reaction over the oxidative cyclization of the PKR (**IV** → **V**, Figure S54). The all-carbon tethers are an exception to this trend, as the [1,2]-hydrogen shift is inaccessible. Others have shown that the formation of cycloisomerization side-products requires a heteroatom in the tether when using Pt or Au catalysis.^50^

Regarding the impact of the counterion on the reaction enantioselectivity, in general, higher enantioselectivities were observed for enyne precursors featuring −NTs tethers (**4a**) and ether tethers (**6a**) when using Rh(cod)_2_OTf. Reactions employing Rh(cod)_2_SbF_6_ gave yields similar to Rh(cod)_2_OTf, but with somewhat lower selectivity (entries 2 and 9). However, for malonate (**8b**) and acetal (**10a**) tethers, low *ees* were observed for both Rh(cod)_2_BF_4_ and Rh(cod)_2_OTf (43–76% ee, entries 15–18).

### Identification of Substrate Descriptors Correlated to the PKR Yield and Enantioselectivity

**Scheme 1:**
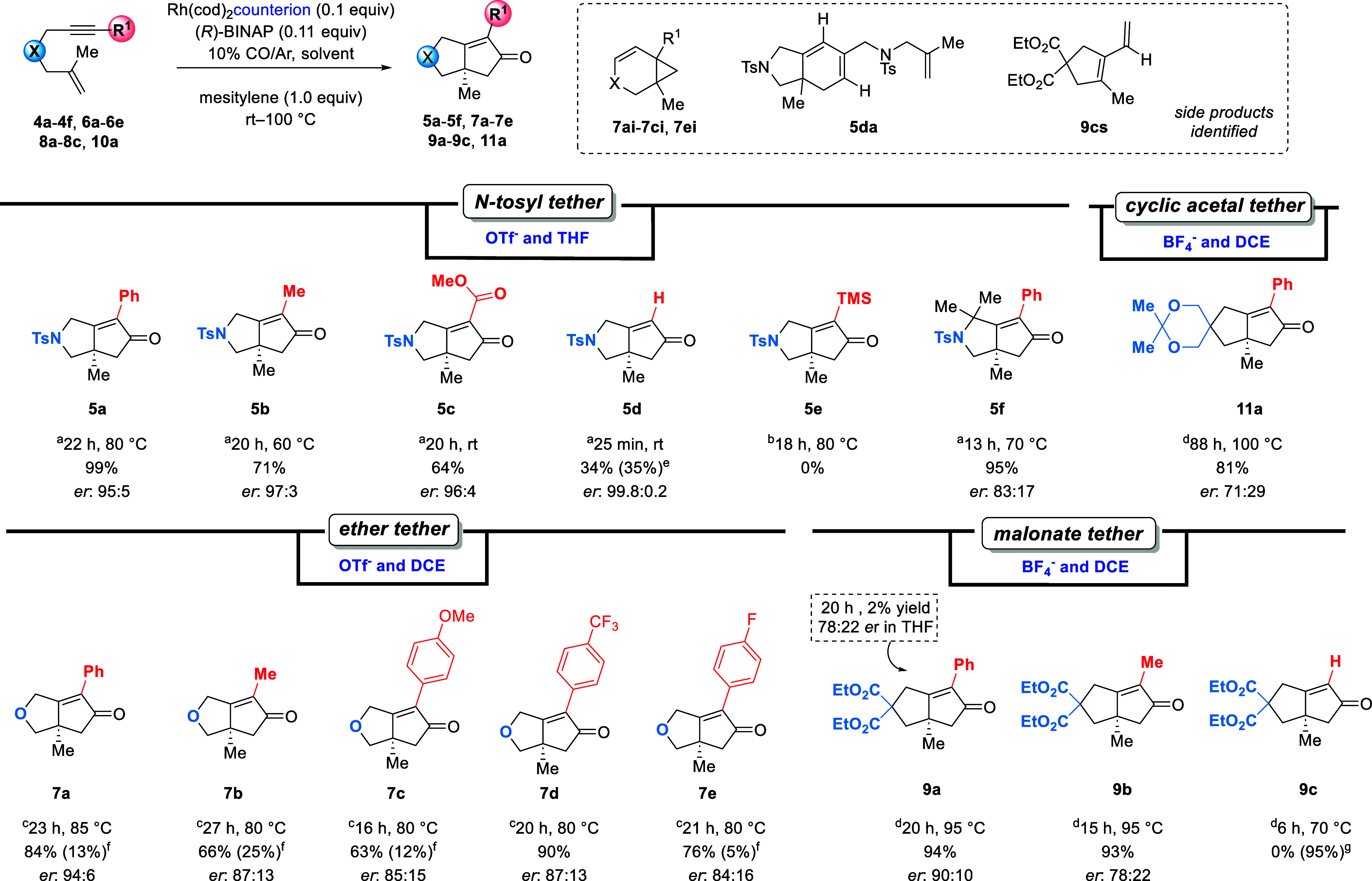
Current Scope of the Asymmetric PKRs Rh(cod)_2_OTf (0.1 equiv) in THF (0.05 M). Rh(cod)_2_BF_4_ (0.1 equiv) in THF (0.05 M). Rh(cod)_2_OTf (0.1 equiv) in DCE (0.05 M). Rh(cod)_2_BF_4_ (0.1 equiv) in DCE (0.05 M). Yield of the [2 + 2 + 2] cycloaddition side-product **5da**. Yields of the cycloisomerized side-products. Yield of the side-product **9cs**.

To determine the effects of the substrate structure on the asymmetric Rh(I)-catalyzed PKR, enynes containing different tethers (X = NTs, O, C(CO_2_Et), and acetal) and alkyne substituents (R^1^ = Ph, aryl, CO_2_Me, Me, H, TMS) were evaluated. The enyne alkenyl group was held constant. The PKR conditions used for each substrate were based upon our findings in the chiral ligand, solvent, and counterion studies reported above. The PKR products afforded, in addition to reaction yields, % ee, reaction time, and side-products, for a range of heteroatoms and carbon-tethered 1,6-enynes with 2,2-disubstituted alkenes are depicted in Scheme 1.

Our experiments show that the alkynyl substituent had the strongest influence on the PKR yield and reactivity. For enynes having an −NTs tether, yields ranged from 0 to 99% (Scheme 1, **5a**–**5e**). Among these enynes, the phenyl-substituted alkyne gives **5a** in 99% yield, the methyl- and methyl ester-substituted alkynes give **5b** and **5c** in 71 and 64% yields, and the terminal alkyne gives **5d** in 34% yield. Unlike substituted enyne precursors, the dimer **5da** was obtained in 35% yield when the unsubstituted terminal alkyne underwent asymmetric PKR. For the TMS-substituted enyne, only recovered starting material was observed after 18 h (**5e**). Additional methyl groups in the tether, likely due to the Thorpe–Ingold effect, accelerated the reaction to give product **5f** in 13 h compared to the 22 h required for complete conversion to product **5a**.^51^ For −NTs-tethered substrates, high selectivities were observed for the terminal alkyne giving **5e** with an *er* of 99.8:0.2, and the phenyl- methyl- and methyl ester-substituted alkynes giving **5a**, **5b,** and **5c** in 97:3 to 95:5 *ers*. An exception is the enyne having additional methyl groups in the tether, giving **5f** in 83:17 *er*.

For ether-tethered enynes, 63–90% yield was observed (**7a**–**7e**). Among these enynes, the electron-withdrawing phenyl- and trifluoroaryl-substituted enynes give **7a** and **7d** in 84 and 90% yields. The electron-donating fluoro- and methoxy-aryl groups give 63 and 76% yields of **7c** and **7e** and for the methyl-substituted alkyne, **7b** was afforded in 66% yield along with significant quantities of **7bi**. For the selectivities of the ether-tethered substrates, with the exception of **7a**, the *er* values were constant, ranging from 84:16 to 87:13. For the malonate-tethered enynes, the phenyl- and the methyl-substituted alkynes give **9a** and **9b** in 94 and 93% yield, having *ers* of 90:10 and 78:22, respectively. The terminal alkyne gives only the side-product **9cs**.

Conversely, the nature of the tether has the greatest influence on selectivity, with the highest % ee observed for precursors with −NTs tethers (90–99.6% ee), with the exception of the *gem*-dimethyl product **5f**. Selectivity was more moderate for ether-tethered enynes (69–89% ee) and low to moderate for the all-carbon tethered enynes (43–80% ee). The yield and selectivity patterns observed with these 2,2-disubstituted alkene enynes are similar to those observed previously for enynes with a monosubstituted alkene.^39^

**Figure 7:**
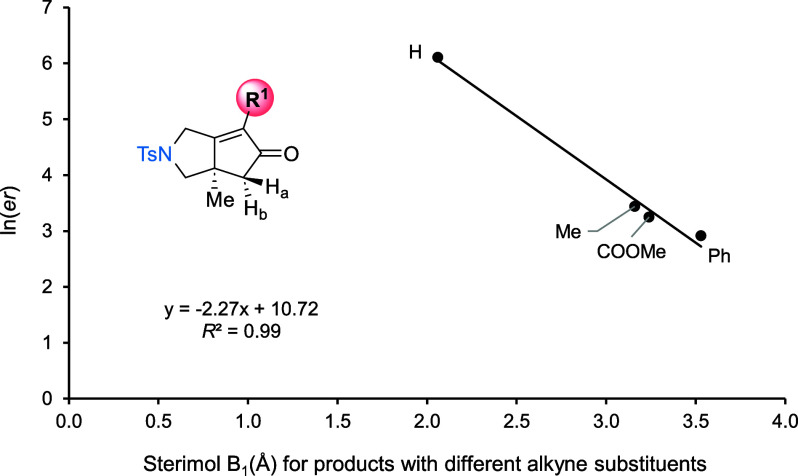
Correlation of ln(*er*) with the calculated Sterimol B_1_ (Å) for products **5a–5d**.

For −NT-tethered substrates, a range of selectivities were observed. To quantify the steric effect, we plotted Sterimol values (B_1_, B_5_, and L) for the lowest-energy conformers of the PKR products **5a**, **5b**, **5c,** and **5d** against their ln(*er*). The PKR products were selected for Sterimol analysis due to their rigidity and structural similarity to the oxidative cyclization transition state—the enantio-determining step of these PKRs. Sterimol B_1_ (Å) values, with the primary axis along the H_b_–C bond, show a strong linear relationship (*R*^2^ = 0.99) with PKR ln(*er*), with smaller B_1_ values giving higher ln(*er*) (Figure 7). Sterimol B_5_ gives a moderate correlation (*R*^2^ = 0.80) and Sterimol L gives a good correlation (*R*^2^ = 0.93) with ln(*er*) (see Figures S56 and S57 in Supporting Information). Using (*R*)-BINAP as a chiral ligand, our experimental ln(*er*) shows a linear free energy relationship (*R*^2^ = 0.87) with the computed ΔΔ*G*^‡^ for the oxidative cyclization step reported by Baik and Evans using (*S*)-Xyl-SEGPHOS as the chiral ligand (see Figure S58 in Supporting Information).^26^

For the ether tether, electronic effects of the phenyl-substituted alkyne showed product **7a** having the highest % ee, while **7c**, **7d**, and **7e** containing either electron-donating or electron-withdrawing groups formed with lower selectivity. The mass balance for these substrates was high, with varying quantities of cycloisomerized products (5–13%) observed. We attribute the formation of this side-product mainly to the DCE solvent and counterion, as discussed above. Subjecting the malonate-tethered precursor **8a**, which features a similar dipole moment (*D* = 1.85) to the ether tethered precursor **6a** (*D* = 1.1), to the PKR conditions in THF gave **9a** in low yield (2%) and low *er* (78:22). As a comparison, performing the same reaction in DCE afforded **9a** in 94% yield and an *er* of 90:10, thus demonstrating that the computed enyne dipole moment serves as a guide for selecting a PKR solvent. The cyclic-acetal-substituted product **11a** was formed in 81% yield, albeit with only moderate selectivity (43% ee). However, the unsubstituted terminal alkyne afforded only side-product **9cs**; the desired product **9c** was not observed. We also found that carbon-tethered enynes required elevated temperatures (95–100 °C), relative to the heteroatom tethers (rt–85 °C), likely due to their lower reactivity, which has been explained by a distortion–interaction analysis involving the relatively planar geometry of the outer five-membered ring in the PKR oxidative cyclization step.^26^

To quantify the effect of the alkynyl group on the yield, we computed the IR C≡C bond wavenumber for each enyne precursor.^52^ Precursors that underwent PKR in moderate to high yields consistently show an alkyne IR wavenumber exceeding 2250 cm^–1^. Conversely, for precursors with an alkyne wavenumber ≤2203, low yields or no reaction were observed (Table S10).

## Conclusions and Future Outlook

It is surmised that the insights from this work can inform the design of catalytic systems based on tether variations. First, for enynes featuring heteroatoms with high dipole moments, the asymmetric PKR is anticipated to be more efficient when utilizing a combination of bisphosphine ligands with electron-deficient phosphorus donor atoms, a rhodium catalyst exhibiting a low degree of ion-pairing (e.g., OTf^–^), and a suitably coordinating solvent such as THF. Second, for enynes with heteroatoms that have low calculated dipole moments, the transformation benefits from ligands displaying a small aryl-P-aryl angle, in conjunction with a rhodium catalyst characterized by low ion-pairing and a halogenated, noncoordinating solvent such as DCE. Finally, for 1,6-enynes with carbon tethers and low dipole moments, the reaction is more compatible with a rhodium catalyst that possesses a higher degree of ion-pairing (e.g., BF_4_^–^) and in a noncoordinating halogenated solvent. The ligand insights gained from the heteroatom-tethered enynes can be used to achieve high yield and enantioselectivity for these carbon-tethered enynes. Overall, enynes with reasonably high C≡C wavenumbers (≥2203 cm^–1^) are expected to be more compatible with the current catalytic system.

In conclusion, we achieved an asymmetric Rh(I)-catalyzed PKR for 1,6-enynes having 2,2-disubstituted alkenes, allowing access to 13 new chiral nonracemic bicyclo[3.3.0]octenone products having a quaternary carbon at the ring fusion. In the process, two descriptors for the chiral bisphosphine ligand were identified that are strongly correlated with PKR ln(*er*). In addition, catalysts that are predicted to have the weakest ion-pairing, as determined by their counterion, provided the fastest reactions and the highest yields for heteroatom-tethered enynes. Further, the dipole moment (*D*) and Abraham’s hydrogen bond basicity (β) of the solvent show a strong correlation with PKR ln(*er*). Finally, the calculated Sterimol B_1_ values correlate with ln(*er*), and the IR C≡C bond wavenumbers correlate with the reaction efficiency for variously substituted enyne PKR precursors. These findings provide useful information for our high-throughput experimentation (HTE) studies to be performed in collaboration with Merck & Co. Inc. In turn, we expect that the combination of these initial data collection studies with statistical analysis of HTE data will facilitate the development of a predictive model (e.g., MLR) for a catalyst-controlled Rh(I)-catalyzed asymmetric PKR of enynes. Such a framework will enable the rational selection of appropriate chiral catalysts and reaction conditions for a particular enyne in order to give the PKR product in high yield and high enantioselectivity.

## Supporting Information Available

Experimental details, spectral data of the new compounds, and HPLC analytical data (PDF)
Computational details, energies, and Cartesian coordinates of all reported structures (PDF)
Crystallographic data of (*R*)-5ax (CIF)
(ZIP)
Crystallographic data of 5ai (CIF)

## Supplementary Material

cs4c04490_si_002.pdf

cs4c04490_si_003.cif

cs4c04490_si_004.zip

cs4c04490_si_005.cif
