# Supplementary material for: Systematic Parameter Determination Aimed at a Catalyst-Controlled Asymmetric Rh(I)-Catalyzed Pauson–Khand Reaction
Source: ACS Catal. 2024 Nov 5;14(22):17065–76. doi: 10.1021/acscatal.4c04490 (PMC11574763; doi:10.1021/acscatal.4c04490)
Supplement: Supplementary file 1 — cs4c04490_si_001.pdf [file cs4c04490_si_001.pdf]

Systematic Parameter Determination Aimed at a Catalyst-controlled Asymmetric Rh(I)-Catalyzed Pauson-Khand Reaction

Yifan Qi, Luke T. Jesikiewicz, Grace E. Scofield, Peng Liu*, and Kay M. Brummond*

Department of Chemistry, University of Pittsburgh, Pittsburgh, Pennsylvania 15260, United States

Corresponding authors: kbrummon@pitt.edu; pengliu@pitt.edu

Table of Contents

General experimental methods 2

Synthesis of 1,6-enyne precursors 3

Asymmetric Pauson-Khand Reactions 25

Chiral Bisphosphine Ligand Study 56

First-Generation Solvent Study Using $Rh(cod)_2BF_4$ and Enyne 4a 79

Second-Generation Solvent Study Using $Rh(cod)_2OTf$ and Enyne 5a and 7a 86

Rh(I) Precatalysts 103

Proposed Mechanisms for the Cycloisomerization and PKR 108

Substrate Study–Alkynyl group 111

Substrate Study–Tether 114

Calculated IR C=C and C≡C bond wavenumbers (cm^{-1}), intensities, and dipole moments for selected enyne precursors (EDF2/6-31G* corrected values reported, Spartan'20) 114

Hammett parameters (σ_p) and ether-tethered PKR products 7a, 7c, 7d and 7e 115

Spectral Data for Solvent Study Using $Rh(cod)_2OTf$ and NTs-Tethered Precursor 4a 117

Spectral Data for Solvent Study Using $Rh(cod)_2OTf$ and Ether-tethered Precursor 6a 147

NMR spectra data for Counterion Study in Table 4 163

X-Ray Crystallography Information for (*R*)-5ax and 5ai 186

^{1}H and ^{13}C NMR spectra 203

References 246

General experimental methods

Unless otherwise stated, all reactions were performed in an oven-dried flask under argon or nitrogen and stirred with a Teflon-coated stir bar. Diethyl ether (Et_2O), Tetrahydrofuran (THF) and dichloromethane (DCM) were passed through alumina using the Sol-Tek ST-002 solvent purification system. Toluene, xylenes, and triethylamine were distilled from calcium hydride. Acetonitrile, 1,2-dichloroethane, and 1,4-dioxane were used as purchased from Sigma Aldrich in a Sure/Seal™ bottle. Chloroform was washed with deionized water (2 ×), dried with anhydrous calcium chloride, filtered, distilled from phosphorous pentoxide, and stored in the dark. Ethyl acetate was washed with 5% sodium carbonate solution, brine, dried with potassium carbonate, filtered, distilled from calcium hydride, and stored over 4 Å molecular sieves. Trifluorotoluene was distilled from phosphorous pentoxide and stored over 4 Å molecular sieves. Trifluoroethanol was reacted with sodium bicarbonate(s), filtered, distilled, and stored over 4 Å molecular sieves in the dark. Chlorobenzene was distilled from phosphorous pentoxide via vacuum distillation (95 mmHg, 101℃) and stored in the dark. Ethanol, dimethoxyethane, and dimethyl carbonate were allowed to stand over 4Å molecular sieves overnight and degassed by argon bubbling. 10% Carbon monoxide in argon (9.97% of CO in the balance gas argon as certified by Matheson) was purchased from Matheson. Glass or plastic syringes with stainless steel needles were used to transfer air-sensitive reagents and intermediates. Flash column chromatography was performed using silica gel (P60, 60 Å mean pore size, 40-63 μm). Analytical thin layer chromatography (TLC) was performed using glass plates coated with 0.25 mm silica gel and F254 as fluorescent indicator. Thin layer chromatography plates were visualized under shortwave ultraviolet light (254 nm). Solvents were removed using rotary evaporators attached to water aspirators. Proton (^{1}H), carbon (^{13}C) and phosphorus (^{31}P) nuclear magnetic resonance spectra were collected with Bruker Advance 300, 400 or 500 MHz spectrometers. All the spectra are reported in parts per million on the δ scale and referenced to deuterated chloroform (^{1}H: 7.26 ppm, ^{13}C: 77.16 ppm). Chemical shifts are reported in ppm and coupling patterns are reported as follows: s = singlet, d = doublet, t= triplet, q = quartet, p = pentet, m= multiplet, br = broad. Coupling constants (*J*) are reported in Hz. All NMR experiments were performed at room temperature. ESI mass spectrometry was performed on a Waters Micromass GCT high resolution mass spectrometer, while ES mass spectrometry was performed on a Waters Q-TOF Ultima API, Micromass UK Limited high resolution mass spectrometer. IR spectra were collected using a Perkin Elmer Spectrum Two FT-IR Spectrometer. HPLCs were performed using Shimadzu Nexera Series-Modular HPLC system with a SPD-M40 photodiode array detector and a Daicel Chiralpak AD-3, Chiralpak IB N-3 or Chiralpak IH-3 column with an injection volume of 1.0 μL and a flow rate of 1.0 mL/min or 0.8 mL/min. Optical rotations were measured at 589 nm (sodium D line) using a JASCO P-2000 Digital Polarimeter.

Synthesis of 1,6-enyne precursors

Syntheses of 1,6-enynes with NTs tether

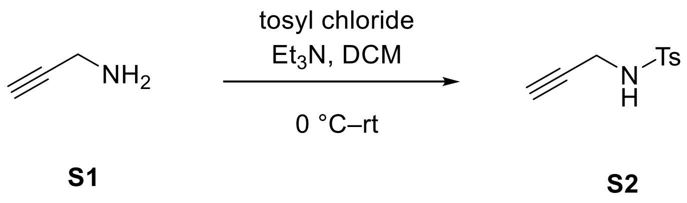

General Procedure A: Tosylation of Primary Amine

4-methyl-*N*-(prop-2-yn-1-yl) benzenesulfonamide (S2).

The synthesis of 4-methyl-*N*-(prop-2-yn-1-yl) benzenesulfonamide was performed using a modified literature procedure.[1] A two-necked, 100-mL, round-bottomed flask equipped with a magnetic stir bar, rubber septum, and nitrogen inlet adaptor was charged with propargyl amine **S1** (1.34 mL, 21.0 mmol, 1.05 equiv), triethylamine (7 mL, 50 mmol, 2.5 equiv) and dichloromethane (50 mL). The flask was placed in an ice/water bath, *p*-toluenesulfonyl chloride (3.8 g, 20 mmol, 1.0 equiv) was added portion wise over 10 min via a solid addition funnel. The reaction mixture was slowly warmed to rt and maintained overnight. The reaction mixture was poured into a separatory funnel and washed with saturated NH_4Cl solution (100 mL) and brine (20 mL). The organic layer was dried over magnesium sulfate and concentrated under rotary evaporation. The crude residue was purified by silica gel flash column chromatography (25% ethyl acetate in hexane) to yield the title compound as a white powder (3.2 g, 15.3 mmol, 77%). Spectral data matched that previously reported for compound **S2.**[2]

^{1}H NMR (400 MHz, $CDCl_3$)

δ 7.77 (d, J = 8.2 Hz, 2 H), 7.31 (d, J = 8.2 Hz, 2 H), 4.56 (br, s, 1 H), 3.83 (dd, J = 6.1, 2.5 Hz, 2 H), 2.44 (s, 3 H), 2.11 (t, J = 2.5 Hz, 1 H)

TLC

R_f = 0.31 (20% ethyl acetate/Hexanes) [silica gel, UV]

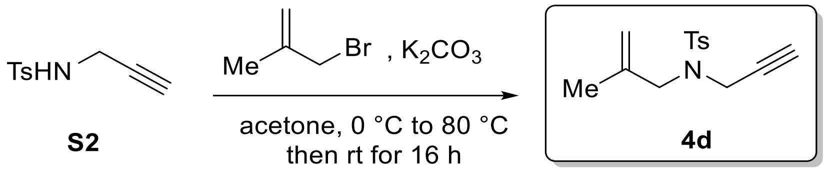

4-methyl-*N*-(2-methylallyl)-*N*-(prop-2-ynyl)benzenesulfonamide (4d).

The synthesis of 4-methyl-*N*-(2-methylallyl)-*N*-(prop-2-ynyl) benzenesulfonamide was performed using a modified procedure.[3] A single-necked, 250-mL, round-bottomed flask equipped with a magnetic stir bar was charged with 4-methyl-*N*-(prop-2-yn-1-yl) benzenesulfonamide **S2** (3.7 g, 17.7 mmol, 1.0 equiv). The flask was sealed with a rubber septum, evacuated, and refilled with nitrogen (3×) using a needle connected to a Schlenk manifold. Acetone (106 mL) was added to the flask via a syringe. The flask was placed in an ice/water bath. Potassium carbonate (3.99 g, 28.9 mmol, 1.6 equiv) was added in one portion to the flask by removal of the rubber septum. After addition, the neck was quickly replaced with a condenser under a flow of nitrogen. 3-Bromo-2-methylpropene (2.9 mL, 28.3 mmol, 1.6 equiv) was added to the reaction flask dropwise over 10 min by a syringe. The mixture was lowered into an oil bath and heated at reflux (oil bath, 80 °C) for 5 h. The flask was removed from the oil bath and maintained at rt for 16 h. The solvent was removed by rotatory evaporation. The resulting solid was dissolved in ethyl acetate (100 mL) and transferred to a separatory funnel, followed by the addition of water (30 mL). The aqueous layer was separated and extracted with ethyl acetate (3×40 mL). The combined organic layers were washed with brine (30 mL) and dried over magnesium sulfate. The organic layers were concentrated using rotatory evaporation and the crude residue was purified by flash column chromatography on silica gel (15% ethyl acetate/hexanes) to afford the title compound as a white solid (4.68 g, 17.68 mmol, 100%). Spectral data matched that previously reported for compound **4d**.[4]

^{1}H NMR (400 MHz, $CDCl_3$ with 0.03% v/v TMS)

δ 7.74 (d, *J* = 8.2 Hz, 2 H), 7.29 (d, *J* = 8.2 Hz, 2 H), 4.97 (br s, 2 H), 4.05 (d, *J* = 2.4 Hz, 2 H), 3.73 (s, 2 H), 2.43 (s, 3 H), 1.96 (t, *J* = 2.4 Hz, 1 H), 1.76 (s, 3 H)

^{13}C NMR (100.6 MHz, $CDCl_3$ with 0.03% v/v TMS)

δ 143.6, 139.3, 136.2, 129.6 (2C), 128.0 (2C), 115.7, 76.5, 73.8, 52.5, 35.6, 21.7, 19.8

TLC

R_f = 0.42 (15% ethyl acetate/ hexanes) [silica gel, UV, *p*-anisaldehyde]

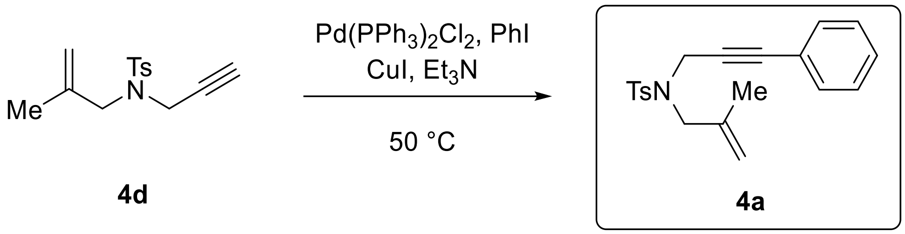

General Procedure B (Sonogashira Coupling)

(4-methyl-*N*-(2-methylallyl)-*N*-(prop-2-ynyl)benzenesulfonamide (4a).

The synthesis of 4-methyl-*N*-(2-methylallyl)-*N*-(prop-2-ynyl) benzenesulfonamide was performed in a manner analogous to that previously reported.[3] A 100-mL, single-necked round-bottomed flask equipped with a magnetic stir bar was charged with $Pd(PPh_3)_2Cl_2$ (84.2 mg, 0.12 mmol, 0.02 equiv), CuI (22.9 mg, 0.12 mmol, 0.02 equiv), and 4-methyl-*N*-(2-methylallyl)-*N*-(prop-2-ynyl) benzenesulfonamide **4d** (1.5 g, 5.69 mmol, 1.00 equiv). The flask was equipped with a reflux condenser and evacuated, refilled with nitrogen (3×) using a needle connected to a Schlenk manifold. To this reflux apparatus, freshly distilled triethylamine (52 mL) was added followed by the addition of iodobenzene (0.7 mL, 6.3 mmol, 1.1 equiv) via a syringe. The flask was placed in an oil bath (50 °C) and the resulting mixture was maintained for 16 h. Methyl tert-butyl ether (28 mL) was added to the reaction mixture and filtered through celite, the residual solvent was removed by rotary evaporation and the crude residue was purified by silica gel flash column chromatography (5% ethyl acetate/hexanes) to afford the title compound (1.8 g, 5.30 mmol, 93%) as a white solid. Spectral data matched that previously reported for compound **4a.**[3]

^{1}H NMR (400 MHz, $CDCl_3$)

δ 7.78 (d, J = 8.2 Hz, 2 H), 7.29-7.20 (m, 5 H), 7.03–7.01 (m, 2 H), 5.00 (br s, 2 H), 4.25 (s, 2 H), 3.80 (s, 2 H), 2.33 (s, 3 H), 1.81 (s, 3 H)

^{13}C NMR (100.6 MHz, $CDCl_3$)

δ 143.6, 139.5, 136.2, 131.6 (2C), 129.6 (2C), 128.5, 128.2 (2C), 128.0 (2C), 122.4, 115.7, 85.8, 81.8, 52.9, 36.5, 21.5, 19.9

TLC

R_f = 0.3 (5% ethyl acetate/hexanes) [silica gel, UV]

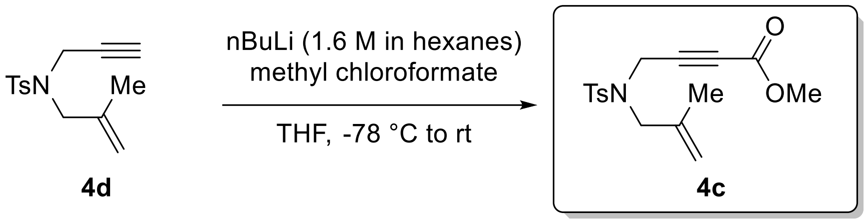

methyl 4-((4-methyl-*N*-(2-methylallyl)phenyl)sulfonamido)but-2-ynoate (4c)

A 50-mL round bottomed flask equipped with a magnetic stir bar was charged with 4-methyl-*N*-(2-methylallyl)-*N*-(prop-2-ynyl) benzenesulfonamide **4d** (550 mg, 2.09 mmol, 1.0 equiv). The flask was sealed with a rubber septum, evacuated, and refilled with nitrogen (3×) using a needle connected to a Schlenk manifold. THF (10 mL) was added to the flask and the flask was cooled to -78 ℃ in a dry ice-acetone bath. nBuLi solution in hexanes (2.0 mL, 3.14 mmol, 1.5 equiv) was added dropwise via a glass syringe and the resulting yellow solution was maintained at -78 ℃ for 1 h. Methyl chloroformate (1.0 mL, 12.54 mmol, 6.0 equiv) was added dropwise to the yellow solution at -78 ℃, the resulting orange solution was maintained at -78 ℃ for 1 h before letting it gradually warm up to rt for overnight. A saturated ammonium chloride solution was added dropwise until bubbling ceased. The mixture was transferred to a separatory funnel, and the aqueous layer was separated and extracted with diethyl ether (3×20 mL). The combined organic layers were washed with brine and dried over magnesium sulfate. The residual solvent was removed by rotary evaporation and the crude residue was purified by silica gel flash column chromatography (25% ethyl acetate/hexanes) to afford the title compound (285 mg, 0.89 mmol, 43%) as a white solid. Spectral data matched that previously reported for compound **4c.**[5]

^{1}H NMR (400 MHz, $CDCl_3$)

δ 7.73 (d, *J* = 8.2 Hz, 2 H), 7.31 (d, *J* = 8.2 Hz, 2 H), 4.99 (s, 1 H), 4.96 (s, 1H), 4.15 (s, 2 H), 3.71 (s, 2 H), 3.69 (s, 3 H), 2.42 (s, 3 H), 1.76 (s, 3 H)

small impurities at 4.33, 4.09, 3.97–3.85, 3.54, 3.50, 3.46

^{13}C NMR (100.6 MHz, $CDCl_3$)

δ 153.1, 144.1, 138.9, 135.5, 129.8 (2C), 127.8 (2C), 116.3, 80.6, 77.4, 53.2, 52.8, 35.6, 21.7, 19.8

TLC

R_f = 0.25 (25% ethyl acetate/hexanes) [silica gel, UV]

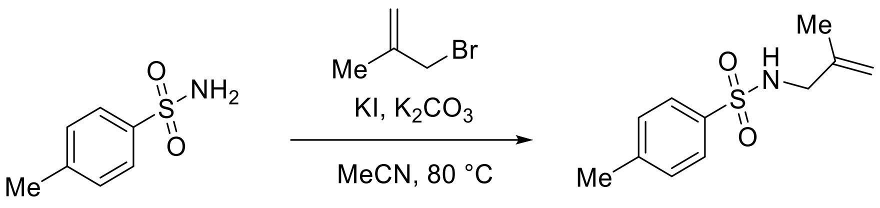

4-methyl-*N*-(2-methylallyl)benzenefulfonamide (S4)**.**

The synthesis of 4-methyl-*N*-(2-methylallyl)benzenefulfonamide was performed in a manner analogous to that previously reported.[6] A 50 mL single-necked round bottomed flask equipped with a magnetic stir bar was charged with *p*-toluenesulfonamide **S3** (1.78 g, 10.38 mmol, 2.0 equiv), potassium iodide (86.2 mg, 0.52 mmol, 0.1 equiv) and potassium carbonate (1.43g, 10.38 mmol, 2.0 equiv). The flask was equipped with a reflux condenser and the entire reflux apparatus was evacuated, refilled with nitrogen (3×) using a needle connected to a Schlenk manifold. Acetonitrile (16 mL) was added followed by the addition of 3-bromo-2-methylpropene (0.52 mL, 5.19 mmol, 1.0 equiv) dropwise over 2 min. The resulting mixture was heated at 80 °C (oil bath) under argon for 20 h. The reaction mixture was concentrated using rotary evaporation. Water (5 mL) was added to the residue followed by ethyl acetate (20 mL), and the mixture was transferred to a separatory funnel. The aqueous layer was separated and extracted with ethyl acetate (3×20 mL). The combined organic layers were washed brine (5 mL) and dried over magnesium sulfate. The combined organic layers were concentrated using rotary evaporation and the crude residue was purified by flash column chromatography on silica gel (8%–20% ethyl acetate/hexanes) to afford the title compound as sticky yellow oil (910 mg, 4.04 mmol, 78%). Spectral data matched that previously reported for compound **S4**.[7]

^{1}H NMR (400 MHz, $CDCl_3$)

δ 7.75 (d, J = 8.1 Hz, 2 H), 7.31 (d, J = 8.1 Hz, 2 H), 4.85 (s, 1 H), 4.83 (s, 1 H), 4.42 (br s, 1 H), 3.49 (d, J = 6.4 Hz, 2 H), 2.43 (s, 3 H), 1.68 (s, 3 H)

^{13}C NMR (100.6 MHz, $CDCl_3$)

δ 143.6, 140.7, 137.1, 129.9 (2C), 127.3 (2C), 112.9, 49.2, 21.7, 20.3

TLC

R_f = 0.35 (20% ethyl acetate/hexanes) [silica gel, UV]

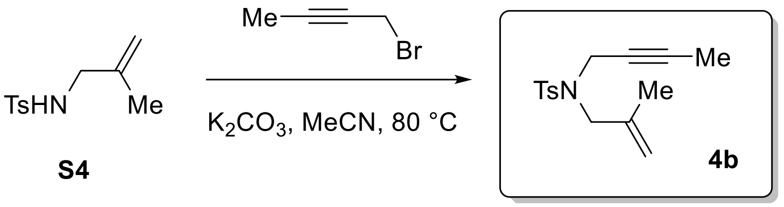

***N*-(but-2-ynyl)-4-methyl-*N*-(2-methylallyl)benzenesulfonamide (4b).**

The synthesis of *N*-(but-2-ynyl)-4-methyl-*N*-(2-methylallyl)benzenesulfonamide was performed in a manner analogous to that previously reported.[6] A 50-mL round-bottomed flask equipped with a magnetic stir bar was charged with potassium carbonate (1.2 g, 8.08 mmol, 2.0 equiv). The flask was equipped with a reflux condenser and the apparatus evacuated/refilled with argon (×3) via a needle connected to a Schlenk manifold. *N*-2-methylallyl-*p*-toluenesulfonamide **S4** (910 mg, 4.04 mmol, 1.0 equiv) was dissolved in acetonitrile (10 mL) in a separate 25-mL round-bottomed flask. This solution was added dropwise to the reaction flask followed by the addition of a rinse of the 25-mL flask using acetonitrile (5 mL). 1-Bromo-2-butyne (0.46 mL, 5.25 mmol, 1.3 equiv) was added dropwise to the rection flask. The reaction was heated at 80 ℃ and maintained under an argon atmosphere for 14 h. The volatiles were removed using rotary evaporation and the residue was dissolved in ethyl acetate (30 mL), followed by the addition of water (20 mL). The mixture was transferred to a separatory funnel and the aqueous layer was separated and extracted with ethyl acetate (3×20 mL). The combined organic layers were washed with brine (10 mL) and dried over magnesium sulfate. The organic layers were concentrated using rotary evaporation and the crude residue was purified by flash column chromatography on silica gel (6% ethyl acetate/hexanes) to afford the title compound as white solid (1.08 g, 3.89 mmol, 96 %). Spectral data matched that previously reported for compound **4b**.[3]

^{1}H NMR (400 MHz, $CDCl_3$)

δ 7.74 (d, *J* = 7.9 Hz, 2 H), 7.29 (d, *J* = 7.9 Hz, 2 H), 4.95 (s, 2 H), 3.97 (s, 2 H), 3.70 (s, 2 H), 2.42 (s, 3 H). 1.76 (s, 3 H), 1.50 (s, 3 H)

^{13}C NMR (100.6 MHz, $CDCl_3$)

δ 143.3, 139.7, 136.4, 129.3 (2C), 128.1 (2C), 115.3, 81.6, 71.7, 52.6, 36.2, 21.6, 19.9, 3.3

TLC

R_f = 0.2 (6% ethyl acetate/hexanes) [silica gel, UV]

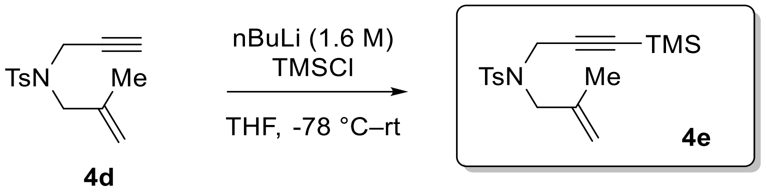

4-methyl-*N*-(2-methylallyl)-*N*-[3-(trimethylsilyl)prop-2-ynyl]benzenesulfonamide (**4e**)

A 15-mL round-bottomed flask equipped with a magnetic stir bar was charged with 4-methyl-*N*-(2-methylallyl)-*N*-(prop-2-ynyl) benzenesulfonamide **4d** (600 mg, 2.28 mmol, 1.0 equiv). The flask was sealed with a rubber septum, evacuated, and refilled with nitrogen (3×) using a needle connected to a Schlenk manifold. THF (5 mL) was added to the reaction flask and the flask was cooled to -78 ℃ in an acetone-dry ice bath. nBuLi (1.6 M in hexanes, 1.85 mL, 2.92 mmol, 1.3 equiv) was slowly added to the solution through a syringe. After stirring for 30 min, TMSCl (0.59 mL, 4.67 mmol, 2.1 equiv) was added dropwise. The resulting orange solution was maintained at -78 ℃ for 1 h. A saturated solution of ammonium chloride (5 mL) was added, and the mixture was transferred to a separatory funnel. The aqueous layer was separated and extracted with ethyl acetate (3×15mL). The combined organic layers were washed with water, brine and dried over magnesium sulfate. The organic layers were concentrated using rotary evaporation and the crude residue was purified by flash column chromatography on silica gel (6% ethyl acetate/hexanes) to afford the title compound as light-yellow solid (450 mg, 1.34 mmol, 59 %). Spectral data matched that previously reported for compound **4e**. [8]

^{1}H NMR ($CDCl_3$, 400 MHz)

δ 7.74 (d, *J* = 8.2 Hz, 2 H), 7.29 (d, *J* = 8.2 Hz, 2 H), 4.98 (s, 1 H), 4.96 (s, 1 H), 4.05 (s, 2 H), 3.73 (s, 2 H), 2.42 (s, 3 H), 1.78 (s, 3 H), -0.03 (s, 9 H)

^{13}C NMR (100.6 MHz, $CDCl_3$)

δ 143.4, 139.3, 136.3, 129.6 (2C), 128.0 (2C), 115.7, 97.9, 91.1, 52.5, 36.6, 21.7, 19.9, -0.28

HRMS

HRMS-ESI (m/z): $[M + H]^+$ calcd for $C_{17}H_{26}NO_2SSi$, 336.1448; found: 336.1446

TLC

R_f = 0.29 (10% ethyl acetate/hexanes) [silica gel, UV]

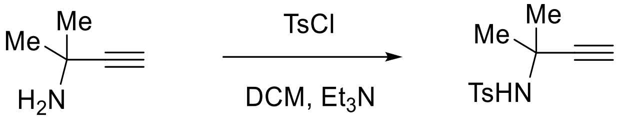

S5 **S6**

4-methyl-*N*-(2-methylbut-3-yn-2-yl)benzenesulfonamide (**S6**)

Follows General Procedure A: A 100 mL round-bottomed flask equipped with a magnetic stir bar was charged with 1,1-dimethylpropargyl **S5** (1.0 mL, 9.6 mmol, 1.0 equiv) and triethylamine (1.5 mL, 10.6 mmol, 1.1 equiv) in DCM (3 mL). The flask was cooled to 0 ℃. A solution of *p*-toluenesulfonyl chloride (2.0 g, 10.6 mmol, 1.1 equiv) in DCM (20 mL) was added dropwise to the reaction flask. The mixture was allowed to warm to r.t and maintained overnight. A saturated solution of ammonium chloride (10 mL) was added, and the mixture was transferred to a separatory funnel. The aqueous layer was separated and extracted with ethyl acetate (25 mL×3). The combined organic layers were washed with brine and dried over magnesium sulfate. The organic layers were concentrated using rotatory evaporation and the crude residue was purified by flash column chromatography on silica gel (20% ethyl acetate/hexanes) to afford the title compound as an off-white solid (1.9 g, 7.95 mmol, 83%). Spectral data matched that previously reported for compound **S6**.[9]

^{1}H NMR (400 MHz, $CDCl_3$)

δ 7.79 (d, *J* = 8.2 Hz, 2 H), 7.28 (d, *J* = 8.2 Hz, 2 H), 4.67 (br s, 1 H), 2.42 (s, 3 H), 2.10 (s, 1 H), 1.55 (s, 6 H)

TLC

R_f = 0.18 (20% ethyl acetate/Hexanes) [silica gel, UV]

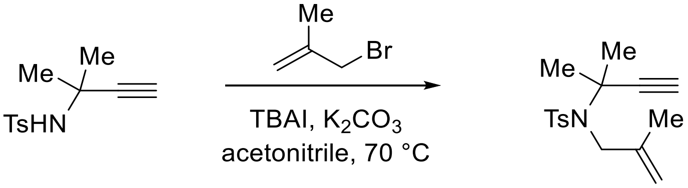

S6 **S7**

4-methyl-*N*-(2-methylallyl)-*N*-(2-methylbut-3-yn-2-yl)benzenesulfonamide (**S7**)

A 50 mL round-bottomed flask equipped with a magnetic bar was charged with 4-methyl-*N*-(2-methylbut-3-yn-2-yl)benzenesulfonamide **S6** (500 mg, 2.1 mmol, 1.0 equiv), potassium carbonate (581 mg, 4.2 mmol,

2.0 equiv) and tetra-*n*-butylammonium iodide (78 mg, 0.2 mmol, 0.1 equiv). The flask was equipped with a reflex condenser, evacuated, and refilled with nitrogen (3×) using a needle connected to a Schlenk manifold, after which acetonitrile (11 mL) was added to the flask, followed by dropwise addition of 3-bromo-2-methylpropene (0.23 mL, 2.3 mmol, 1.1 equiv). The flask was placed in pre-heated oil bath at 70 °C and maintained for 20 h. The reaction mixture was poured into a separatory funnel that contained water (5 mL), and ethyl acetate (12 mL). The aqueous layer was separated and extracted with ethyl acetate (3× 12 mL). The combined organic layers were washed with brine and dried over magnesium sulfate. The organic layers were concentrated using rotatory evaporation and the crude residue was purified by flash column chromatography on silica gel (10% ethyl acetate/hexanes) to afford the title compound as a white solid (576 mg, 1.98 mmol, 94%). Spectral data matched that previously reported for compound **S7**.[10]

<u>^{1}H NMR</u> (400 MHz, $CDCl_3$)

δ 7.73 (d, *J* = 8.3 Hz, 2 H), 7.26 (d, *J* = 8.3 Hz, 2 H), 5.10–5.09 (m, 1 H), 4.94–4.94 (m, 1 H), 4.11 (s, 2 H), 2.41 (s, 3 H), 2.19 (s, 1 H), 1.79 (s, 3 H), 1.65 (s, 6 H)

<u>TLC</u>

R_f = 0.29 (15% ethyl acetate/ hexanes) [silica gel, UV]

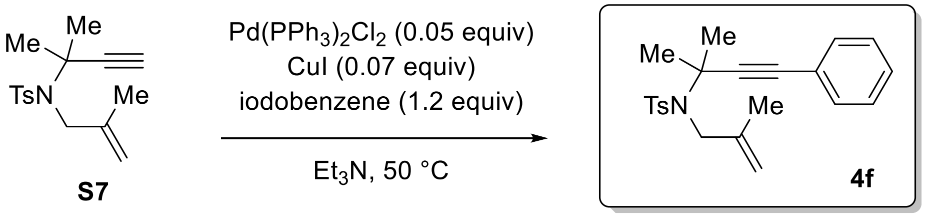

4-methyl-*N*-(2-methyl-4-phenylbut-3-yn-2-yl)-*N*-(2-methylallyl)benzenesulfonamide (4f)

<u>Follows General Procedure B</u>: $Pd(PPh_3)_2Cl_2$ (61.4 mg, 0.088 mmol, 0.05 equiv), CuI (23.3 mg, 0.123 mmol, 0.07 equiv), and 4-methyl-*N*-(2-methylallyl)-*N*-(2-methylbut-3-yn-2-yl)benzenesulfonamide **S7** (510 mg, 1.75 mmol, 1.0 equiv) were added to a 25 mL round-bottomed flask, the flask was equipped with a reflux condenser. The reflux apparatus was evacuated and refilled with argon (3×) through a needle connected to a Schenk manifold. Triethylamine (14 mL) was added dropwise to the flask via a syringe. Iodobenzene (0.24 mL, 2.1 mmol, 1.2 equiv) was added all at once via a syringe. The resulting mixture was maintained at 50 °C for 20 h. The mixture was poured into a separatory funnel that contained saturated sodium bicarbonate solution (10 mL). The aqueous layer was separated and extracted with ethyl acetate (3×20 mL).

The combined organic layers were washed with brine and dried over magnesium sulfate. The organic layers were concentrated using rotatory evaporation and the crude residue was purified by flash column chromatography on silica gel (10% ethyl acetate/hexanes) to afford the title compound as a sticky light-yellow oil (640 mg, 1.75 mmol, 100%). Spectral data matched that previously reported for compound **4f**.[10]

^{1}H NMR (400 MHz, $CDCl_3$)

δ 7.75 (d, *J* = 8.3 Hz, 2 H), 7.28–7.12 (m, 7 H), 5.15–5.14 (m, 1 H), 4.97–4.96 (m, 1 H), 4.14 (s, 2 H), 2.33 (s, 3 H), 1.82 (s, 3 H), 1.74 (s, 6 H)

TLC

R_f = 0.24 (10% ethyl acetate/hexanes) [silica gel, UV]

Syntheses of 1,6-enynes with ether tether

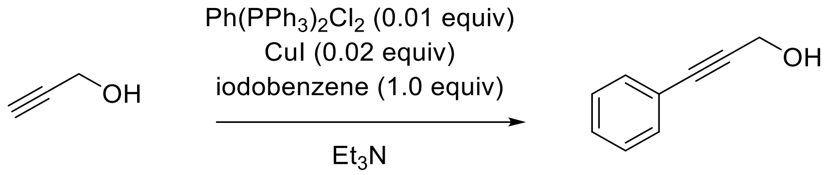

3-phenyl-2-propyn-1-ol (**S9**)

Follows General Procedure B: A 500-mL round-bottomed flask was charged with $Pd(PPh_3)_2Cl_2$ (225 mg, 0.32 mmol, 0.01 equiv) and CuI (122 mg, 0.64 mmol, 0.02 equiv). Triethylamine (128 mL) was added followed by dropwise addition of iodobenzene (3.56 mL, 32 mmol, 1.0 equiv) and propargyl alcohol **S8** (2.06 mL, 35.2 mmol, 1.1 equiv), and the reaction was maintained at rt for 4 h. The reaction mixture was then poured into a separatory funnel, which contained a saturated solution of ammonium chloride (40 mL). The aqueous layer was separated and extracted with diethyl ether (3×60 mL). The combined organic layers were washed with brine, dried over sodium sulfate, and then filtered. The organic layers were concentrated using rotary evaporation and the crude residue was purified via flash column chromatography on silica gel (25% ethyl acetate in hexanes) to afford the title compound as red oil (4.27 g, 32 mmol, 100%). The product **S9** was previously characterized and all spectra information match those reported.[11,12]

^{1}H NMR ($CDCl_3$, 400 MHz)

δ 7.45–7.43 (m, 2 H), 7.33–7.31 (m, 3 H), 4.50 (s, 2 H), 1.67 (s, 1 H)

TLC

R_f = 0.2 (25% ethyl acetate in hexanes); silica gel, UV

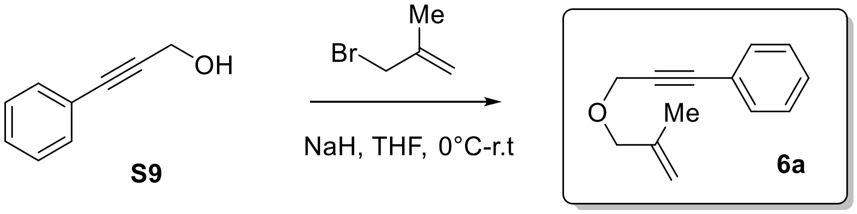

General Procedure C (Williamson Ether Synthesis)

3-[(2-methyl-2-propenyl)oxy]-1-phenyl-1-propyne (6a).

A 50-mL round-bottomed flask was charged with sodium hydride (60% dispersion in mineral oil, 0.48 g, 12 mmol, 1.5 equiv), the flask was then evacuated/refilled with argon (3×) through a needle connected to a Schlenk Manifold. Freshly distilled hexanes (5 mL) were added, the suspension was stirred for 5 min and allowed to settle for 2 min. The liquid layer was carefully removed using a syringe equipped with a 6-inch steel needle. The remaining sodium hydride was placed under high vacuum to remove the residual hexanes. THF (17 mL) was added to the flask and the flask was placed in an ice-water bath. A solution of 3-phenyl-2-propyn-1-ol **S9** (1.06 g, 8.02 mmol, 1.0 equiv) dissolved in THF (2 mL) was added over 5 min via syringe. The resulting brownish mixture was allowed to warm slowly to rt and maintained for 2 h. The flask was placed in an ice-water bath and 3-bromo-2-methylpropene (1.6 mL, 16 mmol, 2.0 equiv) was added dropwise over 6 min via syringe. The mixture was allowed to slowly warm to rt and maintained overnight. Water (6 mL) was added slowly to the brownish mixture until bubbling ceased. The mixture was poured into a separatory funnel and the layers separated. The aqueous layer was extracted with diethyl ether (3×20 mL). The combined organic layers were washed with water (10 mL) and brine (10 mL) and dried over sodium sulfate. The organic layers were concentrated by rotary evaporation and the crude residue was purified via flash column chromatography on silica gel (10% ethyl acetate in hexanes) to afford the title compound as light-yellow oil (1.14 g, 6.1 mmol, 76%). Spectral data matched that previously reported for compound **6a.**[13]

^{1}H NMR ($CDCl_3$, 400 MHz)

δ 7.47–7.44 (m, 2 H), 7.32–7.29 (m, 3 H), 5.04–5.03 (m, 1 H), 4.95 (s, 1 H), 4.36 (s, 2 H), 4.05 (s, 2 H), 1.79 (s, 3 H)

^{13}C NMR ($CDCl_3$, 100.6 MHz)

δ 141.6, 131.8 (2C), 128.4, 128.3 (2C), 122.7, 113.0, 86.2, 85.2, 73.7, 57.8, 19.6

TLC

R_f = 0.38 (10% ethyl acetate in hexanes); silica gel, UV

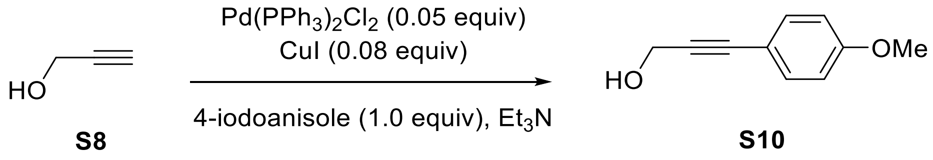

4-(4-methoxyphenyl)prop-2-yn-1-ol (**S10**)

Follows General Procedure B: A 50-mL round-bottomed flask was charged with $Pd(PPh_3)_2Cl_2$ (175 mg, 0.25 mmol, 0.05 equiv), CuI (76 mg, 0.4 mmol, 0.08 equiv) and 4-iodoanisole (1.17 g, 5 mmol, 1.0 equiv). Triethylamine (12 mL) was added followed by dropwise addition of propargyl alcohol **S8** (0.35 mL, 6 mmol, 1.2 equiv). The mixture was maintained at rt for 4 h. A saturated solution of ammonium chloride (3 mL) and diethyl ether (10 mL) was added in this sequence and the mixture was transferred to a separatory funnel. The aqueous layer was separated and extracted with diethyl ether (3×10 mL). The combined organic layers were washed with brine (4 mL) and dried over magnesium sulfate. The organic layers were concentrated using rotary evaporation and the crude residue was purified by flash column chromatography on silica gel (25%–30% acetate/hexanes) to afford the title compound as yellow solid (802 mg, 4.95 mmol, 99%). Spectral data matched that previously reported for compound **S10**.[14]

^{1}H NMR ($CDCl_3$, 500 MHz)

δ 7.39–7.37 (m, 2 H), 6.85–6.83 (m, 2 H), 4.48 (d, J = 6.2 Hz, 1 H), 3.81 (s, 3 H), 1.61 (t, J = 6.2 Hz, 1H)

TLC

R_f = 0.25 (40% ethyl acetate/hexanes) [silica gel, UV]

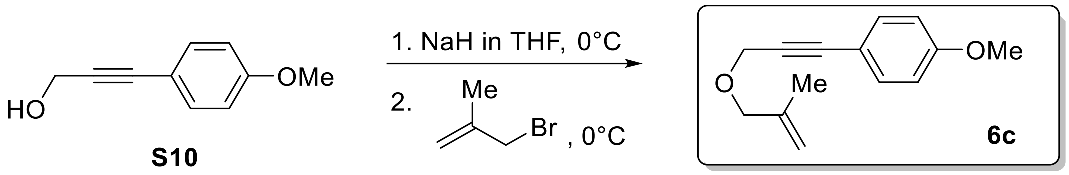

1-methoxy-4-(3-((2-methylallyl)oxy)prop-1-yn-1-yl)benzene (**6c**)

Follows General Procedure C: To a suspension of NaH (60% dispersion in mineral oil, 300 mg, 7.43 mmol, 1.5 equiv) in THF (11 mL) was added dropwise a solution of 3-(4-methoxyphenyl)prop-2-yn-1-ol **S10** (0.8 g, 4.95 mmol, 1.0 equiv) in THF (2 mL) at 0 °C over a period of 4.5 min under argon. The vial containing 3-(4-methoxyphenyl)prop-2-yn-1-ol was washed with an additional 1 mL of THF and added to the reaction mixture. The suspension was warmed to rt and maintained for an additional 2 h. The reaction mixture was cooled in an ice-water bath and 3-bromo-2-methylpropene (0.95 mL, 9.9 mmol, 2 equiv) was added dropwise. The mixture was allowed to slowly warm to rt and maintained overnight. The subsequent workup and purification by flash column chromatography on silica gel (2%–5% acetate/hexanes) afforded the title compound **6c** as light-yellow oil (1.02 g, 4.72 mmol, 95%).

^{1}H NMR ($CDCl_3$, 400 MHz)

δ 7.39 (d, *J* =8.8 Hz, 2 H), 6.83 (d, *J* = 8.8 Hz, 2 H), 5.03 (s, 1 H), 4.94 (s, 1 H), 4.34 (s, 2 H), 4.04 (s, 2 H), 3.80 (s, 3 H), 1.78 (s, 3 H)

^{13}C NMR ($CDCl_3$, 100.6 MHz)

δ 159.8, 141.8, 133.3 (2C), 114.9, 114.0 (2C), 113.0, 86.2, 83.9, 73.8, 58.0, 55.4, 19.7

HRMS

HRMS-ESI (m/z): $[M + H]^+$ calcd for $C_{14}H_{17}O_2$, 217.1223; found: 217.1218

IR

1605, 1245, 1031 cm^{-1}

TLC

R_f = 0.28 (5% ethyl acetate/hexanes) [silica gel, UV]

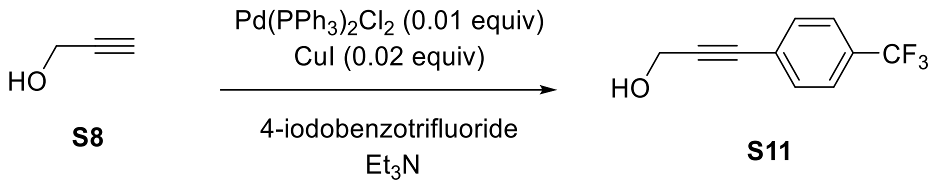

3-(4-trifluoromethylphenyl)prop-2-yn-1-ol (**S11**)

Follows General Procedure B: A 50-mL round-bottomed flask was charged with $Pd(PPh_3)_2Cl_2$ (42.1 mg, 0.06 mmol, 0.01 equiv) and CuI (22.8 mg, 0.12 mmol, 0.02 equiv). Triethylamine (24 mL) was added followed by dropwise addition of 4-iodobenzotrifluoride (0.88 mL, 6.0 mmol, 1.0 equiv) and propargyl alcohol **S8** (0.38 mL, 6.6 mmol, 1.1 equiv). The mixture was maintained at rt for 3 h. A saturated solution of sodium bicarbonate (4.5 mL) was added, and the mixture was transferred to a separatory funnel. The aqueous layer was separated and extracted with ethyl acetate (3×13 mL). The combined organic layers were washed with brine (3 mL) and dried over magnesium sulfate. The organic layers were concentrated using rotary evaporation and the crude residue was purified by flash column chromatography on silica gel (30% acetate/hexanes) to afford the title compound as reddish-brown solid (910 mg, 4.55 mmol, 76%). Spectral data matched that previously reported for compound **S11**.[14]

^{1}H NMR ($CDCl_3$, 400 MHz)

δ 7.58 (d, *J* = 8.5 Hz, 2 H), 7.54 (d, *J* = 8.5 Hz, 2 H), 4.52 (s, 2 H), 1.66 (br s, 1 H)

TLC

R_f = 0.25 (30% ethyl acetate/hexanes) [silica gel, UV]

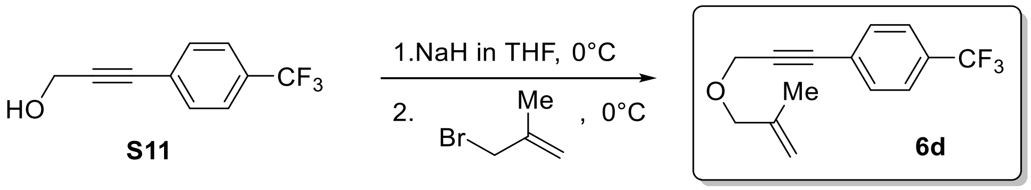

1-(3-((2-methylallyl)oxy)prop-1-yn-1-yl)-4-(trifluoromethyl)benzene (**6d**)

Follows General Procedure C: To a suspension of NaH (60% dispersion in mineral oil, 0.13 g, 5.3 mmol, 1.2 equiv, pre-washed with hexanes) in THF (7.5 mL) was added dropwise a solution of 3-(4-trifluoromethylphenyl)prop-2-yn-1-ol **S11** (0.86 g, 4.3 mmol, 1 equiv) in THF (1 mL) at 0 °C under argon. The suspension was warmed to rt and maintained for an additional 2 h. The reaction mixture was cooled in an ice-water bath and 3-bromo-2-methylpropene (1.16 g, 8.6 mmol, 2 equiv) dissolved in THF (1 mL) was

added dropwise. The mixture was allowed to slowly warm to rt and maintained overnight. The subsequent workup and purification by flash column chromatography on silica gel (5% acetate/hexanes) afforded the title compound as yellow oil (0.86 g, 3.38 mmol, 79%). Spectral data matched that previously reported for compound **6d**.[15]

^{1}H NMR ($CDCl_3$, 400 MHz)

δ 7.58 (d, *J* = 8.7 Hz, 2 H), 7.54 (d, *J* = 8.7 Hz, 2 H), 5.03 (s, 1 H), 4.96 (s, 1 H), 4.37 (s, 2 H), 4.04 (s, 2 H), 1.79 (s, 3 H)

^{13}C NMR ($CDCl_3$, 100.6 MHz)

δ 141.5, 132.1 (2C), 130.3 (q, *J* = 32.7 Hz), 126.7, 125.3 (2C, q, *J* = 3.7 Hz), 124.0 (q, *J* = 272.4 Hz), 113.3, 88.0, 84.9, 74.1, 57.8, 19.7

TLC

R_f = 0.7 (25% ethyl acetate/hexanes) [silica gel, UV]

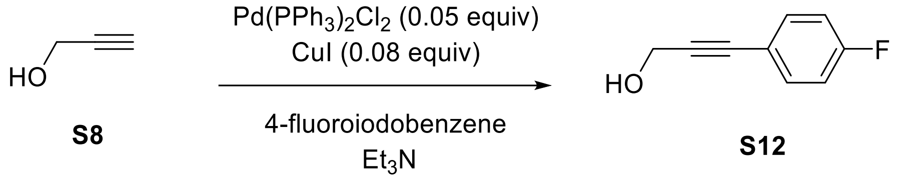

3-(4-fluoro-phenyl)-prop-2-yn-1-ol (**S12**)

Follows General Procedure B: A 50-mL round-bottomed flask was charged with $Pd(PPh_3)_2Cl_2$ (175 mg, 0.25 mmol, 0.05 equiv), CuI (76 mg, 0.4 mmol, 0.08 equiv) and 4-fluoroiodobenzene (0.58 mL, 5.0 mmol, 1.0 equiv). Triethylamine (12 mL) was added dropwise followed by dropwise addition propargyl alcohol **S8** (0.35 mL, 6.0 mmol, 1.2 equiv). The mixture was maintained at rt for 15 h. A saturated solution of ammonium chloride (3 mL) and diethyl ether (10 mL) were added in this sequence. The mixture was transferred to a separatory funnel, the aqueous layer was separated and extracted with diethyl ether (3×10 mL). The combined organic layers were washed with brine (4 mL) and dried over magnesium sulfate. The organic layers were concentrated using rotary evaporation and the crude residue was purified by flash column chromatography on silica gel (20%–30% acetate/hexanes) to afford the title compound as yellow oil (752 mg, 5.0 mmol, 100%). Spectral data matched that previously reported for compound **S12**.[16]

^{1}H NMR ($CDCl_3$, 400 MHz)

δ 7.44-7.40 (m, 2 H), 7.03–6.99 (m, 2 H), 4.48 (d, J = 6.1 Hz, 2 H), 1.66–1.63 (m, 1 H)

TLC

R_f = 0.3 (40% ethyl acetate/hexanes) [silica gel, UV]

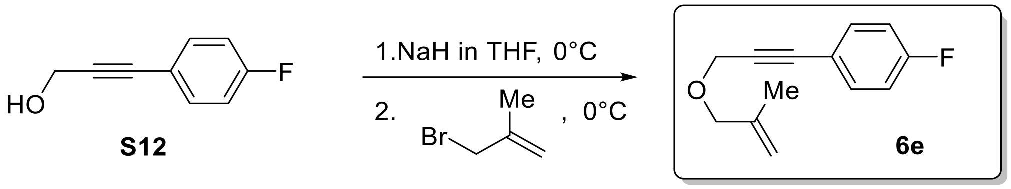

1-Fluoro-4-(3-((2-methylallyl)oxy)prop-1-yn-1-yl)benzene (**6e**)

Follows General Procedure C: To a suspension of NaH (60% dispersion in mineral oil, 0.3 g, 7.5 mmol, 1.5 equiv) in THF (11 mL) was added dropwise a solution of 3-(4-fluoro-phenyl)-prop-2-yn-1-ol **S12** (0.75 g, 5.0 mmol, 1 equiv) in THF (1 mL) at 0 °C under argon. The suspension was warmed to rt and maintained for an additional 2 h. The reaction mixture was cooled in an ice-water bath and 3-bromo-2-methylpropene (1.35 g, 10 mmol, 2 equiv) was added dropwise. The mixture was allowed to slowly warm to rt and maintained overnight. Subsequent workup and purification by flash column chromatography on silica gel (5% acetate/hexanes) afforded the title compound as yellow oil (918mg, 4.49 mmol, 90%). Spectral data matched that previously reported for compound **6e**.[15]

^{1}H NMR ($CDCl_3$, 400 MHz)

δ 7.44–7.41 (m, 2 H), 7.02–6.98 (m, 2 H), 5.03 (s, 1 H), 4.95 (s, 1 H), 4.33 (s, 2 H), 4.03 (s, 2 H), 1.78 (s, 3 H)

^{13}C NMR ($CDCl_3$, 100.6 MHz)

δ 162.7 (d, J =249.6 Hz), 141.6, 133.8 (2C, d, J = 8.8 Hz), 118.9 (d, J = 3.4 Hz), 115.7 (2C, d, J = 22.3 Hz), 113.2, 85.2, 85.0 (d, J = 1.5 Hz), 73.9, 57.8, 19.7

HRMS

HRMS-ESI (m/z): $[M + H]^+$ calcd for $C_{13}H_{14}OF$, 205.1023; found: 205.1018

TLC

R_f = 0.25 (3% ethyl acetate/hexanes) [silica gel, UV]

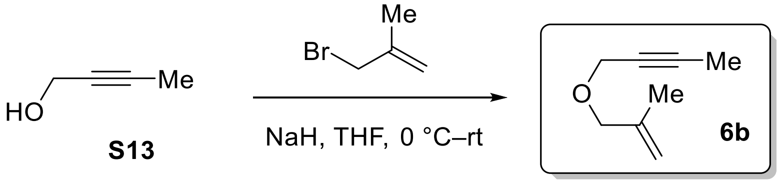

1-((2-methylallyl)oxy)but-2-yne (6b)

Follows General Procedure C: To suspension of NaH (60% dispersion in mineral oil, 0.43 g, 10.7 mmol, 1.5 equiv) in THF (15 mL) was added dropwise a solution of 2-butyn-1-ol **S13** (500 mg, 7.1 mmol, 1.0 equiv) in THF (1.7 mL) via syringe under argon at 0 ℃ over 4 min. The red suspension was slowly warmed to rt and maintained for 2 h. The brownish mixture was cooled in an ice-water bath and 3-bromo-2-methylpropene (1.42 mL, 14.26 mmol, 2.0 equiv) was added dropwise. The mixture was allowed to slowly warm up to rt and maintained overnight. Subsequent workup and purification by flash column chromatography on silica gel (2% diethyl ether in pentane) afforded the title compound **6b** as colorless oil (318 mg, 2.56 mmol, 36%).

^{1}H NMR ($CDCl_3$, 500 MHz)

δ 4.98–4.98 (m, 1 H), 4.91–4.91 (m, 1 H), 4.08 (q, *J* = 2.4 Hz, 2 H), 3.95 (s, 2 H), 1.86 (t, *J* = 2.4 Hz, 3 H), 1.75 (s, 3 H)

^{13}C NMR ($CDCl_3$, 125.8 MHz)

δ 114.7, 112.7, 82.3, 75.2, 73.5, 57.6, 19.5, 3.6

HRMS

HRMS-ESI (m/z): $[M + H]^+$ calcd for $C_8H_{13}O$, 125.0961; found: 125.1150

IR

2222, 1656, 1077 cm^{-1}

TLC

R_f = 0.25 (5% diethyl ether in pentane); silica gel, *p*-anisaldehyde stain

Synthesis of 1,6-enynes with malonate tether

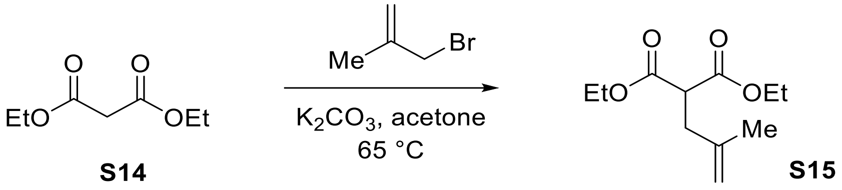

2-(2-methyl-allyl)-malonic acid diethyl ester (**S15**)

The synthesis of 2-(2-methyl-allyl)-malonic acid diethyl ester was performed using a modified literature procedure.[17] A 50-mL round-bottomed flask was charged with potassium carbonate (2.73 g, 19.72 mmol, 2.8 equiv) and equipped with a reflux condenser. The apparatus was evacuated and refilled with argon (3×) using a needle attached to a Schlenk manifold. Acetone (28 mL) was added followed by the addition of diethyl malonate **S14** (1.5 mL, 9.86 mmol, 1.4 equiv) dropwise over 5 min. The mixture was maintained at rt for 10 min followed by the addition of 3-bromo-2-methylpropene (0.71 mL, 7.04 mmol, 1.0 equiv) dropwise over 5 min. The reaction flask was lowered to a pre-heated oil bath at 65 °C and maintained for 18 h under an argon atmosphere. The solids were removed by gravity filtration and washed with ethyl acetate. The filtrate was concentrated via rotary evaporation. Water (50 mL) was added to the residue followed by ethyl acetate (30 mL) and the mixture was transferred to a separatory funnel. The aqueous layer was separated and extracted with ethyl acetate (3×25 mL) and the combined organic layers were washed with brine and dried over magnesium sulfate. The organic layers were concentrated using rotary evaporation and the crude residue was purified by silica gel flash column chromatography (5% ethyl acetate/hexanes) to afford the title compound as a clear oil (1.21 g, 5.65 mmol, 80 %). Spectral data matched that previously reported for compound **S15.**[18]

^{1}H NMR ($CDCl_3$, 400 MHz)

δ 4.78 (s, 1 H), 4.73 (s, 1 H), 4.19 (q, J = 7.1 Hz, 4 H), 3.57 (t, J = 7.8 Hz, 1 H), 2.61 (d, J = 7.8 Hz, 2 H), 1.75 (s, 3 H), 1.26 (t, J = 7.1 Hz, 6 H)

TLC

R_f = 0.37 (10% ethyl acetate/hexanes); silica gel, *p*-anisaldehyde stain

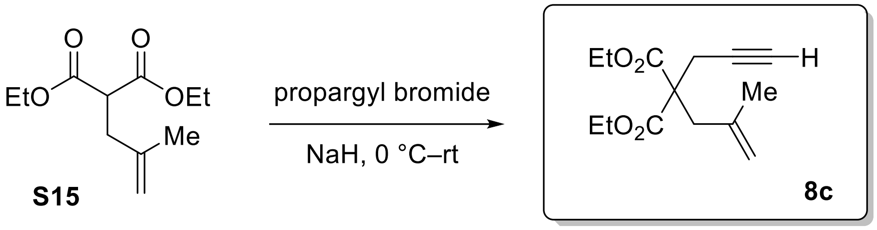

diethyl 2-(2-methylallyl)-2-(prop-2-ynyl)malonate (8c)

Follows General Procedure C: A 25-mL round-bottomed flask was charged with NaH (60% dispersion in mineral oil, 0.17 g, 4.3 mmol, 1.4 equiv) and THF (12 mL) and the flask was placed in an ice-water bath. 2-(2-Methyl-allyl)-malonic acid diethyl ester **S15** (672 mg, 3.14 mmol, 1.0 equiv) dissolved in THF (1.2 mL) was added dropwise to the reaction flask over 10 min. After 1 h, propargyl bromide (60% in toluene, 0.66 mL, 3.83 mmol, 1.2 equiv) was added dropwise. The resulting mixture was slowly warmed to rt and maintained for 18 h. Saturated ammonium chloride (5 mL) was added and the mixture was transferred to a separatory funnel. The aqueous layer was separated and extracted with ethyl acetate (3×25 mL). The combined organic layers were washed with brine (6 mL) and dried over magnesium sulfate. The organic layers were concentrated using rotary evaporation and the crude residue was purified by flash column chromatography on silica gel (5% ethyl acetate/hexanes) to afford the title compound as colorless oil (711 mg, 2.82 mmol, 90%). Spectral data matched that previously reported for compound **8c**.[19]

^{1}H NMR ($CDCl_3$, 400 MHz)

δ 4.92–4.91 (m, 1 H), 4.86–4.86 (m, 1 H), 4.27–4.14 (m, 4 H), 2.84–2.83 (m, 4 H), 2.02 (t, *J* = 2.7 Hz, 1 H), 1.67 (s, 3 H), 1.26 (t, *J* = 7.1 Hz, 6 H)

^{13}C NMR (100.6 MHz, $CDCl_3$)

δ 170.3 (2C), 140.1, 116.4, 79.5, 71.7, 61.8 (2C), 56.5, 39.5, 23.4, 22.7, 14.1 (2C)

TLC

R_f = 0.2 (5% ethyl acetate/hexanes); silica gel, *p*-anisaldehyde stain

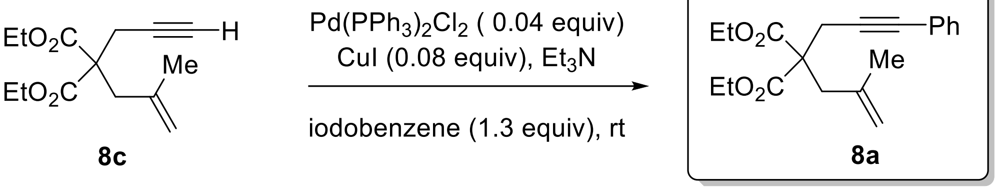

diethyl 6-methyl-1-phenyl-6-hepten-1-yne-4,4-dicarboxylate (8a)

Follows General Procedure B: $Pd(PPh_3)_2Cl_2$ (77.8 mg, 0.11 mmol, 0.04 equiv) and CuI (42.2 mg, 0.22 mol, 0.08 equiv) were weighed into a 25-mL round-bottomed flask. Triethylamine (1.5 mL), iodobenzene (0.4 mL, 3.61 mmol, 1.3 equiv), and enyne **8c** (700 mg, 2.77 mmol, 1.0 equiv) dissolved in THF (1.1 mL) were each added dropwise. The reaction was maintained at rt overnight. A saturated sodium bicarbonate solution (12 mL) was added, and the mixture was transferred to a separatory funnel. The aqueous layer was separated and extracted with dichloromethane (3×30 mL). The combined organic layers were washed with brine (10 mL) and dried over magnesium sulfate. The organic layers were concentrated using rotary evaporation and the crude residue was purified by flash column chromatography on silica gel (5% ethyl acetate/hexanes) to afford the title compound as colorless oil (680 mg, 2.07 mmol, 75%). Spectral data matched that previously reported for compound **8a**.[20]

^{1}H NMR ($CDCl_3$, 400 MHz)

δ 7.38–7.36 (m, 2 H), 7.28–7.27 (m, 3 H), 4.94–4.93 (m, 1 H), 4.90 (s, 1 H), 4.27–4.17 (m, 4 H), 3.05 (s, 2 H), 2.90 (s, 2 H), 1.71 (s, 3 H), 1.27 (t, J = 7.1 Hz, 6 H)

TLC

R_f = 0.4 (5% ethyl acetate/hexanes) [silica gel, UV]

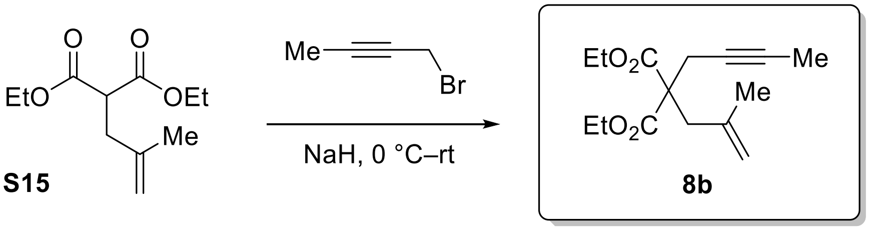

diethyl 2-but-2-ynyl-2-(2-methylallyl)malonate (**8b**)

Follows General Procedure C: A 25-mL round-bottomed flask was charged with NaH (60% dispersion in mineral oil, 0.15 g, 3.8 mmol, 1.3 equiv) and THF (6 mL) and the flask was placed in an ice-water bath. 2-(2-Methyl-allyl)-malonic acid diethyl ester **S15** (600 mg, 2.8 mmol, 1.0 equiv) dissolved in THF (1.5 mL) was added dropwise to the reaction flask over 10 min. After 1 h, 1-bromo-2-butyne (0.28 mL, 3.2 mmol, 1.2 equiv) was added dropwise. The resulting yellowish-brown mixture was allowed to slowly warm to rt and maintained for 18 h. Subsequent workup and purification by flash column chromatography on silica gel (5% ethyl acetate/hexanes) afforded the title compound as colorless oil (560 mg, 2.11 mmol, 75%). Spectral data matched that previously reported for compound **8b**.[8]

^{1}H NMR ($CDCl_3$, 400 MHz)

δ 4.89–4.88 (m, 1 H), 4.83 (s, 1 H), 4.25–4.13 (m, 4 H), 2.81 (s, 2 H), 2.77 (q, J = 2.5 Hz, 2 H), 1.75 (t, J = 2.5 Hz, 3 H), 1.67 (s, 3 H), 1.25 (t, J =7.1 Hz, 6 H)

^{13}C NMR (100.6 MHz, $CDCl_3$)

δ 170.6 (2C), 140.4, 116.1, 79.1, 73.9, 61.6 (2C), 56.8, 39.5, 23.5, 23.1, 14.2 (2C), 3.6 ppm

TLC

R_f = 0.2 (5% ethyl acetate/hexanes); silica gel, *p*-anisaldehyde stain

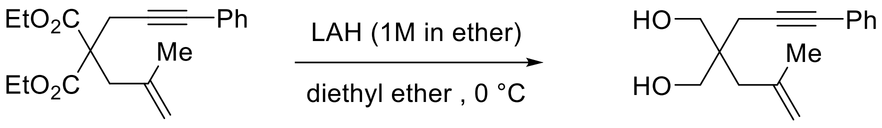

8a **S16**

2-(2-methylallyl)-2-(3-phenylprop-2-yn-1-yl)propane-1,3-diol (**S16**)

A 25-mL round bottomed flask was charged with diethyl 6-methyl-1-phenyl-6-hepten-1-yne-4,4-dicarboxylate **8a** (510 mg, 1.55 mmol, 1.0 equiv) and diethyl ether (4 mL). The flask was cooled in an ice-water bath. A solution of lithium aluminum hydride (1.0 M in ether, 3.1 mL, 3.1 mmol, 2.0 equiv) was added dropwise to the 25-mL reaction flask via a glass syringe. The resulting mixture was maintained at 0 °C for 2 h. Water (1 mL) was added dropwise to the reaction mixture at 0 °C followed by the addition of 15% NaOH (1 mL) and water (3 mL), the mixture was warmed to rt and maintained for additional 20 min. The insoluble material was filtered through a pad of celite. The filtrate was transferred to a separatory funnel and the aqueous layer was separated and extracted with ethyl acetate (3×20 mL). The combined organic layers were washed with brine and dried over magnesium sulfate. The organic layers were concentrated using rotary evaporation and the crude residue was purified by flash column chromatography on silica gel (40% ethyl acetate/hexanes) to afford the title compound **S16** as white solid (307 mg, 1.26 mmol, 81%).

^{1}H NMR ($CDCl_3$, 400 MHz)

δ 7.41–7.39 (m, 2 H), 7.29–7.28 (m, 3 H), 4.96 (s, 1 H), 4.87 (s, 1 H), 3.78–3.70 (m, 4 H), 2.52 (s, 2 H), 2.40 (br s, 2 H), 2.21 (s, 2 H), 1.86 (s, 3 H)

^{13}C NMR (100.6 MHz, $CDCl_3$)

δ 142.2, 131.7 (2C), 128.4 (2C), 128.0, 123.7, 115.7, 86.8, 83.6, 68.1 (2C), 43.4, 39.4, 25.3, 23.0 ppm

HRMS

HRMS-ESI (m/z): $[M + H]^+$ calcd for $C_{16}H_{21}O_2$, 245.1536; found: 245.1538

IR

3353, 1642, 1031 cm^{-1}

mp

83–89℃

TLC

R_f = 0.26 (40% ethyl acetate/hexanes) [silica gel, UV]

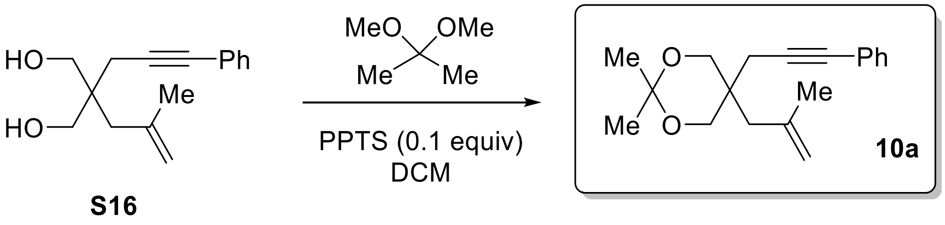

4-(4,4-dimethyl-[3,5]-dioxanyl)-6-methyl-1-phenylhept-6-en-1-yne (10a)

The synthesis of 4-(4,4-dimethyl-[3,5]-dioxanyl)-6-methyl-1-phenylhept-6-en-1-yne was performed in a manner that is analogous to that previously reported.[6] A 25-mL round-bottomed flask was charged with 2-(2-methylallyl)-2-(3-phenylprop-2-yn-1-yl)propane-1,3-diol **S16** (298 mg, 1.22 mmol, 1.0 equiv) and pyridinium *p*-toluenesulfonate (30.7 mg, 0.12 mmol, 0.1 equiv). The reaction flask was evacuated and refilled with argon (3×) through a needle attached to a Schlenk manifold. DCM (4.6 mL) was added followed by dropwise addition of 2,2-dimethoxypropane (0.72 mL, 6.1 mmol, 5.0 equiv). The reaction was maintained at rt for 4 h. Saturated sodium bicarbonate solution (3 mL) was added to the reaction followed by water (9 mL), the mixture was transferred to a separatory funnel and the aqueous layer was extracted with DCM (3×25 mL). The combined organic layers were washed with brine and dried over magnesium sulfate. The organic layers were concentrated using rotary evaporation and the crude residue was purified by flash column chromatography on silica gel (3% ethyl acetate/hexanes) to afford the title compound **10a** as clear oil (347 mg, 1.22 mmol, 100%). Spectral data matched that previously reported for compound **10a**.[21]

^{1}H NMR ($CDCl_3$, 400 MHz)

δ 7.42–7.39 (m, 2 H), 7.30–7.28 (m, 3 H), 4.96–4.96 (m, 1 H), 4.84–4.84 (m, 1 H), 3.75 (s, 4 H), 2.67 (s, 2 H), 2.18 (s, 2 H), 1.84 (s, 3 H), 1.45 (s, 3 H), 1.44 (s, 3 H)

^{13}C NMR (100.6 MHz, $CDCl_3$)

δ 141.0, 131.7 (2C), 128.4 (2C), 127.8, 124.0, 115.7, 98.2, 87.0, 83.6, 67.4 (2C), 40.3, 36.6, 26.4, 25.4, 23.6, 21.6 ppm

HRMS

HRMS-ESI (m/z): $[M + H]^+$ calcd for $C_{19}H_{25}O_2$, 285.1849; found: 285.1855

IR

1643,1068 cm^{-1}

TLC

R_f = 0.2 (3% ethyl acetate/hexanes) [silica gel, UV]

Asymmetric Pauson-Khand Reactions

*General Procedure **D**. Asymmetric Pauson-Khand reactions (Unless otherwise stated, the racemic PKR product was obtained in the same manner using (±)-BINAP)*

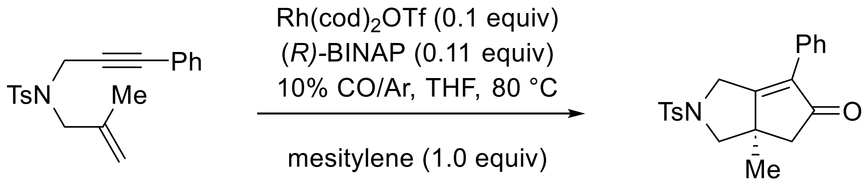

4a **5a**

(*R*)-3a-methyl-6-phenyl-2-tosyl-2,3,3a,4-tetrahydrocyclopenta[*c*]pyrrol-5(*1H*)-one (**5a**)

Mesitylene stock solution preparation.

A 25-mL Schlenk tube equipped with a septum and an argon inlet needle was charged with mesitylene (139 uL, 1.0 mmol) and THF (20.0 mL) to give a stock solution of mesitylene in THF (0.05 M).

Enyne stock solution preparation.

A 15-mL flask equipped with a septum and an argon inlet needle was charged with enyne (61.2 mg, 0.182 mmol) and the prepared solution of mesitylene in THF (0.6 mL) to give a stock solution of enyne in mesitylene/THF (0.3 M enyne/THF).

Asymmetric PKR protocol

A 15-mL flask fused with a reflux condenser (**Figure S1**) was charged with $Rh(cod)_2OTf$ (7.1 mg, 0.015 mmol, 0.1 equiv) and (*R*)-BINAP (10.3 mg, 0.0165 mmol, 0.11 equiv) in a nitrogen-filled glovebox.

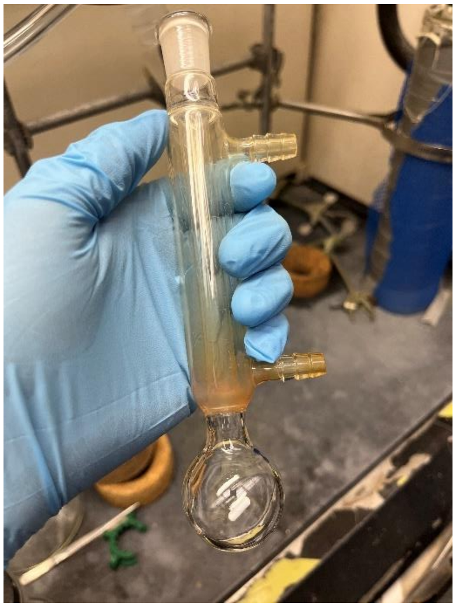

Figure S1. The apparatus (15 mL flask fused with a Liebig reflux condenser (105 mm Jacket length)) used for the Pauson-Khand reactions.

The apparatus was removed from the glovebox and placed under an atmosphere of Ar. The mesitylene/THF stock solution (2.5 mL, containing 0.125 mmol of mesitylene) was added via syringe and the flask was lowered into a preheated oil bath (60 °C), during which time the solution was orange in color. After 1 h, the argon atmosphere was replaced with 10% CO/Ar release cycles (3×) using a needle connected to a vacuum manifold and the reaction was maintained for 1 h at 60 °C, the solution remained orange in color. The flask was removed from the oil bath and allowed to cool to rt. The stock solution of 1,6-enyne **4a** in mesitylene/THF (0.5 mL, containing 0.15 mmol of enyne and 0.025 mmol of mesitylene with a final reaction concentration of 0.05 M) was added via a syringe, and a 0.1 mL of the reaction aliquot was taken

for 0 h timepoint. The flask was lowered into the preheated oil bath (80 °C) and maintained until no starting material remained as evidenced by ^{1}H NMR and TLC (22 h). The PKR yield (99%), b.r.s.m. (99%) and starting material remaining (0%) were determined based on the integral comparison of the product peak (d, 4.62 ppm, 1 H) and starting material peak (s, 4.25 ppm, 2 H) to the internal standard mesitylene peak (s, 6.78 ppm, 3 H) via ^{1}H NMR. Silica gel (0.4 g) was added to the reaction flask and the solvent was removed by rotary evaporation. The resulting residue was chromatographed using a SiO_2 column eluting with 30% ethyl acetate/hexanes to give the title compound **5a** as white solid. Alternatively, purification could be performed using preparative TLC eluting with 30% ethyl/hexanes. HPLC analysis was performed on the purified product using Chiralpak IB N-3 column eluting with 15% IPA/hexanes at a flow rate of 1.0 mL/min and detecting at 254 nm to give the product in 90% *ee*.

- Formula used to calculate b.r.s.m (yield based on recovered starting material)

$$brsm = \frac{PKR\ product\ \%}{100\% - starting\ material\ remaining\%} \times 100\%$$

^{1}H NMR ($CDCl_3$, 400 MHz)

δ 7.71 (d, *J* = 8.0 Hz, 2 H), 7.44-7.36 (m, 5 H), 7.30 (d, *J* = 8.0 Hz, 2 H), 4.62 (d, *J* = 16.6 Hz, 1 H), 4.11 (d, *J* = 16.6 Hz, 1 H), 3.74 (d, *J* = 9.1 Hz, 1 H), 2.84 (d, *J* = 9.1 Hz, 1 H), 2.57 (d, *J* =17.5 Hz, 1 H), 2.47 (d, *J* = 17.5 Hz, 1 H), 2.40 (s, 3 H), 1.27 (s, 3 H)

^{13}C NMR ($CDCl_3$, 100.6 MHz)

δ 205.4, 175.2, 144.1, 134.7, 134.2, 130.1 (2C), 130.0, 129.1, 128.8 (2C), 128.5 (2C), 127.4 (2C), 57.7, 49.4, 47.2, 47.0, 25.5, 21.7

HRMS

HRMS-ESI (m/z): $[M + H]^+$ calcd for $C_{21}H_{22}NO_3S$, 368.1315; found: 368.1305

IR

2965, 1708, 1664, 1343, 1155, 733, 695 cm^{-1}

mp

136–143 ℃

TLC

R_f = 0.26 (30% ethyl acetate/ hexanes); silica gel, UV

$[\alpha]^{20}_{D}$ = -117.2º (c.= 0.6, $CHCl_3$)

<u>HPLC trace (*racemic*):</u>

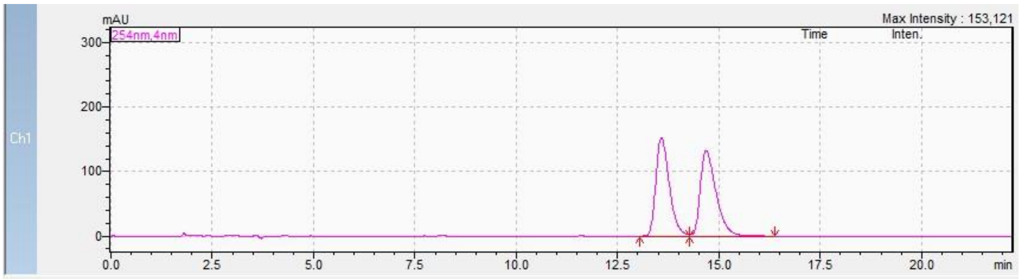

Results View - Peak Table

Peak Table | Compound | Group | Calibration Curve

Peak#	Ret. Time	Area	Height	Mark	Area%
1	13.582	3537328	152959		49.698
2	14.685	3580331	132305	V	50.302
Total		7117659	285264		100.000

<u>HPLC trace (*chiral*):</u>

Chromatogram View Peak Channel Ch1

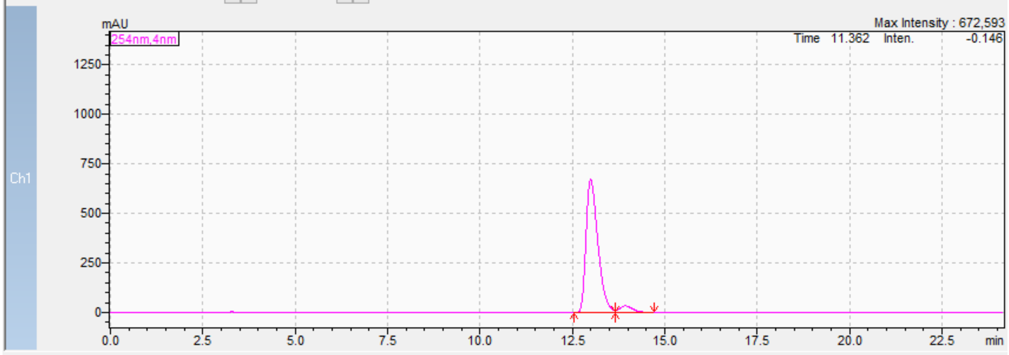

Results View - Peak Table

Peak Table | Compound | Group | Calibration Curve

Peak#	Ret. Time	Area	Height	Mark	Area%
1	12.997	15118127	672911	M	94.861
2	13.939	818951	33633	V M	5.139
Total		15937078	706544		100.000

Extracted 190nm | Ch1 254nm

%*ee*= 94.861-5.139= 89.722 = 90%

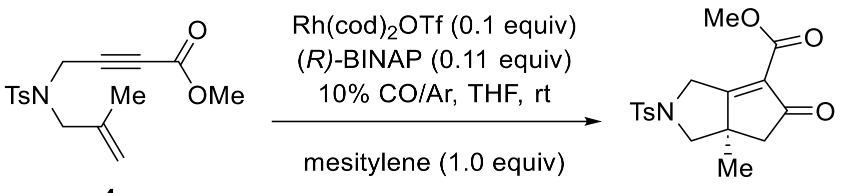

methyl (*R*)-6a-methyl-5-oxo-2-tosyl-1,2,3,5,6,6a-hexahydrocyclopenta[c]pyrrole-4-carboxylate (**5c**)

Follows General Procedure **D** for the asymmetric PKR: Rh(cod)$_2$OTf (7.1 mg, 0.015 mmol, 0.1 equiv), (*R*)-BINAP (10.3 mg, 0.0165 mmol, 0.11 equiv), mesitylene (18 mg, 0.15 mmol, 1.0 equiv) and enyne **4c** (0.5 mL, from a stock solution of 0.3 M) in THF (3 mL, 0.05 M). The reaction was performed at rt. After 20 h, no significant progress was seen as evidenced by ^{1}H NMR. The PKR yield (64%), b.r.s.m. (81%) and starting material remaining (21%) were determined based on the integral comparison of the product peak (d, 4.56 ppm, 1 H) and starting material peak (s, 4.15 ppm, 2 H) to the internal standard mesitylene peak (s, 6.78 ppm, 3 H) via ^{1}H NMR. The crude was purified by prep TLC eluting with 50% ethyl acetate/hexanes (a few drops of acetic acid was added to avoid streaking) to give the title compound as clear oil. HPLC analysis was performed using Chiralpak IH-3 column eluting with 35% IPA/hexanes at a flow rate of 1.0 mL/min and detecting at 230 nm to give the product **5c** in 93% *ee*. Spectral data matched that previously reported for compound **5c**.[5]

^{1}H NMR (CDCl$_3$, 400 MHz)

δ 7.73 (d, *J* = 8.1 Hz, 2 H), 7.34 (d, *J* = 8.1 Hz, 2 H), 4.56 (d, *J* = 19.1 Hz, 1 H), 4.37 (d, *J* = 19.1 Hz, 1 H), 3.81 (s, 3 H), 3.76 (d, *J* = 9.3 Hz, 1 H), 2.82 (d, *J* =9.3 Hz, 1 H), 2.53 (d, *J* = 17.3 Hz, 1 H), 2.44 (s, 3 H), 2.39 (d, *J* = 17.3 Hz, 1 H), 1.27 (s, 3 H)

small impurities at: 7.65–7.63, 4.27–4.24, 4.11 (ethyl acetate), 3.99–3.92, 3.27–3.25, 2.89–2.87, 2.64–2.60, 2.32–2.06, 2.04 (ethyl acetate), 1.67–1.32, 1.07, 0.98–0.78 (grease)

TLC

R_f = 0.25 (50% ethyl acetate/ hexanes); silica gel, UV

HPLC trace (*racemic*):

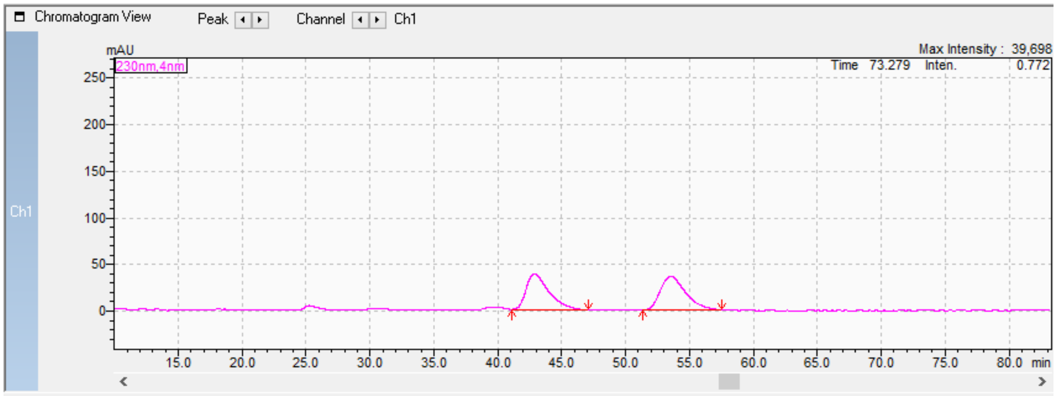

Results View - Peak Table

Peak Table | Compound | Group | Calibration Curve

Peak#	Ret. Time	Area	Height	Mark	Area%
1	42.886	4740841	38075	M	48.514
2	53.498	5031237	36458	M	51.486
Total		9772078	74533		100.000

<u>HPLC trace (*chiral*):</u>

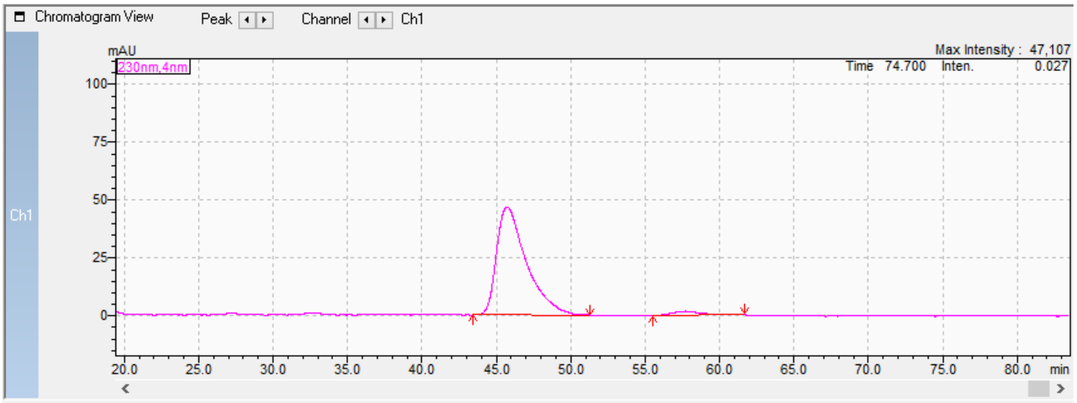

Results View - Peak Table

Peak Table | Compound | Group | Calibration Curve

Peak#	Ret. Time	Area	Height	Mark	Area%
1	45.714	6500725	46685	M	96.268
2	57.687	252023	1793	M	3.732
Total		6752748	48478		100.000

%*ee* = 96.268 – 3.732 = 92.536 = 93%

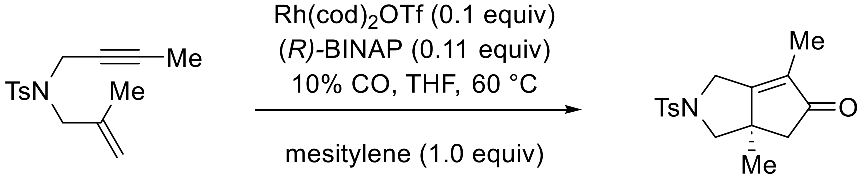

4b **5b**

(*R*)-3a,6-dimethyl-2-tosyl-2,3,3a,4-tetrahydrocyclopenta[c]pyrrol-5(1H)-one (5b)

Follows General Procedure **D** for asymmetric PKR: $Rh(cod)_2OTf$ (7.1 mg, 0.015 mmol, 0.1 equiv), (*R*)-BINAP (10.3 mg, 0.0165 mmol, 0.11 equiv), mesitylene (18 mg, 0.15 mmol, 1.0 equiv) and enyne **4b** (0.5 mL from a stock solution of 0.3 M) in THF (3.0 mL, 0.05 M). The reaction flask was lowered into the preheated oil bath (60 °C). After 20 h, no significant progress was seen as evidenced by ^{1}H NMR. The PKR yield (71%), b.r.s.m. (86%) and starting material remaining (17%) were determined based on the integral comparison of the product peak (d, 4.22 ppm, 1 H) and starting material peak (s, 4.95 ppm, 2 H) to the internal standard mesitylene peak (s, 6.78 ppm, 3 H) via ^{1}H NMR. The crude was purified by SiO_2 chromatography eluting with 20-30% ethyl acetate/hexanes to give the title compound **5b** as white solid. HPLC analysis was performed using Chiralpak AD-3 column eluting with 15% IPA/hexanes at a flow rate of 1.0 mL/min and detecting at 230 nm to give 94% *ee*.

^{1}H NMR ($CDCl_3$, 400 MHz)

δ 7.72 (d, *J* = 8.0 Hz, 2 H), 7.33 (d, *J* = 8.0 Hz, 2 H), 4.22 (d, *J* = 15.6 Hz, 1 H), 4.00 (d, *J* = 15.6, 1 H), 3.64 (d, *J* = 9.0 Hz, 1 H), 2.78 (d, *J* = 9.0 Hz, 1 H), 2.43 (s, 3 H), 2.36 (d, *J* = 17.6 Hz, 1 H), 2.24 (d, *J* = 17.6 Hz, 1H), 1.63 (s, 3H), 1.10 (s, 3 H)

small impurities at 7.56-7.49, 5.29 (DCM)

^{13}C NMR ($CDCl_3$, 100.6 MHz)

δ 207.4, 174.4, 144.1, 134.1, 132.8, 130.0 (2C), 127.5 (2C), 58.3, 47.8, 46.9, 45.6, 25.2, 21.7, 8.8

HRMS

HRMS-ESI (m/z): $[M + H]^+$ calcd for $C_{16}H_{20}NO_3S$, 306.1159; found: 306.1166

IR

1714, 1154 cm^{-1}

m.p

104 °C –122 °C

TLC

R_f = 0.25 (30% ethyl acetate/ hexanes); silica gel, UV

$[\alpha]^{20}_D$ = +28.7° (c.= 0.5, $CHCl_3$)

HPLC trace (*racemic*):

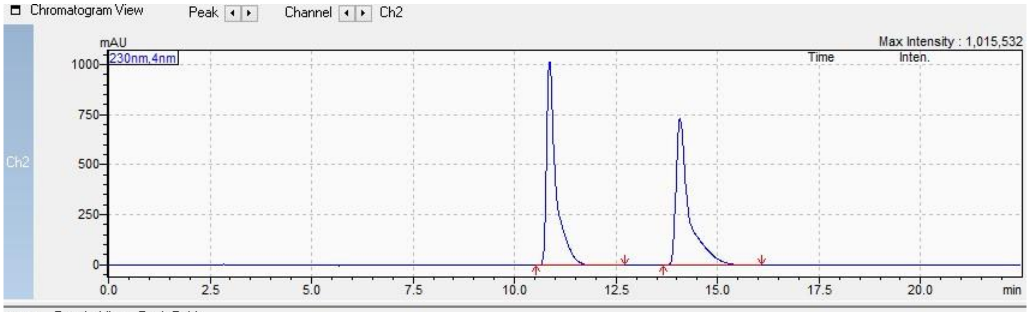

Results View - Peak Table

Peak Table | Compound | Group | Calibration Curve

Peak#	Ret. Time	Area	Height	Mark	Area%
1	10.864	16505806	1019471	S	49.990
2	14.077	16512689	737031		50.010
Total		33018495	1756502		100.000

HPLC trace (*chiral*):

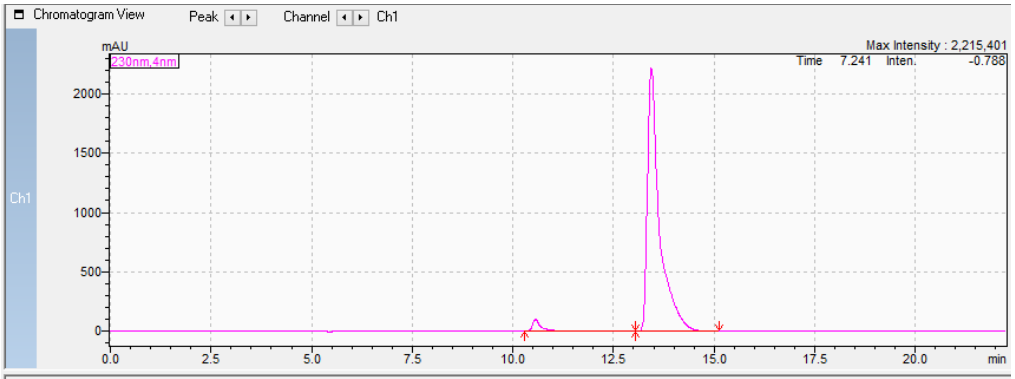

Results View - Peak Table

Peak Table | Compound | Group | Calibration Curve

Peak#	Ret. Time	Area	Height	Mark	Area%
1	10.565	1489669	98635	SV	3.111
2	13.442	46396401	2215999	V	96.889
Total		47886070	2314634		100.000

%*ee* = 96.889 – 3.111 = 93.778 = 94%

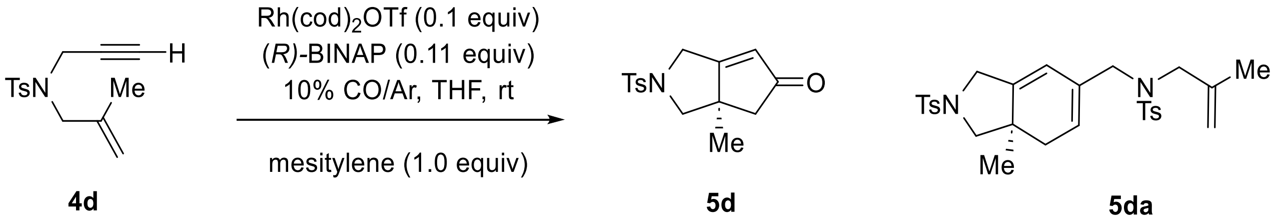

(*R*)-3a-methyl-2-tosyl-2,3,3a,4-tetrahydrocyclopenta[c]pyrrol-5(1H)-one (**5d**)

Follows General Procedure **D** for the asymmetric PKR: Rh(cod)$_2$OTf (7.1 mg, 0.015 mmol, 0.1 equiv), (*R*)-BINAP (10.3 mg, 0.0165 mmol, 0.11 equiv), mesitylene (18 mg, 0.15 mmol, 1.0 equiv) and enyne **4d** (0.5

mL, from a stock solution of 0.30 M) in THF (3 mL, 0.05 M). The reaction was performed at rt. After 25 min, no starting remaining by ^{1}H NMR. The PKR yield (34%), b.r.s.m.. (34%) and starting material remaining (0%), [2+2+2] side product yield (35%) were determined based on the integral comparison of the product peak (dd, 4.33 ppm, 1 H), starting material peak (br s, 4.97 ppm, 2 H) and [2+2+2] side product peak (s, 4.81 ppm, 1H) to the internal standard mesitylene peak (s, 6.78 ppm, 3 H) via ^{1}H NMR. The crude was purified by SiO_2 chromatography eluting with 25-45% ethyl acetate/hexanes to give the title compound **5d** as white solid. HPLC analysis was performed using Chiralpak IB N-3 column eluting with 30% IPA/hexanes at a flow rate of 1.1 mL/min and detecting at 230 nm to give the product **5d** in 99.6% *ee*. Spectral data matched that previously reported for compound **5d**.[22]

<u>^{1}H NMR ($CDCl_3$, 400 MHz)</u>

δ 7.72 (d, *J* = 8.2 Hz, 2 H), 7.34 (d, *J* = 8.2 Hz, 2 H), 5.86 (s, 1 H), 4.33 (dd, *J* = 16.2, 1.4 Hz, 1 H), 4.04 (d, *J* = 16.2 Hz, 1 H), 3.68 (d, *J* = 9.1 Hz, 1 H), 2.83 (d, *J* = 9.1 Hz, 1 H), 2.44 (s, 3 H), 2.37 (d, *J* = 17.5 Hz, 1 H), 2.27 (d, *J* = 17.5 Hz, 1H), 1.18 (s, 3 H)

small impurities at 0.06, 0.00

<u>HRMS</u>

HRMS-ESI (m/z): $[M + H]^+$ calcd for $C_{15}H_{18}NO_3S$, 292.1002; found: 292.0997

<u>TLC</u>

R_f = 0.29 (45% ethyl acetate/ hexanes); silica gel, UV

$[\alpha]^{20}_D$ = 39.8° (c.= 0.5, $CHCl_3$)

<u>HPLC trace (*racemic*):</u>

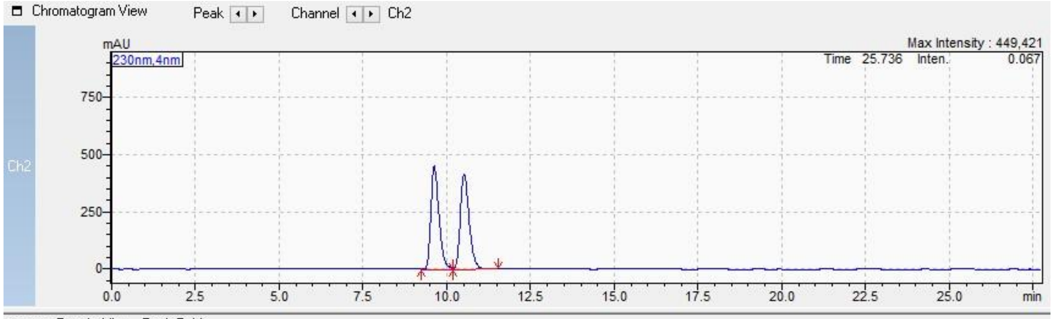

Results View - Peak Table

Peak Table | Compound | Group | Calibration Curve

Peak#	Ret. Time	Area	Height	Mark	Area%
1	9.626	7621869	449945	V	50.026
2	10.518	7613935	416973	V	49.974
Total		15235804	866918		100.000

<u>HPLC trace (*chiral*)</u>

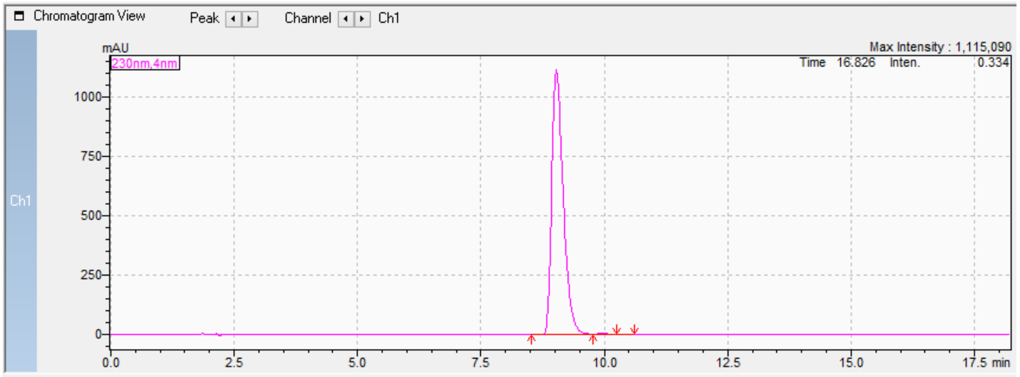

Results View - Peak Table

Peak Table | Compound | Group | Calibration Curve

Peak#	Ret. Time	Area	Height	Mark	Area%
1	9.027	18289438	1114503	S	99.778
2	9.960	40628	3006	T	0.222
Total		18330066	1117509		100.000

%*ee* = 99.778 – 0.222 = 99.556 = 99.6%

Characterization of [2+2+2] cycloaddition side product 5da

^{1}H NMR ($CDCl_3$, 400 MHz)

δ 7.70 (d, *J* = 8.1 Hz, 2 H), 7.66 (d, *J* = 8.1 Hz, 2 H), 7.33 (d, *J* = 8.1 Hz, 2 H), 7.27 (d, J = 8.1 Hz, 2 H), 5.56 (s, 1 H), 5.34 (bs s, 1 H), 4.81 (s, 1 H), 4.71 (s, 1 H), 4.09 (d, *J* = 15.5 Hz, 1 H), 3.80–3.56 (m, 5 H), 3.49 (d, *J* = 9.0 Hz, 1 H), 2.77 (d, *J* = 9.0 Hz, 1H), 2.43 (s, 3 H), 2.41 (s, 3 H), 2.14–1.98 (m, 2 H), 1.54 (s, 3 H), 0.96 (s, 3 H)

Key resonances at 4.81 (s, 1 H), 4.71 (s, 1 H), 4.09 (d, *J* = 15.5 Hz, 1 H), 3.49 (d, *J* = 9.0 Hz, 1 H), 2.77 (d, *J* = 9.0 Hz, 1H), 2.43 (s, 3 H), 2.41 (s, 3 H), 1.54 (s, 3 H), 0.96 (s, 3 H) match or close to a simialr side product reported previsouly.[5]

HRMS

HRMS-ESI (m/z): $[M + H]^+$ calcd for $C_{28}H_{35}N_2O_2S_2$, 527.2033; found: 527.2034

TLC

R_f = 0.26 (30% ethyl acetate/ hexanes); silica gel, UV

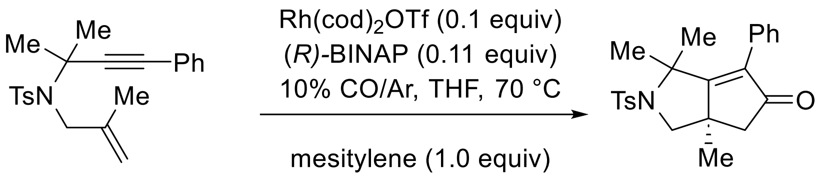

4f **5f**

(*R*)-1,1,3a-trimethyl-6-phenyl-2-tosyl-2,3,3a-4-tetrahydrocyclopenta[*c*]pyrrol-5(1*H*)-one (5f)

Follows General Procedure **D** for asymmetric PKR: $Rh(cod)_2OTf$ (7.1 mg, 0.015 mmol, 0.1 equiv), (*R*)-BINAP (10.3 mg, 0.0165 mmol, 0.11 equiv), mesitylene (18 mg, 0.15 mmol, 1.0 equiv) and enyne **4f** (0.5 mL from a stock solution of 0.3 M) in THF (3.0 mL, 0.05 M). The reaction flask was lowered into the preheated oil bath (70 °C). After 13 h, no starting material remained as evidenced by ^{1}H NMR and TLC. The PKR yield (95%), b.r.s.m.. (95%) and starting material remaining (0%) were determined based on the integral comparison of the product peak (d, 2.93 ppm, 1 H) and starting material peak (m, 5.15–5.14 ppm, 1 H) to the internal standard mesitylene peak (s, 6.78 ppm, 3 H) via ^{1}H NMR. The crude was purified by SiO_2 chromatography eluting with 15–30% ethyl acetate/hexanes to give the title compound **5f** as white solid. HPLC analysis was performed using Chiralpak IB N-3 column eluting with 15% IPA/hexanes at a flow rate of 1.0 mL/min and detecting at 230 nm to give 67% *ee*.

^{1}H NMR ($CDCl_3$, 400 MHz)

δ 7.64 (d, *J* = 8.2 Hz, 2 H), 7.29–7.17 (m, 5 H), 7.07–7.04 (m, 2 H), 3.55 (d, *J* = 9.1 Hz, 1 H), 2.93 (d, *J* = 9.1 Hz, 1 H), 2.42 (d, *J* = 17.3 Hz, 1 H), 2.36 (d, *J* = 17.3 Hz, 1 H), 2.31 (s, 3 H), 1.91 (s, 3 H), 1.31 (s, 3 H), 1.14 (s, 3 H)

^{13}C NMR ($CDCl_3$, 100.6 MHz)

δ 206.3, 185.7, 143.4, 137.5, 136.3, 130.8, 129.7 (2C), 129.3 (2C), 128.7, 128.6 (2C), 127.5 (2C), 66.2, 57.9, 49.2, 46.6, 29.7, 26.6, 24.8, 21.6

HRMS

HRMS-ESI (m/z): $[M + H]^+$ calcd for $C_{23}H_{26}NO_3S$, 396.1628; found: 396.1632

IR

1708, 1150 cm^{-1}

m.p

68 °C –72 °C

TLC

R_f = 0.25 (30% ethyl acetate/ hexanes); silica gel, UV

$[\alpha]^{20}_D$ = -21.0º (c.= 0.5, $CHCl_3$)

HPLC trace (*racemic*):

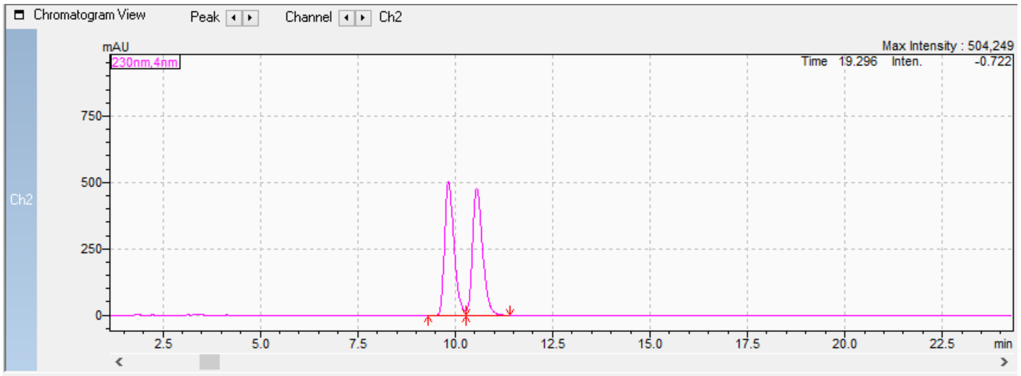

Results View - Peak Table

Peak Table | Compound | Group | Calibration Curve

Peak#	Ret. Time	Area	Height	Mark	Area%
1	9.830	8509696	504817		49.770
2	10.556	8588284	476316	V	50.230
Total		17097980	981133		100.000

HPLC trace (*chiral*):

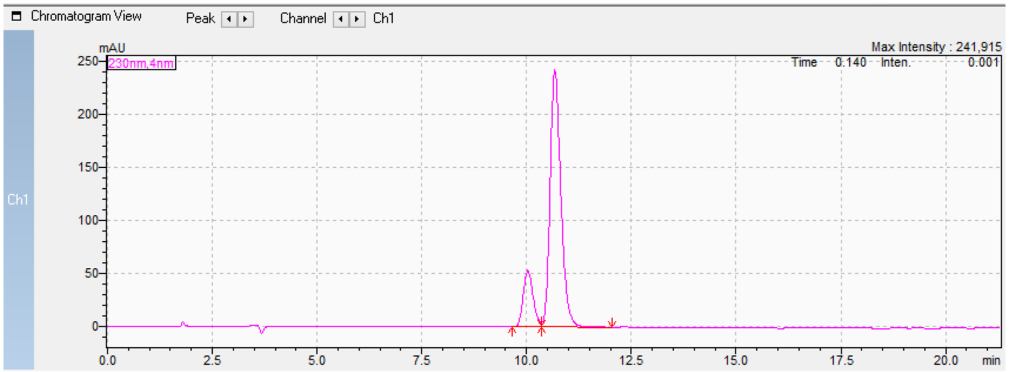

Results View - Peak Table

Peak Table | Compound | Group | Calibration Curve

Peak#	Ret. Time	Area	Height	Mark	Area%
1	10.029	868847	52979		16.718
2	10.673	4328323	242032	V	83.282
Total		5197170	295011		100.000

%*ee* = 83.282 – 16.718 = 66.564 = 67%

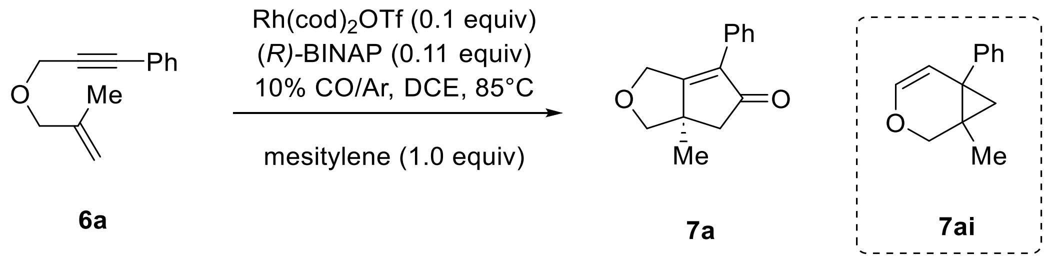

6a **7a** **7ai**

(*R*)-2-phenyl-5-methyl-7-oxabicyclo[.3.0]oct-1-en-3-one (**7a**)

Follows General Procedure **D** for asymmetric PKR: $Rh(cod)_2OTf$ (7.1 mg, 0.015 mmol, 0.1 equiv), (*R*)-BINAP (10.3 mg, 0.0165 mmol, 0.11 equiv), mesitylene (18 mg, 0.15 mmol, 1.0 equiv) and enyne **6a** (0.5 mL from a stock solution of 0.3 M) in DCE (3.0 mL, 0.05 M). The reaction flask was lowered into the preheated oil bath (85 °C). After 23 h, the PKR yield (84%), b.r.s.m. (85%), starting material remaining (1%) and cycloisomerized side product **7ai** (13%) were determined based on the integral comparison of the product peak (d, 4.61 ppm, 1 H), starting material peak (s, 4.36 ppm, 2 H) and cycloisomerized product peak (d, 5.21 ppm, 1 H) to the internal standard mesitylene peak (s, 6.78 ppm, 3 H) via ^{1}H NMR. The crude was purified by SiO_2 chromatography eluting with 20% ethyl acetate/hexanes to give the title compound **7a** as colorless oil. Spectral data matched that previously reported for compound **7a**.[23] HPLC analysis was performed using Chiralpak IH-3 column eluting with 10% IPA/hexanes at a flow rate of 1.0 mL/min and detecting at 254 nm to give 89% *ee*.

<u>^{1}H NMR ($CDCl_3$, 400 MHz)</u>

δ 7.52–7.50 (m, 2 H), 7.43–7.33 (m, 3 H), 4.99 (d, *J* = 16.4 Hz, 1 H), 4.61 (d, *J* = 16.4 Hz, 1 H), 4.04 (d, *J* = 8.1 Hz, 1 H), 3.44 (d, *J* = 8.1 Hz, 1 H), 2.61 (d, *J* = 17.3 Hz, 1 H), 2.55 (d, *J* = 17.3 Hz, 1 H), 1.39 (s, 3 H)

<u>TLC</u>

R_f = 0.22 (20% ethyl acetate/ hexanes); silica gel, UV

$[\alpha]^{20}_D$ = -93.7° (c.= 0.5, $CHCl_3$)

<u>HPLC trace (*racemic*):</u>

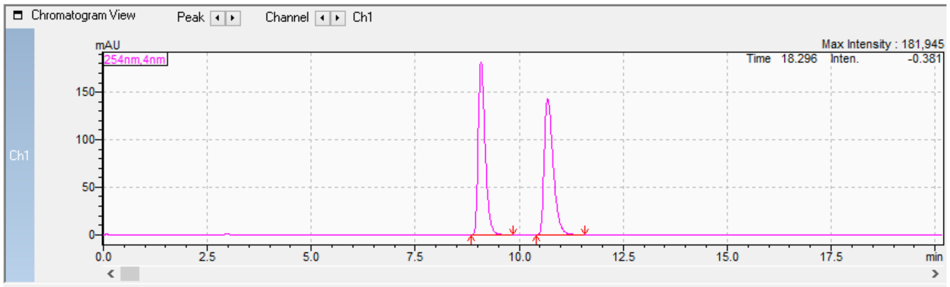

Results View - Peak Table

Peak Table | Compound | Group | Calibration Curve

Peak#	Ret. Time	Area	Height	Mark	Area%
1	9.082	2119954	182086	V	49.990
2	10.685	2120806	143799		50.010
Total		4240760	325884		100.000

<u>HPLC trace (*chiral*):</u>

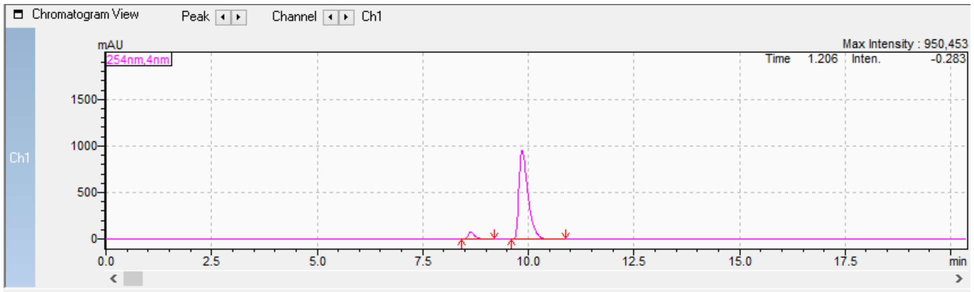

Results View - Peak Table

Peak Table | Compound | Group | Calibration Curve

Peak#	Ret. Time	Area	Height	Mark	Area%
1	8.637	832455	77352		5.572
2	9.854	14108387	950865		94.428
Total		14940842	1028217		100.000

%*ee* = 94.428 – 5.572 = 88.856 = 89%

<u>Characterization of the Cycloisomerized size product 7ai</u> (crude residue was purified by SiO_2 chromatography eluting with 5% EtOAc/hexanes to give the title compound as colorless oil)

1-methyl-6-phenyl-3-oxabicyclo[4.1.0]hept-4-ene (7ai)

<u>^{1}H NMR (400 MHz, $CDCl_3$)</u>

7.33-7.29 (m , 2 H), 7.27–7.20 (m, 3 H), 6.22 (d, *J* = 5.9 Hz, 1 H), 5.21 (d, *J* = 5.9 Hz, 1 H), 4.07 (d, *J* = 10.4 Hz, 1 H), 3.57 (d, *J* = 10.4 Hz, 1 H), 1.40 (d, *J* = 4.6 Hz, 1 H), 1.36 (d, *J* = 4.6 Hz, 1 H), 0.79 (s, 3 H)

<u>^{13}C NMR (100.6 MHz, $CDCl_3$)</u>

141.8, 141.0, 129.1 (2C), 128.2 (2C), 126.3, 111.7, 67.1, 31.3, 27.2, 23.0, 16.9

<u>HRMS</u>

(FTMS + p ESI) [M + H]+ calcd for $C_{13}H_{15}O$: 187.2569, found 187.1122

IR

1638, 1224 cm^{-1}

TLC

R_f = 0.27 (5% ethyl acetate/hexanes) [silica gel, p-anisaldehyde stain]

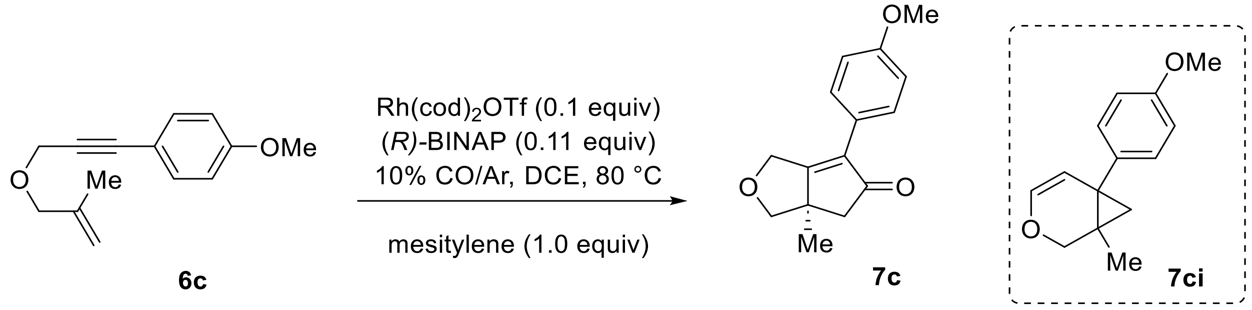

(*R*)-6-(4-methoxyphenyl)-3a-methyl-3a,4-dihydro-1*H*-cyclopenta[c]furan-5(3*H*)-one (**7c**)

Follows General Procedure **D** for asymmetric PKR: Rh(cod)$_2$OTf (7.1 mg, 0.015 mmol, 0.1 equiv), (*R*)-BINAP (10.3 mg, 0.0165 mmol, 0.11 equiv), mesitylene (18 mg, 0.15 mmol, 1.0 equiv) and enyne **6c** (0.5 mL from a stock solution of 0.3 M) in DCE (3.0 mL, 0.05 M). The reaction flask was lowered into the preheated oil bath (80 °C). After 16 h, the PKR yield (63%), b.r.s.m. (63%), starting material remaining (0%) and cycloisomerized side product **7ci** (12%) were determined based on the integral comparison of the product peak (d, 4.57 ppm, 1 H), starting material peak (s, 4.34 ppm, 2 H) and cycloisomerized product peak (d, 5.15 ppm, 1 H) to the internal standard mesitylene peak (s, 2.25 ppm, 9 H) via ^{1}H NMR. The crude was purified by SiO_2 chromatography eluting with 10–25% ethyl acetate/hexanes to give the title compound **7c** as light-yellow solid. HPLC analysis was performed using Chiralpak IH-3 column eluting with 10% IPA/hexanes at a flow rate of 1.0 mL/min and detecting at 230 nm to give 70% *ee*.

^{1}H NMR ($CDCl_3$, 400 MHz)

δ 7.46 (d, *J* = 8.8 Hz, 2 H), 6.92 (d, *J* = 8.8 Hz, 2 H), 4.95 (d, *J* = 16.3 Hz, 1 H), 4.57 (d, *J* = 16.3 Hz, 1 H), 4.01 (d, *J* = 8.0 Hz, 1 H), 3.81 (s, 3 H), 3.40 (d, *J* = 8.0 Hz, 1 H), 2.57 (d, *J* = 17.3 Hz, 1 H), 2.51 (d, *J* = 17.3 Hz, 1 H), 1.36 (s, 3 H)

^{13}C NMR ($CDCl_3$, 100.6 MHz)

δ 207.2, 178.6, 159.9, 132.8, 129.6 (2C), 123.3, 114.2 (2C), 76.7, 65.5, 55.4, 48.7, 47.8, 24.9

HRMS

HRMS-ESI (m/z): $[M + H]^+$ calcd for $C_{15}H_{17}O_3$, 245.1172; found: 245.1175

IR

1702, 1023 cm^{-1}

m.p

91 ℃–95 ℃

TLC

R_f = 0.21 (25% ethyl acetate/ hexanes); silica gel, UV

$[\alpha]^{20}_D$ = -54.0° (c.= 0.5, $CHCl_3$)

HPLC trace (*racemic*):

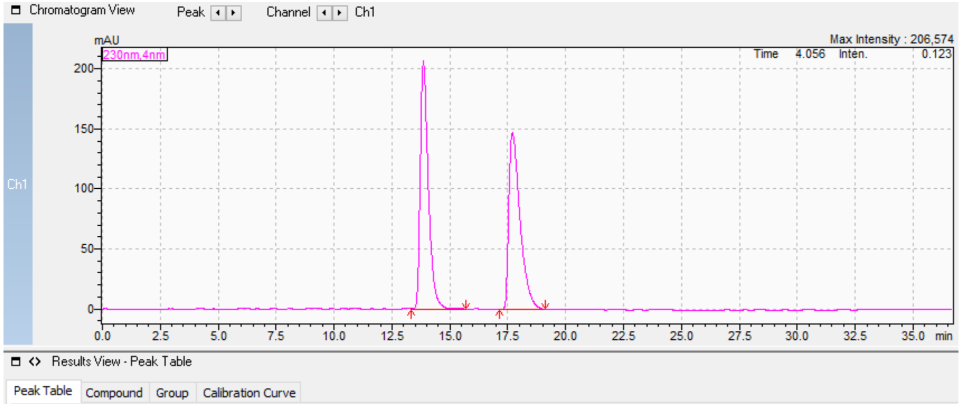

Peak#	Ret. Time	Area	Height	Mark	Area%
1	13.856	4984265	206661	V	50.061
2	17.704	4972195	146919		49.939
Total		9956460	353581		100.000

HPLC trace (*chiral*):

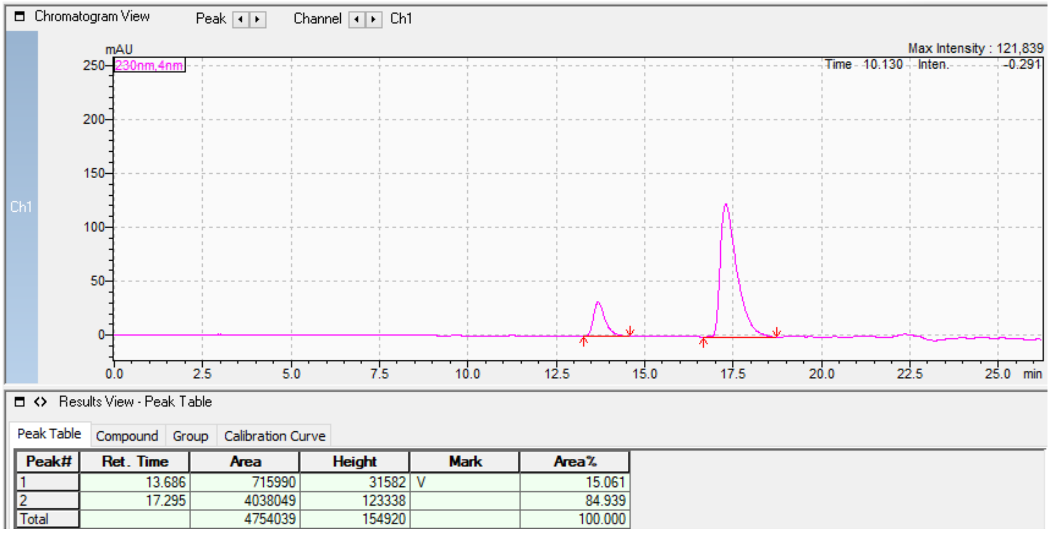

Peak#	Ret. Time	Area	Height	Mark	Area%
1	13.686	715990	31582	V	15.061
2	17.295	4038049	123338		84.939
Total		4754039	154920		100.000

%*ee* = 84.939 – 15.061 = 69.878 = 70%

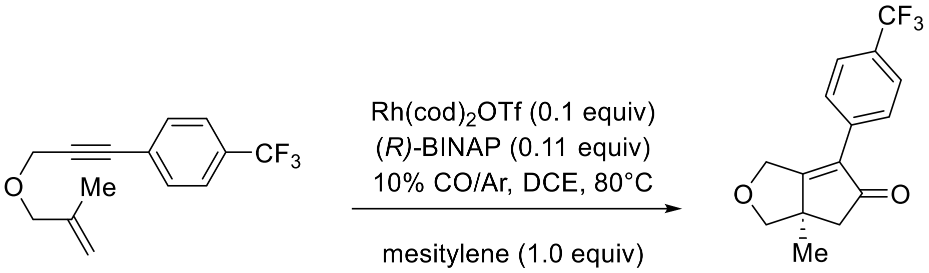

6d **7d**

(*R*)-3a-methyl-6-(4-trifluoromethyl)phenyl)-3a,4-dihydro-1H-cyclopental[c]furan-5(3H)-one (7d)

Follows General Procedure **D** for asymmetric PKR: Rh(cod)$_2$OTf (7.1 mg, 0.015 mmol, 0.1 equiv), (*R*)-BINAP (10.3 mg, 0.0165 mmol, 0.11 equiv), mesitylene (18 mg, 0.15 mmol, 1.0 equiv) and enyne **6d** (0.5 mL from a stock solution of 0.3 M) in DCE (3.0 mL, 0.05 M). The reaction flask was lowered into the preheated oil bath (80 °C). After 20 h, the PKR yield (90%), b.r.s.m. (92%), starting material remaining (3%) were determined based on the integral comparison of the product peak (d, 4.59 ppm, 1 H) and starting material peak (s, 4.37 ppm, 2 H) to the internal standard mesitylene peak (s, 6.78 ppm, 3 H) via ^{1}H NMR. The crude was purified by SiO$_2$ chromatography eluting with 10–25% ethyl acetate/hexanes to give the title compound **7d** as white solid. HPLC analysis was performed using Chiralpak IB N-3 column eluting with 10% IPA/hexanes at a flow rate of 1.0 mL/min and detecting at 254 nm to give 75% *ee*.

^{1}H NMR ($CDCl_3$, 400 MHz)

δ 7.66–7.61 (m, 4 H), 5.00 (d, *J* = 16.6 Hz, 1 H), 4.59 (d, *J* = 16.6 Hz, 1 H), 4.05 (d, *J* = 8.1 Hz, 1 H), 3.45 (d, *J* = 8.1 Hz, 1 H), 2.63 (d, *J* = 17.3 Hz, 1 H), 2.56 (d, *J* = 17.3 Hz, 1 H), 1.40 (s, 3 H)

^{13}C NMR ($CDCl_3$, 100.6 MHz)

δ 206.2, 183.0, 134.1, 132.1, 130.5 (q, *J* = 32.5 Hz), 128.5 (2C), 125.6 (2C, q, *J* = 3.7 Hz), 124.1 (q, *J* = 273.2 Hz), 76.6, 65.4, 48.7, 48.4, 24.9 ppm

HRMS

HRMS-ESI (m/z): $[M + H]^+$ calcd for $C_{15}H_{14}O_2F_3$, 283.0940; found: 283.0951

IR

1710 cm^{-1}

m.p

107 °C–114 °C

$[\alpha]^{20}_D$ = 10.0º (c.= 0.5, $CHCl_3$)

TLC

R_f = 0.29 (30% ethyl acetate/ hexanes); silica gel, UV

HPLC trace (*racemic*):

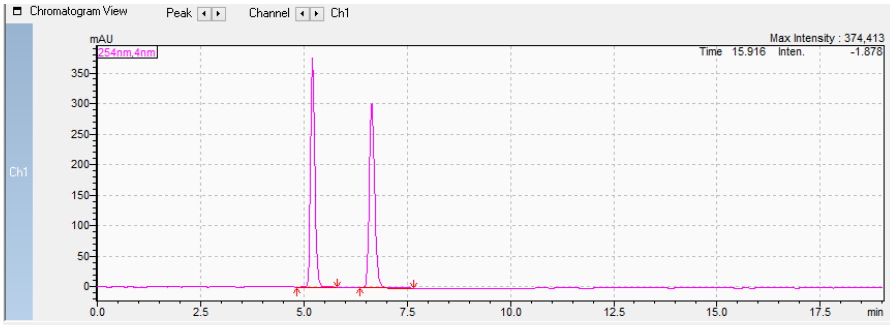

Results View - Peak Table

Peak Table | Compound | Group | Calibration Curve

Peak#	Ret. Time	Area	Height	Mark	Area%
1	5.208	2737864	375284	S	50.143
2	6.642	2722274	300468		49.857
Total		5460138	675752		100.000

HPLC trace (*chiral*):

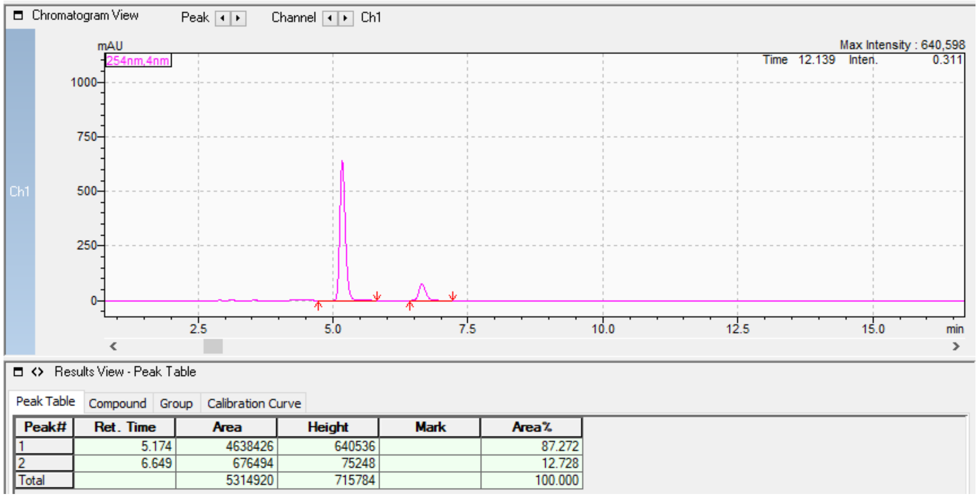

Peak#	Ret. Time	Area	Height	Mark	Area%
1	5.174	4638426	640536		87.272
2	6.649	676494	75248		12.728
Total		5314920	715784		100.000

%*ee* = 87.272 – 12.728 = 74.544= 75%

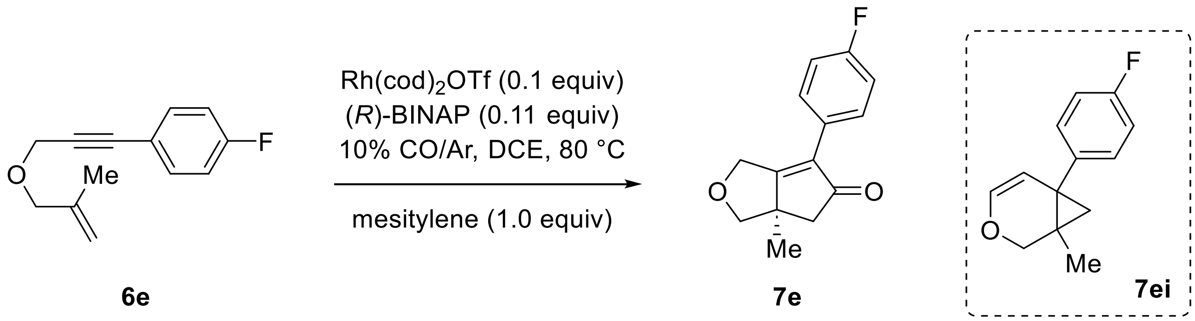

(*R*)-6-(4-fluorophenyl)-3a-methyl-3a,4-dihydro-1*H*-cyclopenta[*c*]furan-5(3*H*)-one (7e)

Follows General Procedure **D** for asymmetric PKR: $Rh(cod)_2OTf$ (7.1 mg, 0.015 mmol, 0.1 equiv), (*R*)-BINAP (10.3 mg, 0.0165 mmol, 0.11 equiv), mesitylene (18 mg, 0.15 mmol, 1.0 equiv) and enyne **6e** (0.5 mL from a stock solution of 0.3 M) in DCE (3.0 mL, 0.05 M). The reaction flask was lowered into the preheated oil bath (80 °C). After 21 h, no significant progress was observed based on ^1H NMR. The PKR yield (76%), b.r.s.m. (81%), starting material remaining (6%) and cycloisomerized side product **7ei** (5%) were determined based on the integral comparison of the product peak (d, 4.58 ppm, 1 H), starting material peak (s, 4.33 ppm, 2 H) and cycloisomerized product peak (d, 5.13 ppm, 1 H) to the internal standard mesitylene peak (s, 6.78 ppm, 3 H) via ^1H NMR. The crude was purified by SiO_2 chromatography eluting with 10–25% ethyl acetate/hexanes to give the title compound **7e** as light-yellow oil. HPLC analysis was performed using Chiralpak IB-N3 column eluting with 10% IPA/hexanes at a flow rate of 1.0 mL/min and detecting at 254 nm to give 69% *ee*.

^1H NMR ($CDCl_3$, 400 MHz)

δ 7.52–7.49 (m, 2 H), 7.12-7.07 (m, 2 H), 4.96 (d, J = 16.4 Hz, 1 H), 4.58 (d, J = 16.4 Hz, 1 H), 4.03 (d, J = 8.2 Hz, 1 H), 3.43 (d, J = 8.2 Hz, 1 H), 2.60 (d, J = 17.3 Hz, 1 H), 2.53 (d, J = 17.3 Hz, 1 H), 1.38 (s, 3 H)

^{13}C NMR ($CDCl_3$, 100.6 MHz)

δ 206.7, 180.5 (d, J = 1.4 Hz), 163.0 (d, J =249.6 Hz), 132.3, 130.0 (2C, d, J = 8.1 Hz), 126.9 (q, J = 3.2 Hz), 115.8 (2C, d, J = 21.9 Hz), 76.7, 65.4, 48.7, 48.0, 24.9

HRMS

HRMS-ESI (m/z): $[M + H]^+$ calcd for $C_{14}H_{14}FO_2$, 233.0972; found: 233.0972

IR

1705 cm^{-1}

TLC

R_f = 0.26 (25% ethyl acetate/ hexanes); silica gel, UV

$[\alpha]^{20}_D$ = -50.8° (c.= 0.5, $CHCl_3$)

HPLC trace (*racemic*):

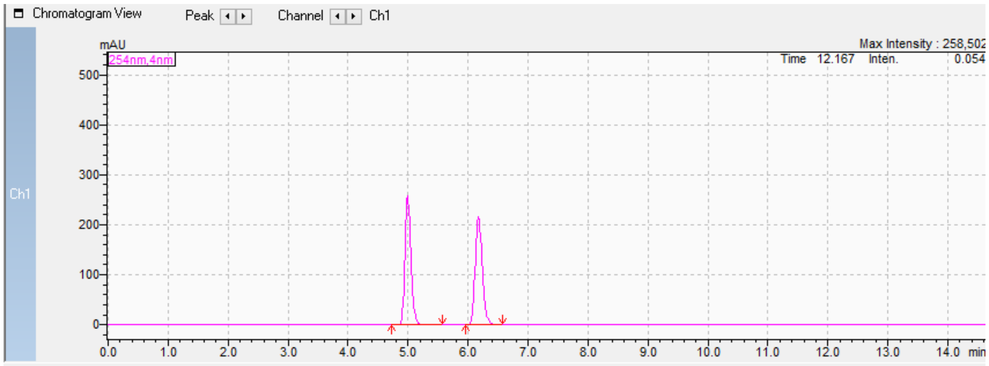

Results View - Peak Table

Peak Table | Compound | Group | Calibration Curve

Peak#	Ret. Time	Area	Height	Mark	Area%
1	4.992	1811793	259140		50.376
2	6.173	1784758	216431	V	49.624
Total		3596551	475570		100.000

HPLC trace (*chiral*):

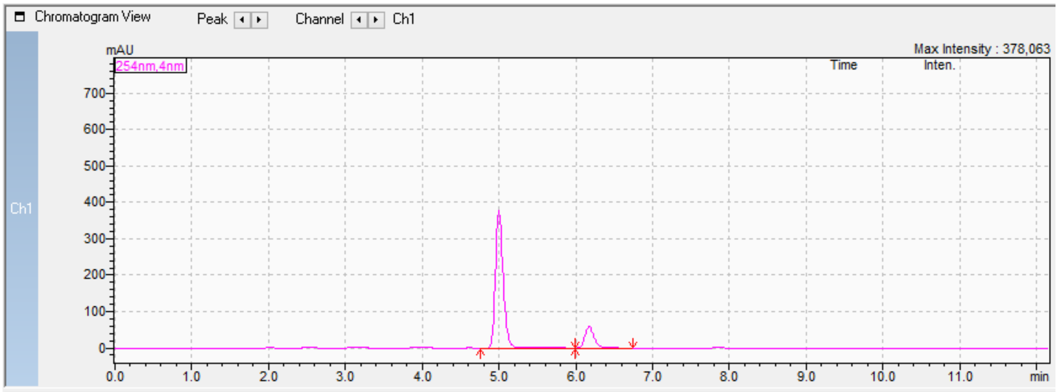

Peak#	Ret. Time	Area	Height	Mark	Area%
1	4.998	2649509	378461	S	84.262
2	6.177	494868	59216	V	15.738
Total		3144377	437677		100.000

%*ee* = 84.262 – 15.738 = 68.524 = 69%

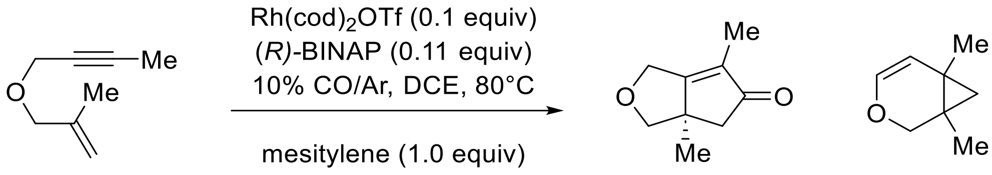

6b **7b** **7bi**

(*R*)-3a,6-dimethyl-3a,4-dihydro-1H-cyclopenta[c]furan-5(3H)-one (**7b**)

Follows General Procedure **D** for asymmetric PKR: Rh(cod)$_2$OTf (7.1 mg, 0.015 mmol, 0.1 equiv), (*R*)-BINAP (10.3 mg, 0.0165 mmol, 0.11 equiv), mesitylene (18 mg, 0.15 mmol, 1.0 equiv) and enyne **6b** (0.5 mL from a stock solution of 0.3 M) in DCE (3.0 mL, 0.05 M). The reaction flask was lowered into the preheated oil bath (80 °C). After 27 h, the PKR yield (66%), b.r.s.m. (66%), starting material remaining (trace) and cycloisomerized side product **7bi** (25%) were determined based on the integral comparison of the product peak (dd, 4.61 ppm, 1 H), starting material peak (m, 4.98–4.98 ppm, 1 H) and cycloisomerized product peak (d, 6.04 ppm, 1 H) to the internal standard mesitylene peak (s, 6.78 ppm, 3 H) via ^{1}H NMR. The crude was purified by SiO_2 chromatography eluting with 15–25% ethyl acetate/hexanes to give the title compound **7b** as colorless oil. HPLC analysis was performed using Chiralpak IH-3 column eluting with 10% IPA/hexanes at a flow rate of 1.0 mL/min and detecting at 230 nm to give 75% *ee*.

<u>^{1}H NMR ($CDCl_3$, 400 MHz)</u>

δ 4.61 (dd, J = 15.5, 1.0 Hz, 1 H), 4.46 (d, J = 15.5, 1 H), 3.94 (d, J = 8.0 Hz, 1 H), 3.35 (d, J = 8.0 Hz, 1 H), 2.39 (d, J = 17.4 Hz, 1 H), 2.30 (d, J = 17.4 Hz, 1 H), 1.70 (s, 3 H), 1.25 (s, 3 H)

<u>^{13}C NMR ($CDCl_3$, 100.6 MHz)</u>

δ 208.9, 179.6, 131.3, 77.2, 63.8, 47.9, 47.2, 24.7, 9.0

<u>HRMS</u>

HRMS-ESI (m/z): $[M + H]^+$ calcd for $C_9H_{13}O_2$, 153.0910; found: 153.0909

<u>IR</u>

1713 cm^{-1}

<u>TLC</u>

R_f = 0.2 (25% ethyl acetate/ hexanes); silica gel, UV

$[\alpha]^{20}_D$ = 36.3º (c.= 0.5, $CHCl_3$)

<u>HPLC trace (*racemic*):</u>

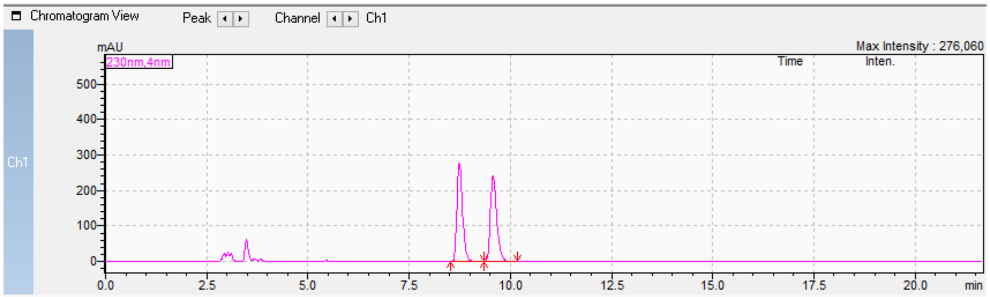

Results View - Peak Table

Peak Table | Compound | Group | Calibration Curve

Peak#	Ret. Time	Area	Height	Mark	Area%
1	8.740	2676970	276426	V	50.006
2	9.571	2676333	242587	V	49.994
Total		5353304	519012		100.000

<u>HPLC trace (*chiral*):</u>

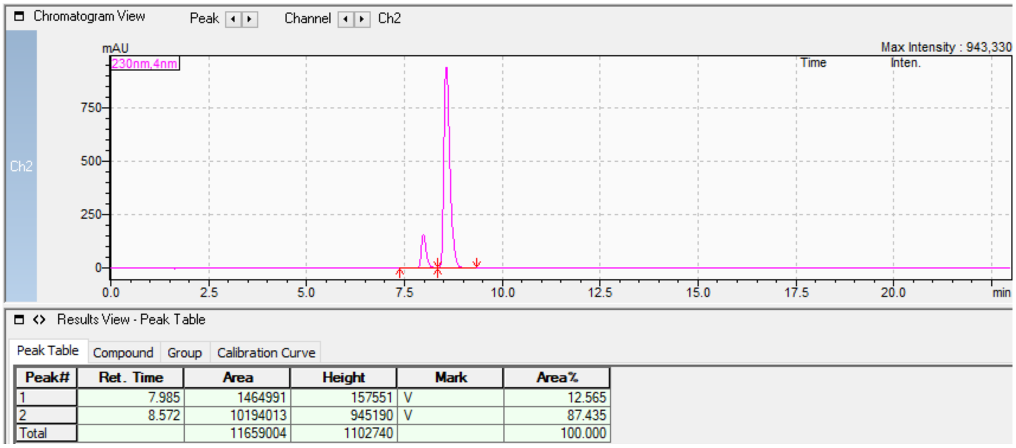

Peak#	Ret. Time	Area	Height	Mark	Area%
1	7.985	1464991	157551	V	12.565
2	8.572	10194013	945190	V	87.435
Total		11659004	1102740		100.000

%*ee* = 87.435 – 12.565 = 74.87 = 75%

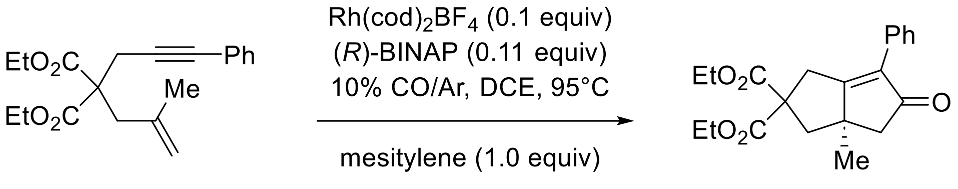

8a **9a**

diethyl-(*R*)-3a-methyl-5-oxo-6-phenyl-3,3a,4,5-tetrahydropentalene-2,2-(1*H*)-dicarboxylate (9a)

Follows General Procedure **D** for asymmetric PKR: $Rh(cod)_2BF_4$ (6.1 mg, 0.015 mmol, 0.1 equiv), (*R*)-BINAP (10.3 mg, 0.0165 mmol, 0.11 equiv), mesitylene (18 mg, 0.15 mmol, 1.0 equiv) and enyne **8a** (0.5 mL from a stock solution of 0.3 M) in DCE (3.0 mL, 0.05 M). The reaction flask was lowered into the preheated oil bath (95 °C). After 20 h, the PKR yield (94%), b.r.s.m. (94%) and starting material remaining (0%) were determined based on the integral comparison of the product peak (d, 2.63 ppm, 1 H) and starting material peak (m, 4.94–4.93 ppm, 1 H) to the internal standard mesitylene peak (s, 6.78 ppm, 3 H) via ^1H NMR. The crude was purified by SiO_2 chromatography eluting with 20% ethyl acetate/hexanes to give the title compound as colorless oil. Spectral data matched that previously reported for compound **9a**.[24] HPLC analysis was performed using Chiralpak IH-3 column eluting with 3% IPA/hexanes at a flow rate of 0.9 mL/min and detecting at 254 nm to give 80% *ee*.

<u>^1H NMR ($CDCl_3$, 400 MHz)</u>

δ 7.52–7.50 (m, 2 H), 7.42–7.31 (m, 3 H), 4.29 (q, *J* = 7.1 Hz, 2 H), 4.18–4.04 (m, 2 H), 3.79 (d, *J* = 18.3 Hz, 1 H), 3.27 (d, *J* = 18.3 Hz, 1 H), 2.63 (d, *J* = 13.7 Hz, 1 H), 2.58 (s, 2 H), 2.29 (d, *J* = 13.7 Hz, 1 H), 1.30 (t, *J* = 7.1 Hz, 3 H), 1.22 (s, 3 H), 1.16 (t, *J* = 7.1 Hz, 3 H)

^{13}C NMR ($CDCl_3$, 100.6 MHz)

δ 207.1, 182.2, 171.9, 171.3, 134.3, 130.9, 128.8 (2C), 128.5 (2C), 128.3, 62.4, 62.2, 60.9, 52.5, 48.0, 44.4, 34.8, 27.0, 14.2, 14.0

HRMS

HRMS-ESI (m/z): $[M + H]^+$ calcd for $C_{21}H_{25}O_5$, 357.1697; found: 357.1699

TLC

R_f = 0.17 (20% ethyl acetate/ hexanes); silica gel, UV

$[\alpha]^{20}_D$ = 25.0° (c.= 0.5, $CHCl_3$)

HPLC trace (*racemic*):

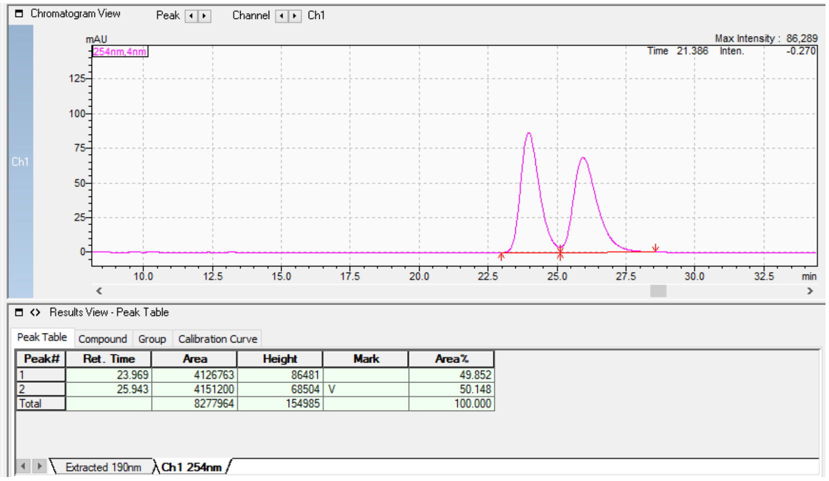

Peak#	Ret. Time	Area	Height	Mark	Area%
1	23.969	4126763	86481		49.852
2	25.943	4151200	68504	V	50.148
Total		8277964	154985		100.000

HPLC trace (*chiral*):

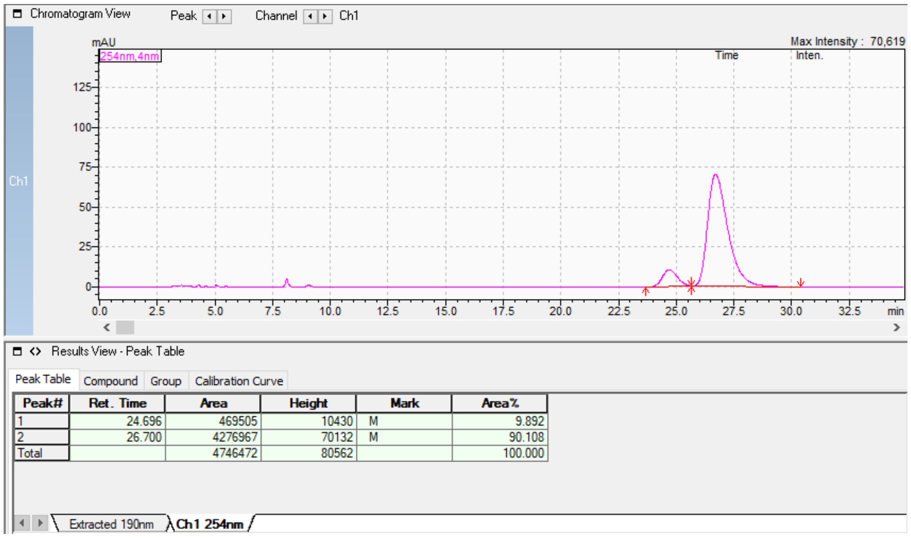

Peak#	Ret. Time	Area	Height	Mark	Area%
1	24.696	469505	10430	M	9.892
2	26.700	4276967	70132	M	90.108
Total		4746472	80562		100.000

%*ee* = 90.108 – 9.892 = 80.216 = 80%

****Follows General Procedure D except the PKR was performed in THF:*

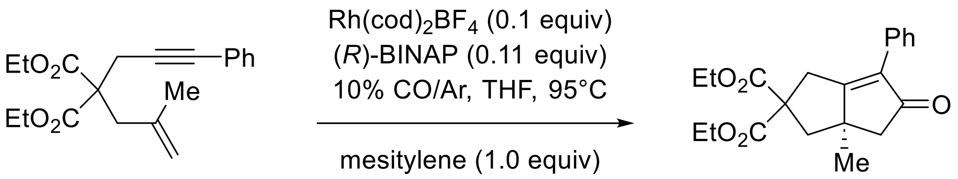

8a **9a**

diethyl-(*R*)-3a-methyl-5-oxo-6-phenyl-3,3a,4,5-tetrahydropentalene-2,2-(1*H*)-dicarboxylate (9a)

Follows General Procedure **D** for asymmetric PKR: $Rh(cod)_2BF_4$ (6.1 mg, 0.015 mmol, 0.1 equiv), (*R*)-BINAP (10.3 mg, 0.0165 mmol, 0.11 equiv), mesitylene (18 mg, 0.15 mmol, 1.0 equiv) and enyne **8a** (0.5 mL from a stock solution of 0.3 M) in THF (3.0 mL, 0.05 M). The reaction flask was lowered into the preheated oil bath (95 °C). After 20 h, the PKR yield (2%), b.r.s.m. (20%) and starting material remaining (90%) were determined based on the integral comparison of the product peak (d, 2.63 ppm, 1 H) and starting material peak (m, 4.94–4.93 ppm, 1 H) to the internal standard mesitylene peak (s, 6.78 ppm, 3 H) via ^1H NMR. The crude was purified by SiO_2 chromatography eluting with 20% ethyl acetate/hexanes to give the title compound as colorless oil. Spectral data matched that previously reported for compound **9a**.[24] HPLC analysis was performed using Chiralpak IH-3 column eluting with 3% IPA/hexanes at a flow rate of 0.9 mL/min and detecting at 254 nm to give 56% *ee*.

<u>HPLC trace (*chiral*):</u>

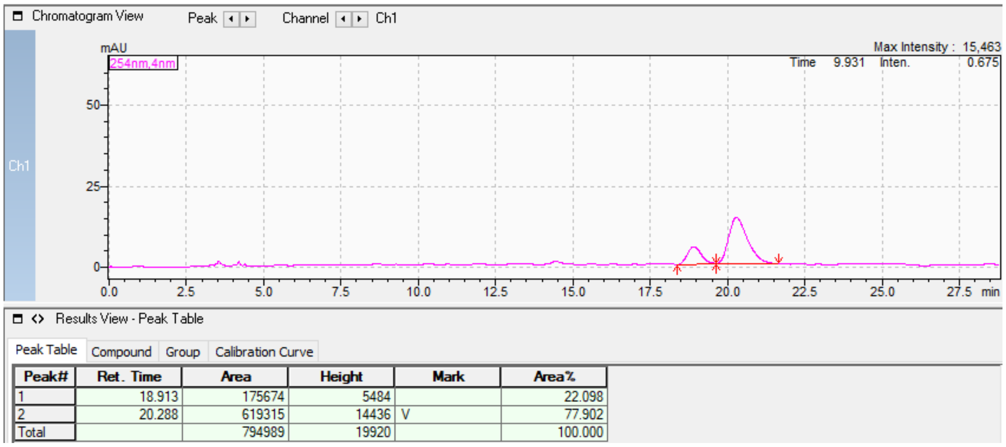

Peak#	Ret. Time	Area	Height	Mark	Area%
1	18.913	175674	5484		22.098
2	20.288	619315	14436	V	77.902
Total		794989	19920		100.000

%*ee* = 77.902 – 22.098 = 55.804 = 56%

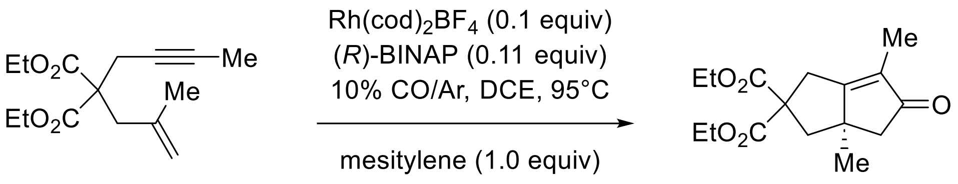

8b **9b**

Diethyl-(*R*)-3a,6-dimethyl-5-oxo-3,3a,4,5-tetrahydropentalene-2,2(1*H*)-dicarboxylate (9b)

Follows General Procedure **D** for asymmetric PKR: $Rh(cod)_2BF_4$ (6.1 mg, 0.015 mmol, 0.1 equiv), (*R*)-BINAP (10.3 mg, 0.0165 mmol, 0.11 equiv), mesitylene (18 mg, 0.15 mmol, 1.0 equiv) and enyne **8b** (0.5 mL from a stock solution of 0.3 M) in DCE (3.0 mL, 0.05 M). The reaction flask was lowered into the preheated oil bath (95 °C). After 15 h, the PKR yield (93%), b.r.s.m. (93%) and starting material remaining (trace) were determined based on the integral comparison of the product peak (d, 2.58 ppm, 1 H) and starting material peak (m, 4.89–4.88 ppm, 1 H) to the internal standard mesitylene peak (s, 6.78 ppm, 3 H) via ^{1}H NMR. The crude was purified by SiO_2 chromatography eluting with 15%–20% ethyl acetate/hexanes to give the title compound **9b** as colorless oil. HPLC analysis was performed using Chiralpak IH-3 column eluting with 15% IPA/hexanes at a flow rate of 1.0 mL/min and detecting at 230 nm to give 55% *ee*

^{1}H NMR ($CDCl_3$, 400 MHz)

δ 4.25 (q, *J* = 7.2 Hz, 2 H), 4.19–4.13 (m, 2 H), 3.36 (dd, *J* = 17.5, 1.3 Hz, 1 H), 3.15 (d, *J* = 17.5 Hz, 1 H), 2.58 (d, *J* = 13.6 Hz, 1 H), 2.39 (d, *J* = 17.5 Hz, 1 H), 2.32 (d, *J* = 17.5 Hz, 1 H), 2.11 (d, *J* = 13.6 Hz, 1 H) , 1.69–1.68 (m, 3 H), 1.28 (t, *J* = 7.2 Hz, 3 H), 1.22 (t, *J* = 7.2 Hz, 3 H), 1.10 (s, 3 H)

^{13}C NMR ($CDCl_3$, 100.6 MHz)

δ 209.4, 181.0, 171.9, 171.7, 131.7, 62.2, 62.1, 60.4, 51.2, 47.7, 44.8, 33.1, 26.7, 14.2 (2C), 8.6

HRMS

HRMS-ESI (m/z): $[M + H]^+$ calcd for $C_{16}H_{23}O_5$, 295.1540; found: 295.1538

IR

1729, 1708 cm^{-1}

TLC

R_f = 0.25 (20% ethyl acetate/ hexanes); silica gel, UV

$[\alpha]^{20}_D = 45.4^o$ (c.= 0.5, $CHCl_3$)

HPLC trace (*racemic*):

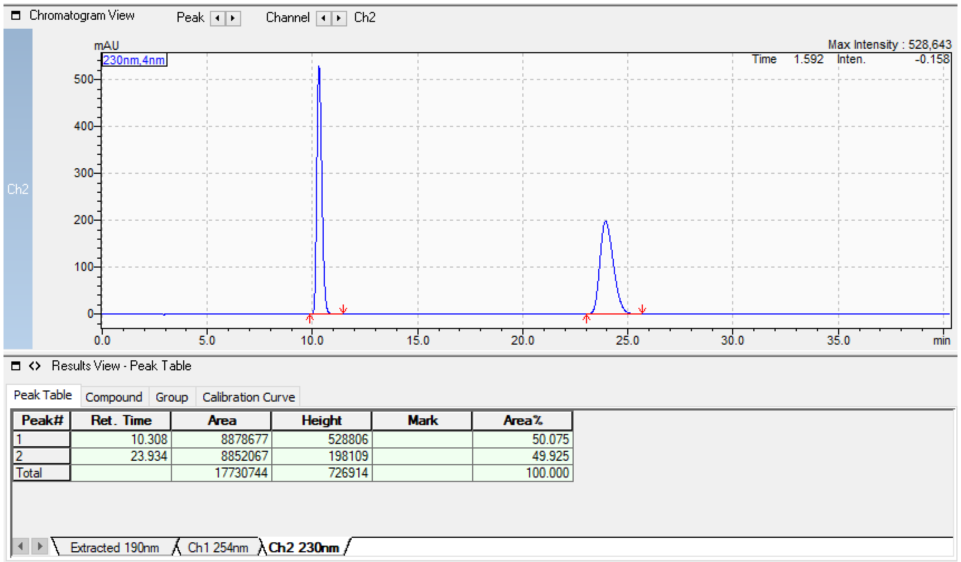

Peak#	Ret. Time	Area	Height	Mark	Area%
1	10.308	8878677	528806		50.075
2	23.934	8852067	198109		49.925
Total		17730744	726914		100.000

HPLC trace (*chiral*):

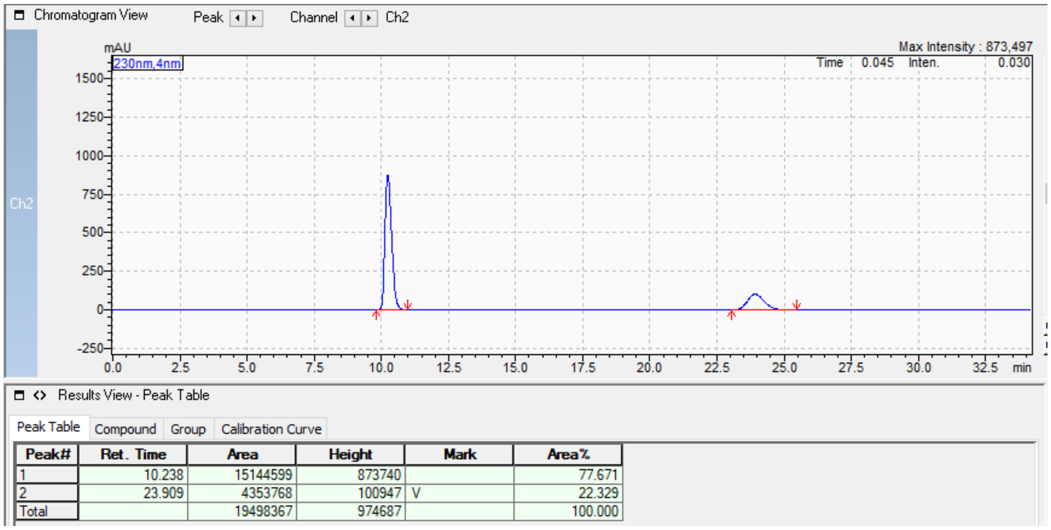

Peak#	Ret. Time	Area	Height	Mark	Area%
1	10.238	15144599	873740		77.671
2	23.909	4353768	100947	V	22.329
Total		19498367	974687		100.000

%*ee* = 77.671 – 22.329 = 55.342 = 55%

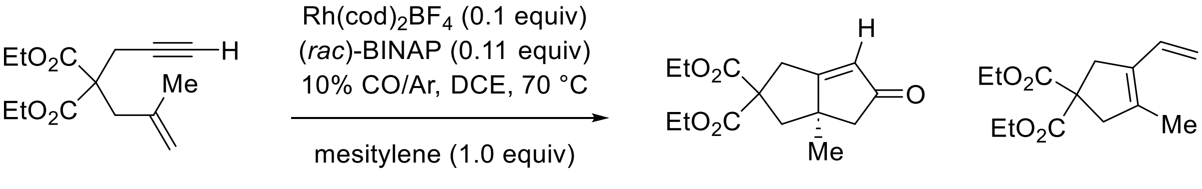

8c **9c** **9cs**

Follows General Procedure **D** for asymmetric PKR: $Rh(cod)_2BF_4$ (6.1 mg, 0.015 mmol, 0.1 equiv), (*rac*)-BINAP (10.3 mg, 0.0165 mmol, 0.11 equiv), mesitylene (18 mg, 0.15 mmol, 1.0 equiv) and enyne **8c** (0.5 mL from a stock solution of 0.3 M) in DCE (3.0 mL, 0.05 M). The reaction flask was lowered into the preheated oil bath (70 °C). After 6 h, the PKR yield (0%), b.r.s.m. (0%), starting material remaining (0%) and side product **9cs** (95%) were determined based on the integral comparison of the starting material peak (m, 4.92–4.91 ppm, 1 H) and the side product peak (m, 5.07–5.02 ppm, 2 H) to the internal standard mesitylene peak (s, 6.78 ppm, 3 H) via ^{1}H NMR. The side product **9cs** was purified by SiO_2 chromatography eluting with 5% ethyl acetate/hexanes to give the title compound as colorless oil, the resonances match that previously reported for compound **9cs**.[25]

<u>^{1}H NMR ($CDCl_3$, 400 MHz)</u>

δ 6.56 (dd, *J* = 16.9, 11.2 Hz, 1 H), 5.07–5.02 (m, 2 H), 4.19 (q, *J* = 7.1 Hz, 4 H), 3.13 (s, 2 H), 3.05 (s, 2 H), 1.75 (s, 3 H), 1.25 (t, *J* = 7.1 Hz, 6 H)

<u>TLC</u>

R_f = 0.46 (25% ethyl acetate/ hexanes); silica gel, UV

HRMS

HRMS-ESI (m/z): $[M + H]^+$ calcd for $C_{14}H_{21}O_4$, 253.1434; found: 253.1427

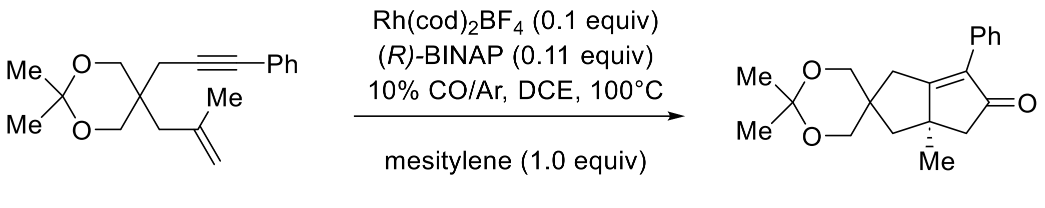

(*R*)-2’,2’,3a-trimethyl-6-phenyl-3a, 4-dihydro-1*H*-spiro[pentalene-2,5’-[1,3]dioxan]-5(3*H*)-one (11a)

Follows General Procedure **D** for asymmetric PKR: $Rh(cod)_2BF_4$ (6.1 mg, 0.015 mmol, 0.1 equiv), (*R*)-BINAP (10.3 mg, 0.0165 mmol, 0.11 equiv), mesitylene (18 mg, 0.15 mmol, 1.0 equiv) and enyne **10a** (0.5 mL from a stock solution of 0.3 M) in DCE (3.0 mL, 0.05 M). The reaction flask was lowered into the preheated oil bath (100 °C). After 88 h, the PKR yield (81%), b.r.s.m. (81%) and starting material remaining (trace) were determined based on the integral comparison of the product peak (d, 3.03 ppm, 1 H) and starting material peak (m, 4.96–4.96 ppm, 1 H) to the internal standard mesitylene peak (s, 6.78 ppm, 3 H) via ^{1}H NMR. The crude was purified by SiO_2 chromatography eluting with 15–20% ethyl acetate/hexanes to give the title compound **11a** as white solid. HPLC analysis was performed using Chiralpak IH-3 column eluting with 10% IPA/hexanes at a flow rate of 1.0 mL/min and detecting at 254 nm to give 43% *ee*.

^{1}H NMR ($CDCl_3$, 400 MHz)

δ 7.51–7.49 (m, 2 H), 7.43–7.39 (m, 2 H), 7.35–7.32 (m, 1 H), 3.95 (d, *J* = 11.3 Hz, 1 H), 3.90 (dd, *J* = 11.3, 1.5 Hz, 1 H), 3.51 (d, *J* = 11.3 Hz, 1 H), 3.28 (dd, *J* = 11.3, 1.5 Hz, 1 H), 3.03 (d, *J* = 17.4 Hz, 1 H), 2.86 (d, *J* = 17.4 Hz, 1 H), 2.58 (d, *J* = 17.4 Hz, 1H), 2.48 (d, *J* = 17.4 Hz, 1H), 1.84 (d, *J* = 13.7 Hz, 1H 1.44 (s, 3H), 1.41 (s, 3 H), 1.30 (s, 3 H), 1.28 (d, *J* = 13.7 Hz, 1H)

^{13}C NMR ($CDCl_3$, 100.6 MHz)

δ 207.5, 184.2, 134.5, 131.2, 128.9 (2C),128.5 (2C), 128.2, 98.0, 70.9, 69.4, 53.0, 47.0, 43.9, 43.5, 35.4, 28.9, 26.4, 21.3

HRMS

HRMS-ESI (m/z): $[M + H]^+$ calcd for $C_{20}H_{25}O_3$, 313.1798; found: 313.1800

IR

1698, 1107 cm^{-1}

m.p

130–135 °C

TLC

R_f = 0.2 (25% ethyl acetate/ hexanes); silica gel, UV

$[\alpha]^{20}_D$ = -35.2° (c.= 0.5, $CHCl_3$)

HPLC trace (*racemic*):

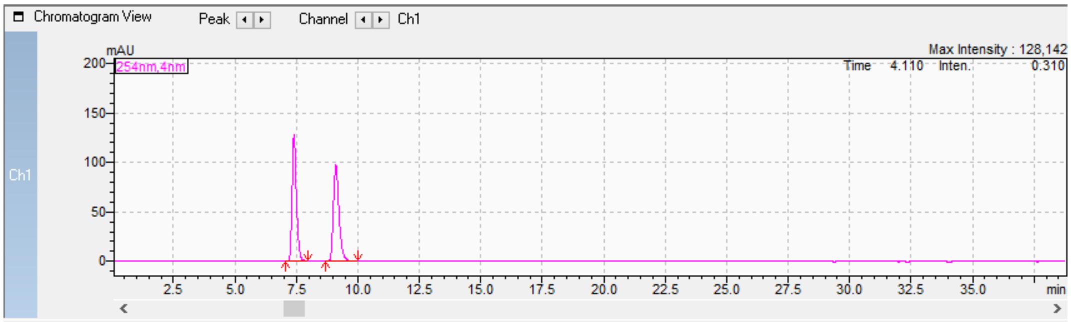

Results View - Peak Table

Peak Table | Compound | Group | Calibration Curve

Peak#	Ret. Time	Area	Height	Mark	Area%
1	7.404	1561744	128113		49.934
2	9.107	1565862	97396		50.066
Total		3127605	225509		100.000

HPLC trace (*chiral*):

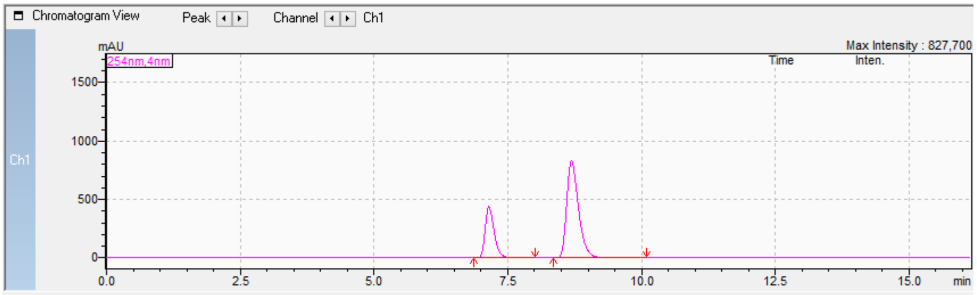

Results View - Peak Table

Peak Table | Compound | Group | Calibration Curve

Peak#	Ret. Time	Area	Height	Mark	Area%
1	7.149	5135301	439400	V	28.627
2	8.688	12803345	827523		71.373
Total		17938646	1266923		100.000

%*ee* = 71.373 – 28.627 = 42.746 = 43%

Chiral Bisphosphine Ligand Study

1,6-enyne with NTs tether (training set):

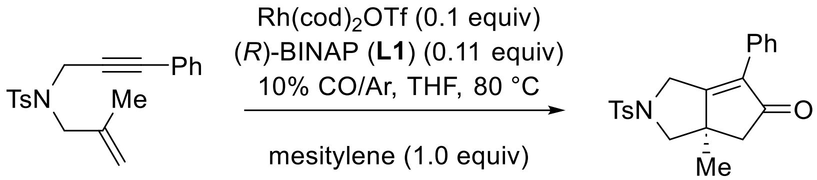

4a **5a**

Follows General Procedure D: Rh(cod)$_2$OTf (7.1 mg, 0.015 mmol, 0.1 equiv), (*R*)-BINAP (**L1**) (10.3 mg, 0.0165 mmol, 0.11 equiv), mesitylene (18.0 mg, 0.15 mmol, 1.0 equiv) and enyne **4a** (0.5 mL from a stock solution of 0.3 M) in THF (3.0 mL, 0.05 M). The reaction flask was lowered into the preheated oil bath (80 °C). After 22 h, the PKR yield (99%), b.r.s.m. (99%) and starting material remaining (0%) were determined based on the integral comparison of the product peak (d, 4.62 ppm, 1H) and starting material peak (s, 4.25 ppm, 2 H) to the internal standard mesitylene peak (s, 6.78 ppm, 3 H) via ^{1}H NMR. The crude was purified by SiO$_2$ chromatography eluting with 30% ethyl acetate/hexanes for HPLC analysis to get an *er* of 95:5.

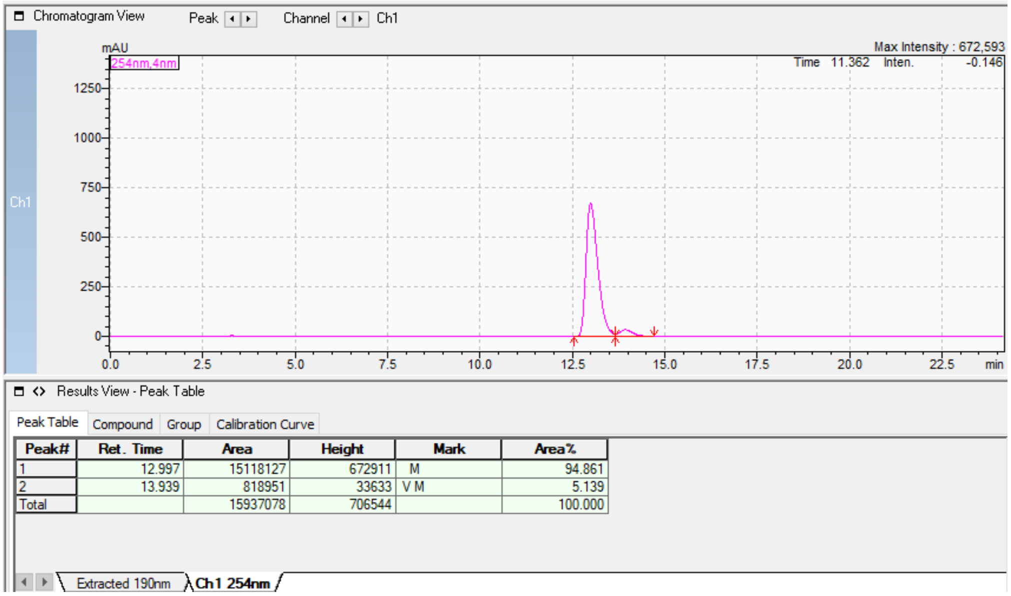

Peak#	Ret. Time	Area	Height	Mark	Area%
1	12.997	15118127	672911	M	94.861
2	13.939	818951	33633	V M	5.139
Total		15937078	706544		100.000

%*ee* = 94.861 – 5.139 = 89.722% = 90%

*An additional experiment was performed as above with aliquots taken at 6 h and 21 h for HPLC analyses (see HPLC traces below and Table **S1**).

HPLC trace at 6 h

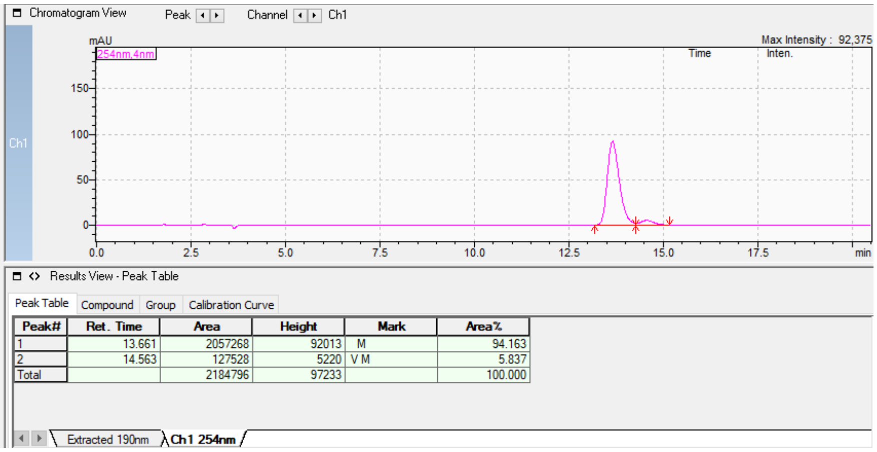

Peak#	Ret. Time	Area	Height	Mark	Area%
1	13.661	2057268	92013	M	94.163
2	14.563	127528	5220	V M	5.837
Total		2184796	97233		100.000

HPLC trace at 21 h

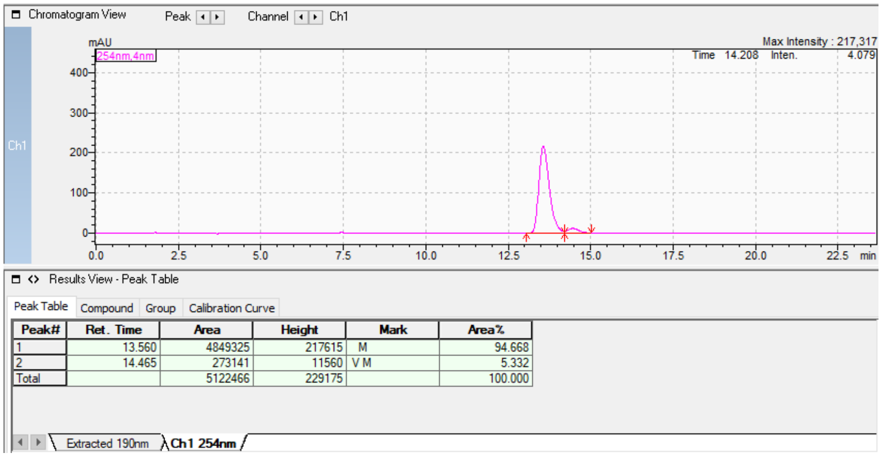

Peak#	Ret. Time	Area	Height	Mark	Area%
1	13.560	4849325	217615	M	94.668
2	14.465	273141	11560	V M	5.332
Total		5122466	229175		100.000

Table S1. Monitoring PKR product **5a** by HPLC for enantioselectivity determination over the course of the reaction

	SM **4a** remaining	yield% **5a**	*er* **5a**
6 h	30	68	94:6
21 h	2	98	95:5

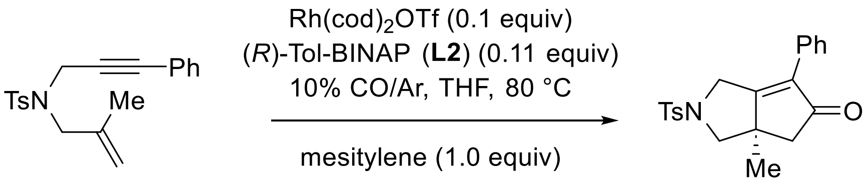

4a **5a**

Follows general procedure D. $Rh(cod)_2OTf$ (7.1 mg, 0.015 mmol, 0.1 equiv), (*R*)-Tol-BINAP (**L2**) (11.2 mg, 0.0165 mmol, 0.11 equiv), mesitylene (18.0 mg, 0.15 mmol, 1.0 equiv) and enyne **4a** (0.5 mL from a stock solution of 0.3 M) in THF (3.0 mL, 0.05 M). After 21 h, the PKR yield (96%), b.r.s.m. (96%) and starting material remaining (trace) were determined based on the integral comparison of the product peak (d, 4.62 ppm, 1H) and starting material peak (s, 4.25 ppm, 2 H) to the internal standard mesitylene peak (s, 6.78 ppm, 3 H) via ^{1}H NMR. The crude residue was purified by SiO_2 chromatography eluting with 30% ethyl acetate/hexanes. HPLC analysis was performed on chromatographed product and showed an *er* of 91:9.

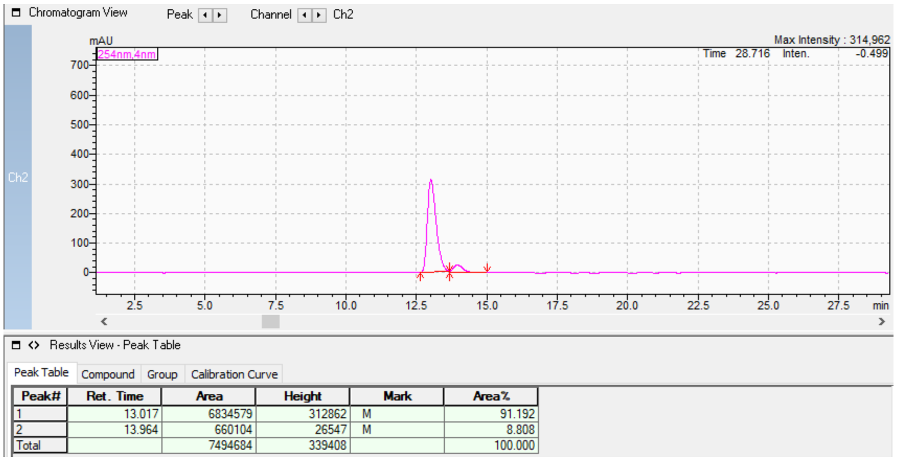

Peak#	Ret. Time	Area	Height	Mark	Area%
1	13.017	6834579	312862	M	91.192
2	13.964	660104	26547	M	8.808
Total		7494684	339408		100.000

%*ee* = 91.192-8.808= 82.384 = 82%

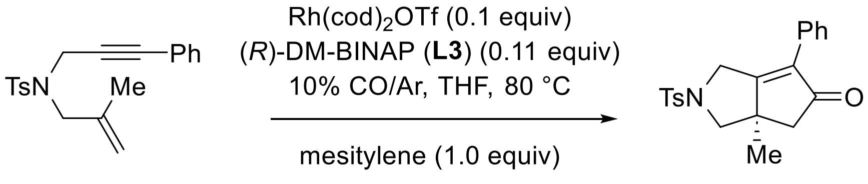

4a **5a**

Follows general procedure D. $Rh(cod)_2OTf$ (7.1 mg, 0.015 mmol, 0.1 equiv), (*R*)-DM-BINAP (**L3**) (12.1 mg, 0.0165 mmol, 0.11 equiv), mesitylene (18.0 mg, 0.15 mmol, 1.0 equiv) and enyne **4a** (0.5 mL from a stock solution of 0.3 M) in THF (3.0 mL, 0.05 M). After 19 h, the PKR yield (96%), b.r.s.m. (96%) and starting material remaining (0%) were determined based on the integral comparison of the product peak (d, 4.62 ppm, 1H) and starting material peak (s, 4.25 ppm, 2 H) to the internal standard mesitylene peak (s, 6.78 ppm, 3 H) via ^{1}H NMR. The crude was purified by SiO_2 chromatography eluting with 30% ethyl acetate/hexanes. HPLC analysis was performed on chromatographed product and showed an *er* of 89:11.

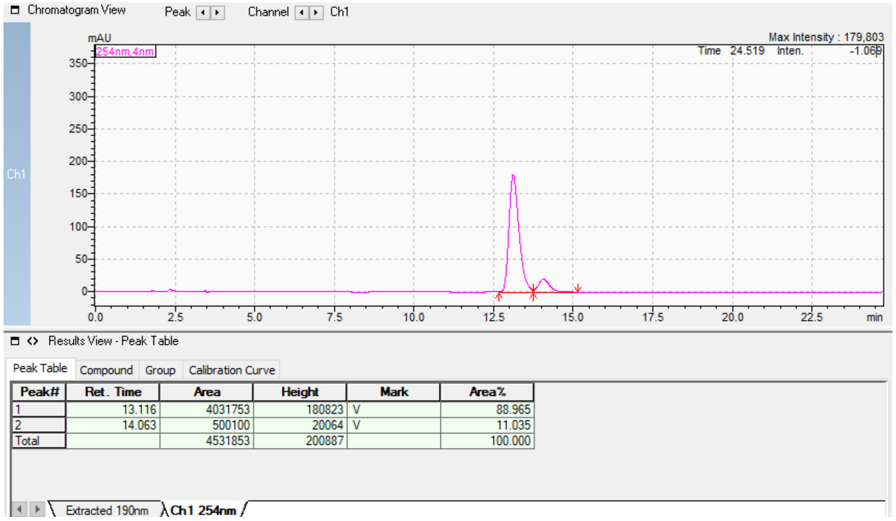

Peak#	Ret. Time	Area	Height	Mark	Area%
1	13.116	4031753	180823	V	88.965
2	14.063	500100	20064	V	11.035
Total		4531853	200887		100.000

%*ee* =88.965-11.035=77.93= 78%

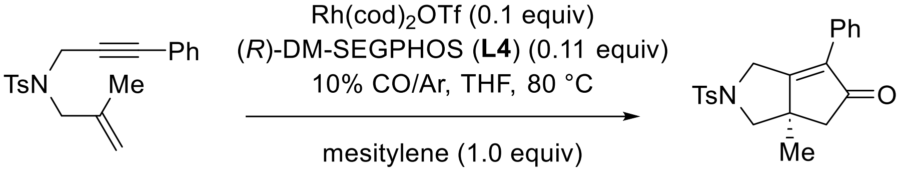

4a **5a**

Follows general procedure D. Rh(cod)$_2$OTf (7.1 mg, 0.015 mmol, 0.1 equiv), (*R*)-DM-SEGPHOS (**L4**) (11.9 mg, 0.0165 mmol, 0.11 equiv), mesitylene (18.0 mg, 0.15 mmol, 1.0 equiv) and enyne **4a** (0.5 mL from a stock solution of 0.3 M) in THF (3.0 mL, 0.05 M). After 27 h, the PKR yield (98%), b.r.s.m. (98%) and starting material remaining (0%) were determined based on the integral comparison of the product peak (d, 4.62 ppm, 1H) and starting material peak (s, 4.25 ppm, 2 H) to the internal standard mesitylene peak (s, 6.78 ppm, 3 H) via ^{1}H NMR. The crude was purified by SiO$_2$ chromatography eluting with 30% ethyl acetate/hexanes. HPLC analysis was performed on chromatographed product and showed an *er* of 85:15.

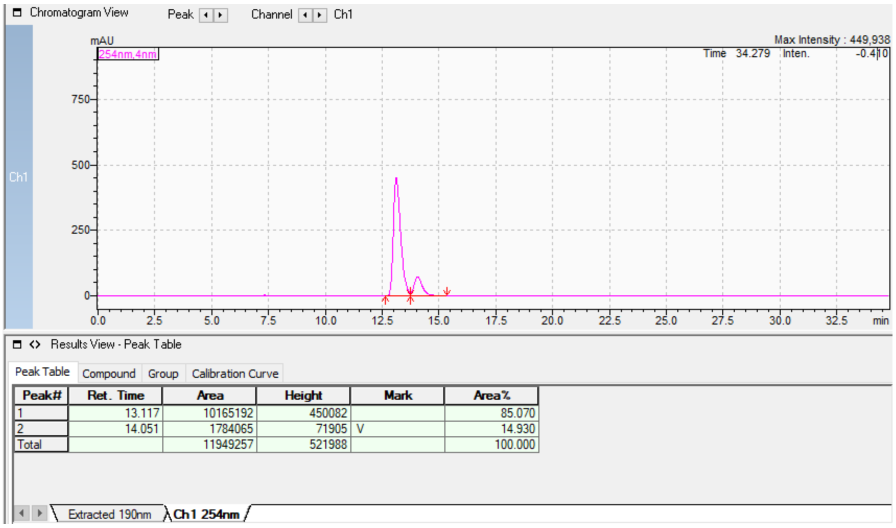

Peak#	Ret. Time	Area	Height	Mark	Area%
1	13.117	10165192	450082		85.070
2	14.051	1784065	71905	V	14.930
Total		11949257	521988		100.000

%*ee* =85.070-14.930=70.14= 70%

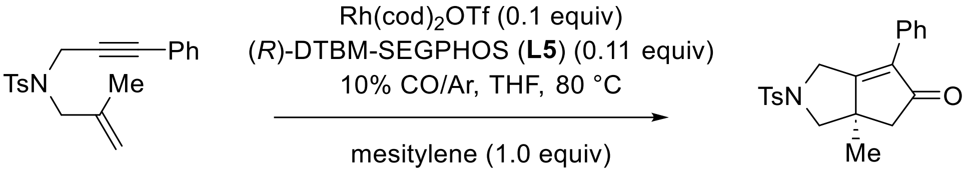

4a **5a**

Follows general procedure D. Rh(cod)$_2$OTf (7.1 mg, 0.015 mmol, 0.1 equiv), (*R*)-DTBM-SEGPHOS (**L5**) (19.5 mg, 0.0165 mmol, 0.11 equiv), mesitylene (18.0 mg, 0.15 mmol, 1.0 equiv) and enyne **4a** (0.5 mL from a stock solution of 0.3 M) in THF (3.0 mL, 0.05 M). After 40 h, the PKR yield (39%), b.r.s.m. (89%) and starting material remaining (56%) were determined based on the integral comparison of the product peak (d, 4.62 ppm, 1H) and starting material peak (s, 4.25 ppm, 2 H) to the internal standard mesitylene peak (s, 6.78 ppm, 3 H) via ^{1}H NMR. The crude was purified by SiO$_2$ chromatography eluting with 30% ethyl acetate/hexanes. HPLC analysis was performed on chromatographed product and showed an *er* of 93:7.

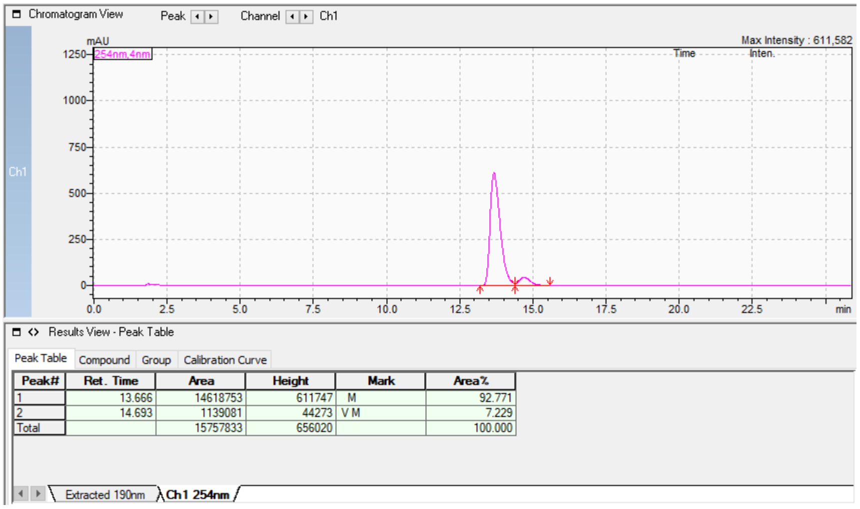

Peak#	Ret. Time	Area	Height	Mark	Area%
1	13.666	14618753	611747	M	92.771
2	14.693	1139081	44273	V M	7.229
Total		15757833	656020		100.000

%*ee* =92.771-7.229=85.542= 86%

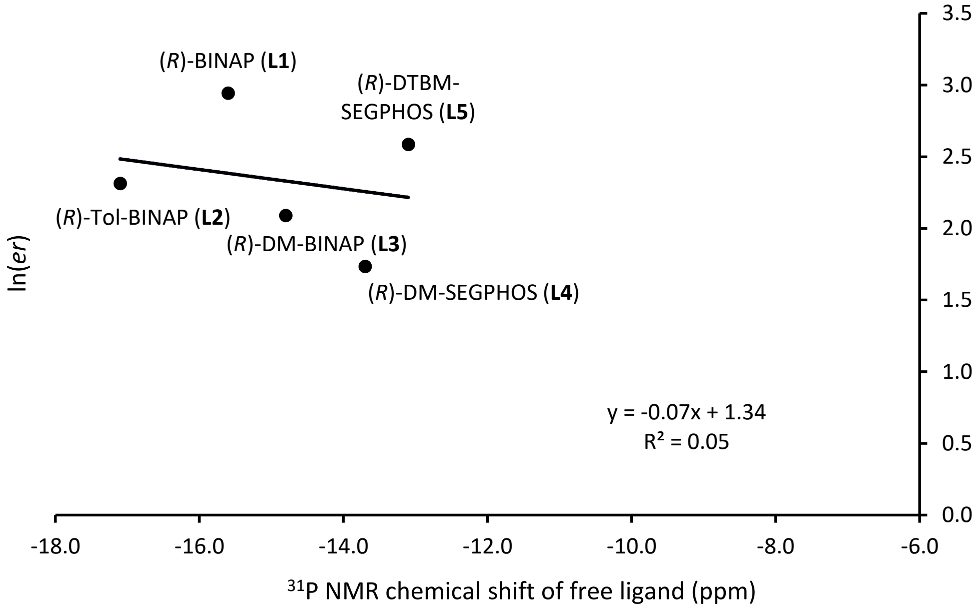

Figure S2. ln(*er*) of **5a** plotted against ^{31}P NMR chemical shift of free chiral bisphosphine ligands (^{31}P NMR were collected on Bruker Advance 400 MHz spectrometer).

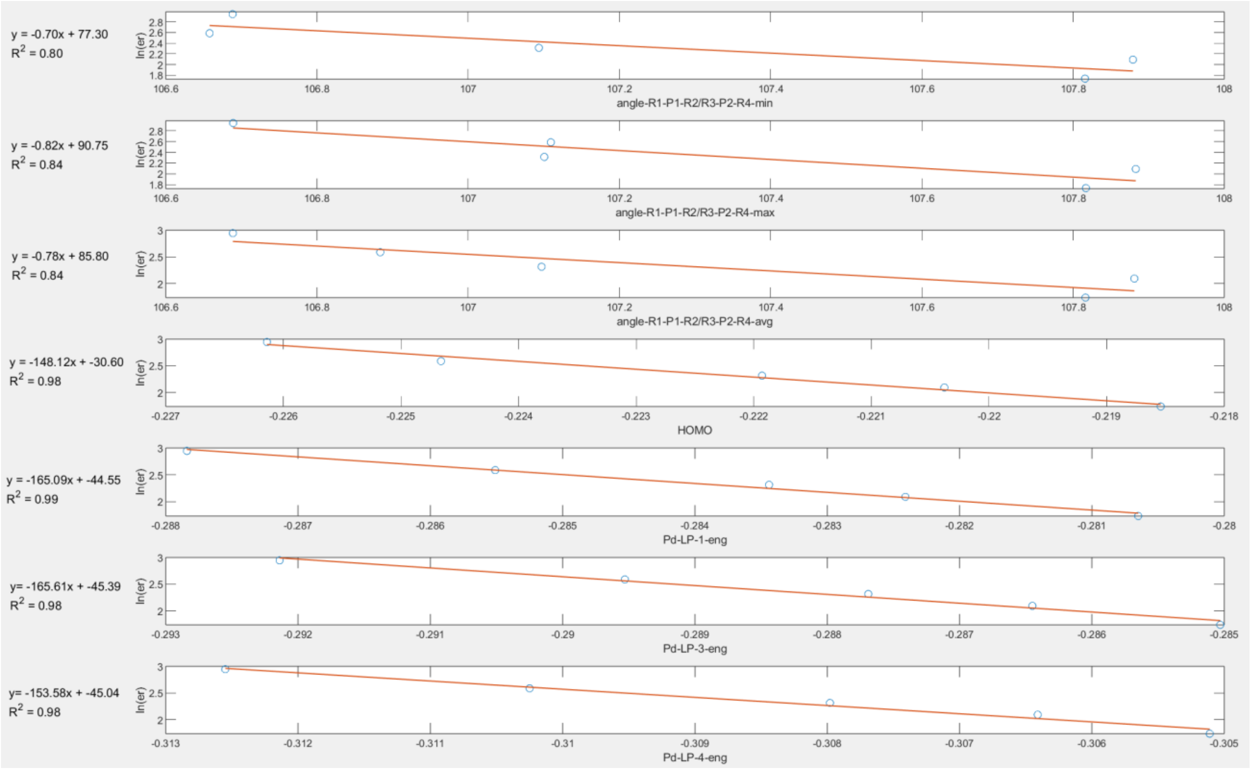

Figure S3. ln(*er*) of **5a** plotted against 7/181 computed chiral bisphosphine ligand parameters reported previsouly.[26] The other plots showed an R^2 value < 0.8 on MATLAB and were not further analyzed.

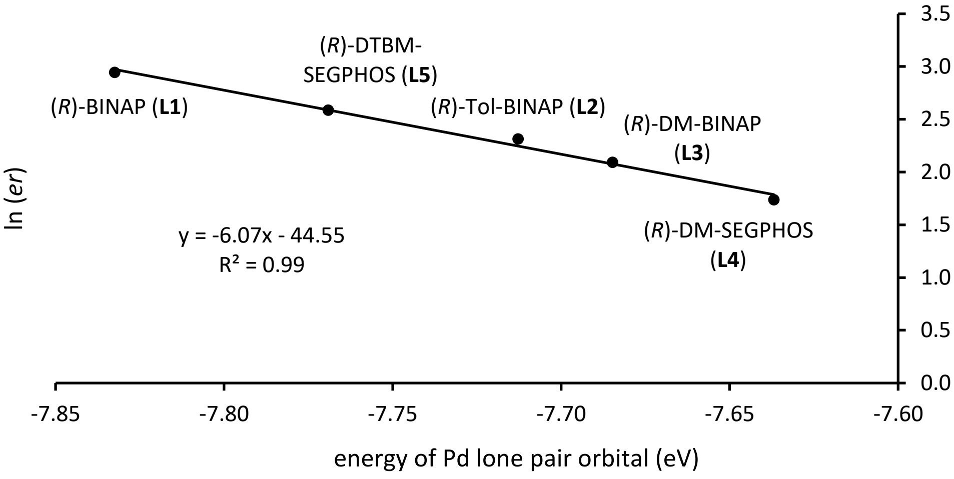

Figure S4. Plot of ln(*er*) of product **5a** relative to energy of Pd lone pair orbital (eV). *Hartree units from the ligand database reported previously were converted into electron volts (eV)[26]

1,6-enyne with NTs tether (test set):

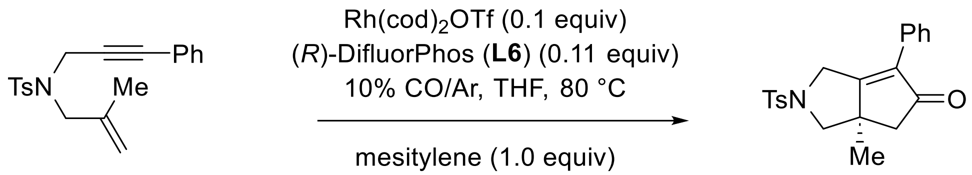

4a **5a**

Follows general procedure D. Rh(cod)$_2$OTf (7.1 mg, 0.015 mmol, 0.1 equiv), (*R*)-DifluorPhos (**L6**) (11.3 mg, 0.0165 mmol, 0.11 equiv), mesitylene (18.0 mg, 0.15 mmol, 1.0 equiv) and enyne **4a** (0.5 mL from a stock solution of 0.3 M) in THF (3.0 mL, 0.05 M). After 26 h, the PKR yield (78%), b.r.s.m. (100%) and starting material remaining (22%) were determined based on the integral comparison of the product peak (d, 4.62 ppm, 1H) and starting material peak (s, 4.25 ppm, 2 H) to the internal standard mesitylene peak (s, 6.78 ppm, 3 H) via ^{1}H NMR. The crude was purified by SiO_2 chromatography eluting with 30% ethyl acetate/hexanes. HPLC analysis was performed on chromatographed product and showed an *er* of 97:3.

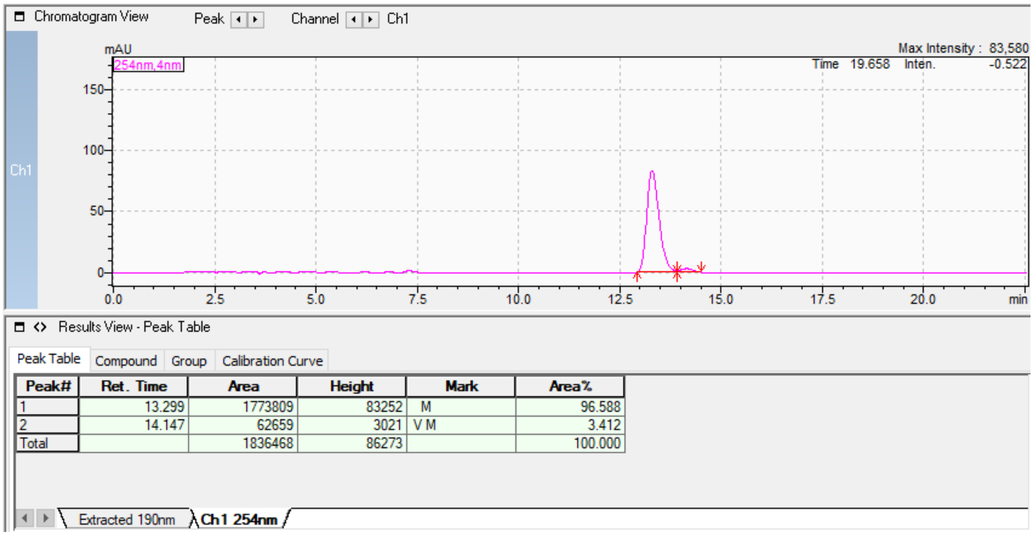

Peak#	Ret. Time	Area	Height	Mark	Area%
1	13.299	1773809	83252	M	96.588
2	14.147	62659	3021	V M	3.412
Total		1836468	86273		100.000

%*ee* = 96.588-3.412 = 93.176 = 93%

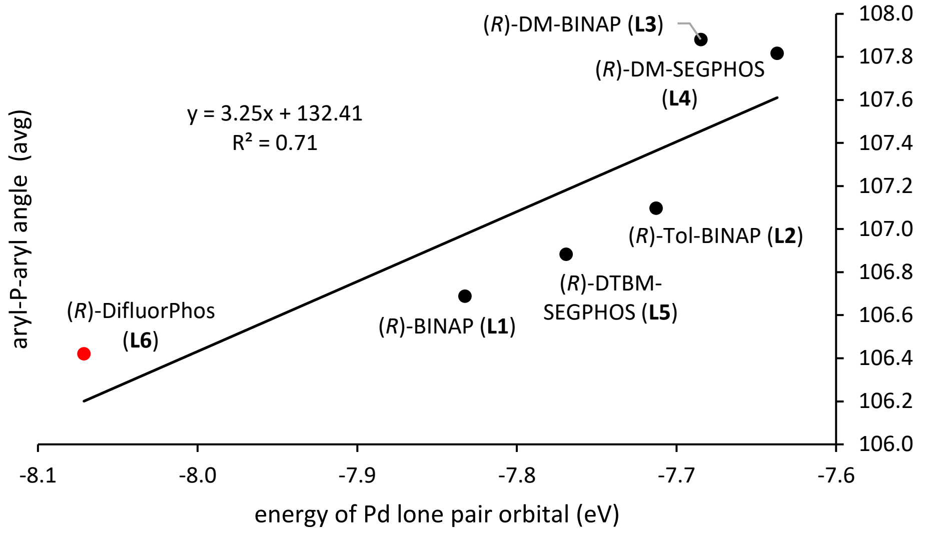

Figure S5. Correlation plot between energy of Pd lone pair orbital (eV) and aryl-P-aryl angle (avg) (angle-R1-P1-R2/R3-P2-R4-avg in Figure S3) with (*R*)-DifluorPhos (**L6**) included . *Hartree units from the ligand database reported previously were converted into electronvolts (eV)[26]

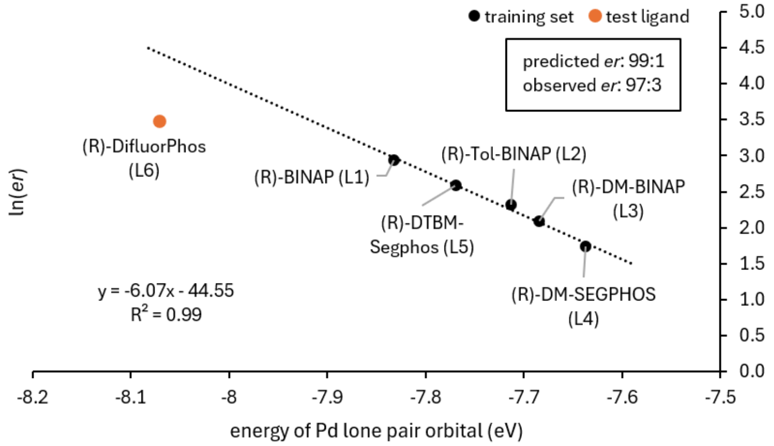

Figure S6. Plot of ln(*er*) of product **5a** relative to energy of Pd lone pair orbital (eV) with (*R*)-DifluorPhos (**L6**) included. *Hartree units from the ligand database reported previously were converted into electronvolts (eV)[26]

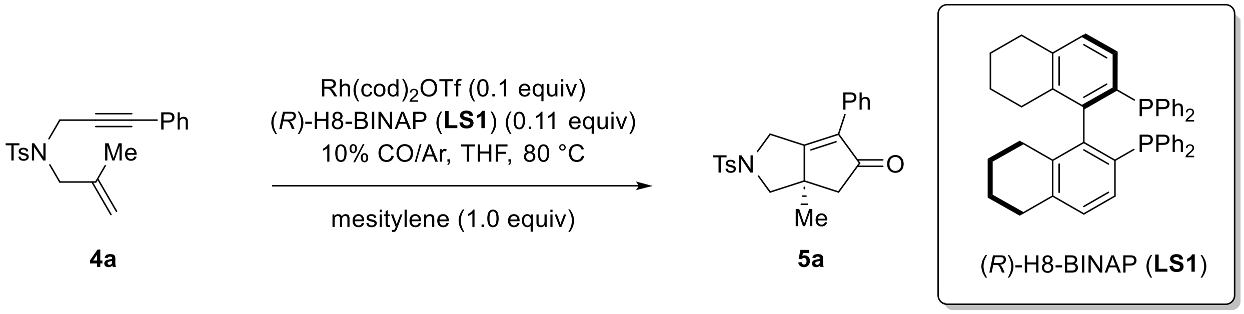

Follows general procedure D. Rh(cod)$_2$OTf (7.1 mg, 0.015 mmol, 0.1 equiv), (*R*)-H8-BINAP (**LS1**) (10.4 mg, 0.0165 mmol, 0.11 equiv), mesitylene (18.0 mg, 0.15 mmol, 1.0 equiv) and enyne **4a** (0.5 mL from a stock olution of 0.3 M) in THF (3.0 mL, 0.05 M). After 15 h, the PKR yield (98%), b.r.s.m. (98%) and starting material remaining (0%) were determined based on the integral comparison of the product peak (d, 4.62 ppm, 1H) and starting material peak (s, 4.25 ppm, 2 H) to the internal standard mesitylene peak (s, 6.78 ppm, 3 H) via ^{1}H NMR. The crude was purified by SiO$_2$ chromatography eluting with 30% ethyl acetate/hexanes. HPLC analysis was performed on chromatographed product and showed an *er* of 90:10.

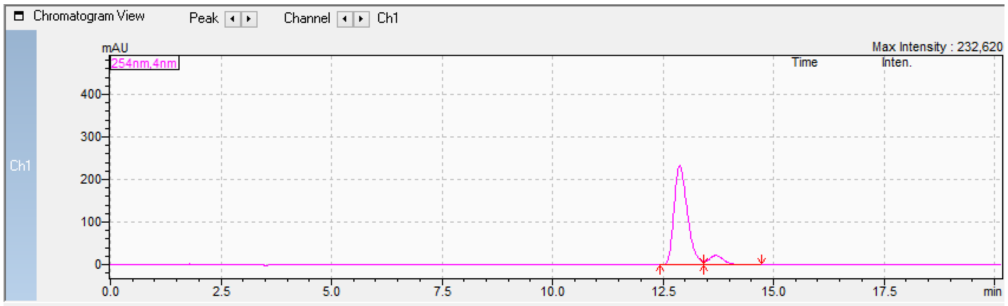

Results View - Peak Table

Peak Table | Compound | Group | Calibration Curve

Peak#	Ret. Time	Area	Height	Mark	Area%
1	12.876	4916808	233059		90.372
2	13.693	523815	22161	V	9.628
Total		5440624	255220		100.000

%*ee* = 90.372-9.628=80.744 = 81%

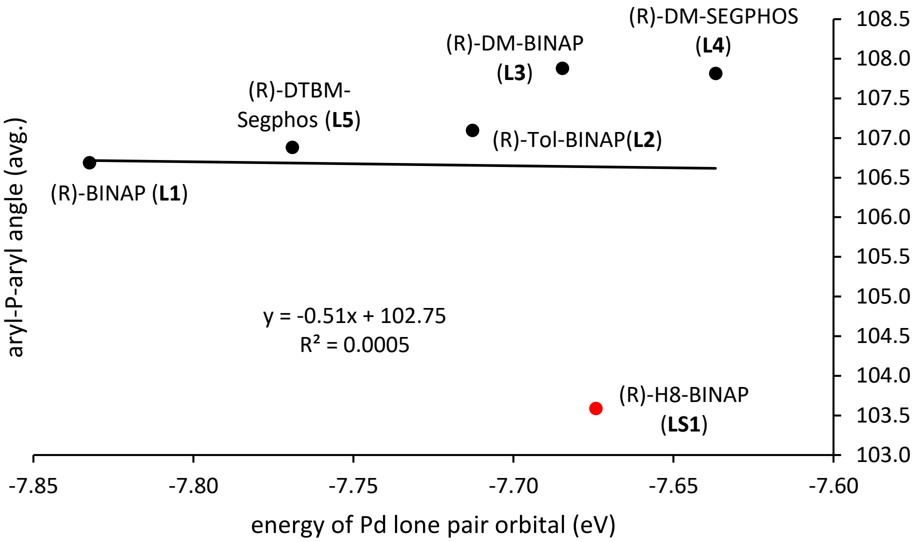

Figure S7. Correlation plot between energy of Pd lone pair orbital (eV) and aryl-P-aryl angle (avg) (angle-R1-P1-R2/R3-P2-R4-avg in Figure S3) with (*R*)-H8-BINAP (**LS1**) included . *Hartree units from the ligand database reported previsouly were converted into electronvolts (eV)[26]

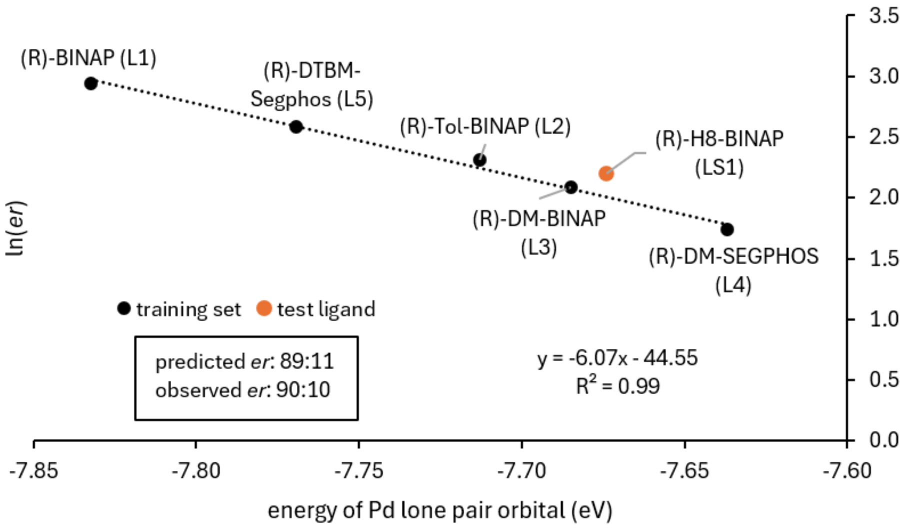

Figure S8. Plot of ln(*er*) of product **5a** relative to energy of Pd lone pair orbital (eV) with (*R*)-H8-BINAP (**LS1**) included. *Hartree units from the ligand database reported previously were converted into electronvolts (eV)[26]

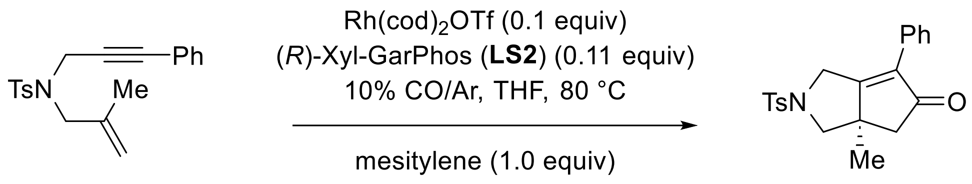

4a **5a**

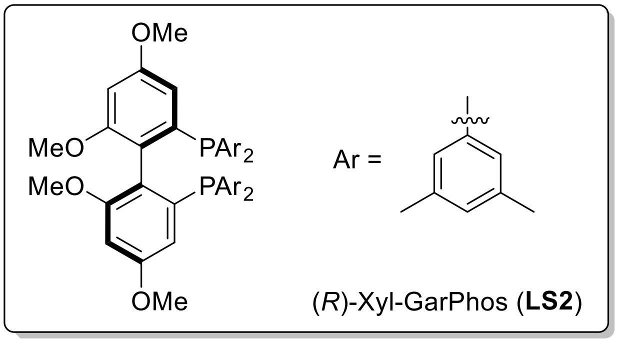

Follows general procedure D. Rh(cod)$_2$OTf (7.1 mg, 0.015 mmol, 0.1 equiv), (*R*)-Xyl-GarPhos (**LS2**) (12.5 mg, 0.0165 mmol, 0.11 equiv), mesitylene (18.0 mg, 0.15 mmol, 1.0 equiv) and enyne **4a** (0.5 mL from a stock olution of 0.3 M) in THF (3.0 mL, 0.05 M). After 15 h, the PKR yield (98%), b.r.s.m. (98%) and starting material remaining (0%) were determined based on the integral comparison of the product peak (d, 4.62 ppm, 1H) and starting material peak (s, 4.25 ppm, 2 H) to the internal standard mesitylene peak (s, 6.78 ppm, 3 H) via ^{1}H NMR. The crude was purified by SiO$_2$ chromatography eluting with 30% ethyl acetate/hexanes. HPLC analysis was performed on chromatographed product and showed an *er* of 91:9.

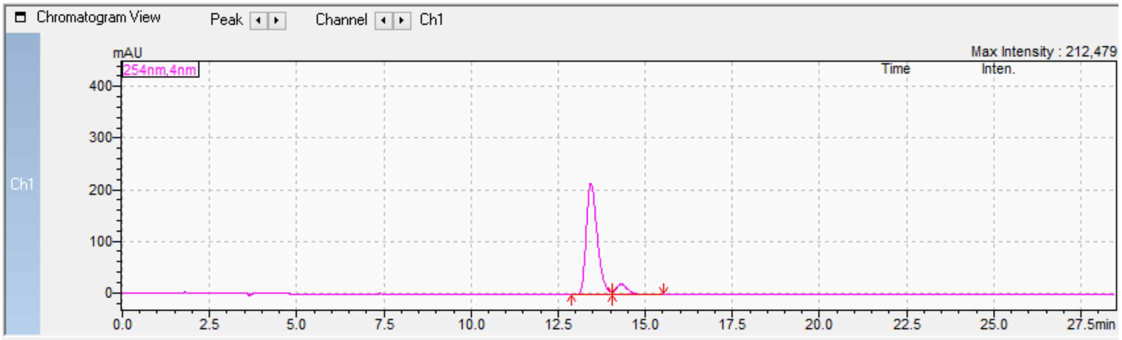

Results View - Peak Table

Peak Table | Compound | Group | Calibration Curve

Peak#	Ret. Time	Area	Height	Mark	Area%
1	13.439	4790557	216498		90.500
2	14.312	502886	20283	V	9.500
Total		5293444	236781		100.000

%*ee* = 90.5- 9.5= 81%

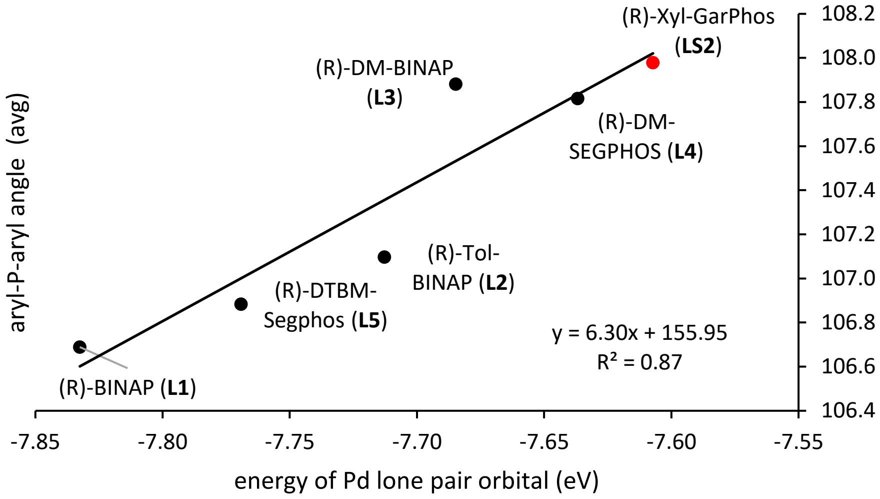

Figure S9. Correlation plot between energy of Pd lone pair orbital (eV) and aryl-P-aryl angle (avg) (angle-R1-P1-R2/R3-P2-R4-avg in Figure S3) with (*R*)-Xyl-GarPhos (**LS2**) included . *Hartree units from the ligand database reported previously were converted into electronvolts (eV)[26]

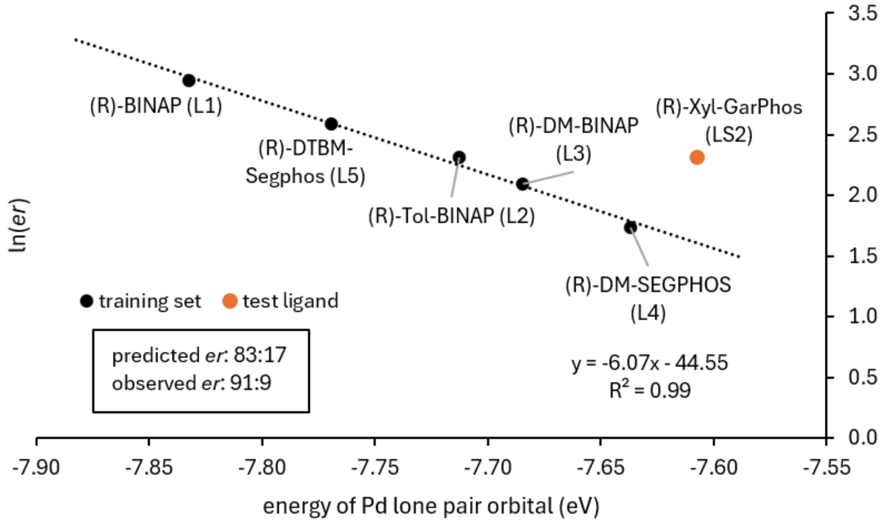

Figure S10. Plot of ln(*er*) of product **5a** relative to energy of Pd lone pair orbital (eV) with (*R*)-Xyl-GarPhos (**LS2**) included. *Hartree units from the ligand database reported previously were converted into electronvolts (eV)[26]

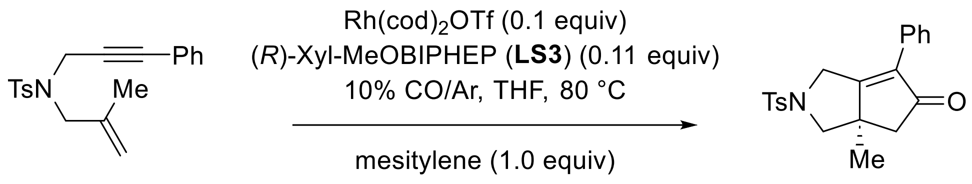

4a **5a**

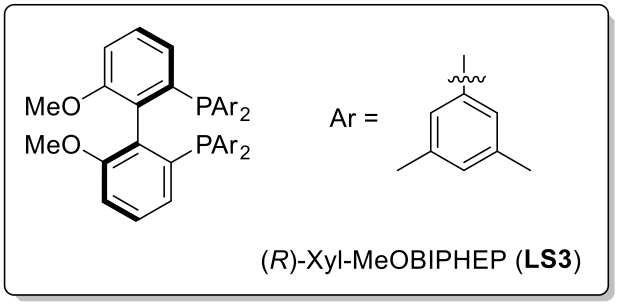

Follows general procedure D. Rh(cod)$_2$OTf (7.1 mg, 0.015 mmol, 0.1 equiv), (*R*)-Xyl-MeOBIPHEP (**LS3**) (11.5 mg, 0.0165 mmol, 0.11 equiv), mesitylene (18.0 mg, 0.15 mmol, 1.0 equiv) and enyne **4a** (0.5 mL from a stock olution of 0.3 M) in THF (3.0 mL, 0.05 M). After 23 h, the PKR yield (98%), b.r.s.m. (98%) and starting material remaining (0%) were determined based on the integral comparison of the product peak (d, 4.62 ppm, 1H) and starting material peak (s, 4.25 ppm, 2 H) to the internal standard mesitylene peak (s, 6.78 ppm, 3 H) via ^{1}H NMR. The crude was purified by SiO$_2$ chromatography eluting with 30% ethyl acetate/hexanes. HPLC analysis was performed on chromatographed product and showed an *er* of 90:10.

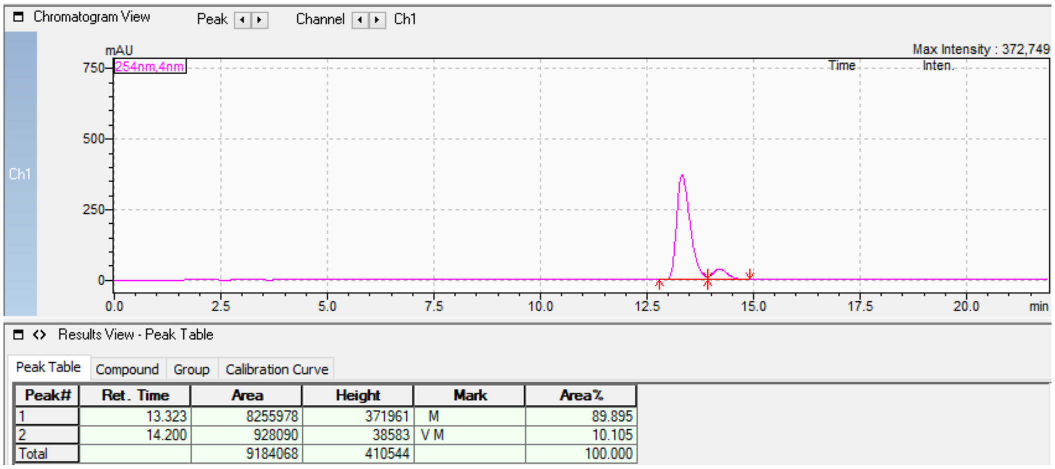

Peak#	Ret. Time	Area	Height	Mark	Area%
1	13.323	8255978	371961	M	89.895
2	14.200	928090	38583	V M	10.105
Total		9184068	410544		100.000

%*ee*= 89.895-10105= 79.79 = 80%

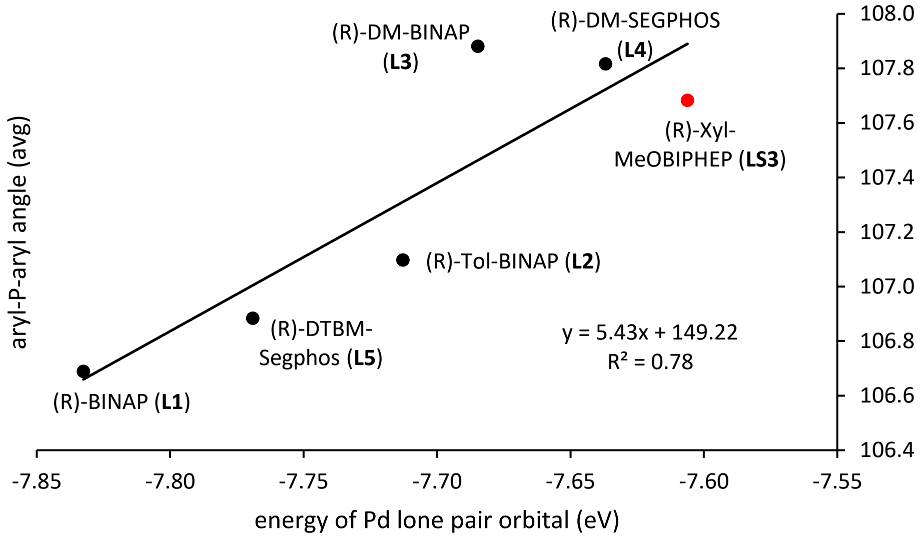

Figure S11. Correlation plot between energy of Pd lone pair orbital (eV) and aryl-P-aryl angle (avg) (angle-R1-P1-R2/R3-P2-R4-avg in Figure S3) with (*R*)-Xyl-MeOBIPHEP (**LS3**) included. *Hartree units from the ligand database reported previously were converted into electronvolts (eV)[26]

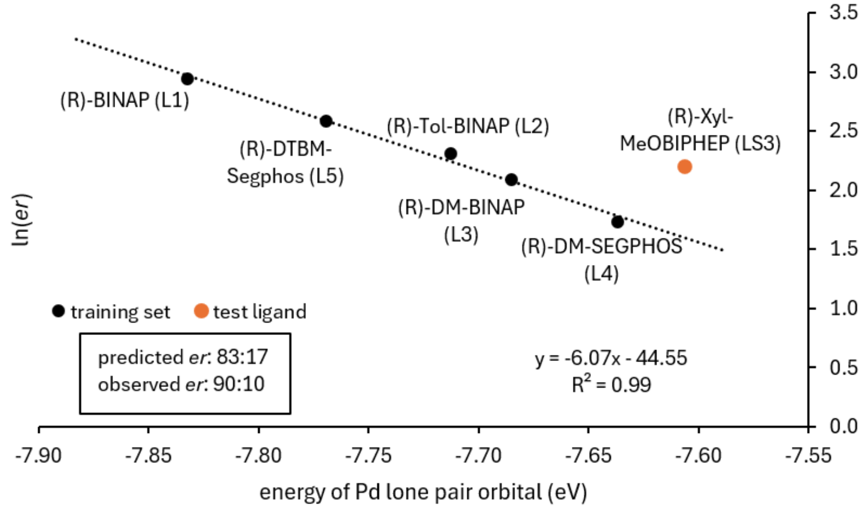

Figure S12. Plot of ln(*er*) of product **5a** relative to energy of Pd lone pair orbital (eV) with (*R*)-Xyl-MeOBIPHEP (**LS3**) included. *Hartree units from the ligand database reported previsouly were converted into electronvolts (eV)[26]

1,6-enyne with ether tether (training set):

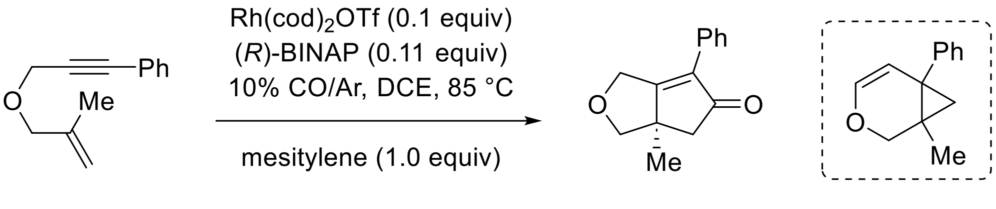

6a **7a** **7ai**

Follows General Procedure D for asymmetric PKR: Rh(cod)$_2$OTf (7.1 mg, 0.015 mmol, 0.1 equiv), (*R*)-BINAP (**L1**) (10.3 mg, 0.0165 mmol, 0.11 equiv), mesitylene (18 mg, 0.15 mmol, 1.0 equiv) and enyne **6a** (0.5 mL from a stock solution of 0.3 M) in DCE (3.0 mL, 0.05 M). The reaction flask was lowered into the preheated oil bath (85 °C). After 23 h, the PKR yield (84%), b.r.s.m. (85%), starting material remaining (1%) and cycloisomerized product (13%) were determined based on the integral comparison of the product peak (d, 4.61 ppm, 1 H), starting material peak (s, 4.36 ppm, 2 H) and cycloisomerized product peak (d, 5.21 ppm, 1 H) to the internal standard mesitylene peak (s, 6.78 ppm, 3 H) via ^{1}H NMR. The crude was purified by SiO$_2$ chromatography eluting with 20% ethyl acetate/hexanes. HPLC analysis was performed on chromatographed product and showed an *er* of 94:6.

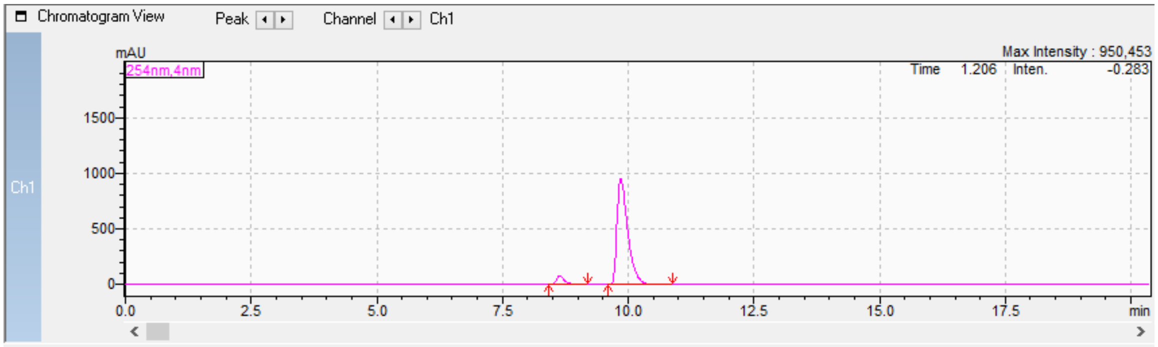

Results View - Peak Table

Peak Table | Compound | Group | Calibration Curve

Peak#	Ret. Time	Area	Height	Mark	Area%
1	8.637	832455	77352		5.572
2	9.854	14108387	950865		94.428
Total		14940842	1028217		100.000

ee% = 94.428-5.572 = 88.856 = 89%

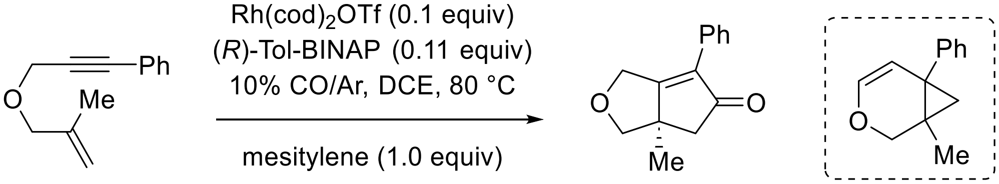

6a **7a** **7ai**

Follows general procedure D. Rh(cod)$_2$OTf (7.1 mg, 0.015 mmol, 0.1 equiv), (*R*)-Tol-BINAP (**L2**) (11.2 mg, 0.0165 mmol, 0.11 equiv), mesitylene (18 mg, 0.15 mmol, 1.0 equiv) and enyne **6a** (0.5 mL from a stock solution of 0.3 M) in DCE (3.0 mL, 0.05 M). After 29 h, the PKR yield (79%), b.r.s.m. (83%), starting material remaining (5%) and cycloisomerized product (7%) were determined based on the integral comparison of the product peak (d, 4.61 ppm, 1 H), starting material peak (s, 4.36 ppm, 2 H) and cycloisomerized product peak (d, 5.21 ppm, 1 H) to the internal standard mesitylene peak (s, 6.78 ppm, 3 H) via ^{1}H NMR. The crude was purified by prep TLC eluting with 25% ethyl acetate/hexanes. HPLC analysis was performed on chromatographed product and showed an *er* of 83:17.

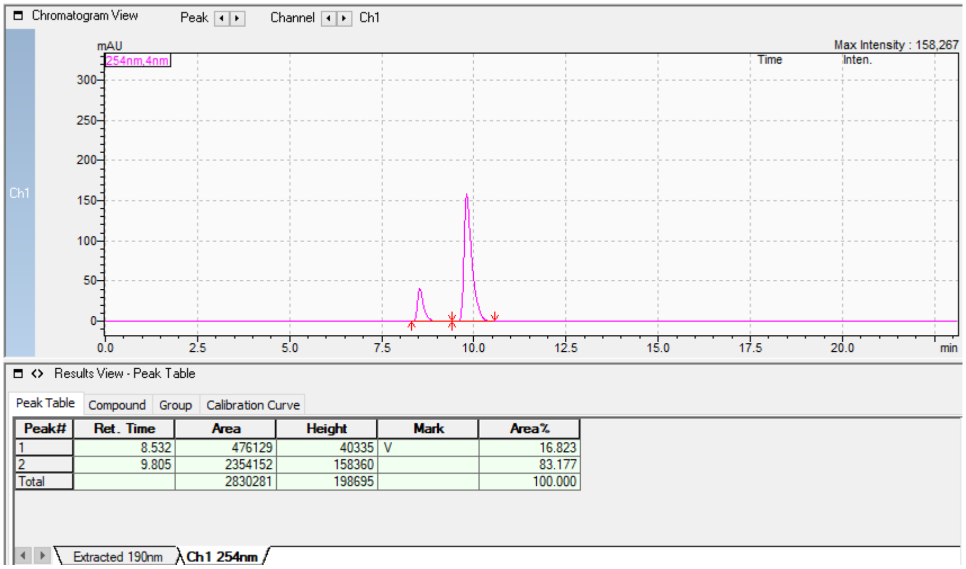

Peak#	Ret. Time	Area	Height	Mark	Area%
1	8.532	476129	40335	V	16.823
2	9.805	2354152	158360		83.177
Total		2830281	198695		100.000

%*ee* = 83.177-16.823= 66.354 = 66%

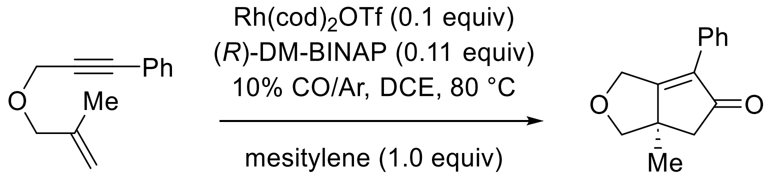

6a **7a**

Follows general procedure D. Rh(cod)$_2$OTf (7.1 mg, 0.015 mmol, 0.1 equiv), (*R*)-DM-BINAP (**L3**) (12.1 mg, 0.0165 mmol, 0.11 equiv), mesitylene (18 mg, 0.15 mmol, 1.0 equiv) and enyne **6a** (0.5 mL from a stock solution of 0.3 M) in DCE (3.0 mL, 0.05 M). After 20 h, the PKR yield (74%), b.r.s.m. (74%) and starting material remaining (0%) were determined based on the integral comparison of the product peak (d, 4.61 ppm, 1 H) and starting material peak (s, 4.36 ppm, 2 H) to the internal standard mesitylene peak (s, 6.78 ppm, 3 H) via ^{1}H NMR. The crude was purified by prep TLC eluting with 25% ethyl acetate/hexanes. HPLC analysis was performed on chromatographed product and showed an *er* of 70:30.

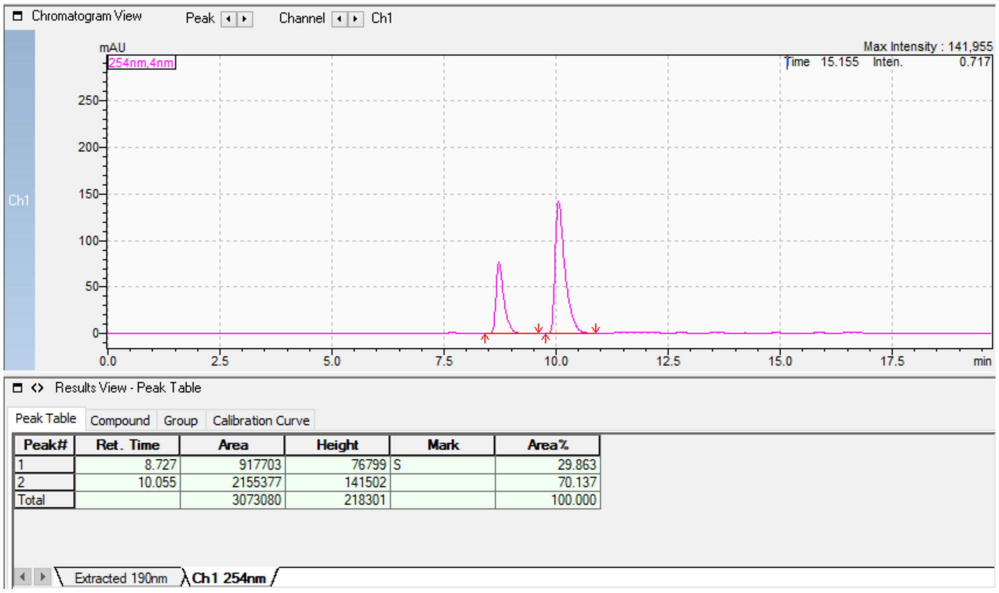

Peak#	Ret. Time	Area	Height	Mark	Area%
1	8.727	917703	76799	S	29.863
2	10.055	2155377	141502		70.137
Total		3073080	218301		100.000

%*ee* = 70.137-29.863= 40.274 = 40%

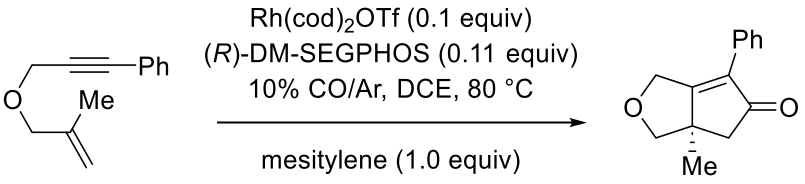

6a **7a**

Follows general procedure D. $Rh(cod)_2OTf$ (7.1 mg, 0.015 mmol, 0.1 equiv), (*R*)-DM-SEGPHOS (**L4**) (11.9 mg, 0.0165 mmol, 0.11 equiv), mesitylene (18 mg, 0.15 mmol, 1.0 equiv) and enyne **6a** (0.5 mL from a stock solution of 0.3 M) in DCE (3.0 mL, 0.05 M). After 17 h, the PKR yield (62%), b.r.s.m. (62%) and starting material remaining (0%) were determined based on the integral comparison of the product peak (d, 4.61 ppm, 1 H) and starting material peak (s, 4.36 ppm, 2 H) to the internal standard mesitylene peak (s, 6.78 ppm, 3 H) via ^{1}H NMR. The crude was purified by prep TLC eluting with 25% ethyl acetate/hexanes. HPLC analysis was performed on chromatographed product and showed an *er* of 74:26.

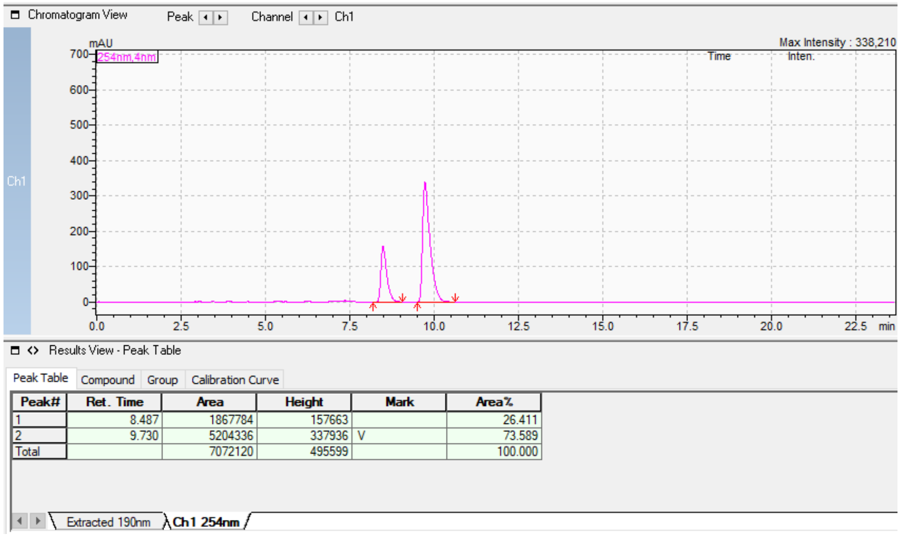

Peak#	Ret. Time	Area	Height	Mark	Area%
1	8.487	1867784	157663		26.411
2	9.730	5204336	337936	V	73.589
Total		7072120	495599		100.000

%*ee* = 73.589-26.411= 47.178 = 47%

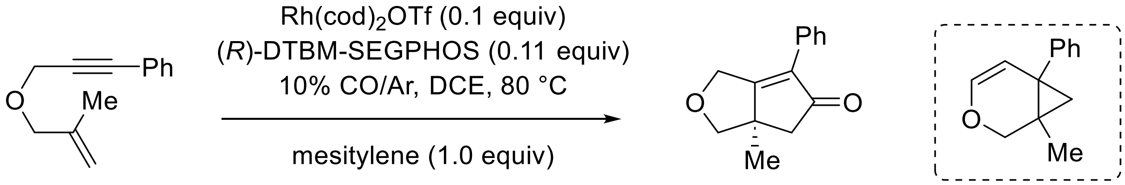

6a **7a** **7ai**

Follows general procedure D. Rh(cod)$_2$OTf (7.1 mg, 0.015 mmol, 0.1 equiv), (*R*)-DTBM-SEGPHOS (**L5**) (19.5 mg, 0.0165 mmol, 0.11 equiv), mesitylene (18 mg, 0.15 mmol, 1.0 equiv) and enyne **6a** (0.5 mL from a stock solution of 0.3 M) in DCE (3.0 mL, 0.05 M). After 44 h, the PKR yield (69%), b.r.s.m. (91%), starting material remaining (24%) and cycloisomerized product (3%) were determined based on the integral comparison of the product peak (d, 4.61 ppm, 1 H), starting material peak (s, 4.36 ppm, 2 H) and cycloisomerized product peak (d, 5.21 ppm, 1 H) to the internal standard mesitylene peak (s, 6.78 ppm, 3 H) via ^{1}H NMR. The crude was purified by prep TLC eluting with 25% ethyl acetate/hexanes. HPLC analysis was performed on chromatographed product and showed an *er* of 89:11.

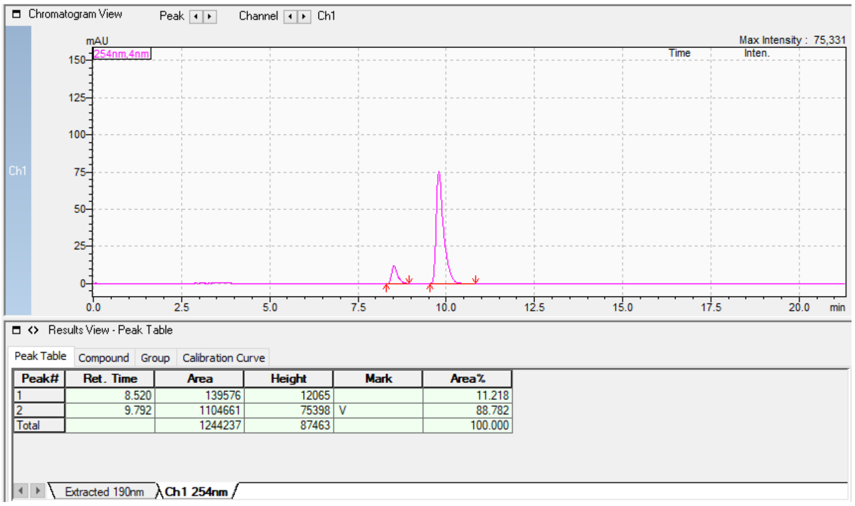

Peak#	Ret. Time	Area	Height	Mark	Area%
1	8.520	139576	12065		11.218
2	9.792	1104661	75398	V	88.782
Total		1244237	87463		100.000

%*ee* = 88.782-11.218= 77.564 = 78%

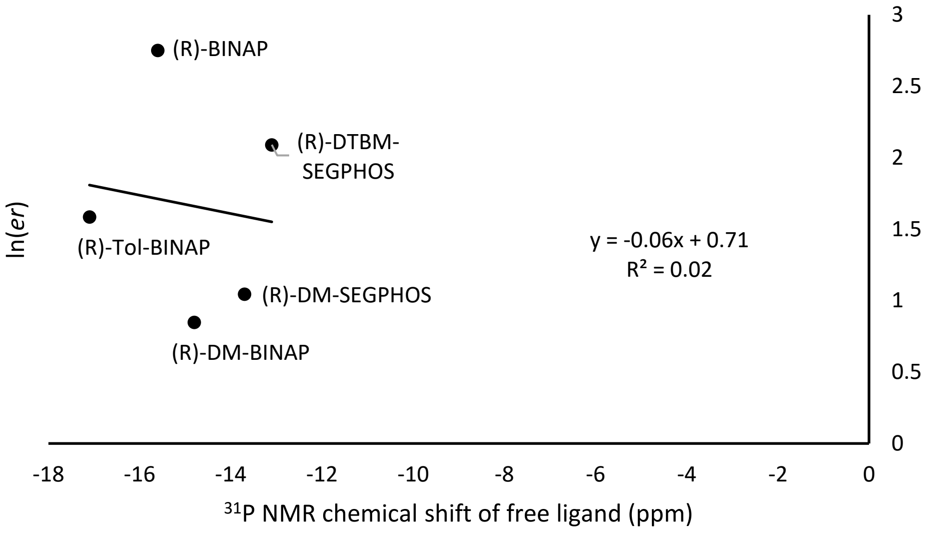

Figure S13. ln(*er*) of product **7a** plotted against ^{31}P NMR chemical shift of free chiral bisphosphine ligands. (^{31}P NMR were collected on Bruker Advance 400 MHz spectrometer).

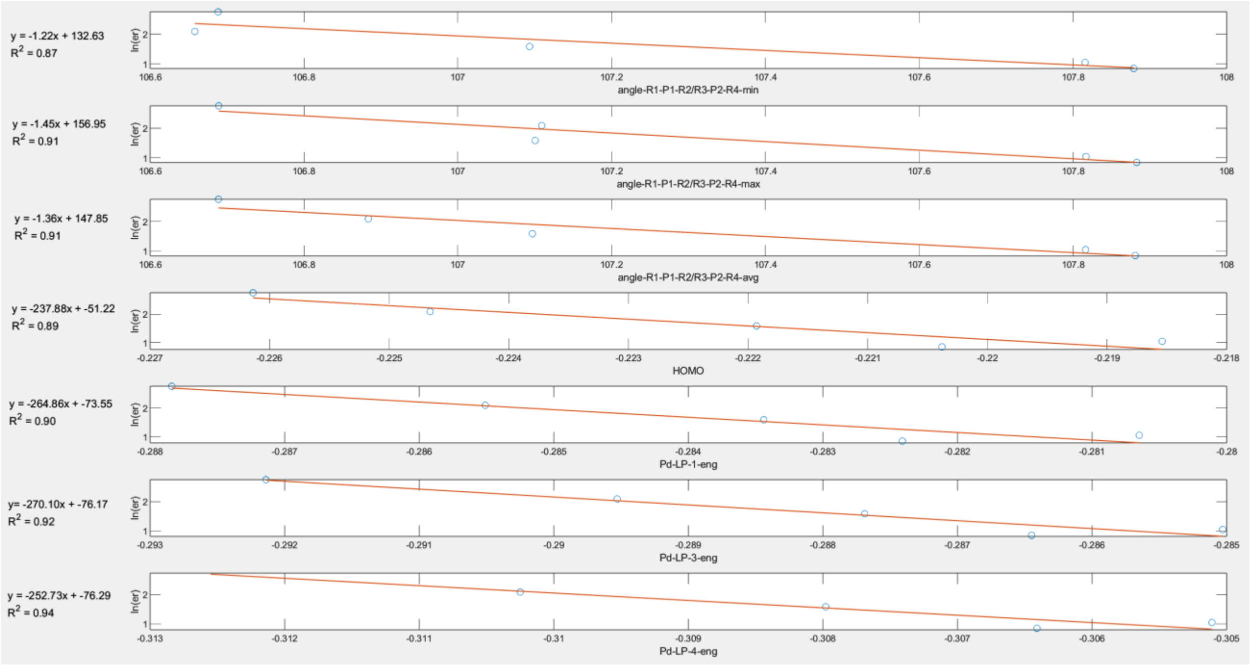

Figure S14. ln(*er*) of product **7a** plotted against 7/181 computed chiral bisphosphine ligand parameters reported previsouly.[26] The other plots showed an R^2 value < 0.8 on MATLAB and were not further analyzed.

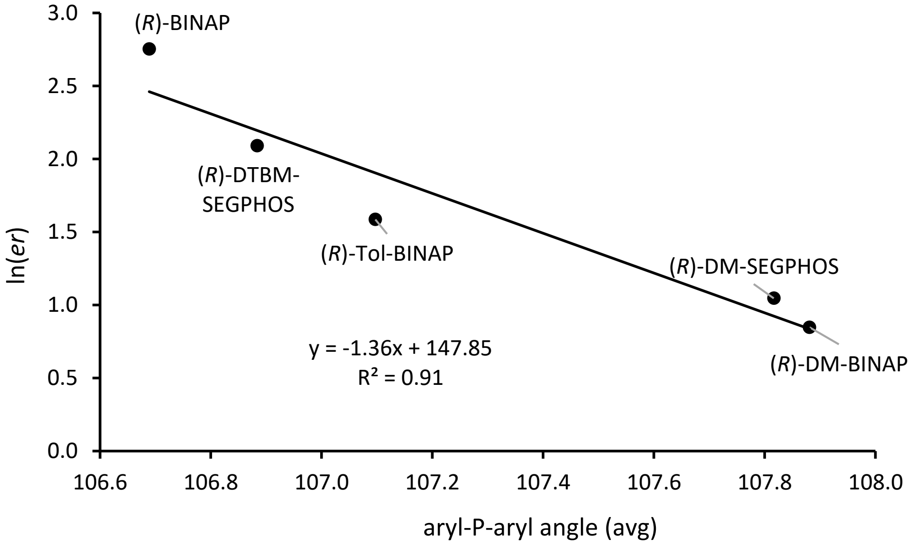

Figure S15. Plot of ln(*er*) of product **7a** relative to aryl-P-aryl angle (angle-R1-P1-R2/R3-P2-R4-avg in Figure S14).

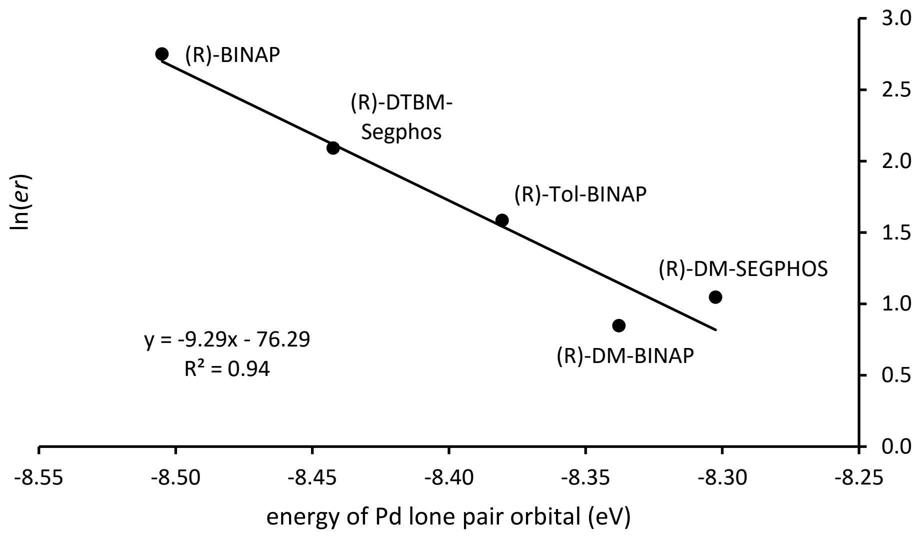

Figure S16. Plot of ln(*er*) of product **7a** relative to energy of Pd lone pair orbital (eV). *Hartree units from the ligand database reported previously were converted into electronvolts (eV)[26]

1,6-enyne with ether tether (test set):

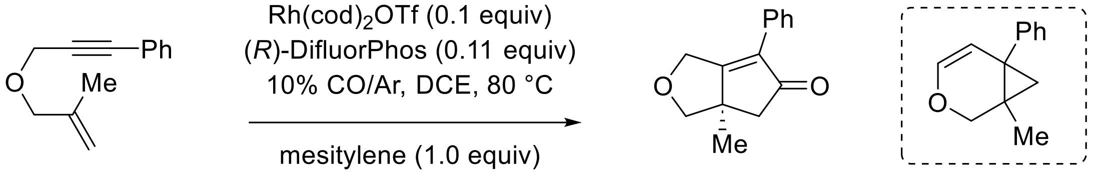

6a **7a** **7ai**

Follows general procedure D. Rh(cod)$_2$OTf (7.1 mg, 0.015 mmol, 0.1 equiv), (*R*)-DifluorPhos (**L6**) (11.3 mg, 0.0165 mmol, 0.11 equiv), mesitylene (18 mg, 0.15 mmol, 1.0 equiv) and enyne **6a** (0.5 mL from a stock solution of 0.3 M) in DCE (3.0 mL, 0.05 M). After 15 h, the PKR yield (47%), b.r.s.m. (57%), starting material remaining (23%) and cycloisomerized product (17%) were determined based on the integral comparison of the product peak (d, 4.61 ppm, 1 H), starting material peak (s, 4.36 ppm, 2 H) and cycloisomerized product peak (d, 5.21 ppm, 1 H) to the internal standard mesitylene peak (s, 6.78 ppm, 3 H) via ^{1}H NMR. The crude was purified by prep TLC eluting with 25% ethyl acetate/hexanes. HPLC analysis was performed on chromatographed product and showed an *er* of 93:7.

HPLC trace at 3 h

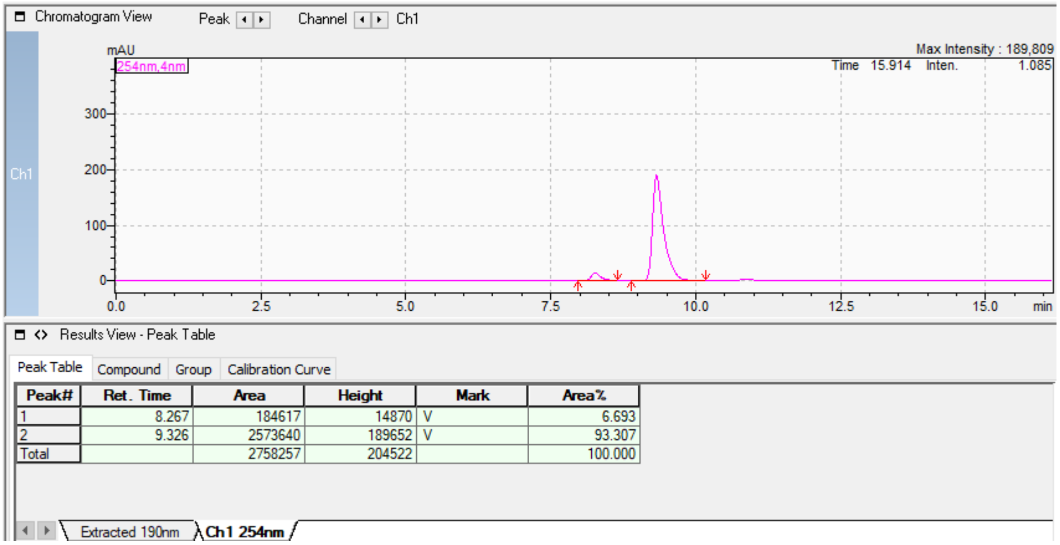

Peak#	Ret. Time	Area	Height	Mark	Area%
1	8.267	184617	14870	V	6.693
2	9.326	2573640	189652	V	93.307
Total		2758257	204522		100.000

%*ee* = 93.307-6.693 = 86.614 = 87%

HPLC trace at 15 h

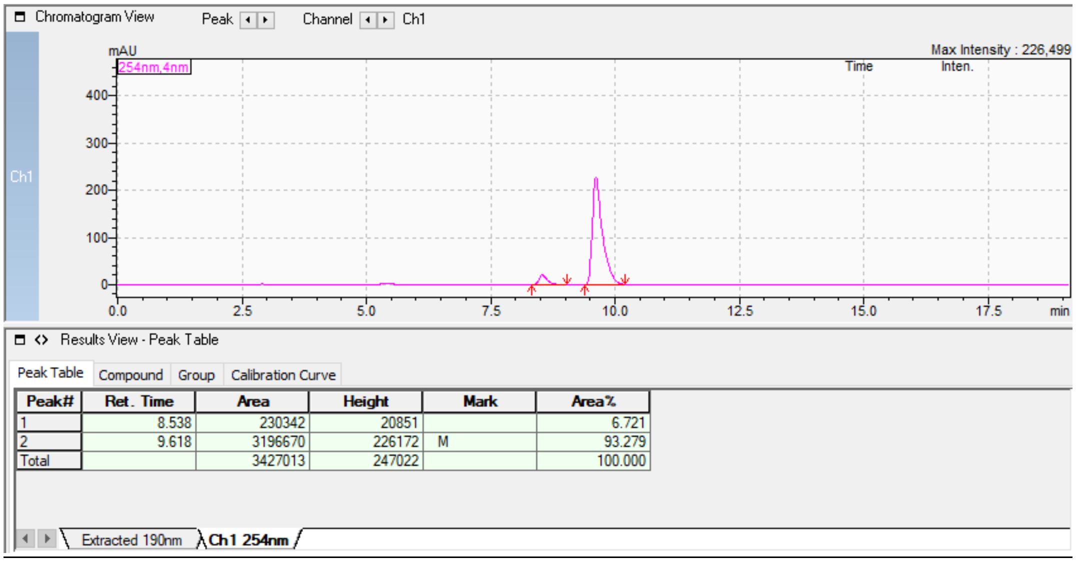

Peak#	Ret. Time	Area	Height	Mark	Area%
1	8.538	230342	20851		6.721
2	9.618	3196670	226172	M	93.279
Total		3427013	247022		100.000

%*ee* = 93.279-6.721 = 86.558 = 87%

Table S2. Reaction monitoring on product **7a** enantioselectivity change over time

	SM **6a** remaining	yield% **7a**	yield% **7ai**	*er* **7a**
3 h	67	22	4	93:7
15 h	23	47	17	93:7

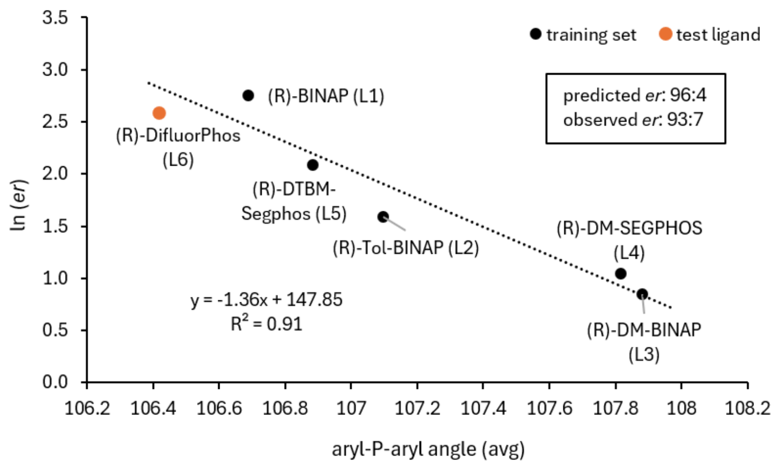

Figure S17. Plot of ln(*er*) of product **7a** relative to aryl-P-aryl angle (average) with (*R*)-DifluorPhos (**L6**) included.

First-Generation Solvent Study Using Rh(cod)$_2$BF$_4$ and Enyne 4a

We performed an initial solvent study using a traditional one-factor-at-a-time (OFAT) approach aimed at determining the impact of different solvent parameters on reactivity and selectivity and to identify solvent parameters that correlate with the measured reaction outcomes of yield and enantioselectivity of the PKR product **5a**, and side product **5ai** formation (**Table S3**).

Enyne **4a** was selected for this study as this substrate, having a 1,1-disubstituted alkene, has not been demonstrated previously in the asymmetric PKR. Rh(cod)$_2$BF$_4$ was selected because of its successful application in the allenic PKR and desymmetrization PKR.[27,28] (*R*)-BINAP was used as the chiral ligand, as the computed activation barrier for the oxidative cyclization step of the asymmetric PKR of 1,6-enynes is low for cationic Rh(I) catalysts having bisphosphine ligands when compared to the background reaction involving only CO-ligands on the Rh metal.[29] A concentration of 10% CO in argon was chosen to maximize enantioselectivity.[5,30] Mesitylene was used as an internal standard for determination of yield and side product formation.[29] Each PKR was performed once using the following amounts: Rh(cod)$_2$BF$_4$ (0.1 equiv), (*R*)-BINAP (0.11 equiv), 10% CO/argon atmosphere, 80 °C, solvent (0.05 M), and mesitylene (1.0 equiv). Each reaction was monitored by ^{1}H NMR and TLC and stopped upon complete consumption of the starting material or no evidence of reaction progress. The yield was determined by comparing the mesitylene peak (s, 6.78 ppm, 3 H) to the product peak (d, 4.62 ppm, 1 H) via ^{1}H NMR of the crude residue. The crude

residue was purified by SiO_2 column chromatography eluting with 30% EtOAc/hexanes prior to HPLC analyses.

Table S3. Solvent study using enyne **4a** and $Rh(cod)_2BF_4$

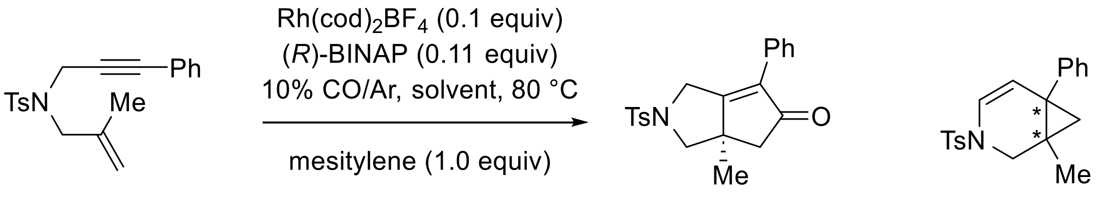

4a **5a** **5ai**

entry	solvent	time h	PKR product **5a** yield% (SM remained)[a]	cycloisomerized side product% **5ai**	*er (ee)*[b] **5a**
1	DCE	43	63 (13)	7	94:6 (88)
2	THF	28	91 (3)	0	90:10 (80)
3	ethanol	65	3 (64)	13	82:18 (64)
4	ethyl acetate	65	74 (trace)	17	94:6 (88)
5	toluene	42	7 (77)	8	90:10 (80)
6	trifluorotoluene	24	81 (2)	0	95:5 (90)
7	dimethyl carbonate	91	46 (4)	33	91:9 (82)
8	acetonitrile	43	0 (100)	0	N.A
9	trifluoroethanol	61	88 (trace)	5	95:5 (90)
10	chloroform	43	7 (62)	0	83:17 (66)
11	1,4-dioxane	90	4 (15)	52	63:37 (26)
12	1,2-dimethoxyethane (DME)	90	16 (6)	59	88:12 (76)

[a]yield determined by ^1H NMR of crude material by the integral comparison of mesitylene (*s*, 6.78 ppm) to the product peak (*d*, 4.62 ppm) [b]enantiomeric excess was determined by chiral HPLC

Initial studies show that for enyne **4a**, the solvent greatly impacted the yield of PKR product **5a** and, to a lesser degree, the enantioselectivity of **5a**, or formation of side product **5ai** (**Table S3**). Tetrahydrofuran (THF), trifluorotoluene, trifluoroethanol provide **5a** in the highest yield (81–91%) with 80–90% ees. Dichloroethane (DCE), ethyl acetate, and dimethylcarbonate gave **5a** in moderate yields (46–74%) with 82–88% *ees*, and varying amounts of **5ai** (7–33%). Ethanol, toluene, chloroform, 1,4-dioxane, 1,2-dimethoxyethane (DME) gave low yields of **5a** (3–16%) having a wide range of *ees* (26–80%) and varying amounts of **5ai** (0–59%).

A preliminary analysis of this data shows that solvents having a dipole moment in the range of 1.48–2.86 and a dielectric constant of 6.08–10.42 perform well in the asymmetric PKR providing **5a** in high yield and high enantioselectivity (**Figure S18**). Solvents extending outside this area are mostly ineffective.

Acetonitrile gave no product after 43 h, which we attribute to the solvent strongly coordinating to the Rh(I) catalyst due to its high dipole moment (dipole moment (D) = 3.924 for acetonitrile, all other solvents (D = 0–2.86). Trifluoroethanol gave an 88% yield and 90% *ee*. This finding was in direct contrast with ethanol, which only afforded 3% of **5a**. We reason that hydrogen bond basicity (β), which is a measure of coordinating ability, may explain this result as the value for ethanol (β = 0.48) is significantly higher than TFE (β = 0.25).

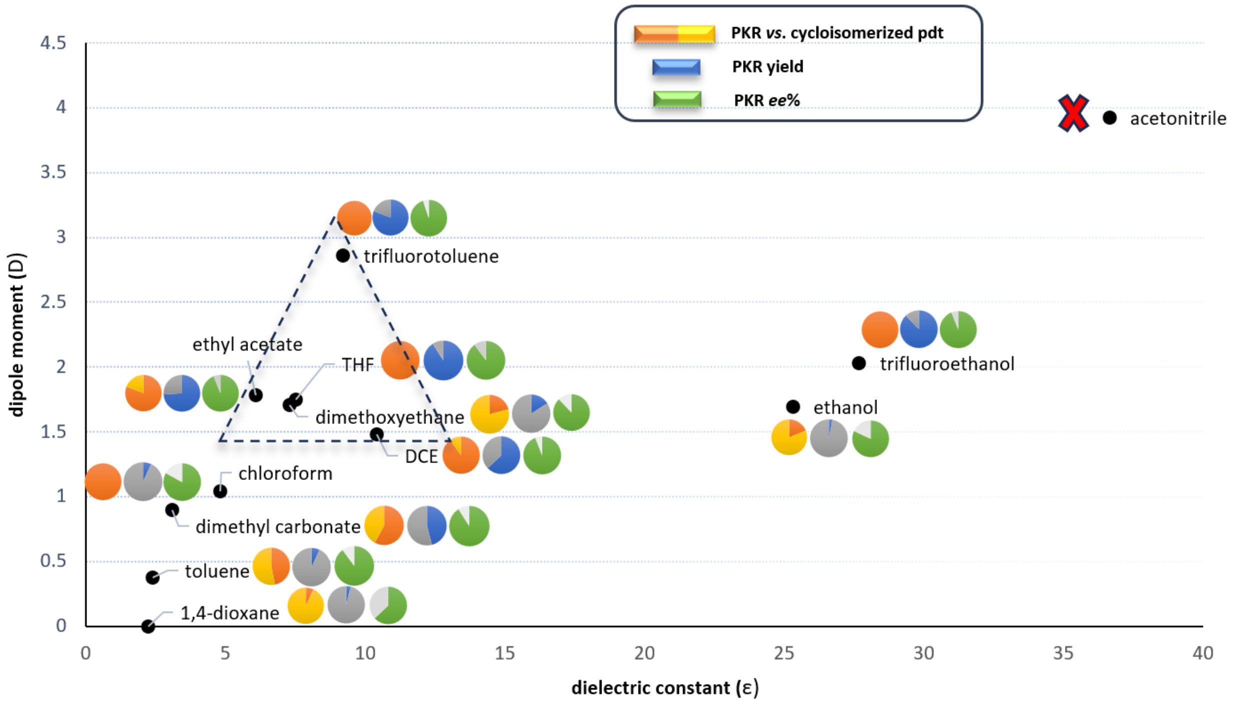

Figure S18. Plot of yield and %*ee* of **5a** against the dielectric constant and the dipole moment of different solvents.

Plotting the yield of **5a** relative to dielectric constant of the solvent shows no correlation (R^2 = 0.005, **Figure S19**. An increase in solvent dipole moment shows an increase in yield for some solvents (**Figure S20**), and a linear fit was observed including 7 of 12 solvents tested (R^2 =0.95) if the same set of low-yielding solvents as observed in Figure S19 was excluded.

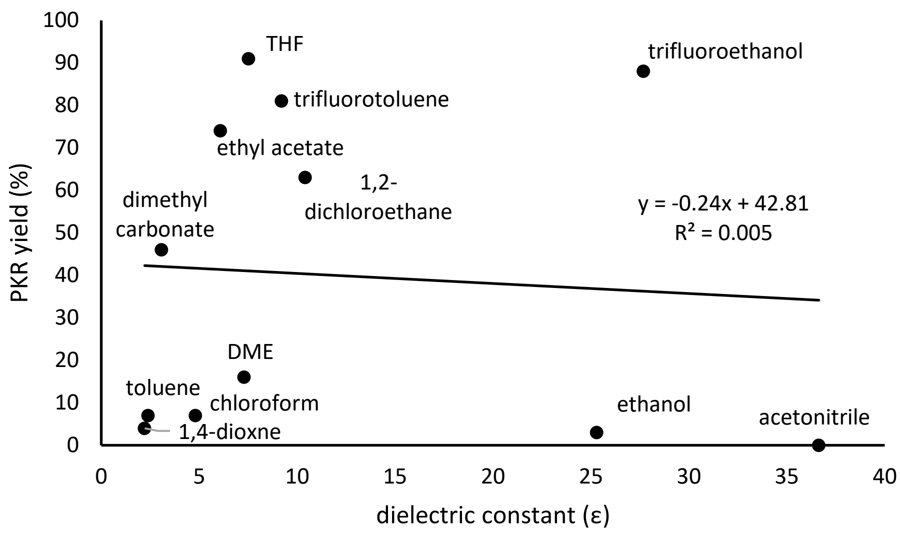

Figure S19. Plot of yield of product **5a** relative to solvent dielectric constant.

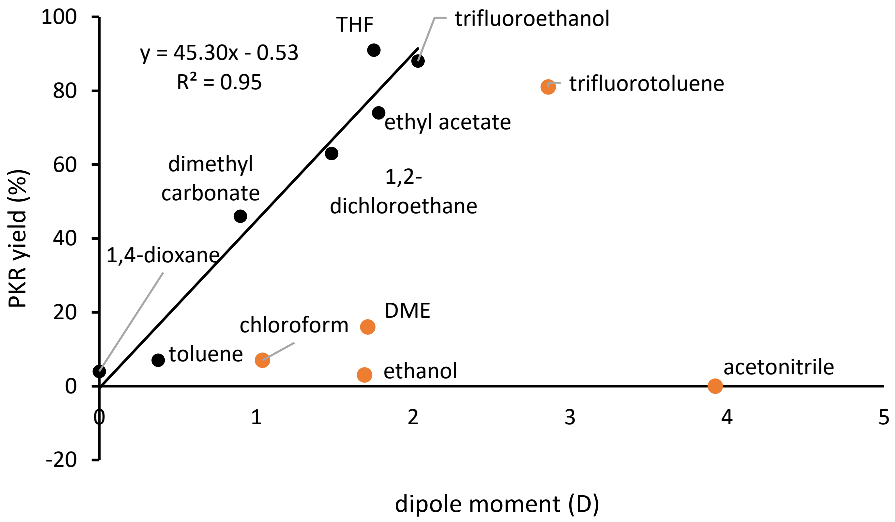

Figure S20. Plot of yield of product **5a** relative to solvent dipole moment (linear fit was generated using the black dots with the orange dots excluded).

To understand the effects of solvent on *ee*%, dielectric constant and dipole moment were plotted relative to ln(*er*) of **5a**. The ln(*er*) showed a low correaltion with solvent dielectric constant (R^2 = 0.06, **Figure S21**). On the contrary, a positive correlation (R^2 = 0.48) was observed when plotting ln(*er*) of **5a** relative to solvent dipole moment (**Figure S22**).

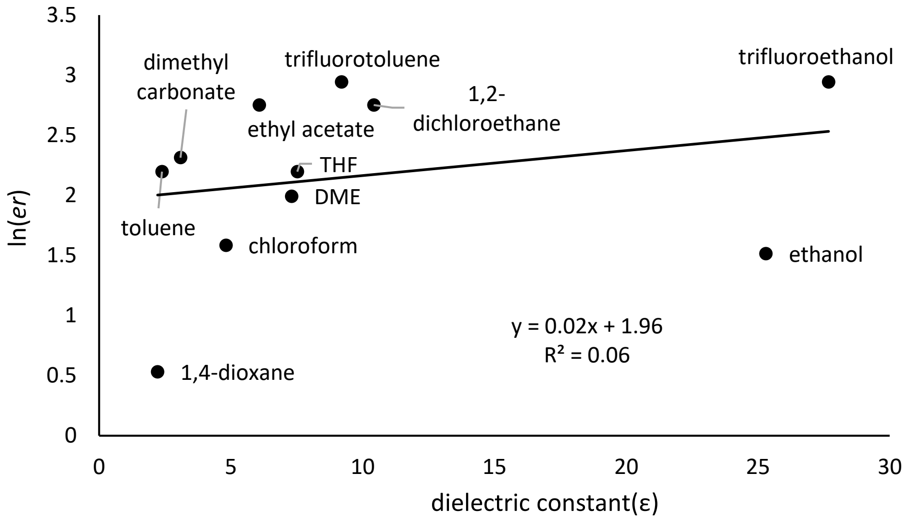

Figure S21. Plot of ln(*er*) of product **5a** relative to the solvent dielectric constant.

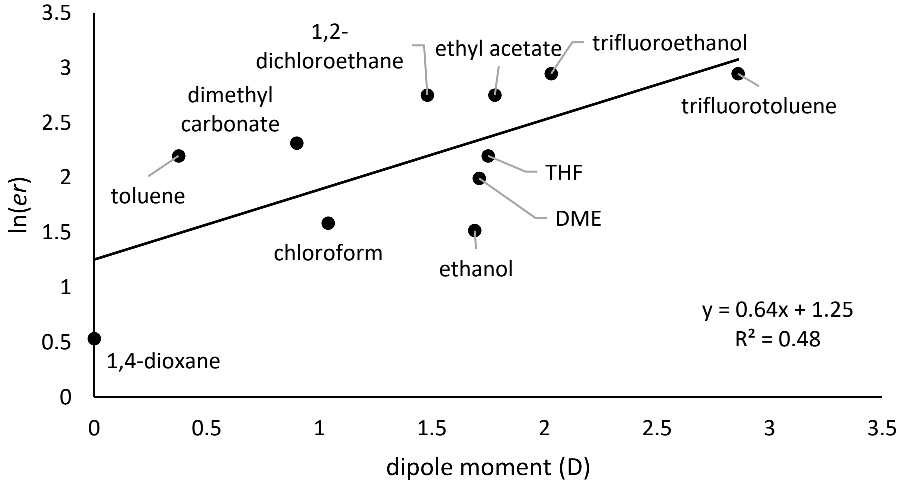

Figure S22. Plot of ln(*er*) of product **5a** relative to the solvent dipole moment.

To identify other relevant descriptors, ln(*er*) were plotted relative to Abraham's hydrogen bond basicity values, as this parameter is also associated with coordination ability of the solvent; a negative correlation (R^2 = 0.26) was observed (**Figure S23**). No correlation was observed when plotting solvent hydrogen bond basicity against the yield of **5a** **(**R^2 = 0.005, **Figure S24**). Plotting solvent π* values, descriptor for solvent polarity and polarizability, relative to the yield and ln(*er*) of **5a** gave low correlations of R^2 = 0.10 and 0.31, respectively (**Figures S25 and S26**).

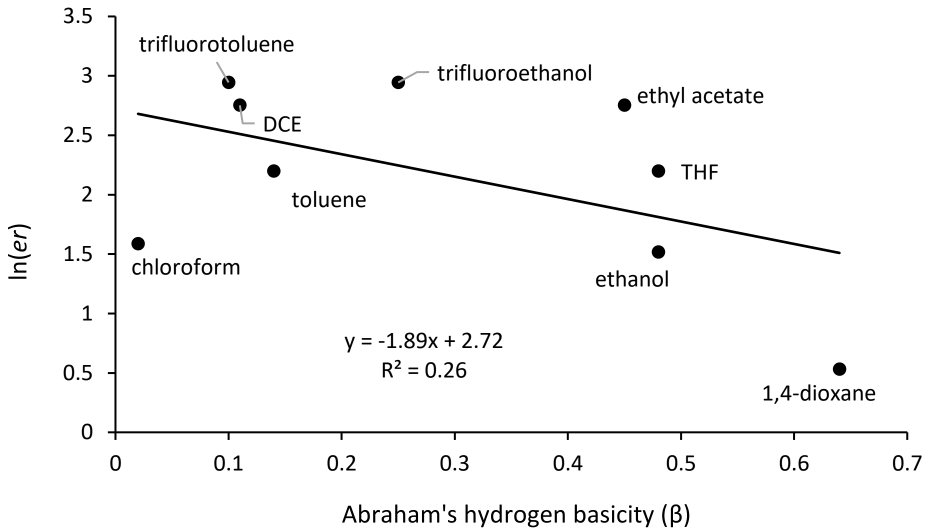

Figure S23. Plot of ln(*er*) of product **5a** relative to solvent hydrogen bond basicity (β).

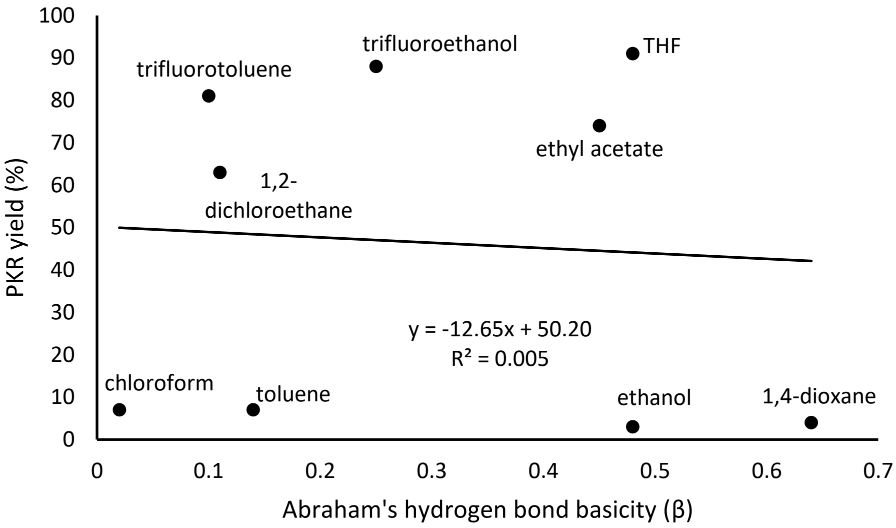

Figure S24. Plot of yield of product **5a** relative to solvent hydrogen bond basicity (β).

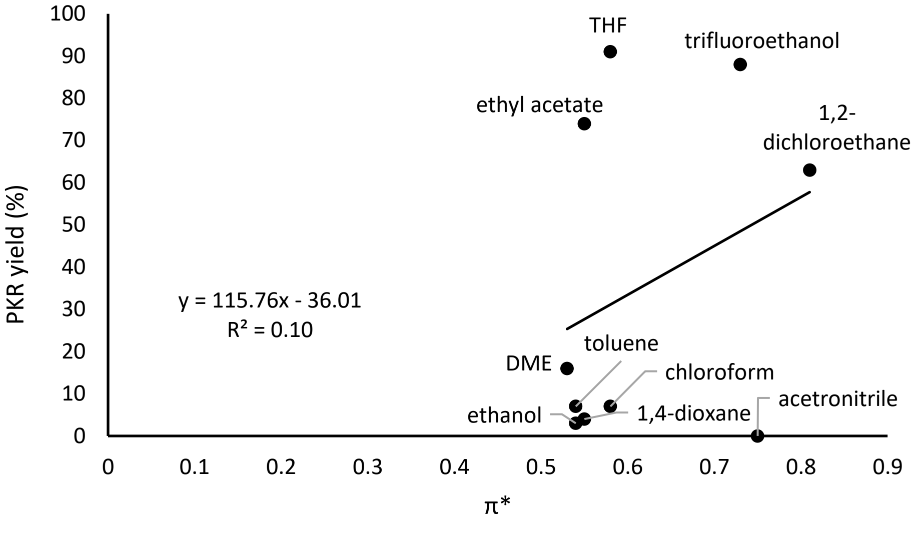

Figure S25. Plot of yield of product **5a** relative to solvent π* values.

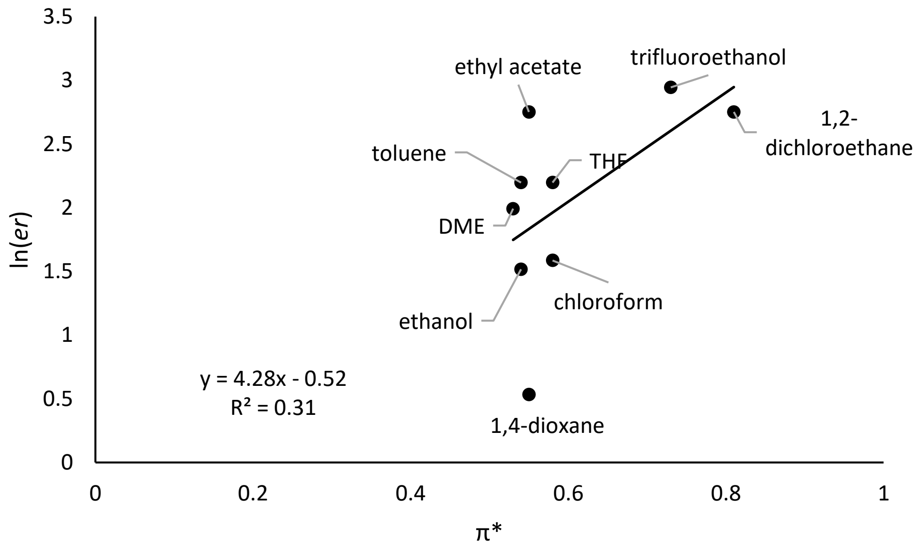

Figure S26. Plot of ln(*er*) of product **5a** relative to solvent π* values.

Second-Generation Solvent Study Using $Rh(cod)_2OTf$ and Enyne 5a and 7a

In the first-generation solvent study, $Rh(cod)_2BF_4$ was used as the precatalyst; however, we subsequently found that $Rh(cod)_2OTf$ afforded the PKR products in higher yield and *ee*% for ether and NTs tethered precursors (Table 4). For the second-generation solvent study, all reagents and reaction conditions used in the first-generation study were maintained except for the precatalyst, $Rh(cod)_2OTf$.

Solvent selection process:

For the second-generation solvent study, we selected tetrahydrofuran (THF), 1,2-dichloroethane (DCE), chloroform, chlorobenzene, trifluorotoluene, ethyl acetate, toluene, ethanol, 1,4-dioxane, trifluoroethanol and dimethyl carbonate. These solvents were selected as they feature a range of values for dipole moment (0–2.86) and dielectric constant (2.219–27.68), and hydrogen bond basicity (0.02–0.64). Chlorobenzene was added because its dipole moment and dielectric constant are similar to THF, which gave high yield and %*ee* in the first generation solvent study. Two solvents that were used in the first-generation solvent study were removed from this study: acetonitrile as it did not provide any PKR product; and dimethoxyethane due to the side product being the major product.

These solvents encompass distinct qualitative categories, including non-polar aprotic (i.e., toluene, 1,4-dioxane), polar protic (ethanol, trifluoroethanol) and polar aprotic (THF, DCE, chloroform, trifluorotoluene, ethyl acetate, chlorobenzene, and dimethyl carbonate) (**Table S4**).

Table S4. Solvents used in the second-generation solvent study of asymmetric PKR

Solvent	Dielectric constant (ε)[31]	Dipole moment (**D**)[31]	Abraham's hydrogen bond basicity (**β**)[32]	π*[33]	General classification	PKR[b]
tetrahydrofuran	7.52 (22)[a]	1.75 (25, B)[a]	0.48	0.58	Polar aprotic	Yes
1,2-dichloroethane	10.42 (20)	1.48	0.11	0.81	polar aprotic	Yes
ethyl acetate	6.081 (20)	1.78	0.45	0.55	Polar aprotic	Yes
chlorobenzene	5.69 (20)	1.69	0.07	0.71	Polar aprotic	Yes
dimethyl carbonate	3.087 (25)	0.9	N/A	N/A	Polar aprotic	No
trifluorotoluene	9.2 (30)	2.86[34]	0.1[35]	0.64[36]	Polar aprotic	No
toluene	2.385 (20)	0.375	0.14	0.54	Nonpolar aprotic	Yes
trifluoroethanol	27.68 (20)	2.03 (25, cHex)	0.25	0.73	Polar protic	No
1,4-dioxane	2.219 (20)	0	0.64	0.55	Nonpolar aprotic	Yes
chloroform	4.807 (25)	1.04	0.02	0.58	Polar aprotic	Yes
ethanol	25.3 (20)	1.69	0.48	0.54	Polar protic	No

Dimethoxyethane[c]	7.3	1.71 (25, B)	N/A	0.53	Polar aprotic	Yes
Acetonitrile[c]	36.64	3.924	0.32	0.75	Polar aprotic	Yes

a. The temperature at which the dielectric constant or dipole moment were determined is shown in parentheses, the solvent used to measure dipole moment is indicated as a symbol, with benzene as "B" and cyclohexane as "cHex".

b. "Yes" indicates this solvent has been used in a successful enyne PKR, whereas "No" indicates it hasn't been used in a successful enyne PKR .

c. Solvent that was only used in the first-generation solvent study with $Rh(cod)_2BF_4$

The solvent selection process was guided by those that have been successfully used in the Rh(I)-catalyzed PKR (labelled as "Y" in **Table S4**). Solvents previously used in the PKR that were not selected for this study are dibutyl ether, xylenes, and acetone. Each of these solvents was not included for technical reasons associated with their boiling points: xylenes and dibutyl ether make product purification difficult because of their high boiling points, and acetone evaporation was problematic during the PKR due to its volatility. Trifluoroethanol and ethanol were included to test PKR outcomes in the presence of protic solvents and to establish a hydrogen bond basicity comparison. Trifluorotoluene and toluene were included to establish a comparison of dielectric constant and dipole moment on PKR reactivity and selectivity. Dimethyl carbonate was selected as it is categorized as green solvent due to its low toxicity and rapid biodegradability.[37]

For each solvent, the PKR was performed twice using the N-Ts tethered precursor **4a** and the yield and *ees* are reported as run 1 and run 2. The averages for these two runs are reported. The ether-tethered PKRs were each performed one time. Each PKR was conducted using the following reaction conditions: $Rh(cod)_2OTf$ (0.10 equiv), (*R*)-BINAP (0.11 equiv), 10% CO in argon atmosphere, 80 °C, solvent (0.05 M), mesitylene (1.0 equiv). An initial aliquot was removed from the flask to determine the enyne/mesitylene concentration by ^{1}H NMR using the enyne **4a** (s, 4.25 ppm) and **6a** (s, 4.36 ppm) relative to the mesitylene (s, 6.78 ppm). A second aliquot was taken at 24 h to determine product yield (d, 4.62 ppm for **5a** and d, 4.61 ppm for **7a**), b.r.s.m. (yield based on recovered starting material) and %*ee* (purified by prep TLC to get %*ee* by HPLC) (**Table S5** for the NTs-tether precursor **4a** and Table **S6** the ether-tethered precursor **6a**).

Table S5. Second-generation solvent study using $Rh(cod)_2OTf$ and enyne **4a**

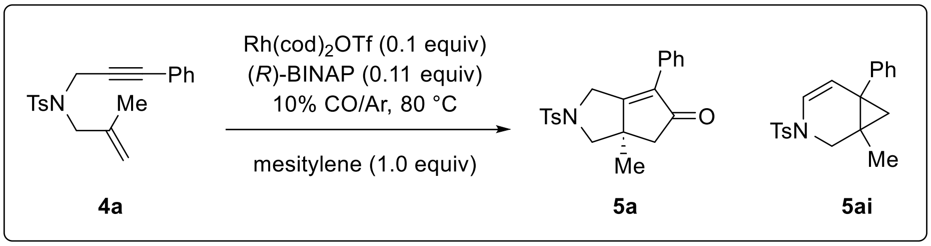

solvent	run 1 **5a** yield% (**5ai**)[a]	run 2 **5a** yield% (**5ai**)	avg. **5a** yield% (**5ai**)	run 1 **5a** b.r.s.m %	run 2 **5a** b.r.s.m. %	avg. **5a** b.r.s.m. %	run 1 **5a** *er*	run 2 **5a** *er*	avg. **5a** %*ee*
THF	99	98	99	99	99	99	95:5	95:5	90
1,2-dichloroethane	72 (7)	82 (5)	77 (6)	92	94	93	93:7	93:7	86
ethyl acetate	25 (trace)	21 (trace)	23 (trace)	96	91	94	93:7	94:6	88
trifluorotoluene	46% (trace)	46(trace)	46 (trace)	81	82	82	92:8	90:10	82
trifluoroethanol	27 (4)	22 (3)	25 (4)	84	59	72	94:6	95:5	89
toluene	25 (5)	30 (4)	28 (5)	61	71	66	89:11	89:11	78
ethanol	36	34	35	72	69	71	94:6	95:5	89
chlorobenzene	31 (2)	32 (2)	32 (2)	72	73	73	83:17	83:17	66
1,4-dioxane	36 (4)	26 (trace)	31 (2)	78	76	77	89:11	88:12	76
chloroform	61	69	65	86	91	89	97:3	97:3	94
dimethyl carbonate	46 (3)	50 (trace)	48(2)	87	93	90	91:9	91:9	83

[a]cycloisomerized side product formed as determined by ^{1}H NMR of crude material with internal standard mesitylene

Table S6. Second-generation solvent study using Rh(cod)$_2$OTf and enyne **6a**

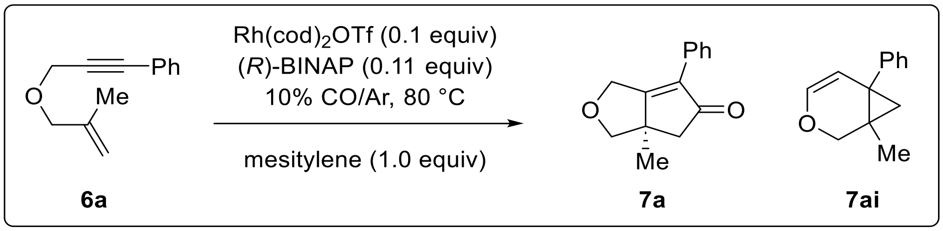

solvent	run 1 **7a** yield% (**7ai**)[a]	run 1 **7a** b.r.s.m.%	run 1 **7a** *er*	run 1 **7a** %*ee*
THF	52	78	15:85	69
1,2-dichloroethane[b]	84 (13)	85	6:94	89
ethyl acetate	40	53	15:85	69
trifluorotoluene	26 (15)	55	7:93	87
trifluoroethanol	80	81	8:92	85
toluene	0 (4)	0	N/A	N/A
ethanol	52	53	15:85	69
chlorobenzene	22 (3)	67	8:92	84
1,4-dioxane	19 (4)	36	20:80	59
chloroform	trace	0	N/A	N/A
dimethyl carbonate	32	39	22:78	56

[a]cycloisomerized side product formed as determined by ^{1}H NMR of crude material; [b]reaction performed at 85 °C

Selected plots of physical descriptors relative to PKR yield, b.r.s.m. and ln(*er*) for the second-generation solvent study using $Rh(cod)_2OTf$ and NTs tethered precursor 4a

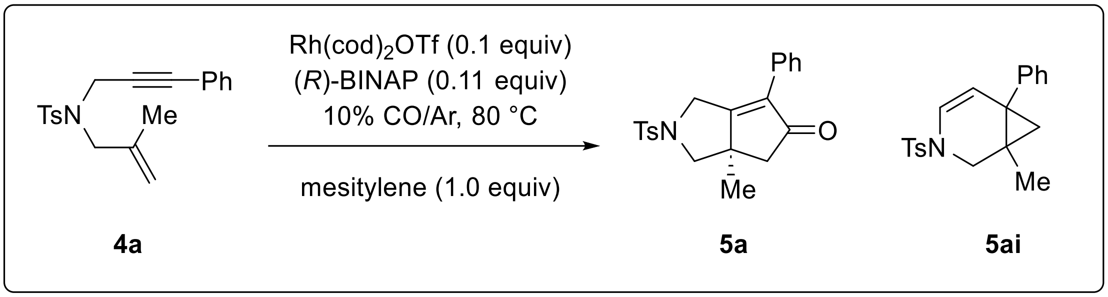

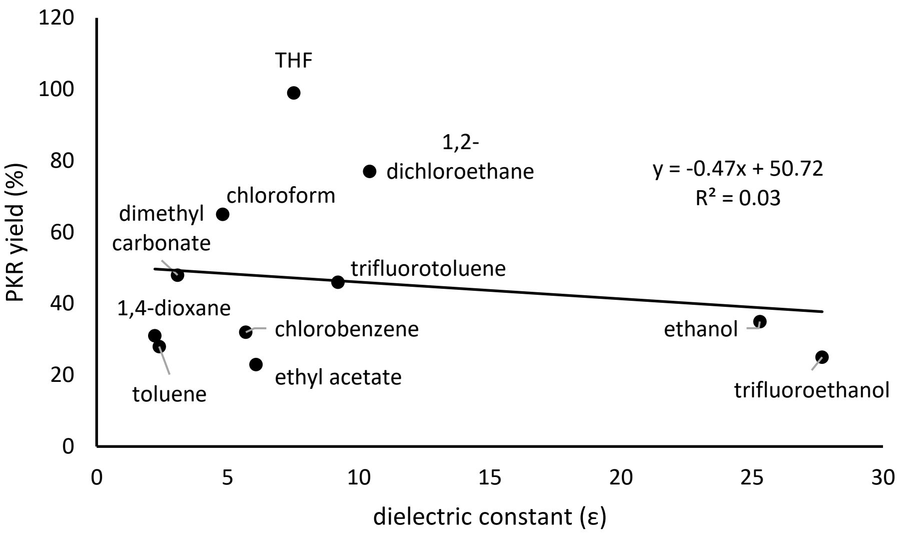

Figure S27. Plot of yield of product **5a** relative to solvent dielectric constants.

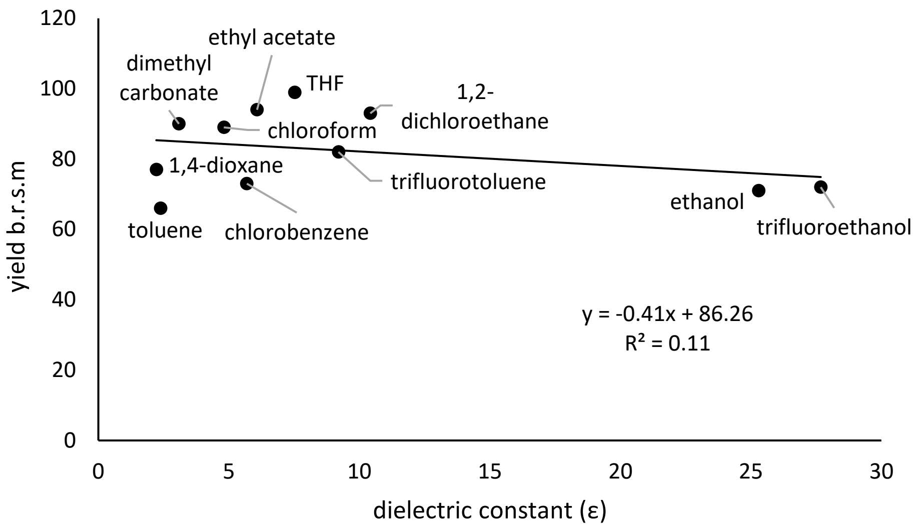

Figure S28. Plot of yield b.r.s.m of product **5a** relative to solvent dielectric constants.

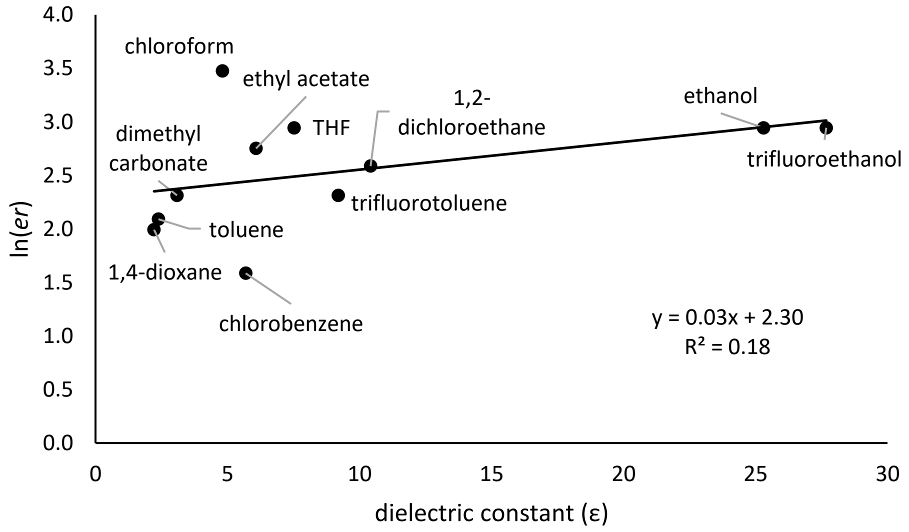

Figure S29. Plot of ln(*er*) of product **5a** relative to solvent dielectric constant.

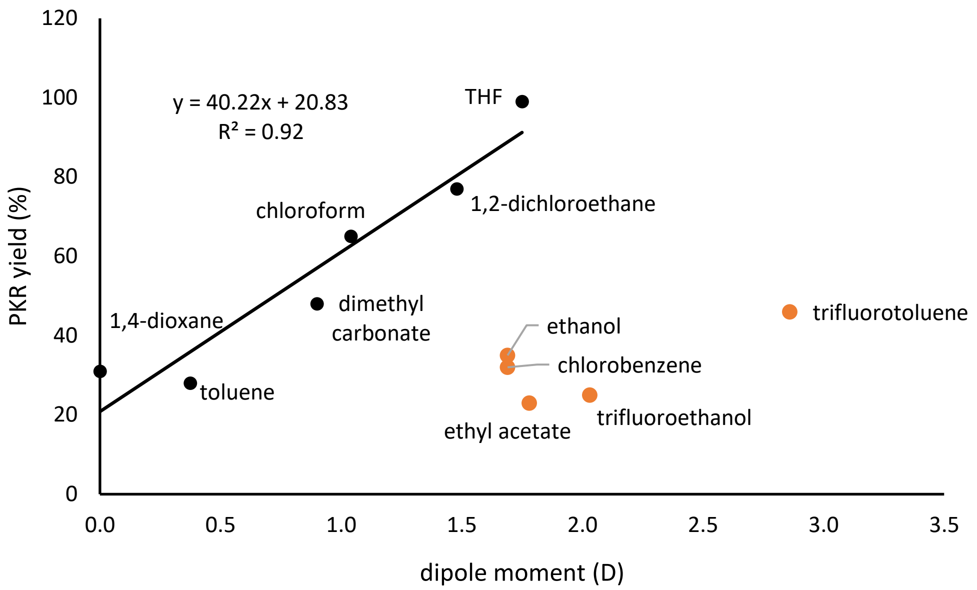

Figure S30. Plot of yield of product **5a** relative to solvent dipole moment (linear fit was generated using the black dots with the orange dots excluded).

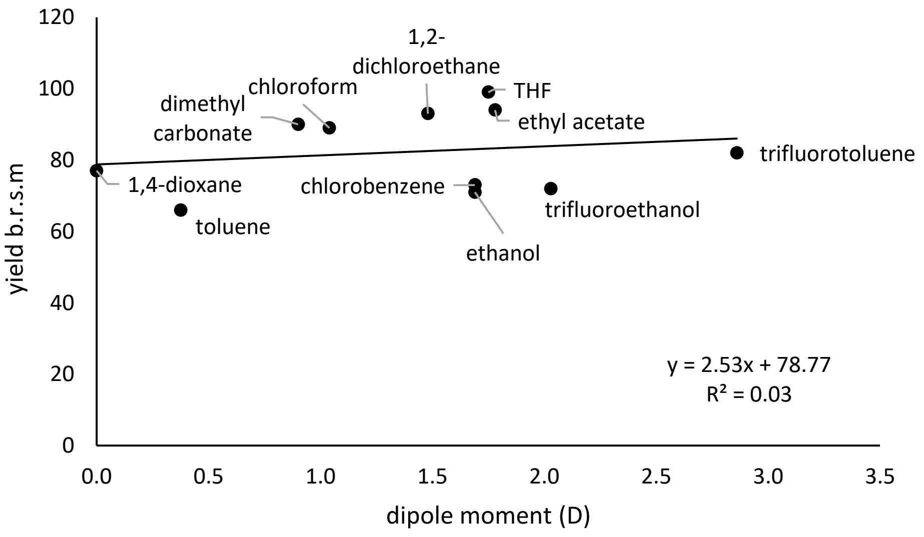

Figure S31. Plot of yield b.r.s.m. of product **5a** relative to solvent dipole moment.

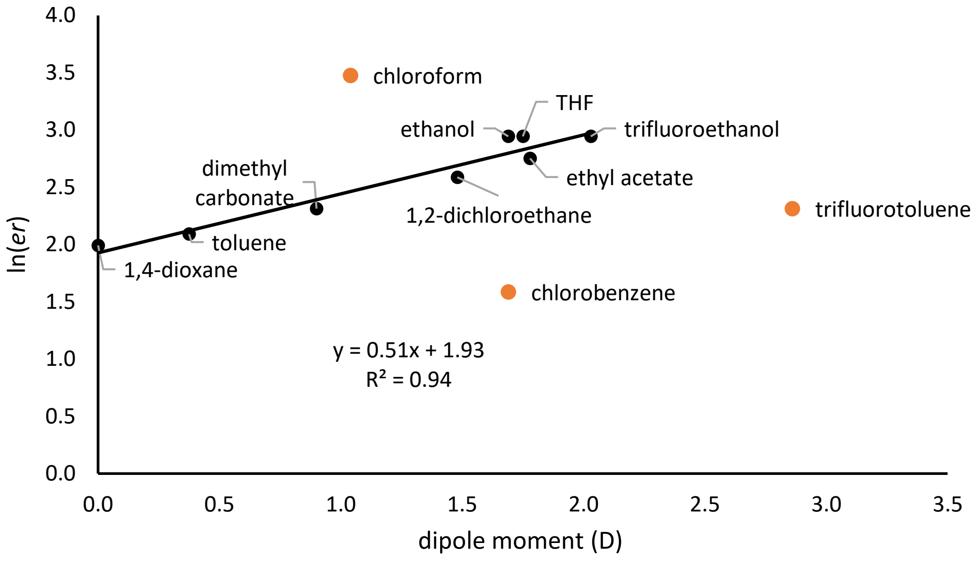

Figure S32. Plot of ln(*er*) of product **5a** relative to solvent dipole moment.

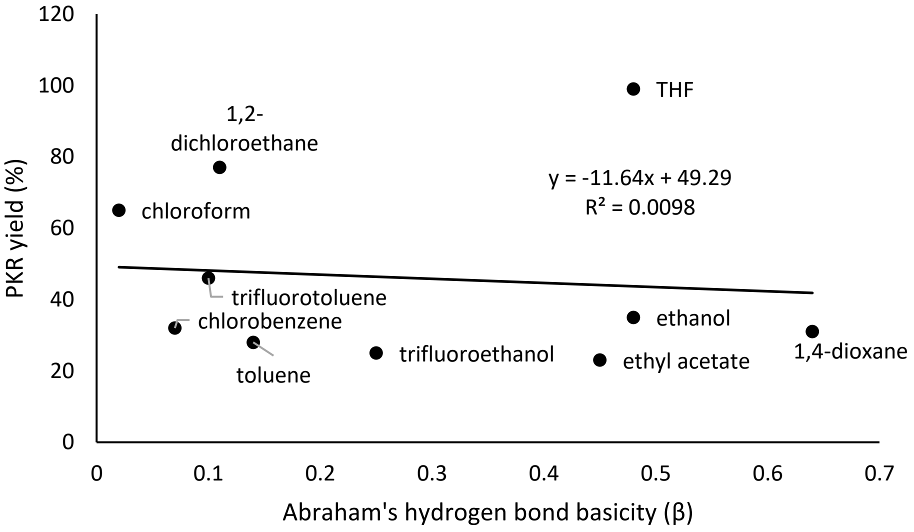

Figure S33. Plot of yield of product **5a** relative to solvent Abraham's hydrogen bond basicity

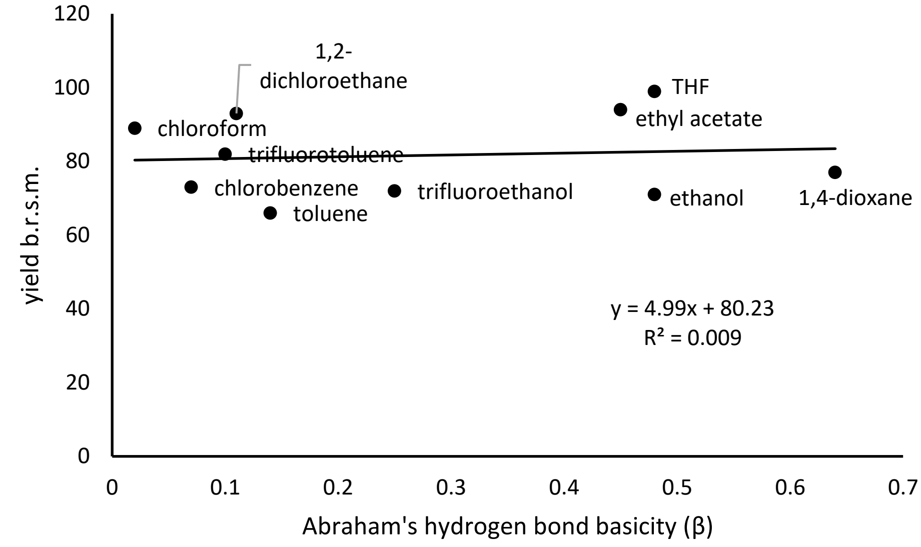

Figure S34. Plot of yield b.r.s.m. of product **5a** relative to solvent Abraham's hydrogen bond basicity.

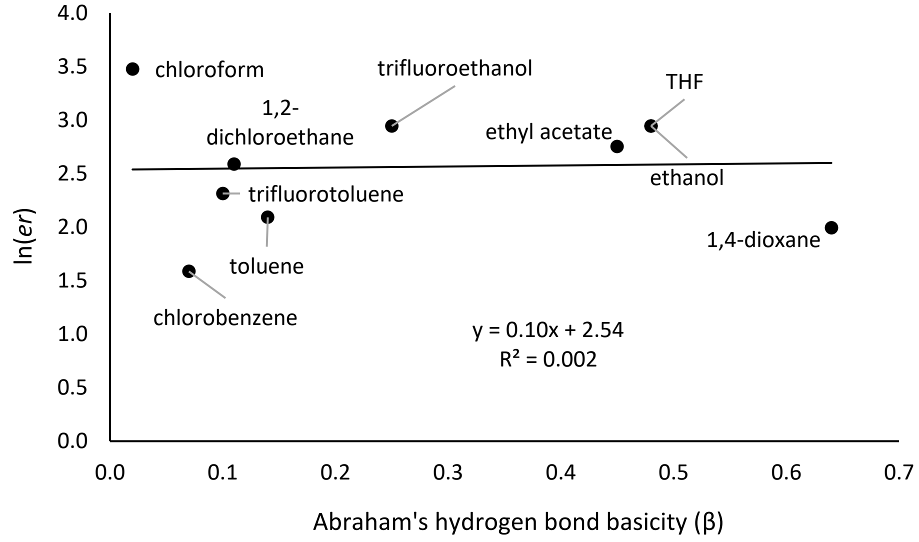

Figure S35. Plot of ln(*er*) of product **5a** relative to Abraham hydrogen bond basicity (β).

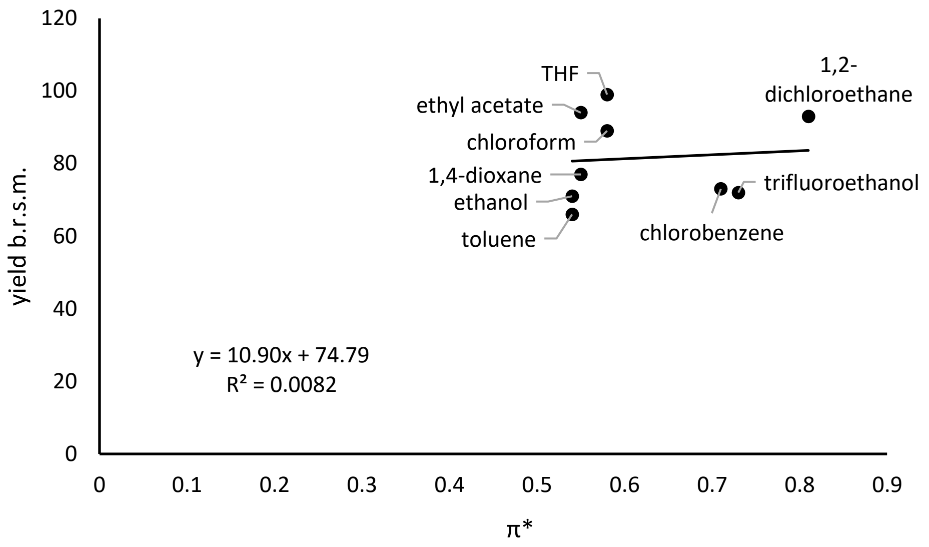

Figure S36. Plot of yield b.r.s.m. of product **5a** relative to solvent π*.

Interestingly, our investigation revealed that solvent boiling points correlated well with ln(*er*) of product **5a** (**Figure S37**). Given the multifaceted nature of solvent properties influencing boiling points, including factors such as dipole-dipole, hydrogen-bonding, London dispersion forces, we explored the simultaneous consideration of two descriptors to identify a correlation with PKR %*ee*. For example, when plotting the experimental Kamlet-Taft β values relative to the experimental π* values,[38] solvents that gave lower %*ee* are clustered in a specific region (**Figure S38**), including 1,4-dioxane (76% *ee*), toluene (78% *ee*) and chlorobenzene (66% *ee*). We interpret this clustering as indicative of the collective influence of multiple physical properties of solvents on PKR enantioselectivity, which are reflected by a singular parameter, such as boiling point.

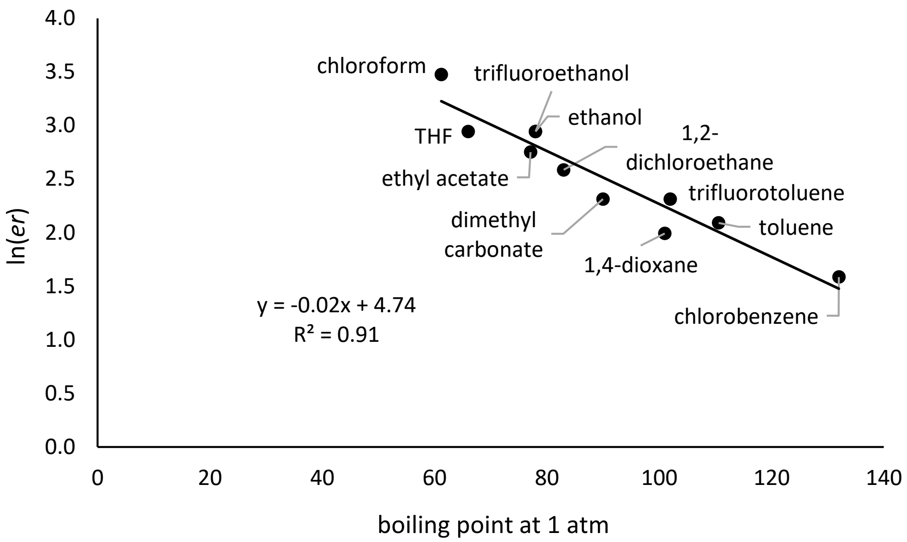

Figure S37. Plot of ln(*er*) of product **5a** relative to solvent boiling points.

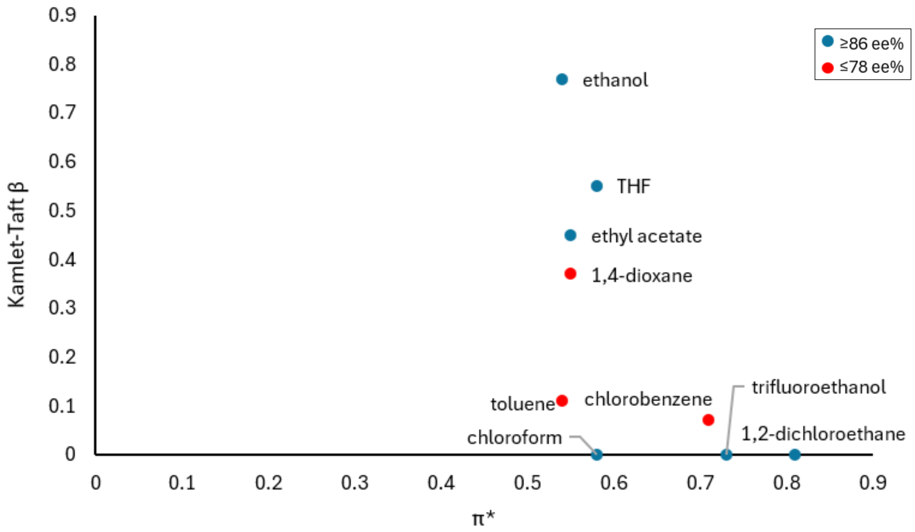

Figure S38. Plot of %*ee* of product **5a** relative to experimental Kamlet-Taft β values and experimental π* values (trifluorotoluene and dimethyl carbonate were not included in the analysis due to the lack of experimental Kamlet-Taft β and π* values).

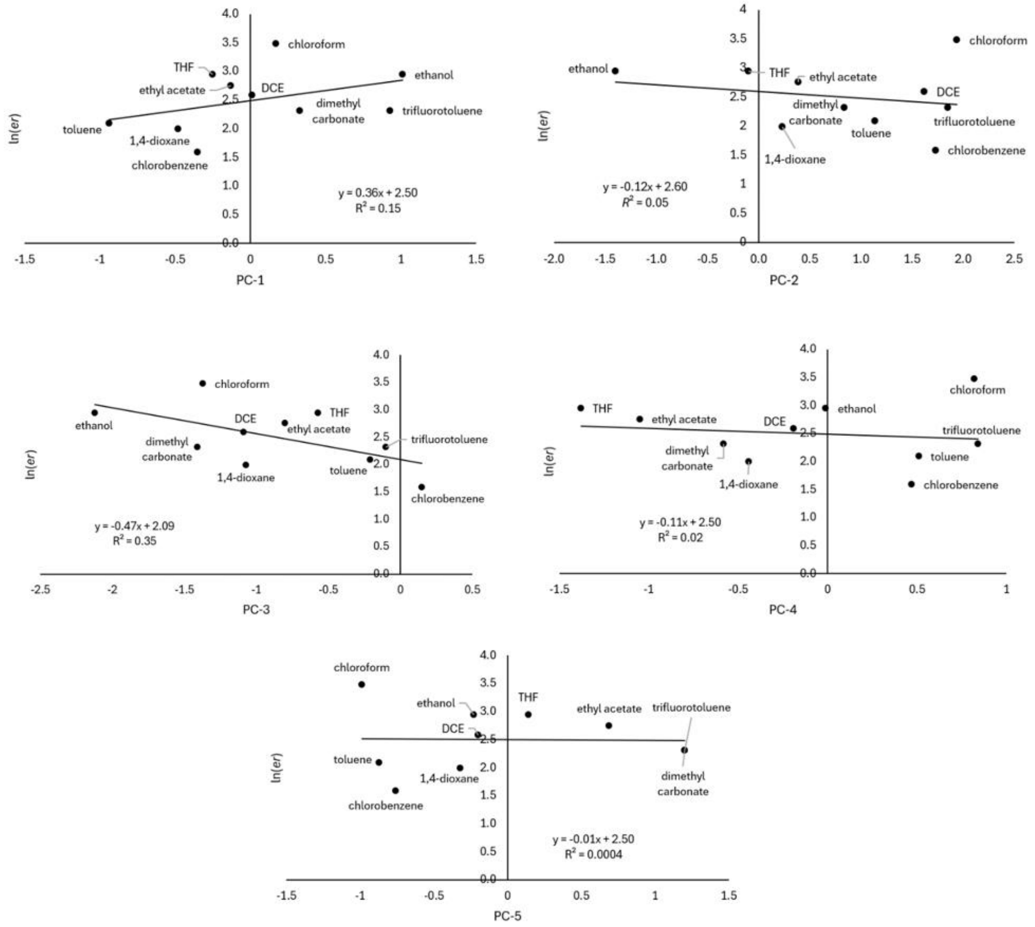

Figure S39. Plot of ln(*er*) of product **5a** relative to the solvent database developed by Katritzky (trifluoroethanol is not represented as it was not included in the Katritzky database)[39]

Selected plots of physical descriptors relative to PKR yield, b.r.s.m. and ln(*er*) for the second-generation solvent study using Rh(cod)$_2$OTf and O tethered precursor 6a

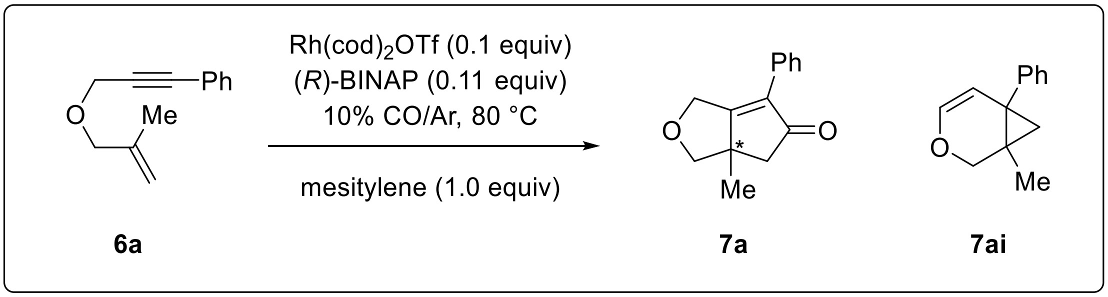

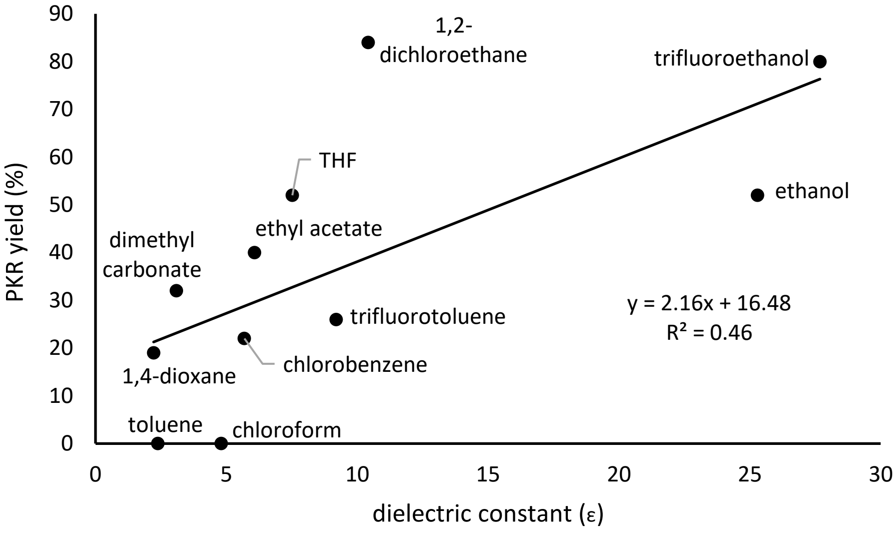

Figure S40. Plot of yield of product **7a** relative to solvent dielectric constants.

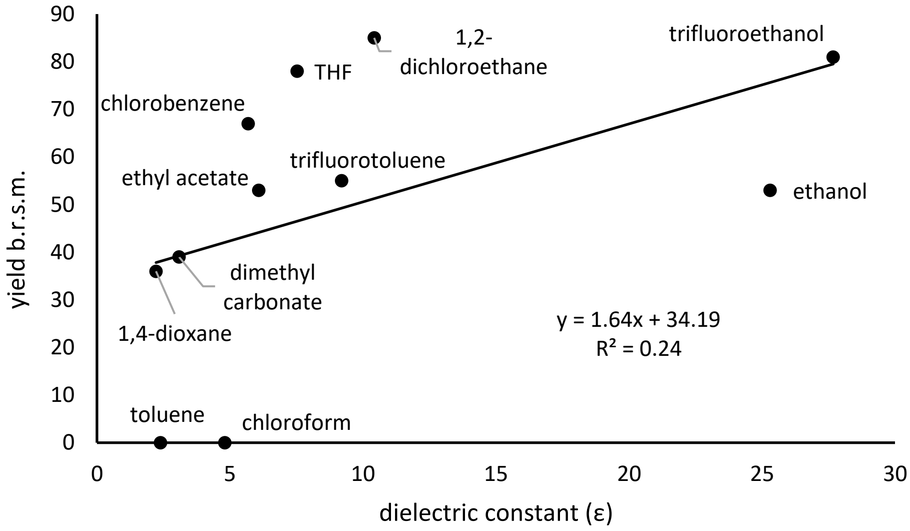

Figure S41. Plot of yield b.r.s.m. of product **7a** relative to solvent dielectric constants.

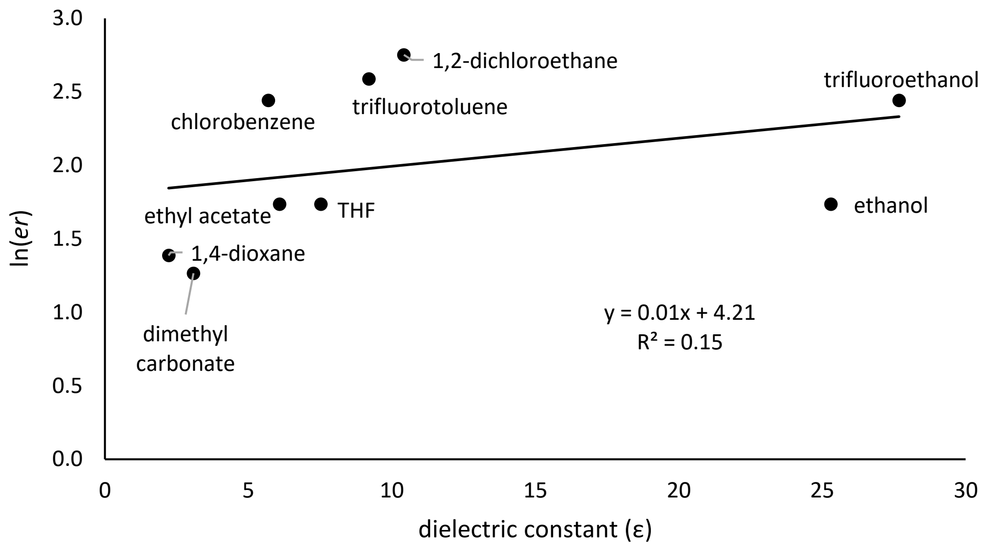

Figure S42. Plot of ln(*er*) of product **7a** relative to solvent dielectric constants.

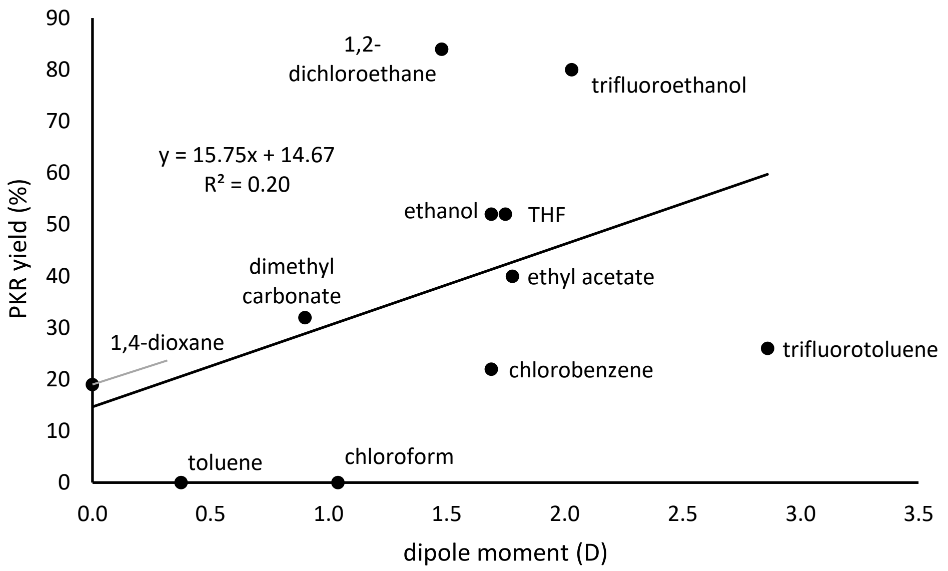

Figure S43. Plot of yield of product **7a** relative to solvent dipole moments.

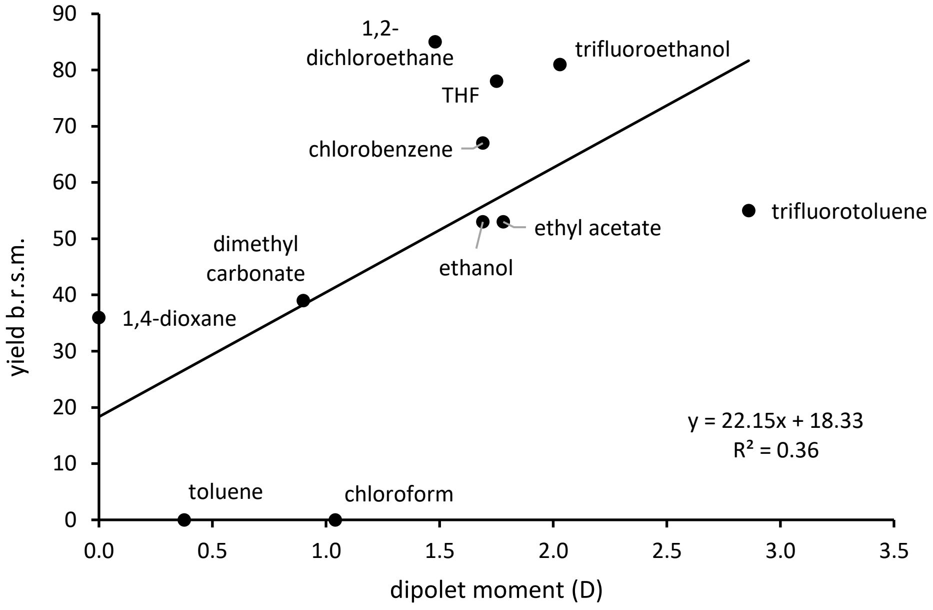

Figure S44. Plot of yield b.r.s.m. of product **7a** relative to solvent dipole moments.

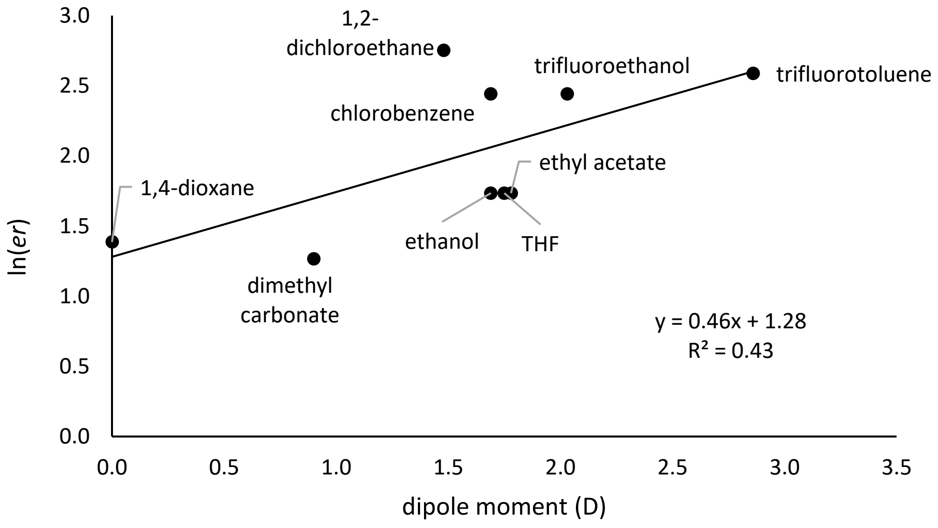

Figure S45. Plot of ln(*er*) of product **7a** relative to solvent dipole moments.

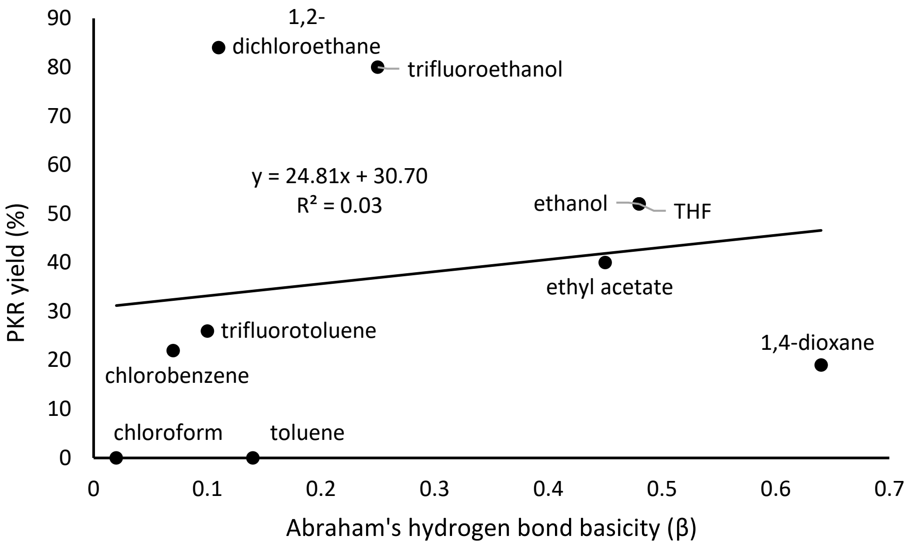

Figure S46. Plot of yield of product **7a** relative to Abraham's hydrogen bond basicity (β).

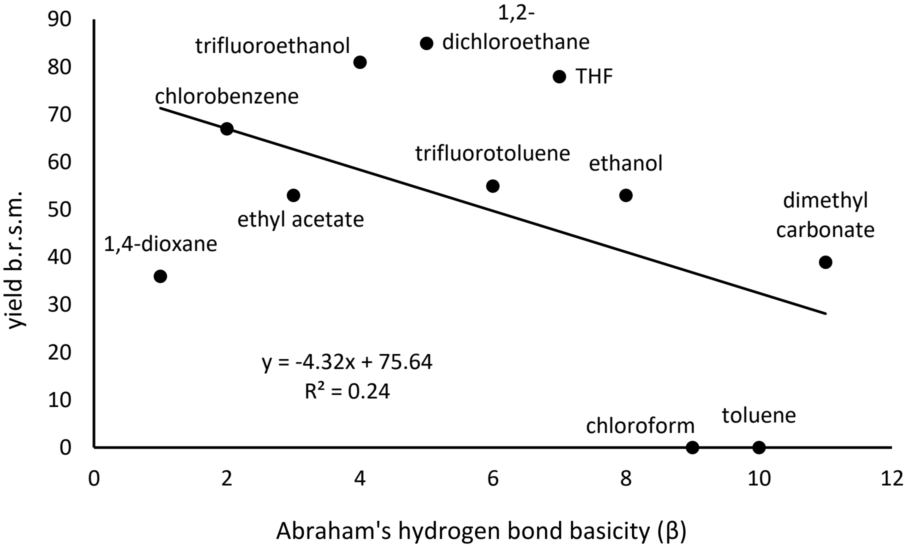

Figure S47. Plot of yield b.r.s.m. of product **7a** relative to solvent Abraham's hydrogen bond basicities.

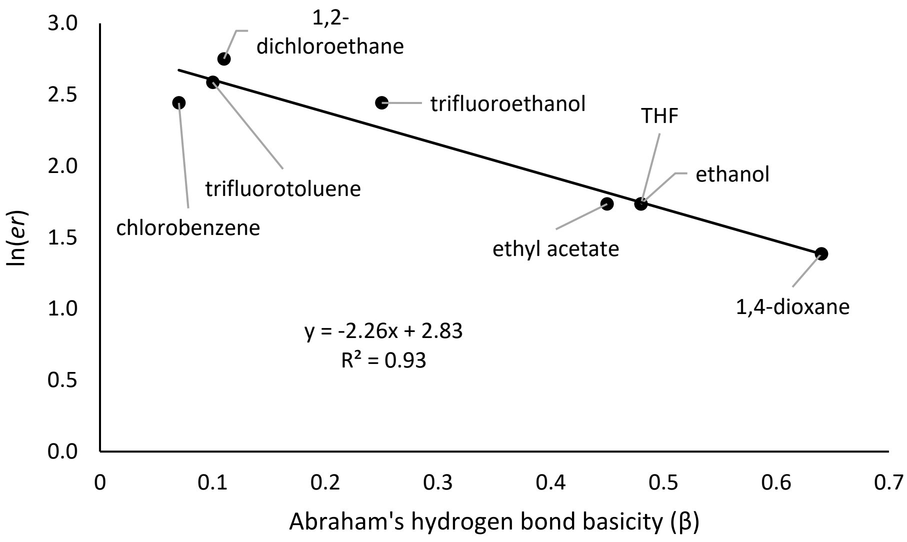

Figure S48. Plot of ln(*er*) of product **7a** relative to Abraham's hydrogen bond basicity (β).

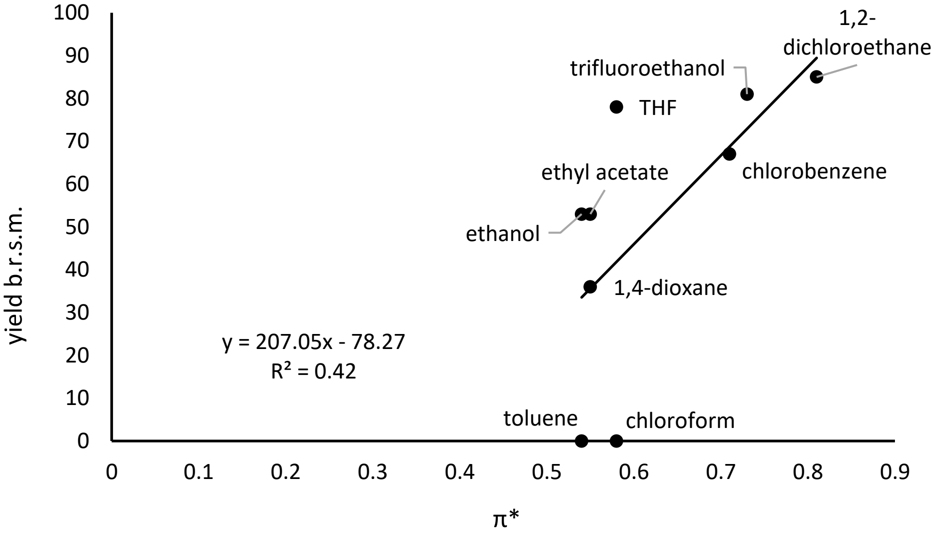

Figure S49. Plot of yield b.r.s.m. of product **7a** relative to solvent π*.

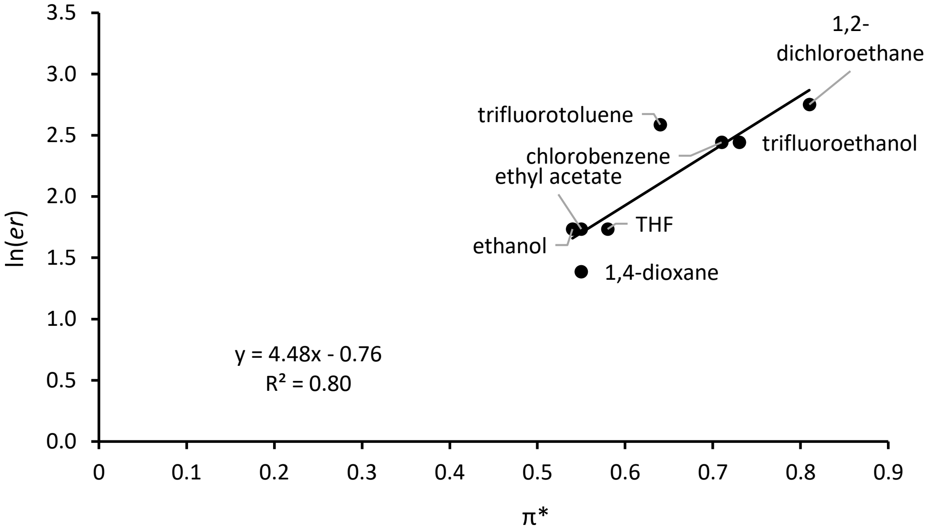

Figure S50. Plot of ln(*er*) of product **7a** relative to solvent π*.

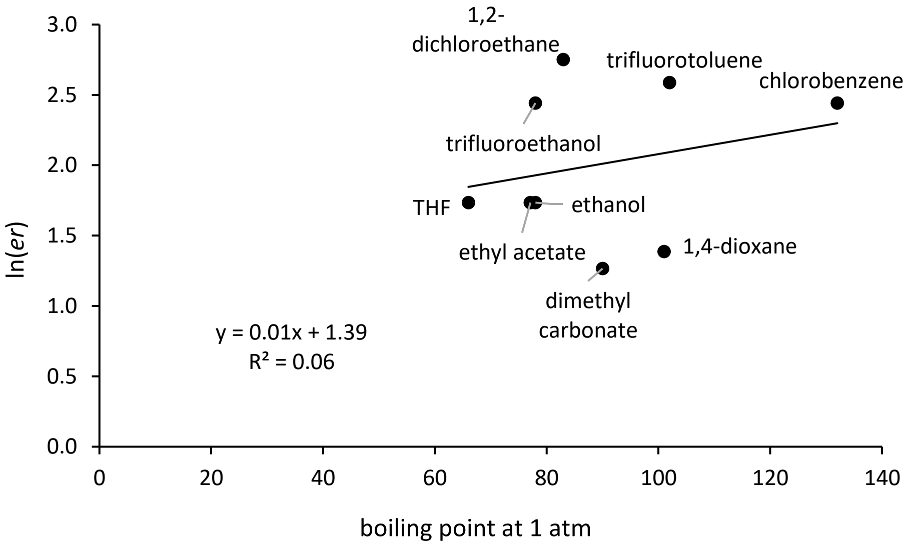

Figure S51. Plot of ln(*er*) of product **7a** relative to solvent boiling points at 1 atm.

Rh(I) Precatalysts

Table S7. Identification of reagents and reaction conditions for solvent studies

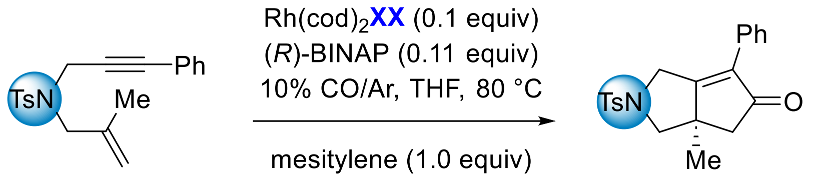

4a **5a**

entry	Rh catalyst	time h	PKR yield%[a]	b.r.s.m. %	*er*[b]	coordinating ability of counterion (a^{TM})[40]
1	$Rh(cod)_2BF_4$	28	91(3)[c]	94	90:10	-1.1
**2**	**$Rh(cod)_2OTf$**	**22**	**99**	**99**	**95:5**	-0.4
3	$Rh(cod)_2SbF_6$	36	91(2)[c]	93	89:11	-1.0
4	$[Rh(CO)_2Cl]_2$[d]	64	39(37)[c]	62	93:7	-0.4
5	$[Rh(cod)Cl]_2$[d]	22	80(20)[c,e]	100	96:4	-0.4

[a]yield determined through ^{1}H NMR of crude material by comparing the mesitylene peak (s, 6.78 ppm) to the product peak (d, 4.62 ppm); [b]*er* determined by HPLC using Chiralpak IB N-3 column eluting with 15% IPA/hexanes with a flow rate of 1.0 mL/min and detect at 254 nm; [c]yield in parentheses shows the starting material remaining as determined by ^{1}H NMR; [d]5 mol% of the Rh catalyst and 15 mol% of AgOTf were used to generate cationic Rh *in situ;* [e]reaction stopped progressing at 22 h as evidenced by ^{1}H NMR of crude material

Entry 1: Follows General Procedure **D** for asymmetric PKR: $Rh(cod)_2BF_4$ (6.1 mg, 0.015 mmol, 0.1 equiv), (*R*)-BINAP (10.3 mg, 0.0165 mmol, 0.11 equiv), mesitylene (18.0 mg, 0.15 mmol, 1.0 equiv) and enyne **4a** (0.5 mL from a stock solution of 0.3 M) in THF (3.0 mL, 0.05 M). The reaction flask was lowered into the preheated oil bath (80 °C). After 28 h, the PKR yield (91%), b.r.s.m. (94%) and starting material remaining (3%) were determined based on the integral comparison of the product peak (d, 4.62 ppm, 1H) and starting material peak (s, 4.25 ppm, 2 H) to the internal standard mesitylene peak (s, 6.78 ppm, 3 H) via ^{1}H NMR. The crude was purified by SiO_2 chromatography eluting with 30% ethyl acetate/hexanes for HPLC analysis to get an *er* of 90:10.

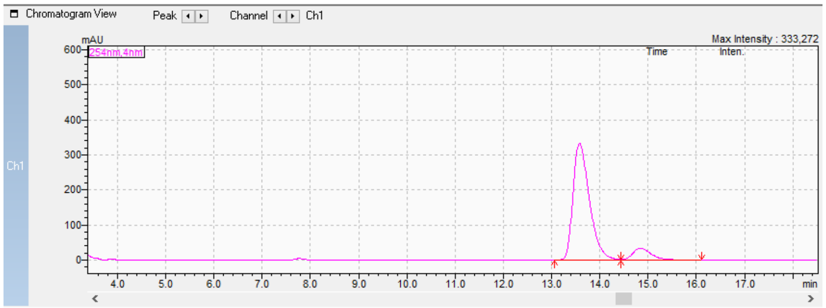

Results View - Peak Table

Peak#	Ret. Time	Area	Height	Mark	Area%
1	13.578	7865484	332846		89.931
2	14.841	880677	33558	V	10.069
Total		8746161	366404		100.000

%*ee* = 89.931-10.069 = 79.862 = 80%

Entry 2: Follows General Procedure **D** for asymmetric PKR: Rh(cod)$_2$OTf (7.1 mg, 0.015 mmol, 0.1 equiv), (*R*)-BINAP (10.3 mg, 0.0165 mmol, 0.11 equiv), mesitylene (18.0 mg, 0.15 mmol, 1.0 equiv) and enyne **4a** (0.5 mL from a stock solution of 0.3 M) in THF (3.0 mL, 0.05 M). The reaction flask was lowered into the preheated oil bath (80 °C). After 22 h, the PKR yield (99%), b.r.s.m. (99%) and starting material remaining (0%) were determined based on the integral comparison of the product peak (d, 4.62 ppm, 1H) and starting material peak (s, 4.25 ppm, 2 H) to the internal standard mesitylene peak (s, 6.78 ppm, 3 H) via ^{1}H NMR. The crude was purified by SiO_2 chromatography eluting with 30% ethyl acetate/hexanes for HPLC analysis to get an *er* of 95:5.

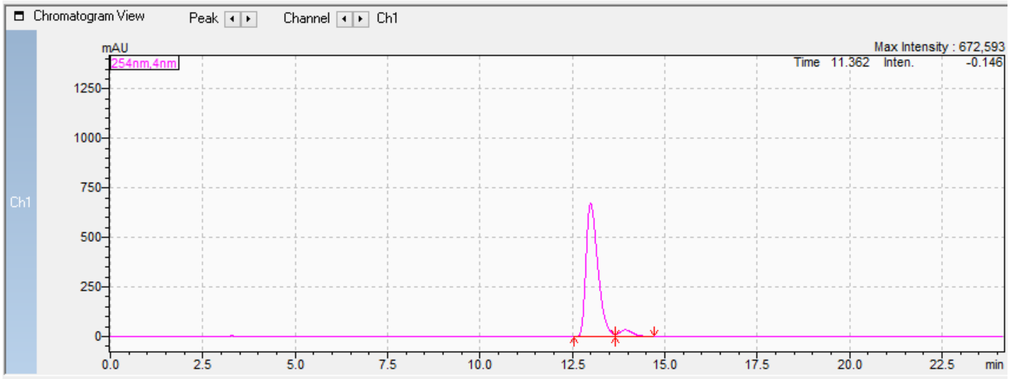

Results View - Peak Table

Peak#	Ret. Time	Area	Height	Mark	Area%
1	12.997	15118127	672911	M	94.861
2	13.939	818951	33633	V M	5.139
Total		15937078	706544		100.000

%*ee* = 94.861 – 5.139 = 89.722% = 90%

Entry 3: Follows General Procedure **D** for asymmetric PKR: Rh(cod)$_2$SbF$_6$ (8.3 mg, 0.015 mmol, 0.1 equiv), (*R*)-BINAP (10.3 mg, 0.0165 mmol, 0.11 equiv), mesitylene (18.0 mg, 0.15 mmol, 1.0 equiv) and enyne **4a** (0.5 mL from a stock solution of 0.3 M) in THF (3.0 mL, 0.05 M). The reaction flask was lowered into the preheated oil bath (80 °C). After 36 h, the PKR yield (91%), b.r.s.m. (93%) and starting material remaining (2%) were determined based on the integral comparison of the product peak (d, 4.62 ppm, 1H) and starting material peak (s, 4.25 ppm, 2 H) to the internal standard mesitylene peak (s, 6.78 ppm, 3 H) via ^{1}H NMR. The crude was purified by SiO$_2$ chromatography eluting with 30% ethyl acetate/hexanes for HPLC analysis to get an *er* of 89:11.

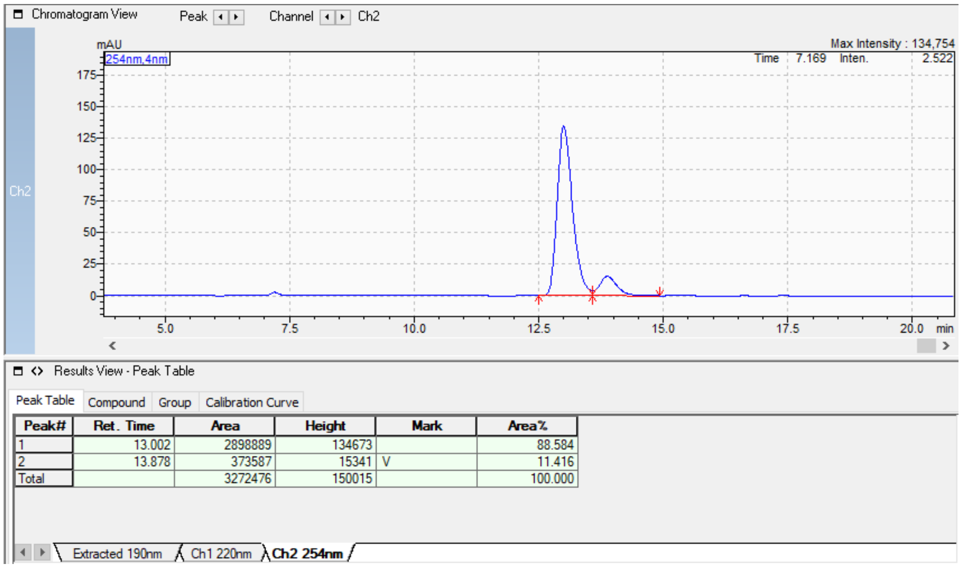

Peak#	Ret. Time	Area	Height	Mark	Area%
1	13.002	2898889	134673		88.584
2	13.878	373587	15341	V	11.416
Total		3272476	150015		100.000

%*ee* = 88.584-11.416 = 77.168 = 77%

Entry 4: Follows a modified version of General Procedure **D** for asymmetric PKR.[41] [Rh(CO)$_2$Cl]$_2$ (5.2 mg, 0.0134 mmol) and (*R*)-BINAP (24.8 mg, 0.0398 mmol) were each weighed into a separate 10-mL round-bottomed flask in a nitrogen-filled glovebox. To the flask containing [Rh(CO)$_2$Cl]$_2$ was added 2.2 mL of the prepared THF/mesitylene solution and a portion of this solution (1.25 mL) was transferred to another 10 mL flask. To the flask containing (*R*)-BINAP was added 3.0 mL of the prepared THF/mesitylene solution and a portion of this solution (1.25 mL) was transferred to the same 10-mL flask. The solution (containing [Rh(CO)$_2$Cl]$_2$ (2.95 mg, 0.0076 mmol, 0.05 equiv), (*R*)-BINAP (10.3 mg, 0.0165 mmol, 0.11

equiv)) was maintained for 1 h at rt and transferred via a syringe to a 10-mL round-bottomed flask equipped with a reflux condenser and charged with AgOTf (5.8 mg, 0.0226 mmol, 0.15 equiv). The resulting suspension was maintained for 30 min at rt followed by the replacement of argon atmosphere with 10% CO. The stock solution of 1,6-enyne **4a** (0.5 mL, 0.3 M) was added via a syringe to make the final volume of 3 mL (0.05 M, containing 0.15 mmol of mesitylene). The flask was lowered into the preheated oil bath (80 °C) and maintained. After 64 h, the PKR yield (39%), b.r.s.m. (62%) and starting material remaining (37%) were determined based on the integral comparison of the product peak (d, 4.62 ppm, 1H) and starting material peak (s, 4.25 ppm, 2 H) to the internal standard mesitylene peak (s, 6.78 ppm, 3 H) via ^{1}H NMR. The crude was purified by SiO_2 chromatography eluting with 30% ethyl acetate/hexanes for HPLC analysis to get an *er* of 93:7.

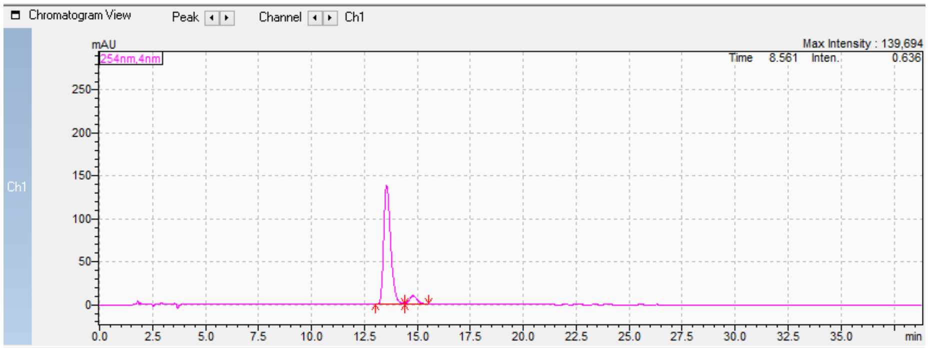

Results View - Peak Table

Peak Table | Compound | Group | Calibration Curve

Peak#	Ret. Time	Area	Height	Mark	Area%
1	13.548	3208014	139187		92.666
2	14.792	253911	10018	V	7.334
Total		3461926	149205		100.000

Extracted 190nm | Ch1 254nm

%*ee* = 92.666-7.334 = 85.332= 85%

Entry 5: Follows a modified version of General Procedure **D** for asymmetric PKR.[5] $[Rh(cod)Cl]_2$ (3.7 mg, 0.0075 mmol, 0.05 equiv), (*R*)-BINAP (10.3 mg, 0.0165 mmol, 0.11 equiv) and AgOTf (5.8 mg, 0.0225 mmol, 0.15 equiv) were each weighed into a 10-mL round-bottomed flask equipped with a reflux condenser in a nitrogen-filled glovebox. The flask was removed from the glovebox and 2.5 mL of the prepared THF/mesitylene solution was added. The mixture was maintained for 1 h at 60 °C to generate the cationic Rh *in situ*. The flask was removed from the oil bath and the argon atmosphere was replaced with 10% CO. The stock solution of 1,6-enyne **4a** (0.5 mL, 0.3 M) was added via a syringe to make the final volume of 3 mL (0.05 M, containing 0.15 mmol of mesitylene). The flask was lowered into the preheated oil bath (80 °C) and maintained. After 22 h, no significant progress could be observed based on ^{1}H NMR. The PKR yield

(80%), b.r.s.m. (100%) and starting material remaining (20%) were determined based on the integral comparison of the product peak (d, 4.62 ppm, 1 H) and starting material peak (s, 4.25 ppm, 2 H) to the internal standard mesitylene peak (s, 6.78 ppm, 3 H) via ^{1}H NMR. The crude was purified by SiO_2 chromatography eluting with 30% ethyl acetate/hexanes for HPLC analysis to get an *er* of 96:4.

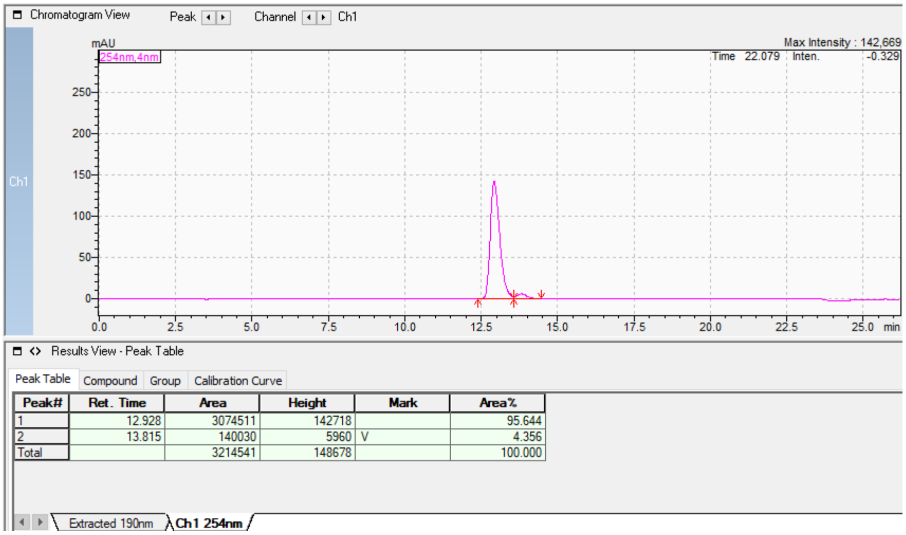

Peak#	Ret. Time	Area	Height	Mark	Area%
1	12.928	3074511	142718		95.644
2	13.815	140030	5960	V	4.356
Total		3214541	148678		100.000

%*ee* = 95.644-4.356 = 91.288=91%

Proposed Mechanisms for the Cycloisomerization and PKR

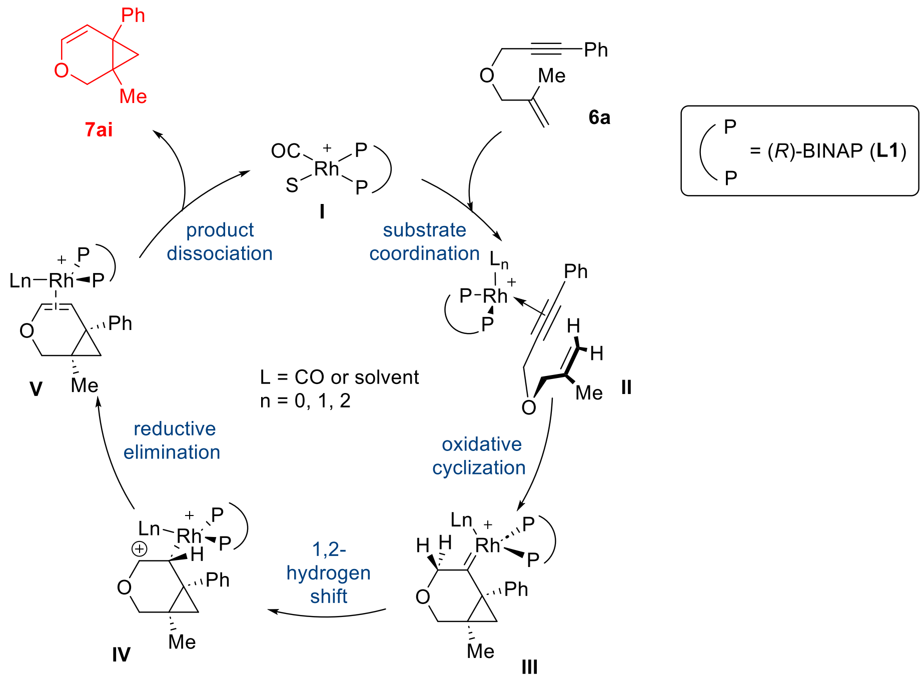

Figure S52. Pathway A for enyne cycloisomerization. Proposed mechanism based upon $PtCl_2$ catalyzed reaction.[42]

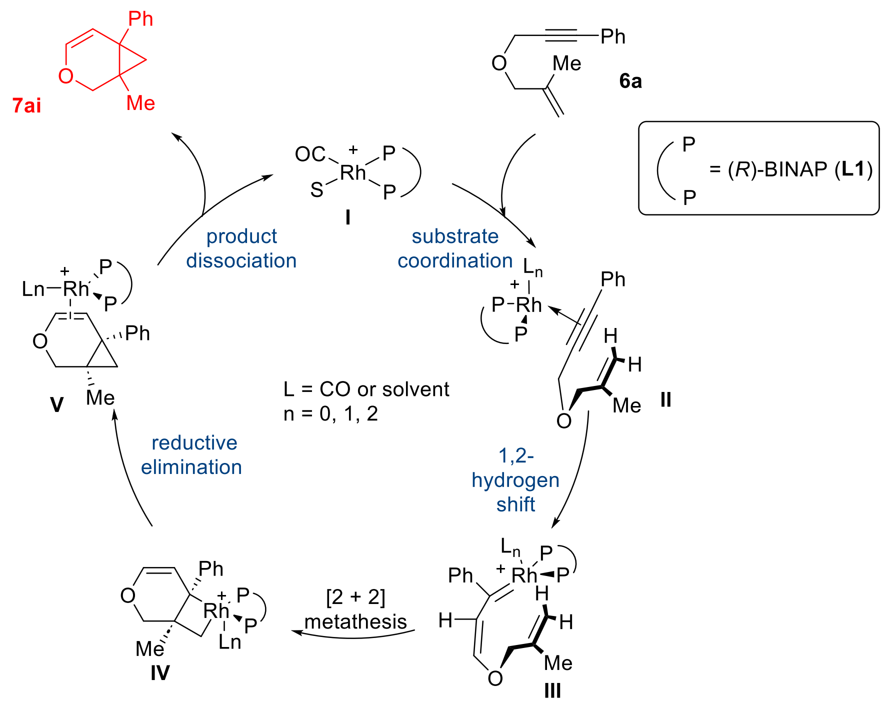

Figure S53. Pathway B for enyne cycloisomerization.

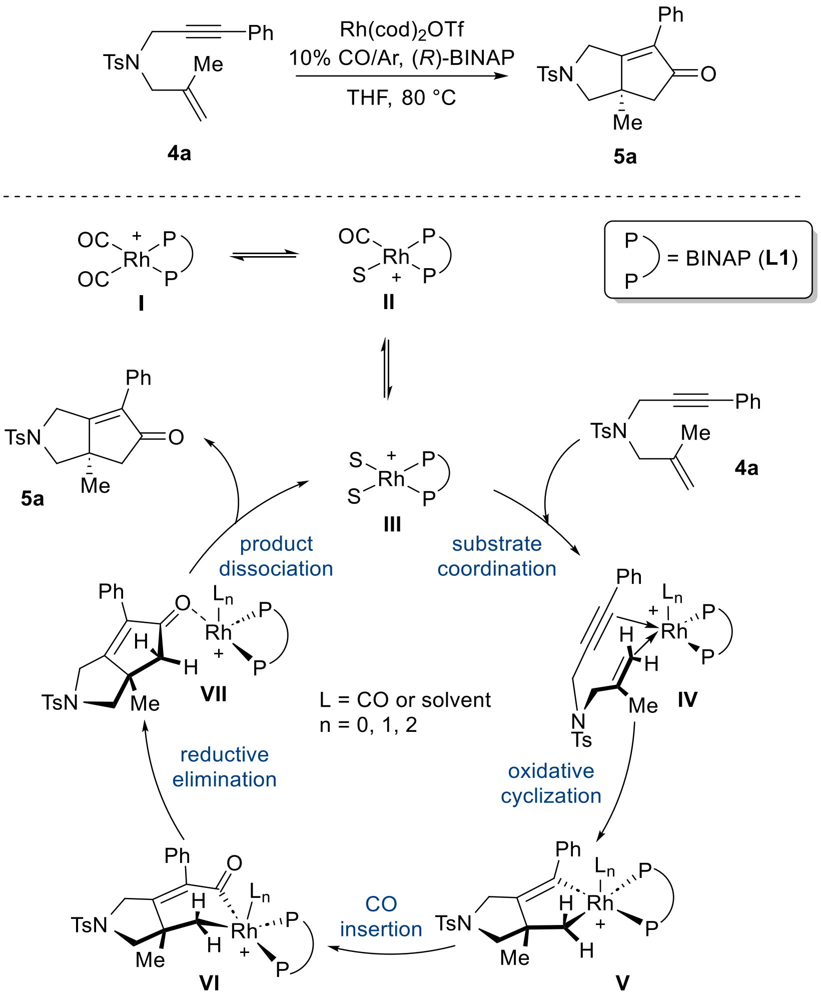

Figure S54. Generally accepted mechanism of asymmetric PKR.

Substrate Study–Alkynyl group

Table S8. Steric parameters for groups on alkyne of enyne

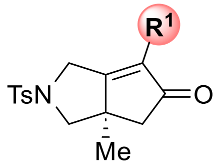

**R^1**	**Ph<br>5a**	**CO_2Me<br>5c**	**Me<br>5b**	**H<br>5d**
$\Delta\Delta G^\ddagger$ kcal/mol[a]	0.26	1.53	2.2	4.19
yield%[b]	99	64	71	34
ee %[b]	89.7	92.5	93.8	99.6
Sterimol B_1[c]	3.53	3.24	3.16	2.06
Sterimol B_5[c]	6.75	6.48	6.35	7.55
Sterimol L[c]	10.57	10.38	10.81	11.71
A value kcal/mol[43]	3.0	1.1	1.8	–

[a] Computed Gibbs free energy of activation differences for diastereomeric transition states ($\Delta\Delta G^\ddagger$) for the oxidative cyclization step using **4a**, **4b**, **4c**, **4d** and (*S*)-Xyl-SEGPHOS by Baik *et al.*[5] ;[b]Yield and *ee*% of product **5a**, **5b**, **5c**, **5d** from our work using (*R*)-BINAP; [c] Sterimol values were calculated using PK product **5a**, **5b**, **5c**, **5d** (see below)

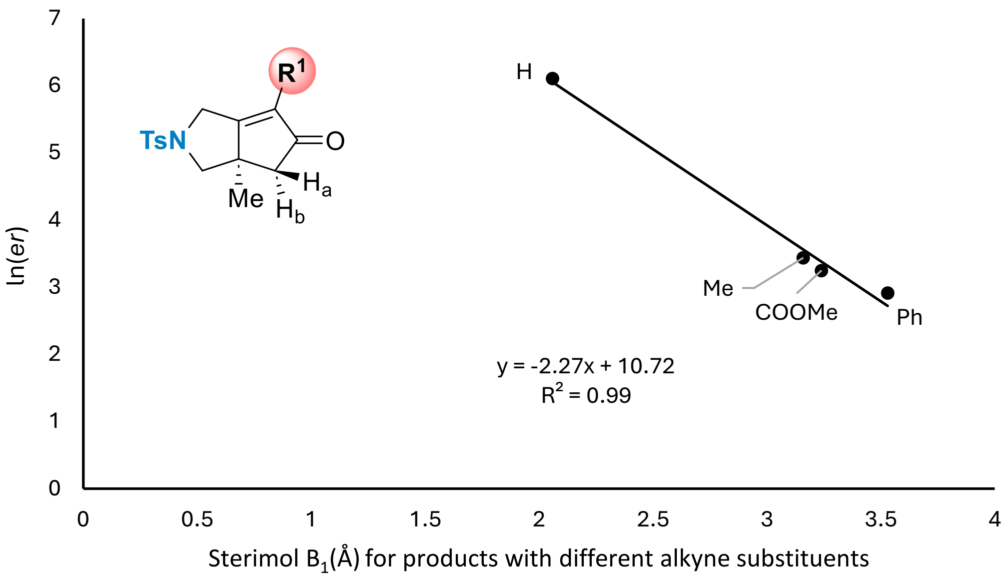

Figure S55. Sterimol B_1(Å) values (C-H_b from product **5a**, **5b**, **5c** and **5d** as the primary axis) plotted against ln(*er*) of **5a** (R^1=Ph), **5b** (R^1=Me), **5c** (R^1 = CO_2Me), and **5d** (R^1= H).

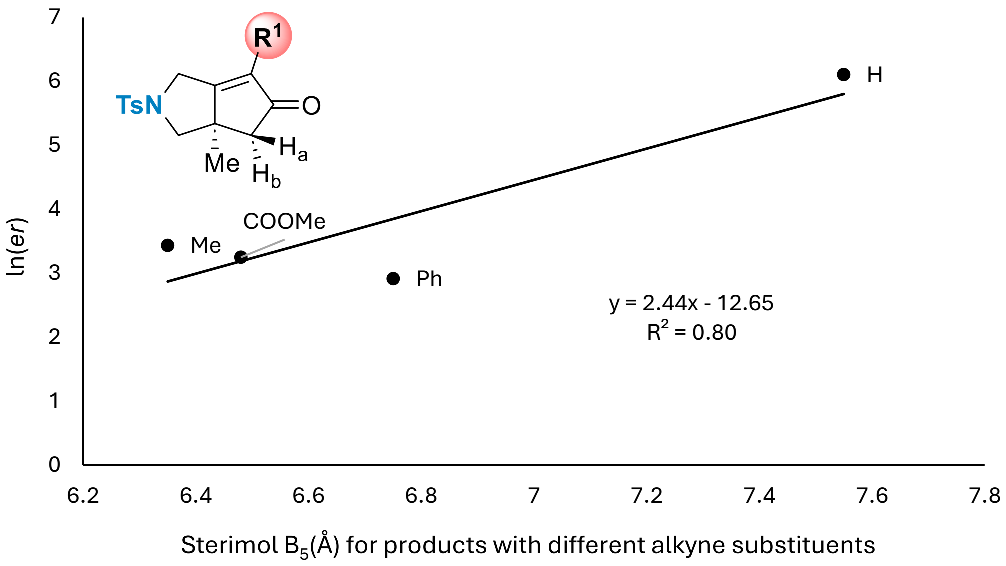

Figure S56. Sterimol B_5 (Å) values (C-H_b from product **5a**, **5b**, **5c** and **5d** as the primary axis) plotted against ln(*er*) of **5a** (R^1=Ph), **5b** (R^1=Me), **5c** (R^1 = CO_2Me), and **5d** (R^1= H).

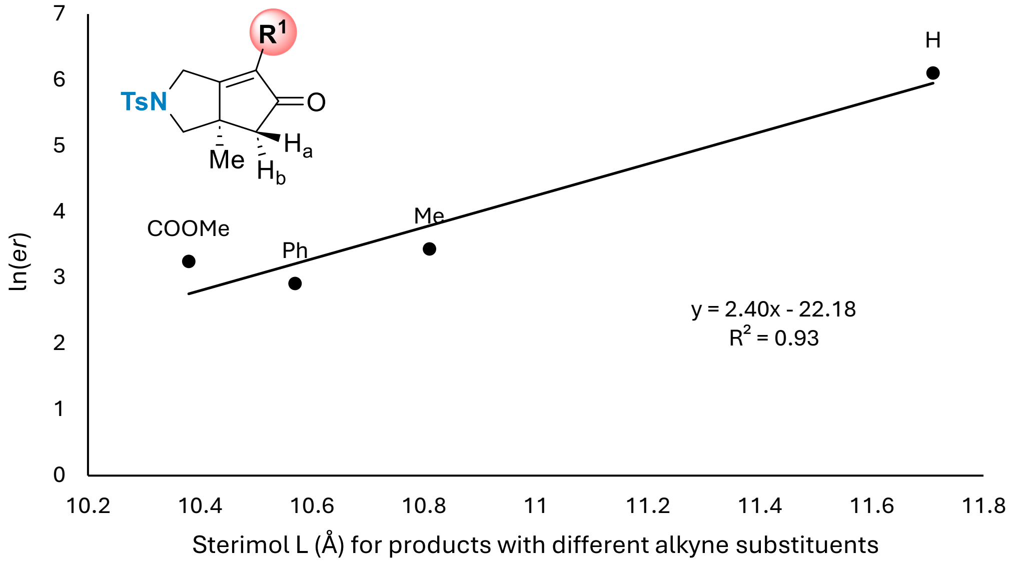

Figure S57. Sterimol L(Å) values (C-H_b from product **5a**, **5b**, **5c** and **5d** as the primary axis) plotted against ln(*er*) of **5a** (R^1=Ph), **5b** (R^1=Me), **5c** (R^1 = CO_2Me), and **5d** (R^1= H).

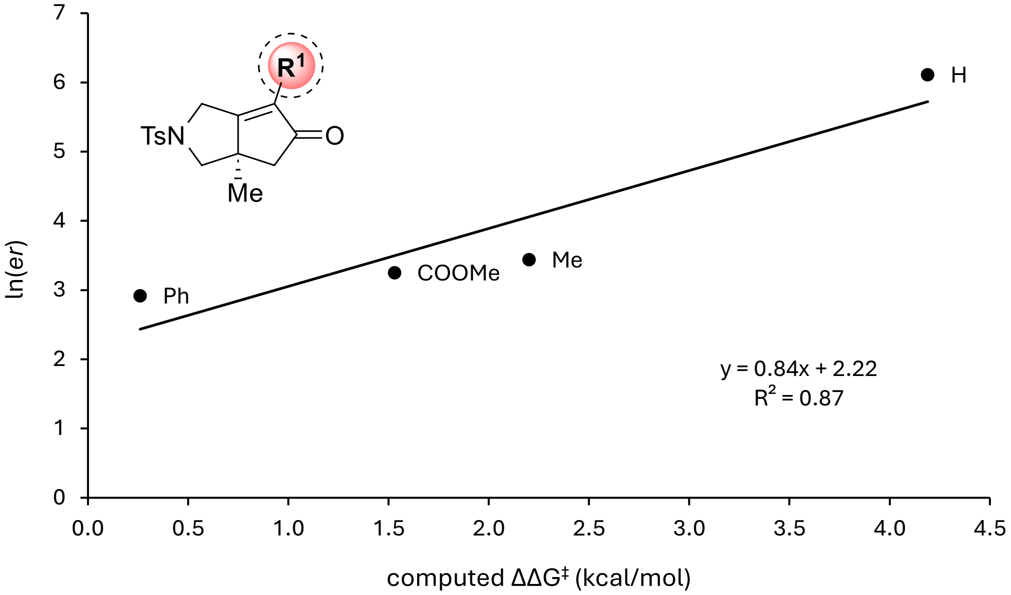

Figure S58. ln(*er*) of **5a**, **5b**, **5c** and **5d** plotted against computed $\Delta\Delta G^{\ddagger}$ ($\Delta\Delta G^{\ddagger}$ calculated by Baik et al. using (*S*)-Xyl-SEGPHOS).[5]

Substrate Study–Tether

Table S9. Comparison of the effect of oxygen, nitrogen and malonate tethers on the reactivity of the PKR

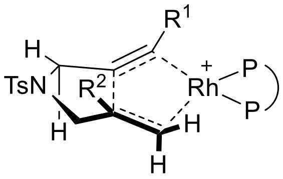

TS-SA

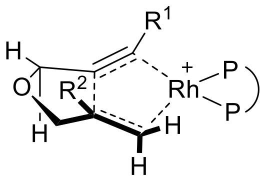

TS-SB

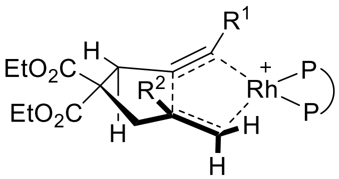

TS-SC

$\Delta G^{\ddagger}$ (kcal/mol)[a]	12.8	11.78	15.34[b]
temperature (°C)[c]	80	85	95
time (h)[c]	22	23	20
yield (%)[c]	99	84	94
er[c]	95:5	94:6	90:10

[a]Previously computed activation energy by Baik *et al*. using (*S*)-Xyl-SEGPHOS,[5] R^1 = Cl, R^2 = ^{i}Pr;[b]calculated $\Delta G^{\ddagger}$ using the tether $C(CO_2Me)_2$ by Baik *et al*.[5];[c] Our work with (*R*)-BINAP, R^1 = Ph, R^2 = Me

Calculated IR C=C and C≡C bond wavenumbers (cm^{-1}), intensities, and dipole moments for selected enyne precursors (EDF2/6-31G* corrected values reported, Spartan'20)

Table S10. IR C=C and C≡C bond wavenumbers (cm^{-1}), intensities, and dipole moments (D) for enyne precursors featuring different tether and alkyne substituents (EDF2/6-31G* corrected values reported, Spartan'20)

(a)

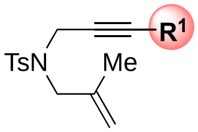

R^1 =	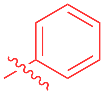	Me	H	TMS	Cl
	**4a**	**4b**	**4d**	**4e**	**S17**
IR C=C (cm^{-1})	1680 (1658)[a]	1679	1680 (1656)[a]	1680 (1656)[a]	1680
IR C=C intensity	8.57	9.37	9.3	8.81	9.1
IR C≡C (cm^{-1})	2257 (2243)[a]	2284	2156 (2120)[a]	2200 (2177)[a]	2262
IR C≡C intensity	0.87	9.08	0.69	28.28	47.27
dipole moment (D)	5.47	5.76	5.04	5.2	4.46

(b)

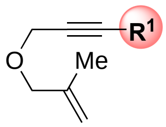

R1 = 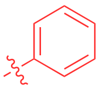 Me H TMS Cl

	6a	6b	S18	S19	S20
IR C=C (cm^{-1})	1679 (1657)[a]	1677 (1656)[a]	1678	1679	1677
IR C=C intensity	6.66	7.11	7.18	7.01	6.88
IR C≡C (cm^{-1})	2254 (2239)[a]	2283 (2223)[a]	2155	2198	2261
IR C≡C intensity	4.01	14.47	1.84	28.07	64.21
dipole moment (D)	1.1	1.29	0.98	0.96	1.17

(c)

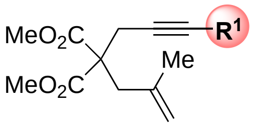

R1 = 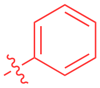 Me H TMS Cl

	S21	S22	S23	S24	S25
IR C=C (cm^{-1})	1671 (1645)[a,b]	1671	1671 (1646)[a,b]	1671	1671
IR C=C intensity	10.41	11.47	11.79	10.62	11.35
IR C≡C (cm^{-1})	2261 (2244)[a,b]	2288	2161 (2123)[a,b]	2203	2269
IR C≡C intensity	1.44	2.34	0.83	71.78	32.22
dipole moment (D)	1.85	1.46	1.95	1.83	2.54

[a]experimentally determined IR;[b]IR measurement was taken on precursor **8a** or **8c**

Hammett parameters (σ_p) and ether-tethered PKR products 7a, 7c, 7d and 7e

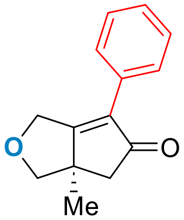

7a

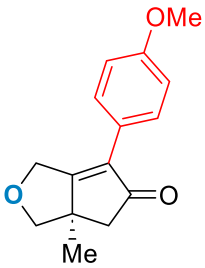

7c

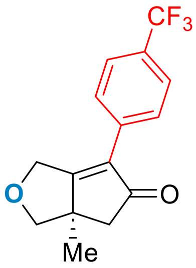

7d

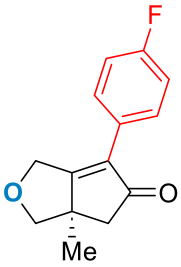

7e

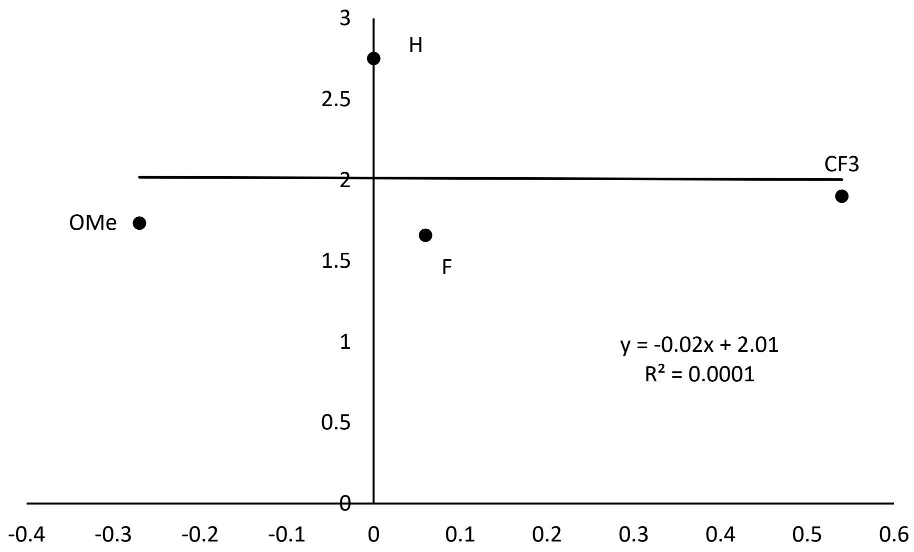

Figure S59. Hammett parameters (σ_p) plotted against ln(*er*) of products **7a**, **7c**, **7d** and **7e**.[44]

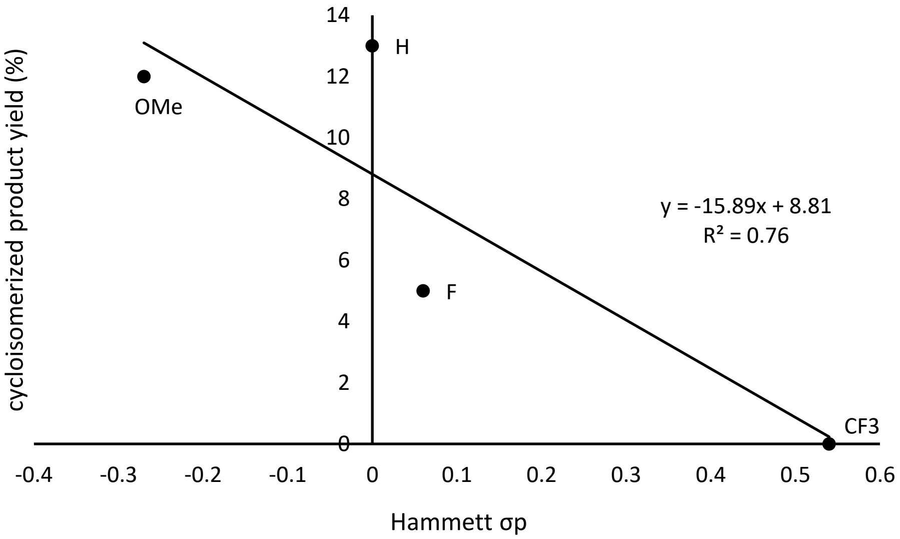

Figure S60. Hammett parameters (σ_p) plotted against yields of the cycloisomerized products **7ai**, **7ci**, **7di** and **7ei**.[44]

Spectral Data for Solvent Study Using Rh(cod)$_2$OTf and NTs-Tethered Precursor 4a

Unless indicated otherwise, the reactions for the solvent study were performed following **General Procedure D** for the asymmetric Pauson-Khand reactions. An initial timepoint was taken to determine the enyne **4a** or **6a** concentration relative to mesitylene. A second timepoint was taken at 24 h to determine the yield of PK product **5a** or **7a**, yield based on recovered starting material (b.r.s.m.), starting material **4a** or **6a** remaining, yield of the cycloisomerized side product **5ai** or **7ai**, and %*ee* of PK product **5a** or **7a** (purified by prep TLC and *ee*% determined by HPLC).

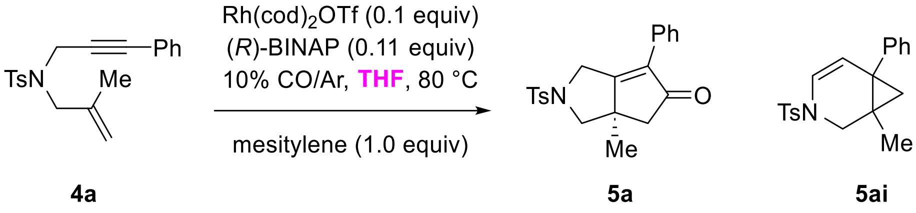

Run 1-Follows general procedure D. Rh(cod)$_2$OTf (7.1 mg, 0.015 mmol, 0.1 equiv), (*R*)-BINAP (10.3 mg, 0.0165 mmol, 0.11 equiv), mesitylene (18.0 mg, 0.15 mmol, 1.0 equiv) and enyne **4a** (0.5 mL from a stock solution of 0.3 M) in THF (3.0 mL, 0.05 M). After 22 h, PKR yield (99%), b.r.s.m. (99%), starting material remaining (0%) and cycloisomerized side product (0%) were determined by integral comparison to the internal standard mesitylene.

run 1:

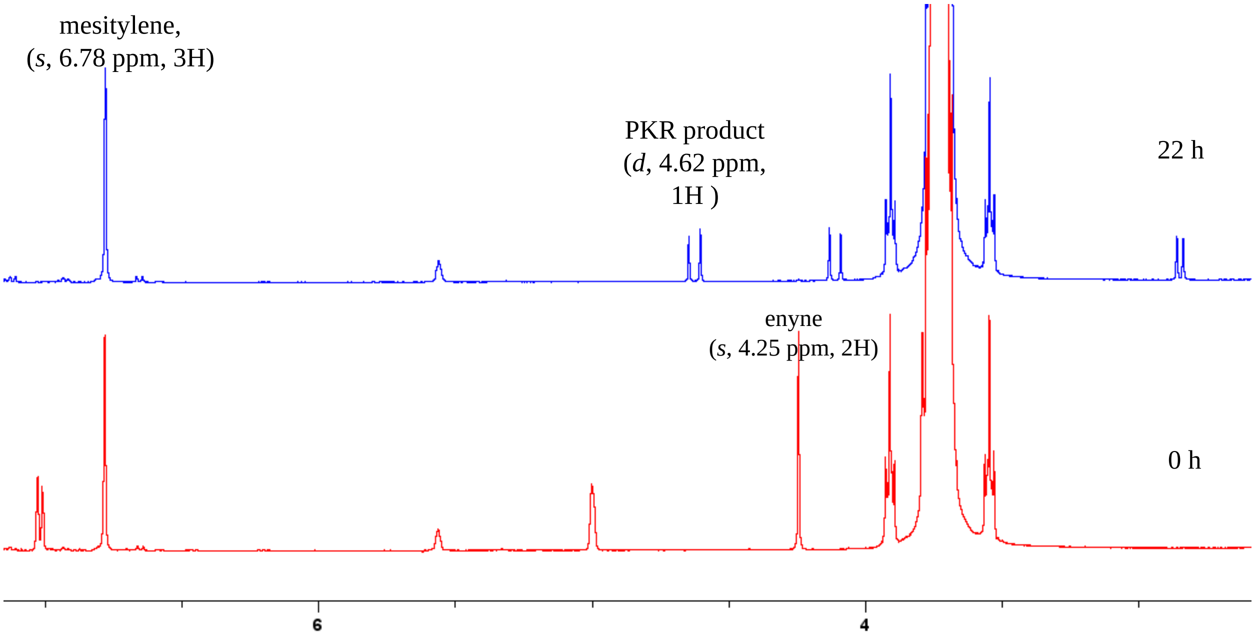

	mesitylene integration (*s*, 6.78 ppm, 3 H)	enyne integration(*s*, 4.25 ppm, 2 H)	PKR product integration (*d*, 4.62 ppm, 1H)	Cycloisomerized product integration (*d*, 5.34 ppm, 1H)
0 h	1.0000	0.6758	0.0000	0.0000
22 h	1.0000	trace	0.3308	0.0000

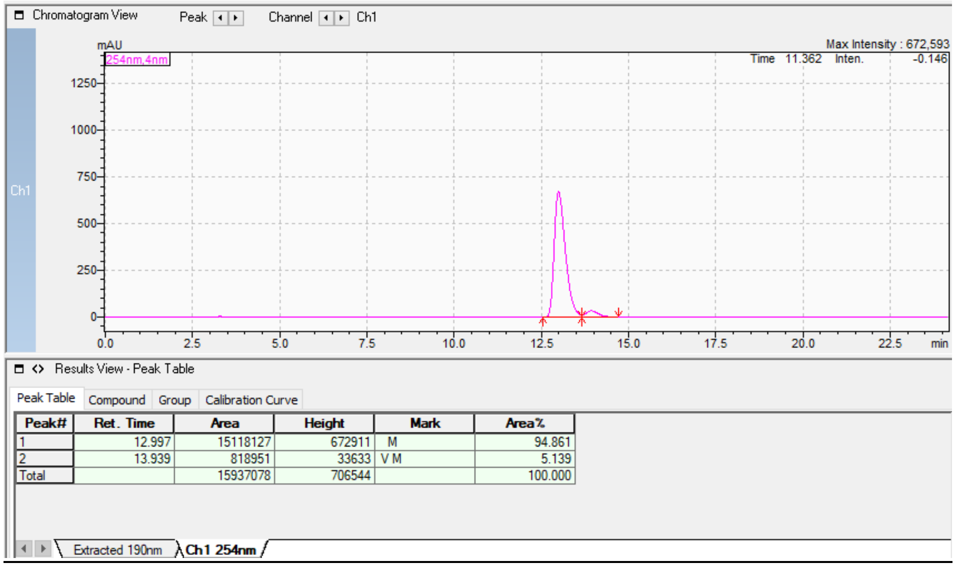

%*ee* (run 1) = 94.861-5.139 = 89.722 = 90%

Run 2-Follows general procedure D. Rh(cod)$_2$OTf (7.1 mg, 0.015 mmol, 0.1 equiv), (*R*)-BINAP (10.3 mg, 0.0165 mmol, 0.11 equiv), mesitylene (18.0 mg, 0.15 mmol, 1.0 equiv) and enyne **4a** (0.5 mL from a stock solution of 0.3 M) in THF (3.0 mL, 0.05 M). After 24 h, PKR yield (98%), b.r.s.m. (99%), starting material remaining (0%) and cycloisomerized side product (0%) were determined by integral comparison to the internal standard mesitylene.

run 2:

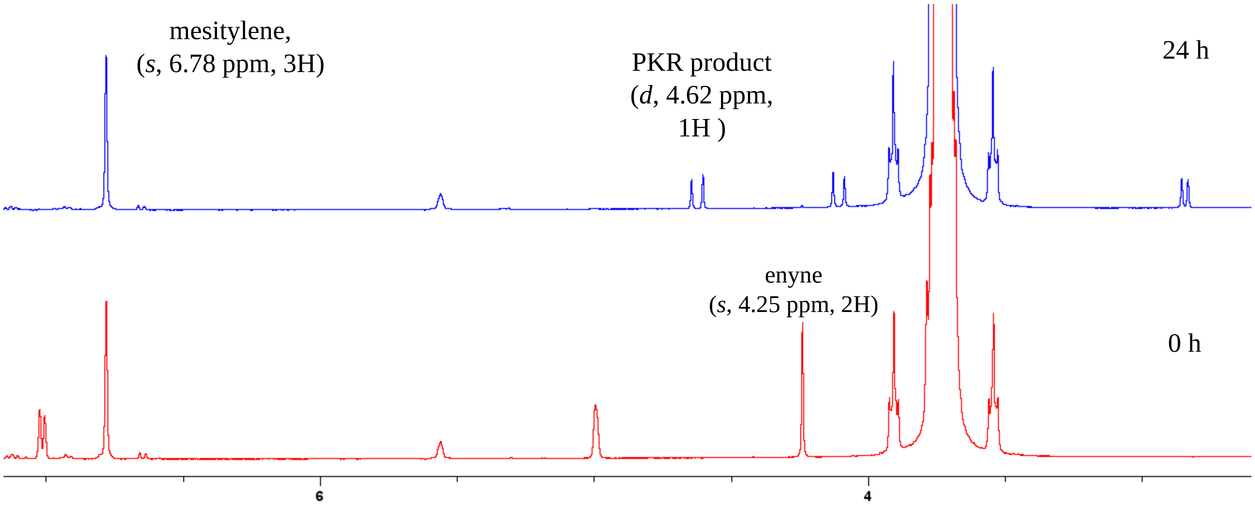

	mesitylene integration (*s*, 6.78 ppm, 3H)	enyne integration(*s*, 4.25 ppm, 2H)	PKR product integration (*d*, 4.62 ppm, 1H)	Cycloisomerized product integration (*d*, 5.34 ppm, 1H)
0 h	1.0000	0.6708	0.0000	0.0000
24 h	1.0000	0.0000	0.3257	0.0000

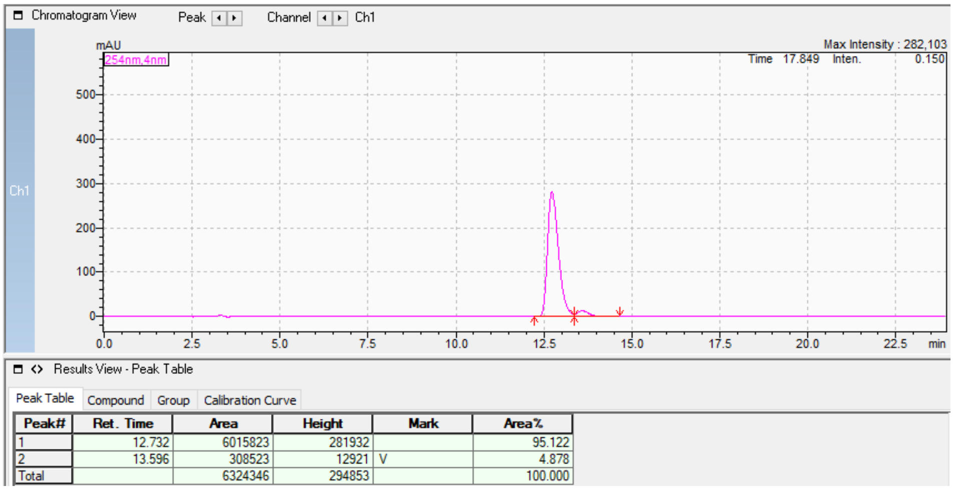

Peak#	Ret. Time	Area	Height	Mark	Area%
1	12.732	6015823	281932		95.122
2	13.596	308523	12921	V	4.878
Total		6324346	294853		100.000

%*ee* (run 2) = 95.122 - 4.878 = 90.244%

%*ee* (average)= (89.722 + 90.244)/2 = 89.983 = 90%

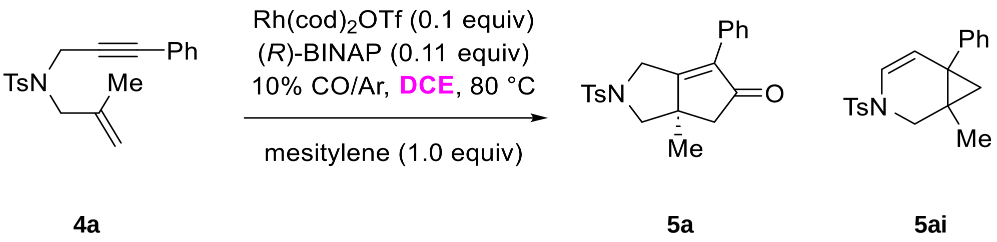

Run 1-Follows general procedure D. Rh(cod)$_2$OTf (7.1 mg, 0.015 mmol, 0.1 equiv), (*R*)-BINAP (10.3 mg, 0.0165 mmol, 0.11 equiv), mesitylene (18.0 mg, 0.15 mmol, 1.0 equiv) and enyne **4a** (0.5 mL from a stock solution of 0.3 M) in DCE (3.0 mL, 0.05 M). After 27 h, PKR yield (72%), b.r.s.m. (92%), starting material remaining (22%) and cycloisomerized side product (7%) were determined by integral comparison to the internal standard mesitylene.

run 1:

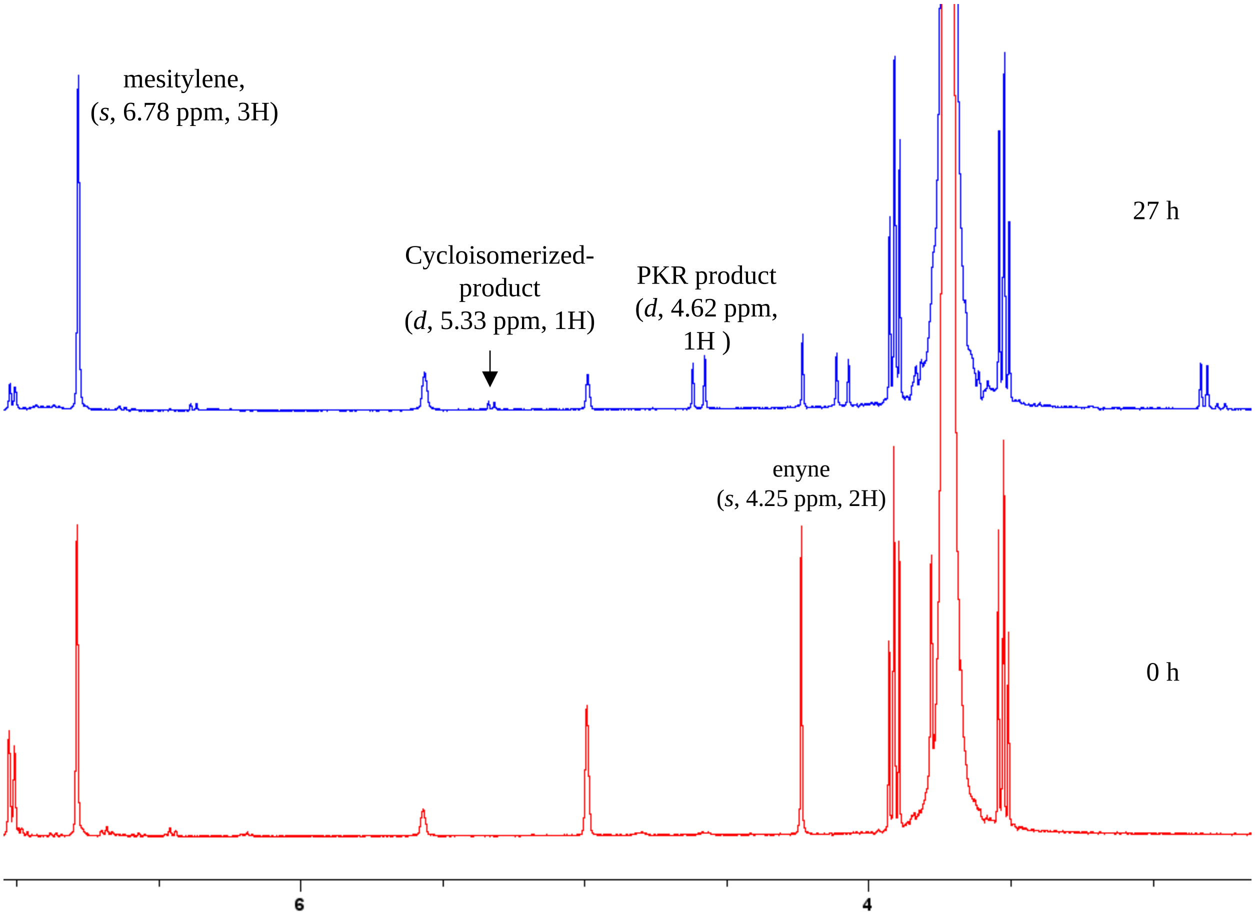

	mesitylene integration (*s*, 6.78 ppm, 3H)	enyne integration(*s*, 4.25 ppm, 2H)	PKR product integration (*d*, 4.62 ppm, 1H)	Cycloisomerized product integration (d, 5.33 ppm, 1H)

0 h	1.0000	0.6653	0.0000	0.0000
27 h	1.0000	0.1470	0.2388	0.0240

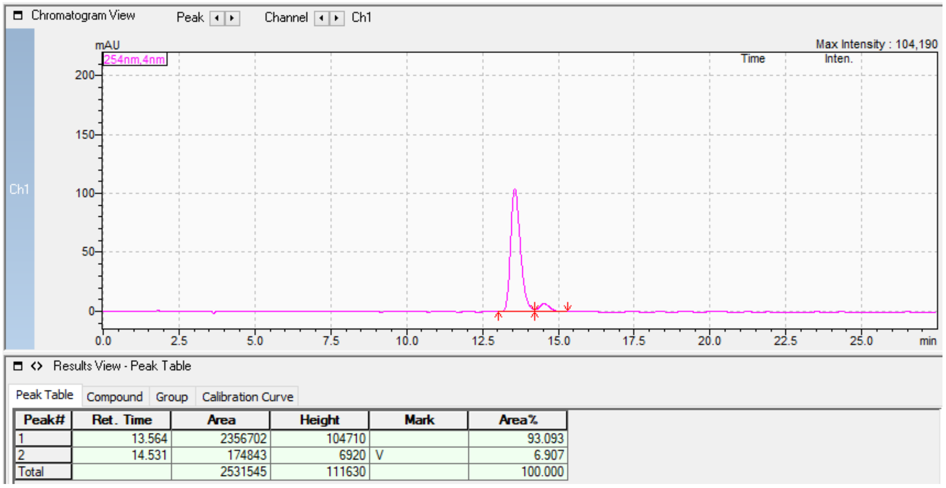

Peak#	Ret. Time	Area	Height	Mark	Area%
1	13.564	2356702	104710		93.093
2	14.531	174843	6920	V	6.907
Total		2531545	111630		100.000

%*ee* (run 1) = 93.093 – 6.907 = 86.186%

Run 2-Follows general procedure D. Rh(cod)$_2$OTf (7.1 mg, 0.015 mmol, 0.1 equiv), (*R*)-BINAP (10.3 mg, 0.0165 mmol, 0.11 equiv), mesitylene (18.0 mg, 0.15 mmol, 1.0 equiv) and enyne **4a** (0.5 mL from a stock solution of 0.3 M) in DCE (3.0 mL, 0.05 M). After 24 h, PKR yield (82%), b.r.s.m. (94%), starting material remaining (13%) and cycloisomerized side product (5%) were determined by integral comparison to the internal standard mesitylene.

run 2:

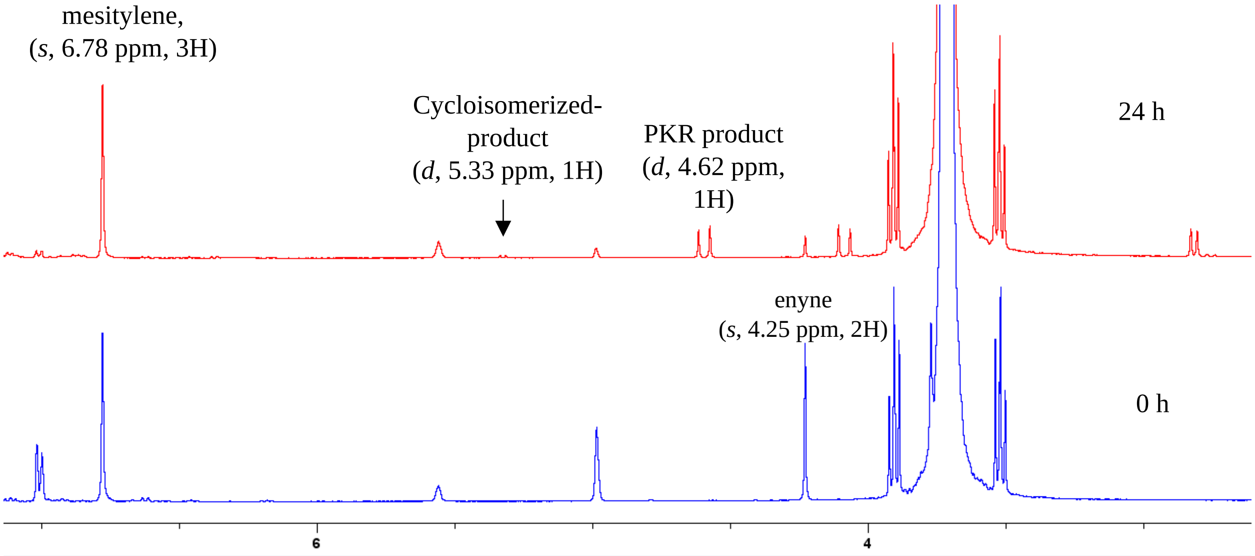

	mesitylene integration (*s*, 6.78 ppm, 3H)	enyne integration(*s*, 4.25 ppm, 2H)	PKR product integration (*d*, 4.62 ppm, 1H)	Cycloisomerized product integration (*d*, 5.33 ppm, 1H)
0 h	1.0000	0.6463	0.0000	0.0000
24 h	1.0000	0.0871	0.2640	0.0160

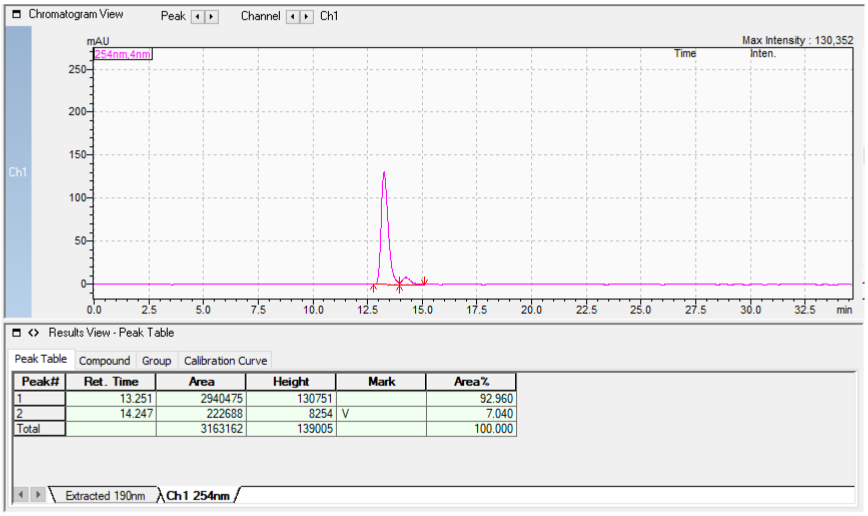

%*ee* (run 2) = 92.960 – 7.040 = 85.920 %

%*ee* (average) = (86.186 + 85.920)/2 = 86.053 = 86%

Characterization of the cycloisomerized side product 5ai (crude purified by SiO_2 chromatography eluting with 5% EtOAc/Hexanes to give the title compound as white solid)

^{1}H NMR (400 MHz, $CDCl_3$)

7.70 (d, *J* = 8.3 Hz, 2 H), 7.35 (d, *J* = 8.3 Hz, 2 H), 7.28-7.12 (m , 5 H), 6.40 (dd, *J* = 8.0, 0.75 Hz, 1 H), 5.35 (d, *J* = 8.0 Hz, 1 H), 3.94 (d, *J* = 11.6 Hz, 1 H), 2.78 (d, *J* = 11.6 Hz, 1 H), 2.45 (s, 3 H), 1.22 (d, *J* = 4.9 Hz, 1 H), 1.03 (d, *J* = 4.9 Hz, 1 H), 0.79 (s, 3 H)

^{13}C NMR (100.6 MHz, $CDCl_3$)

143.9, 141.4, 135.1, 130.0 (2C), 129.2 (2C), 128.3 (2C), 127.2 (2C), 126.5, 120.7, 117.4, 46.6, 32.7, 28.4, 23.5, 21.7, 19.0

IR

1635, 1344, 1162 cm^{-1}

mp

125–129 °C

HRMS

HRMS-ESI (m/z): $[M + H]^+$ calcd for $C_{20}H_{22}NO_2S$, 340.1366; found: 340.1357

TLC

R_f = 0.25 (5% ethyl acetate/hexanes) [silica gel, *p*-anisaldehyde stain]

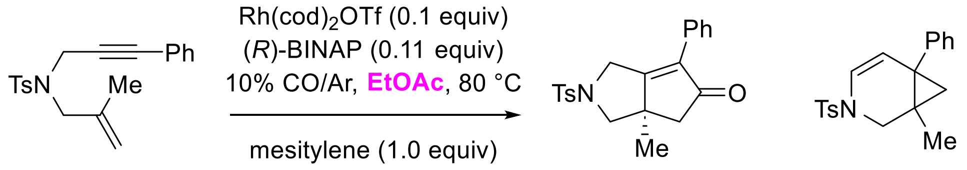

4a **5a** **5ai**

Run 1-Follows general procedure D. Rh(cod)$_2$OTf (7.1 mg, 0.015 mmol, 0.1 equiv), (*R*)-BINAP (10.3 mg, 0.0165 mmol, 0.11 equiv), mesitylene (18.0 mg, 0.15 mmol, 1.0 equiv) and enyne **4a** (0.5 mL from a stock solution of 0.3 M) in EtOAc (3.0 mL, 0.05 M). After 24 h, PKR yield (25%), b.r.s.m. (96%), starting material remaining (74%) and cycloisomerized side product (trace) were determined by integral comparison to the internal standard mesitylene.

run 1:

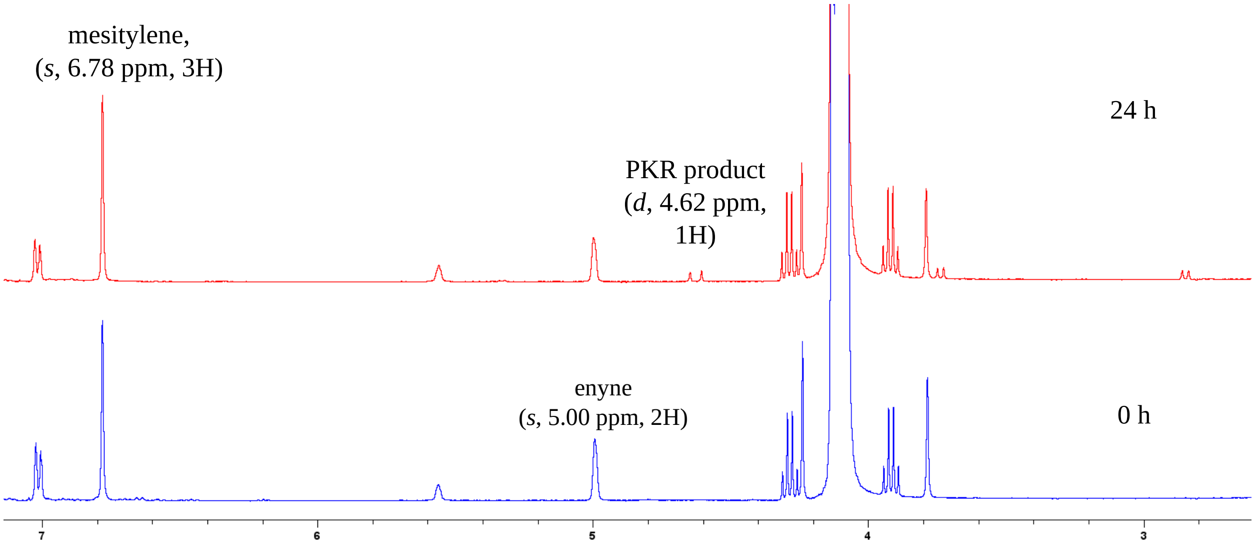

	mesitylene integration (*s*, 6.78 ppm, 3H)	enyne integration(*s*, 5.00 ppm, 2H)	PKR product integration (*d*, 4.62 ppm, 1H)	Cycloisomerized product integration (*d*, 5.33 ppm, 1H)
0 h	1.0000	0.6479	0.0000	0.0000
24 h	1.0000	0.4843	0.0820	trace

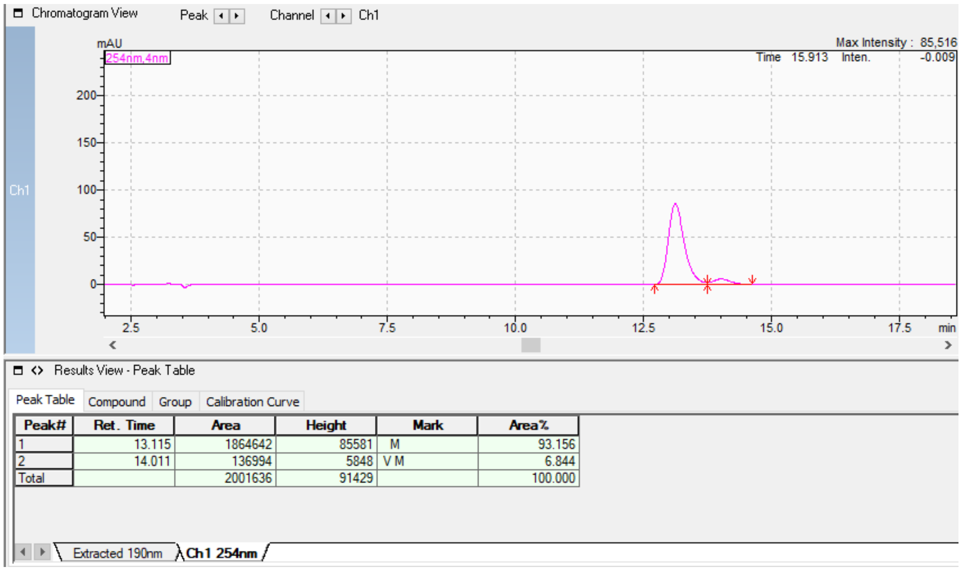

%*ee* (run 1) = 93.156 – 6.844 = 86.312%

Run 2-Follows general procedure D. Rh(cod)$_2$OTf (7.1 mg, 0.015 mmol, 0.1 equiv), (*R*)-BINAP (10.3 mg, 0.0165 mmol, 0.11 equiv), mesitylene (18.0 mg, 0.15 mmol, 1.0 equiv) and enyne **4a** (0.5 mL from a stock solution of 0.3 M) in EtOAc (3.0 mL, 0.05 M). After 24 h, PKR yield (21%), b.r.s.m. (91%), starting

material remaining (77%) and cycloisomerized side product (trace) were determined by integral comparison to the internal standard mesitylene.

run 2:

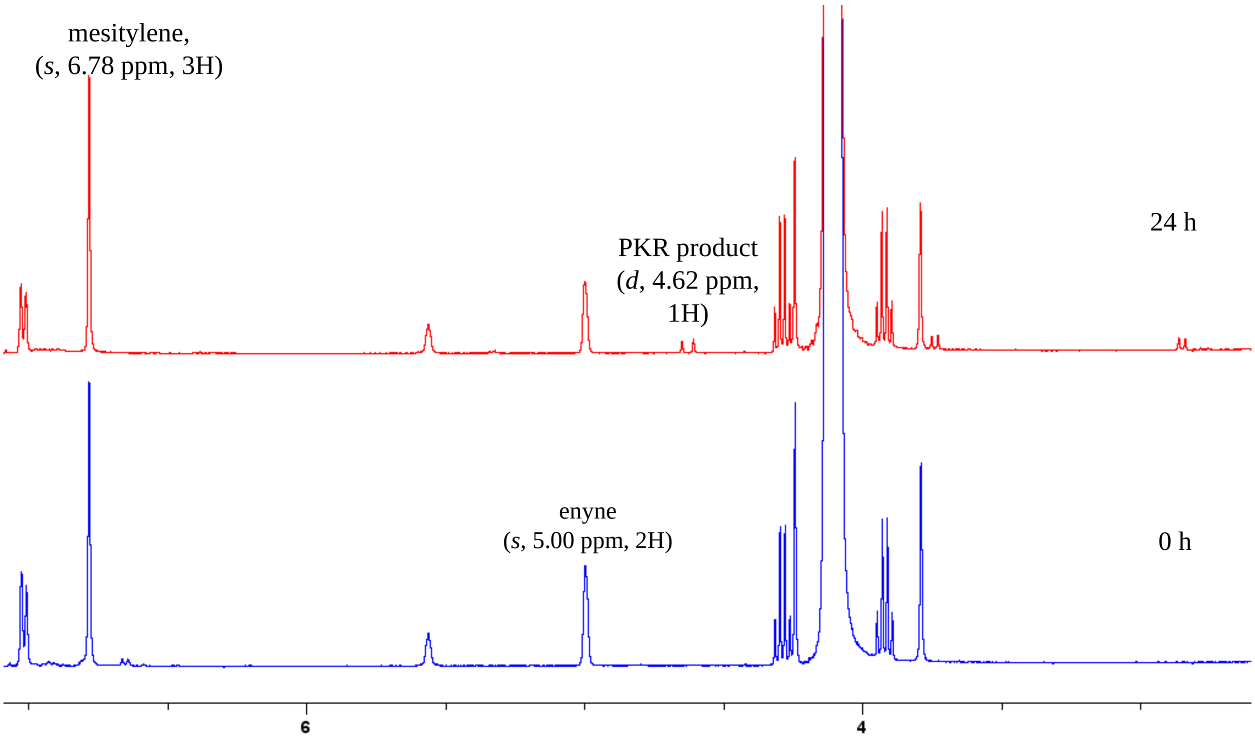

	mesitylene integration (*s*, 6.78 ppm, 3H)	enyne integration(*s*, 5.00 ppm, 2H)	PKR product integration (*d*, 4.62 ppm, 1H)	Cycloisomerized product integration (*d*, 5.33 ppm, 1H)
0 h	1.0000	0.6894	N/A	N/A
24 h	1.0000	0.5355	0.0725	trace

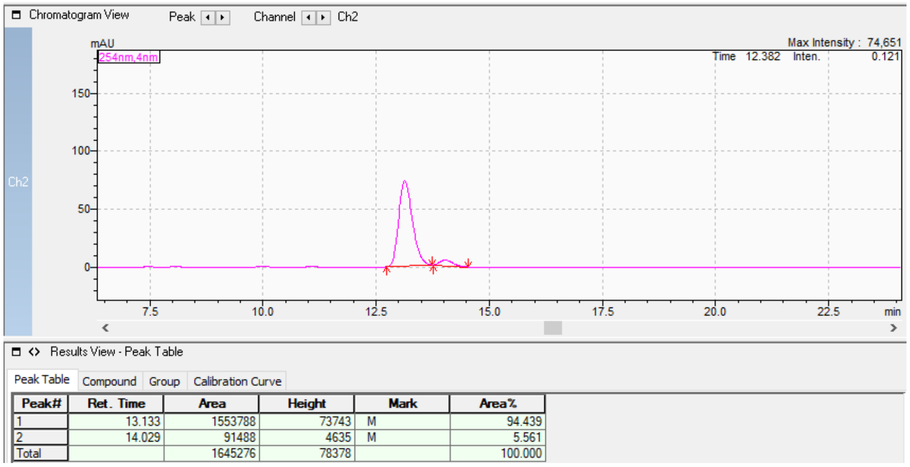

%*ee* (run 2) = 94.439 – 5.561 = 88.878 %

%*ee* (average) = (86.312 + 88.878)/2 = 87.595 = 88%

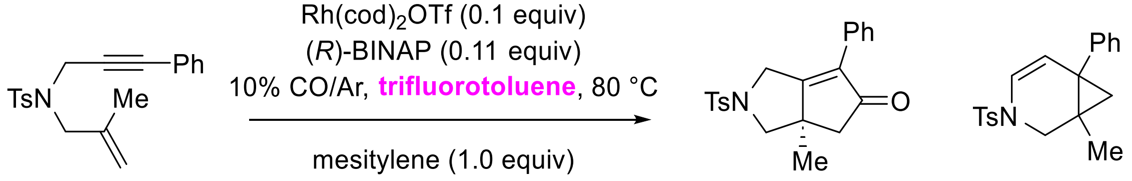

4a **5a** **5ai**

Run 1-Follows general procedure D. Rh(cod)$_2$OTf (7.1 mg, 0.015 mmol, 0.1 equiv), (*R*)-BINAP (10.3 mg, 0.0165 mmol, 0.11 equiv), mesitylene (18.0 mg, 0.15 mmol, 1.0 equiv) and enyne **4a** (0.5 mL from a stock solution of 0.3 M) in trifluorotoluene (3.0 mL, 0.05 M). After 24 h, PKR yield (46%), b.r.s.m. (81%), starting material remaining (43%) and cycloisomerized side product (trace) were determined by integral comparison to the internal standard mesitylene.

<u>run 1:</u>

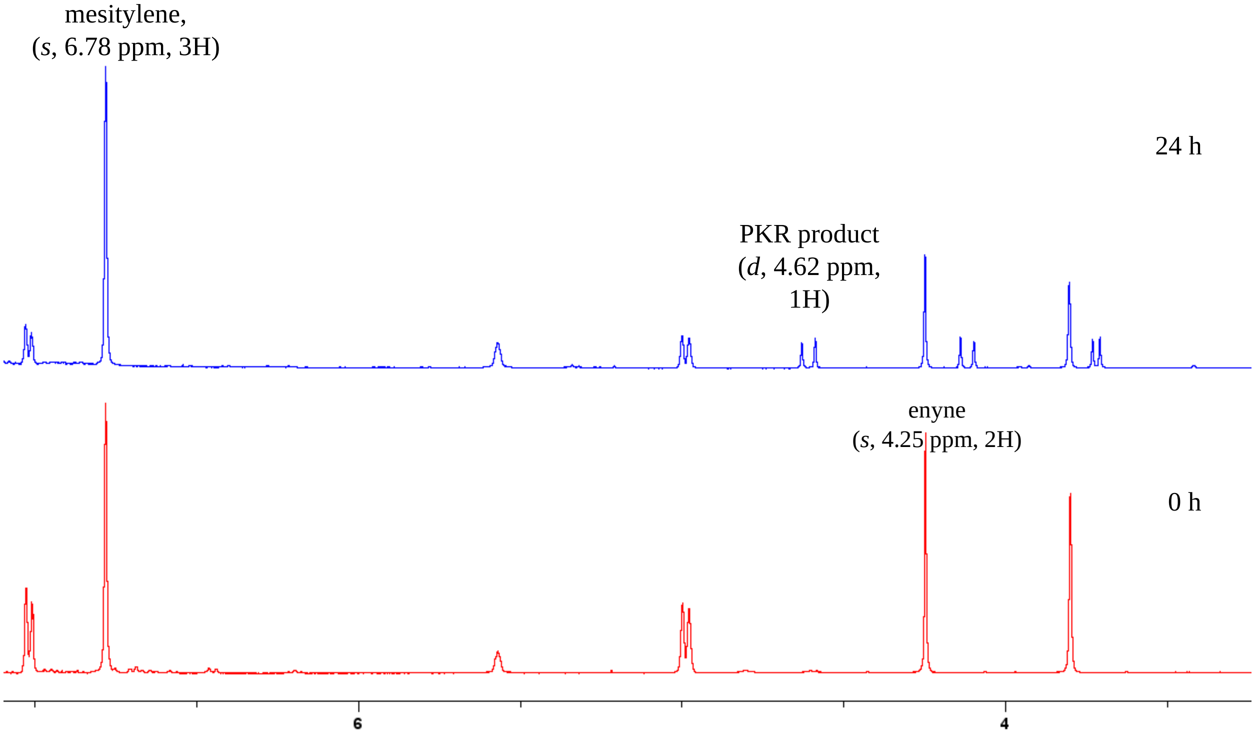

	mesitylene integration (*s*, 6.78 ppm, 3H)	enyne integration(*s*, 4.25 ppm, 2H)	PKR product integration (*d*, 4.62 ppm, 1H)	Cycloisomerized product integration (*d*, 5.33 ppm, 1H)
0 h	1.0000	0.6733	0.0000	N/A
24 h	1.0000	0.2870	0.1547	trace

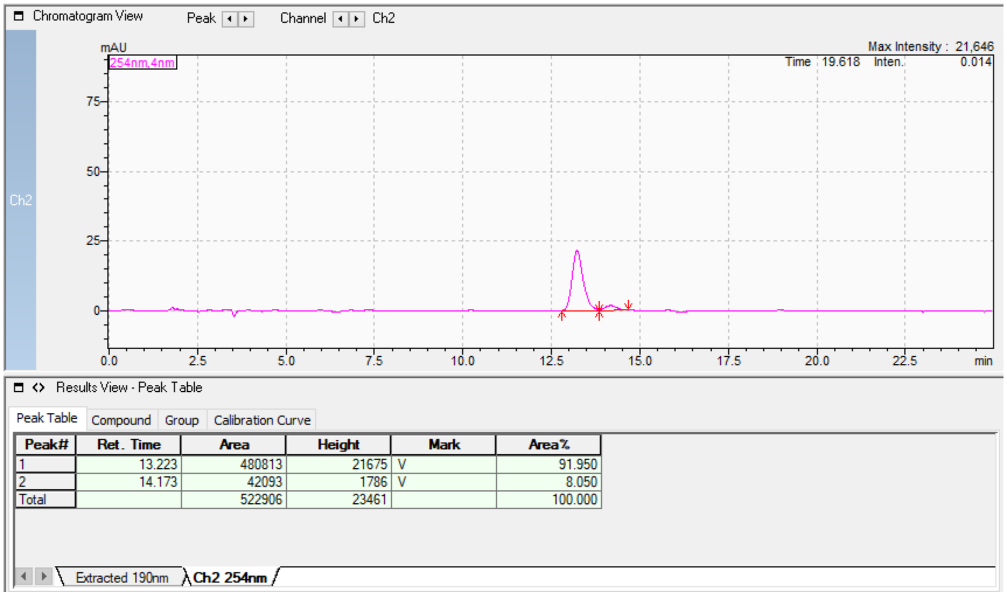

%*ee* (run 1) = 91.950 – 8.050 = 83.900%

Run 2-Follows general procedure D. Rh(cod)$_2$OTf (7.1 mg, 0.015 mmol, 0.1 equiv), (*R*)-BINAP (10.3 mg, 0.0165 mmol, 0.11 equiv), mesitylene (18.0 mg, 0.15 mmol, 1.0 equiv) and enyne **4a** (0.5 mL from a stock solution of 0.3 M) in trifluorotoluene (3.0 mL, 0.05 M). After 24 h, PKR yield (46%), b.r.s.m. (82%), starting material remaining (44%) and cycloisomerized side product (trace) were determined by integral comparison to the internal standard mesitylene.

run 2:

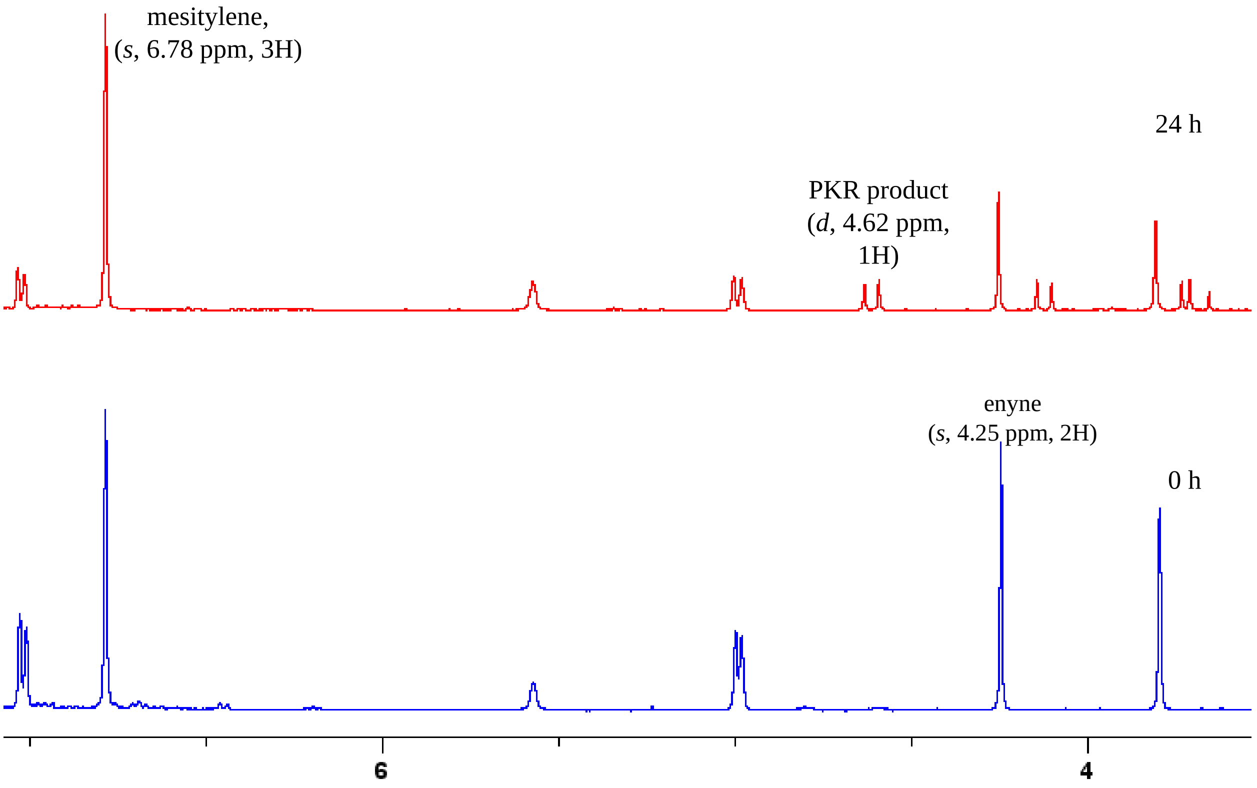

	mesitylene integration (*s*, 6.78 ppm, 3H)	enyne integration(*s*, 4.25 ppm, 2H)	PKR product integration (*d*, 4.62 ppm, 1H)	Cycloisomerized product integration (*d*, 5.33 ppm, 1H)
0 h	1.0000	0.6978	0.0000	0.0000
24 h	1.0000	0.3075	0.1627	trace

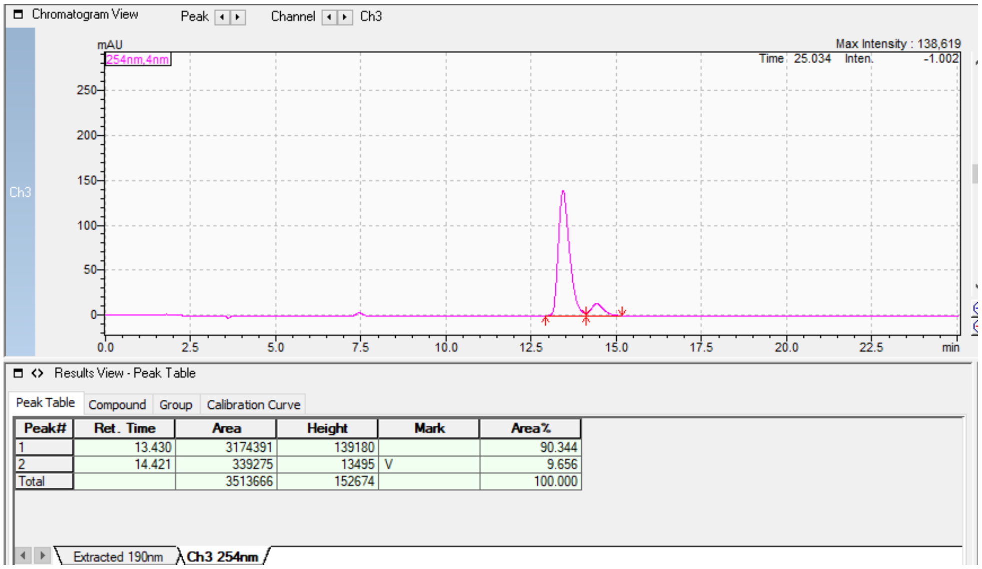

Peak#	Ret. Time	Area	Height	Mark	Area%
1	13.430	3174391	139180		90.344
2	14.421	339275	13495	V	9.656
Total		3513666	152674		100.000

%ee (run 2) = 90.344 – 9.656 = 80.688 %

%ee (average) = (83.900 + 80.688) = 82.294 = 82%

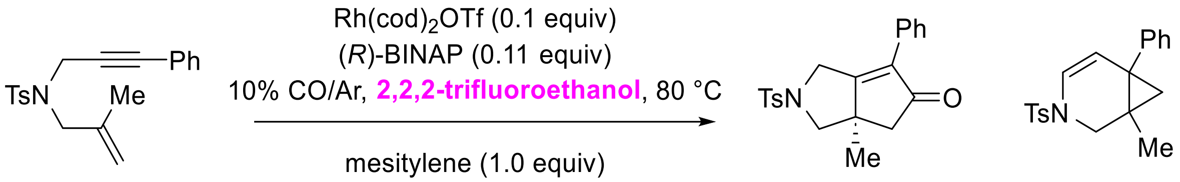

4a **5a** **5ai**

Run 1-Follows general procedure D. Rh(cod)$_2$OTf (7.1 mg, 0.015 mmol, 0.1 equiv), (*R*)-BINAP (10.3 mg, 0.0165 mmol, 0.11 equiv), mesitylene (18.0 mg, 0.15 mmol, 1.0 equiv) and enyne **4a** (0.5 mL from a stock solution of 0.3 M) in 2,2,2-trifluoroethanol (3.0 mL, 0.05 M). After 24 h, PKR yield (27%), b.r.s.m. (84%), starting material remaining (68%) and cycloisomerized side product (4%) were determined by integral comparison to the internal standard mesitylene.

__run 1:__

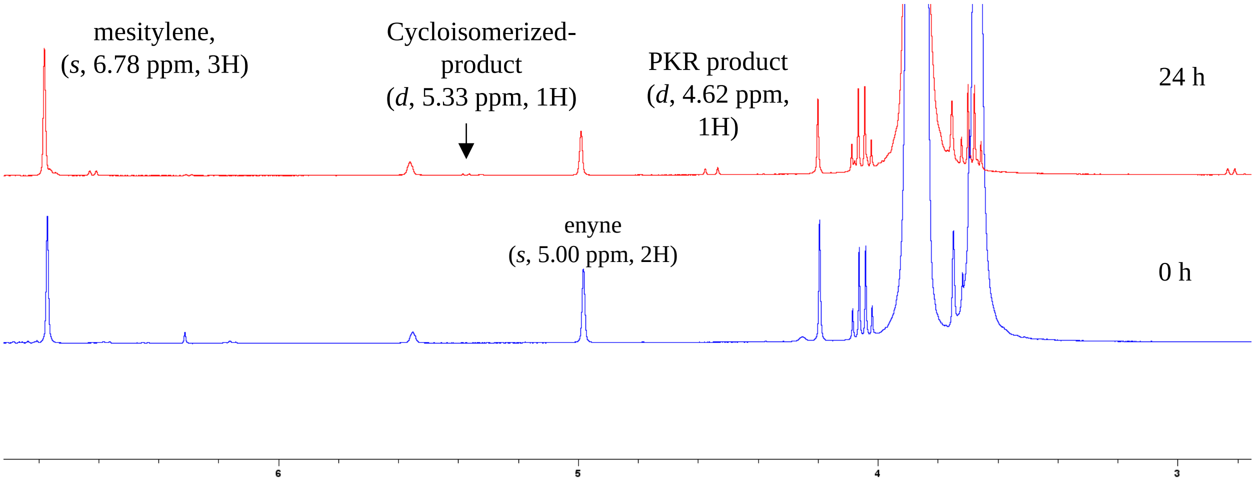

	mesitylene integration (*s*, 6.78 ppm, 3H)	enyne integration(*s*, 5.00 ppm, 2H)	PKR product integration (*d*, 4.62 ppm, 1H)	Cycloisomerized product integration (*d*, 5.33 ppm, 1H)
0 h	1.0000	0.7025	0.0000	0.0000
24 h	1.0000	0.4799	0.0932	0.0132

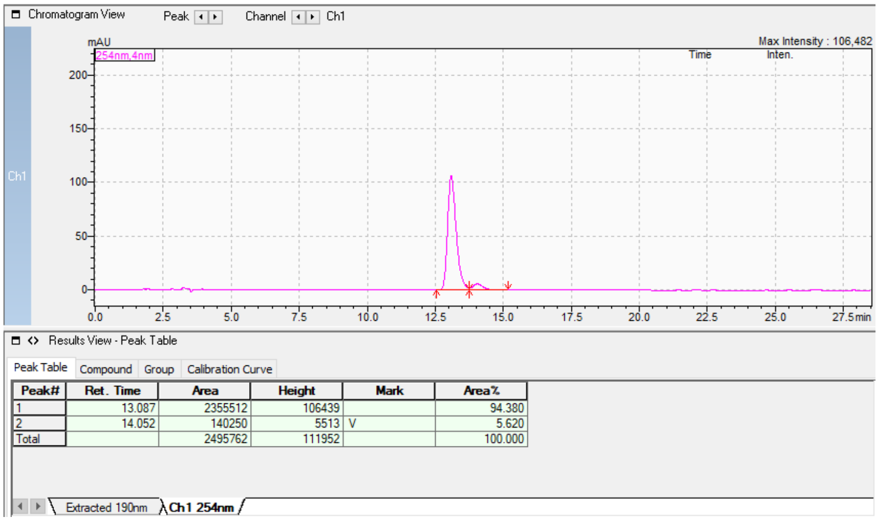

Peak#	Ret. Time	Area	Height	Mark	Area%
1	13.087	2355512	106439		94.380
2	14.052	140250	5513	V	5.620
Total		2495762	111952		100.000

%*ee* (run 1) = 94.380 – 5.620 = 88.760 %

Run 2-Follows general procedure D. Rh(cod)$_2$OTf (7.1 mg, 0.015 mmol, 0.1 equiv), (*R*)-BINAP (10.3 mg, 0.0165 mmol, 0.11 equiv), mesitylene (18.0 mg, 0.15 mmol, 1.0 equiv) and enyne **4a** (0.5 mL from a stock solution of 0.3 M) in 2,2,2-trifluoroethanol (3.0 mL, 0.05 M). After 24 h, PKR yield (22%), b.r.s.m. (59%), starting material remaining (63%) and cycloisomerized side product (3%) were determined by integral comparison to the internal standard mesitylene.

run 2:

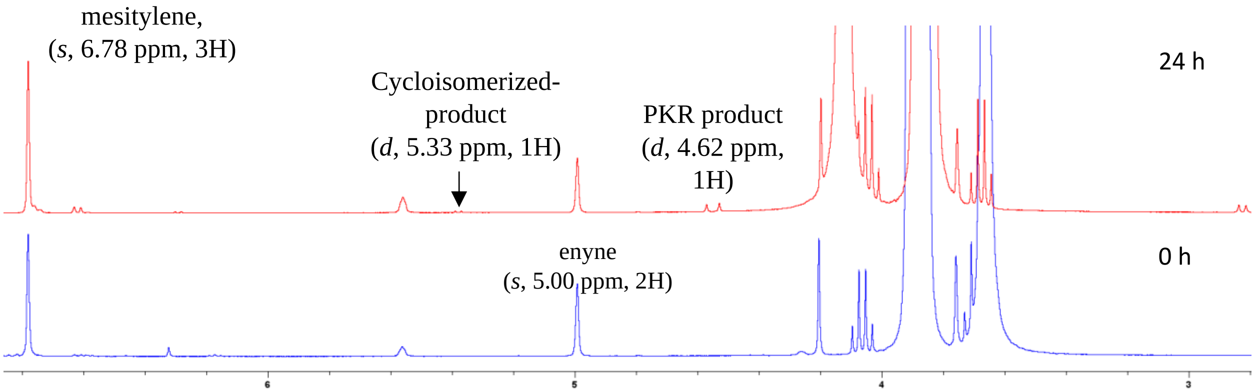

	mesitylene integration (*s*, 6.78 ppm, 3H)	enyne integration(*s*, 5.00 ppm, 2H)	PKR product integration (*d*, 4.62 ppm, 1H)	cycloisomerized product integration (d, 2.
0 h	1.0000	0.7293	0.0000	0.0000
24 h	1.0000	0.4587	0.0790	0.0099

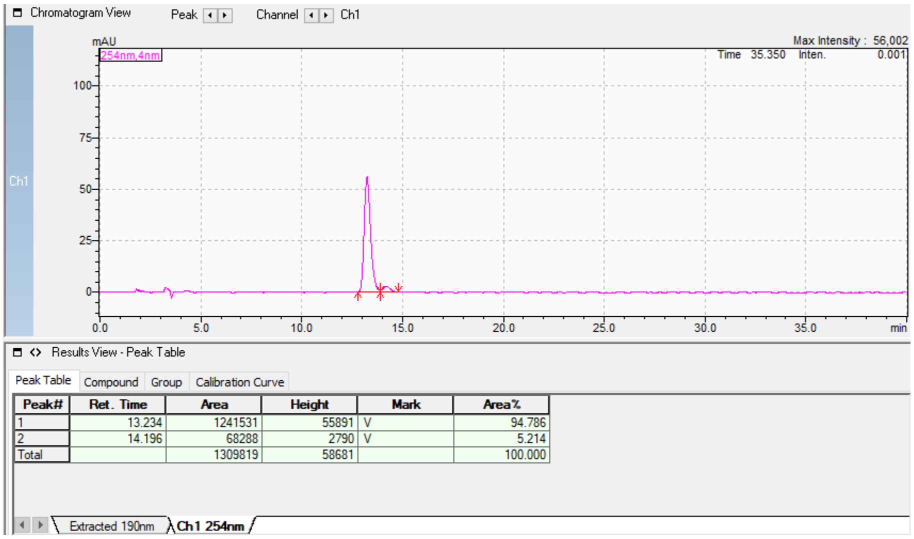

Peak#	Ret. Time	Area	Height	Mark	Area%
1	13.234	1241531	55891	V	94.786
2	14.196	68288	2790	V	5.214
Total		1309819	58681		100.000

%*ee* (run 2) = 94.786 – 5.214 = 89.572%

%*ee* (average) = (88.760 + 89.572)/2 = 89.166 = 89%

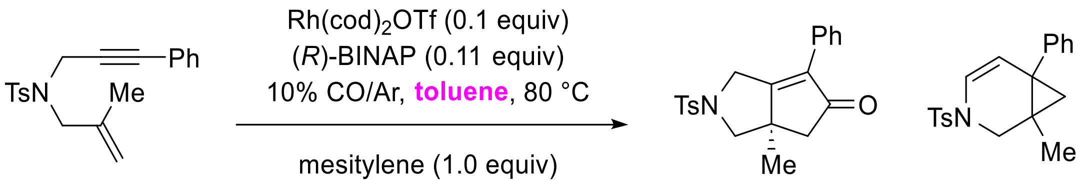

4a **5a** **5ai**

Run 1-Follows general procedure D. $Rh(cod)_2OTf$ (7.1 mg, 0.015 mmol, 0.1 equiv), (*R*)-BINAP (10.3 mg, 0.0165 mmol, 0.11 equiv), mesitylene (18.0 mg, 0.15 mmol, 1.0 equiv) and enyne **4a** (0.5 mL from a stock solution of 0.3 M) in toluene (3.0 mL, 0.05 M). After 24 h, PKR yield (25%), b.r.s.m. (61%), starting material remaining (59%) and cycloisomerized side product (5%) were determined by integral comparison to the internal standard mesitylene.

run 1:

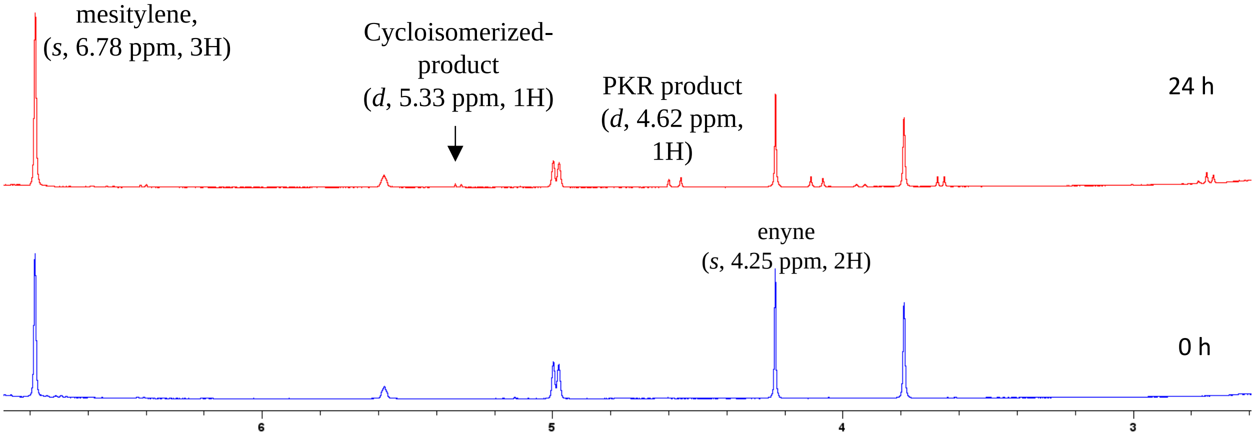

	mesitylene integration (*s*, 6.78 ppm, 3H)	enyne integration(*s*, 4.25 ppm, 2H)	PKR product integration (*d*, 4.62 ppm, 1H)	Cycloisomerized product integration (*d*, 5.33 ppm, 1H)
0 h	1.0000	0.6705	0.0000	0.0000
24 h	1.0000	0.3946	0.0831	0.0167

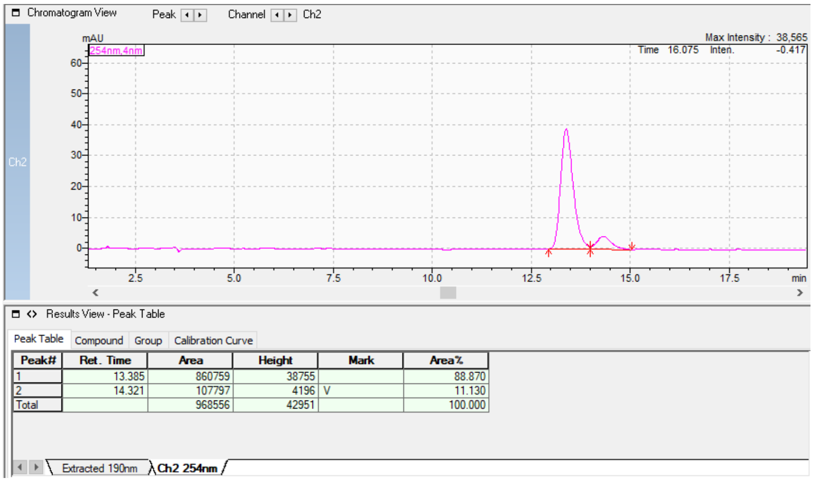

Peak#	Ret. Time	Area	Height	Mark	Area%
1	13.385	860759	38755		88.870
2	14.321	107797	4196	V	11.130
Total		968556	42951		100.000

%*ee* (run 1) = 88.870 – 11.130 = 77.740%

Run 2-Follows general procedure D. Rh(cod)$_2$OTf (7.1 mg, 0.015 mmol, 0.1 equiv), (*R*)-BINAP (10.3 mg, 0.0165 mmol, 0.11 equiv), mesitylene (18.0 mg, 0.15 mmol, 1.0 equiv) and enyne **4a** (0.5 mL from a stock solution of 0.3 M) in toluene (3.0 mL, 0.05 M). After 24 h, PKR yield (30%), b.r.s.m. (71%), starting material remaining (58%) and cycloisomerized side product (4%) were determined by integral comparison to the internal standard mesitylene.

run 2:

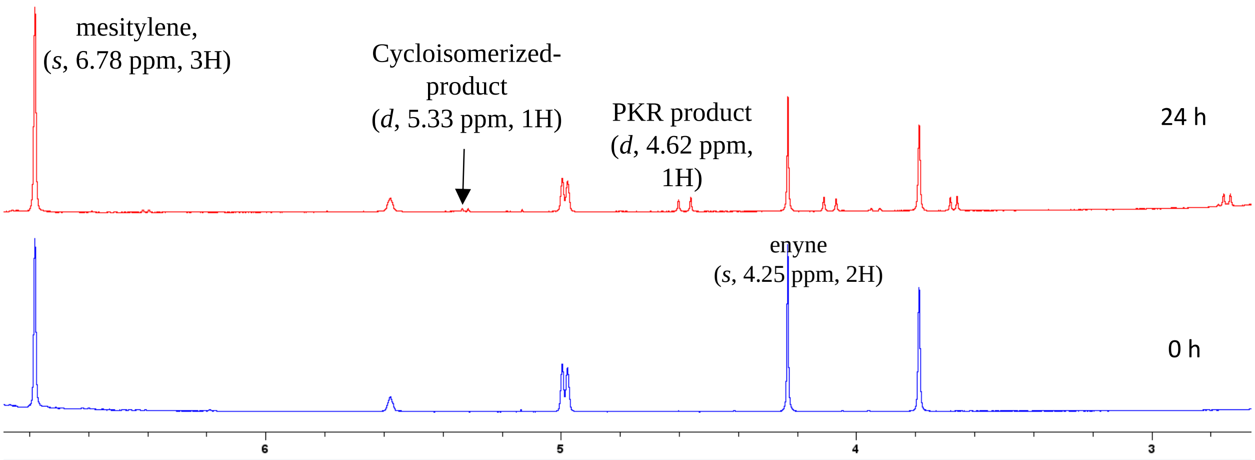

	mesitylene integration (*s*, 6.78 ppm, 3H)	enyne integration(*s*, 4.25 ppm, 2H)	PKR product integration (*d*, 4.62 ppm, 1H)	Cycloisomerized product integration (*d*, 5.33 ppm, 1H)
0 h	1.0000	0.7234	0.0000	0.0000
24 h	1.0000	0.4152	0.1082	0.0153

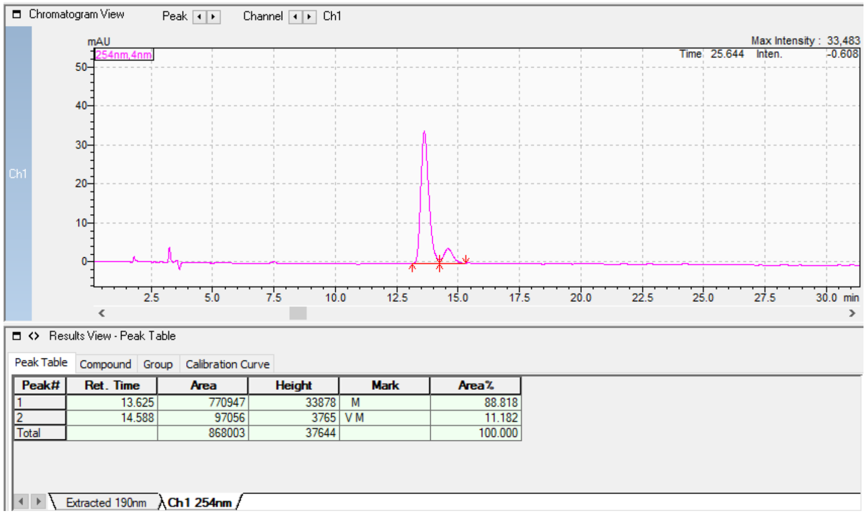

Peak#	Ret. Time	Area	Height	Mark	Area%
1	13.625	770947	33878	M	88.818
2	14.588	97056	3765	V M	11.182
Total		868003	37644		100.000

%*ee* (run 2) = 88.818 – 11.182 = 77.636%

%*ee* (average) = (77.740 + 77.636)/2 = 77.688 = 78%

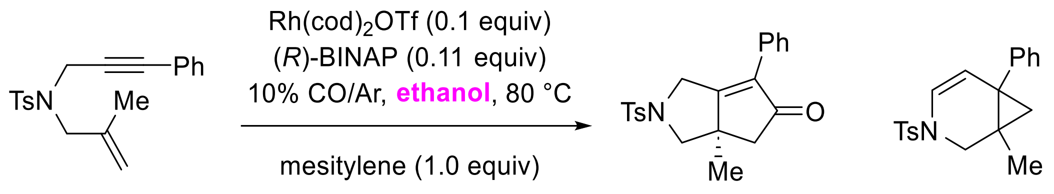

4a **5a** **5ai**

Run 1-Follows a modified procedure of general procedure D due to low solubility of enyne 4a in ethanol. A 15-mL flask was charged with Rh(cod)$_2$OTf (17 mg, 0.036 mmol)and (*R*)-BINAP (25 mg, 0.040 mmol) and equipped with a reflux condenser in a nitrogen-filled glovebox. The apparatus was removed from the glovebox and placed under an atmosphere of Ar. The ethanol/mesitylene stock solution (7.2 mL) was added via syringe and the flask was lowered into a preheated oil bath (60 °C). After 1 h, the argon atmosphere was replaced with 10% CO/Ar release cycles (3×) using a needle connected to a vacuum manifold and the reaction was maintained for 1 h at 60 °C. The flask was removed from the oil bath and allowed to cool to rt and the resulting catalyst solution (3 mL, containing Rh(cod)$_2$OTf (7.1 mg, 0.015 mmol, 0.1 equiv), (*R*)-BINAP (10.4 mg, 0.0165 mmol, 0.11 equiv) and mesitylene (18.0 mg, 0.15 mmol, 1.0 equiv)) was transferred via a syringe to another reflux apparatus charged with enyne **4a** (51 mg, 0.15 mmol, 1.0 equiv) under 10% CO/Ar atmosphere. The reaction flask was lowered into the preheated oil bath (80 °C). After 24 h, PKR yield (36%), b.r.s.m. (72%), starting material remaining (50%) and cycloisomerized side product (0%) were determined by integral comparison to the internal standard mesitylene.

run 1:

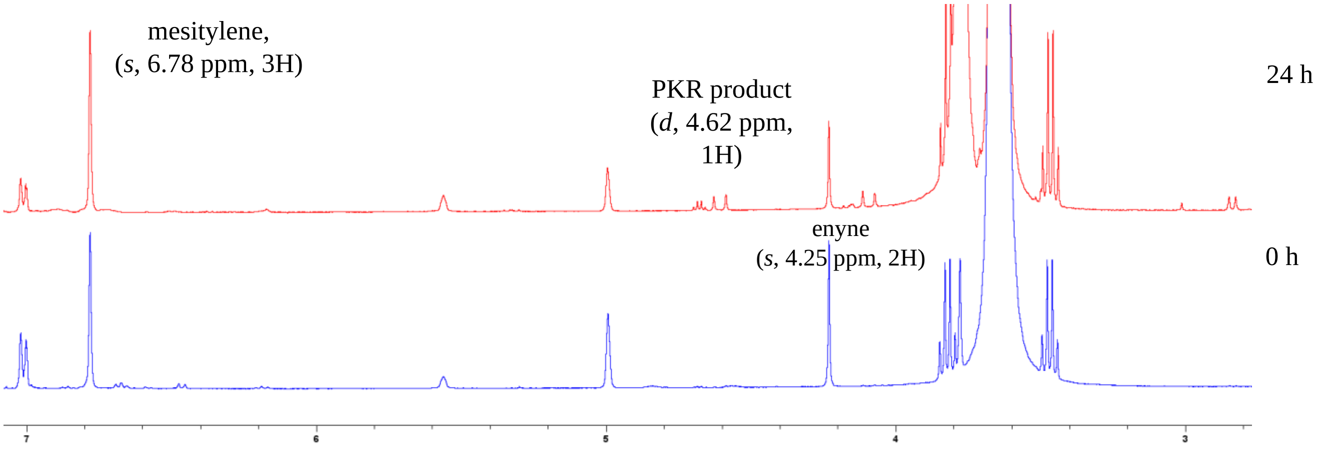

	mesitylene integration (*s*, 6.78 ppm, 3H)	enyne integration(*s*, 4.25 ppm, 2H)	PKR product integration (*d*, 4.62 ppm, 1H)	Cycloisomerized product integration (*d*, 5.33 ppm, 1H)
0 h	1.0000	0.6962	0.0000	0.0000
24 h	1.0000	0.3486	0.1252	0.0000

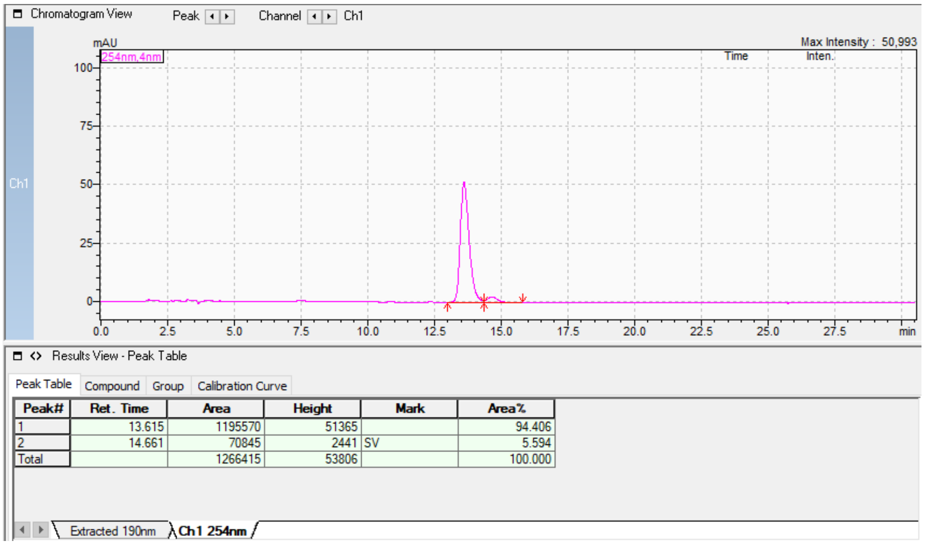

%*ee* (run 1) = 94.406 – 5.594 = 88.812%

Run 2-Follows a modified procedure of general procedure D due to low solubility of enyne 4a in ethanol. A 15-mL flask was charged with Rh(cod)$_2$OTf (17 mg, 0.036 mmol)and (*R*)-BINAP (25 mg, 0.040 mmol) and equipped with a reflux condenser in a nitrogen-filled glovebox. The apparatus was removed from the glovebox and placed under an atmosphere of Ar. The ethanol/mesitylene stock solution (7.2 mL) was added via syringe and the flask was lowered into a preheated oil bath (60 °C). After 1 h, the argon atmosphere was replaced with 10% CO/Ar release cycles (3×) using a needle connected to a vacuum manifold and the reaction was maintained for 1 h at 60 °C. The flask was removed from the oil bath and allowed to cool to rt and the resulting catalyst solution (3 mL, containing Rh(cod)$_2$OTf (7.1 mg, 0.015 mmol, 0.1 equiv), (*R*)-BINAP (10.4 mg, 0.0165 mmol, 0.11 equiv) and mesitylene (18.0 mg, 0.15 mmol, 1.0 equiv)) was transferred via a syringe to another reflux apparatus charged with enyne **4a** (51 mg, 0.15 mmol, 1.0 equiv) under 10% CO/Ar atmosphere. The reaction flask was lowered into the preheated oil bath (80 °C). After 24 h, PKR yield (34%), b.r.s.m. (69%), starting material remaining (51%) and cycloisomerized side product (0%) were determined by integral comparison to the internal standard mesitylene.

<u>run 2:</u>

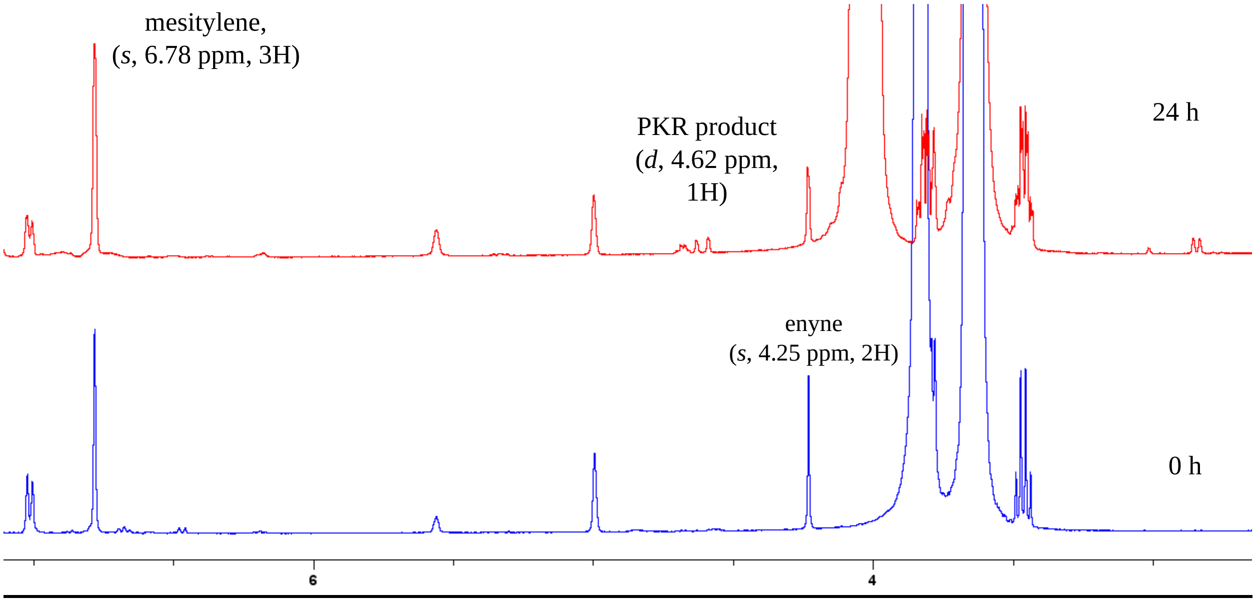

	mesitylene integration (*s*, 6.78 ppm, 3H)	enyne integration(*s*, 4.25 ppm, 2H)	PKR product integration (*d*, 4.62 ppm, 1H)	Cycloisomerized product integration (*d*, 5.33 ppm, 1H)
0 h	1.0000	0.6078	0.0000	0.0000
24 h	1.0000	0.3074	0.1031	0.0000

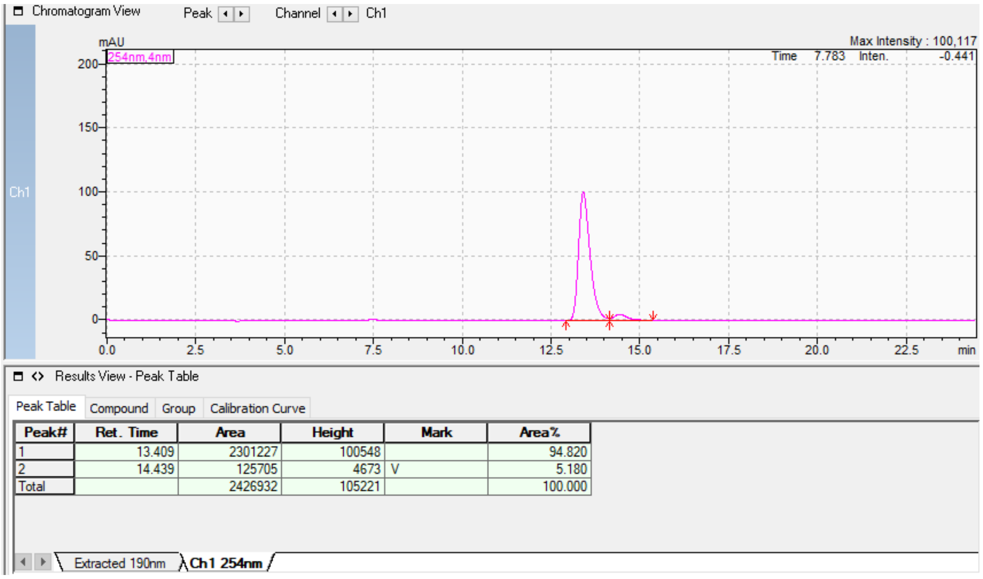

Peak#	Ret. Time	Area	Height	Mark	Area%
1	13.409	2301227	100548		94.820
2	14.439	125705	4673	V	5.180
Total		2426932	105221		100.000

%*ee* (run 2) = 94.820 – 5.180 = 89.640%

%*ee* (average) = (88.812 + 89.640)/2 = 89.226 = 89%

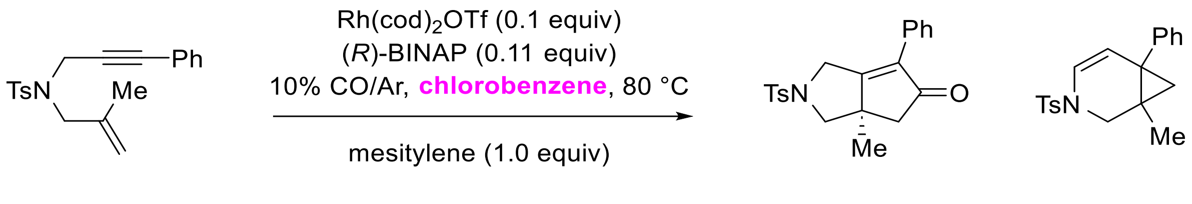

Run 1-Follows general procedure D. Rh(cod)$_2$OTf (7.1 mg, 0.015 mmol, 0.1 equiv), (*R*)-BINAP (10.3 mg, 0.0165 mmol, 0.11 equiv), mesitylene (18.0 mg, 0.15 mmol, 1.0 equiv) and enyne **4a** (0.5 mL from a stock solution of 0.3 M) in chlorobenzene (3.0 mL, 0.05 M). After 24 h, PKR yield (31%), b.r.s.m. (72%), starting material remaining (57%) and cycloisomerized side product (2%) were determined by integral comparison to the internal standard mesitylene.

run 1:

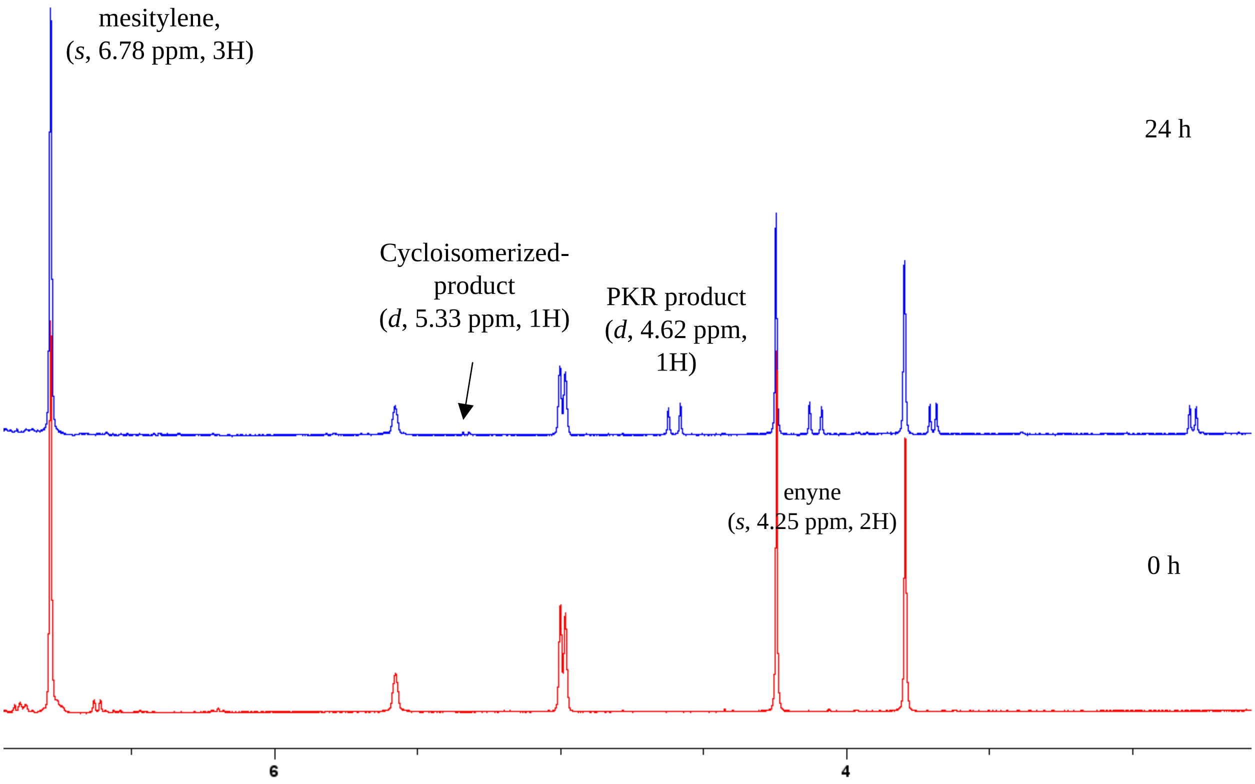

	mesitylene integration (*s*, 6.78 ppm, 3H)	enyne integration(*s*, 4.25 ppm, 2H)	PKR product integration (*d*, 4.62 ppm, 1H)	Cycloisomerized product integration (*d*, 5.33 ppm, 1H)
0 h	1.0000	0.7284	0.0000	0.0000

24 h	1.0000	0.4172	0.1130	0.0081

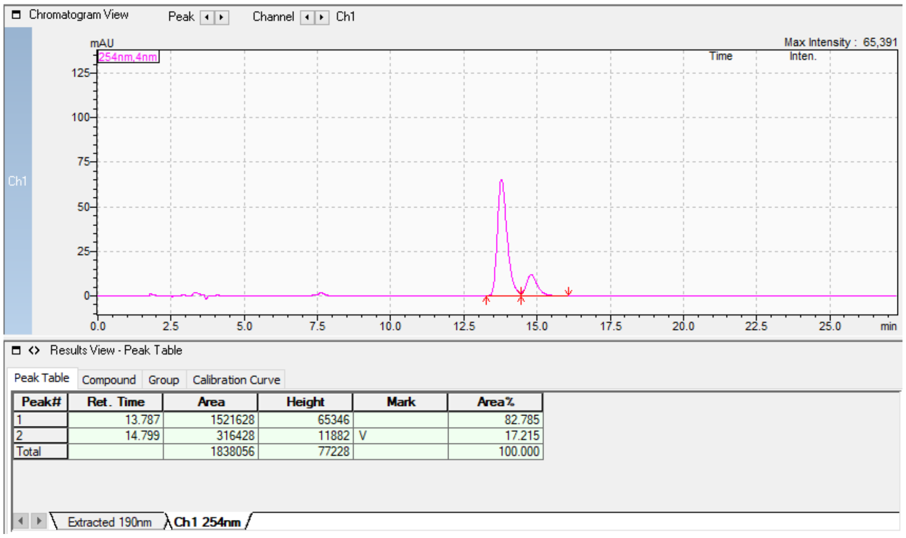

%*ee* (run 1) = 82.785-17.215= 65.570%

Run 2-Follows general procedure D. Rh(cod)$_2$OTf (7.1 mg, 0.015 mmol, 0.1 equiv), (*R*)-BINAP (10.3 mg, 0.0165 mmol, 0.11 equiv), mesitylene (18.0 mg, 0.15 mmol, 1.0 equiv) and enyne **4a** (0.5 mL from a stock solution of 0.3 M) in chlorobenzene (3.0 mL, 0.05 M). After 24 h, PKR yield (32%), b.r.s.m. (73%), starting material remaining (56%) and cycloisomerized side product (2%) were determined by integral comparison to the internal standard mesitylene.

<u>run 2:</u>

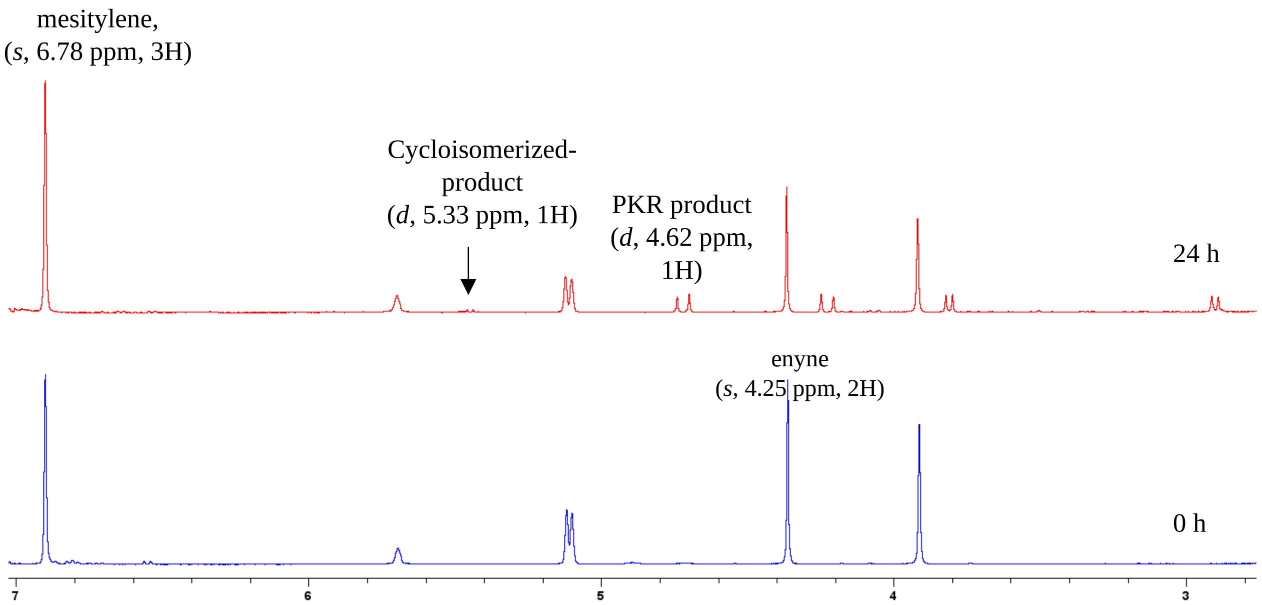

	mesitylene integration (*s*, 6.78 ppm, 3H)	enyne integration(*s*, 4.25 ppm, 2H)	PKR product integration (*d*, 4.62 ppm, 1H)	Cycloisomerized product integration (*d*, 5.33 ppm, 1H)
0 h	1.0000	0.7384	0.0000	0.0000
24 h	1.0000	0.4096	0.1170	0.0069

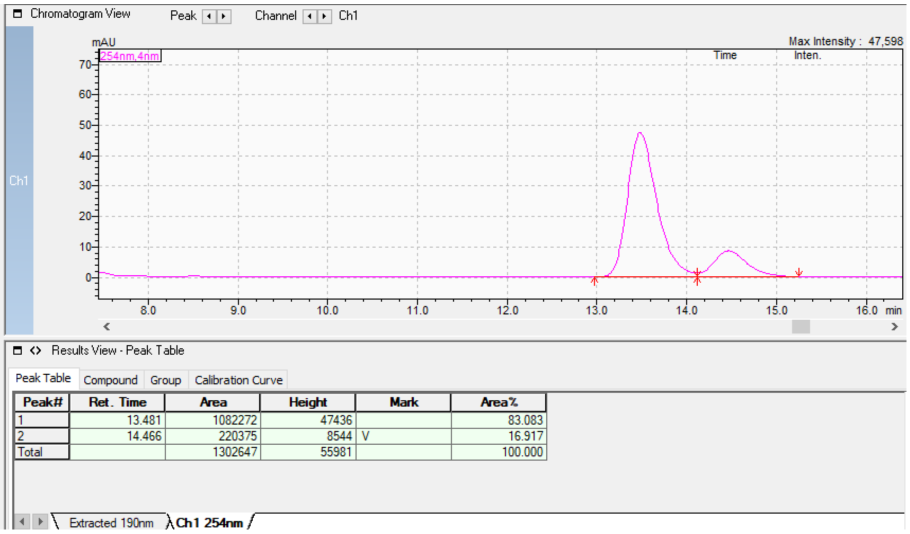

Peak#	Ret. Time	Area	Height	Mark	Area%
1	13.481	1082272	47436		83.083
2	14.466	220375	8544	V	16.917
Total		1302647	55981		100.000

%*ee* (run 2) = 83.083-16.917=66.166%

%*ee* (average) = (65.570+66.166)/2 = 65.868=66%

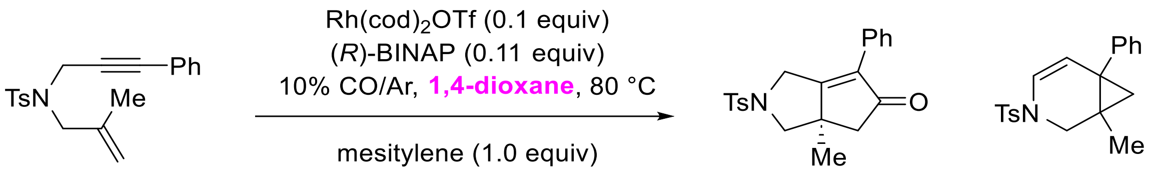

4a **5a** **5ai**

Run 1-Follows general procedure D. $Rh(cod)_2OTf$ (7.1 mg, 0.015 mmol, 0.1 equiv), (*R*)-BINAP (10.3 mg, 0.0165 mmol, 0.11 equiv), mesitylene (18.0 mg, 0.15 mmol, 1.0 equiv) and enyne **4a** (0.5 mL from a stock solution of 0.3 M) in 1,4-dioxane (3.0 mL, 0.05 M). After 24 h, PKR yield (36%), b.r.s.m. (78%), starting material remaining (54%) and cycloisomerized side product (4%) were determined by integral comparison to the internal standard mesitylene.

run 1:

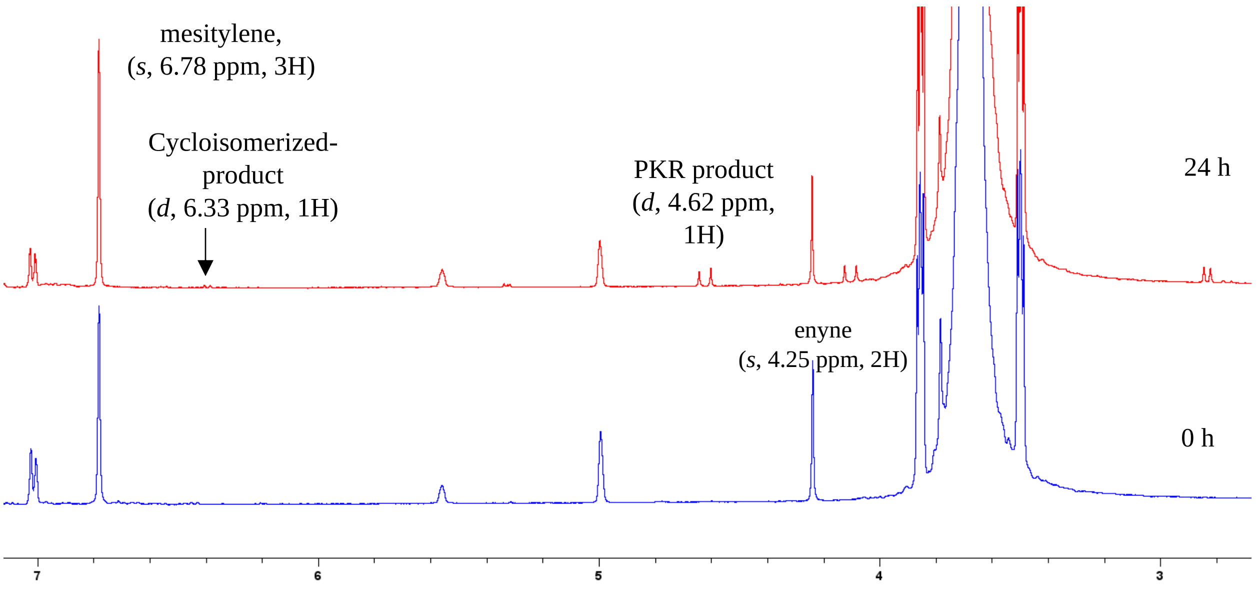

	mesitylene integration (*s*, 6.78 ppm, 3H)	enyne integration(*s*, 4.25 ppm, 2H)	PKR product integration (*d*, 4.62 ppm, 1H)	Cycloisomerized product integration (*d*, 6.33 ppm, 1H)
0 h	1.0000	0.5778	0.0000	0.0000
24 h	1.0000	0.3131	0.1030	0.0124

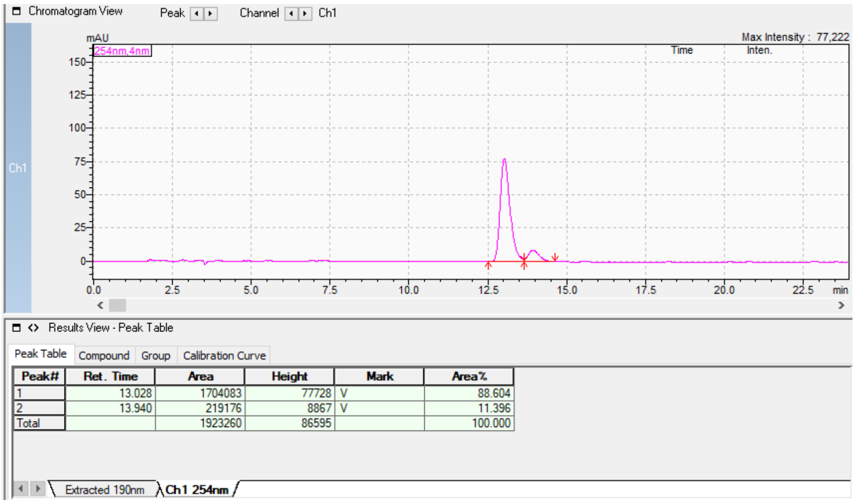

Peak#	Ret. Time	Area	Height	Mark	Area%
1	13.028	1704083	77728	V	88.604
2	13.940	219176	8867	V	11.396
Total		1923260	86595		100.000

%*ee* (run 1) = 88.604-11.396=77.208%

Run 2-Follows general procedure D. Rh(cod)$_2$OTf (7.1 mg, 0.015 mmol, 0.1 equiv), (*R*)-BINAP (10.3 mg, 0.0165 mmol, 0.11 equiv), mesitylene (18.0 mg, 0.15 mmol, 1.0 equiv) and enyne **4a** (0.5 mL from a stock solution of 0.3 M) in 1,4-dioxane (3.0 mL, 0.05 M). After 24 h, PKR yield (26%), b.r.s.m. (76%), starting material remaining (66%) and cycloisomerized side product (0%) were determined by integral comparison to the internal standard mesitylene.

run 2:

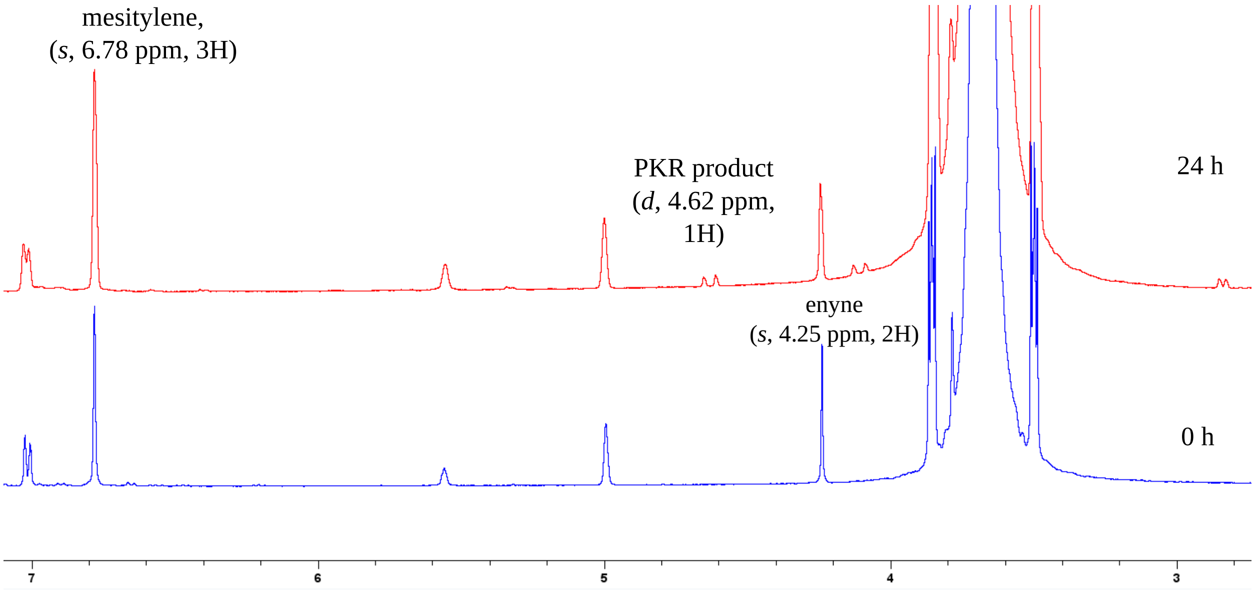

	mesitylene integration (*s*, 6.78 ppm, 3H)	enyne integration(*s*, 4.25 ppm, 2H)	PKR product integration (*d*, 4.62 ppm, 1H)	Cycloisomerized product integration (*d*, 6.33 ppm, 1H)
0 h	1.0000	0.5861	0.0000	0.0000
24 h	1.0000	0.3894	0.0760	0.0000

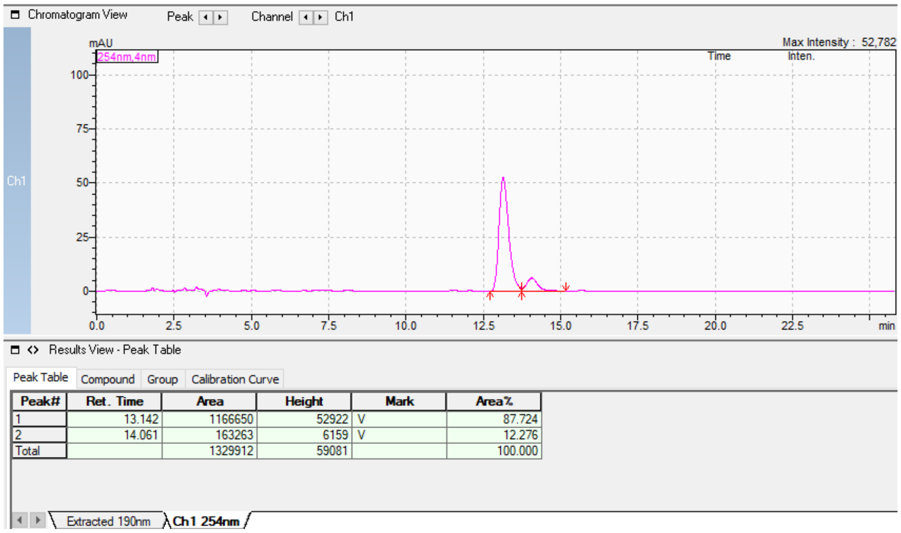

%*ee* (run 2) = 87.724-12.276=75.448%

%*ee* (average) = (77.208+75.448)/2 = 76.328 = 76%

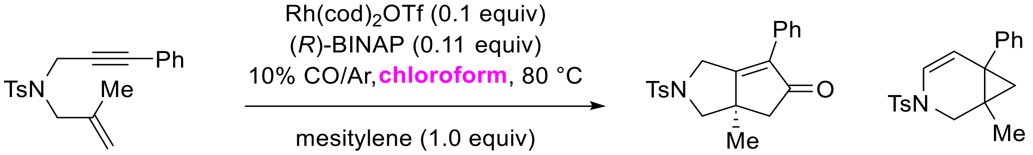

4a **5a** **5ai**

Run 1-Follows general procedure D. Rh(cod)$_2$OTf (7.1 mg, 0.015 mmol, 0.1 equiv), (*R*)-BINAP (10.3 mg, 0.0165 mmol, 0.11 equiv), mesitylene (18.0 mg, 0.15 mmol, 1.0 equiv) and enyne **4a** (0.5 mL from a stock solution of 0.3 M) in chloroform (3.0 mL, 0.05 M). After 24 h, PKR yield (61%), b.r.s.m. (86%), starting material remaining (29%) and cycloisomerized side product (0%) were determined by integral comparison to the internal standard mesitylene.

<u>**run 1:**</u>

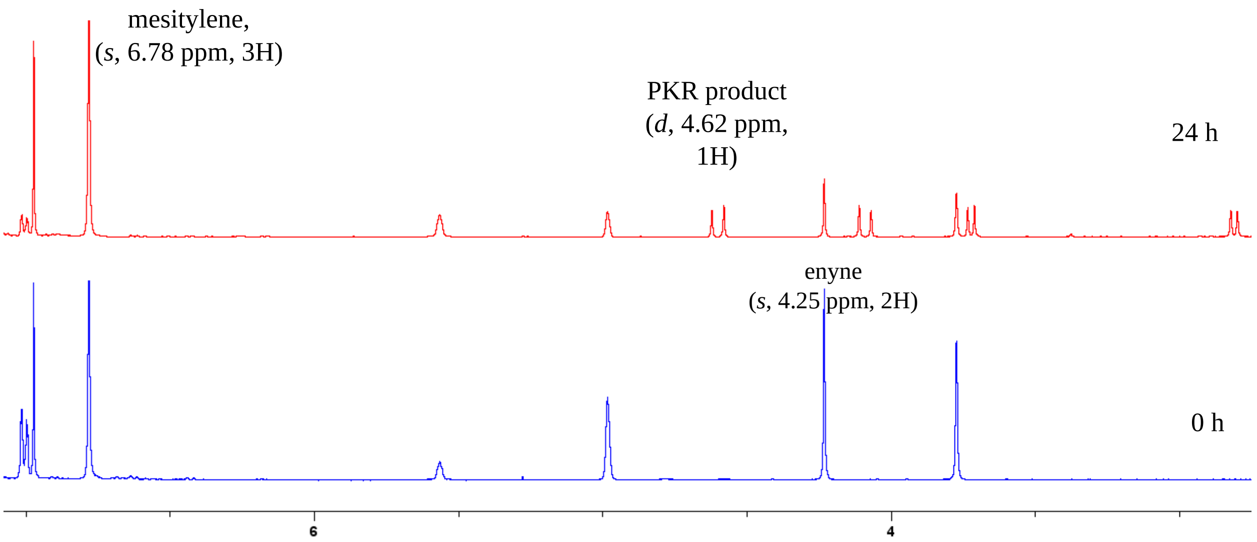

	mesitylene integration (*s*, 6.78 ppm, 3H)	enyne integration(*s*, 4.25 ppm, 2H)	PKR product integration (*d*, 4.62 ppm, 1H)	Cycloisomerized product integration (*d*, 5.3 ppm, 1H)
0 h	1.0000	0.6959	0.0000	0.0000
24 h	1.0000	0.2021	0.2093	0.0000

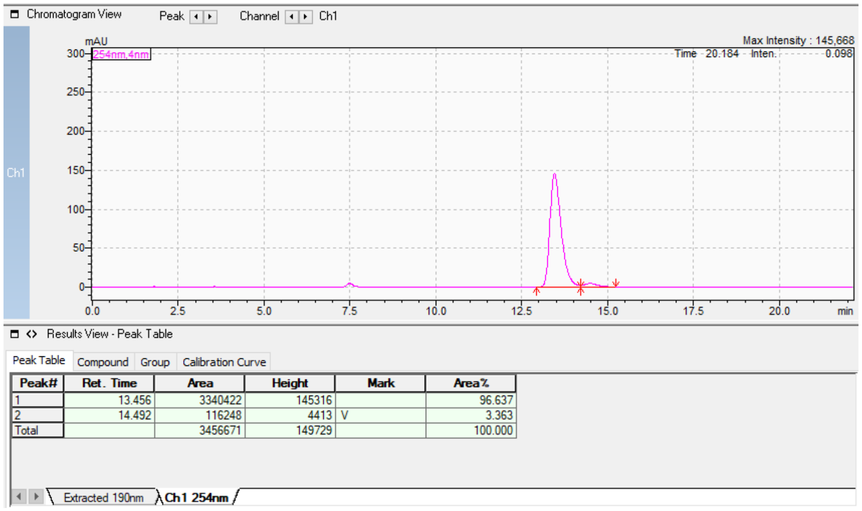

Peak#	Ret. Time	Area	Height	Mark	Area%
1	13.456	3340422	145316		96.637
2	14.492	116248	4413	V	3.363
Total		3456671	149729		100.000

%*ee* (run 1) = 96.637-3.363=93.274%

Run 2-Follows general procedure D. Rh(cod)$_2$OTf (7.1 mg, 0.015 mmol, 0.1 equiv), (*R*)-BINAP (10.3 mg, 0.0165 mmol, 0.11 equiv), mesitylene (18.0 mg, 0.15 mmol, 1.0 equiv) and enyne **4a** (0.5 mL from a stock solution of 0.3 M) in chloroform (3.0 mL, 0.05 M). After 24 h, PKR yield (69%), b.r.s.m. (91%), starting material remaining (24%) and cycloisomerized side product (0%) were determined by integral comparison to the internal standard mesitylene.

run 2:

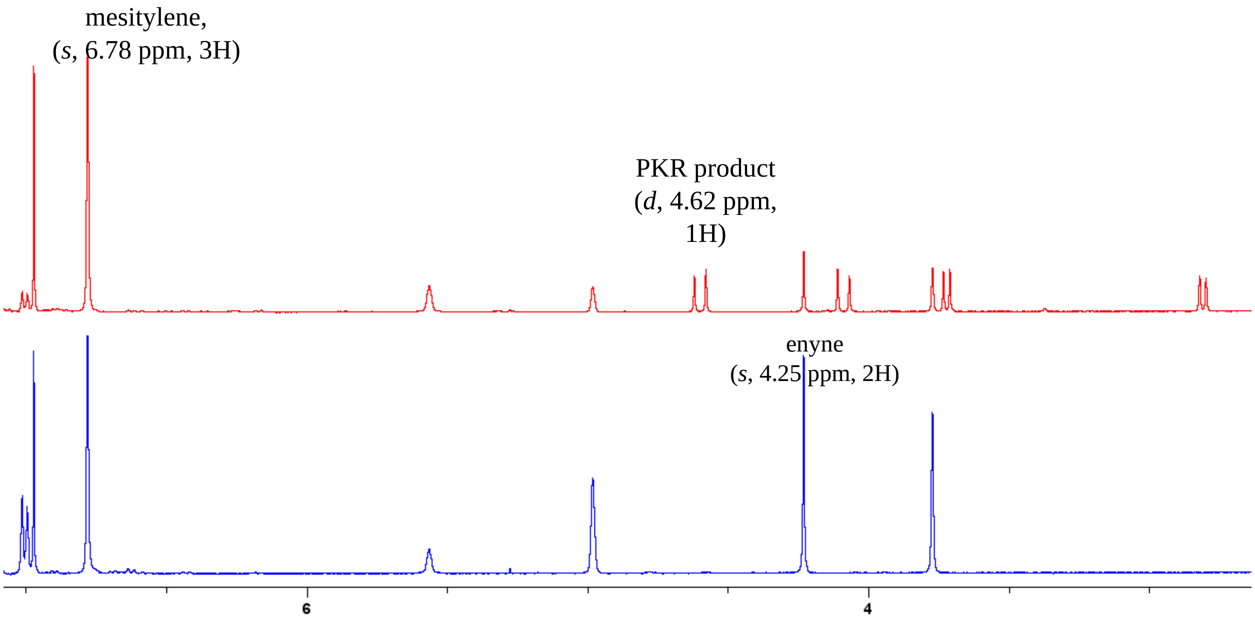

	mesitylene integration (*s*, 6.78 ppm, 3H)	enyne integration(*s*, 4.25 ppm, 2H)	PKR product integration (*d*, 4.62 ppm, 1H)	Cycloisomerized product integration (*d*, 5.3 ppm, 1H)
0 h	1.0000	0.6666	0.0000	0.0000
24 h	1.0000	0.1600	0.2315	0.0000

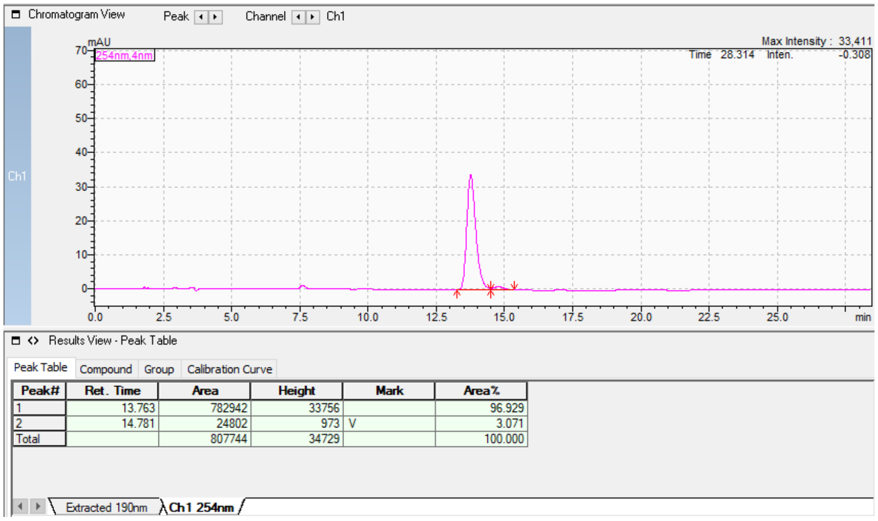

Peak#	Ret. Time	Area	Height	Mark	Area%
1	13.763	782942	33756		96.929
2	14.781	24802	973	V	3.071
Total		807744	34729		100.000

%*ee* (run 2) = 96.929-3.071=93.858%

%*ee* (average) = (93.274+93.858)/2 = 93.566 = 94%

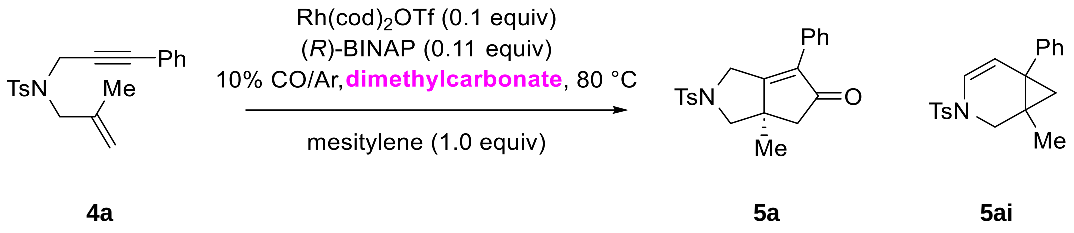

Run 1-Follows general procedure D. Rh(cod)$_2$OTf (7.1 mg, 0.015 mmol, 0.1 equiv), (*R*)-BINAP (10.3 mg, 0.0165 mmol, 0.11 equiv), mesitylene (18.0 mg, 0.15 mmol, 1.0 equiv) and enyne **4a** (0.5 mL from a stock solution of 0.3 M) in dimethylcarbonate (3.0 mL, 0.05 M). After 24 h, PKR yield (46%), b.r.s.m. (87%), starting material remaining (47%) and cycloisomerized side product (3%) were determined by integral comparison to the internal standard mesitylene.

run 1:

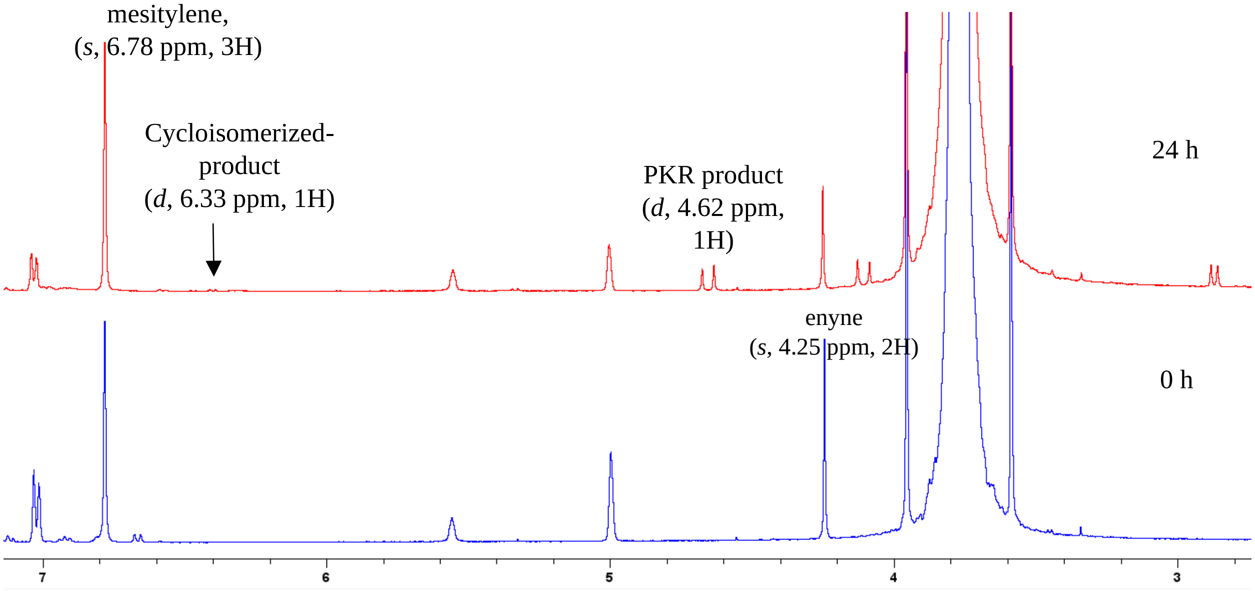

	mesitylene integration (*s*, 6.78 ppm, 3H)	enyne integration(*s*, 4.25 ppm, 2H)	PKR product integration (*d*, 4.62 ppm, 1H)	Cycloisomerized product integration (*d*, 6.33 ppm, 1H)
0 h	1.0000	0.6683	0.0000	0.0000
24 h	1.0000	0.3125	0.1527	0.0089

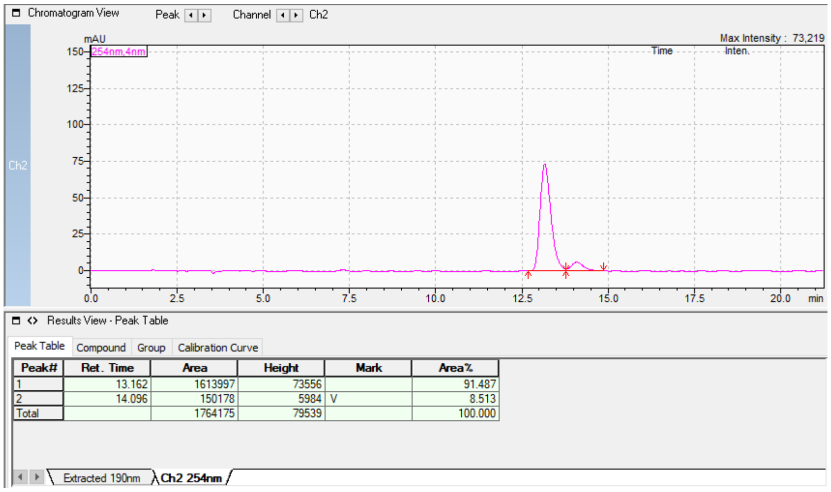

Peak#	Ret. Time	Area	Height	Mark	Area%
1	13.162	1613997	73556		91.487
2	14.096	150178	5984	V	8.513
Total		1764175	79539		100.000

%*ee* (run 1) = 91.487-8.513=82.974%

Run 2-Follows general procedure D. Rh(cod)$_2$OTf (7.1 mg, 0.015 mmol, 0.1 equiv), (*R*)-BINAP (10.3 mg, 0.0165 mmol, 0.11 equiv), mesitylene (18.0 mg, 0.15 mmol, 1.0 equiv) and enyne **4a** (0.5 mL from a stock solution of 0.3 M) in dimethylcarbonate (3.0 mL, 0.05 M). After 24 h, PKR yield (50%), b.r.s.m. (93%), starting material remaining (46%) and cycloisomerized side product (trace) were determined by integral comparison to the internal standard mesitylene.

run 2:

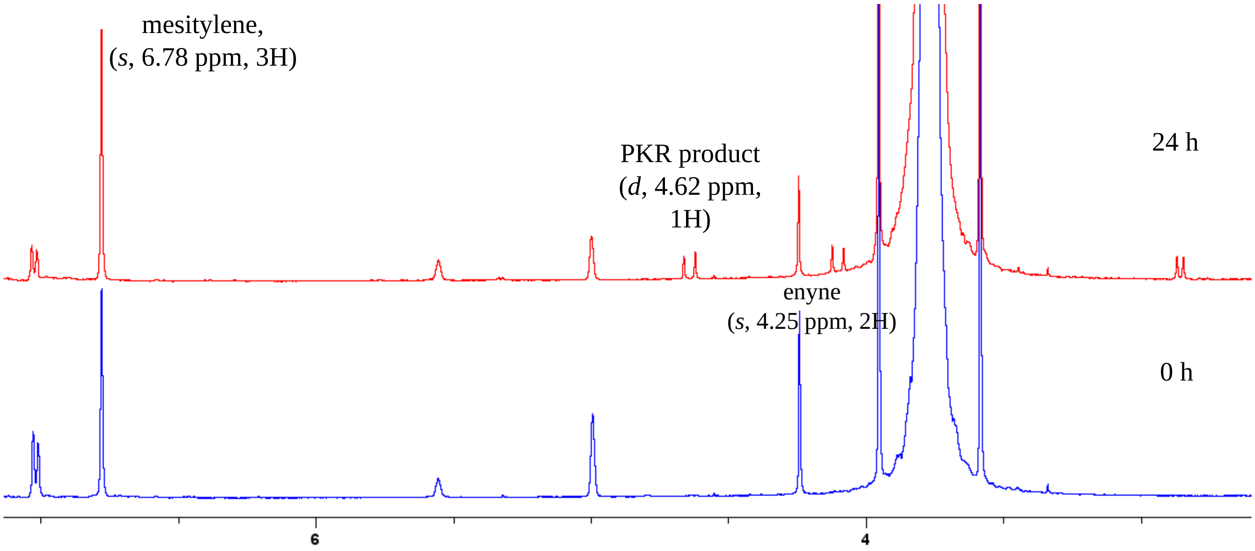

	mesitylene integration (*s*, 6.78 ppm, 3H)	enyne integration(*s*, 4.25 ppm, 2H)	PKR product integration (*d*, 4.62 ppm, 1H)	Cycloisomerized product integration (*d*, 6.33 ppm, 1H)
0 h	1.0000	0.6677	0.0000	0.0000

24 h	1.0000	0.3038	0.1667	trace

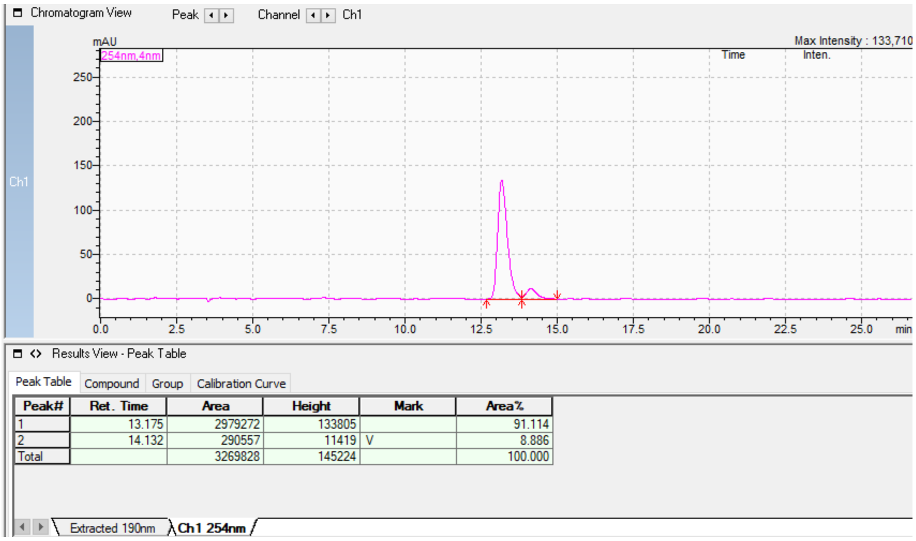

%*ee* (run 2) = 91.114-8.886=82.228%

%*ee* (average) = (82.974 + 82.228)/2 = 82.601= 83%

Spectral Data for Solvent Study Using Rh(cod)$_2$OTf and Ether-tethered Precursor 6a

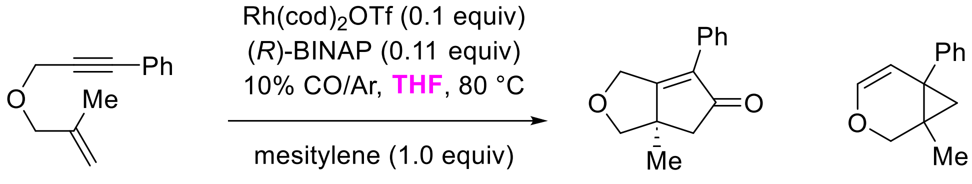

6a **7a** **7ai**

Follows general procedure D. Rh(cod)$_2$OTf (7.1 mg, 0.015 mmol, 0.1 equiv), (*R*)-BINAP (10.3 mg, 0.0165 mmol, 0.11 equiv), mesitylene (18.0 mg, 0.15 mmol, 1.0 equiv) and enyne **6a** (0.5 mL from a stock solution of 0.3 M) in THF (3.0 mL, 0.05 M). After 24 h, PKR yield (52%), b.r.s.m. (78%), starting material remaining (33%) and cycloisomerized side product (0%) were determined by integral comparison to the internal standard mesitylene.

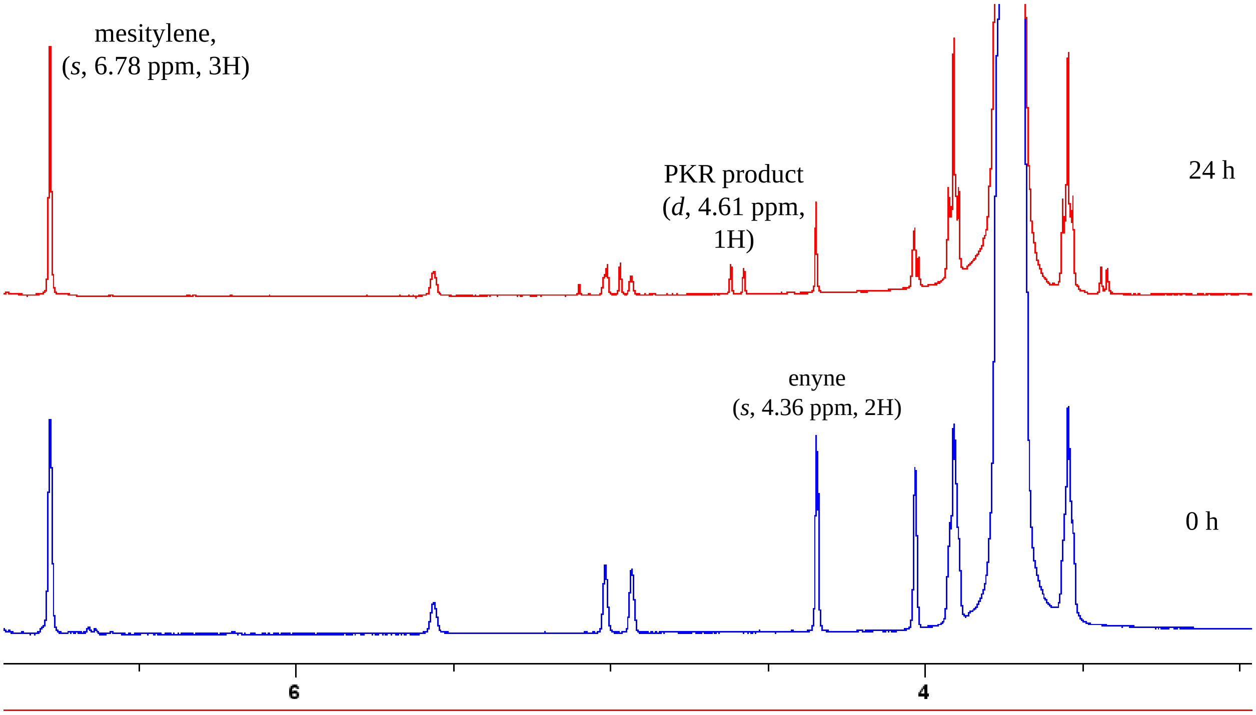

	mesitylene integration (*s*, 6.78 ppm, 3H)	enyne integration(*s*, 4.36 ppm, 2H)	PKR product integration (*d*, 4.61 ppm, 1H)	Cycloisomerized product integration (*d*, 5.21 ppm, 1H)
0 h	1.0000	0.6718	0.0000	0.0000
24 h	1.0000	0.2213	0.1745	0.0000

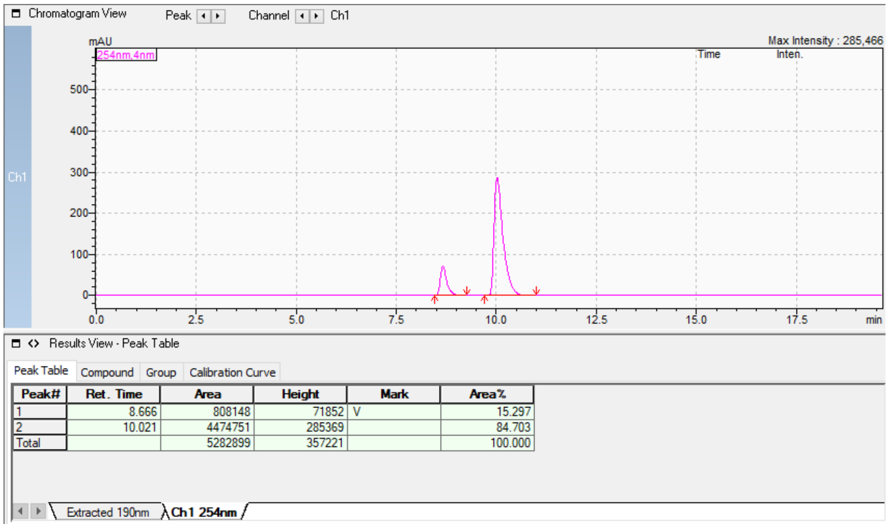

Peak#	Ret. Time	Area	Height	Mark	Area%
1	8.666	808148	71852	V	15.297
2	10.021	4474751	285369		84.703
Total		5282899	357221		100.000

%*ee* = 84.703-15.297 = 69.406 = 69%

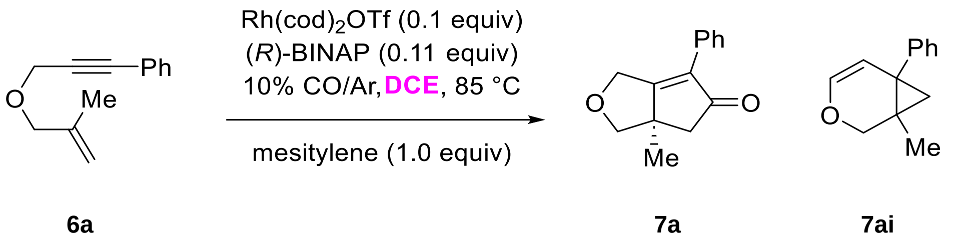

Follows general procedure D. Rh(cod)$_2$OTf (7.1 mg, 0.015 mmol, 0.1 equiv), (*R*)-BINAP (10.3 mg, 0.0165 mmol, 0.11 equiv), mesitylene (18.0 mg, 0.15 mmol, 1.0 equiv) and enyne **6a** (0.5 mL from a stock solution of 0.3 M) in DCE (3.0 mL, 0.05 M). After 23 h, PKR yield (84%), b.r.s.m. (85%), starting material remaining (1%) and cycloisomerized side product (13%) were determined by integral comparison to the internal standard mesitylene.

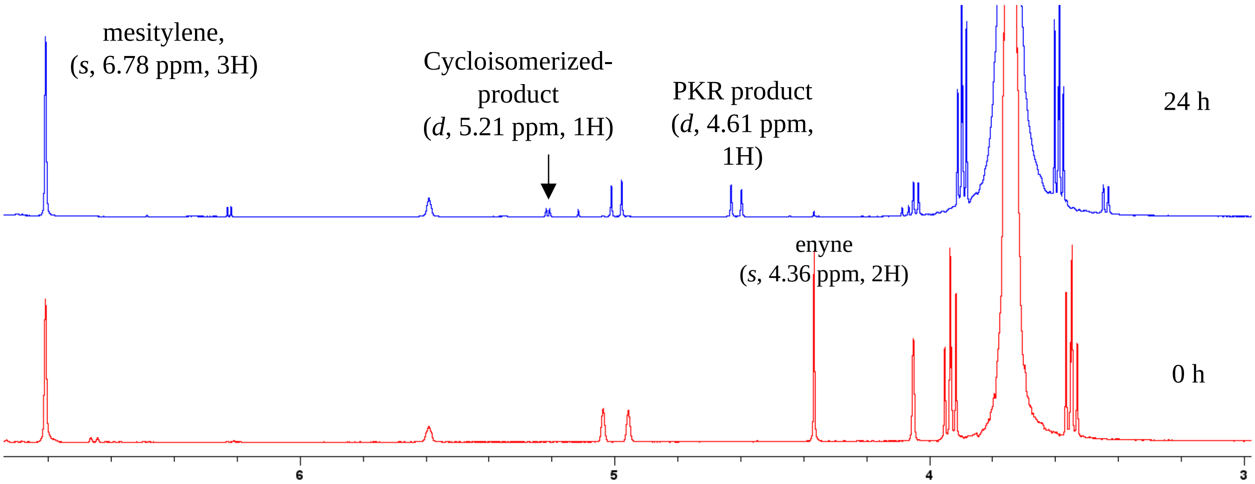

	mesitylene integration (*s*, 6.78 ppm, 3H)	enyne integration(*s*, 4.36 ppm, 2H)	PKR product integration (*d*, 4.61 ppm, 1H)	Cycloisomerized product integration (*d*, 5.21 ppm, 1H)
0 h	1.0000	0.6172	0.0000	0.0000
23 h	1.0000	0.0104	0.2581	0.0388

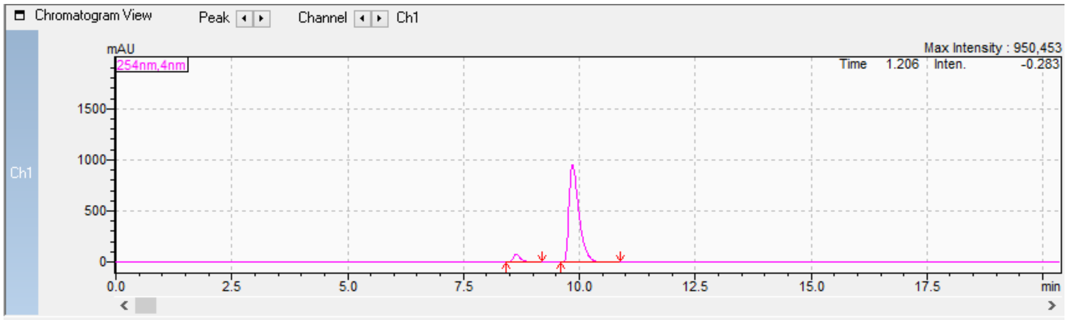

Results View - Peak Table

Peak Table | Compound | Group | Calibration Curve

Peak#	Ret. Time	Area	Height	Mark	Area%
1	8.637	832455	77352		5.572
2	9.854	14108387	950865		94.428
Total		14940842	1028217		100.000

%*ee* = 94.428-5.572 = 88.856 = 89%

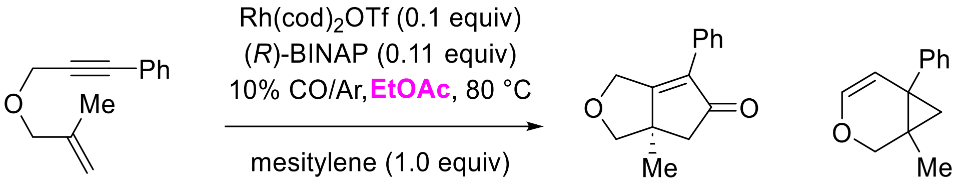

6a **7a** **7ai**

Follows general procedure D. Rh(cod)$_2$OTf (7.1 mg, 0.015 mmol, 0.1 equiv), (*R*)-BINAP (10.3 mg, 0.0165 mmol, 0.11 equiv), mesitylene (18.0 mg, 0.15 mmol, 1.0 equiv) and enyne **6a** (0.5 mL from a stock solution of 0.3 M) in EtOAc (3.0 mL, 0.05 M). After 24 h, PKR yield (40%), b.r.s.m. (53%), starting material remaining (25%) and cycloisomerized side product (0%) were determined by integral comparison to the internal standard mesitylene.

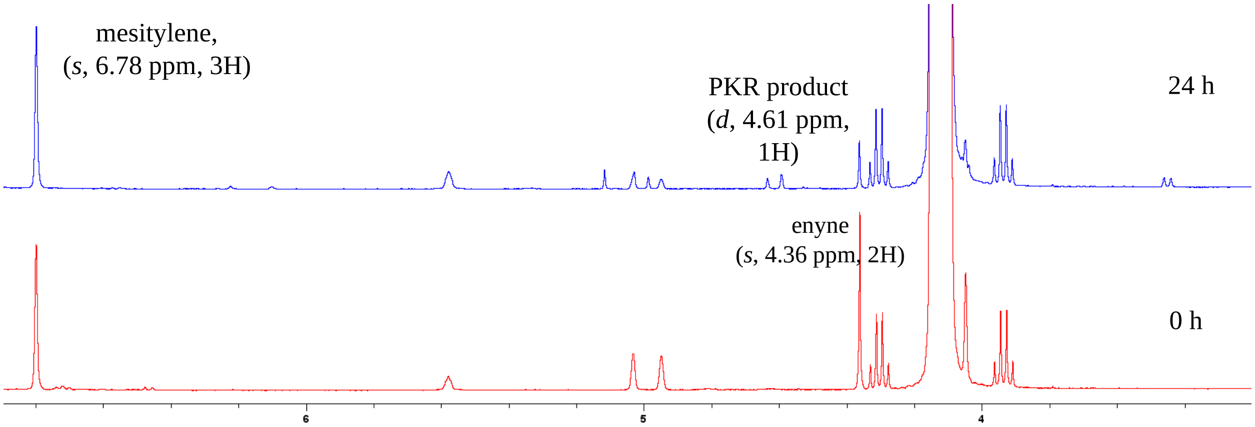

	mesitylene integration (*s*, 6.78 ppm, 3H)	enyne integration(*s*, 4.36 ppm, 2H)	PKR product integration (*d*, 4.61 ppm, 1H)	Cycloisomerized product integration (*d*, 5.21 ppm, 1H)
0 h	1.0000	0.6838	0.0000	0.0000
24 h	1.0000	0.1742	0.1358	0.0000

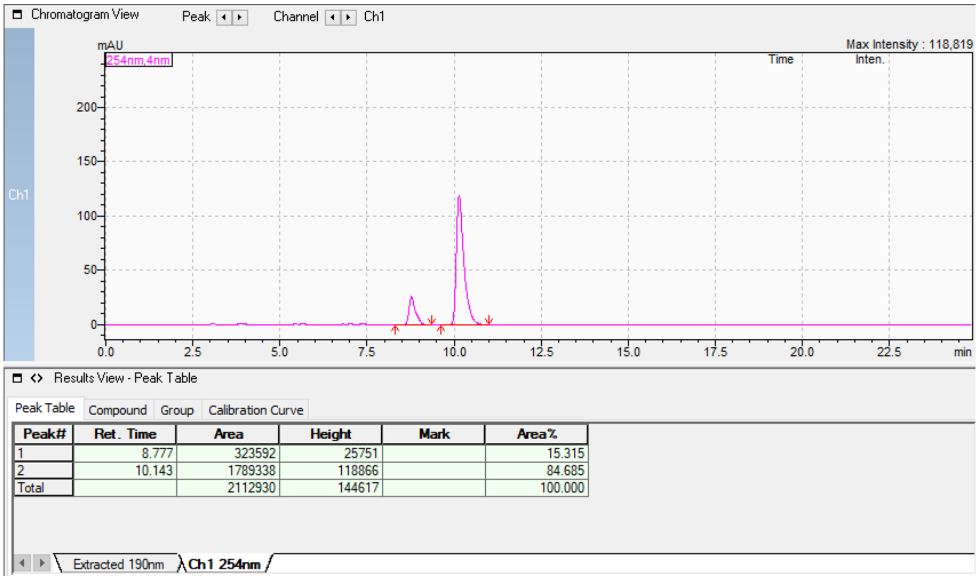

Peak#	Ret. Time	Area	Height	Mark	Area%
1	8.777	323592	25751		15.315
2	10.143	1789338	118866		84.685
Total		2112930	144617		100.000

%*ee*= 84.685-15.315= 69.370 = 69%

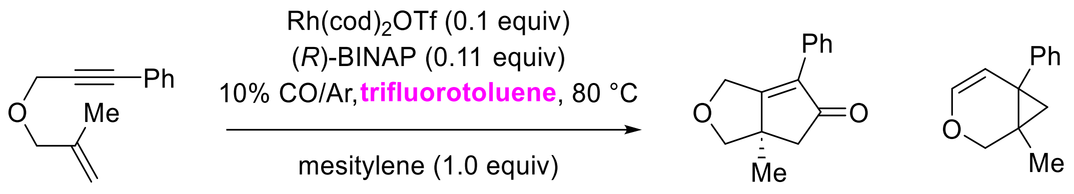

Follows general procedure D. Rh(cod)$_2$OTf (7.1 mg, 0.015 mmol, 0.1 equiv), (*R*)-BINAP (10.3 mg, 0.0165 mmol, 0.11 equiv), mesitylene (18.0 mg, 0.15 mmol, 1.0 equiv) and enyne **6a** (0.5 mL from a stock solution of 0.3 M) in trifluorotoluene (3.0 mL, 0.05 M). After 24 h, PKR yield (26%), b.r.s.m. (55%), starting material remaining (53%) and cycloisomerized side product (15%) were determined by integral comparison to the internal standard mesitylene.

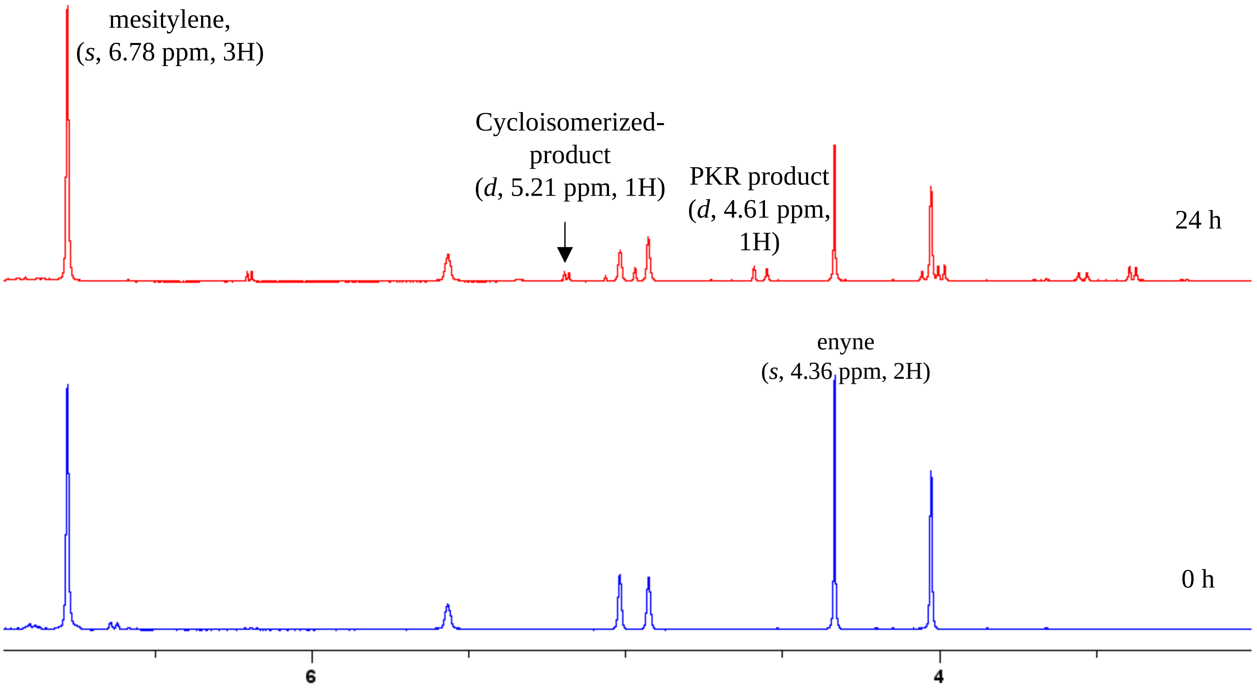

	mesitylene integration (*s*, 6.78 ppm, 3H)	enyne integration(*s*, 4.36 ppm, 2H)	PKR product integration (*d*, 4.61 ppm, 1H)	Cycloisomerized product integration (*d*, 5.21 ppm, 1H)
0 h	1.0000	0.6114	0.0000	0.0000
24 h	1.0000	0.3250	0.0790	0.0448

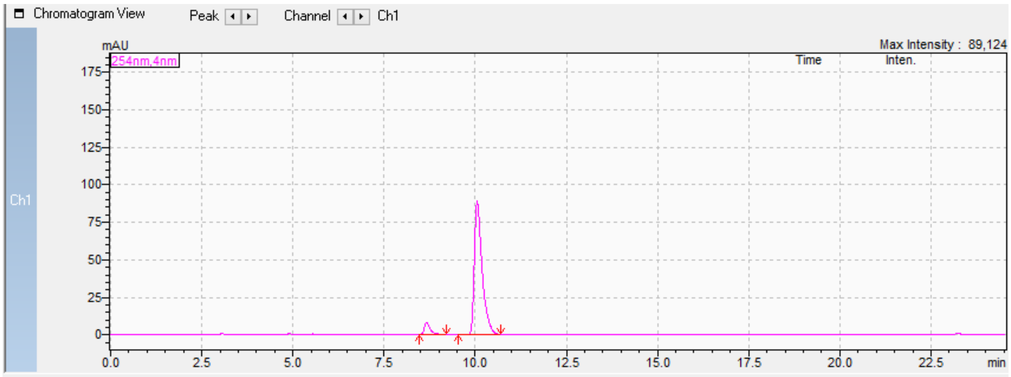

Results View - Peak Table

Peak Table | Compound | Group | Calibration Curve

Peak#	Ret. Time	Area	Height	Mark	Area%
1	8.669	94646	7857	V	6.658
2	10.051	1326875	88917		93.342
Total		1421521	96774		100.000

Extracted 190nm | Ch1 254nm

%*ee* = 93.342 – 6.658 = 86.684 = 87%

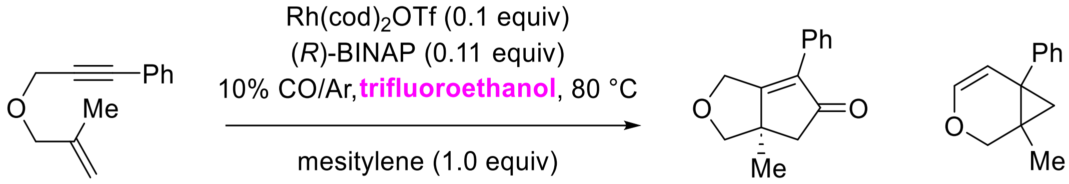

6a **7a** **7ai**

Follows general procedure D. Rh(cod)$_2$OTf (7.1 mg, 0.015 mmol, 0.1 equiv), (*R*)-BINAP (10.3 mg, 0.0165 mmol, 0.11 equiv), mesitylene (18.0 mg, 0.15 mmol, 1.0 equiv) and enyne **6a** (0.5 mL from a stock solution of 0.3 M) in trifluoroethanol (3.0 mL, 0.05 M). After 24 h, PKR yield (80%), b.r.s.m. (81%), starting material remaining (trace) and cycloisomerized side product (0%) were determined by integral comparison to the internal standard mesitylene.

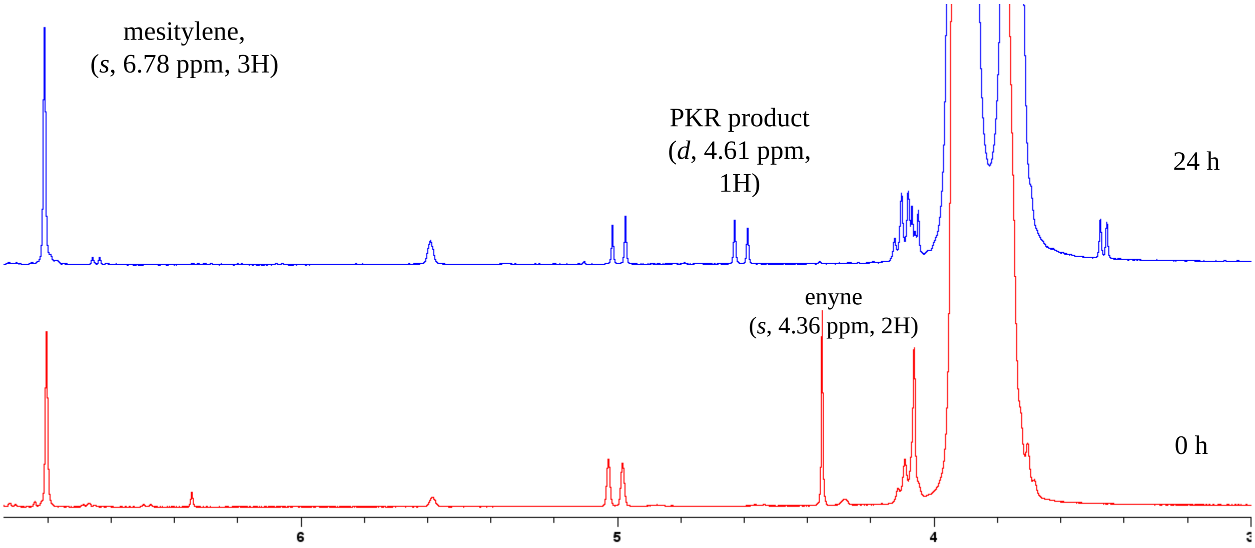

	mesitylene integration (*s*, 6.78 ppm, 3H)	enyne integration(*s*, 4.36 ppm, 2H)	PKR product integration (*d*, 4.61 ppm, 1H)	Cycloisomerized product integration (*d*, 5.21 ppm, 1H)
0 h	1.0000	0.7484	0.0000	0.0000
24 h	1.0000	trace	0.2981	0.0000

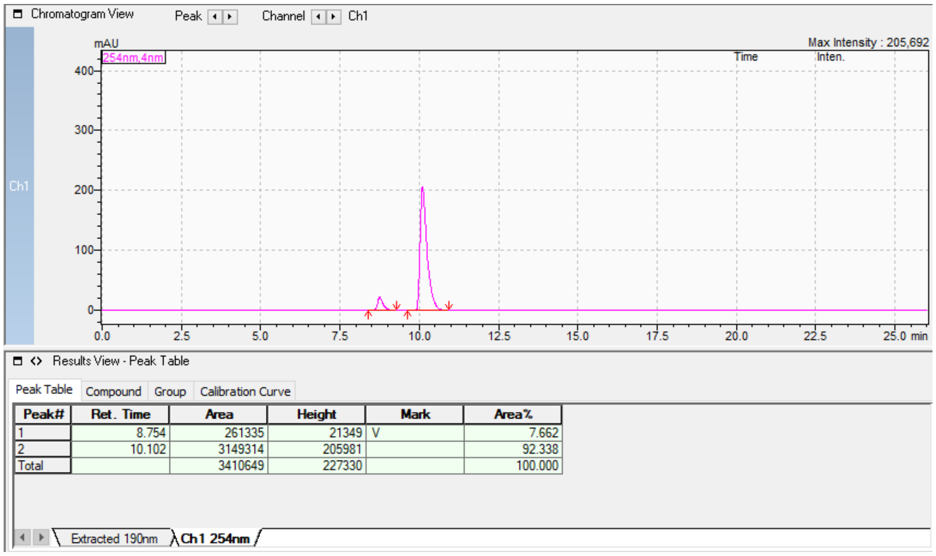

Peak#	Ret. Time	Area	Height	Mark	Area%
1	8.754	261335	21349	V	7.662
2	10.102	3149314	205981		92.338
Total		3410649	227330		100.000

%*ee* = 92.338 – 7.662 = 84.676 = 85%

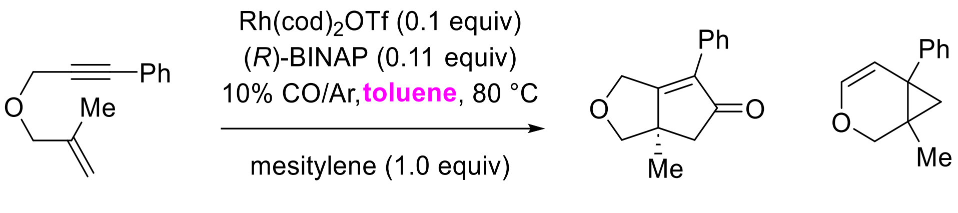

6a **7a** **7ai**

Follows general procedure D. Rh(cod)$_2$OTf (7.1 mg, 0.015 mmol, 0.1 equiv), (*R*)-BINAP (10.3 mg, 0.0165 mmol, 0.11 equiv), mesitylene (18.0 mg, 0.15 mmol, 1.0 equiv) and enyne **6a** (0.5 mL from a stock solution of 0.3 M) in toluene (3.0 mL, 0.05 M). After 24 h, PKR yield (0%), b.r.s.m. (0%), starting material remaining (89%) and cycloisomerized side product (4%) were determined by integral comparison to the internal standard mesitylene.

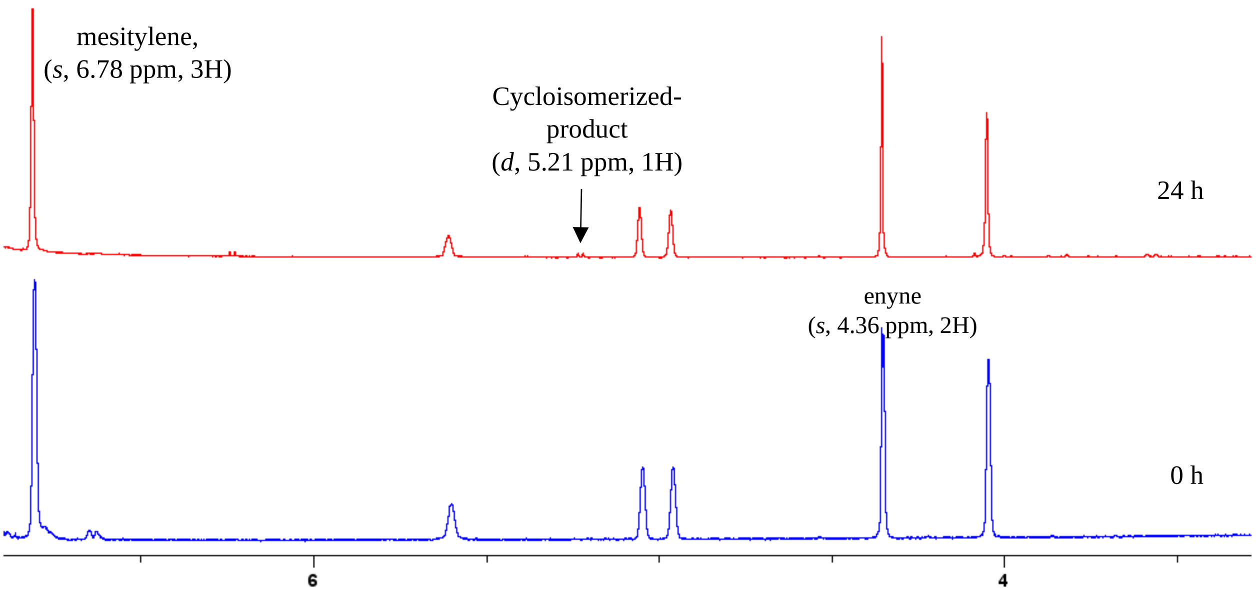

	mesitylene integration (*s*, 6.78 ppm, 3H)	enyne integration(*s*, 4.36 ppm, 2H)	PKR product integration (*d*, 4.61 ppm, 1H)	Cycloisomerized product integration (*d*, 5.21 ppm, 1H)
0 h	1.0000	0.6825	0.0000	0.0000
24 h	1.0000	0.5905	0.0000	0.0120

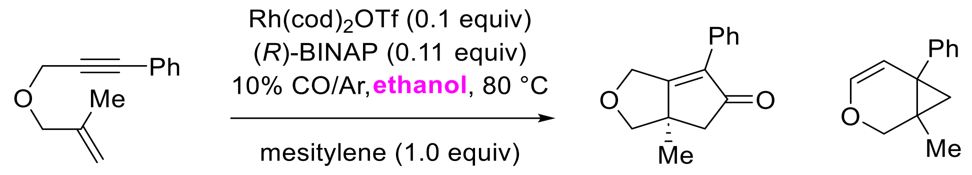

6a **7a** **7ai**

Follows general procedure D. Rh(cod)$_2$OTf (7.1 mg, 0.015 mmol, 0.1 equiv), (*R*)-BINAP (10.3 mg, 0.0165 mmol, 0.11 equiv), mesitylene (18.0 mg, 0.15 mmol, 1.0 equiv) and enyne **6a** (0.5 mL from a stock solution of 0.3 M) in ethanol (3.0 mL, 0.05 M). After 24 h, PKR yield (52%), b.r.s.m. (53%), starting material remaining (2%) and cycloisomerized side product (0%) were determined by integral comparison to the internal standard mesitylene.

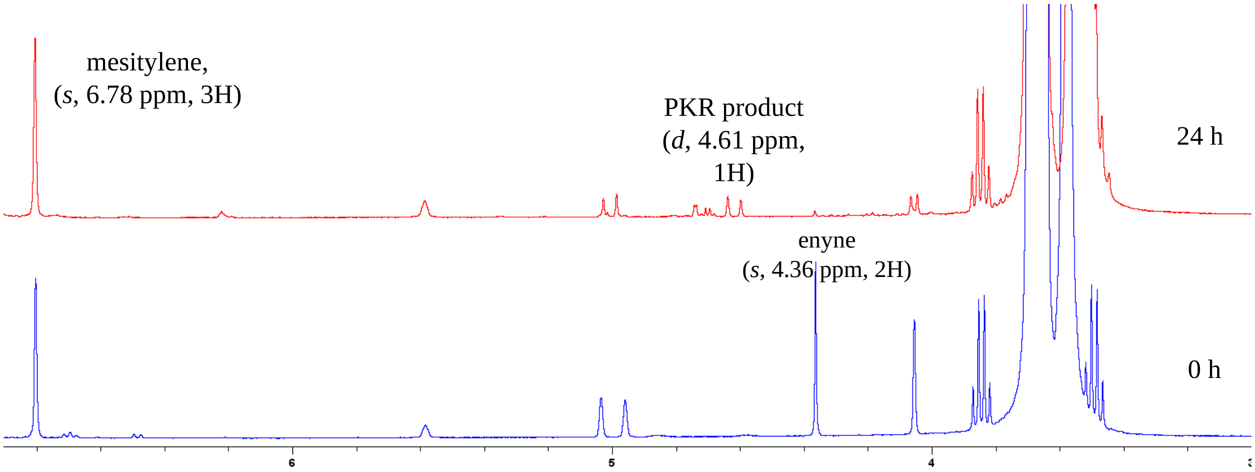

	mesitylene integration (*s*, 6.78 ppm, 3H)	enyne integration(*s*, 4.36 ppm, 2H)	PKR product integration (*d*, 4.61 ppm, 1H)	Cycloisomerized product integration (*d*, 5.21 ppm, 1H)
0 h	1.0000	0.6758	0.0000	0.0000
24 h	1.0000	0.0161	0.1728	0.0000

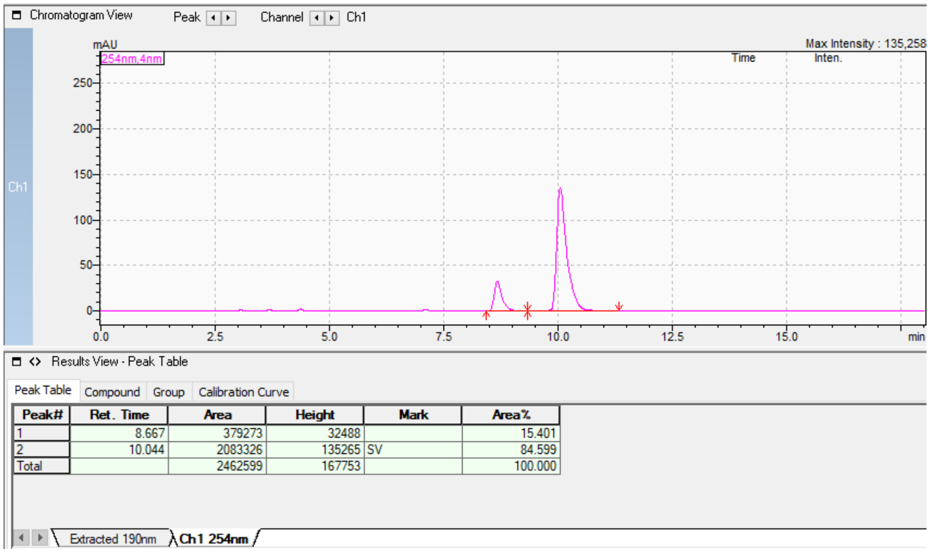

Peak#	Ret. Time	Area	Height	Mark	Area%
1	8.667	379273	32488		15.401
2	10.044	2083326	135265	SV	84.599
Total		2462599	167753		100.000

%*ee* = 84.599 - 15.401= 69.198 = 69%

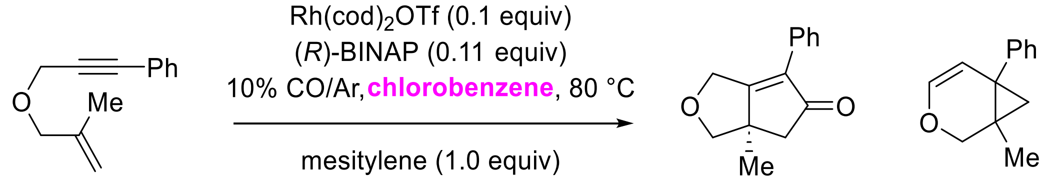

6a **7a** **7ai**

Follows general procedure D. Rh(cod)$_2$OTf (7.1 mg, 0.015 mmol, 0.1 equiv), (*R*)-BINAP (10.3 mg, 0.0165 mmol, 0.11 equiv), mesitylene (18.0 mg, 0.15 mmol, 1.0 equiv) and enyne **6a** (0.5 mL from a stock solution of 0.3 M) in chlorobenzene (3.0 mL, 0.05 M). After 24 h, PKR yield (22%), b.r.s.m. (67%), starting material remaining (67%) and cycloisomerized side product (3%) were determined by integral comparison to the internal standard mesitylene.

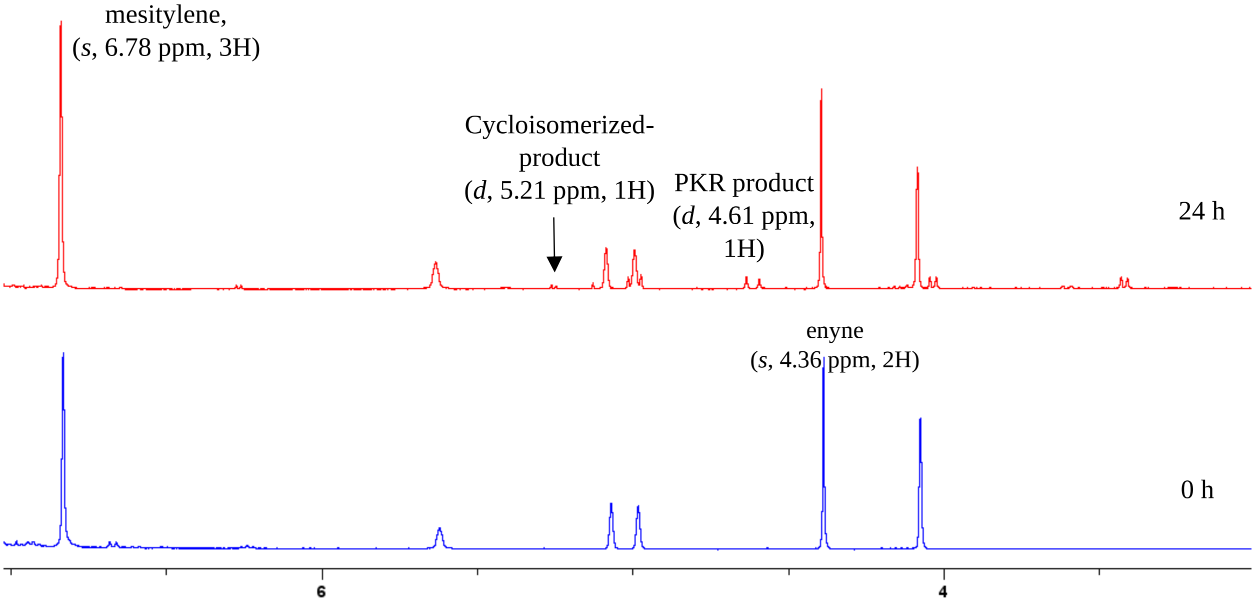

	mesitylene integration (*s*, 6.78 ppm, 3H)	enyne integration(*s*, 4.36 ppm, 2H)	PKR product integration (*d*, 4.61 ppm, 1H)	Cycloisomerized product integration (*d*, 5.21 ppm, 1H)
0 h	1.0000	0.6552	0.0000	0.0000
24 h	1.0000	0.4370	0.0708	0.0107

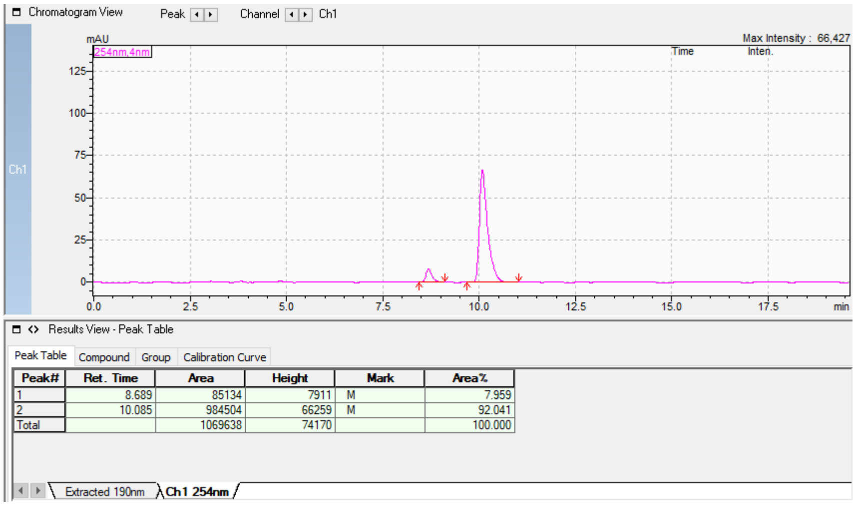

Peak#	Ret. Time	Area	Height	Mark	Area%
1	8.689	85134	7911	M	7.959
2	10.085	984504	66259	M	92.041
Total		1069638	74170		100.000

%*ee* = 92.041 – 7.959= 84.082 = 84%

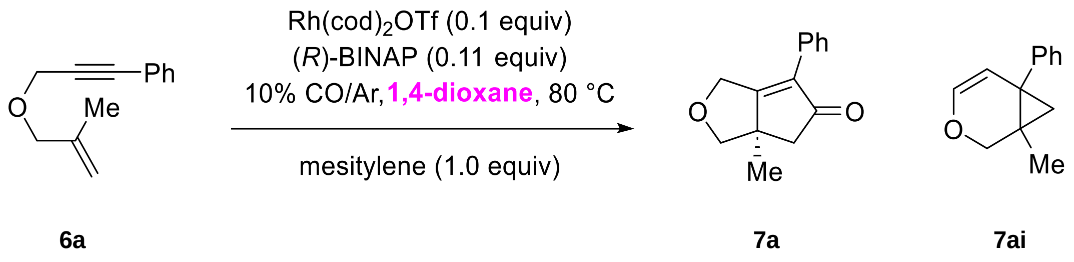

Follows general procedure D. Rh(cod)$_2$OTf (7.1 mg, 0.015 mmol, 0.1 equiv), (*R*)-BINAP (10.3 mg, 0.0165 mmol, 0.11 equiv), mesitylene (18.0 mg, 0.15 mmol, 1.0 equiv) and enyne **6a** (0.5 mL from a stock solution of 0.3 M) in 1,4-dioxane (3.0 mL, 0.05 M). After 24 h, PKR yield (19%), b.r.s.m. (36%), starting material remaining (47%) and cycloisomerized side product (4%) were determined by integral comparison to the internal standard mesitylene.

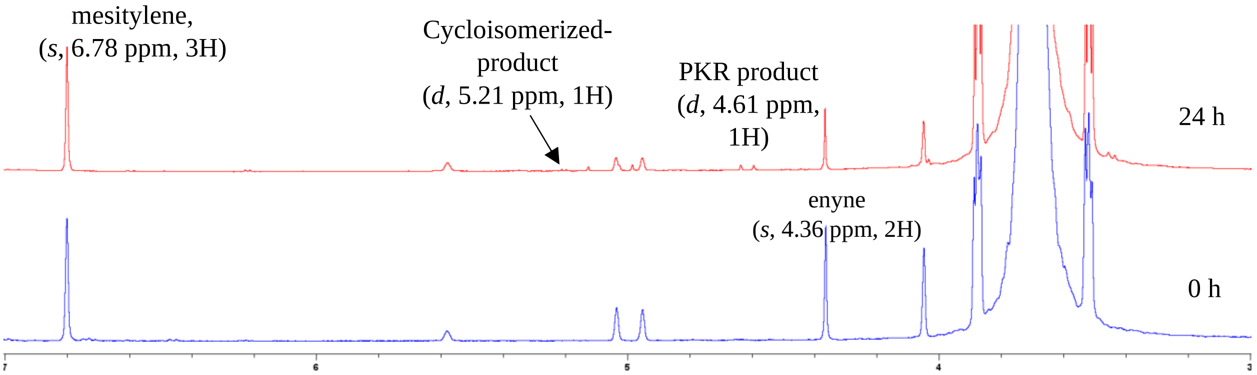

	mesitylene integration (*s*, 6.78 ppm, 3H)	enyne integration(*s*, 4.36 ppm, 2H)	PKR product integration (*d*, 4.61 ppm, 1H)	Cycloisomerized product integration (*d*, 5.21 ppm, 1H)
0 h	1.0000	0.6808	0.0000	0.0000
24 h	1.0000	0.3151	0.0639	0.0145

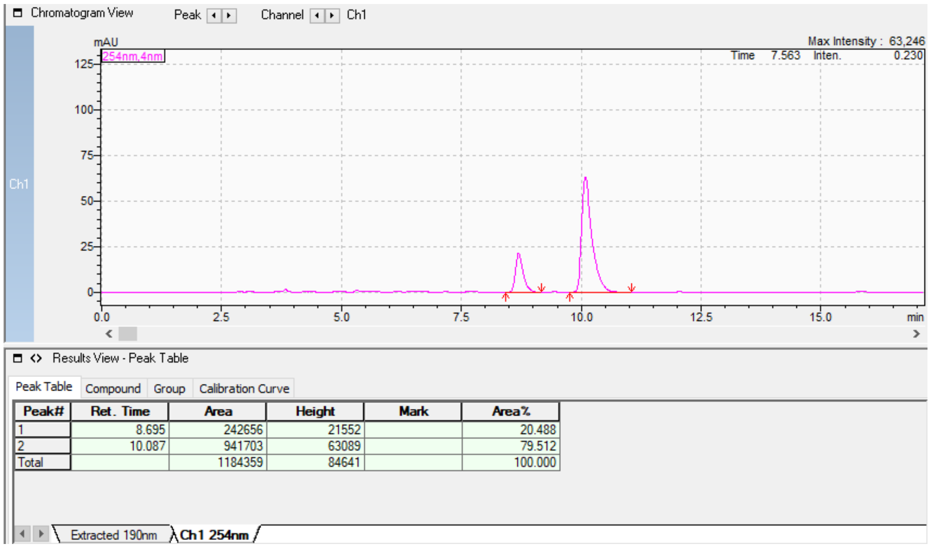

%*ee* = 79.512 – 20.488= 59.024 = 59%

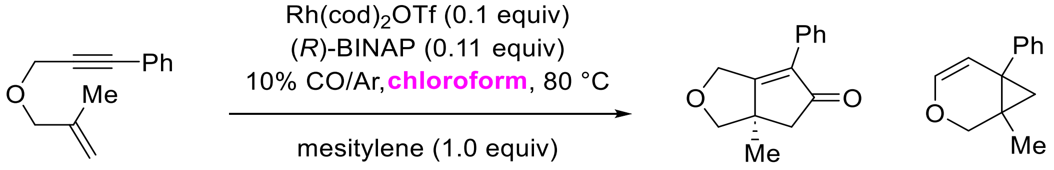

6a **7a** **7ai**

Follows general procedure D. Rh(cod)$_2$OTf (7.1 mg, 0.015 mmol, 0.1 equiv), (*R*)-BINAP (10.3 mg, 0.0165 mmol, 0.11 equiv), mesitylene (18.0 mg, 0.15 mmol, 1.0 equiv) and enyne **6a** (0.5 mL from a stock solution of 0.3 M) in chloroform (3.0 mL, 0.05 M). After 24 h, PKR yield (trace), b.r.s.m. (0%), starting material remaining (76%) and cycloisomerized side product (0%) were determined by integral comparison to the internal standard mesitylene.

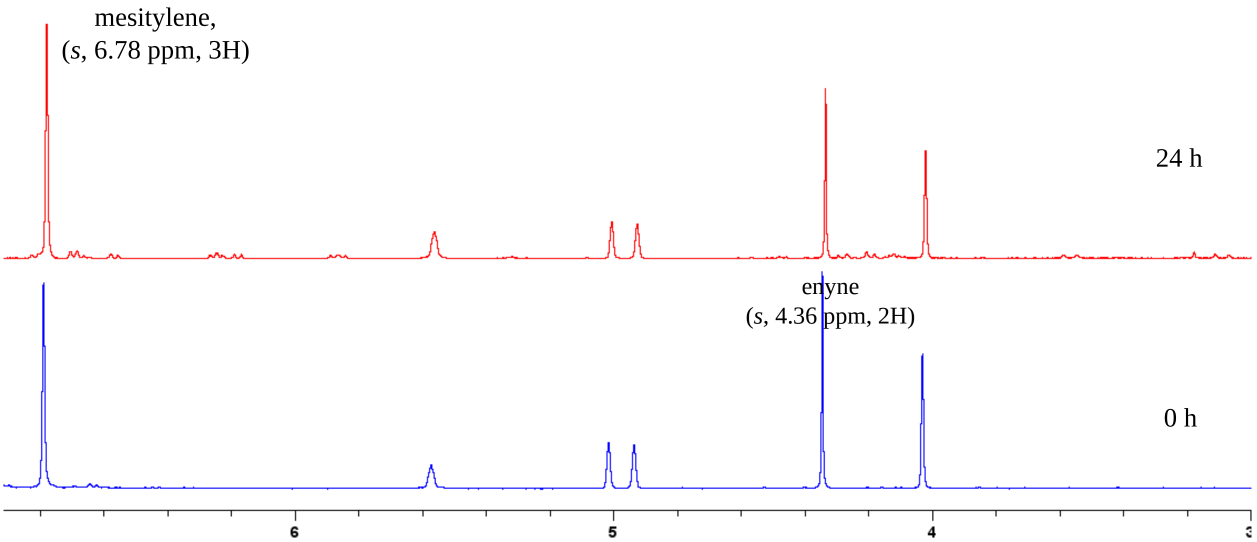

	mesitylene integration (*s*, 6.78 ppm, 3H)	enyne integration(*s*, 4.36 ppm, 2H)	PKR product integration (*d*, 4.61 ppm, 1H)	Cycloisomerized product integration (*d*, 5.21 ppm, 1H)
0 h	1.0000	0.6097	0.0000	0.0000
24 h	1.0000	0.4605	trace	0.0000

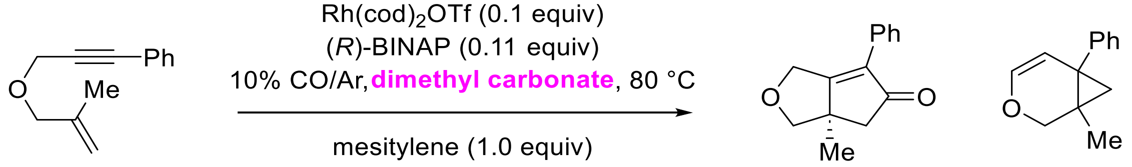

6a **7a** **7ai**

Follows general procedure D. $Rh(cod)_2OTf$ (7.1 mg, 0.015 mmol, 0.1 equiv), (*R*)-BINAP (10.3 mg, 0.0165 mmol, 0.11 equiv), mesitylene (18.0 mg, 0.15 mmol, 1.0 equiv) and enyne **6a** (0.5 mL from a stock solution of 0.3 M) in dimethylcarbonate (3.0 mL, 0.05 M). After 24 h, PKR yield (32%), b.r.s.m. (39%), starting material remaining (17%) and cycloisomerized side product (0%) were determined by integral comparison to the internal standard mesitylene.

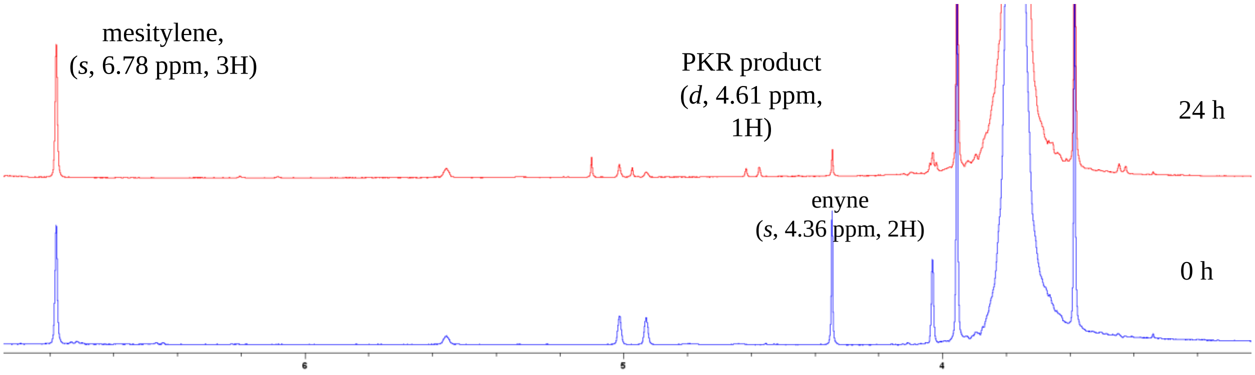

	mesitylene integration (*s*, 6.78 ppm, 3H)	enyne integration(*s*, 4.36 ppm, 2H)	PKR product integration (*d*, 4.61 ppm, 1H)	Cycloisomerized product integration (*d*, 5.21 ppm, 1H)
0 h	1.0000	0.6774	0.0000	0.0000
24 h	1.0000	0.1122	0.1074	0.0000

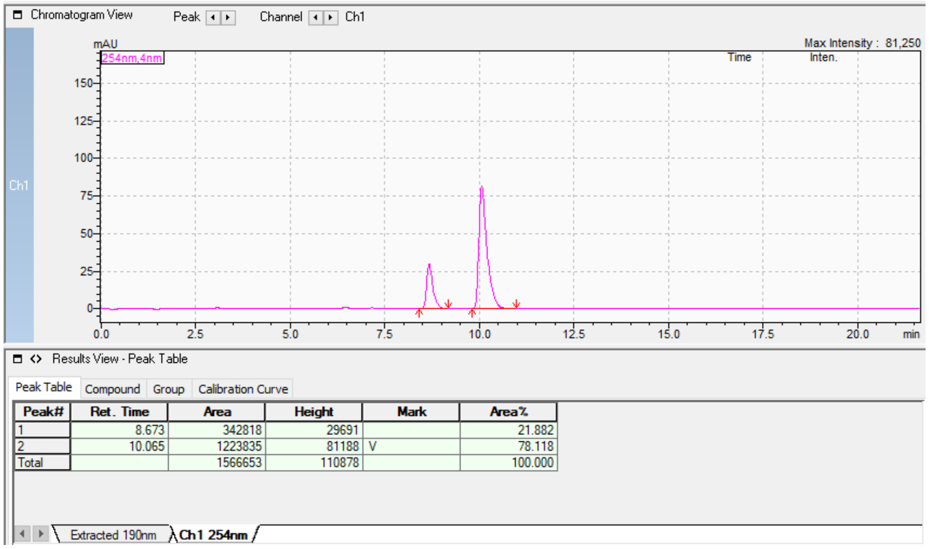

Peak#	Ret. Time	Area	Height	Mark	Area%
1	8.673	342818	29691		21.882
2	10.065	1223835	81188	V	78.118
Total		1566653	110878		100.000

%*ee* = 78.118 – 21.882 = 59.024 = 56.236 = 56%

NMR spectra data for Counterion Study in Table 4

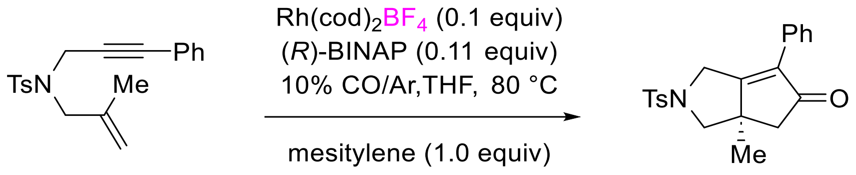

4a **5a**

Table 4, entry 1 (Table S3, entry 2 and Table S7, entry 1)

Entry 1: Follows General Procedure **D** for asymmetric PKR: $Rh(cod)_2BF_4$ (6.1 mg, 0.015 mmol, 0.1 equiv), (*R*)-BINAP (10.3 mg, 0.0165 mmol, 0.11 equiv), mesitylene (18.0 mg, 0.15 mmol, 1.0 equiv) and enyne **4a** (0.5 mL from a stock solution of 0.3 M) in THF (3.0 mL, 0.05 M). The reaction flask was placed in a preheated oil bath (80 °C). After 28 h, the PKR yield (91%), b.r.s.m. (94%) and starting material remaining (3%) were determined by integral comparison to the internal standard mesitylene.

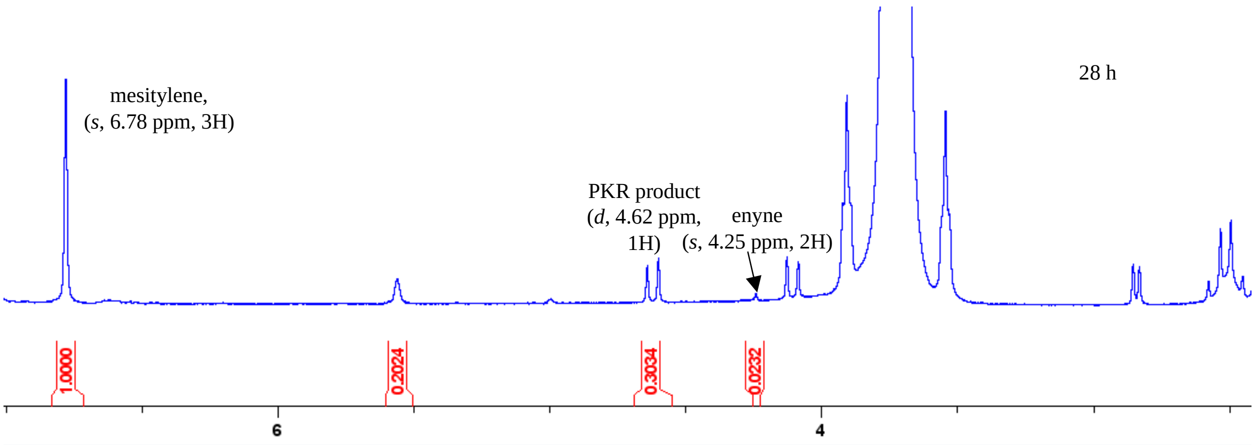

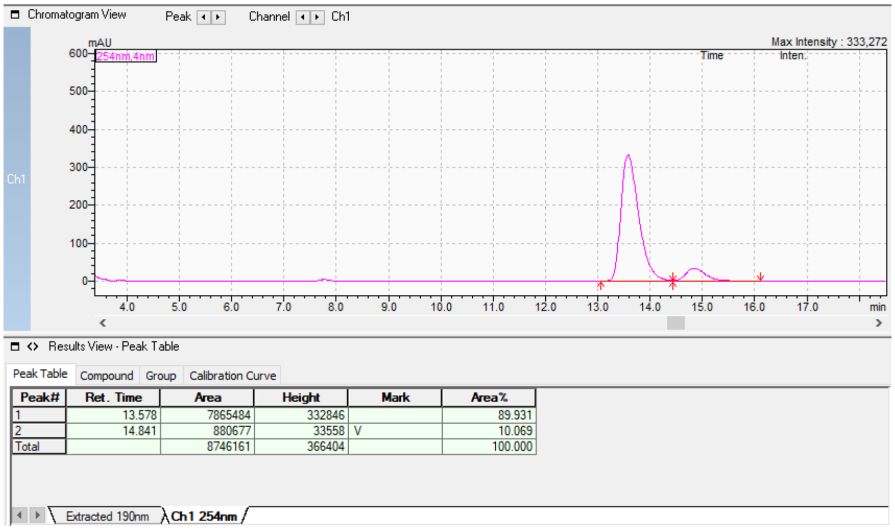

Peak#	Ret. Time	Area	Height	Mark	Area%
1	13.578	7865484	332846		89.931
2	14.841	880677	33558	V	10.069
Total		8746161	366404		100.000

%*ee* = 89.931-10.069 = 79.862 = 80%

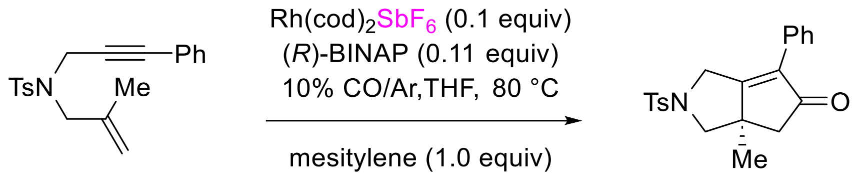

4a **5a**

Table 4, entry 2 and Table S7, entry 3

Follows General Procedure **D** for asymmetric PKR: $Rh(cod)_2SbF_6$ (8.3 mg, 0.015 mmol, 0.1 equiv), (*R*)-BINAP (10.3 mg, 0.0165 mmol, 0.11 equiv), mesitylene (18.0 mg, 0.15 mmol, 1.0 equiv) and enyne **4a** (0.5 mL from a stock solution of 0.3 M) in THF (3.0 mL, 0.05 M). The reaction flask was placed in a preheated oil bath (80 °C). After 36 h, the PKR yield (91%), b.r.s.m. (93%) and starting material remaining (2%) were determined by integral comparison to the internal standard mesitylene.

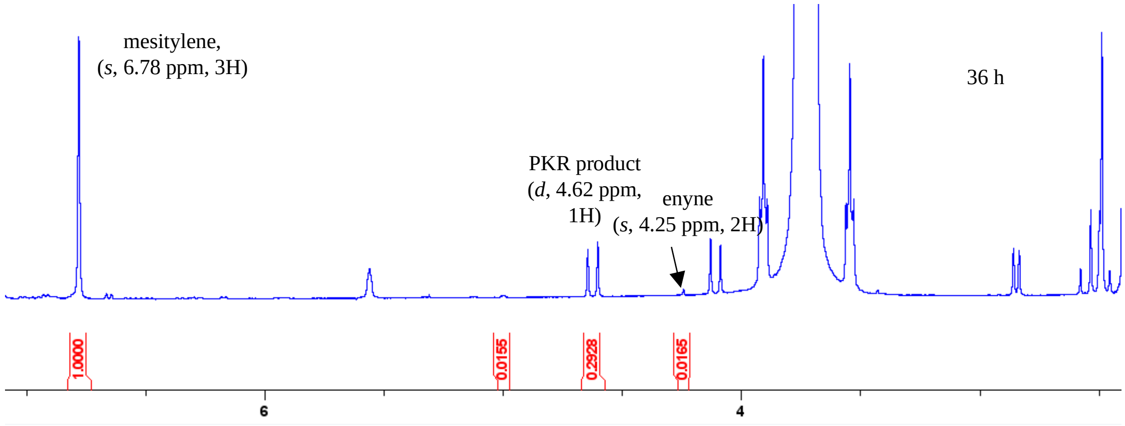

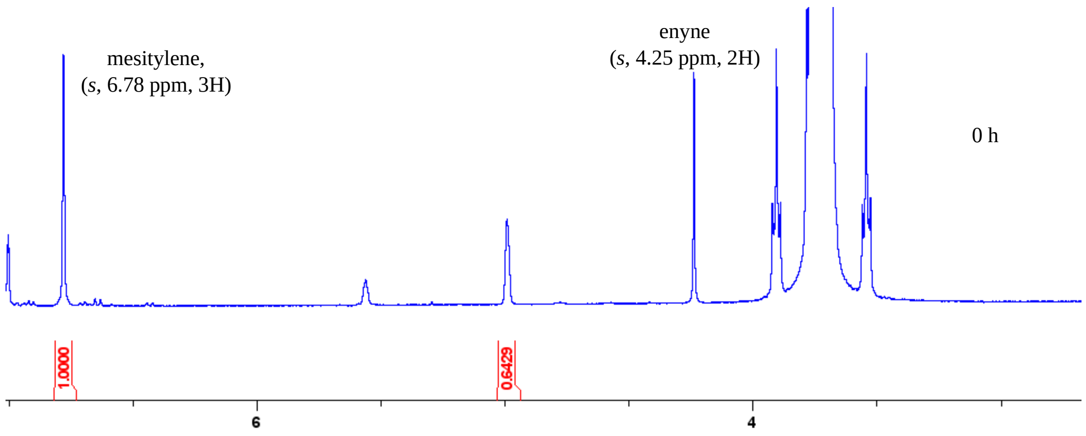

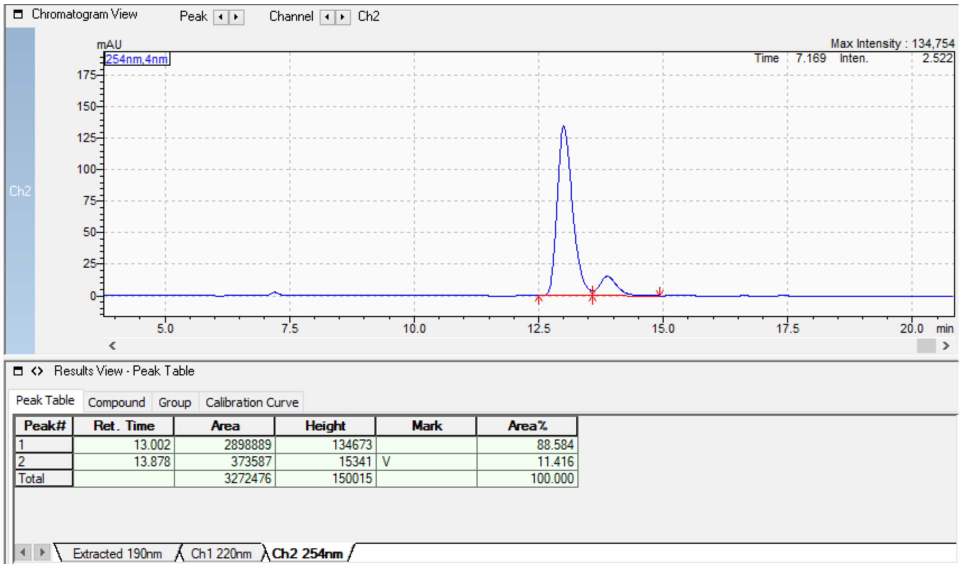

Peak#	Ret. Time	Area	Height	Mark	Area%
1	13.002	2898889	134673		88.584
2	13.878	373587	15341	V	11.416
Total		3272476	150015		100.000

%*ee* = 88.584-11.416 = 77.168 = 77%

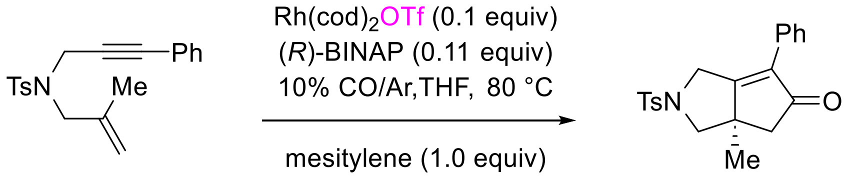

4a **5a**

Table 4, entry 3 (Table 1, entry 1 and Table S7, entry 2 and Table S5, run 1 for the solvent study using THF)

Follows General Procedure **D** for asymmetric PKR: Rh(cod)$_2$OTf (7.1 mg, 0.015 mmol, 0.1 equiv), (*R*)-BINAP (10.3 mg, 0.0165 mmol, 0.11 equiv), mesitylene (18.0 mg, 0.15 mmol, 1.0 equiv) and enyne **4a** (0.5 mL from a stock solution of 0.3 M) in THF (3.0 mL, 0.05 M). The reaction flask was placed in a preheated oil bath (80 °C). After 22 h, the PKR yield (99%), b.r.s.m. (99%) and starting material remaining (0%) were determined by integral comparison to the internal standard mesitylene.

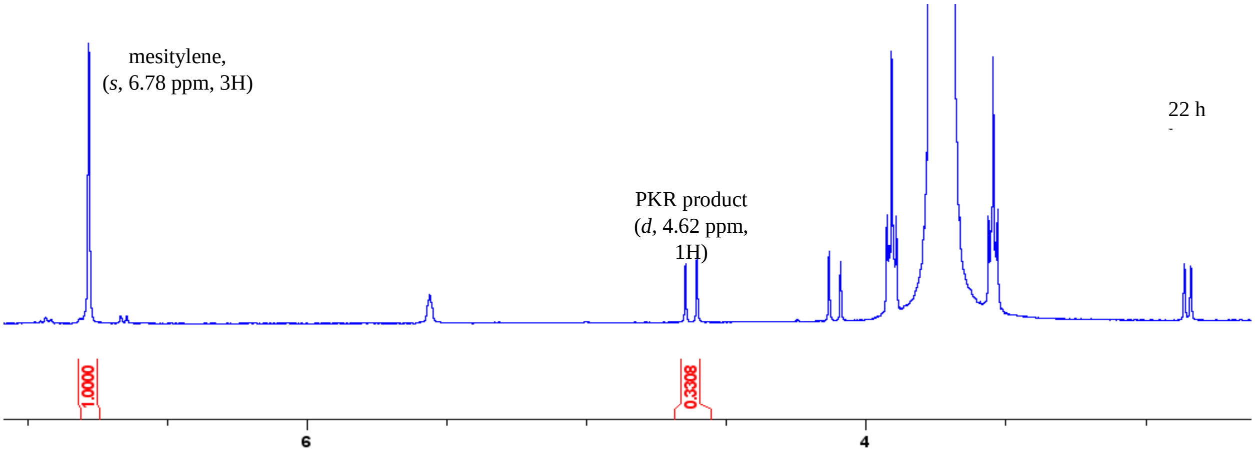

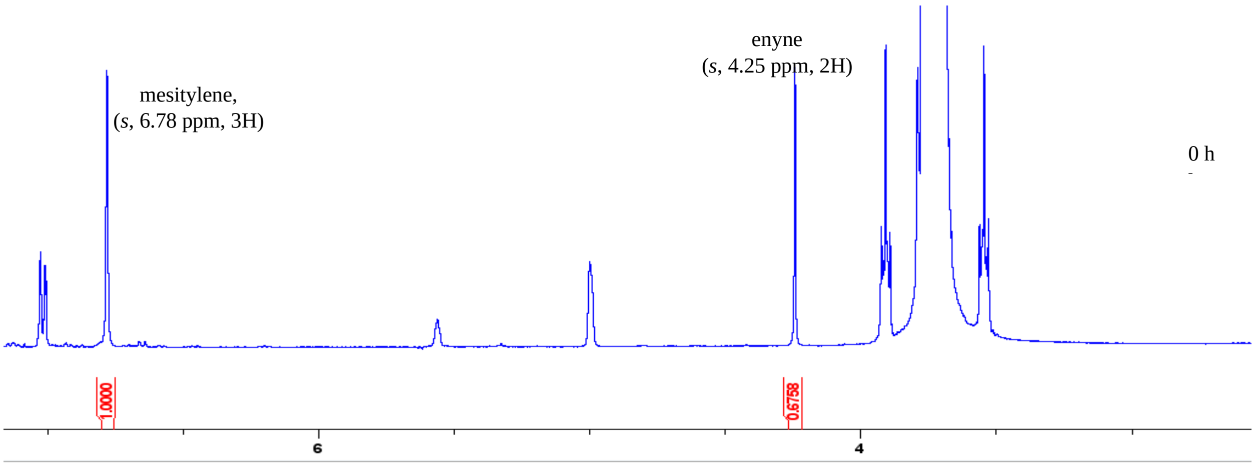

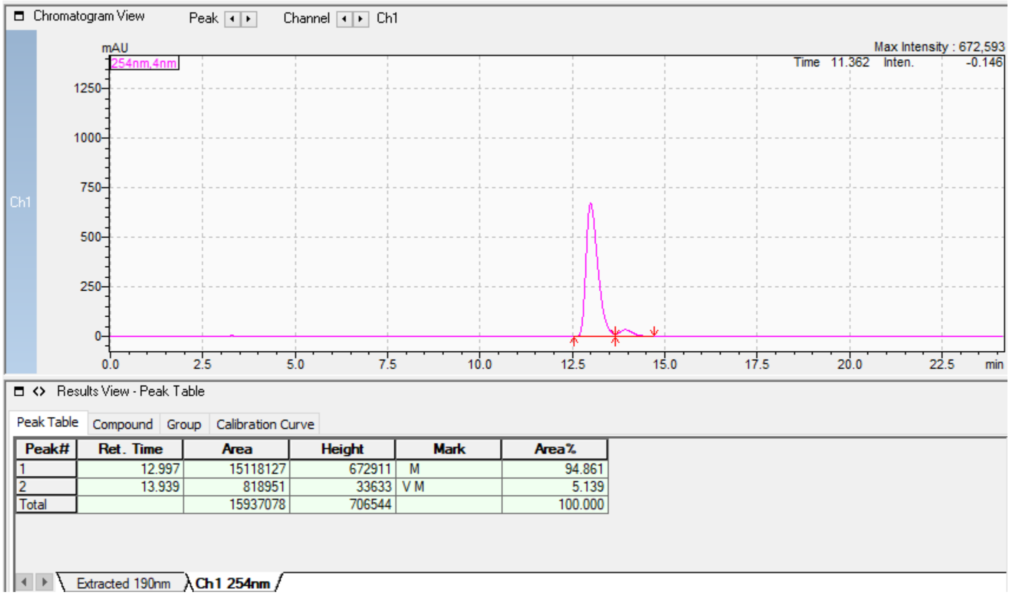

Peak#	Ret. Time	Area	Height	Mark	Area%
1	12.997	15118127	672911	M	94.861
2	13.939	818951	33633	V M	5.139
Total		15937078	706544		100.000

%*ee* = 94.861 – 5.139 = 89.722% = 90%

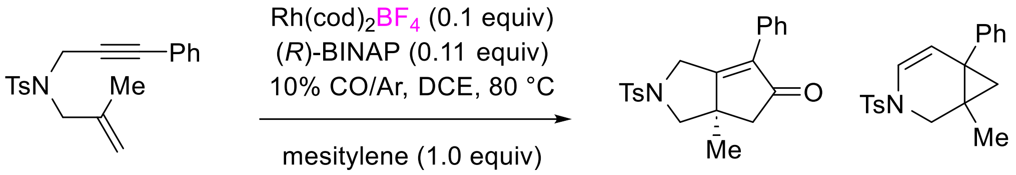

4a **5a** **5ai**

Table 4, **entry 4 (Table S3, entry 1)**

Follows general procedure D. Rh(cod)$_2$BF$_4$ (6.1 mg, 0.015 mmol, 0.1 equiv), (*R*)-BINAP (10.3 mg, 0.0165 mmol, 0.11 equiv), mesitylene (18.0 mg, 0.15 mmol, 1.0 equiv) and enyne **4a** (0.5 mL from a stock solution of 0.3 M) in DCE (3.0 mL, 0.05 M). The reaction flask was placed in a preheated oil bath (80 °C). After 43 h, PKR yield (63%), b.r.s.m. (72%), starting material remaining (13%) and cycloisomerized product (7%) were determined by integral comparison to the internal standard mesitylene.

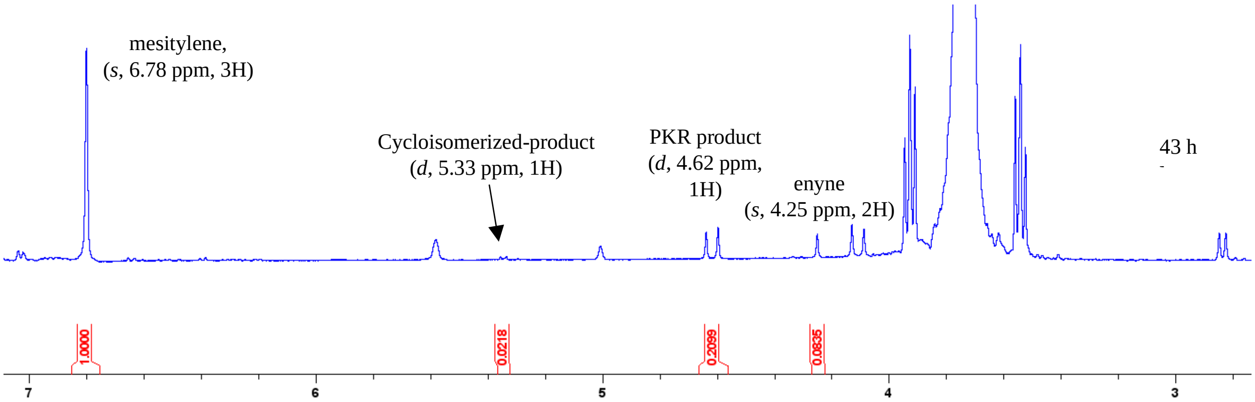

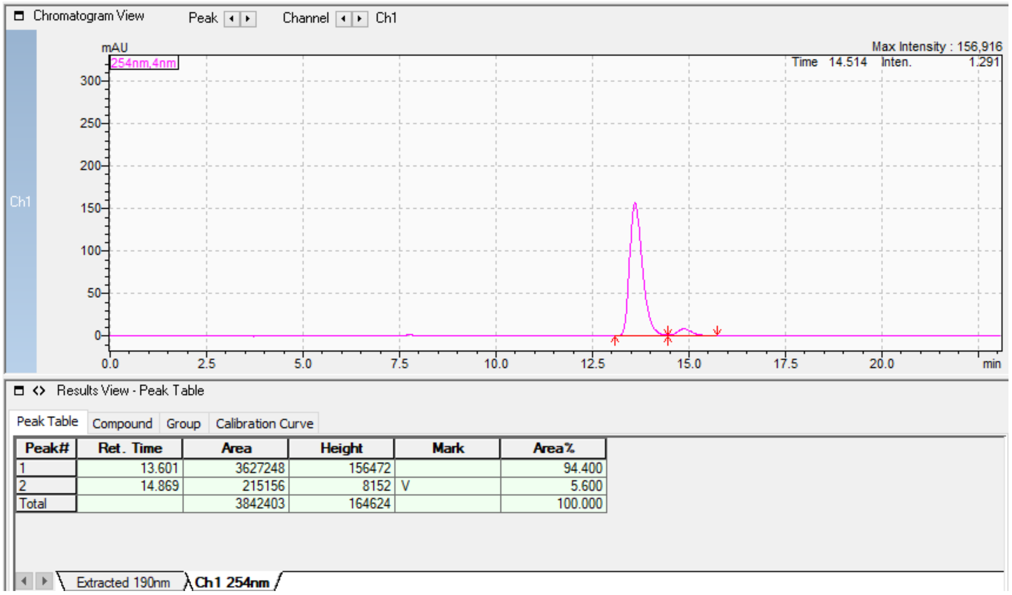

Peak#	Ret. Time	Area	Height	Mark	Area%
1	13.601	3627248	156472		94.400
2	14.869	215156	8152	V	5.600
Total		3842403	164624		100.000

%*ee*= 94.400-5.600 = 88.8% = 89%

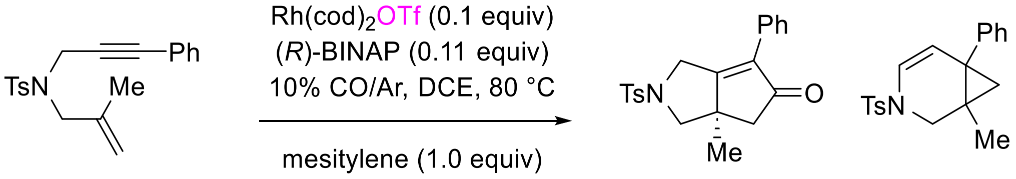

4a **5a** **5ai**

Table 4, entry 5

Follows general procedure D. Rh(cod)$_2$OTf (7.1 mg, 0.015 mmol, 0.1 equiv), (*R*)-BINAP (10.3 mg, 0.0165 mmol, 0.11 equiv), mesitylene (18.0 mg, 0.15 mmol, 1.0 equiv) and enyne **4a** (0.5 mL from a stock solution of 0.3 M) in DCE (3.0 mL, 0.05 M). The reaction flask was placed in a preheated oil bath (80 °C). After 46 h, PKR yield (68%), b.r.s.m. (79%), starting material remaining (14%) and cycloisomerized product (17%) were determined by integral comparison to the internal standard mesitylene.

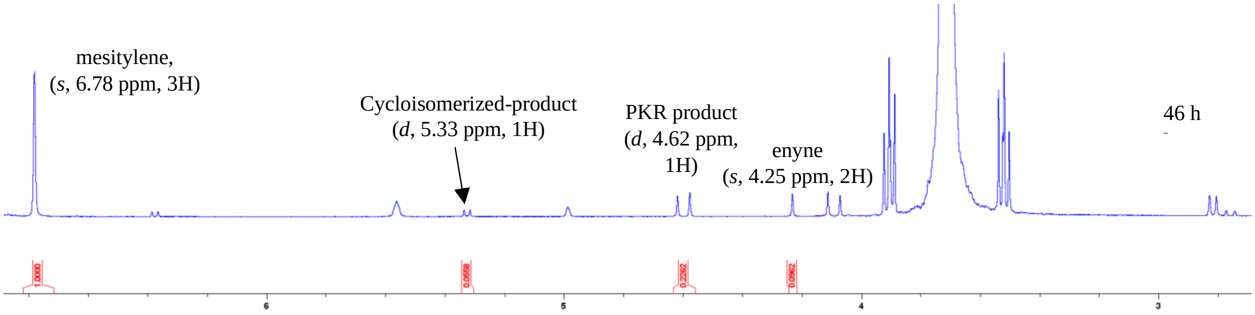

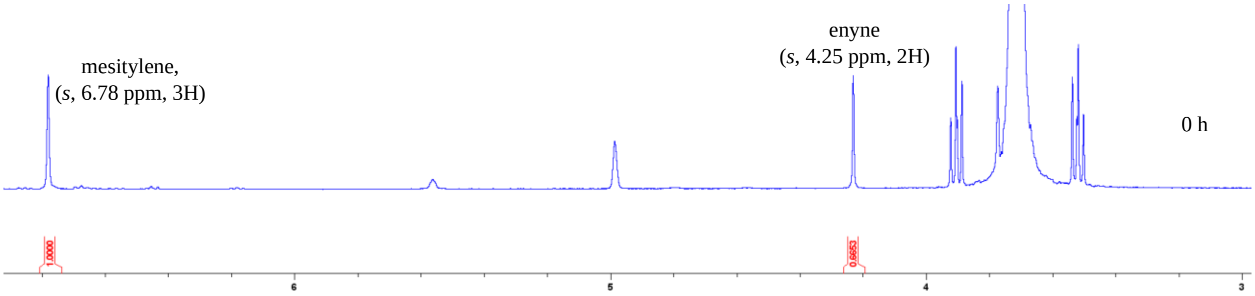

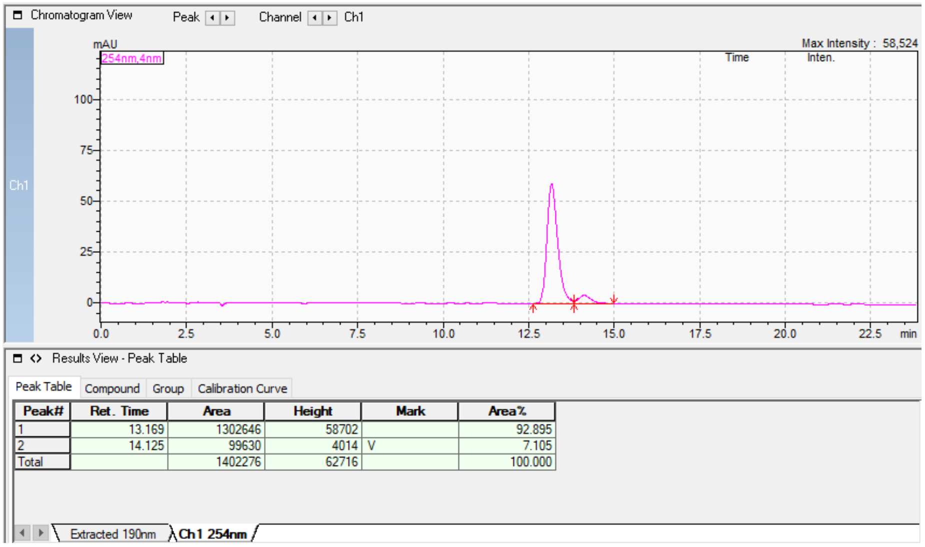

Peak#	Ret. Time	Area	Height	Mark	Area%
1	13.169	1302646	58702		92.895
2	14.125	99630	4014	V	7.105
Total		1402276	62716		100.000

%*ee*= 92.895-7.105 = 85.79% = 86%

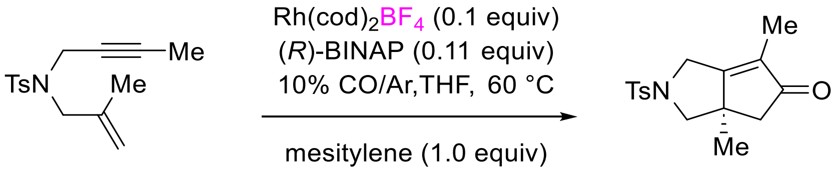

4b **5b**

Table 4, entry 6

Follows general procedure D. Rh(cod)$_2$BF$_4$ (6.1 mg, 0.015 mmol, 0.1 equiv), (*R*)-BINAP (10.3 mg, 0.0165 mmol, 0.11 equiv), mesitylene (18.0 mg, 0.15 mmol, 1.0 equiv) and enyne **4b** (0.5 mL from a stock solution of 0.3 M) in THF (3.0 mL, 0.05 M). The reaction flask was placed in a preheated oil bath (60 °C). After 28 h, PKR yield (44%), b.r.s.m. (55%), starting material remaining (20%) were determined by integral comparison to the internal standard mesitylene.

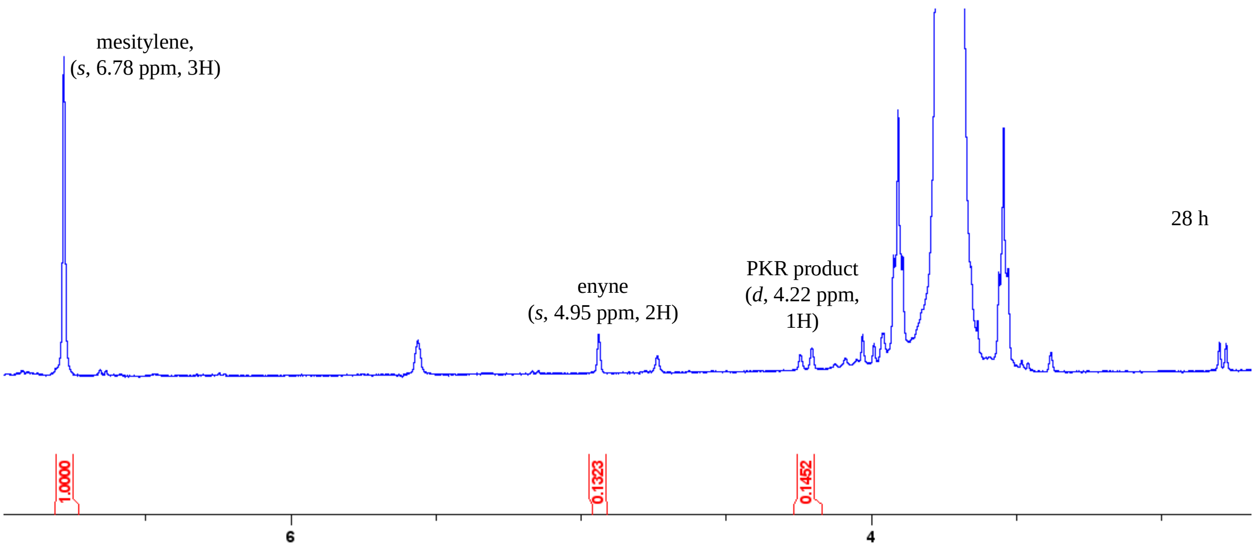

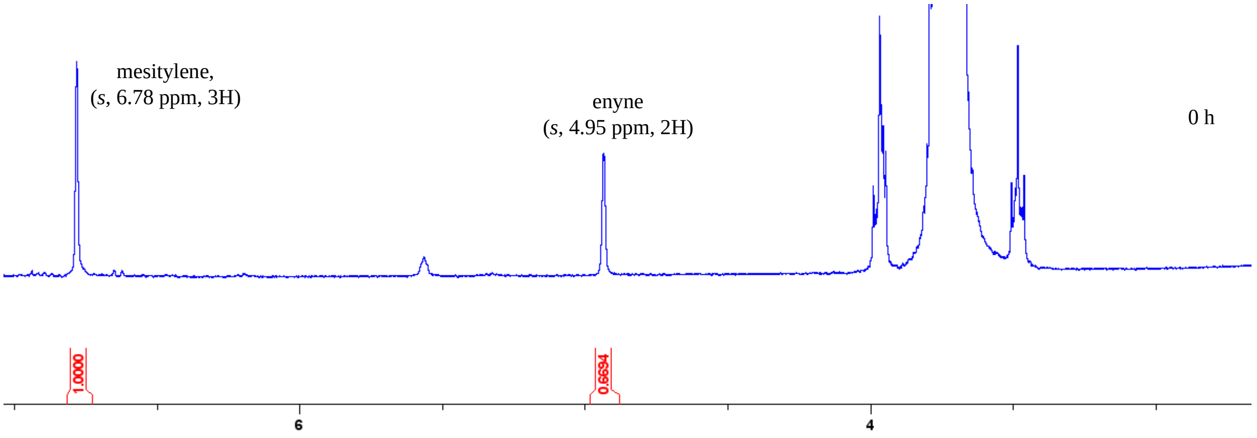

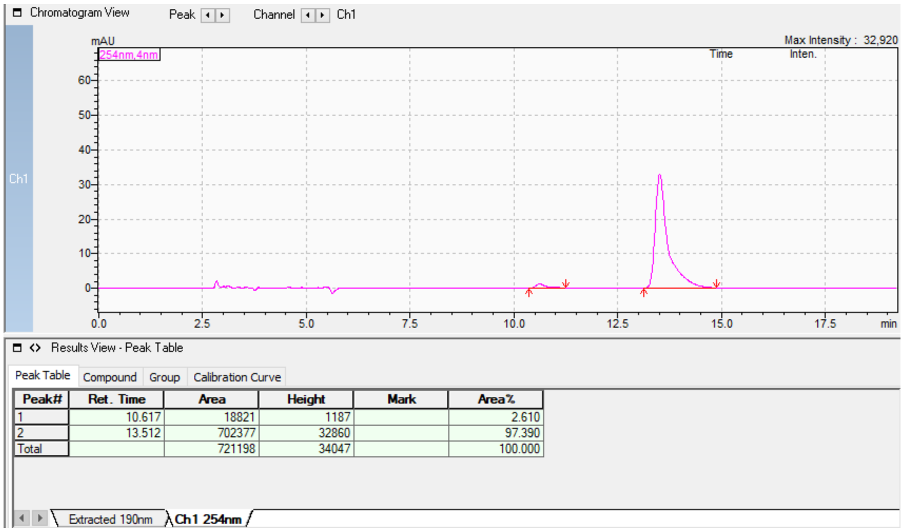

Peak#	Ret. Time	Area	Height	Mark	Area%
1	10.617	18821	1187		2.610
2	13.512	702377	32860		97.390
Total		721198	34047		100.000

%*ee*= 97.390-2.610 = 94.78% = 95%

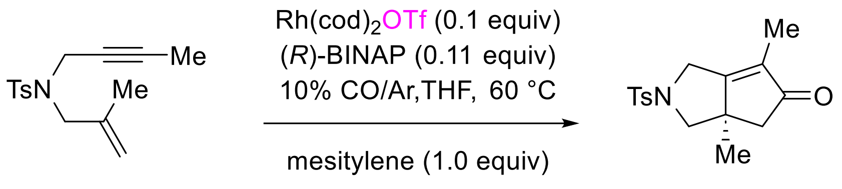

4b **5b**

Table 4, entry 7

Follows general procedure D. Rh(cod)$_2$OTf (7.1 mg, 0.015 mmol, 0.1 equiv), (*R*)-BINAP (10.3 mg, 0.0165 mmol, 0.11 equiv), mesitylene (18.0 mg, 0.15 mmol, 1.0 equiv) and enyne **4b** (0.5 mL from a stock solution of 0.3 M) in THF (3.0 mL, 0.05 M). The reaction flask was placed in a preheated oil bath (60 °C). After 20 h, PKR yield (71%), b.r.s.m. (86%), starting material remaining (17%) were determined by integral comparison to the internal standard mesitylene.

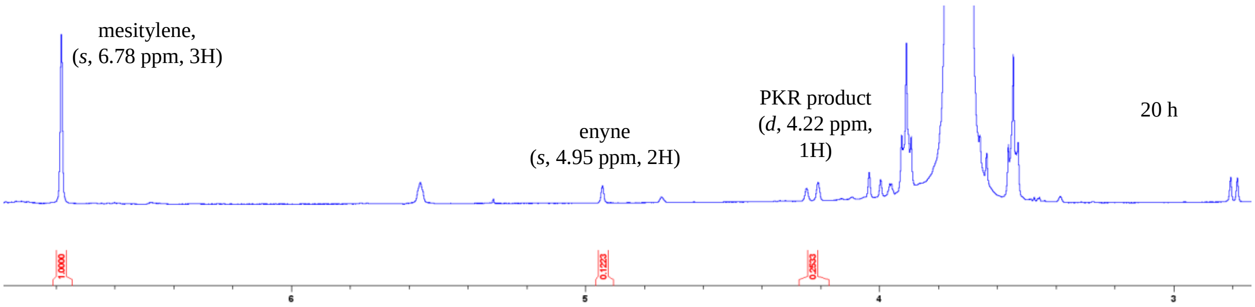

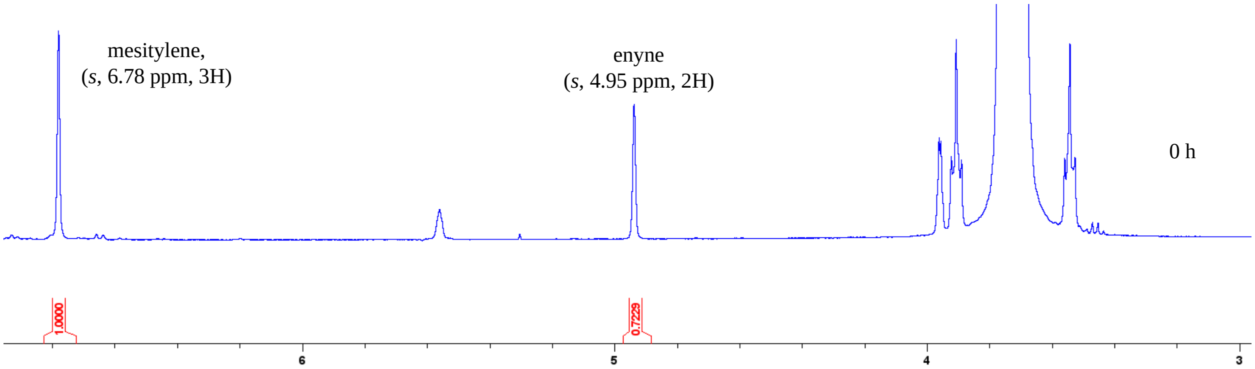

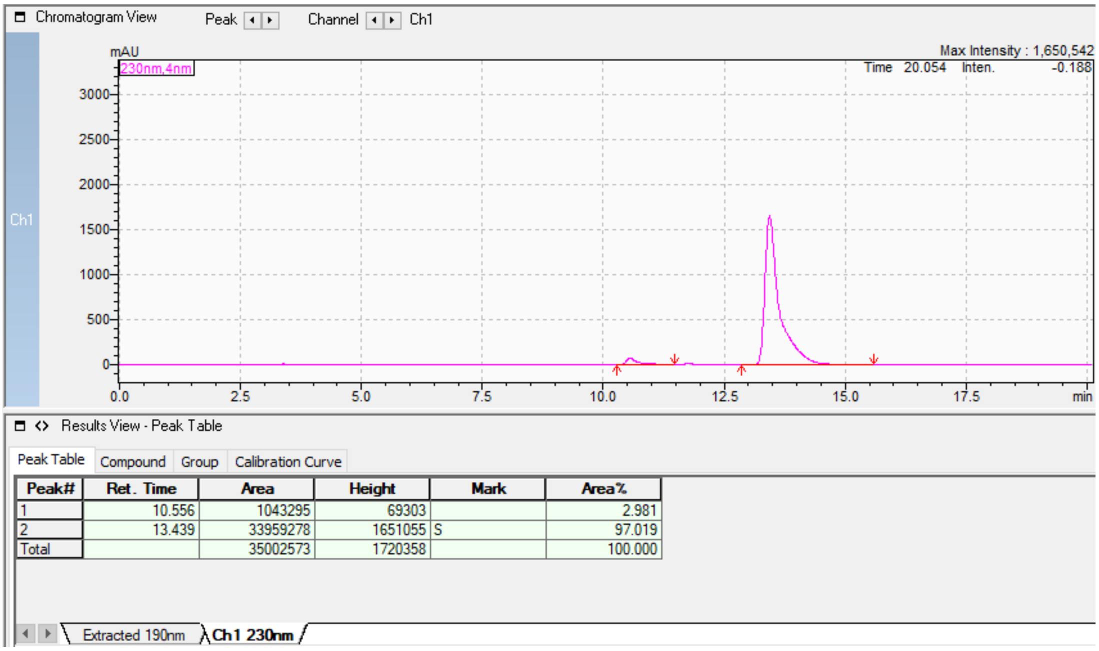

Peak#	Ret. Time	Area	Height	Mark	Area%
1	10.556	1043295	69303		2.981
2	13.439	33959278	1651055	S	97.019
Total		35002573	1720358		100.000

%*ee*= 97.019-2.981 = 94.038% = 94%

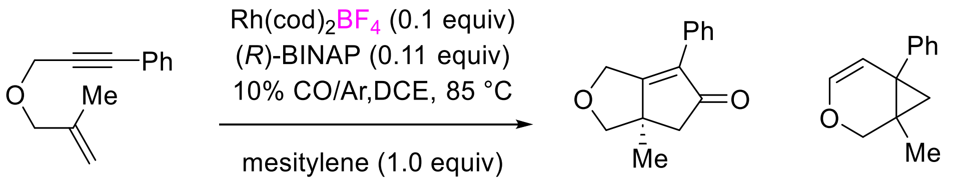

6a **7a** **7ai**

Table 4, **entry 8**

Follows general procedure D. $Rh(cod)_2BF_4$ (6.1 mg, 0.015 mmol, 0.1 equiv), (*R*)-BINAP (10.3 mg, 0.0165 mmol, 0.11 equiv), mesitylene (18.0 mg, 0.15 mmol, 1.0 equiv) and enyne **6a** (0.5 mL from a stock solution of 0.3 M) in DCE (3.0 mL, 0.05 M). The reaction flask was placed in a preheated oil bath (85 °C). After 40 h, PKR yield (54%), b.r.s.m. (54%), starting material remaining (0%) and cycloisomerized side product (31%) were determined by integral comparison to the internal standard mesitylene.

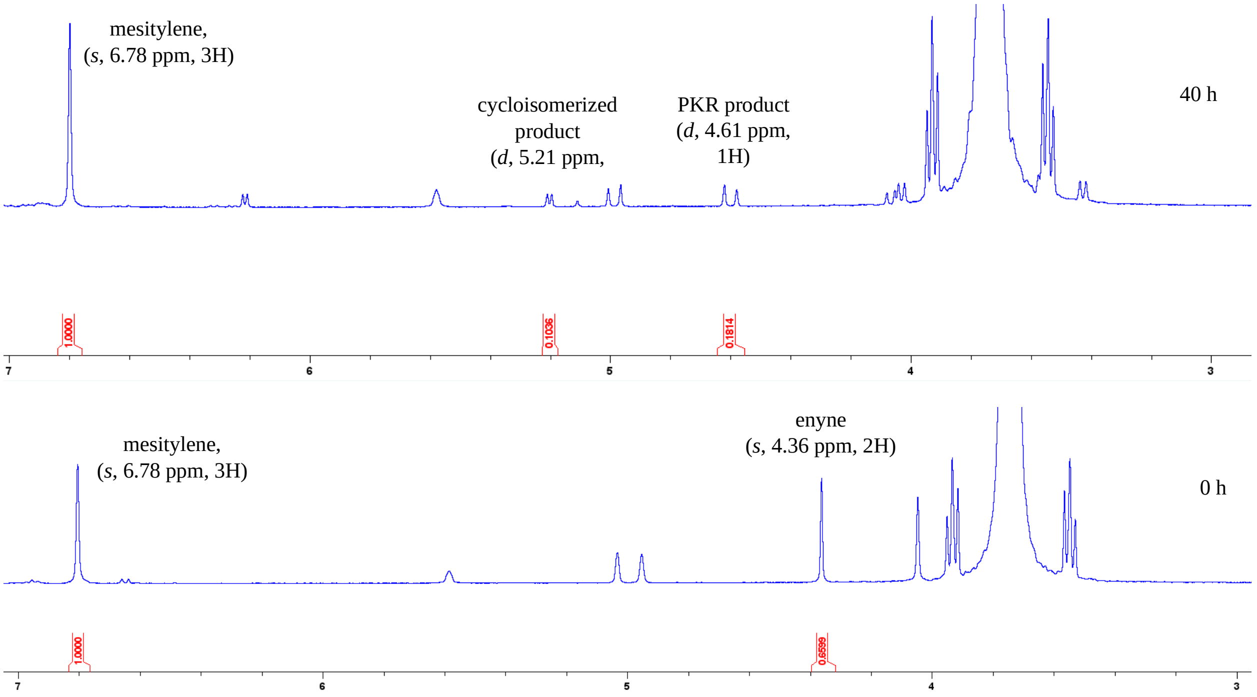

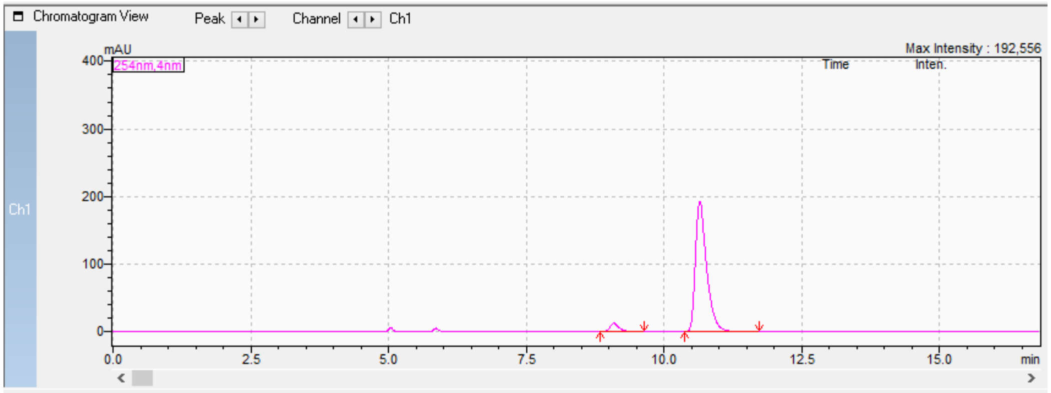

Results View - Peak Table

Peak Table | Compound | Group | Calibration Curve

Peak#	Ret. Time	Area	Height	Mark	Area%
1	9.093	136606	12326		4.764
2	10.653	2731140	192478		95.236
Total		2867746	204804		100.000

Extracted 190nm | Ch1 254nm

% *ee* = 95.236-4.764 = 90.472 = 90%

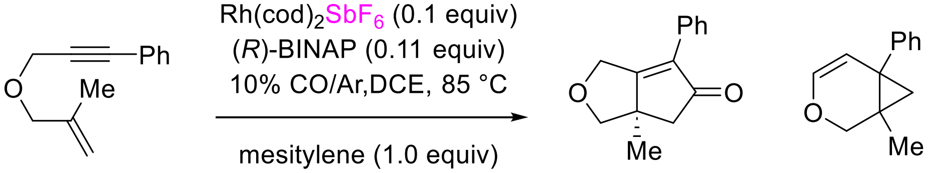

6a **7a** **7ai**

Table 4, entry 9

Follows general procedure D. $Rh(cod)_2SbF_6$ (8.3 mg, 0.015 mmol, 0.1 equiv), (*R*)-BINAP (10.3 mg, 0.0165 mmol, 0.11 equiv), mesitylene (18.0 mg, 0.15 mmol, 1.0 equiv) and enyne **6a** (0.5 mL from a stock solution of 0.3 M) in DCE (3.0 mL, 0.05 M). The reaction flask was placed in a preheated oil bath (85 °C). After 19 h, PKR yield (85%), b.r.s.m. (85%), starting material remaining (trace) and cycloisomerized side product (6%) were determined by integral comparison to the internal standard mesitylene.

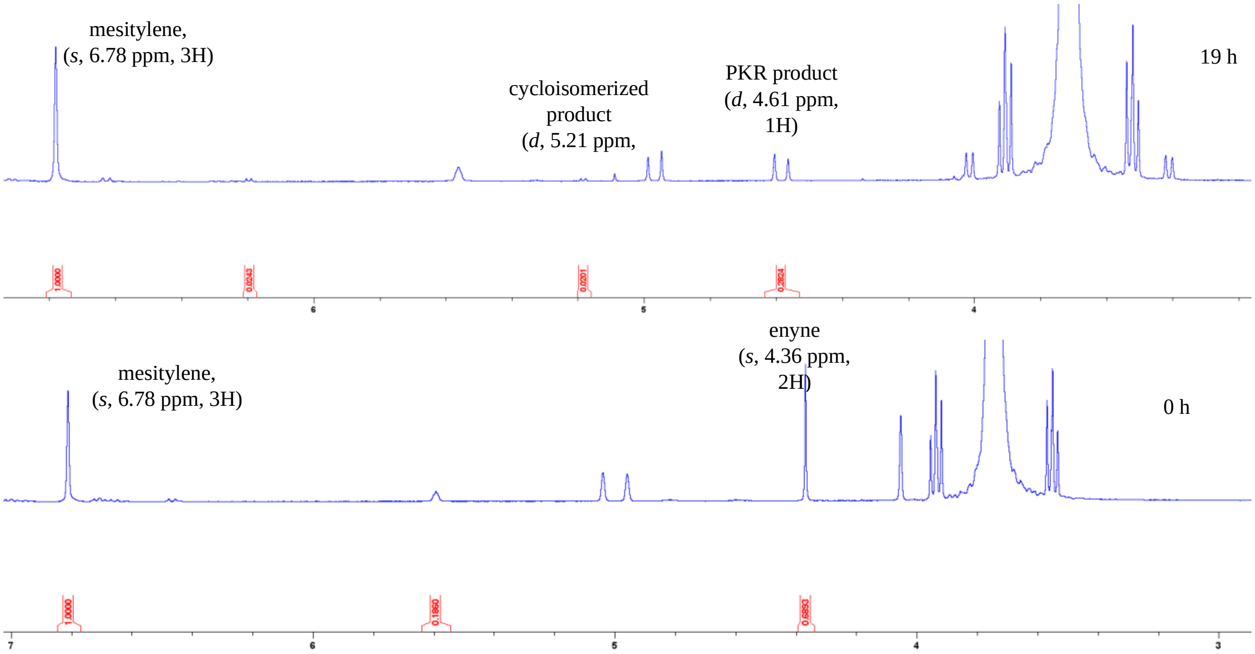

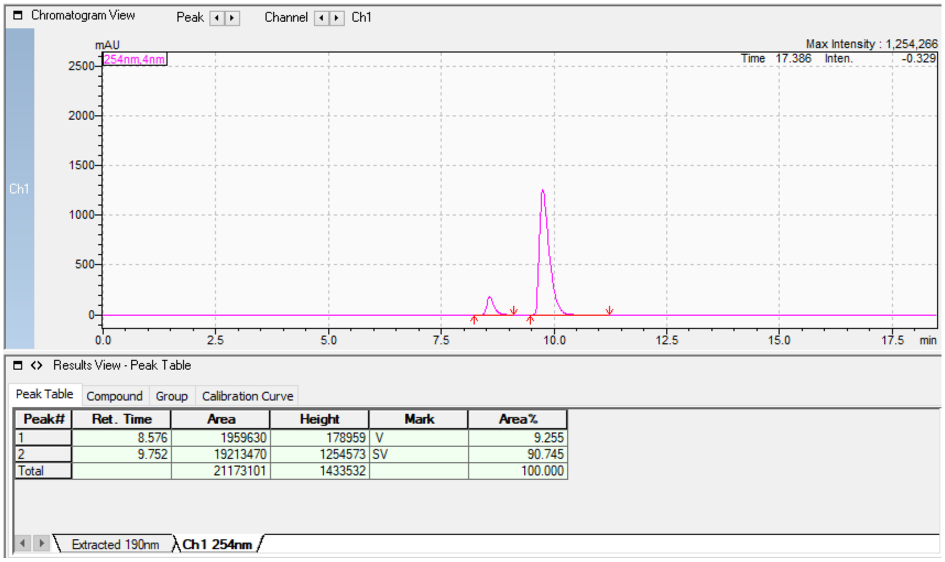

Peak#	Ret. Time	Area	Height	Mark	Area%
1	8.576	1959630	178959	V	9.255
2	9.752	19213470	1254573	SV	90.745
Total		21173101	1433532		100.000

% *ee* = 90.745-9.255 = 81.49 = 81%

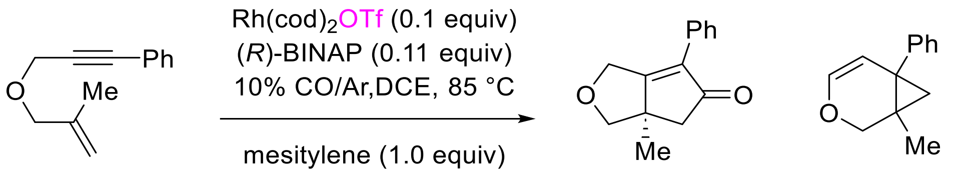

Table 4, entry 10 (Table 1, entry 7 and Table 3, entry 2)

Follows General Procedure **D**: Rh(cod)$_2$OTf (7.1 mg, 0.015 mmol, 0.1 equiv), (*R*)-BINAP (10.3 mg, 0.0165 mmol, 0.11 equiv), mesitylene (18 mg, 0.15 mmol, 1.0 equiv) and enyne **6a** (0.5 mL from a stock solution of 0.3 M) in DCE (3.0 mL, 0.05 M). The reaction flask was lowered into the preheated oil bath (85 °C). After 23 h, the PKR yield (84%), b.r.s.m. (85%), starting material remaining (1%) and cycloisomerized side product (13%) were determined based on the integral comparison to the internal standard mesitylene.

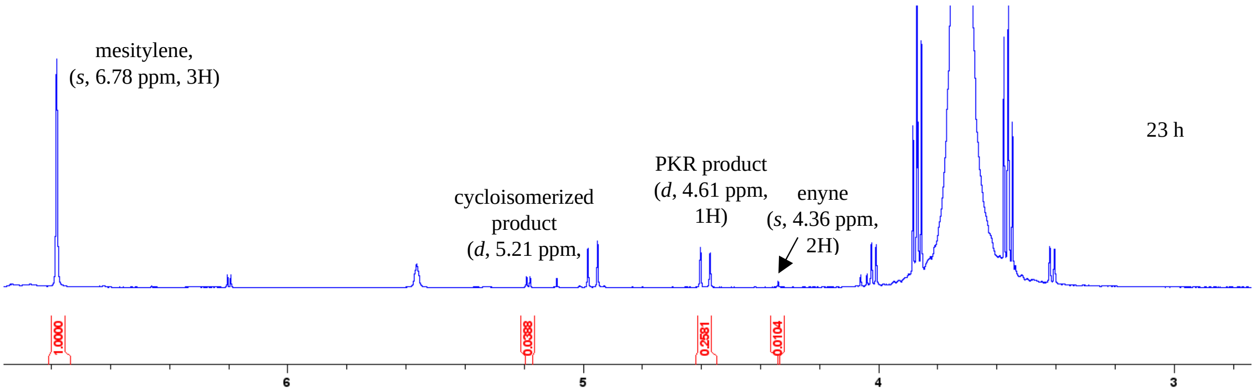

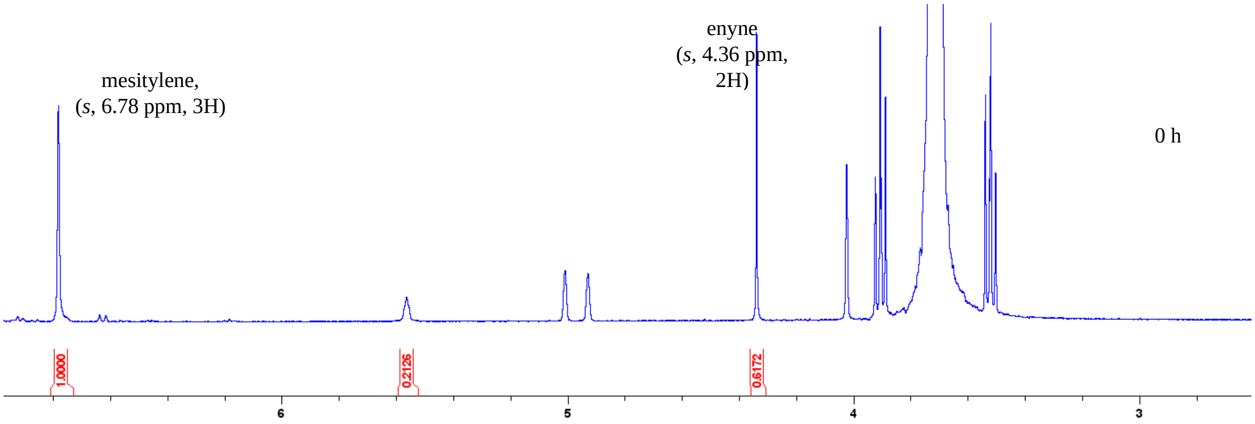

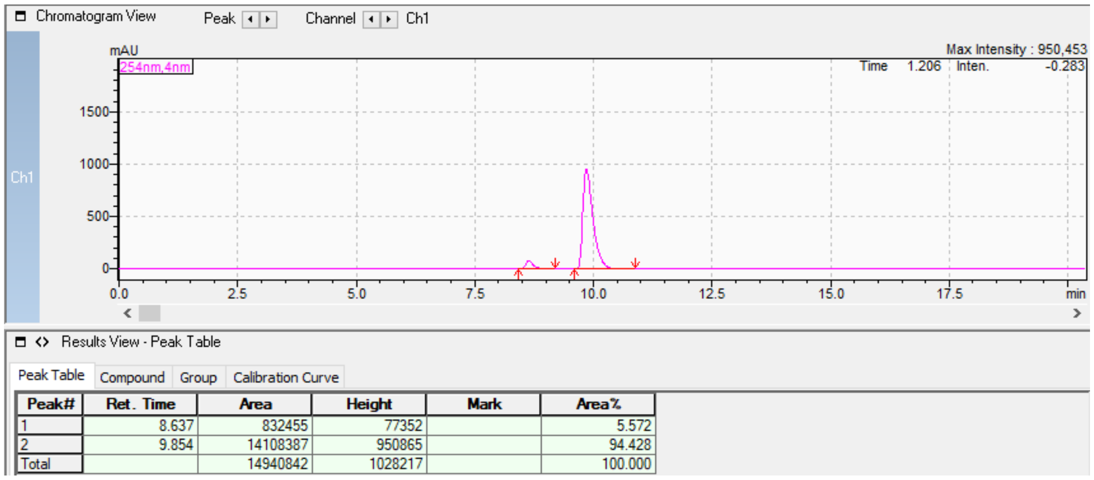

Peak#	Ret. Time	Area	Height	Mark	Area%
1	8.637	832455	77352		5.572
2	9.854	14108387	950865		94.428
Total		14940842	1028217		100.000

%*ee* = 94.428 – 5.572 = 88.856 = 89%

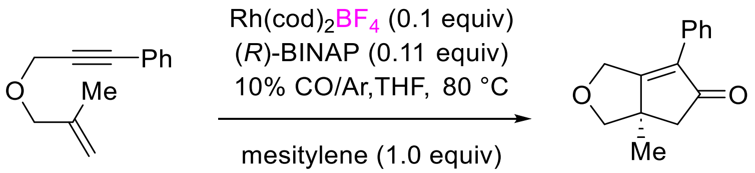

6a **7a**

Table 4, entry 11

Follows general procedure D. Rh(cod)$_2$BF$_4$ (6.1 mg, 0.015 mmol, 0.1 equiv), (*R*)-BINAP (10.3 mg, 0.0165 mmol, 0.11 equiv), mesitylene (18.0 mg, 0.15 mmol, 1.0 equiv) and enyne **6a** (0.5 mL from a stock solution of 0.3 M) in THF (3.0 mL, 0.05 M). The reaction flask was placed in a preheated oil bath (80 °C). After 45 h, PKR yield (8%), b.r.s.m. (8%), starting material remaining (2%) and an unknown aldehyde (34%) were determined by integral comparison to the internal standard mesitylene.

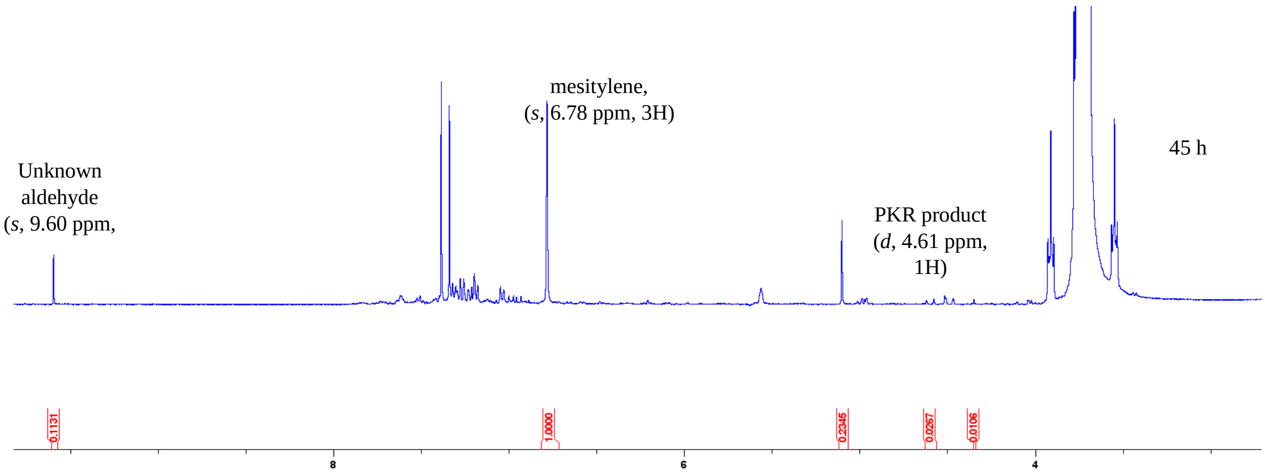

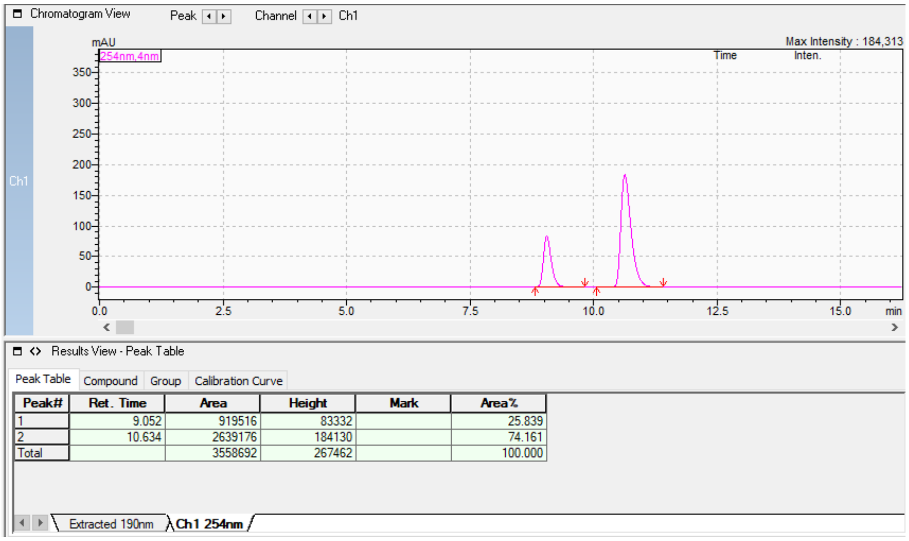

Peak#	Ret. Time	Area	Height	Mark	Area%
1	9.052	919516	83332		25.839
2	10.634	2639176	184130		74.161
Total		3558692	267462		100.000

%*ee* = 74.161-25.839 = 48.322 = 48%

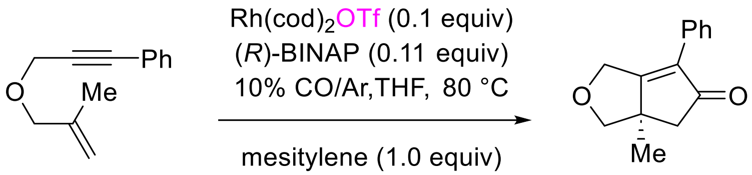

6a **7a**

Table 4, entry 12 and Table 3, entry 1

Follows general procedure D. Rh(cod)$_2$OTf (7.1 mg, 0.015 mmol, 0.1 equiv), (*R*)-BINAP (10.3 mg, 0.0165 mmol, 0.11 equiv), mesitylene (18.0 mg, 0.15 mmol, 1.0 equiv) and enyne **6a** (0.5 mL from a stock solution of 0.3 M) in THF (3.0 mL, 0.05 M). The reaction flask was placed in a preheated oil bath (80 °C). After 24 h, PKR yield (52%), b.r.s.m. (78%), starting material remaining (33%) and cycloisomerized side product (0%) were determined by integral comparison to the internal standard mesitylene.

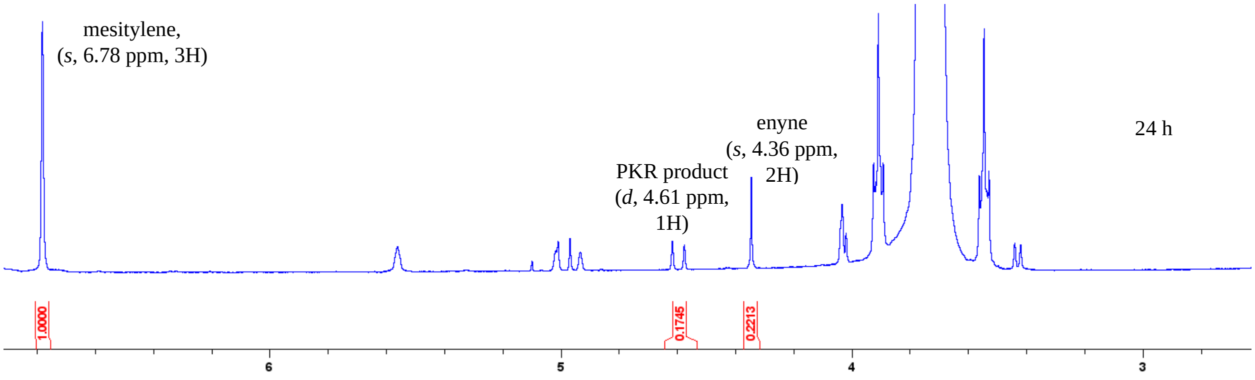

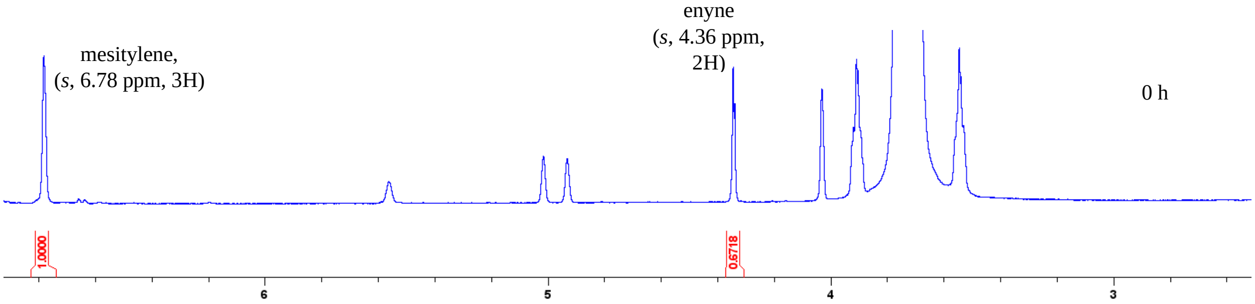

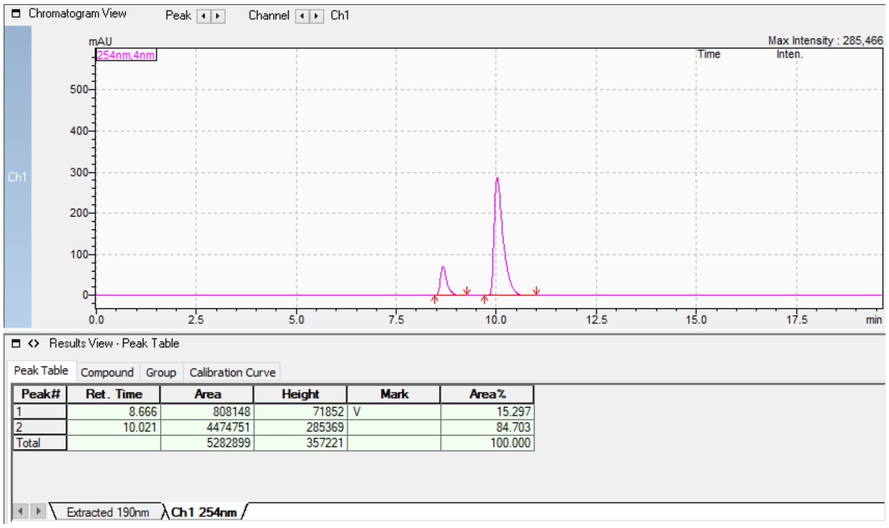

Peak#	Ret. Time	Area	Height	Mark	Area%
1	8.666	808148	71852	V	15.297
2	10.021	4474751	285369		84.703
Total		5282899	357221		100.000

%*ee* = 84.703-15.297 = 69.406 = 69%

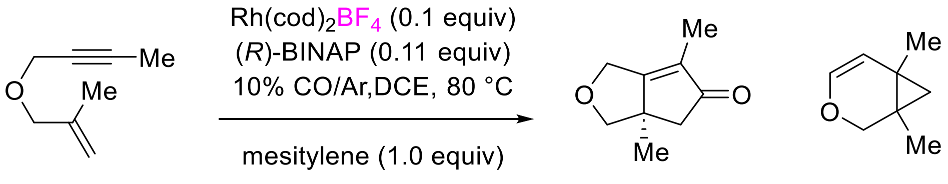

6b **7b** **7bi**

Table 4, **entry 13**

Follows general procedure D. $Rh(cod)_2BF_4$ (6.1 mg, 0.015 mmol, 0.1 equiv), (*R*)-BINAP (10.3 mg, 0.0165 mmol, 0.11 equiv), mesitylene (18.0 mg, 0.15 mmol, 1.0 equiv) and enyne **6b** (0.5 mL from a stock solution of 0.3 M) in DCE (3.0 mL, 0.05 M). The reaction flask was placed in a preheated oil bath (80 °C). After 38 h, PKR yield (35%), b.r.s.m. (35%), starting material remaining (0%) and cycloisomerized side product (55%) were determined by integral comparison to the internal standard mesitylene.

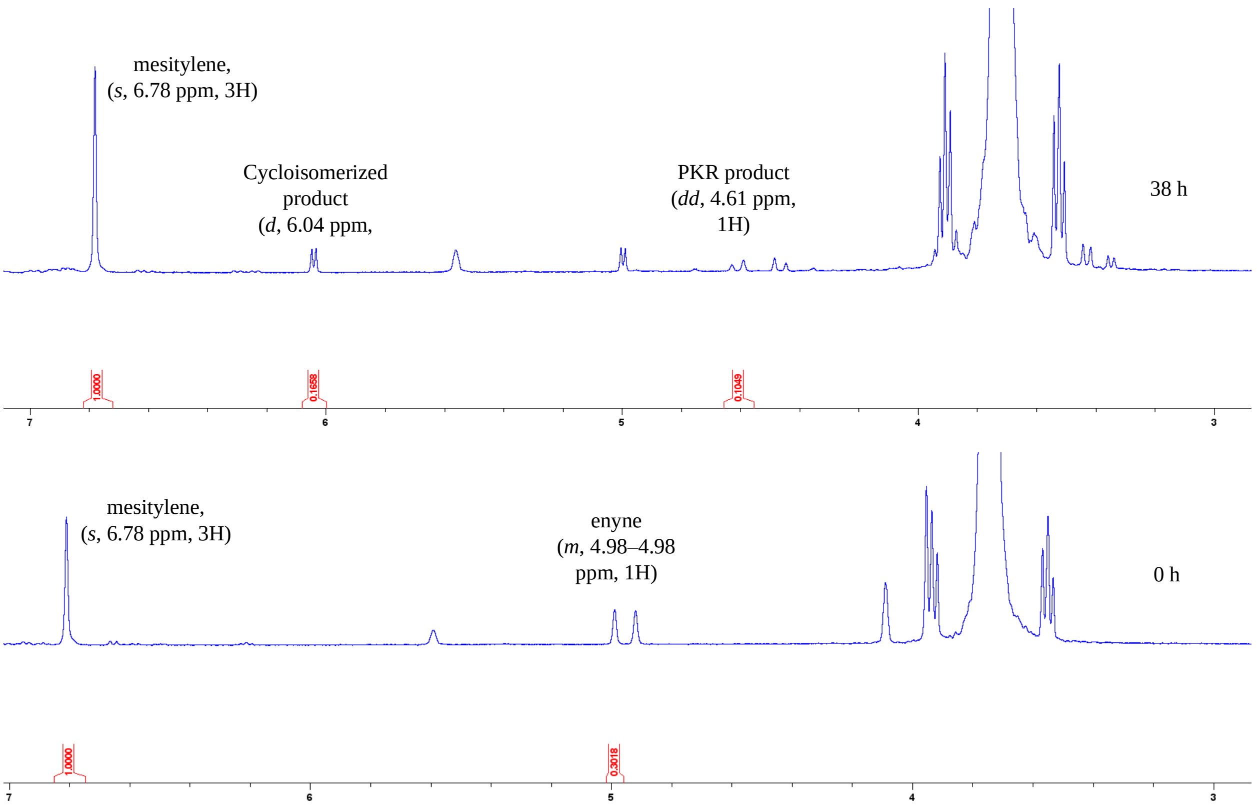

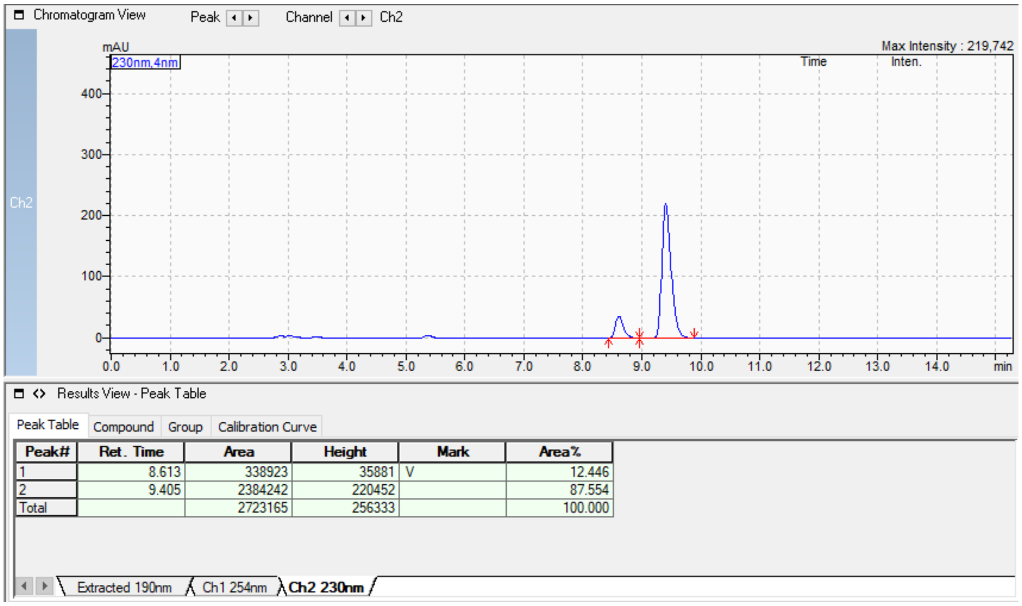

Peak#	Ret. Time	Area	Height	Mark	Area%
1	8.613	338923	35881	V	12.446
2	9.405	2384242	220452		87.554
Total		2723165	256333		100.000

%*ee* = 87.554-12.446 = 75.108 = 75%

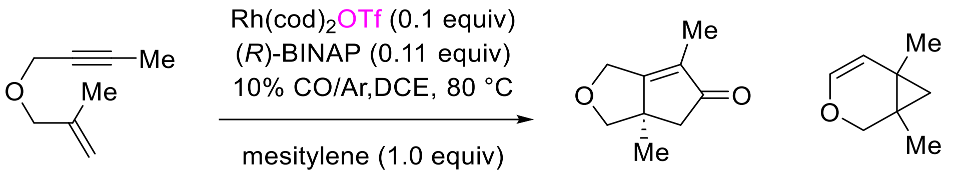

6b **7b** **7bi**

Table 4, entry 14

Follows General Procedure D: Rh(cod)$_2$OTf (7.1 mg, 0.015 mmol, 0.1 equiv), (*R*)-BINAP (10.3 mg, 0.0165 mmol, 0.11 equiv), mesitylene (18 mg, 0.15 mmol, 1.0 equiv) and enyne **6b** (0.5 mL from a stock solution of 0.3 M) in DCE (3.0 mL, 0.05 M). The reaction flask was lowered into the preheated oil bath (80 °C). After 27 h, the PKR yield (66%), b.r.s.m. (66%), starting material remaining (trace) and cycloisomerized side product (25%) were determined based on the integral comparison to the internal standard mesitylene peak (s, 6.78 ppm, 3 H) via ^{1}H NMR.

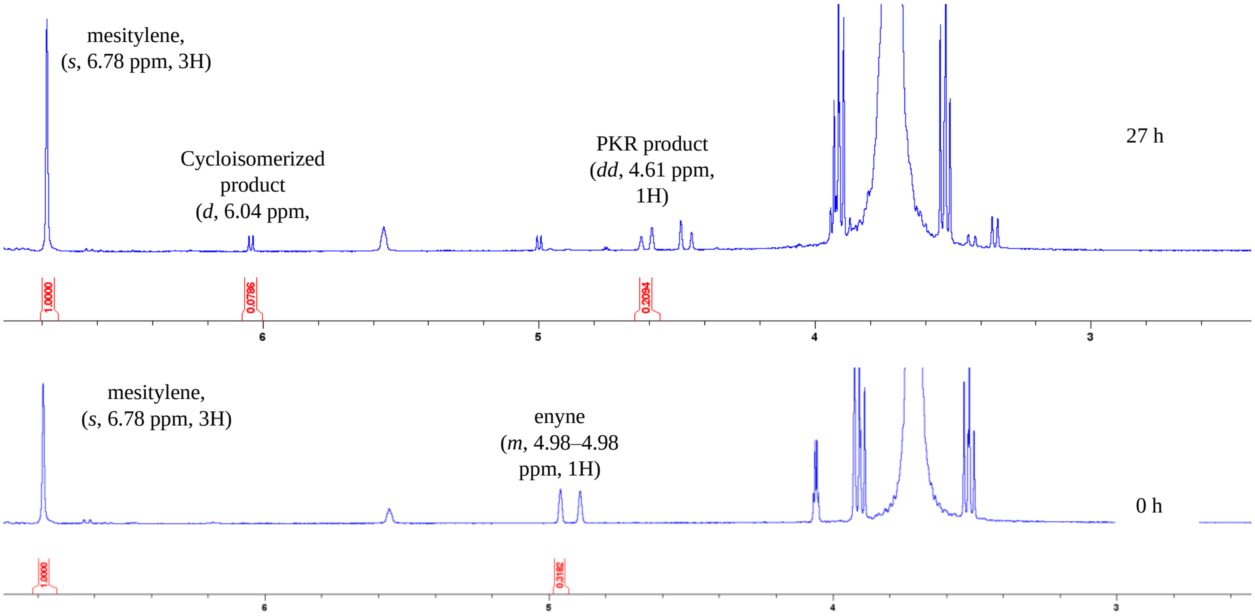

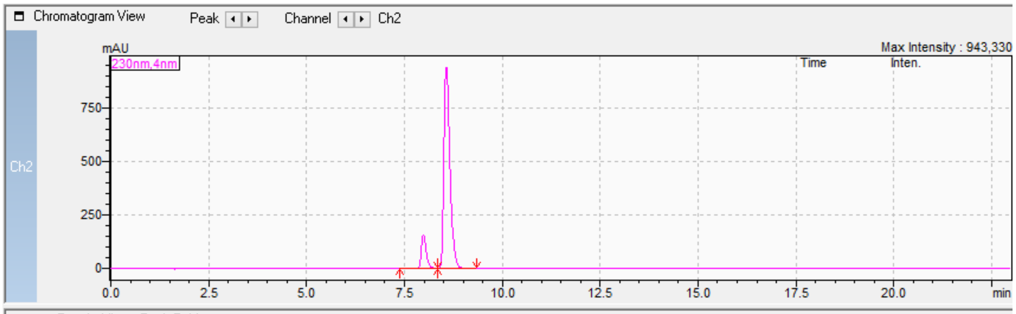

Results View - Peak Table

Peak Table | Compound | Group | Calibration Curve

Peak#	Ret. Time	Area	Height	Mark	Area%
1	7.985	1464991	157551	V	12.565
2	8.572	10194013	945190	V	87.435
Total		11659004	1102740		100.000

%*ee* = 87.435 – 12.565 = 74.87 = 75%

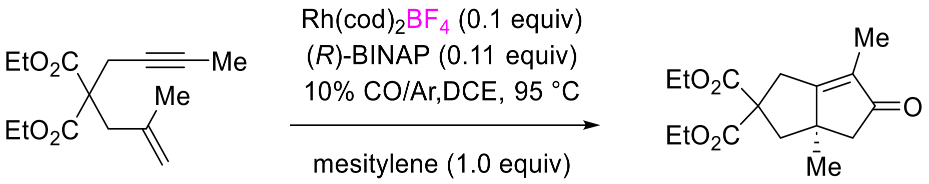

8b **9b**

Table 4, **entry 15**

Follows General Procedure **D** for asymmetric PKR: Rh(cod)$_2$BF$_4$ (6.1 mg, 0.015 mmol, 0.1 equiv), (*R*)-BINAP (10.3 mg, 0.0165 mmol, 0.11 equiv), mesitylene (18 mg, 0.15 mmol, 1.0 equiv) and enyne **8b** (0.5 mL from a stock solution of 0.3 M) in DCE (3.0 mL, 0.05 M). The reaction flask was lowered into the preheated oil bath (95 °C). After 15 h, the PKR yield (93%), b.r.s.m. (93%) and starting material remaining (trace) were determined based on the integral comparison to the internal standard mesitylene.

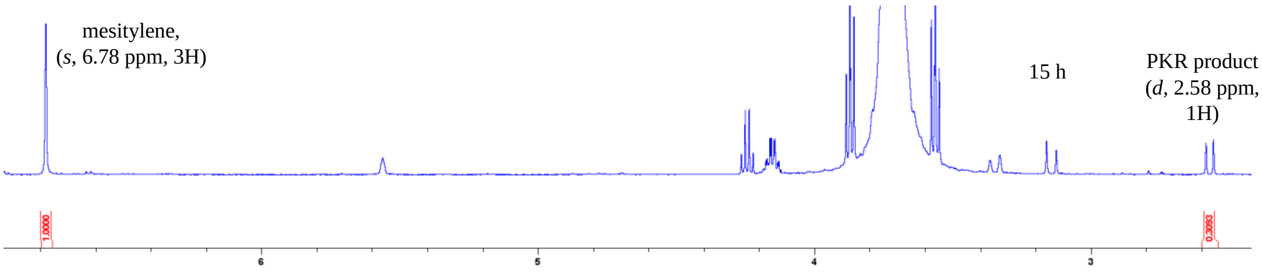

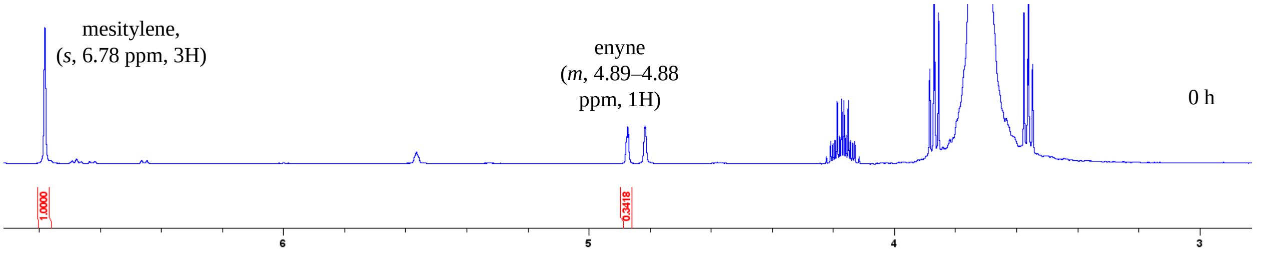

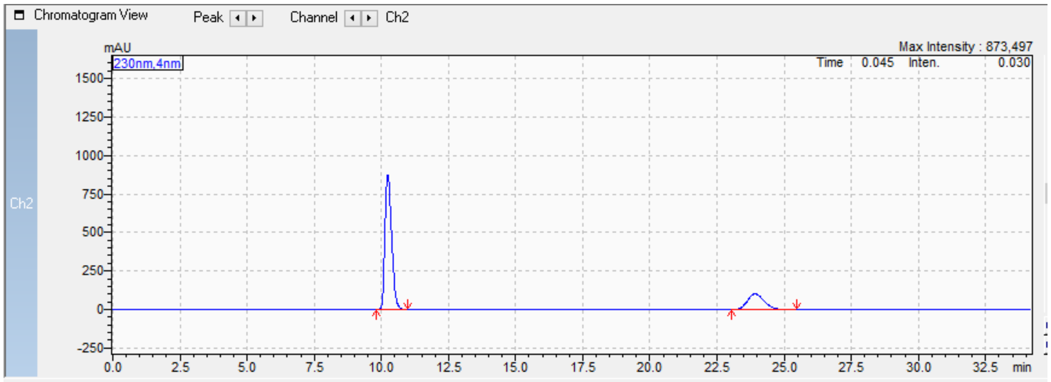

Results View - Peak Table

Peak Table | Compound | Group | Calibration Curve

Peak#	Ret. Time	Area	Height	Mark	Area%
1	10.238	15144599	873740		77.671
2	23.909	4353768	100947	V	22.329
Total		19498367	974687		100.000

%*ee* = 77.671 – 22.329 = 55.342 = 55%

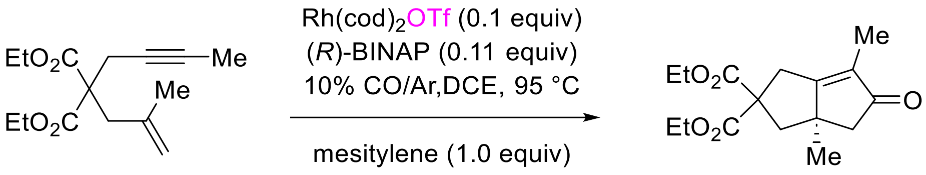

8b **9b**

Table 4, **entry 16**

Follows general procedure D. $Rh(cod)_2OTf$ (7.1 mg, 0.015 mmol, 0.1 equiv), (*R*)-BINAP (10.3 mg, 0.0165 mmol, 0.11 equiv), mesitylene (18 mg, 0.15 mmol, 1.0 equiv) and enyne **8b** (0.5 mL from a stock solution of 0.3 M) in DCE (3.0 mL, 0.05 M). The reaction flask was lowered into the preheated oil bath (95 °C). After 11 h, PKR yield (67%), b.r.s.m. (67%) and starting material remaining (trace) were determined by integral comparison to the internal standard mesitylene.

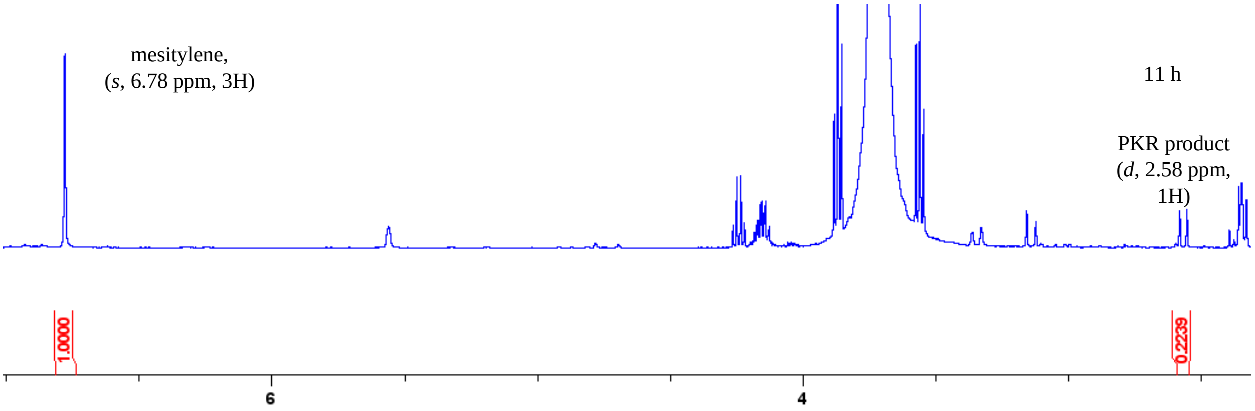

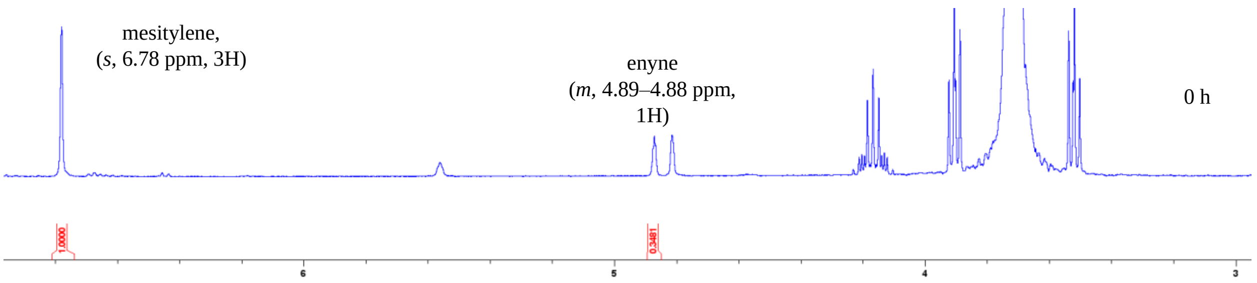

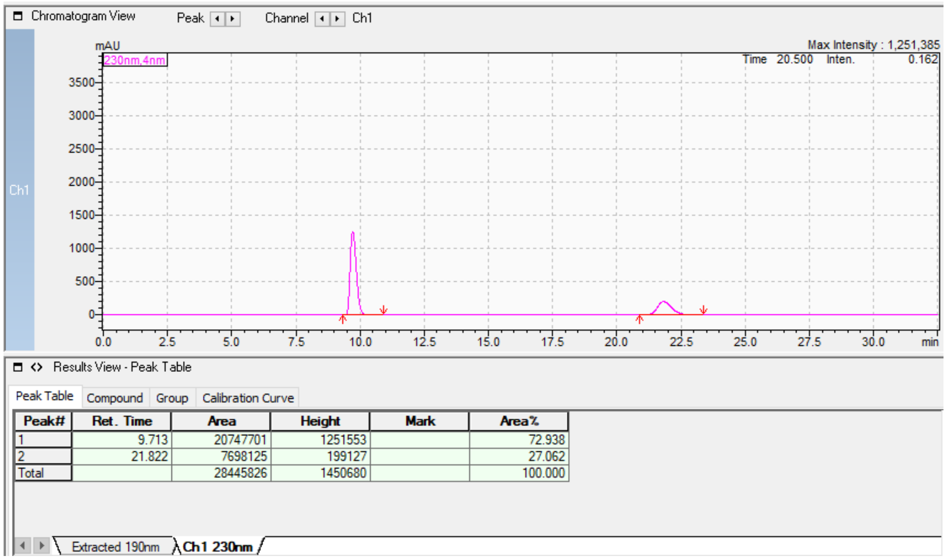

% *ee* = 72.938-27.062 = 45.876 = 46%

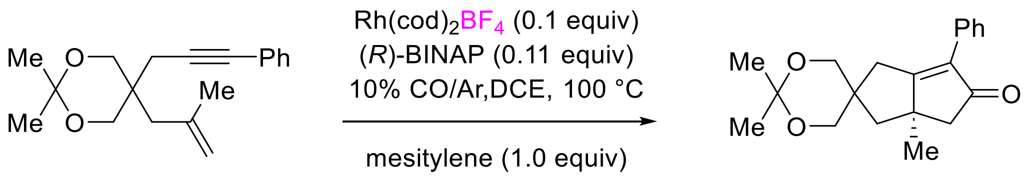

10a **11a**

Table 4, **entry 17**

Follows General Procedure **D** for asymmetric PKR: Rh(cod)$_2$BF$_4$ (6.1 mg, 0.015 mmol, 0.1 equiv), (*R*)-BINAP (10.3 mg, 0.0165 mmol, 0.11 equiv), mesitylene (18 mg, 0.15 mmol, 1.0 equiv) and enyne **10a** (0.5 mL from a stock solution of 0.3 M) in DCE (3.0 mL, 0.05 M). The reaction flask was lowered into the preheated oil bath (100 °C). After 88 h, the PKR yield (81%), b.r.s.m. (81%) and starting material remaining (trace) were determined based on the integral comparison to the internal standard mesitylene.

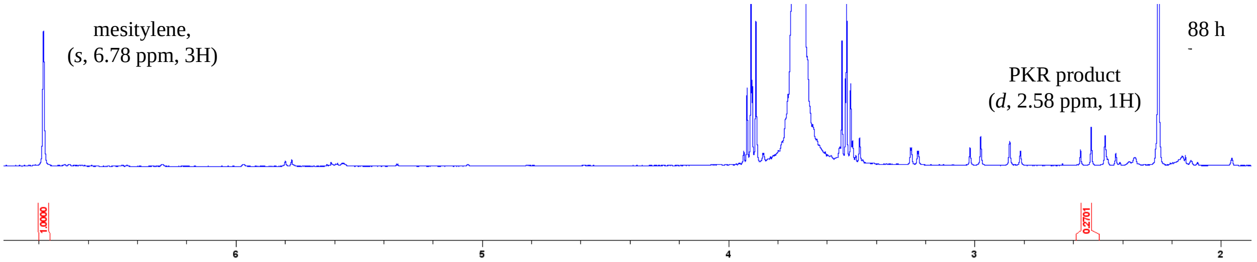

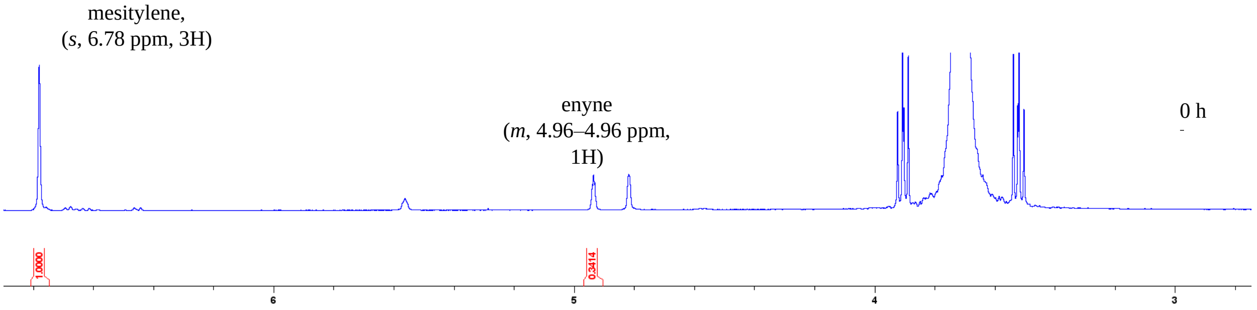

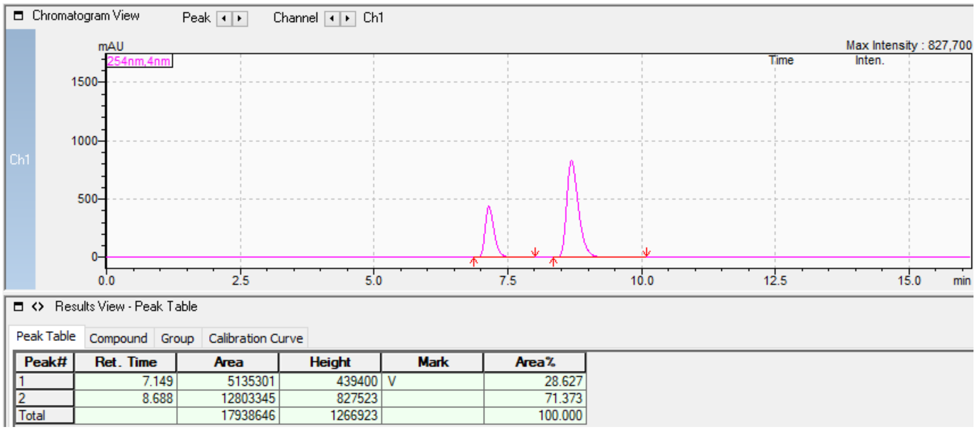

Peak#	Ret. Time	Area	Height	Mark	Area%
1	7.149	5135301	439400	V	28.627
2	8.688	12803345	827523		71.373
Total		17938646	1266923		100.000

%*ee* = 71.373 – 28.627 = 42.746 = 43%

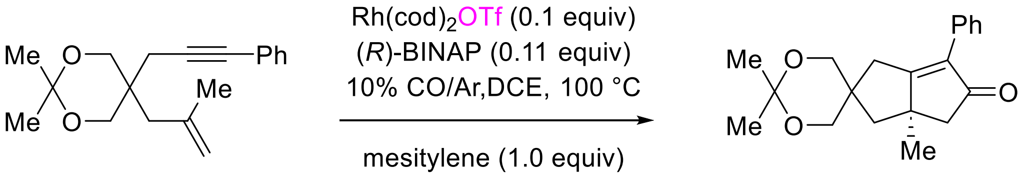

10a **11a**

Table 4, entry 18

Follows general procedure D. Rh(cod)$_2$OTf (7.1 mg, 0.015 mmol, 0.1 equiv), (*R*)-BINAP (10.3 mg, 0.0165 mmol, 0.11 equiv), mesitylene (18 mg, 0.15 mmol, 1.0 equiv) and enyne **10a** (0.5 mL from a stock solution of 0.3 M) in DCE (3.0 mL, 0.05 M). The reaction flask was lowered into the preheated oil bath (100 °C). After 27 h, PKR yield (34%), b.r.s.m. (35%) and starting material remaining (2%) were determined based on the integral comparison to the internal standard mesitylene.

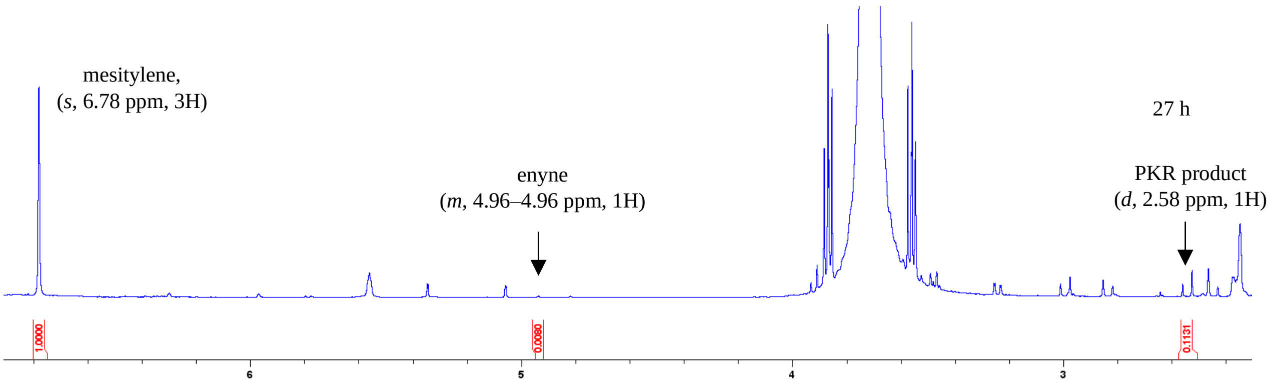

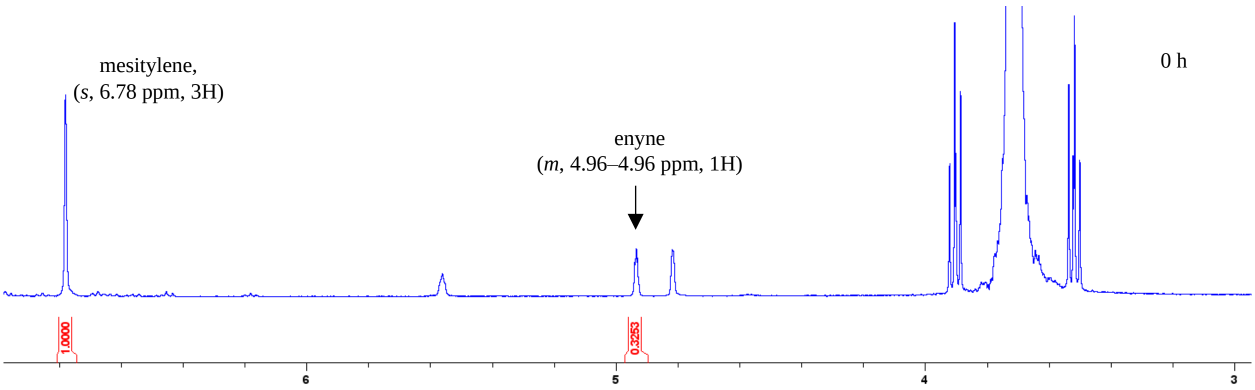

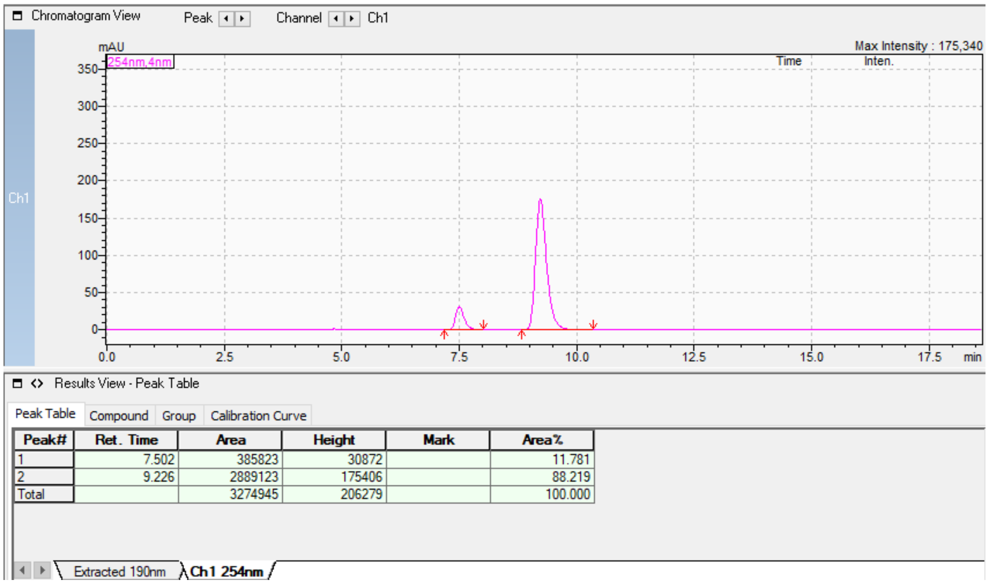

Peak#	Ret. Time	Area	Height	Mark	Area%
1	7.502	385823	30872		11.781
2	9.226	2889123	175406		88.219
Total		3274945	206279		100.000

% *ee* = 88.219-11.781 = 76.438 = 76%

X-Ray Crystallography Information for (*R*)-5ax and 5ai

1. Assignment of the absolute configuration of PKR product 5a

Conversion of PKR product **5a** (*er*: 93:7, as determined by HPLC) to (*R*,*E*)-4-methyl-*N*'-(3a-methyl-6-phenyl-2-tosyl-2,2,3a,4-tetrahydrocyclopenta[*c*]pyrrol-5(1*H*)-ylidene)benzenesulfonohydrazide (*R*)-**5ax.**

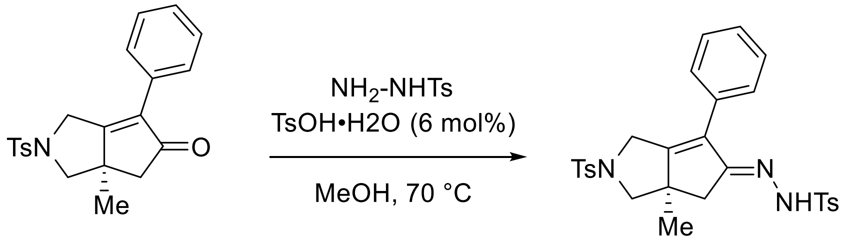

5a *er*: 93:7 **(*R*)-5ax**

A 10-mL round bottomed flask was charged with enyne **5a** (30 mg, 0.08 mmol, 1.0 equiv), *p*-toluenesulfonyl hydrazide (18.4 mg, 0.10 mmol, 1.2 equiv) and *p*-toluenesulfonic acid monohydrate (1 mg, 0.005 mmol, 0.06 equiv). The flask was equipped with a reflux condenser followed by the addition of MeOH (2 mL). The reaction mixture was heated at 70 ℃ in an oil bath for 18 h until no starting material remained as evidenced by TLC. The cloudy mixture was concentrated on rotavap and re-dissolved in DCM. Silica gel (1.0 g) was added, and the mixture was concentrated on rotavap. The residue was loaded onto a SiO_2 column eluting with 40% EtOAc/Hexanes to get the title compound (*R*)-**5ax** as a light-yellow solid (30 mg, 0.06 mmol, 70% yield).

^{1}H NMR ($CDCl_3$, 400 MHz)

δ 7.86 (d, *J* = 7.9 Hz, 2 H), 7.78 (s, br, 1 H), 7.64 (d, *J* = 7.9 Hz, 2 H), 7.33–7.22 (m, 8 H), 4.35 (d, *J* = 15.6 Hz, 1 H), 3.89 (d, *J* = 15.6 Hz, 1 H), 3.55 (d, *J* = 9.0 Hz, 1 H), 2.72 (d, *J* = 9.0 Hz, 1 H), 2.56 (d, *J* = 17.1 Hz, 1 H), 2.44 (m, 1 H) 2.43 (s, 3 H), 2.38 (s, 3 H), 1.09 (s, 3 H)

small impurities at 7.72–7.70, 7.56–7.54, 7.18–7.08, 4.11 (ethyl acetate), 3.38–3.35, 3.23–3.21, 3.01–2.99, 2.65–2.64, 2.27–2.17, 2.04 (ethyl acetate), 1.26 (ethyl acetate)

HRMS

HRMS-ESI (m/z): $[M + H]^+$ calcd for $C_{28}H_{30}N_3O_4S_2$, 536.1672; found: 536.1657

An X-ray quality crystal was grown through vapor diffusion technique using deuterated chloroform and methanol. The product **5ax** was dissolved in a minimum amount of $CDCl_3$ in a 1-mL vial. The 1-mL vial was then placed in a 20-mL scintillation vial that contained methanol (3 mL). The 20-mL scintillation vial was tightly sealed with a cap and placed in a freezer at 0 °C for crystal growth. Crystals of **5ax** were formed after 3 d. A specimen of $C_{28}H_{29}N_3O_4S_2$ was used for X-ray crystallographic analysis. The X-ray intensity data were measured on a microfocus Bruker Venture Duo Mo ImuS system equipped with a Bruker Photon III CPAD detector (MoK_{α}, λ = 0.71073 Å).The integration of the data using a monoclinic unit cell yielded a total of 36338 reflections to a maximum θ angle of 28.29° (0.75 Å resolution), of which 7072 were independent (average redundancy 5.138, completeness = 99.9%, R_{int} = 4.13%, R_{sig} = 3.40%) and 6433 (90.96%) were greater than $2\sigma(F^2)$. The final cell constants of a = 8.3097(3) Å, b = 5.9914(2) Å, c = 28.9828(11) Å, β = 98.1450(10)°, volume = 1428.40(9) $Å^3$, are based upon the refinement of the XYZ-centroids of 122 reflections above 20 σ(I) with 5.000° < 2θ < 45.64°.The final anisotropic full-matrix least-squares refinement on F^2 with 338 variables converged at R1 = 8.48%, for the observed data and wR2 = 24.65% for all data. The goodness-of-fit was 1.133. The largest peak in the final difference electron density synthesis was 2.327 $e^-/Å^3$ and the largest hole was -0.754 $e^-/Å^3$ with an RMS deviation of 0.121 $e^-/Å^3$. On the basis of the final model, the calculated density was 1.318 g/cm^3 and F(000), 598 e^-.

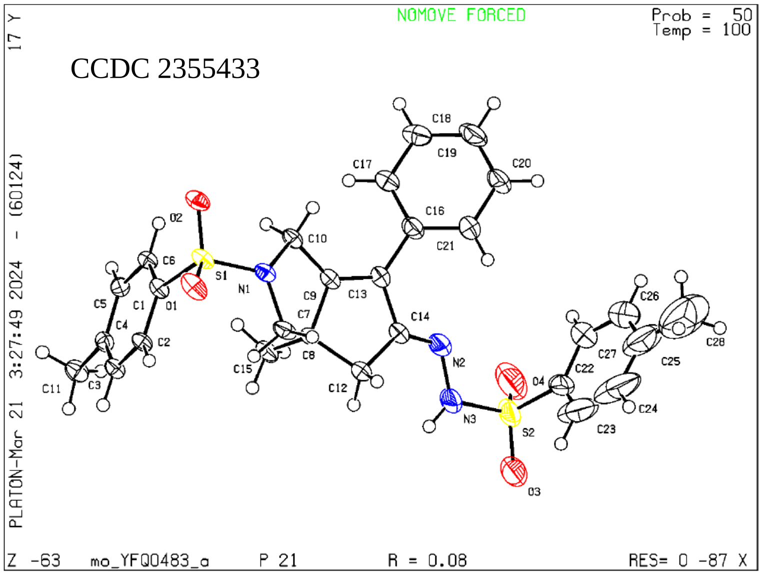

Table S11. Sample and crystal data for YFQ0483

**Identification code**	YFQ0483	
**Chemical formula**	$C_{28}H_{29}N_3O_4S_2$	
**Formula weight**	535.66 g/mol	
**Temperature**	100(2) K	
**Wavelength**	0.71073 Å	
**Crystal system**	monoclinic	
**Space group**	P 1 21 1	
**Unit cell dimensions**	a = 8.3097(3) Å	α = 90°
	b = 5.9914(2) Å	β = 98.1450(10)°
	c = 28.9828(11) Å	γ = 90°
**Volume**	1428.40(9) $Å^3$	
**Z**	2	
**Density (calculated)**	1.245 g/cm^3	
**Absorption coefficient**	0.223 mm^{-1}	
**F(000)**	598	

Table S12. Data collection and structure refinement for YFQ0483

**Diffractometer**	microfocus Bruker Venture Duo Mo ImuS	
**Radiation source**	Bruker Photon III CPAD detector (MoK_{α}, λ = 0.71073 Å)	
**Theta range for data collection**	2.13 to 28.29°	
**Index ranges**	-11<=h<=11, -7<=k<=7, -38<=l<=38	
**Reflections collected**	36338	
**Independent reflections**	7072 [R(int) = 0.0413]	
**Coverage of independent reflections**	99.9%	
**Absorption correction**	multi-scan	
**Refinement method**	Full-matrix least-squares on F^2	
**Refinement program**	SHELXL-2019/1 (Sheldrick, 2019)	
**Function minimized**	$\Sigma w(F_o^2 - F_c^2)^2$	
**Data / restraints / parameters**	7072 / 1 / 338	
**Goodness-of-fit on F^2**	1.133	
**Final R indices**	6433 data; I>2σ(I)	R1 = 0.0848, wR2 = 0.2374
	all data	R1 = 0.0915, wR2 = 0.2465

**Weighting scheme**	w=1/[$\sigma^2(F_o^2)+(0.1507P)^2+1.4128P$] where $P=(F_o^2+2F_c^2)/3$
**Absolute structure parameter**	0.03(16)
**Largest diff. peak and hole**	2.327 and -0.754 eÅ^{-3}
**R.M.S. deviation from mean**	0.121 eÅ^{-3}

Table S13. Atomic coordinates and equivalent isotropic atomic displacement parameters (Å^2) for YFQ0483.

U(eq) is defined as one third of the trace of the orthogonalized U_{ij} tensor.

	x/a	y/b	z/c	U(eq)
S1	0.70181(13)	0.03063(18)	0.09782(4)	0.0245(3)
O1	0.6691(5)	0.2623(6)	0.10449(16)	0.0346(9)
N1	0.7561(5)	0.9248(7)	0.14954(15)	0.0238(8)
C1	0.5177(6)	0.9033(7)	0.07455(17)	0.0212(9)
S2	0.6890(3)	0.2415(11)	0.38254(9)	0.128(2)
O2	0.8248(4)	0.9654(6)	0.07055(15)	0.0300(8)
N2	0.7636(8)	0.3665(17)	0.3045(2)	0.069(2)
C2	0.3720(5)	0.9965(8)	0.08349(17)	0.0226(9)
O3	0.5634(9)	0.306(3)	0.4094(3)	0.184(8)
N3	0.6489(10)	0.395(3)	0.3350(3)	0.115(5)
C3	0.2275(6)	0.8790(8)	0.07157(18)	0.0249(9)
O4	0.7180(9)	0.005(2)	0.3690(3)	0.116(4)
C4	0.2273(6)	0.6683(8)	0.05084(16)	0.0225(9)
C5	0.3745(6)	0.5820(7)	0.04054(16)	0.0225(9)
C6	0.5194(6)	0.6964(8)	0.05251(16)	0.0224(9)
C7	0.6546(6)	0.9616(9)	0.1872(2)	0.0287(11)
C8	0.6310(6)	0.7279(9)	0.20603(17)	0.0265(10)
C9	0.7885(6)	0.6202(9)	0.19818(18)	0.0257(10)
C10	0.8421(6)	0.7055(8)	0.15464(18)	0.0242(9)
C11	0.0713(6)	0.5355(10)	0.03964(19)	0.0300(10)
C12	0.6293(7)	0.6971(13)	0.2587(2)	0.0399(14)
C13	0.8565(6)	0.4941(10)	0.23424(19)	0.0323(12)
C14	0.7516(7)	0.5096(15)	0.2705(2)	0.0446(16)
C15	0.4859(6)	0.6080(9)	0.1781(2)	0.0304(11)
C16	0.0101(7)	0.3662(11)	0.2386(2)	0.0341(12)
C17	0.0587(6)	0.2643(9)	0.1995(2)	0.0310(11)

	x/a	y/b	z/c	U(eq)
C18	0.2019(7)	0.1444(11)	0.2031(2)	0.0394(13)
C19	0.3021(8)	0.1326(15)	0.2456(3)	0.055(2)
C20	0.2565(10)	0.237(2)	0.2846(3)	0.072(3)
C21	0.1107(9)	0.3521(17)	0.2816(2)	0.058(2)
C22	0.8805(11)	0.352(3)	0.4127(3)	0.087(4)
C23	0.892(2)	0.552(3)	0.4375(5)	0.124(7)
C24	0.036(2)	0.614(3)	0.4596(5)	0.107(5)
C25	0.1759(17)	0.498(3)	0.4588(4)	0.097(4)
C26	0.1633(11)	0.304(2)	0.4328(3)	0.071(3)
C27	0.0187(10)	0.224(2)	0.4091(3)	0.067(3)
C28	0.330(2)	0.575(5)	0.4820(6)	0.159(10)

Table S14. Bond lengths (Å) for YFQ0483.

S1-O2	1.432(4)	S1-O1	1.432(4)
S1-N1	1.631(4)	S1-C1	1.756(5)
N1-C7	1.487(7)	N1-C10	1.493(6)
C1-C2	1.391(6)	C1-C6	1.396(6)
S2-O3	1.442(9)	S2-O4	1.498(15)
S2-N3	1.650(8)	S2-C22	1.828(13)
N2-C14	1.301(10)	N2-N3	1.399(9)
C2-C3	1.392(7)	C3-C4	1.398(7)
C4-C5	1.399(7)	C4-C11	1.516(7)
C5-C6	1.386(7)	C7-C8	1.526(8)
C8-C9	1.505(6)	C8-C15	1.533(8)
C8-C12	1.539(7)	C9-C13	1.347(7)
C9-C10	1.487(7)	C12-C14	1.521(9)
C13-C14	1.459(8)	C13-C16	1.479(7)
C16-C17	1.396(8)	C16-C21	1.402(9)
C17-C18	1.381(8)	C18-C19	1.387(11)
C19-C20	1.392(13)	C20-C21	1.384(10)
C22-C23	1.39(3)	C22-C27	1.399(12)
C23-C24	1.33(2)	C24-C25	1.36(2)
C25-C26	1.384(18)	C25-C28	1.43(2)
C26-C27	1.382(14)		

Table S15. Bond angles (°) for YFQ0483.

O2-S1-O1	120.2(2)	O2-S1-N1	105.9(2)

O1-S1-N1	106.6(2)	O2-S1-C1	108.7(2)
O1-S1-C1	107.5(2)	N1-S1-C1	107.3(2)
C7-N1-C10	111.8(4)	C7-N1-S1	120.3(3)
C10-N1-S1	120.0(3)	C2-C1-C6	120.5(4)
C2-C1-S1	119.2(4)	C6-C1-S1	119.8(4)
O3-S2-O4	123.5(9)	O3-S2-N3	103.0(5)
O4-S2-N3	109.2(6)	O3-S2-C22	106.9(7)
O4-S2-C22	107.9(5)	N3-S2-C22	104.8(6)
C14-N2-N3	114.4(7)	C1-C2-C3	119.6(4)
N2-N3-S2	112.6(6)	C2-C3-C4	120.8(4)
C3-C4-C5	118.5(4)	C3-C4-C11	121.0(4)
C5-C4-C11	120.5(4)	C6-C5-C4	121.2(4)
C5-C6-C1	119.3(4)	N1-C7-C8	104.0(4)
C9-C8-C7	100.4(4)	C9-C8-C15	110.8(4)
C7-C8-C15	111.9(4)	C9-C8-C12	103.2(4)
C7-C8-C12	119.0(5)	C15-C8-C12	110.6(5)
C13-C9-C10	135.1(5)	C13-C9-C8	113.7(5)
C10-C9-C8	110.6(4)	C9-C10-N1	101.0(4)
C14-C12-C8	102.2(4)	C9-C13-C14	107.2(5)
C9-C13-C16	128.2(5)	C14-C13-C16	124.6(5)
N2-C14-C13	121.0(6)	N2-C14-C12	129.4(6)
C13-C14-C12	109.5(5)	C17-C16-C21	119.4(5)
C17-C16-C13	120.2(5)	C21-C16-C13	120.3(6)
C18-C17-C16	120.7(6)	C17-C18-C19	119.8(6)
C18-C19-C20	120.0(6)	C21-C20-C19	120.6(7)
C20-C21-C16	119.5(7)	C23-C22-C27	121.1(12)
C23-C22-S2	123.3(9)	C27-C22-S2	115.6(11)
C24-C23-C22	118.6(10)	C23-C24-C25	124.4(14)
C24-C25-C26	116.1(13)	C24-C25-C28	122.6(17)
C26-C25-C28	121.3(15)	C27-C26-C25	123.8(10)
C26-C27-C22	115.9(11)		

Table S16. Anisotropic atomic displacement parameters (Å^2) for YFQ0483.

The anisotropic atomic displacement factor exponent takes the form: $-2\pi^2[h^2 a^{*2} U_{11} + ... + 2 h k a^* b^* U_{12}]$

	U_{11}	U_{22}	U_{33}	U_{23}	U_{13}	U_{12}
S1	0.0167(5)	0.0178(5)	0.0384(6)	0.0025(5)	0.0020(4)	0.0007(4)
O1	0.0263(18)	0.0184(18)	0.057(3)	0.0037(16)	-0.0023(17)	-0.0011(14)

	U_{11}	U_{22}	U_{33}	U_{23}	U_{13}	U_{12}
N1	0.0182(18)	0.0204(19)	0.032(2)	-0.0030(16)	0.0023(15)	0.0034(14)
C1	0.019(2)	0.014(2)	0.029(2)	0.0019(16)	0.0000(16)	0.0001(15)
S2	0.0381(11)	0.297(6)	0.0536(13)	0.088(2)	0.0185(9)	0.054(2)
O2	0.0182(16)	0.0301(19)	0.043(2)	0.0072(15)	0.0070(14)	0.0001(13)
N2	0.039(3)	0.128(7)	0.043(3)	0.035(4)	0.013(2)	0.040(4)
C2	0.0187(19)	0.017(2)	0.031(2)	0.0010(17)	0.0026(16)	0.0037(16)
O3	0.055(4)	0.44(2)	0.069(5)	0.123(9)	0.033(4)	0.088(8)
N3	0.051(4)	0.253(15)	0.045(4)	0.067(6)	0.019(3)	0.073(6)
C3	0.020(2)	0.023(2)	0.031(2)	-0.0019(18)	0.0004(17)	0.0036(17)
O4	0.043(3)	0.207(12)	0.099(6)	0.082(7)	0.010(4)	0.016(6)
C4	0.020(2)	0.023(2)	0.025(2)	0.0049(17)	0.0012(16)	-0.0001(17)
C5	0.024(2)	0.021(2)	0.023(2)	-0.0024(15)	0.0019(16)	0.0012(15)
C6	0.019(2)	0.022(2)	0.027(2)	0.0012(18)	0.0032(16)	0.0033(16)
C7	0.019(2)	0.029(2)	0.038(3)	-0.0084(19)	0.0029(19)	0.0056(17)
C8	0.020(2)	0.034(3)	0.026(2)	0.0030(19)	0.0046(17)	0.0085(19)
C9	0.019(2)	0.026(2)	0.032(2)	-0.0008(19)	0.0000(17)	0.0033(18)
C10	0.020(2)	0.020(2)	0.032(2)	-0.0007(17)	0.0007(17)	0.0058(16)
C11	0.023(2)	0.027(2)	0.039(3)	-0.004(2)	0.0014(18)	-0.003(2)
C12	0.030(3)	0.060(4)	0.030(3)	0.003(3)	0.004(2)	0.022(3)
C13	0.025(2)	0.042(3)	0.029(2)	0.004(2)	0.0006(18)	0.010(2)
C14	0.028(3)	0.073(5)	0.032(3)	0.008(3)	0.003(2)	0.022(3)
C15	0.022(2)	0.028(2)	0.041(3)	0.008(2)	0.003(2)	0.0061(18)
C16	0.026(2)	0.038(3)	0.038(3)	0.008(2)	0.003(2)	0.008(2)
C17	0.025(2)	0.027(2)	0.042(3)	0.001(2)	0.005(2)	0.003(2)
C18	0.034(3)	0.034(3)	0.054(4)	0.008(3)	0.018(3)	0.007(2)
C19	0.033(3)	0.072(5)	0.064(4)	0.032(4)	0.016(3)	0.026(3)
C20	0.042(4)	0.125(9)	0.051(4)	0.036(5)	0.010(3)	0.045(5)
C21	0.042(4)	0.095(6)	0.035(3)	0.011(4)	0.004(3)	0.034(4)
C22	0.047(4)	0.173(12)	0.046(4)	0.050(6)	0.026(4)	0.054(6)
C23	0.144(13)	0.166(15)	0.079(8)	0.062(9)	0.076(9)	0.111(13)
C24	0.163(14)	0.094(9)	0.081(8)	0.004(7)	0.080(10)	0.035(10)
C25	0.108(9)	0.133(12)	0.056(5)	-0.030(7)	0.037(6)	-0.003(9)
C26	0.049(4)	0.105(8)	0.060(5)	-0.012(5)	0.011(4)	0.012(5)
C27	0.043(4)	0.107(8)	0.049(4)	0.002(4)	0.004(3)	0.025(5)

	U_{11}	U_{22}	U_{33}	U_{23}	U_{13}	U_{12}
C28	0.141(14)	0.25(3)	0.096(10)	-0.080(14)	0.033(10)	-0.061(16)

Table S17. Hydrogen atomic coordinates and isotropic atomic displacement parameters (Å^2) for YFQ0483.

	**x/a**	**y/b**	**z/c**	**U(eq)**
H2	0.3710	1.1394	0.0976	0.027000
H3A	0.5646	0.4849	0.3295	0.138000
H3	0.1279	0.9428	0.0776	0.030000
H5	0.3752	0.4423	0.0250	0.027000
H6	0.6188	0.6346	0.0458	0.027000
H7A	0.5489	1.0298	0.1746	0.034000
H7B	0.7110	1.0596	0.2118	0.034000
H10A	0.8068	0.6061	0.1278	0.029000
H10B	0.9616	0.7245	0.1582	0.029000
H11A	-0.0192	0.6207	0.0491	0.045000
H11B	0.0822	0.3933	0.0565	0.045000
H11C	0.0501	0.5067	0.0060	0.045000
H12A	0.6641	0.8349	0.2761	0.048000
H12B	0.5200	0.6536	0.2654	0.048000
H15A	0.4994	0.6056	0.1450	0.046000
H15B	0.3854	0.6871	0.1818	0.046000
H15C	0.4802	0.4546	0.1895	0.046000
H17	0.9925	0.2774	0.1701	0.037000
H18	1.2317	0.0704	0.1766	0.047000
H19	1.4018	0.0530	0.2481	0.067000
H20	1.3260	0.2302	0.3135	0.087000
H21	1.0792	0.4207	0.3084	0.069000
H23	0.7982	0.6424	0.4385	0.149000
H24	1.0412	0.7485	0.4771	0.128000
H26	1.2595	0.2201	0.4311	0.085000
H27	1.0135	0.0896	0.3914	0.080000
H28A	1.4159	0.5308	0.4639	0.239000
H28B	1.3514	0.5087	0.5131	0.239000
H28C	1.3280	0.7379	0.4846	0.239000

2.Confirmation of structure for cycloisomerization side product 5ai

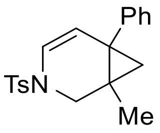

5ai

An X-ray quality crystal was grown through vapor diffusion technique using chloroform and HPLC-grade hexanes. The product **5ai** was dissolved in a minimum amount of chloroform in a 1-mL vial. The 1-mL vial was then placed in a 20-mL scintillation vial that contained HPLC-grade hexanes (3 mL). The 20-mL scintillation vial was tightly sealed with a cap and maintained at rt for crystal growth. Crystals of **5ai** were formed after 2d.

A chunk-like specimen of $C_{20}H_{21}NO_2S$, approximate dimensions 0.100 mm x 0.140 mm x 0.140 mm, was used for the X-ray crystallographic analysis. The X-ray intensity data were measured on a Bruker Apex II CCD system (Cu IMuS, λ = 1.54178 Å). The total exposure time was 12.25 hours. The frames were integrated with the Bruker SAINT software package using a narrow-frame algorithm. The integration of the data using a monoclinic unit cell yielded a total of 23975 reflections to a maximum θ angle of 68.58° (0.83 Å resolution), of which 3221 were independent (average redundancy 7.443, completeness = 98.6%, R_{int} = 2.89%, R_{sig} = 2.12%) and 2435 (75.60%) were greater than $2\sigma(F^2)$. The final cell constants of a = 9.9459(16) Å, b = 8.9977(14) Å, c = 19.839(3) Å, β = 92.339(10)°, volume = 1773.9(5) $Å^3$, are based upon the refinement of the XYZ-centroids of 6363 reflections above 20 σ(I) with 8.922° < 2θ < 133.3°. Data was corrected for absorption effects using the Multi-Scan method (SADABS). The ratio of minimum to maximum apparent transmission was 0.834. The calculated minimum and maximum transmission coefficients (based on crystal size) are 0.6300 and 0.7500. The structure was solved and refined using the Bruker SHELXTL Software Package, using the space group P 1 21/n 1, with Z = 4 for the formula unit, $C_{20}H_{21}NO_2S$. The final anisotropic full-matrix least-squares refinement on F^2 with 217 variables converged at R1 = 8.07%, for the observed data and wR2 = 23.29% for all data. The goodness-of-fit was 2.252. The largest peak in the final difference electron density synthesis was 1.031 $e^-/Å^3$ and the largest hole was -0.557 $e^-/Å^3$ with an RMS deviation of 0.070 $e^-/Å^3$. On the basis of the final model, the calculated density was 1.271 g/cm^3 and F(000), 720 e^-.

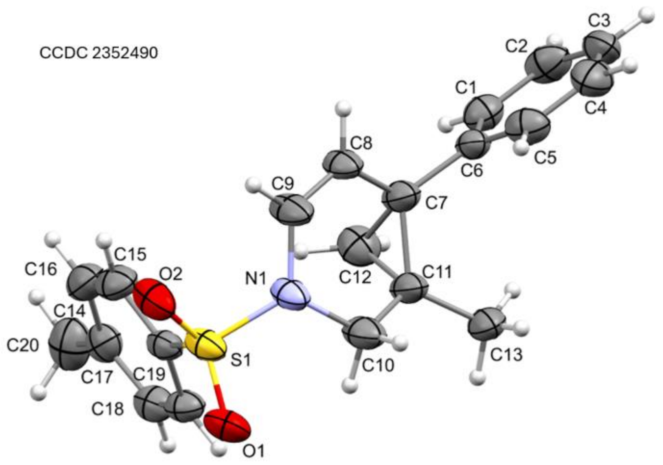

Table S18. Sample and crystal data for Yifan1

**Identification code**	Yifan1	
**Chemical formula**	$C_{20}H_{21}NO_2S$	
**Formula weight**	339.44 g/mol	
**Temperature**	220(2) K	
**Wavelength**	1.54178 Å	
**Crystal size**	0.100 x 0.140 x 0.140 mm	
**Crystal system**	monoclinic	
**Space group**	P 1 21/n 1	
**Unit cell dimensions**	a = 9.9459(16) Å	α = 90°
	b = 8.9977(14) Å	β = 92.339(10)°
	c = 19.839(3) Å	γ = 90°
**Volume**	1773.9(5) Å^3	
**Z**	4	
**Density (calculated)**	1.271 g/cm^3	
**Absorption coefficient**	1.705 mm^{-1}	
**F(000)**	720	

Table S19. Data collection and structure refinement for Yifan1.

**Diffractometer**	Bruker Apex II CCD	
**Theta range for data collection**	4.46 to 68.58°	
**Index ranges**	-11<=h<=11, -10<=k<=9, -23<=l<=23	
**Reflections collected**	23975	
**Independent reflections**	3221 [R(int) = 0.0289]	
**Coverage of independent reflections**	98.6%	
**Absorption correction**	Multi-Scan	
**Max. and min. transmission**	0.7500 and 0.6300	
**Structure solution technique**	direct methods	
**Structure solution program**	SHELXT 2014/5 (Sheldrick, 2014)	
**Refinement method**	Full-matrix least-squares on F^2	
**Refinement program**	SHELXL-2018/3 (Sheldrick, 2018)	
**Function minimized**	Σ w(F_o^2 - F_c^2)^2	
**Data / restraints / parameters**	3221 / 0 / 217	
**Goodness-of-fit on F^2**	2.252	
**Final R indices**	2435 data; I>2σ(I)	R1 = 0.0807, wR2 = 0.2251
	all data	R1 = 0.1031, wR2 = 0.2329
**Weighting scheme**	w=1/[σ^2(F_o^2)+(0.0680P)^2]<br>where P=(F_o^2+2F_c^2)/3	
**Largest diff. peak and hole**	1.031 and -0.557 eÅ^{-3}	
**R.M.S. deviation from mean**	0.070 eÅ^{-3}	

Table S20. Atomic coordinates and equivalent isotropic atomic displacement parameters (Å^2) for Yifan1.

U(eq) is defined as one third of the trace of the orthogonalized U_{ij} tensor.

	**x/a**	**y/b**	**z/c**	**U(eq)**
S1	0.73719(11)	0.99291(10)	0.58081(4)	0.0584(4)
O1	0.6423(3)	0.0857(3)	0.54498(13)	0.0667(8)
N1	0.6492(3)	0.8772(3)	0.62514(14)	0.0583(9)
C1	0.5333(4)	0.3118(5)	0.6780(2)	0.0634(11)
O2	0.8356(3)	0.0553(3)	0.62641(13)	0.0747(9)
C2	0.4763(5)	0.1950(5)	0.7116(3)	0.0753(14)
C3	0.3960(5)	0.2229(5)	0.7620(3)	0.0819(16)
C4	0.3713(5)	0.3665(6)	0.7820(2)	0.0805(14)
C5	0.4286(5)	0.4809(4)	0.7481(2)	0.0672(12)
C6	0.5095(4)	0.4548(4)	0.69592(19)	0.0492(9)
C7	0.5747(4)	0.5807(4)	0.6598(2)	0.0625(11)
C8	0.6718(4)	0.6719(4)	0.70003(18)	0.0576(10)
C9	0.7072(5)	0.8040(5)	0.6820(2)	0.0778(14)
C10	0.5176(5)	0.8224(5)	0.5990(2)	0.0730(13)
C11	0.4969(4)	0.6631(4)	0.6035(2)	0.0647(12)
C12	0.6166(5)	0.5621(5)	0.5847(2)	0.0699(12)
C13	0.3602(4)	0.6125(5)	0.5832(2)	0.0702(12)
C14	0.8194(4)	0.8843(4)	0.52110(17)	0.0518(10)
C15	0.9421(4)	0.8224(5)	0.5381(2)	0.0690(12)
C16	0.0050(5)	0.7334(5)	0.4915(3)	0.0760(14)
C17	0.9458(4)	0.7051(4)	0.4284(2)	0.0613(11)
C18	0.8217(5)	0.7665(4)	0.4136(2)	0.0629(11)
C19	0.7570(4)	0.8562(4)	0.45882(19)	0.0574(10)
C20	0.0135(5)	0.6126(5)	0.3773(3)	0.0912(15)

Table S21. Bond lengths (Å) for Yifan.

S1-O2	1.422(3)	S1-O1	1.428(3)
S1-N1	1.639(3)	S1-C14	1.762(4)
N1-C9	1.409(5)	N1-C10	1.473(5)
C1-C6	1.358(5)	C1-C2	1.379(6)
C1-H1A	0.94	C2-C3	1.329(7)
C2-H2A	0.94	C3-C4	1.376(7)
C3-H3A	0.94	C4-C5	1.366(6)
C4-H4A	0.94	C5-C6	1.357(6)
C5-H5A	0.94	C6-C7	1.502(5)
C7-C8	1.477(5)	C7-C11	1.525(5)
C7-C12	1.571(6)	C8-C9	1.295(5)
C8-H8A	0.94	C9-H9A	0.94
C10-C11	1.452(5)	C10-H10A	0.98
C10-H10B	0.98	C11-C13	1.474(5)
C11-C12	1.556(6)	C12-H12A	0.98
C12-H12B	0.98	C13-H13A	0.97
C13-H13B	0.97	C13-H13C	0.97
C14-C15	1.372(5)	C14-C19	1.383(5)
C15-C16	1.390(6)	C15-H15A	0.94
C16-C17	1.384(6)	C16-H16A	0.94
C17-C18	1.373(6)	C17-C20	1.493(6)
C18-C19	1.384(6)	C18-H18A	0.94
C19-H19A	0.94	C20-H20A	0.97
C20-H20B	0.97	C20-H20C	0.97

Table S22. Bond angles (°) for Yifan.

O2-S1-O1	120.66(18)	O2-S1-N1	106.17(15)
O1-S1-N1	106.41(17)	O2-S1-C14	108.70(18)
O1-S1-C14	107.74(16)	N1-S1-C14	106.31(17)
C9-N1-C10	117.1(3)	C9-N1-S1	121.1(3)
C10-N1-S1	120.6(2)	C6-C1-C2	121.1(4)
C6-C1-H1A	119.5	C2-C1-H1A	119.5
C3-C2-C1	119.4(4)	C3-C2-H2A	120.3
C1-C2-H2A	120.3	C2-C3-C4	120.9(4)
C2-C3-H3A	119.6	C4-C3-H3A	119.6
C5-C4-C3	118.9(5)	C5-C4-H4A	120.5
C3-C4-H4A	120.5	C6-C5-C4	121.1(4)
C6-C5-H5A	119.4	C4-C5-H5A	119.4
C5-C6-C1	118.6(4)	C5-C6-C7	121.0(4)
C1-C6-C7	120.4(4)	C8-C7-C6	116.5(3)
C8-C7-C11	115.2(3)	C6-C7-C11	120.0(3)
C8-C7-C12	112.3(3)	C6-C7-C12	120.6(3)
C11-C7-C12	60.3(3)	C9-C8-C7	122.7(3)
C9-C8-H8A	118.6	C7-C8-H8A	118.6
C8-C9-N1	122.9(3)	C8-C9-H9A	118.6
N1-C9-H9A	118.6	C11-C10-N1	115.8(3)
C11-C10-H10A	108.3	N1-C10-H10A	108.3
C11-C10-H10B	108.3	N1-C10-H10B	108.3
H10A-C10-H10B	107.4	C10-C11-C13	114.8(3)
C10-C11-C7	117.2(3)	C13-C11-C7	119.0(3)
C10-C11-C12	116.7(4)	C13-C11-C12	117.4(4)
C7-C11-C12	61.3(3)	C11-C12-C7	58.4(2)

C11-C12-H12A	117.9	C7-C12-H12A	117.9
C11-C12-H12B	117.9	C7-C12-H12B	117.9
H12A-C12-H12B	115.1	C11-C13-H13A	109.5
C11-C13-H13B	109.5	H13A-C13-H13B	109.5
C11-C13-H13C	109.5	H13A-C13-H13C	109.5
H13B-C13-H13C	109.5	C15-C14-C19	120.5(4)
C15-C14-S1	119.5(3)	C19-C14-S1	119.9(3)
C14-C15-C16	119.4(4)	C14-C15-H15A	120.3
C16-C15-H15A	120.3	C17-C16-C15	121.4(4)
C17-C16-H16A	119.3	C15-C16-H16A	119.3
C18-C17-C16	117.7(4)	C18-C17-C20	120.3(4)
C16-C17-C20	122.0(4)	C17-C18-C19	122.3(4)
C17-C18-H18A	118.9	C19-C18-H18A	118.9
C14-C19-C18	118.8(4)	C14-C19-H19A	120.6
C18-C19-H19A	120.6	C17-C20-H20A	109.5
C17-C20-H20B	109.5	H20A-C20-H20B	109.5
C17-C20-H20C	109.5	H20A-C20-H20C	109.5
H20B-C20-H20C	109.5		

Table S23. Anisotropic atomic displacement parameters (Å^2) for Yifan1.

The anisotropic atomic displacement factor exponent takes the form: $-2\pi^2[h^2 a^{*2} U_{11} + ... + 2 h k a^* b^* U_{12}]$

	U_{11}	U_{22}	U_{33}	U_{23}	U_{13}	U_{12}
S1	0.0864(8)	0.0441(6)	0.0436(6)	0.0053(3)	-0.0093(5)	-0.0162(4)
O1	0.100(2)	0.0474(15)	0.0526(16)	0.0111(11)	-0.0005(15)	0.0020(13)
N1	0.077(2)	0.0570(18)	0.0393(16)	0.0084(13)	-0.0123(15)	-0.0184(16)
C1	0.061(3)	0.071(3)	0.057(3)	-0.0028(19)	-0.006(2)	0.0047(19)
O2	0.108(2)	0.0632(17)	0.0512(16)	0.0008(13)	-0.0113(16)	-0.0379(16)

	U_{11}	U_{22}	U_{33}	U_{23}	U_{13}	U_{12}
C2	0.077(3)	0.046(2)	0.101(4)	-0.003(2)	-0.021(3)	0.006(2)
C3	0.073(3)	0.074(3)	0.096(4)	0.042(3)	-0.022(3)	-0.021(2)
C4	0.077(3)	0.105(4)	0.059(3)	0.015(3)	0.003(2)	0.004(3)
C5	0.080(3)	0.052(2)	0.068(3)	-0.0038(19)	-0.012(2)	0.0043(19)
C6	0.049(2)	0.044(2)	0.054(2)	0.0094(15)	-0.0107(18)	0.0000(15)
C7	0.061(3)	0.056(2)	0.069(3)	0.0146(18)	-0.022(2)	-0.0068(17)
C8	0.071(3)	0.058(2)	0.042(2)	0.0081(16)	-0.0194(19)	-0.0116(18)
C9	0.098(3)	0.076(3)	0.056(3)	0.020(2)	-0.038(2)	-0.037(2)
C10	0.088(3)	0.072(3)	0.057(3)	0.019(2)	-0.021(2)	-0.013(2)
C11	0.063(3)	0.060(2)	0.069(3)	0.0216(19)	-0.024(2)	-0.0118(18)
C12	0.082(3)	0.071(3)	0.057(3)	-0.007(2)	0.004(2)	-0.004(2)
C13	0.061(3)	0.081(3)	0.066(3)	0.020(2)	-0.021(2)	-0.012(2)
C14	0.066(3)	0.0422(19)	0.046(2)	0.0094(14)	-0.0119(18)	-0.0133(16)
C15	0.070(3)	0.069(3)	0.066(3)	0.005(2)	-0.026(2)	-0.009(2)
C16	0.060(3)	0.072(3)	0.095(4)	0.016(2)	-0.016(3)	-0.004(2)
C17	0.066(3)	0.043(2)	0.075(3)	0.0013(18)	-0.002(2)	-0.0097(18)
C18	0.081(3)	0.052(2)	0.054(2)	0.0003(17)	-0.012(2)	-0.014(2)
C19	0.067(3)	0.053(2)	0.051(2)	0.0044(17)	-0.0142(19)	-0.0025(17)
C20	0.085(4)	0.067(3)	0.123(4)	-0.011(3)	0.014(3)	-0.006(2)

Table S24. Hydrogen atomic coordinates and isotropic atomic displacement parameters (Å^2) for Yifan1.

	x/a	y/b	z/c	U(eq)
H1A	0.5896	0.2922	0.6422	0.076
H2A	0.4941	0.0965	0.6989	0.09
H3A	0.3555	0.1435	0.7844	0.098
H4A	0.3159	0.3855	0.8182	0.097

	x/a	y/b	z/c	U(eq)
H5A	0.4117	0.5793	0.7612	0.081
H8A	0.7094	0.6324	0.7404	0.069
H9A	0.7746	0.8533	0.7080	0.093
H10A	0.4471	0.8724	0.6236	0.088
H10B	0.5063	0.8517	0.5515	0.088
H12A	0.6997	0.6104	0.5714	0.084
H12B	0.5958	0.4676	0.5620	0.084
H13A	0.3551	0.5054	0.5878	0.105
H13B	0.2955	0.6588	0.6119	0.105
H13C	0.3402	0.6400	0.5366	0.105
H15A	0.9834	0.8399	0.5808	0.083
H16A	1.0894	0.6916	0.5030	0.091
H18A	0.7792	0.7469	0.3714	0.076
H19A	0.6722	0.8972	0.4474	0.069
H20A	1.0996	0.5784	0.3960	0.137
H20B	0.9573	0.5277	0.3654	0.137
H20C	1.0274	0.6718	0.3373	0.137

^{1}H and ^{13}C NMR spectra

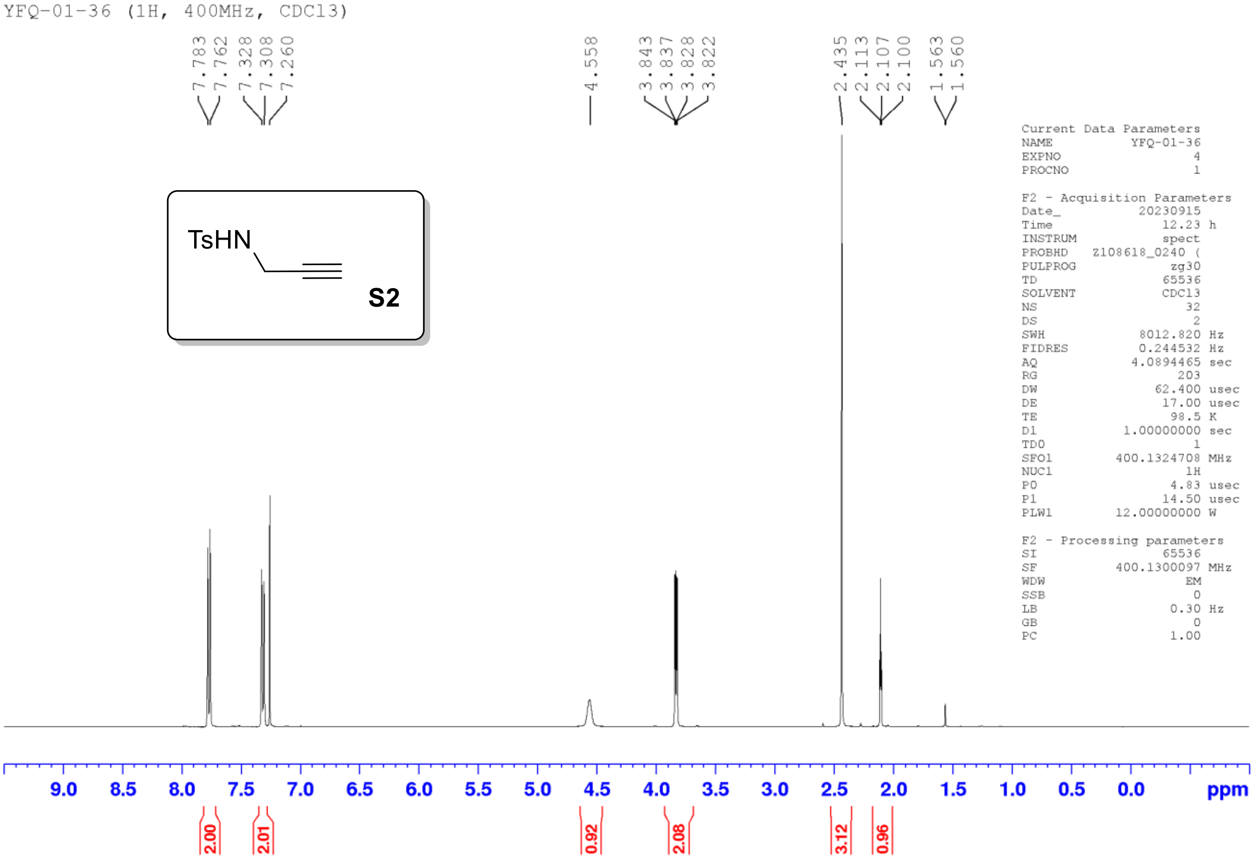

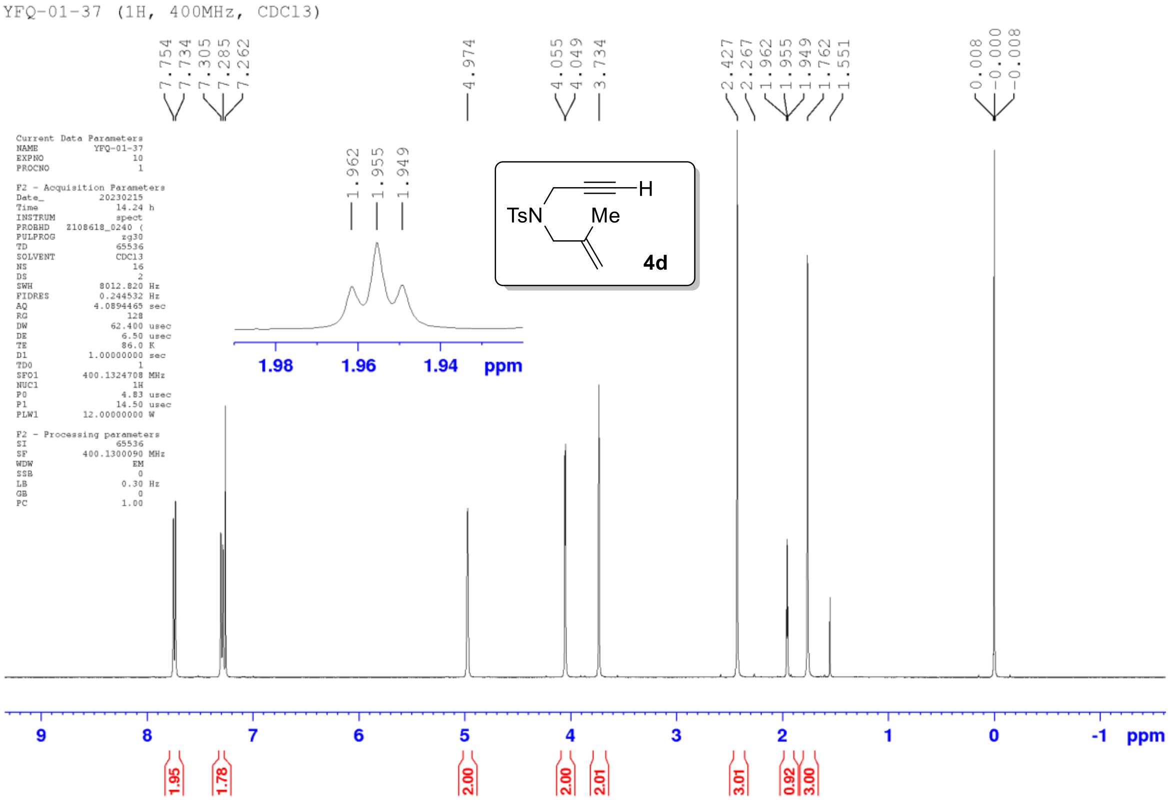
YFQ-01-37 (1H, 400MHz, CDCl3)
7.754
7.734
7.305
7.285
7.262
4.974
4.055
4.049
3.734
2.427
2.267
1.962
1.955
1.949
1.762
1.551
0.008
-0.000
-0.008
TsN
Me
H
4d
1.98
1.96
1.94
ppm
9
8
7
6
5
4
3
2
1
0
-1
ppm
1.95
1.78
2.00
2.00
2.01
3.01
0.92
3.00

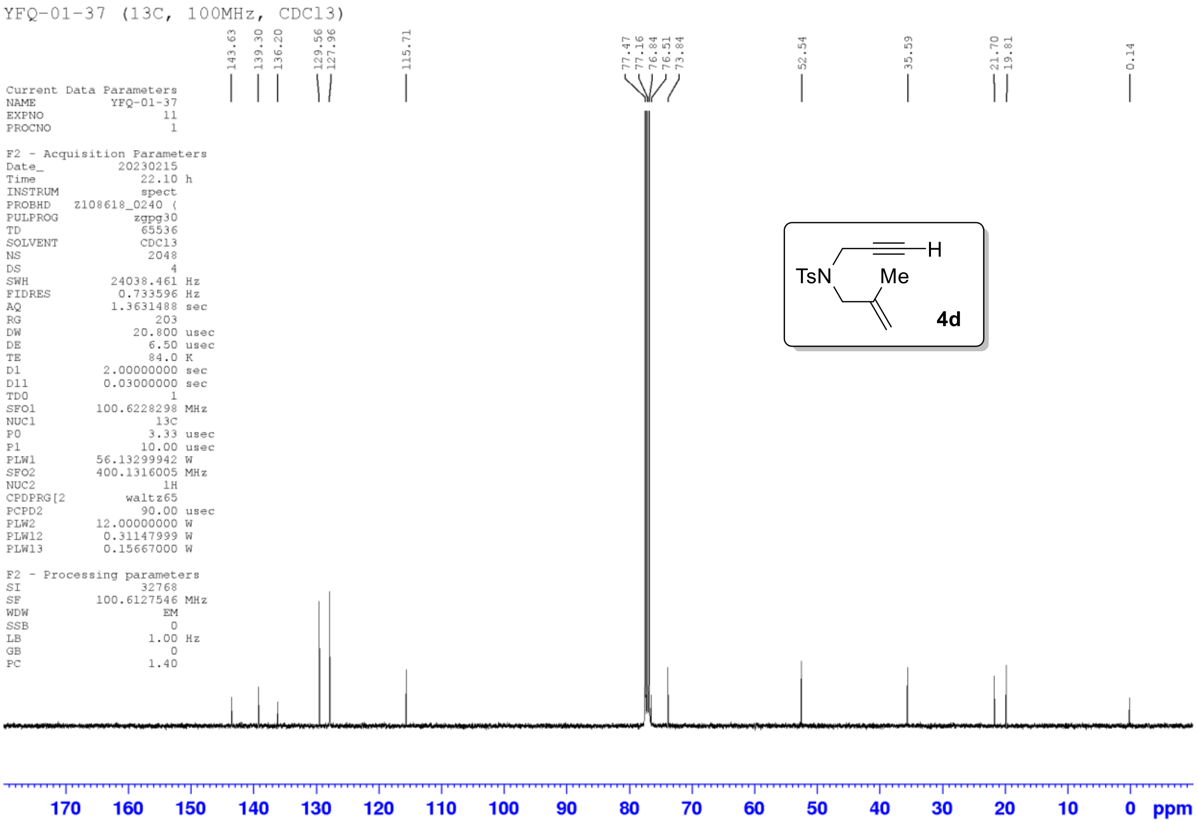
YFQ-01-37 (13C, 100MHz, CDCl3)
143.63
139.30
136.20
129.56
127.96
115.71
77.47
77.16
76.84
76.51
73.84
52.54
35.59
21.70
19.81
0.14
TsN
Me
H
4d
170
160
150
140
130
120
110
100
90
80
70
60
50
40
30
20
10
0
ppm

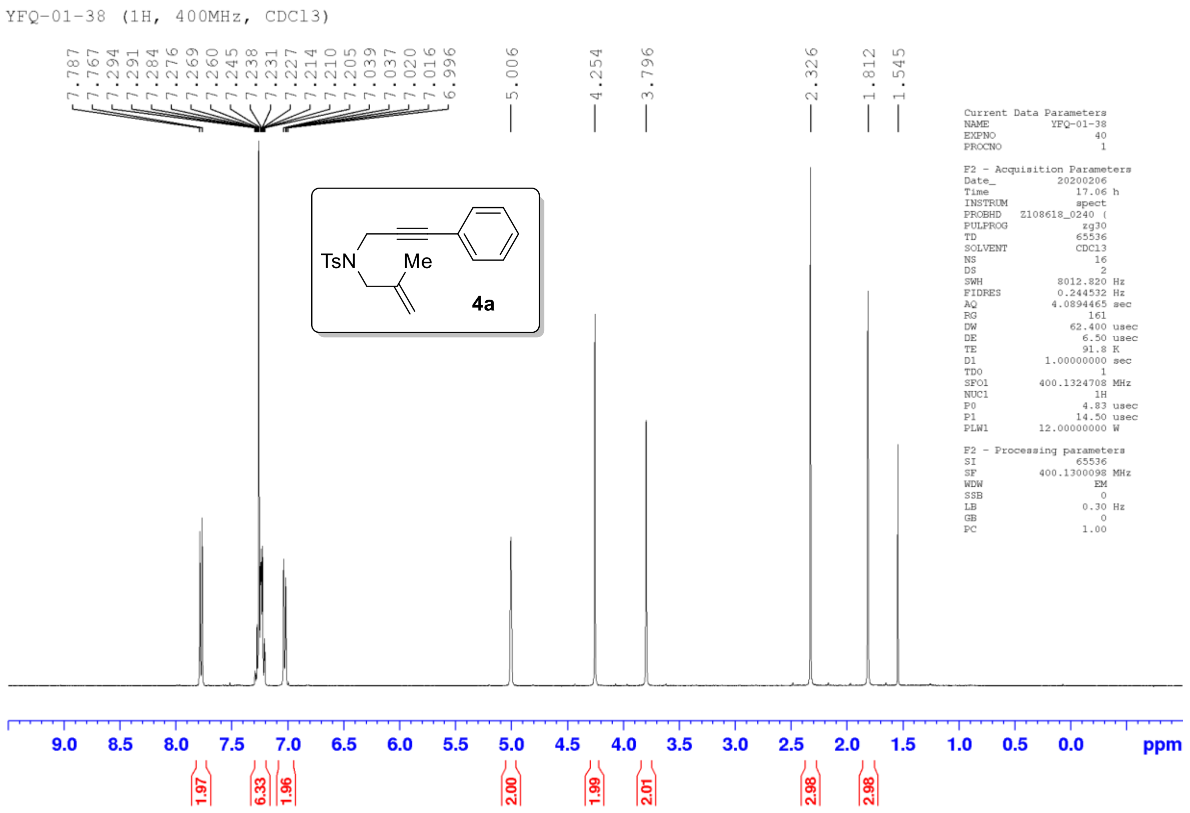
YFQ-01-38 (1H, 400MHz, CDCl3)
7.787 7.767 7.294 7.291 7.284 7.276 7.269 7.260 7.245 7.238 7.231 7.227 7.214 7.210 7.205 7.039 7.037 7.020 7.016 6.996
5.006
4.254
3.796
2.326
1.812
1.545
TsN
Me
4a
Current Data Parameters
NAME YFQ-01-38
EXPNO 40
PROCNO 1
F2 - Acquisition Parameters
Date_ 20200206
Time 17.06 h
INSTRUM spect
PROBHD Z108618_0240 (
PULPROG zg30
TD 65536
SOLVENT CDCl3
NS 16
DS 2
SWH 8012.820 Hz
FIDRES 0.244532 Hz
AQ 4.0894465 sec
RG 161
DW 62.400 usec
DE 6.50 usec
TE 91.8 K
D1 1.00000000 sec
TD0 1
SFO1 400.1324708 MHz
NUC1 1H
P0 4.83 usec
P1 14.50 usec
PLW1 12.00000000 W
F2 - Processing parameters
SI 65536
SF 400.1300098 MHz
WDW EM
SSB 0
LB 0.30 Hz
GB 0
PC 1.00
9.0 8.5 8.0 7.5 7.0 6.5 6.0 5.5 5.0 4.5 4.0 3.5 3.0 2.5 2.0 1.5 1.0 0.5 0.0 ppm
1.97 6.33 1.96 2.00 1.99 2.01 2.98 2.98

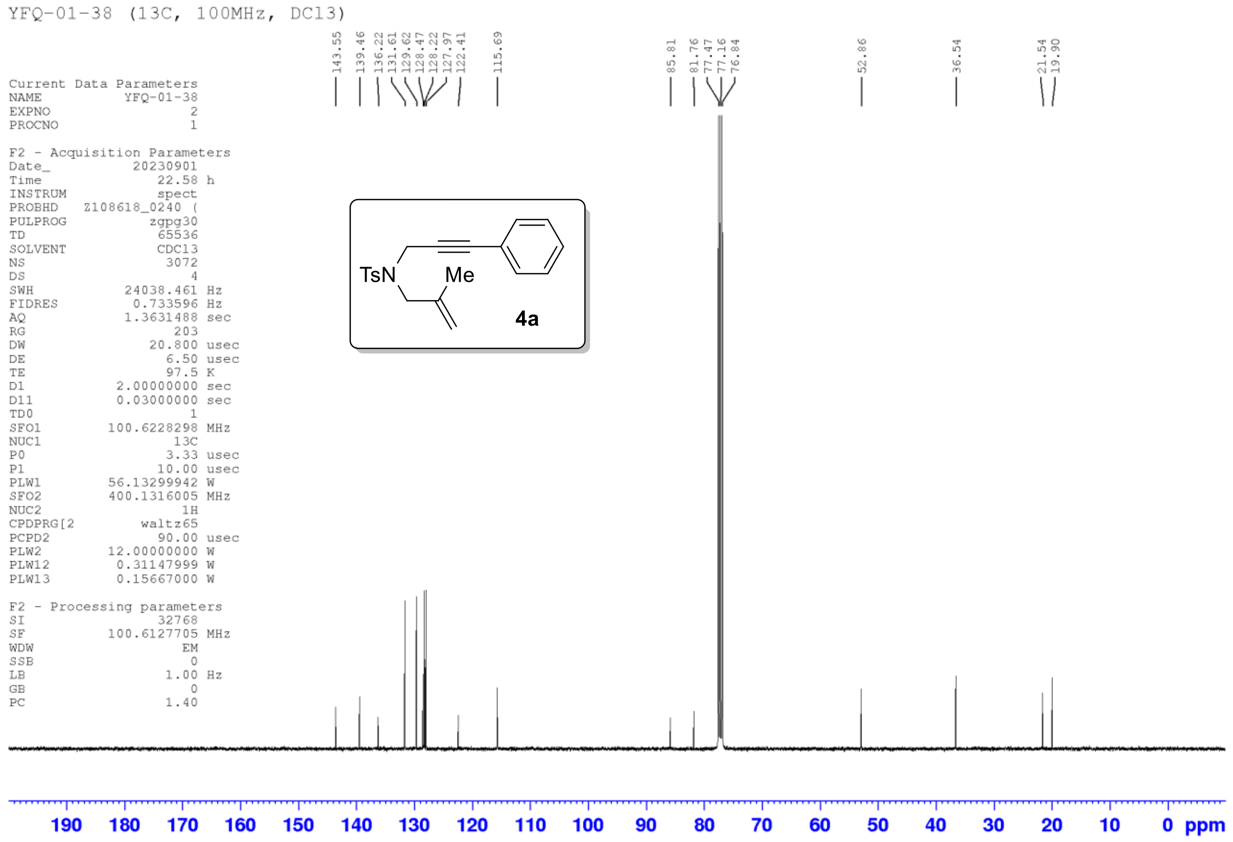
YFQ-01-38 (13C, 100MHz, DC13)
143.55 139.46 136.22 131.61 129.62 128.47 128.22 127.97 122.41 115.69
85.81 81.76 77.47 77.16 76.84
52.86
36.54
21.54 19.90
Current Data Parameters
NAME YFQ-01-38
EXPNO 2
PROCNO 1
F2 - Acquisition Parameters
Date_ 20230901
Time 22.58 h
INSTRUM spect
PROBHD Z108618_0240 (
PULPROG zgpg30
TD 65536
SOLVENT CDCl3
NS 3072
DS 4
SWH 24038.461 Hz
FIDRES 0.733596 Hz
AQ 1.3631488 sec
RG 203
DW 20.800 usec
DE 6.50 usec
TE 97.5 K
D1 2.00000000 sec
D11 0.03000000 sec
TD0 1
SFO1 100.6228298 MHz
NUC1 13C
P0 3.33 usec
P1 10.00 usec
PLW1 56.13299942 W
SFO2 400.1316005 MHz
NUC2 1H
CPDPRG[2 waltz65
PCPD2 90.00 usec
PLW2 12.00000000 W
PLW12 0.31147999 W
PLW13 0.15667000 W
F2 - Processing parameters
SI 32768
SF 100.6127705 MHz
WDW EM
SSB 0
LB 1.00 Hz
GB 0
PC 1.40
TsN
Me
4a
190 180 170 160 150 140 130 120 110 100 90 80 70 60 50 40 30 20 10 0 ppm

YFQ-04-84 (1H, 400MHz, CDCl3)

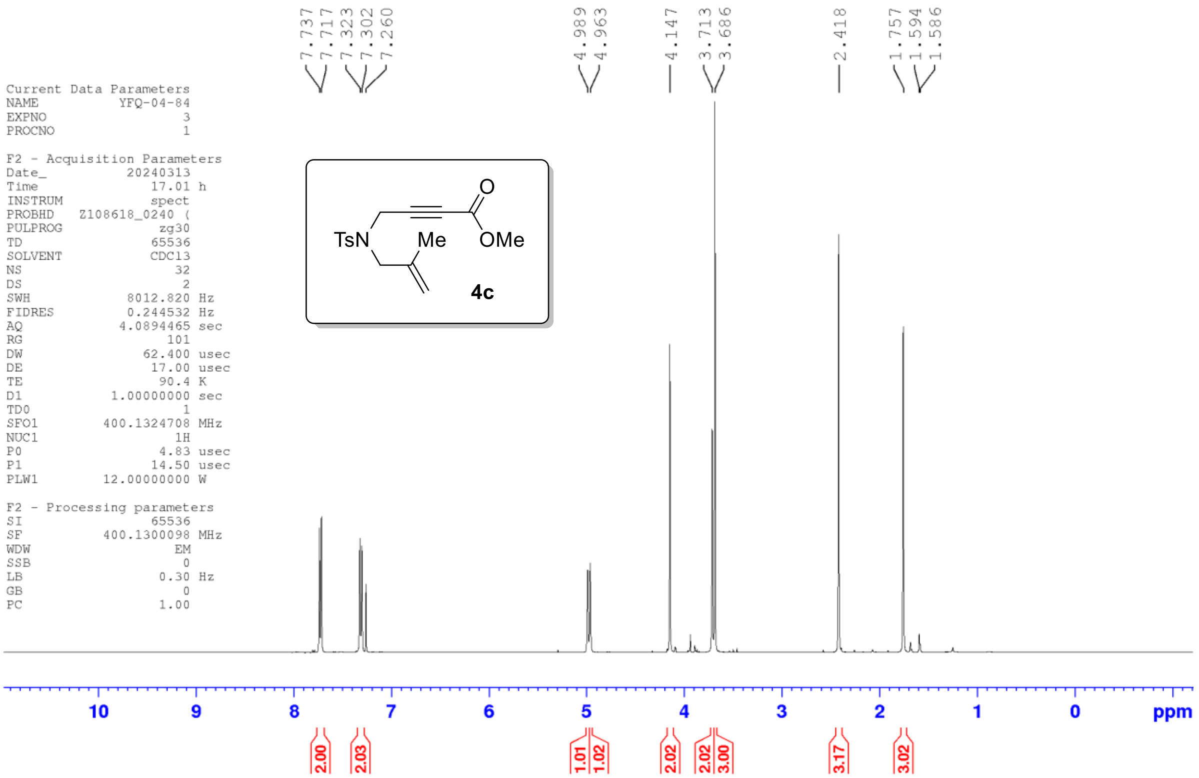

YFQ-04-84 (13C, 100 MHz, CDCl3)

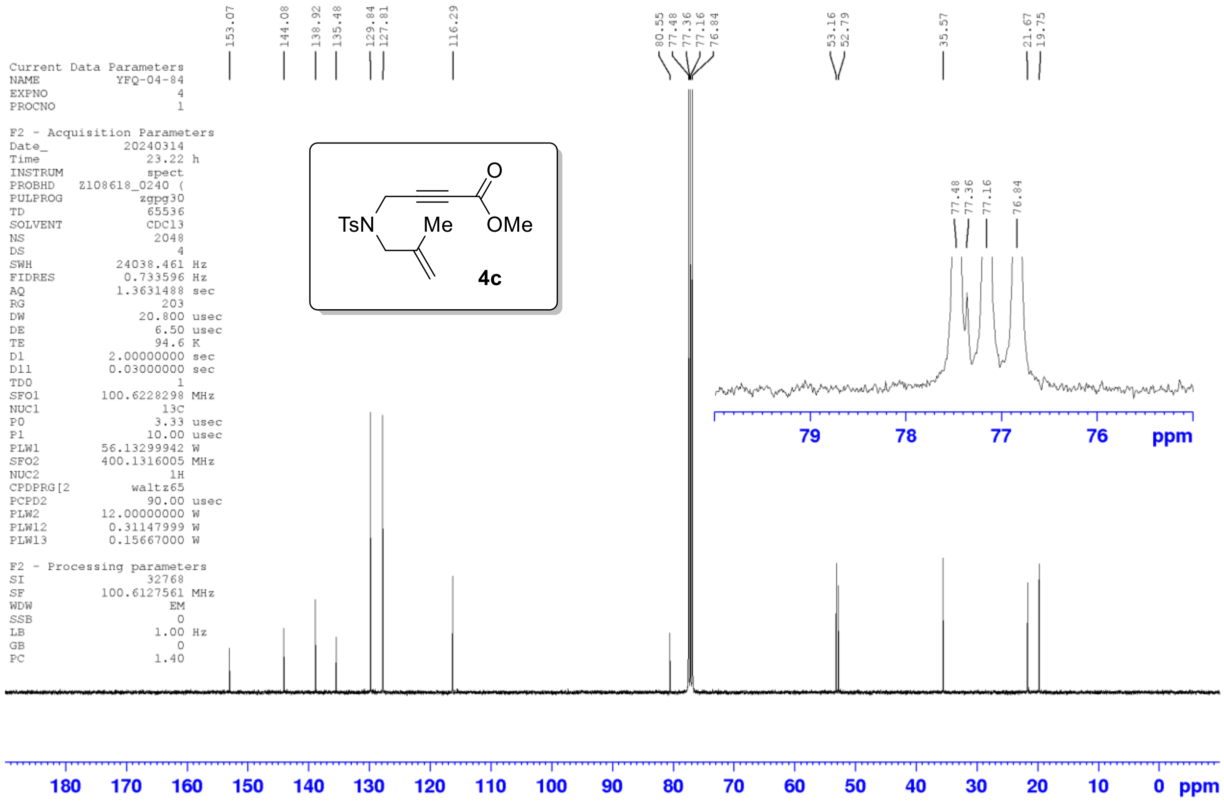

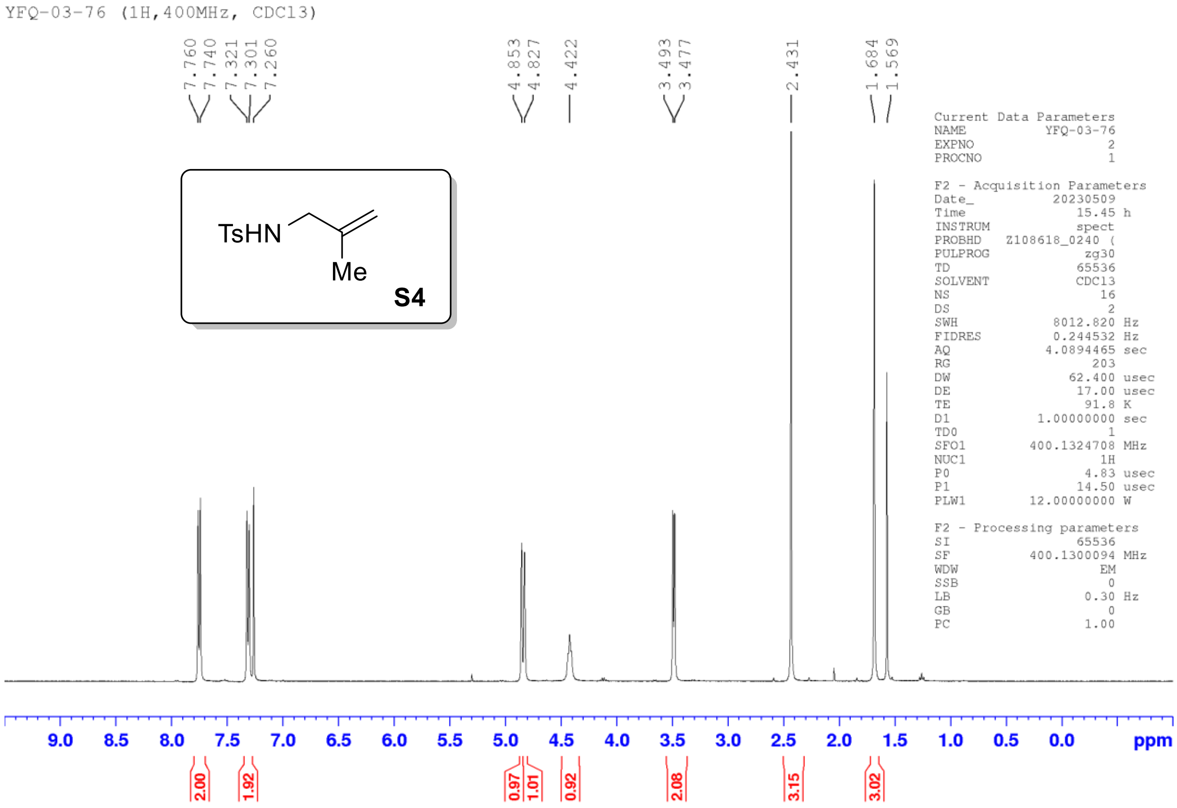
YFQ-03-76 (1H,400MHz, CDCl3)
7.760
7.740
7.321
7.301
7.260
4.853
4.827
4.422
3.493
3.477
2.431
1.684
1.569
TsHN
Me
S4
Current Data Parameters
NAME YFQ-03-76
EXPNO 2
PROCNO 1
F2 - Acquisition Parameters
Date_ 20230509
Time 15.45 h
INSTRUM spect
PROBHD Z108618_0240 (
PULPROG zg30
TD 65536
SOLVENT CDCl3
NS 16
DS 2
SWH 8012.820 Hz
FIDRES 0.244532 Hz
AQ 4.0894465 sec
RG 203
DW 62.400 usec
DE 17.00 usec
TE 91.8 K
D1 1.00000000 sec
TD0 1
SFO1 400.1324708 MHz
NUC1 1H
P0 4.83 usec
P1 14.50 usec
PLW1 12.00000000 W
F2 - Processing parameters
SI 65536
SF 400.1300094 MHz
WDW EM
SSB 0
LB 0.30 Hz
GB 0
PC 1.00
9.0 8.5 8.0 7.5 7.0 6.5 6.0 5.5 5.0 4.5 4.0 3.5 3.0 2.5 2.0 1.5 1.0 0.5 0.0 ppm
2.00
1.92
0.97
1.01
0.92
2.08
3.15
3.02

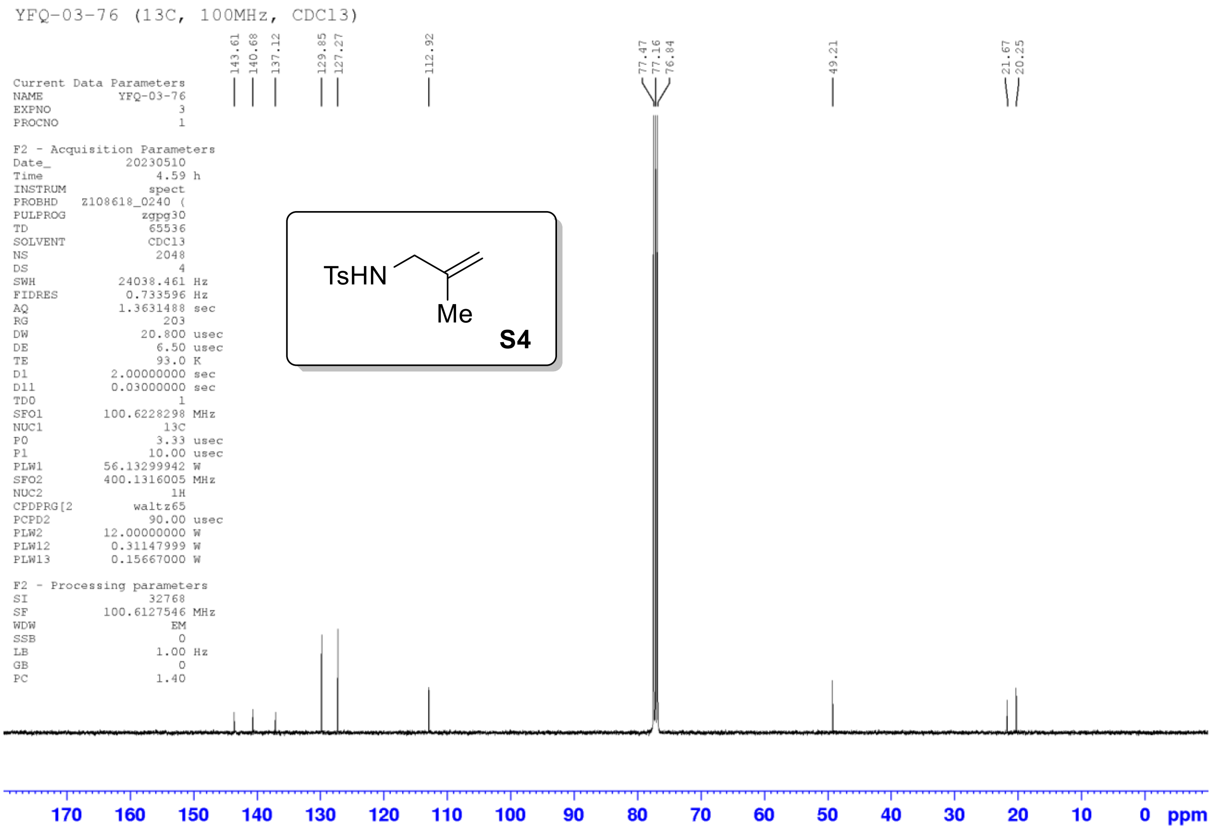
YFQ-03-76 (13C, 100MHz, CDCl3)
143.61
140.68
137.12
129.85
127.27
112.92
77.47
77.16
76.84
49.21
21.67
20.25
Current Data Parameters
NAME YFQ-03-76
EXPNO 3
PROCNO 1
F2 - Acquisition Parameters
Date_ 20230510
Time 4.59 h
INSTRUM spect
PROBHD Z108618_0240 (
PULPROG zgpg30
TD 65536
SOLVENT CDCl3
NS 2048
DS 4
SWH 24038.461 Hz
FIDRES 0.733596 Hz
AQ 1.3631488 sec
RG 203
DW 20.800 usec
DE 6.50 usec
TE 93.0 K
D1 2.00000000 sec
D11 0.03000000 sec
TD0 1
SFO1 100.6228298 MHz
NUC1 13C
P0 3.33 usec
P1 10.00 usec
PLW1 56.13299942 W
SFO2 400.1316005 MHz
NUC2 1H
CPDPRG[2 waltz65
PCPD2 90.00 usec
PLW2 12.00000000 W
PLW12 0.31147999 W
PLW13 0.15667000 W
F2 - Processing parameters
SI 32768
SF 100.6127546 MHz
WDW EM
SSB 0
LB 1.00 Hz
GB 0
PC 1.40
TsHN
Me
S4
170 160 150 140 130 120 110 100 90 80 70 60 50 40 30 20 10 0 ppm

YFQ-03-77 (1H, 400MHz, CDCl3)

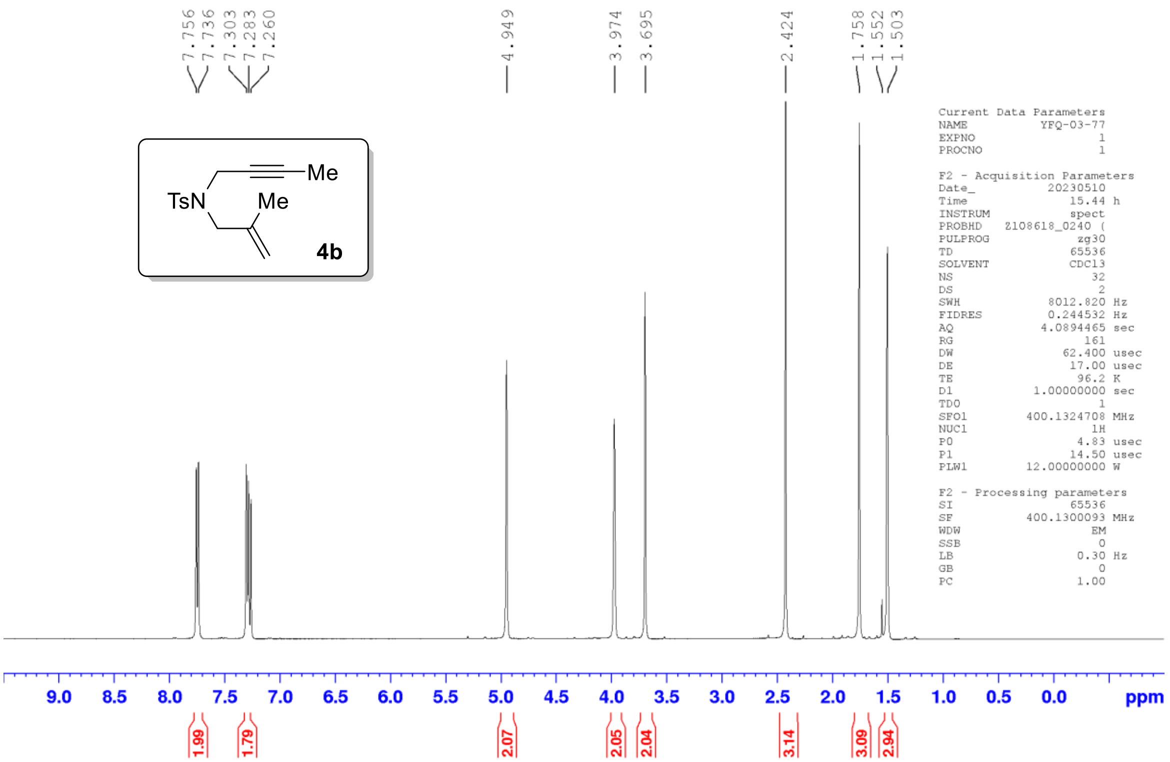

YFQ-03-77 (13C, 100MHz, CDCl3)

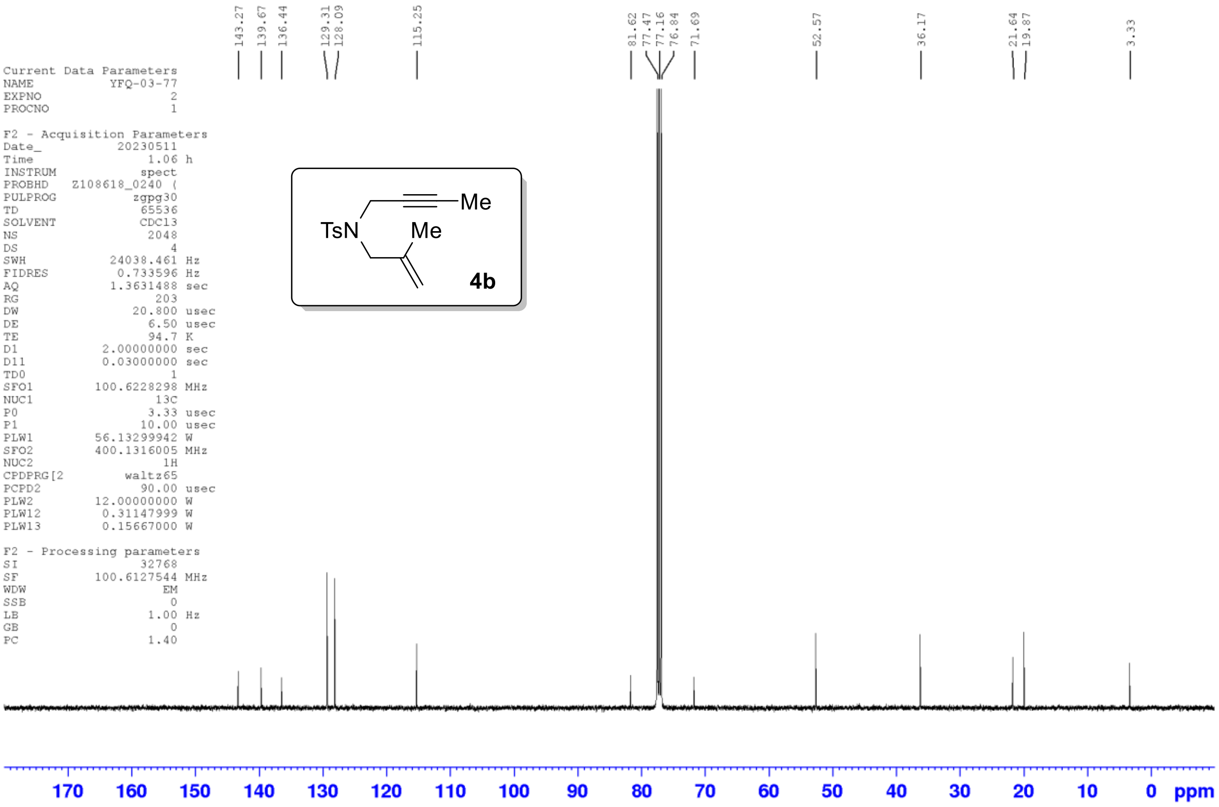

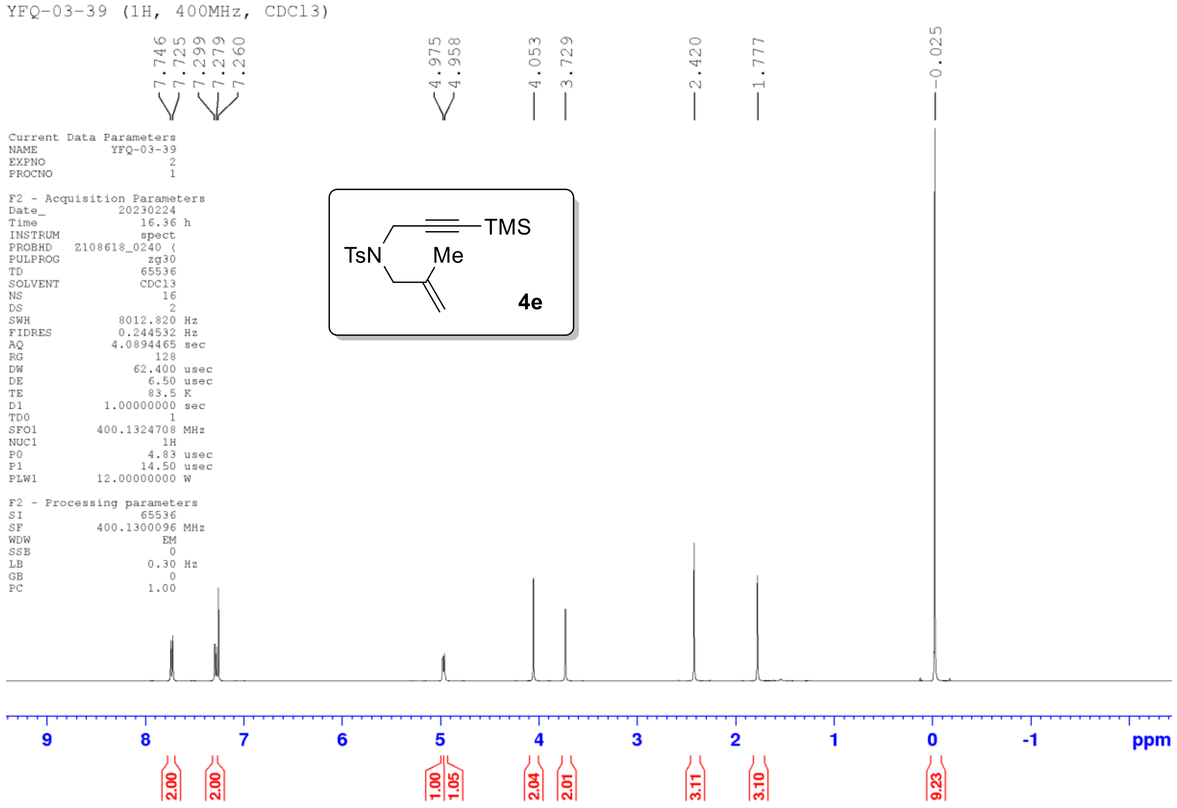

YFQ-03-39 (1H, 400MHz, CDCl3)
7.746
7.725
7.299
7.279
7.260
4.975
4.958
4.053
3.729
2.420
1.777
-0.025
Current Data Parameters
NAME YFQ-03-39
EXPNO 2
PROCNO 1
F2 - Acquisition Parameters
Date_ 20230224
Time 16.36 h
INSTRUM spect
PROBHD Z108618_0240 (
PULPROG zg30
TD 65536
SOLVENT CDCl3
NS 16
DS 2
SWH 8012.820 Hz
FIDRES 0.244532 Hz
AQ 4.0894465 sec
RG 128
DW 62.400 usec
DE 6.50 usec
TE 83.5 K
D1 1.00000000 sec
TD0 1
SFO1 400.1324708 MHz
NUC1 1H
P0 4.83 usec
P1 14.50 usec
PLW1 12.00000000 W
F2 - Processing parameters
SI 65536
SF 400.1300096 MHz
WDW EM
SSB 0
LB 0.30 Hz
GB 0
PC 1.00
TsN
TMS
Me
4e
9
8
7
6
5
4
3
2
1
0
-1
ppm
2.00
2.00
1.00
1.05
2.04
2.01
3.11
3.10
9.23

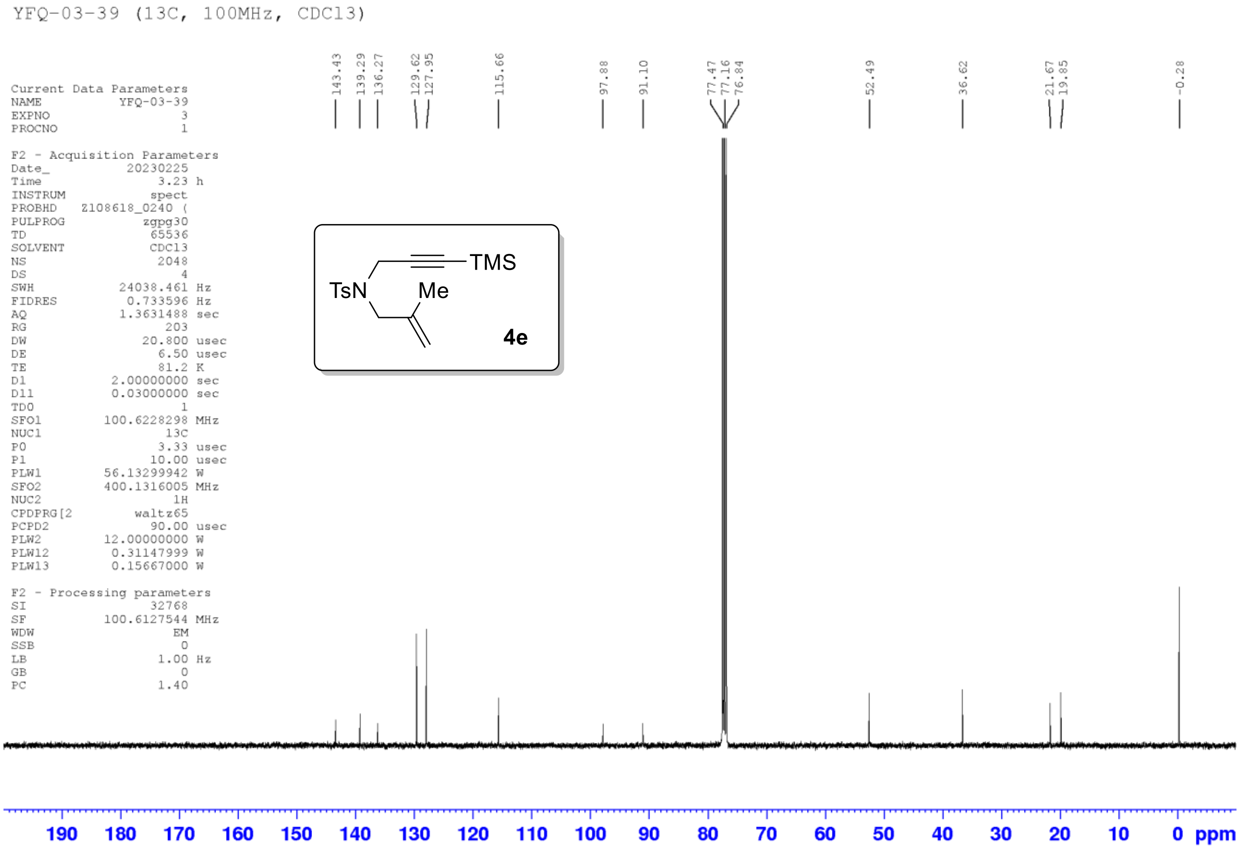

YFQ-03-39 (13C, 100MHz, CDCl3)
143.43
139.29
136.27
129.62
127.95
115.66
97.88
91.10
77.47
77.16
76.84
52.49
36.62
21.67
19.85
-0.28
Current Data Parameters
NAME YFQ-03-39
EXPNO 3
PROCNO 1
F2 - Acquisition Parameters
Date_ 20230225
Time 3.23 h
INSTRUM spect
PROBHD Z108618_0240 (
PULPROG zgpg30
TD 65536
SOLVENT CDCl3
NS 2048
DS 4
SWH 24038.461 Hz
FIDRES 0.733596 Hz
AQ 1.3631488 sec
RG 203
DW 20.800 usec
DE 6.50 usec
TE 81.2 K
D1 2.00000000 sec
D11 0.03000000 sec
TD0 1
SFO1 100.6228298 MHz
NUC1 13C
P0 3.33 usec
P1 10.00 usec
PLW1 56.13299942 W
SFO2 400.1316005 MHz
NUC2 1H
CPDPRG[2 waltz65
PCPD2 90.00 usec
PLW2 12.00000000 W
PLW12 0.31147999 W
PLW13 0.15667000 W
F2 - Processing parameters
SI 32768
SF 100.6127544 MHz
WDW EM
SSB 0
LB 1.00 Hz
GB 0
PC 1.40
TsN
TMS
Me
4e
190
180
170
160
150
140
130
120
110
100
90
80
70
60
50
40
30
20
10
0
ppm

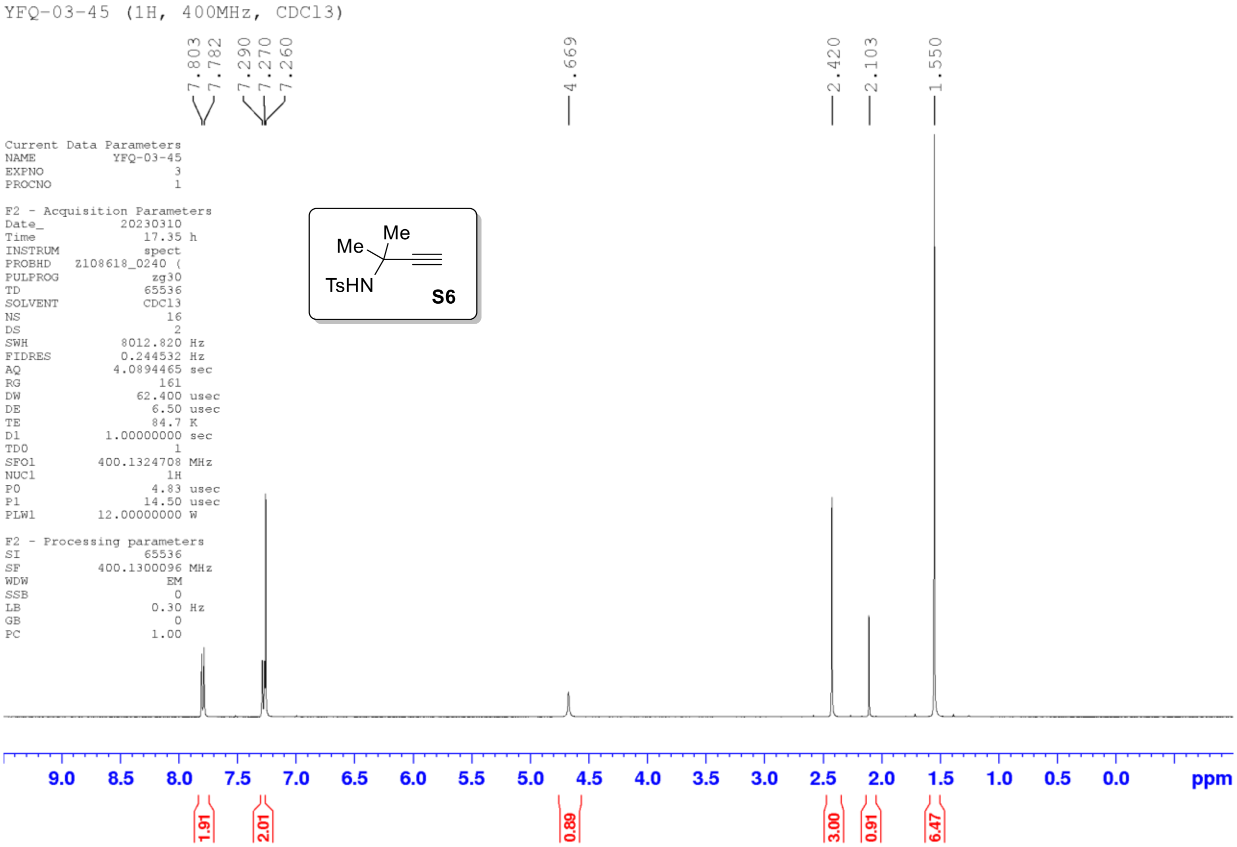
YFQ-03-45 (1H, 400MHz, CDCl3)
7.803
7.782
7.290
7.270
7.260
4.669
2.420
2.103
1.550
Current Data Parameters
NAME YFQ-03-45
EXPNO 3
PROCNO 1
F2 - Acquisition Parameters
Date_ 20230310
Time 17.35 h
INSTRUM spect
PROBHD Z108618_0240 (
PULPROG zg30
TD 65536
SOLVENT CDCl3
NS 16
DS 2
SWH 8012.820 Hz
FIDRES 0.244532 Hz
AQ 4.0894465 sec
RG 161
DW 62.400 usec
DE 6.50 usec
TE 84.7 K
D1 1.00000000 sec
TD0 1
SFO1 400.1324708 MHz
NUC1 1H
P0 4.83 usec
P1 14.50 usec
PLW1 12.00000000 W
F2 - Processing parameters
SI 65536
SF 400.1300096 MHz
WDW EM
SSB 0
LB 0.30 Hz
GB 0
PC 1.00
Me
Me
TsHN
S6
9.0
8.5
8.0
7.5
7.0
6.5
6.0
5.5
5.0
4.5
4.0
3.5
3.0
2.5
2.0
1.5
1.0
0.5
0.0
ppm
1.91
2.01
0.89
3.00
0.91
6.47

YFQ-03-46 (1H, 400MHz, CDCl3)

Current Data Parameters
NAME YFQ-03-46
EXPNO 3
PROCNO 1

F2 - Acquisition Parameters
Date_ 20230316
Time 18.06 h
INSTRUM spect
PROBHD Z108618_0820 (
PULPROG zg30
TD 65536
SOLVENT CDCl3
NS 16
DS 2
SWH 8012.820 Hz
FIDRES 0.244532 Hz
AQ 4.0894465 sec
RG 144
DW 62.400 usec
DE 6.50 usec
TE 86.0 K
D1 1.00000000 sec
TD0 1
SFO1 400.1324708 MHz
NUC1 1H
P0 4.83 usec
P1 14.50 usec
PLW1 12.00000000 W

F2 - Processing parameters
SI 65536
SF 400.1300097 MHz
WDW EM
SSB 0
LB 0.30 Hz
GB 0
PC 1.00

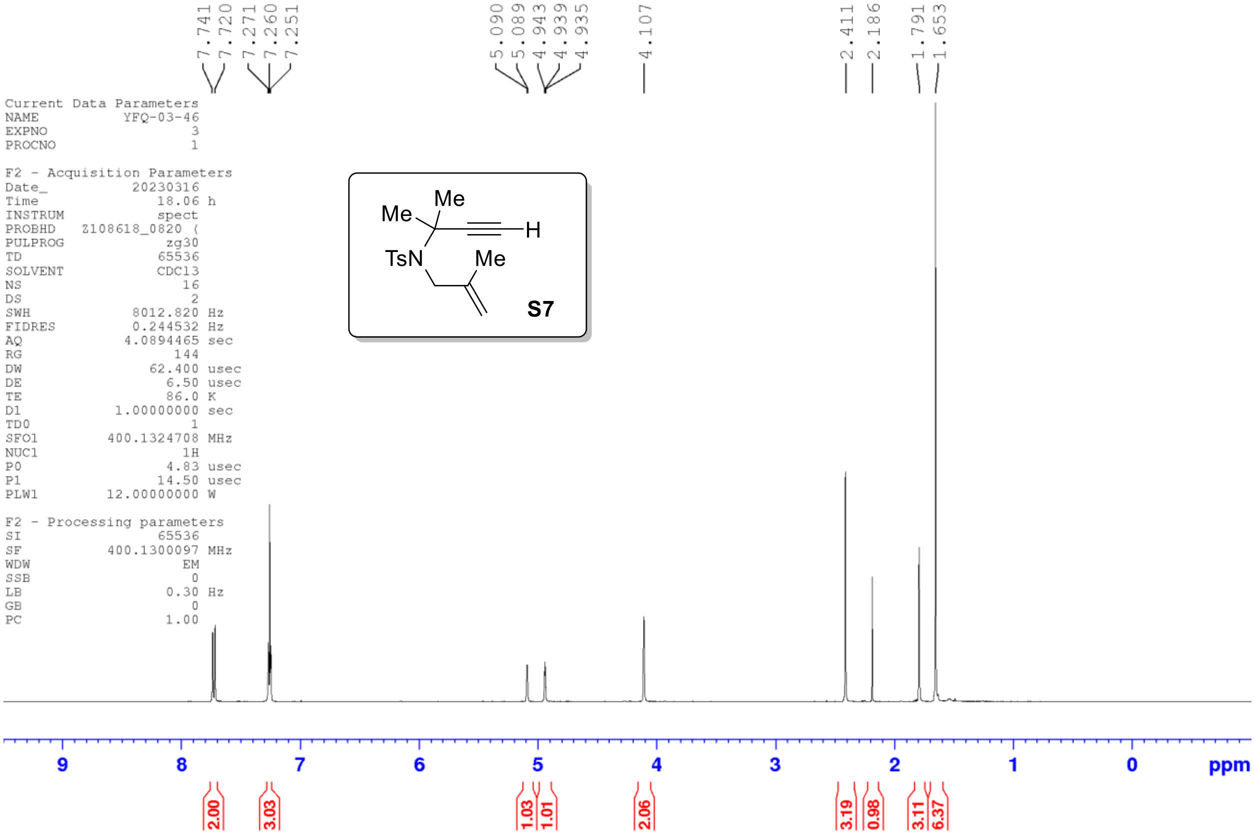

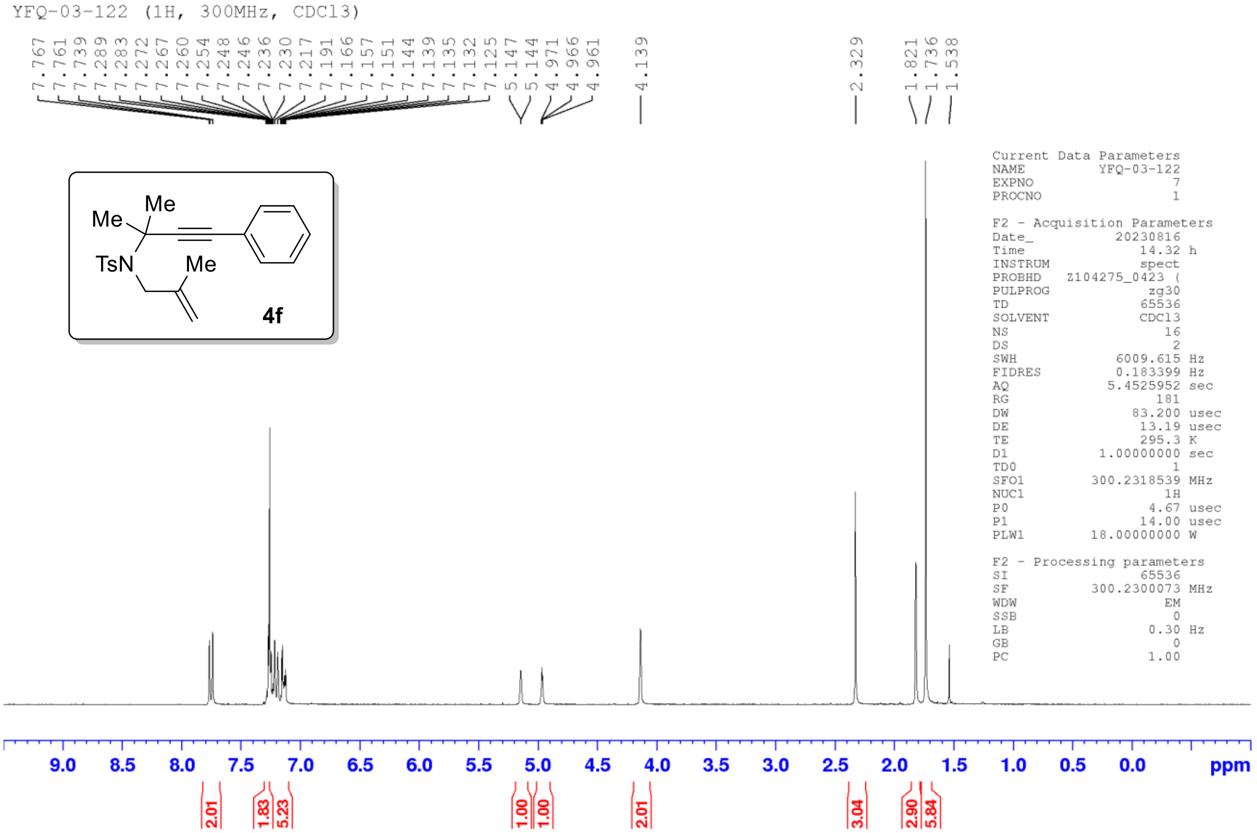
YFQ-03-122 (1H, 300MHz, CDCl3)
7.767
7.761
7.739
7.289
7.283
7.272
7.267
7.260
7.254
7.248
7.246
7.236
7.230
7.217
7.191
7.166
7.157
7.151
7.144
7.139
7.135
7.132
7.125
5.147
5.144
4.971
4.966
4.961
4.139
2.329
1.821
1.736
1.538
Me
Me
TsN
Me
4f
Current Data Parameters
NAME YFQ-03-122
EXPNO 7
PROCNO 1
F2 - Acquisition Parameters
Date_ 20230816
Time 14.32 h
INSTRUM spect
PROBHD Z104275_0423 (
PULPROG zg30
TD 65536
SOLVENT CDCl3
NS 16
DS 2
SWH 6009.615 Hz
FIDRES 0.183399 Hz
AQ 5.4525952 sec
RG 181
DW 83.200 usec
DE 13.19 usec
TE 295.3 K
D1 1.00000000 sec
TD0 1
SFO1 300.2318539 MHz
NUC1 1H
P0 4.67 usec
P1 14.00 usec
PLW1 18.00000000 W
F2 - Processing parameters
SI 65536
SF 300.2300073 MHz
WDW EM
SSB 0
LB 0.30 Hz
GB 0
PC 1.00
9.0
8.5
8.0
7.5
7.0
6.5
6.0
5.5
5.0
4.5
4.0
3.5
3.0
2.5
2.0
1.5
1.0
0.5
0.0
ppm
2.01
1.83
5.23
1.00
1.00
2.01
3.04
2.90
5.84

YFQ-02-85 (1H, 400MHz, CDCl3)

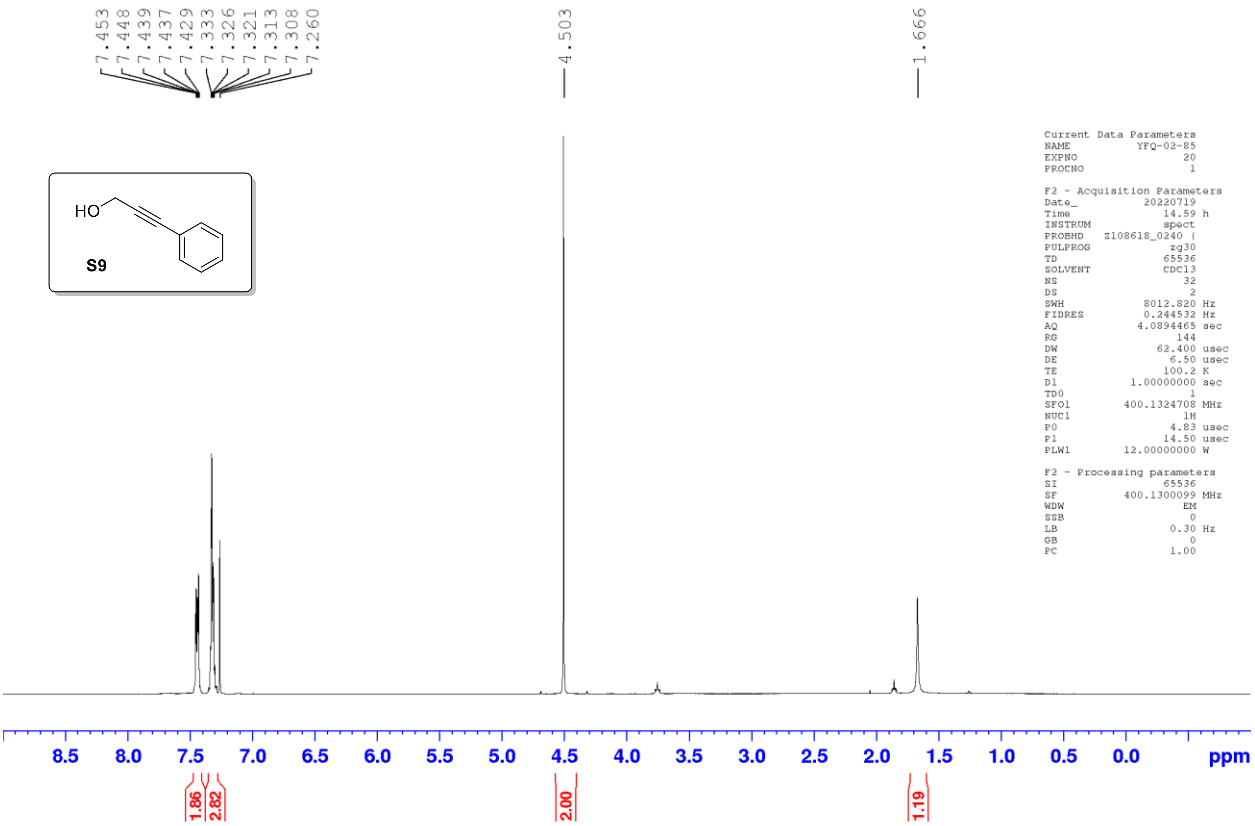

YFQ-01-03 (1H, 400MHz, CDCl3)

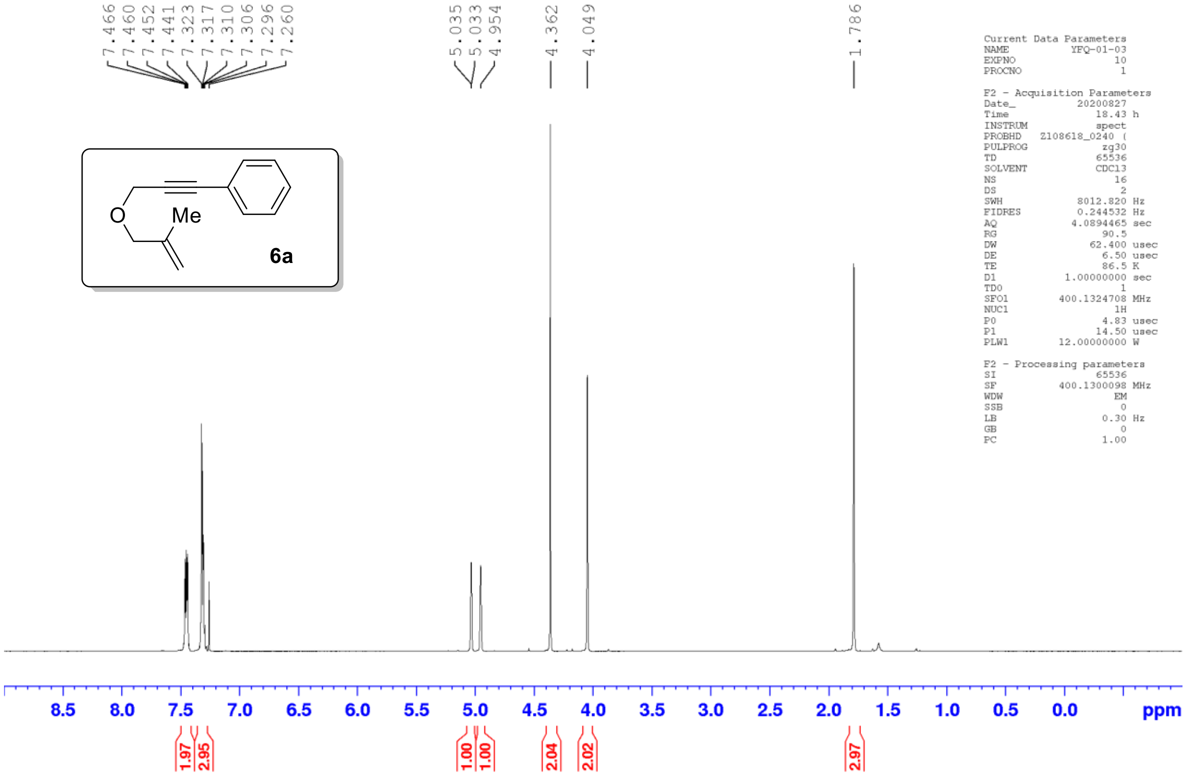

YFQ-01-03 (13C, 100MHz, CDCl3)

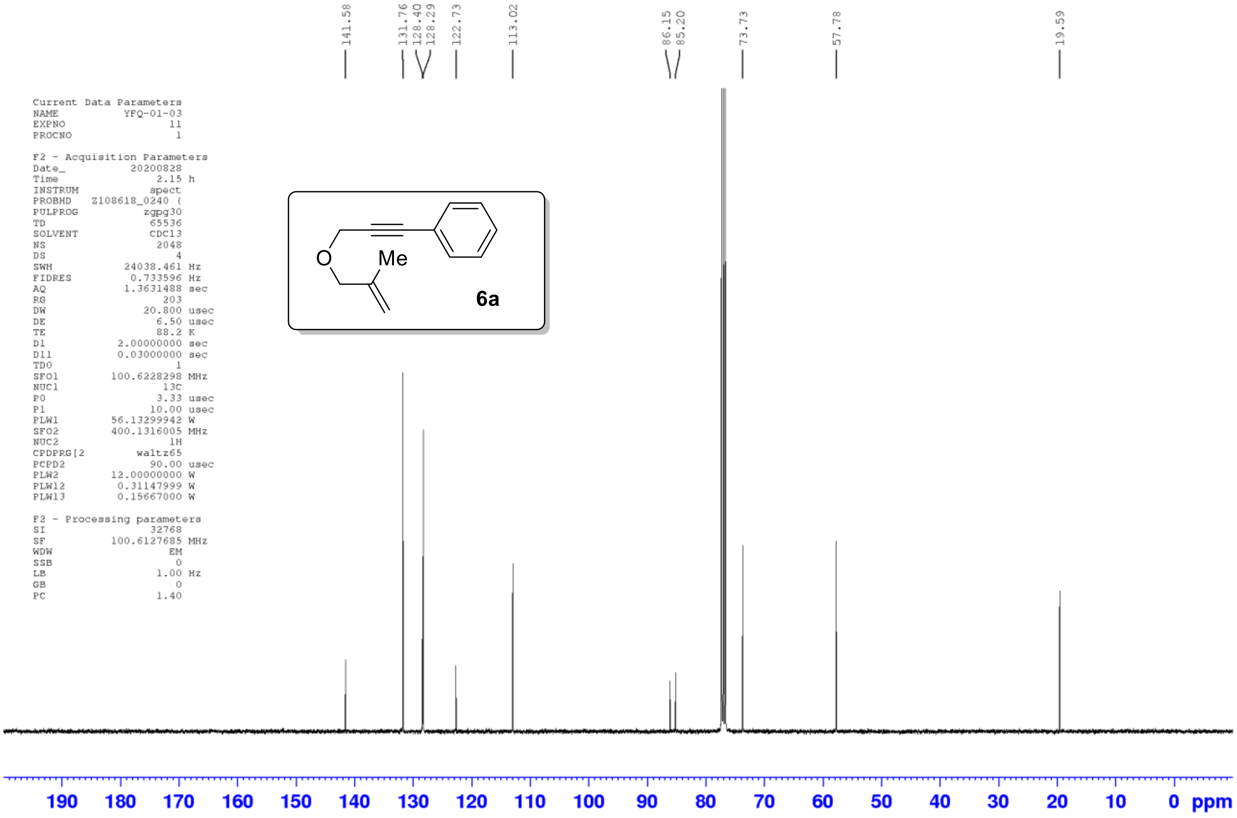

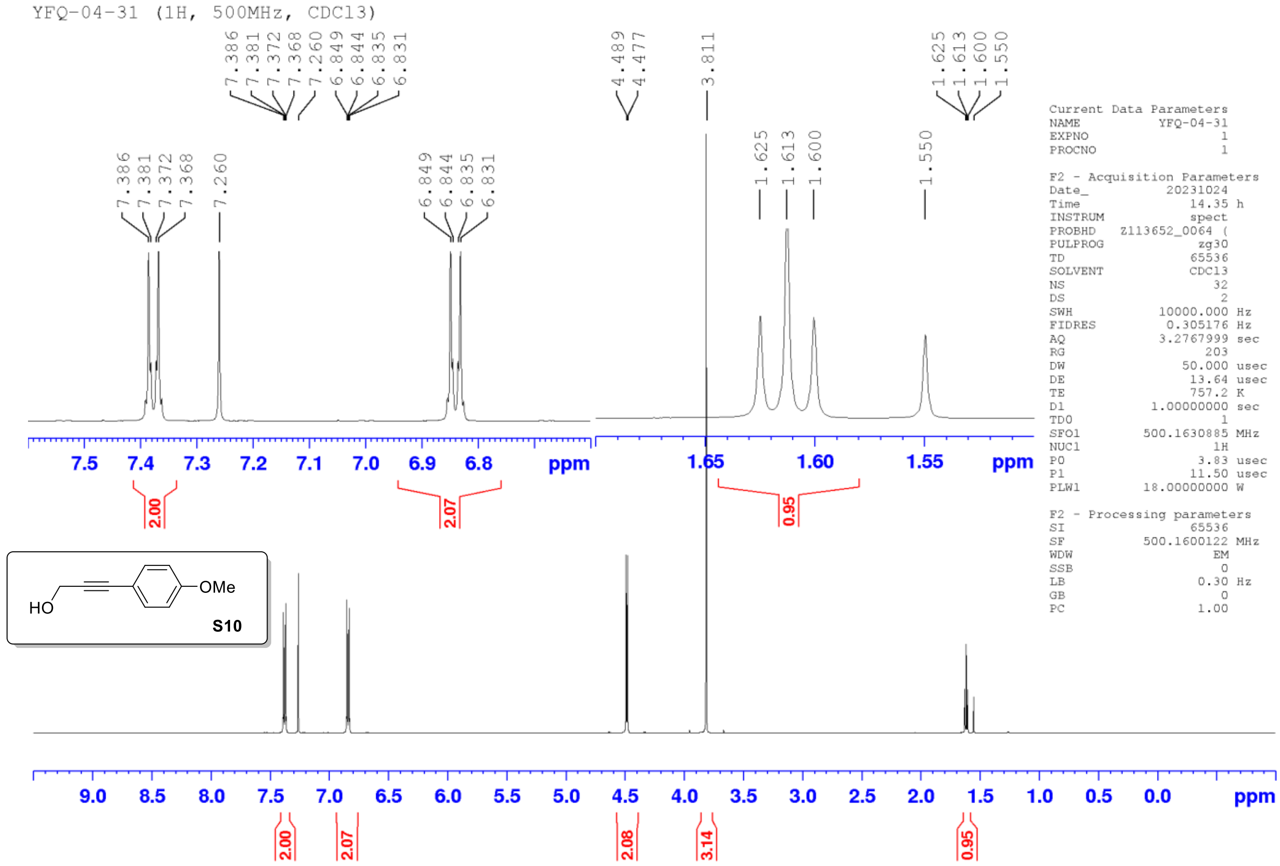
YFQ-04-31 (1H, 500MHz, CDCl3)
7.386
7.381
7.372
7.368
7.260
6.849
6.844
6.835
6.831
4.489
4.477
3.811
1.625
1.613
1.600
1.550
Current Data Parameters
NAME YFQ-04-31
EXPNO 1
PROCNO 1
F2 - Acquisition Parameters
Date_ 20231024
Time 14.35 h
INSTRUM spect
PROBHD Z113652_0064 (
PULPROG zg30
TD 65536
SOLVENT CDCl3
NS 32
DS 2
SWH 10000.000 Hz
FIDRES 0.305176 Hz
AQ 3.2767999 sec
RG 203
DW 50.000 usec
DE 13.64 usec
TE 757.2 K
D1 1.00000000 sec
TD0 1
SFO1 500.1630885 MHz
NUC1 1H
P0 3.83 usec
P1 11.50 usec
PLW1 18.00000000 W
F2 - Processing parameters
SI 65536
SF 500.1600122 MHz
WDW EM
SSB 0
LB 0.30 Hz
GB 0
PC 1.00
HO
OMe
S10
2.00
2.07
0.95
2.08
3.14
ppm

YFQ-04-32 (1H, 400MHz, CDCl3)

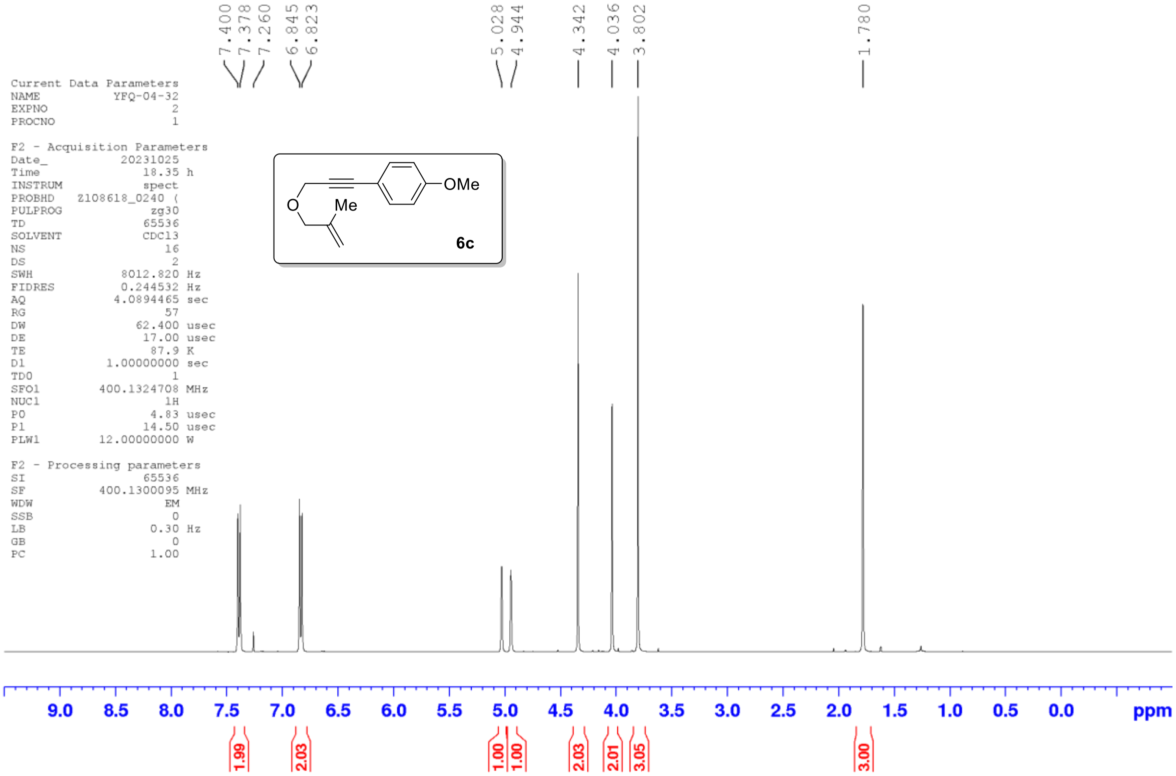


```
Current Data Parameters
NAME          YFQ-04-32
EXPNO                 2
PROCNO                1

F2 - Acquisition Parameters
Date_          20231025
Time              18.35 h
INSTRUM           spect
PROBHD   Z108618_0240 (
PULPROG            zg30
TD                65536
SOLVENT           CDCl3
NS                   16
DS                    2
SWH            8012.820 Hz
FIDRES         0.244532 Hz
AQ            4.0894465 sec
RG                   57
DW               62.400 usec
DE                17.00 usec
TE                 87.9 K
D1           1.00000000 sec
TD0                   1
SFO1        400.1324708 MHz
NUC1                 1H
P0                 4.83 usec
P1                14.50 usec
PLW1        12.00000000 W

F2 - Processing parameters
SI                65536
SF          400.1300095 MHz
WDW                  EM
SSB                   0
LB                 0.30 Hz
GB                    0
PC                 1.00
```

YFQ-04-32 (13C,100MHz, CDCl3)

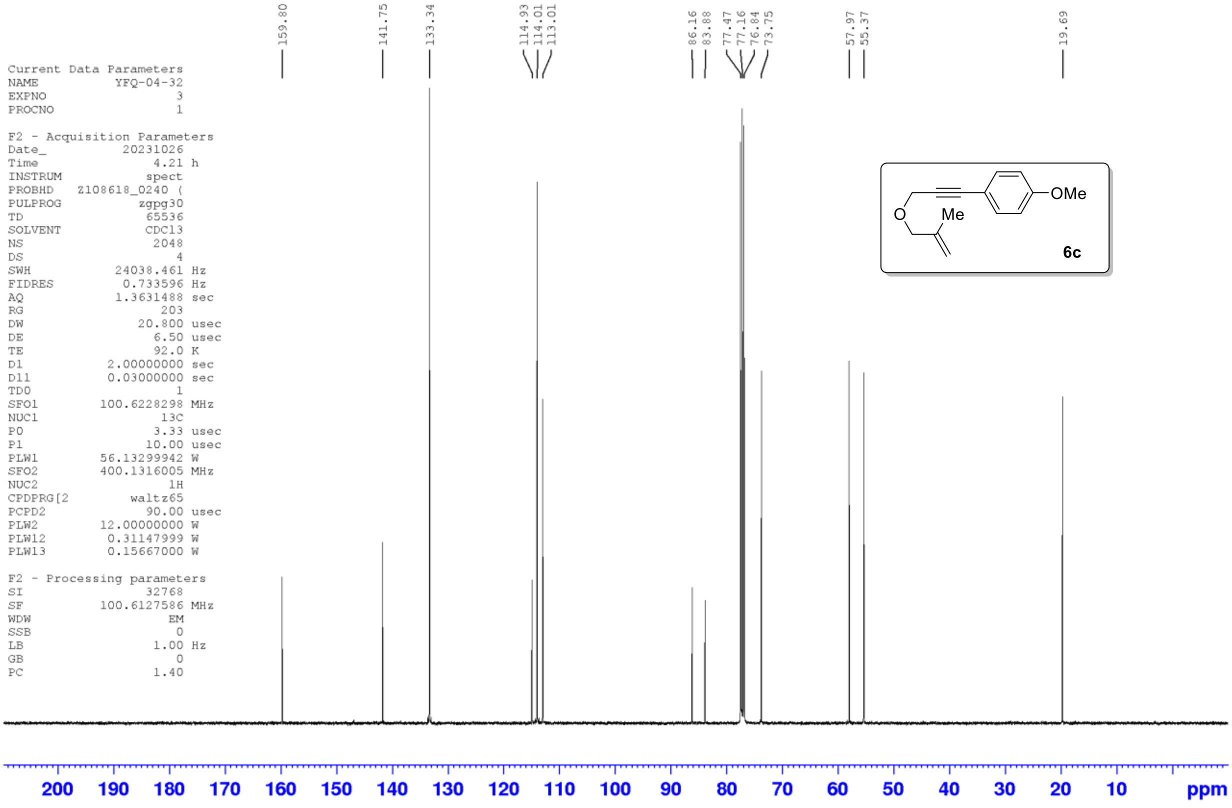


```
Current Data Parameters
NAME          YFQ-04-32
EXPNO                 3
PROCNO                1

F2 - Acquisition Parameters
Date_          20231026
Time               4.21 h
INSTRUM           spect
PROBHD   Z108618_0240 (
PULPROG          zgpg30
TD                65536
SOLVENT           CDCl3
NS                 2048
DS                    4
SWH           24038.461 Hz
FIDRES         0.733596 Hz
AQ            1.3631488 sec
RG                  203
DW               20.800 usec
DE                 6.50 usec
TE                 92.0 K
D1           2.00000000 sec
D11          0.03000000 sec
TD0                   1
SFO1        100.6228298 MHz
NUC1                13C
P0                 3.33 usec
P1                10.00 usec
PLW1        56.13299942 W
SFO2        400.1316005 MHz
NUC2                 1H
CPDPRG[2        waltz65
PCPD2             90.00 usec
PLW2        12.00000000 W
PLW12        0.31147999 W
PLW13        0.15667000 W

F2 - Processing parameters
SI                32768
SF          100.6127586 MHz
WDW                  EM
SSB                   0
LB                 1.00 Hz
GB                    0
PC                 1.40
```

YFQ-01-76 (1H, 400MHz, CDCl3)

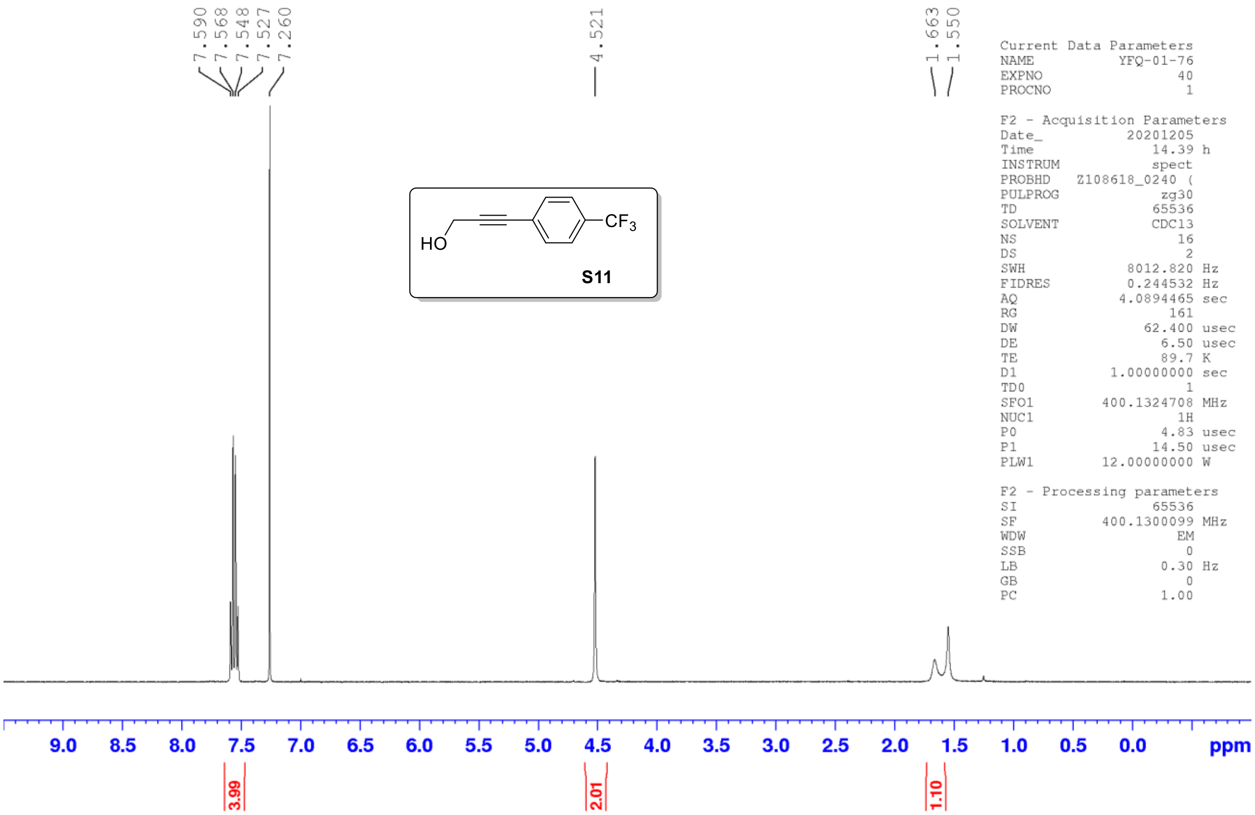

YFQ-01-77 (1H, 400MHz, CDCl3)

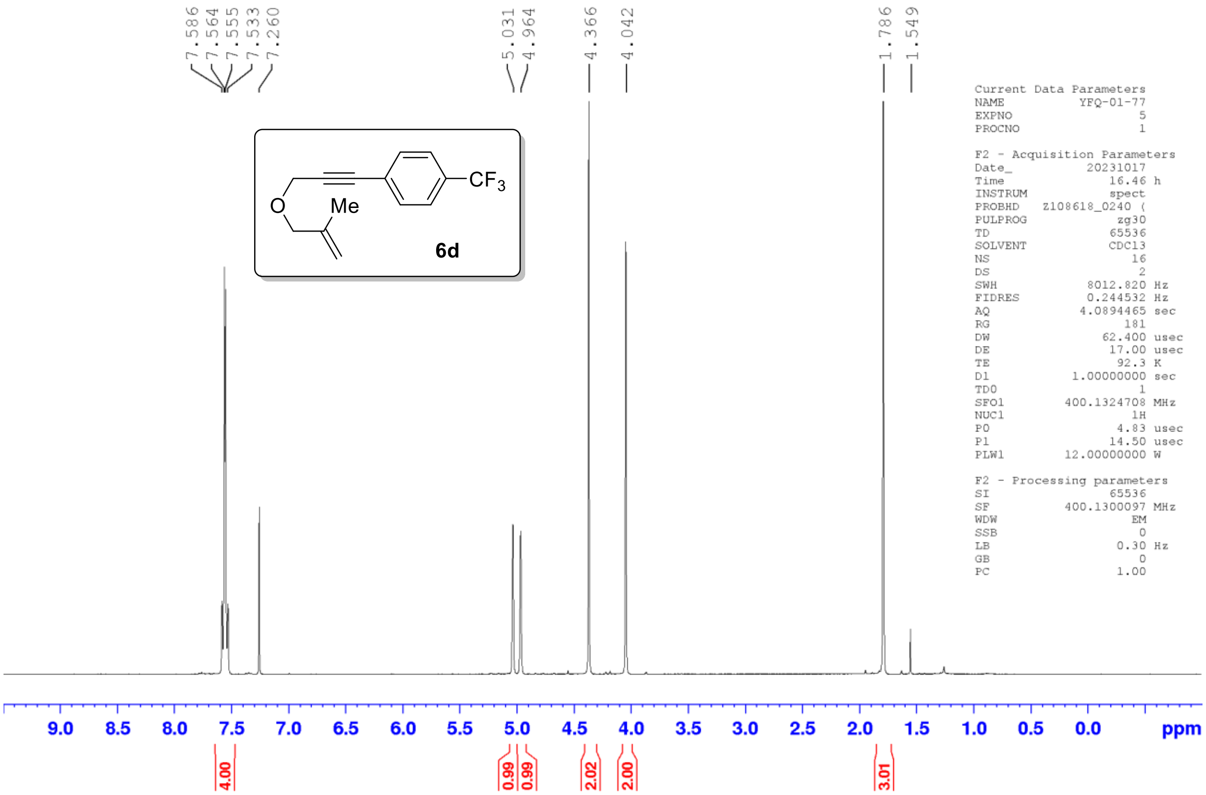

YFQ-01-77 (13C, 100MHz, CDCl3)

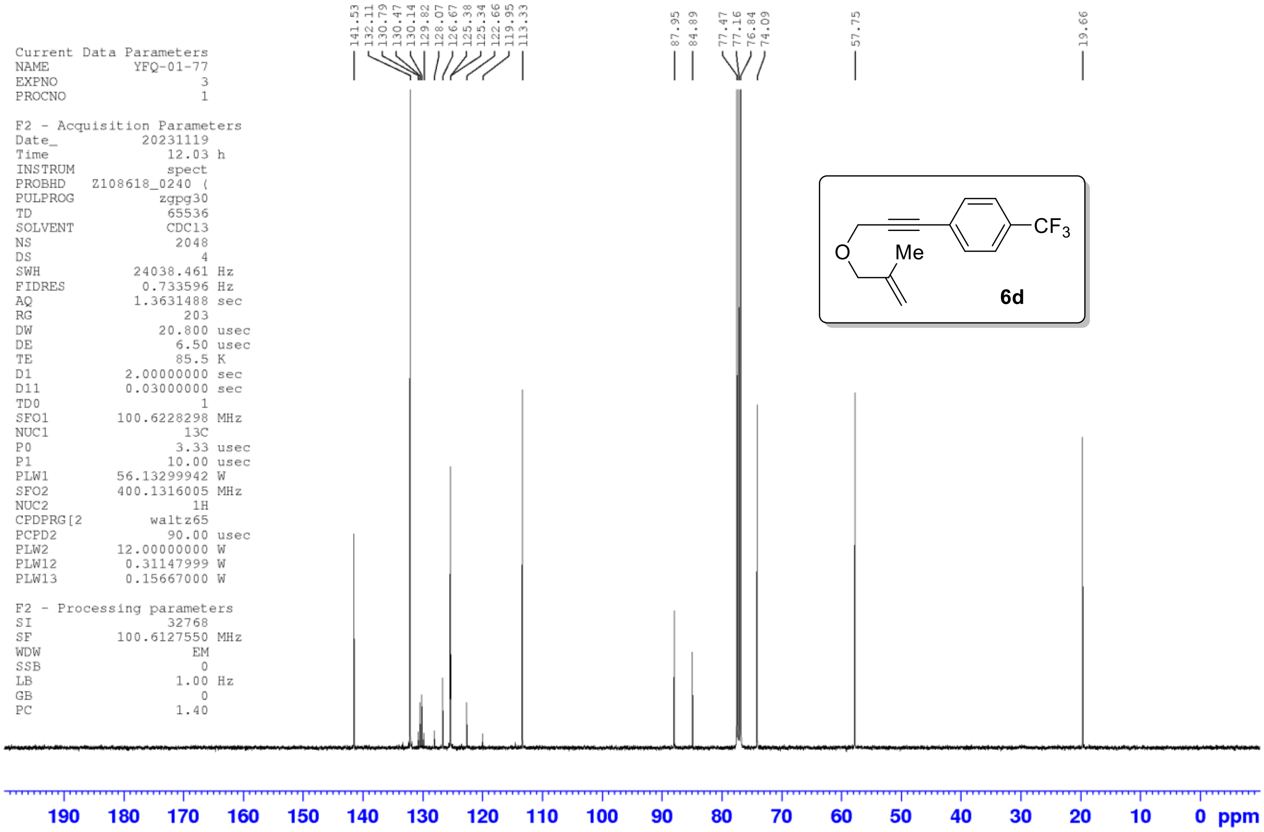

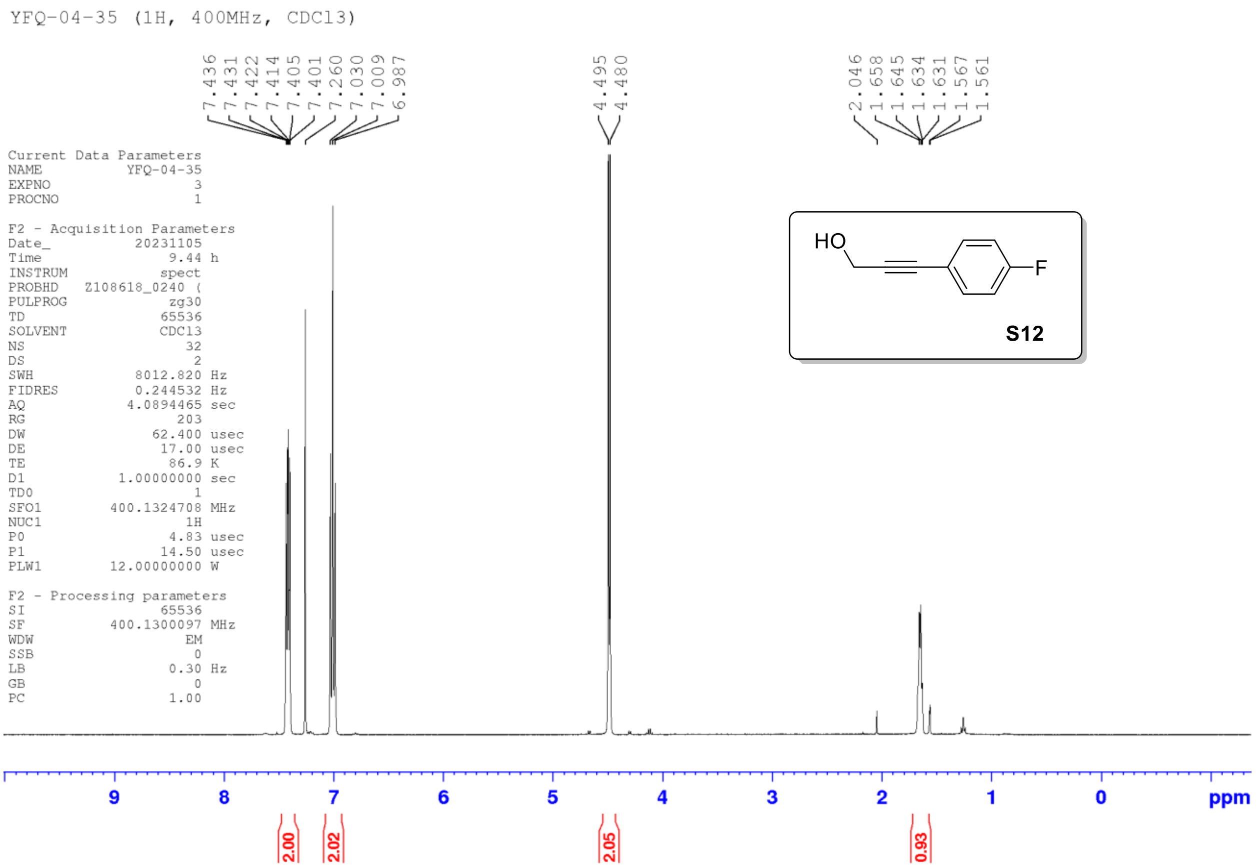
YFQ-04-35 (1H, 400MHz, CDCl3)
7.436
7.431
7.422
7.414
7.405
7.401
7.260
7.030
7.009
6.987
4.495
4.480
2.046
1.658
1.645
1.634
1.631
1.567
1.561
Current Data Parameters
NAME YFQ-04-35
EXPNO 3
PROCNO 1
F2 - Acquisition Parameters
Date_ 20231105
Time 9.44 h
INSTRUM spect
PROBHD Z108618_0240 (
PULPROG zg30
TD 65536
SOLVENT CDCl3
NS 32
DS 2
SWH 8012.820 Hz
FIDRES 0.244532 Hz
AQ 4.0894465 sec
RG 203
DW 62.400 usec
DE 17.00 usec
TE 86.9 K
D1 1.00000000 sec
TD0 1
SFO1 400.1324708 MHz
NUC1 1H
P0 4.83 usec
P1 14.50 usec
PLW1 12.00000000 W
F2 - Processing parameters
SI 65536
SF 400.1300097 MHz
WDW EM
SSB 0
LB 0.30 Hz
GB 0
PC 1.00
HO
F
S12
9
8
7
6
5
4
3
2
1
0
ppm
2.00
2.02
2.05
0.93

YFQ-04-36 (1H, 400MHz, CDCl3)

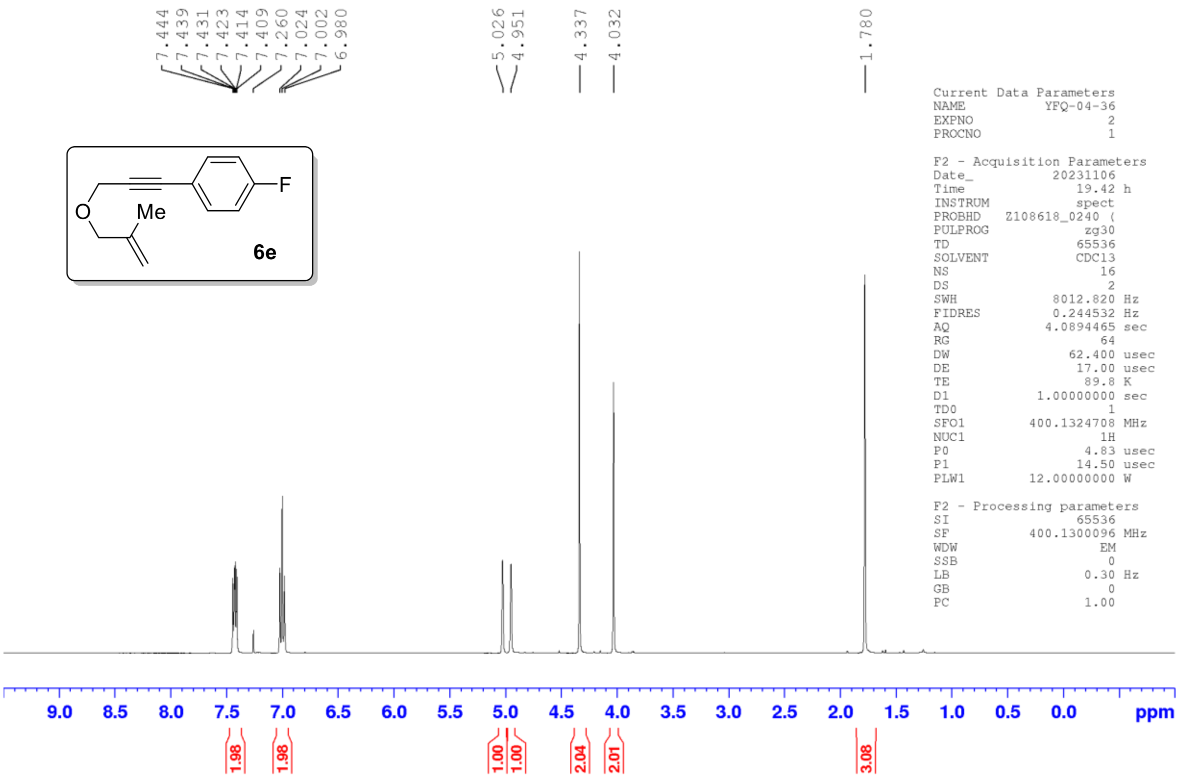

YFQ-04-36 (13C, 100MHz, CDCl3)

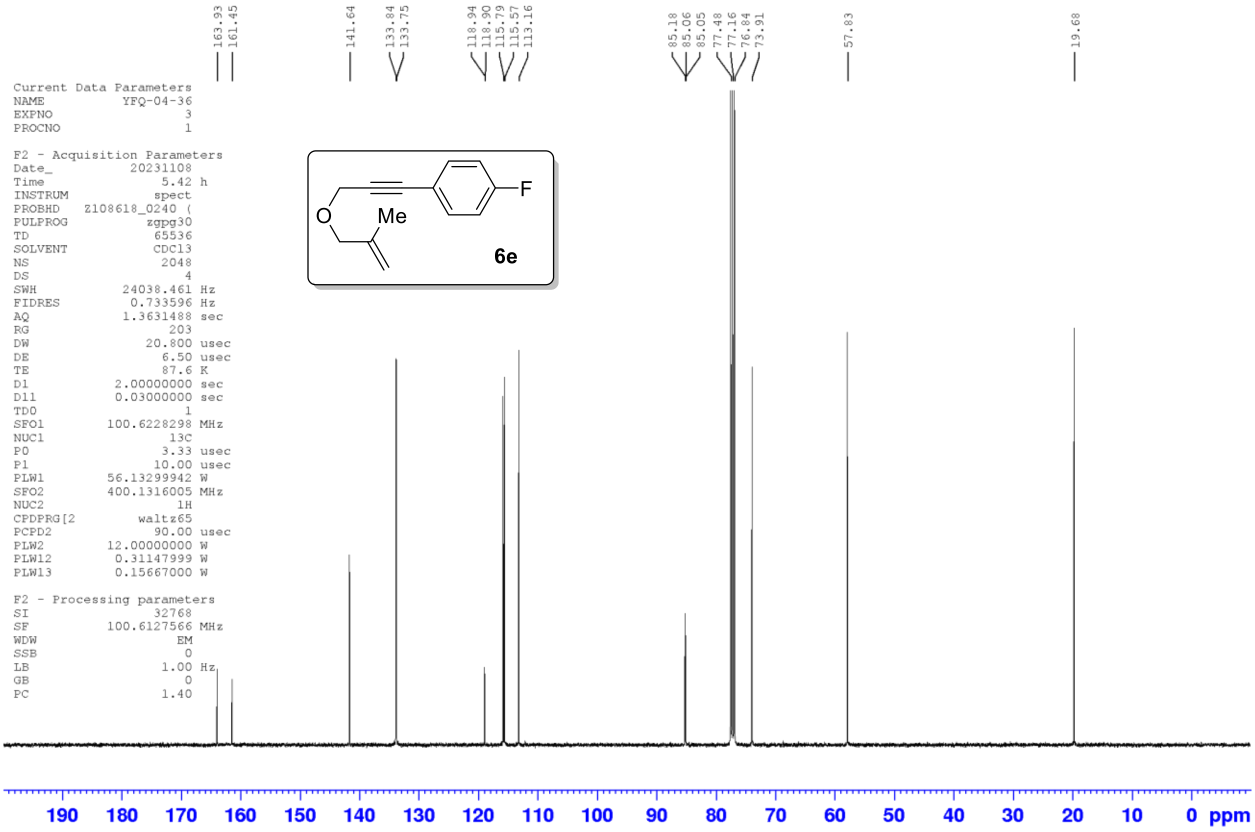

YFQ-03-87 (1H, 500MHz, CDCl3)

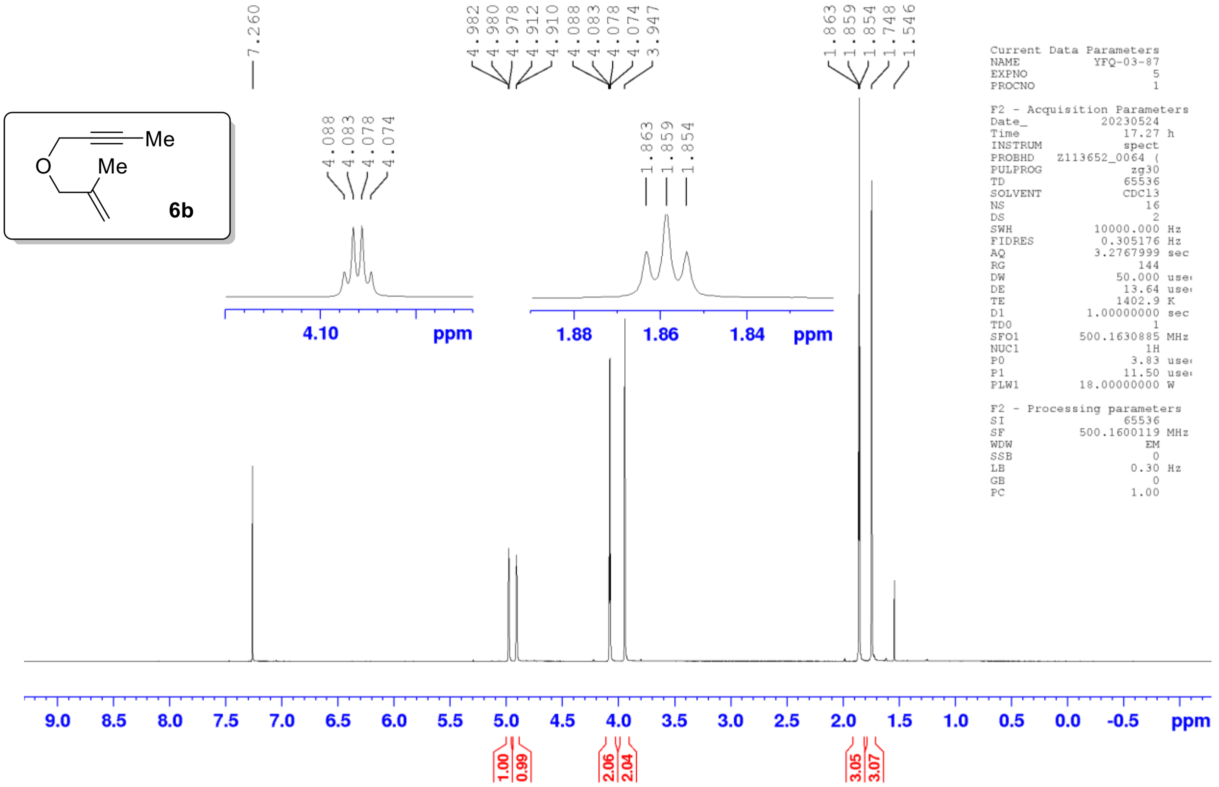

YFQ-03-87 (13C, 125MHz, CDCl3)

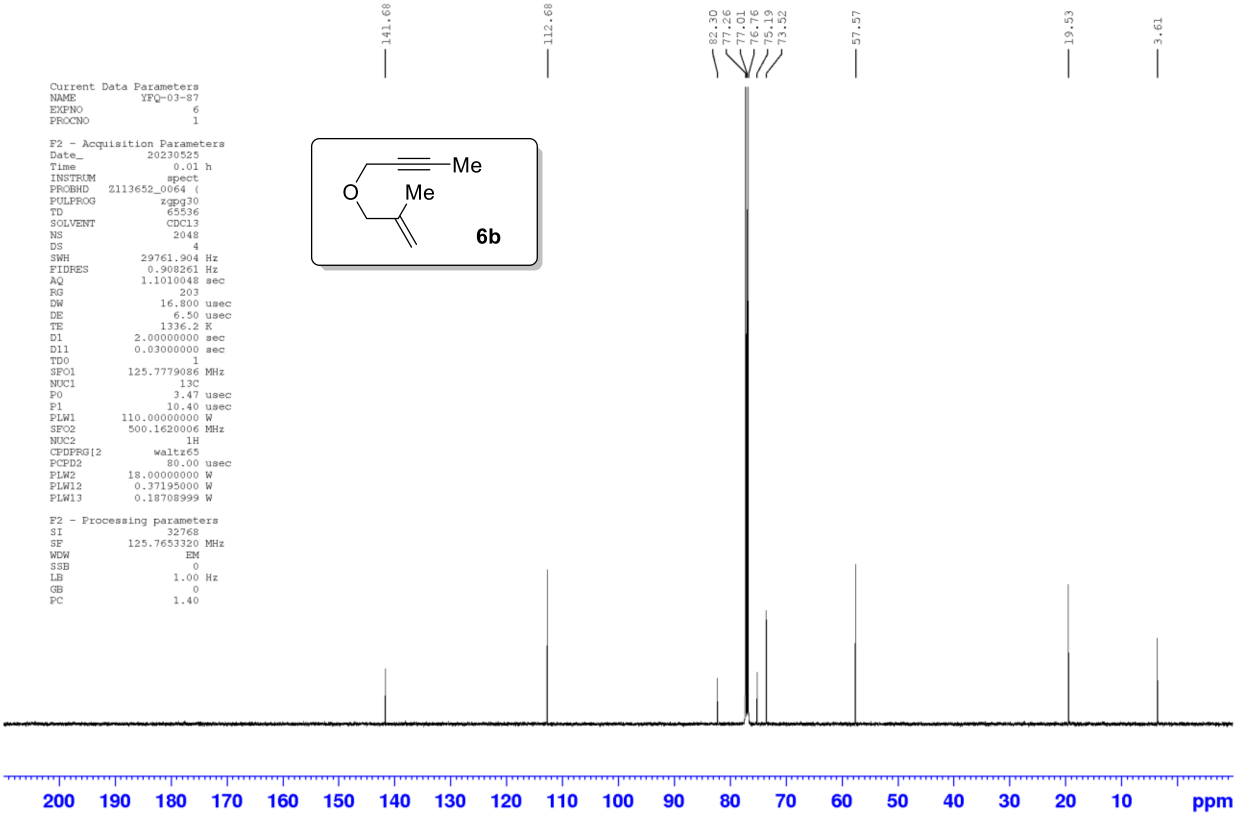

YFQ-03-97 (1H, 400MHz, CDCl3)

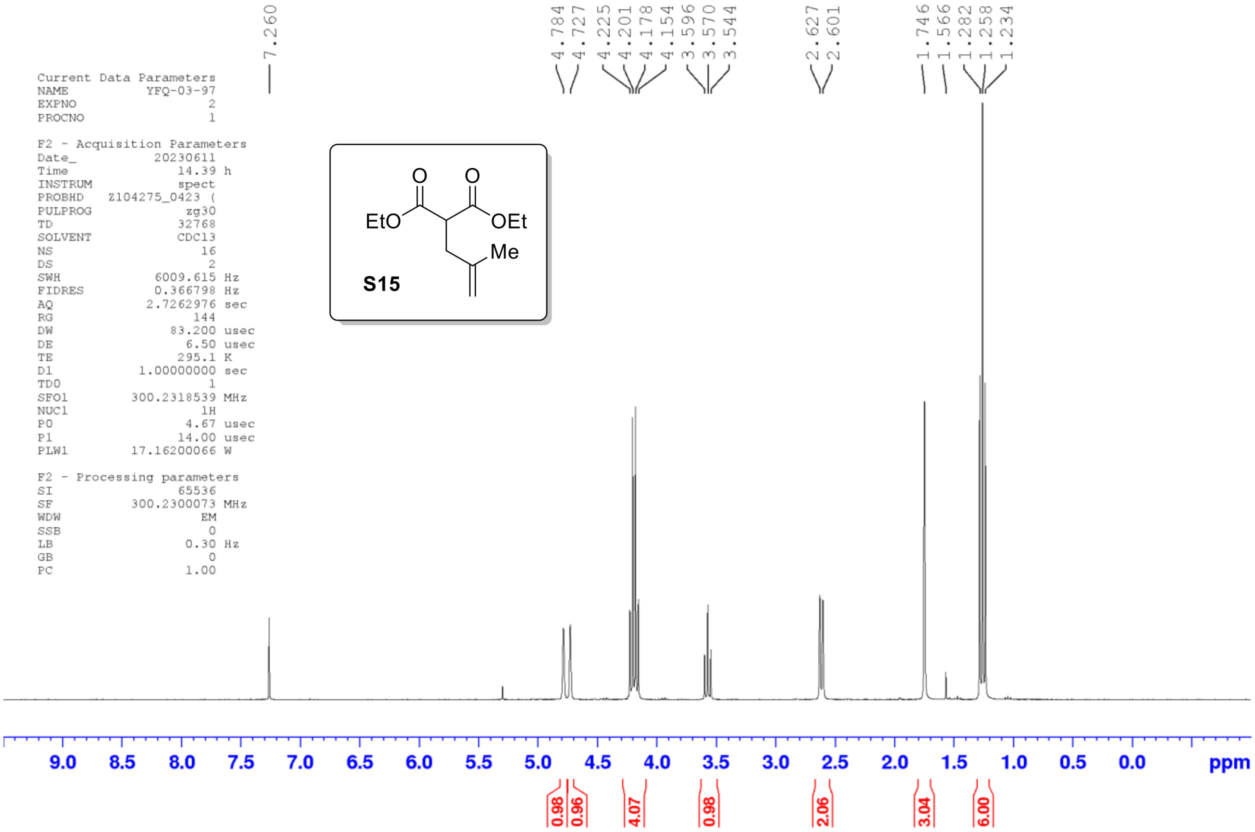

YFQ-03-101 (1H, 400MHz, CDCl3)

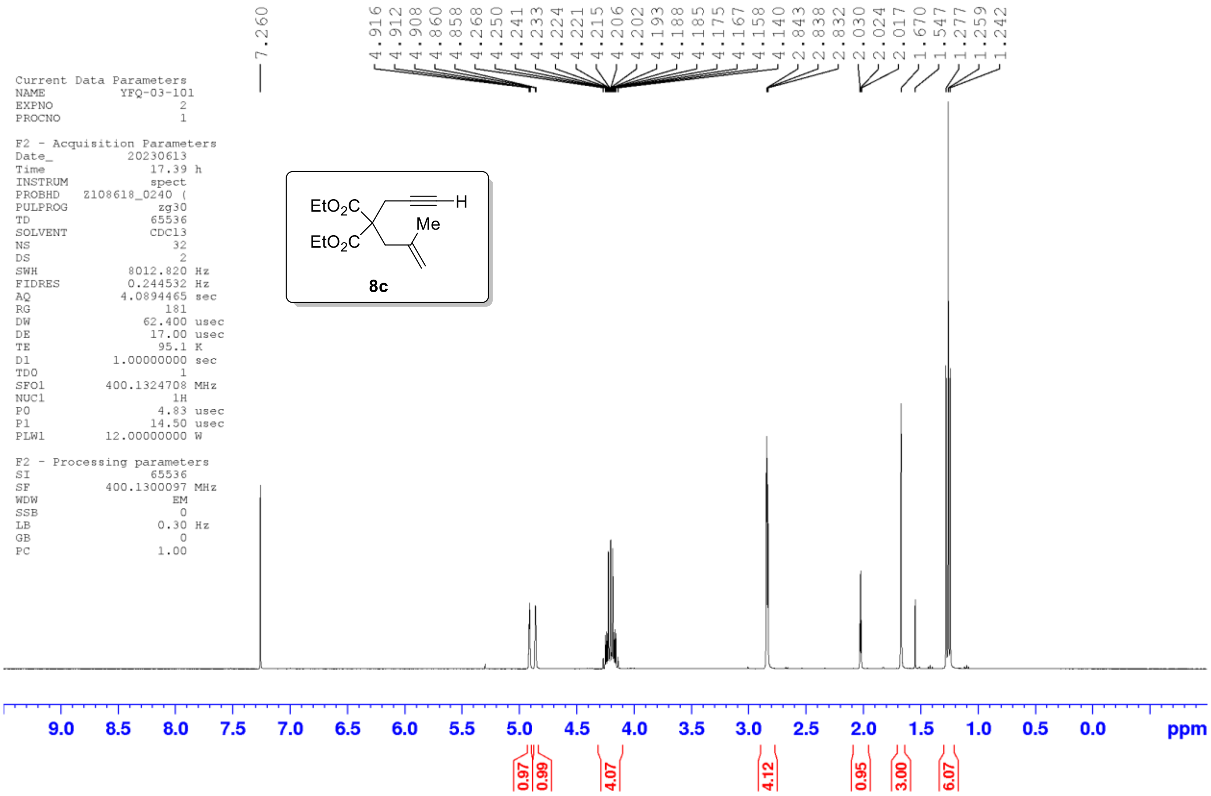

YFQ-03-101 (13C, 100MHz, CDCl3)

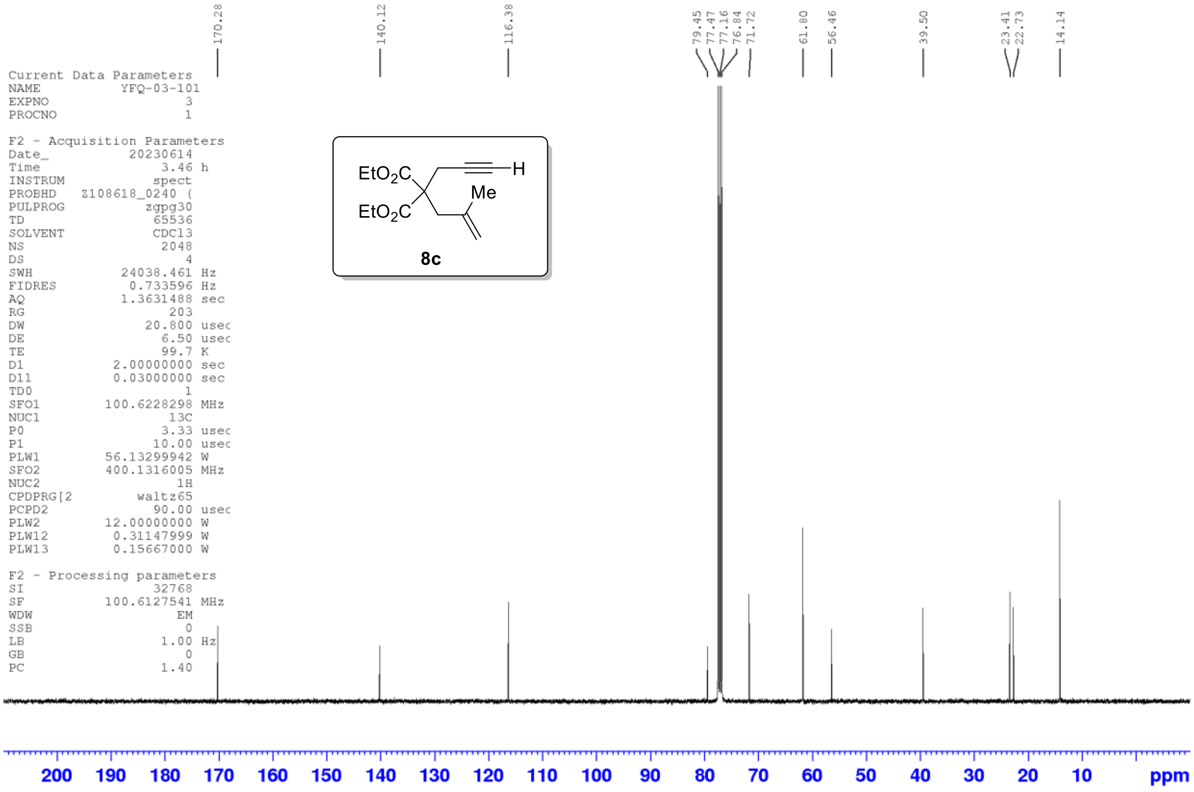

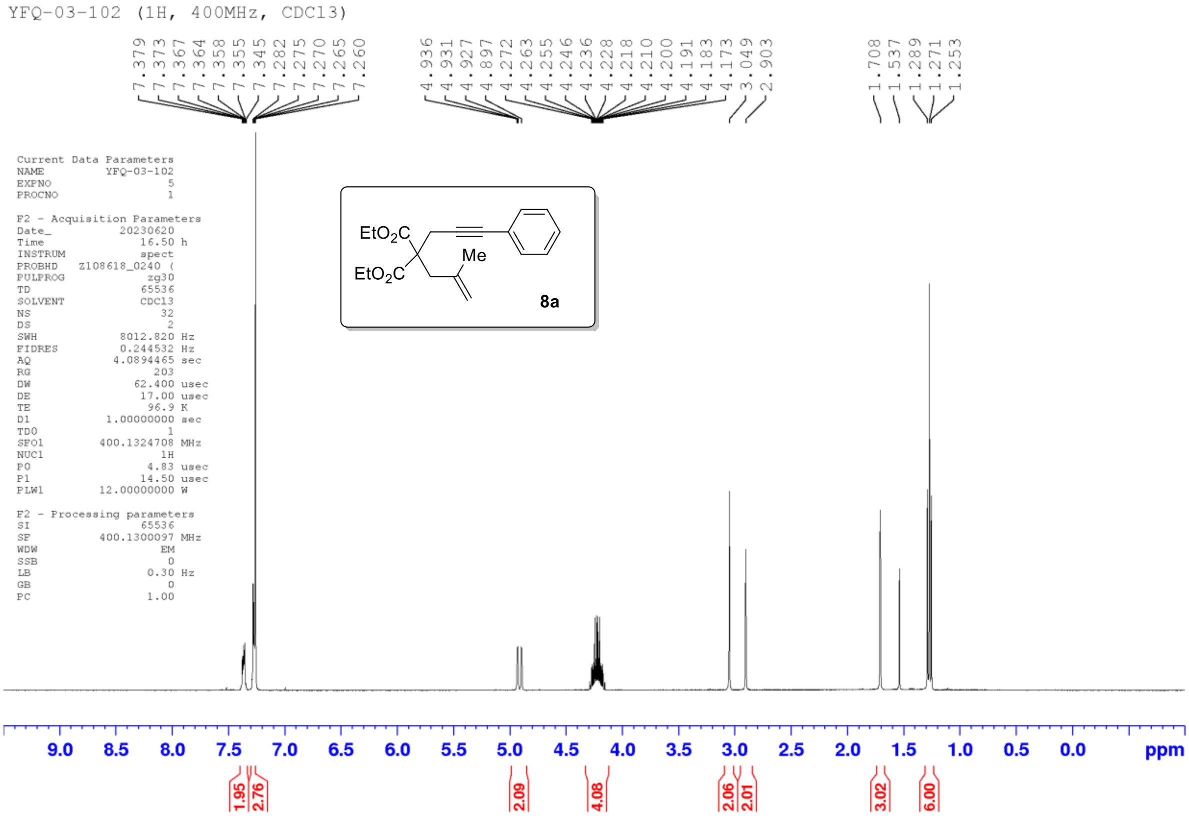
YFQ-03-102 (1H, 400MHz, CDCl3)
7.379 7.373 7.367 7.364 7.358 7.355 7.345 7.282 7.275 7.270 7.265 7.260
4.936 4.931 4.927 4.897 4.272 4.263 4.255 4.246 4.236 4.228 4.218 4.210 4.200 4.191 4.183 4.173 3.049 2.903
1.708 1.537 1.289 1.271 1.253
Current Data Parameters
NAME YFQ-03-102
EXPNO 5
PROCNO 1
F2 - Acquisition Parameters
Date_ 20230620
Time 16.50 h
INSTRUM spect
PROBHD Z108618_0240 (
PULPROG zg30
TD 65536
SOLVENT CDCl3
NS 32
DS 2
SWH 8012.820 Hz
FIDRES 0.244532 Hz
AQ 4.0894465 sec
RG 203
DW 62.400 usec
DE 17.00 usec
TE 96.9 K
D1 1.00000000 sec
TD0 1
SFO1 400.1324708 MHz
NUC1 1H
P0 4.83 usec
P1 14.50 usec
PLW1 12.00000000 W
F2 - Processing parameters
SI 65536
SF 400.1300097 MHz
WDW EM
SSB 0
LB 0.30 Hz
GB 0
PC 1.00
EtO2C
EtO2C
Me
8a
9.0 8.5 8.0 7.5 7.0 6.5 6.0 5.5 5.0 4.5 4.0 3.5 3.0 2.5 2.0 1.5 1.0 0.5 0.0 ppm
1.95 2.76 2.09 4.08 2.06 2.01 3.02 6.00

YFQ-03-108 (1H, 400MHz, CDCl3)

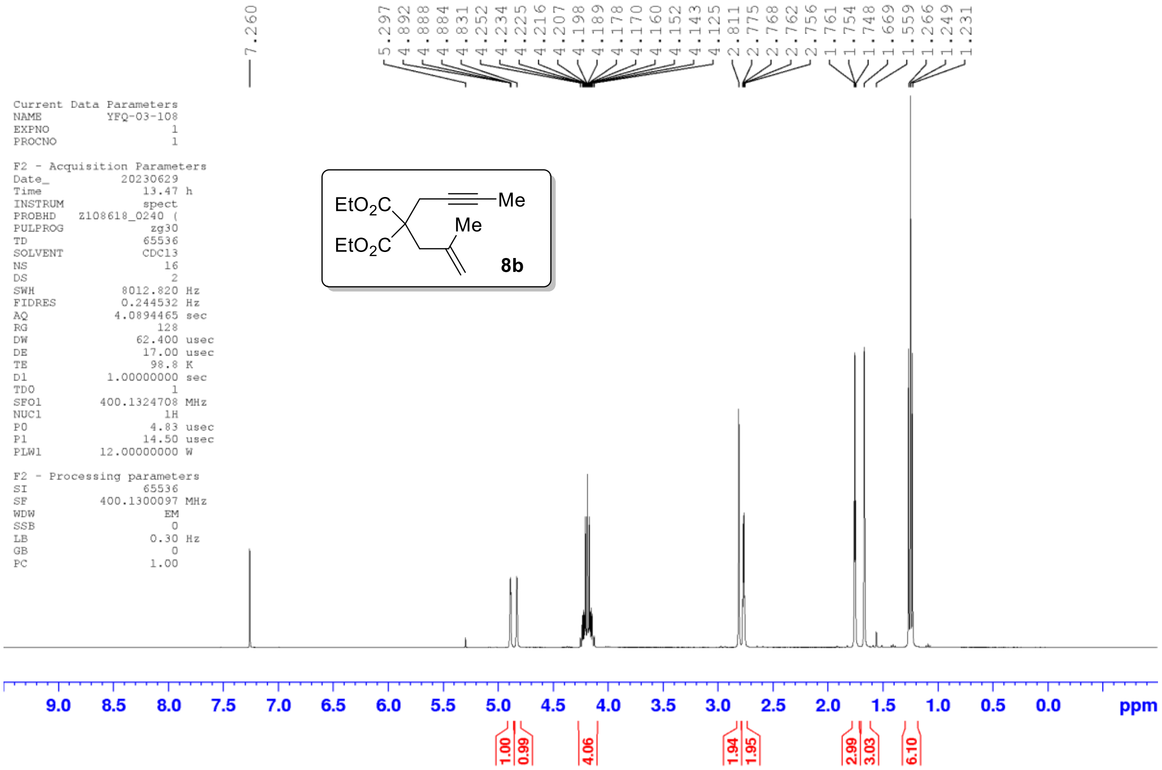

YFQ-03-108 (13C, 100MHz, CDCl3)

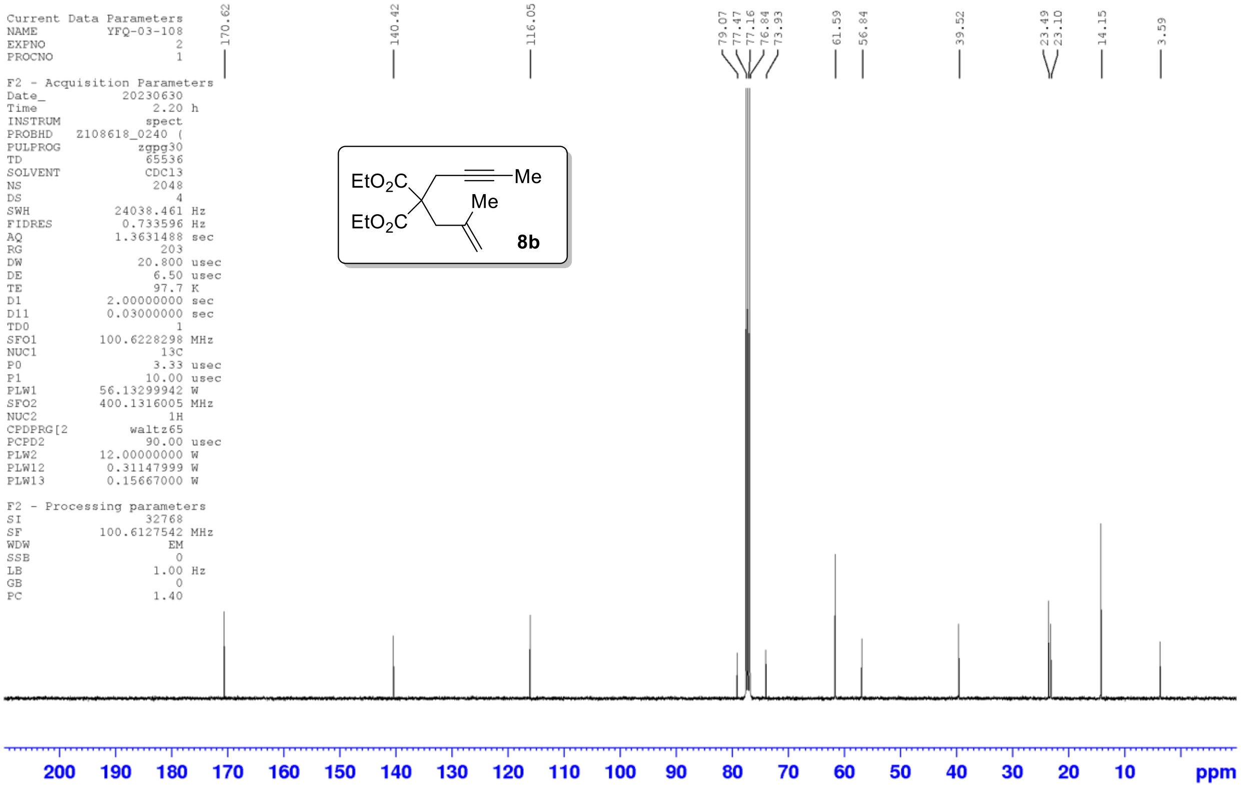

YFQ-03-119 (1H, 400MHz, CDCl3)

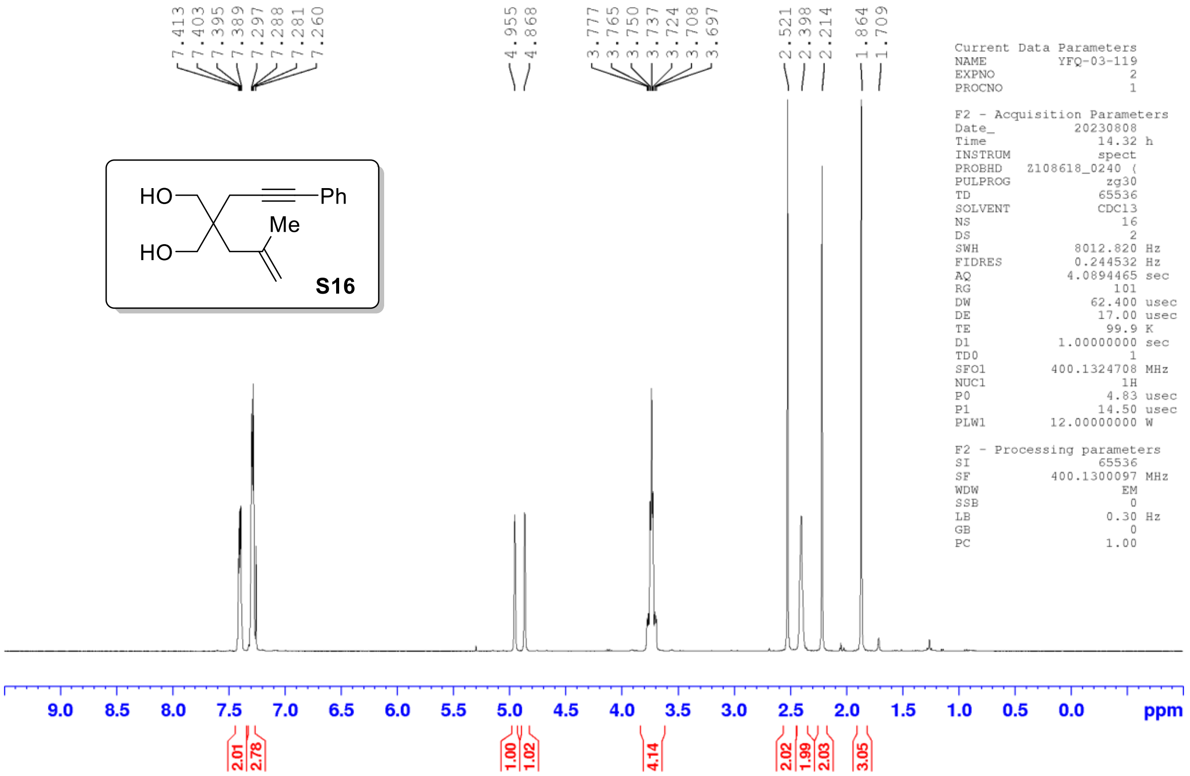

YFQ-03-119 (13C, 100MHz, CDCl3)

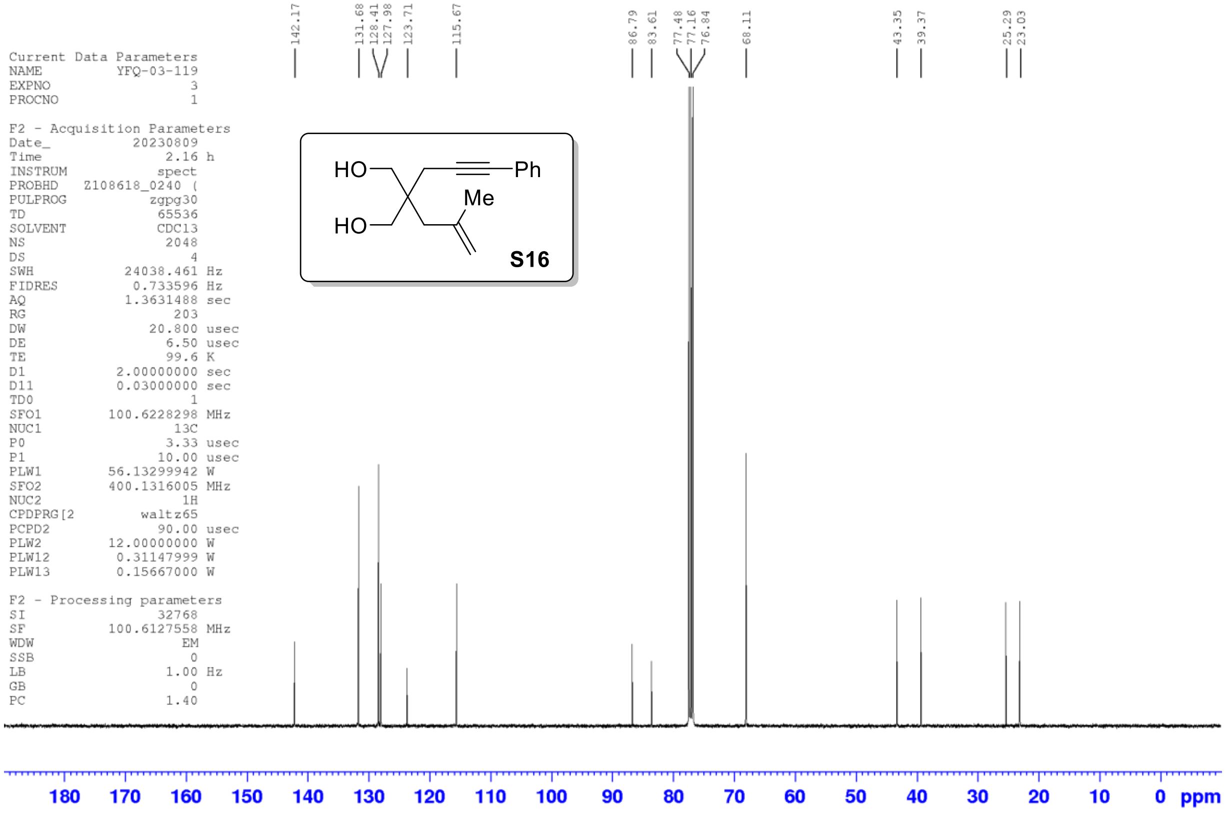

YFQ-03-120 (1H, 400MHz, CDCl3)

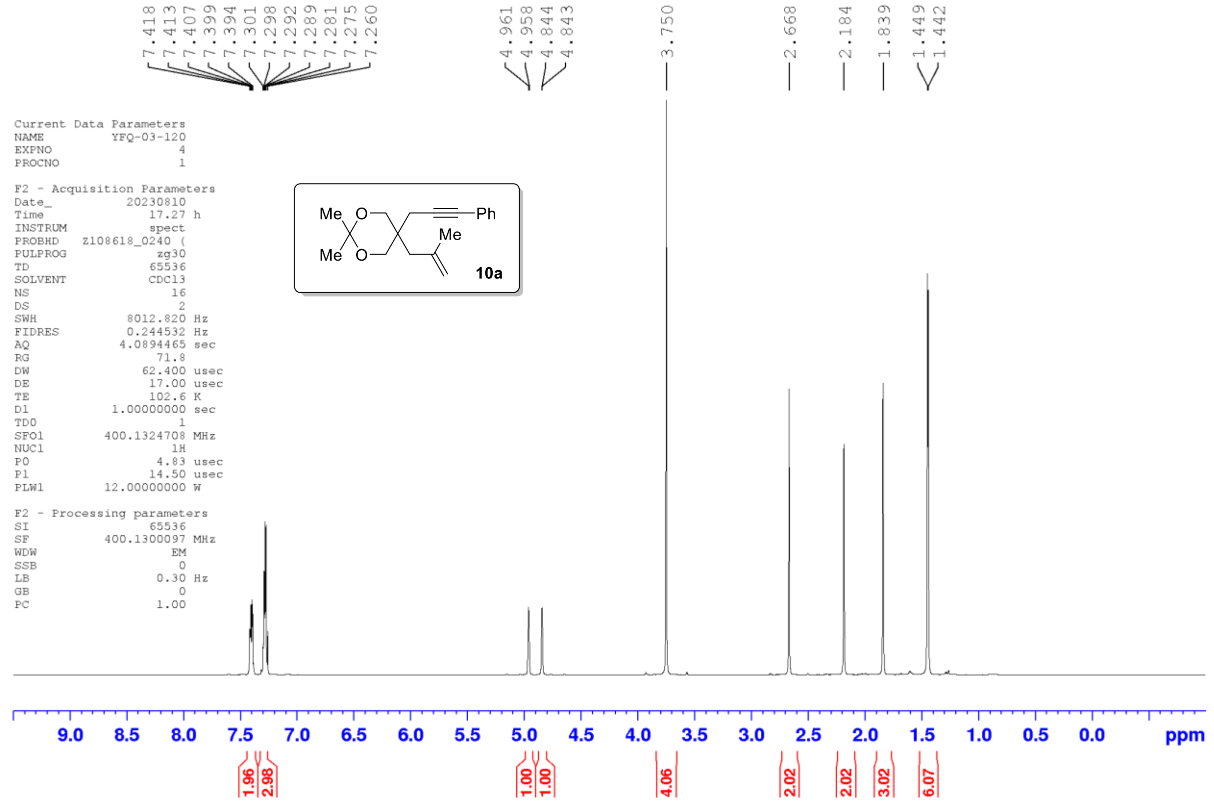

YFQ-03-120 (13C, 100MHz, CDCl3)

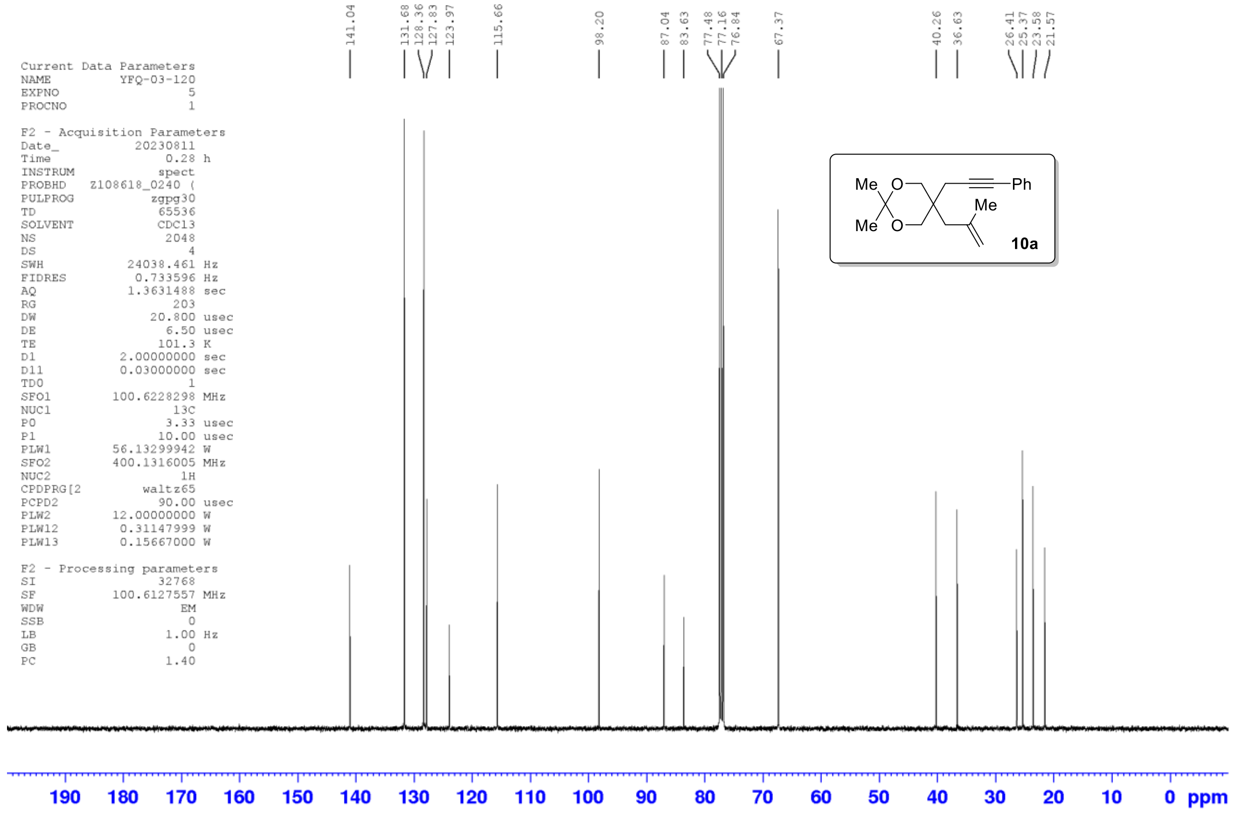

YFQ-04-02 (1H, 400MHz, CDCl3)

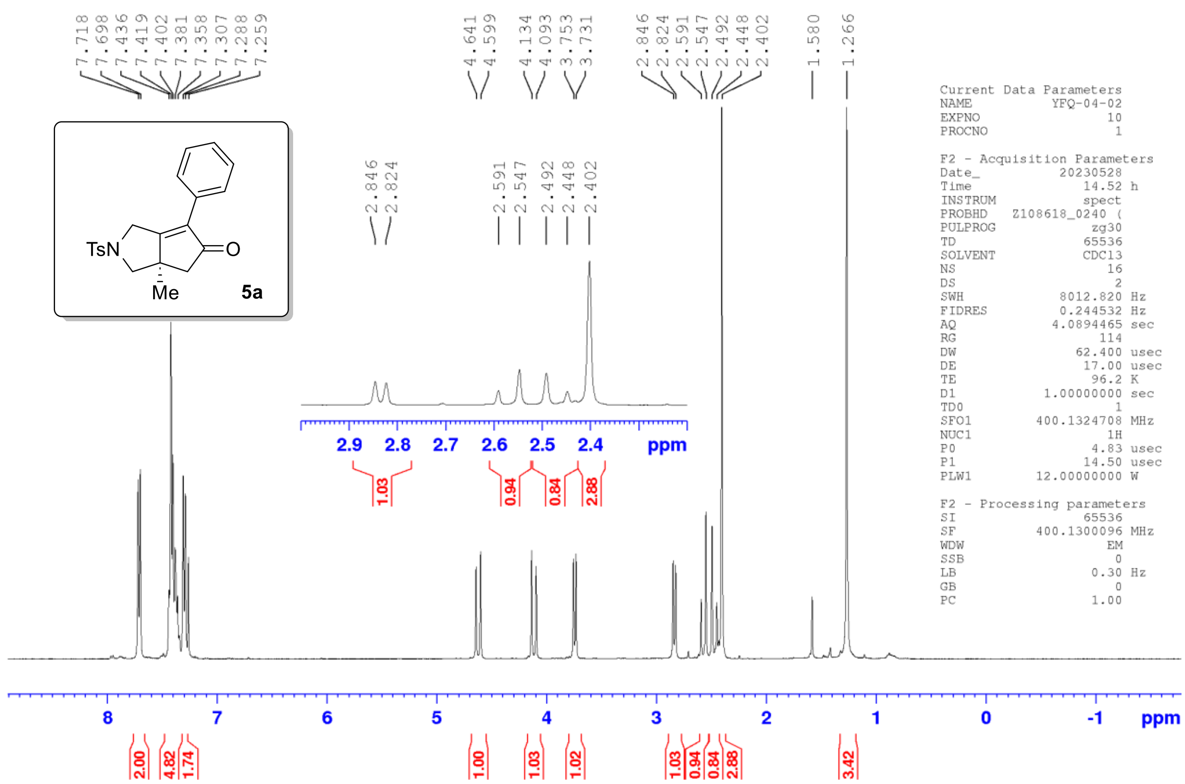

YFQ-04-02 (13C, 100MHz, CDCl3)

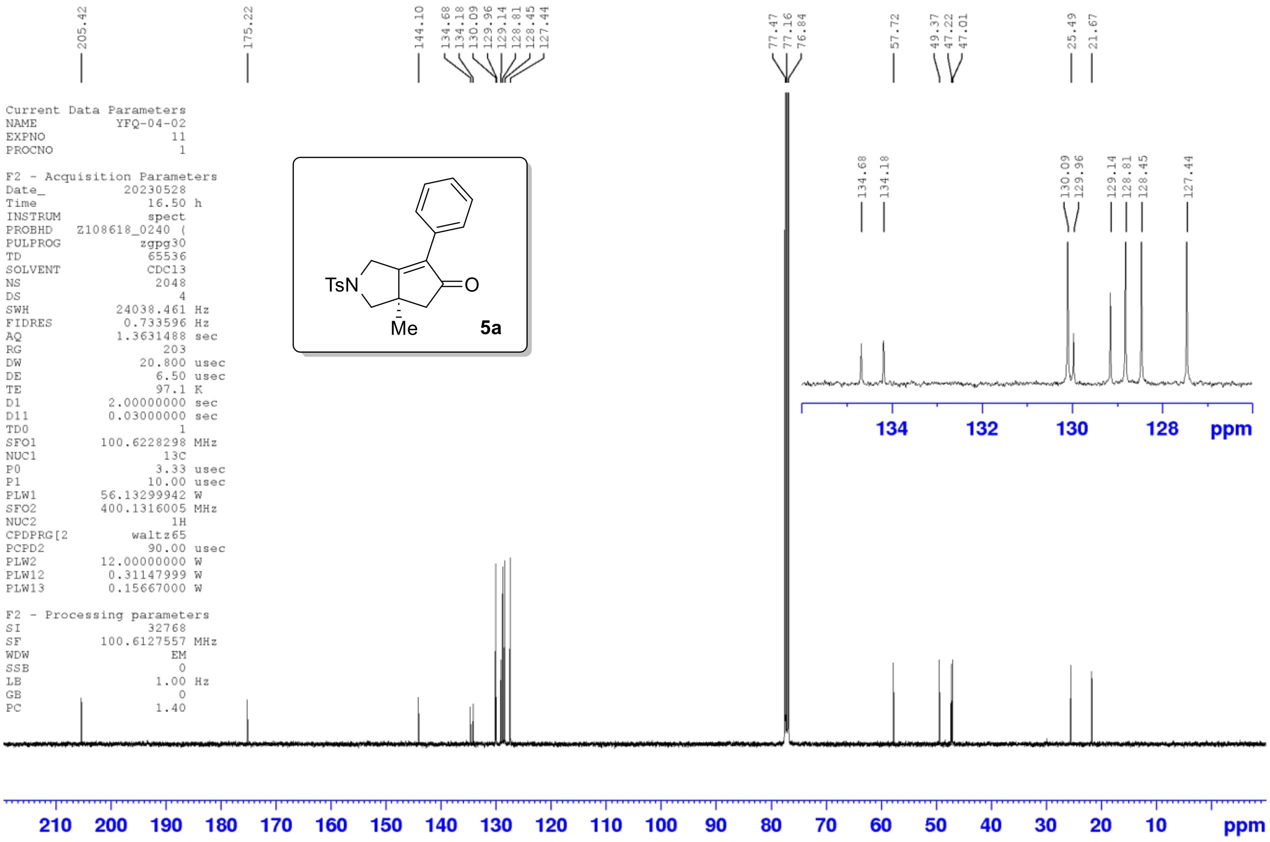

YFQ-04-83 (1H, 400MHz, CDCl3)

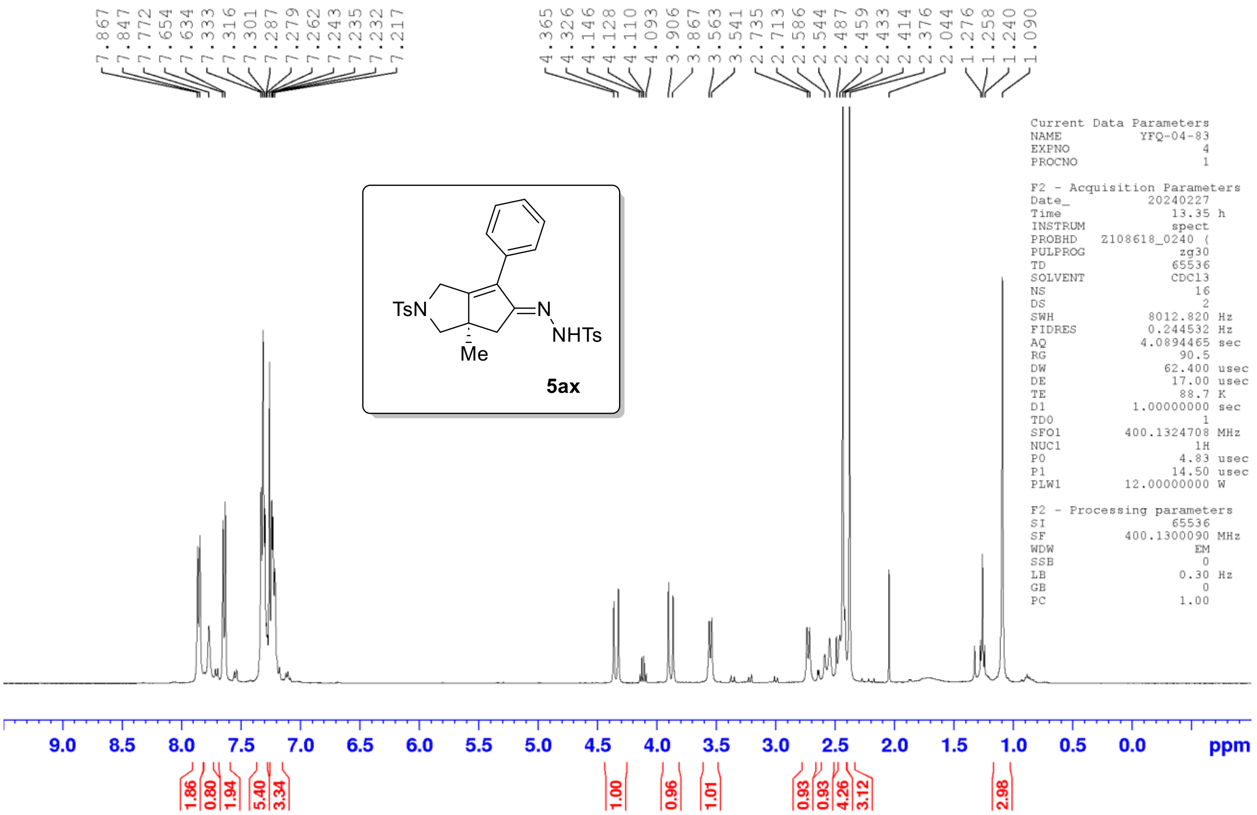

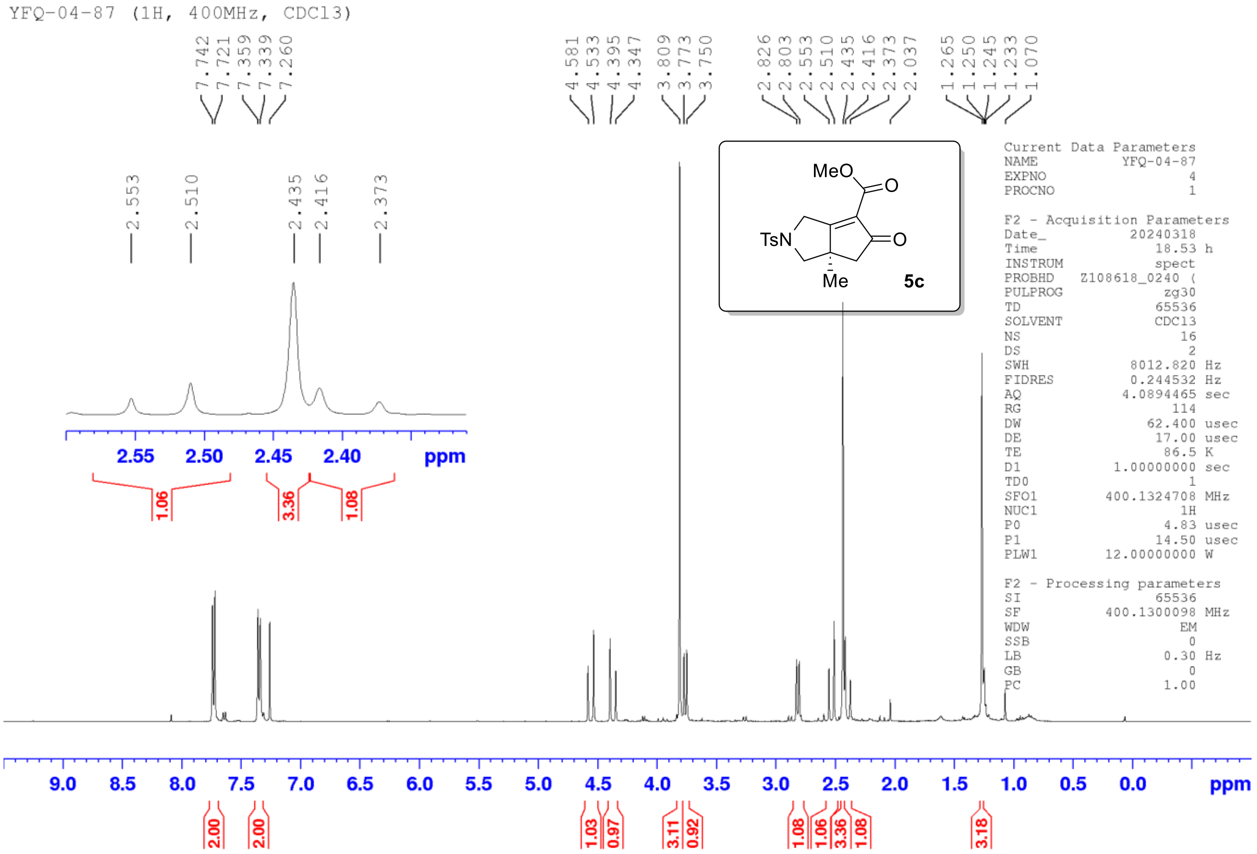
YFQ-04-87 (1H, 400MHz, CDCl3)
7.742 7.721 7.359 7.339 7.260
4.581 4.533 4.395 4.347 3.809 3.773 3.750
2.826 2.803 2.553 2.510 2.435 2.416 2.373 2.037
1.265 1.250 1.245 1.233 1.070
2.553 2.510 2.435 2.416 2.373
2.55 2.50 2.45 2.40 ppm
1.06 3.36 1.08
MeO
O
TsN
O
Me
5c
Current Data Parameters
NAME YFQ-04-87
EXPNO 4
PROCNO 1
F2 - Acquisition Parameters
Date_ 20240318
Time 18.53 h
INSTRUM spect
PROBHD Z108618_0240 (
PULPROG zg30
TD 65536
SOLVENT CDCl3
NS 16
DS 2
SWH 8012.820 Hz
FIDRES 0.244532 Hz
AQ 4.0894465 sec
RG 114
DW 62.400 usec
DE 17.00 usec
TE 86.5 K
D1 1.00000000 sec
TD0 1
SFO1 400.1324708 MHz
NUC1 1H
P0 4.83 usec
P1 14.50 usec
PLW1 12.00000000 W
F2 - Processing parameters
SI 65536
SF 400.1300098 MHz
WDW EM
SSB 0
LB 0.30 Hz
GB 0
PC 1.00
9.0 8.5 8.0 7.5 7.0 6.5 6.0 5.5 5.0 4.5 4.0 3.5 3.0 2.5 2.0 1.5 1.0 0.5 0.0 ppm
2.00 2.00 1.03 0.97 3.11 0.92 1.08 1.06 3.36 1.08 3.18

YFQ-04-20 (1H, 400MHz, CDCl3)

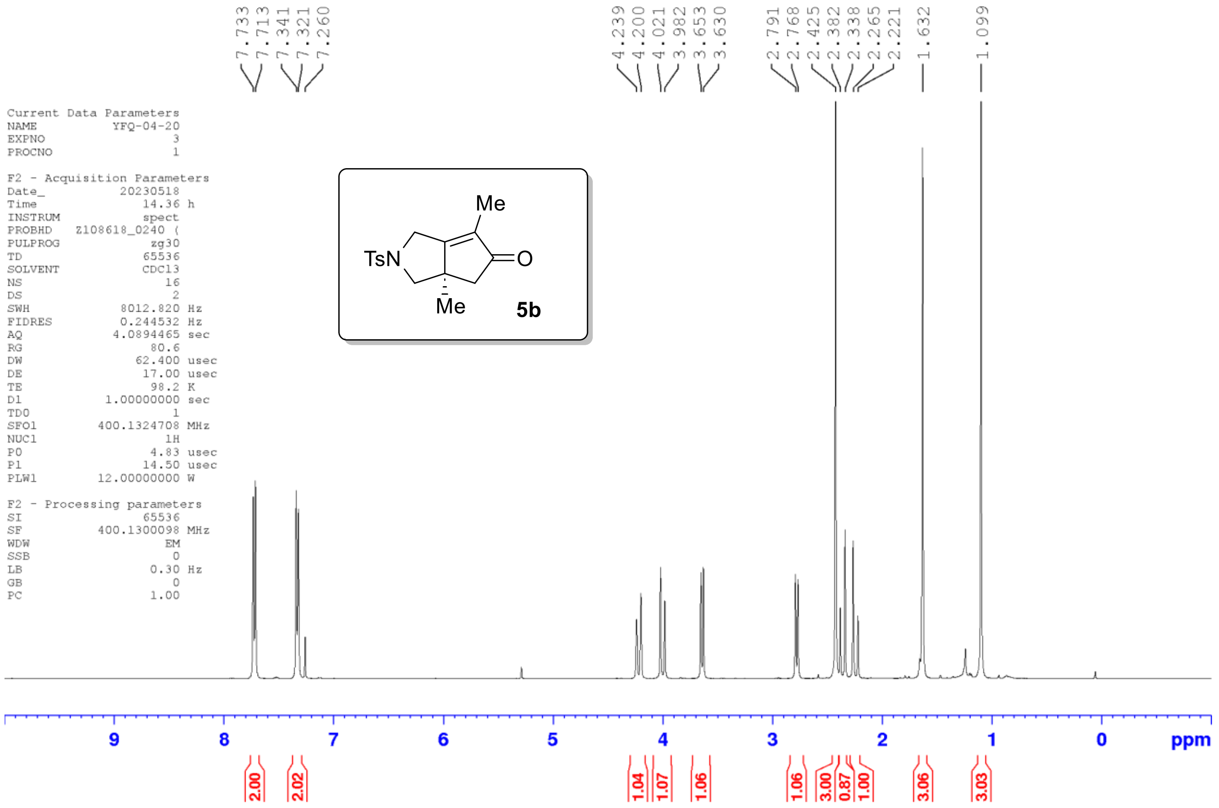

YFQ-04-20 (13C, 100MHz, CDCl3)

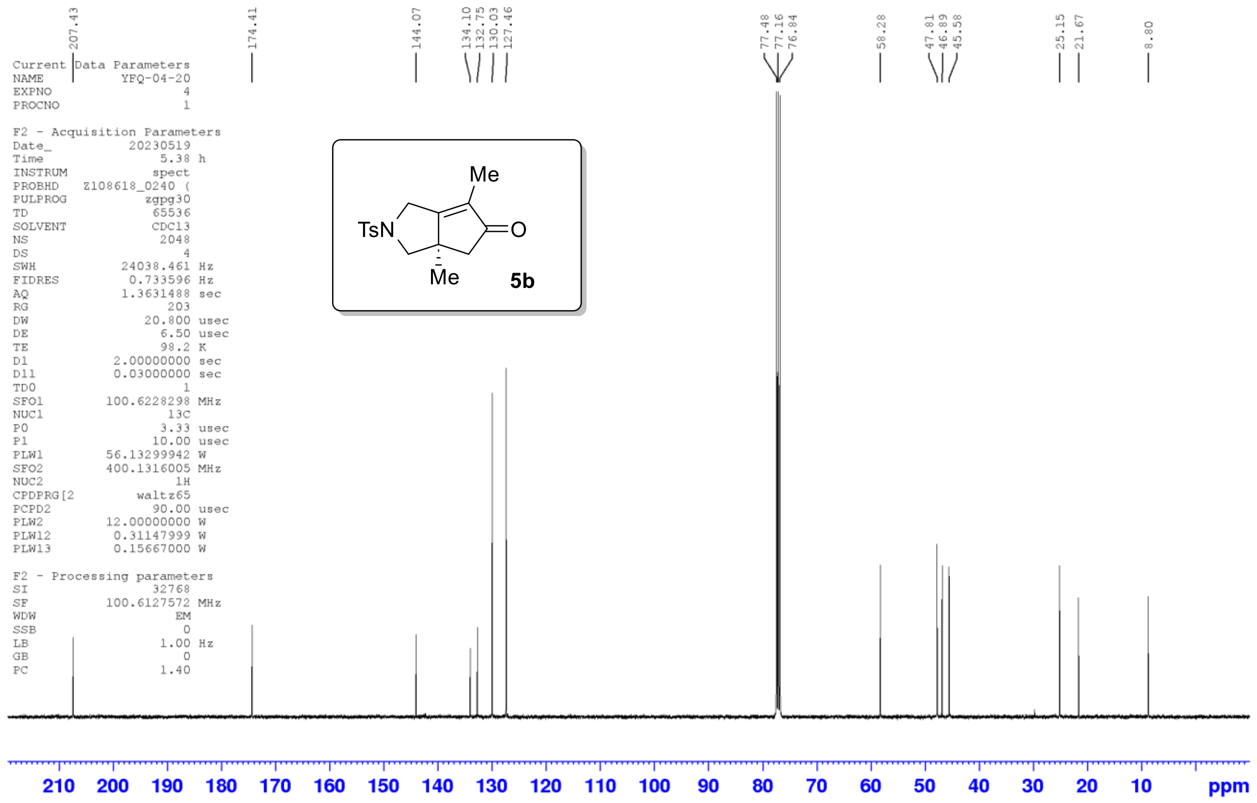

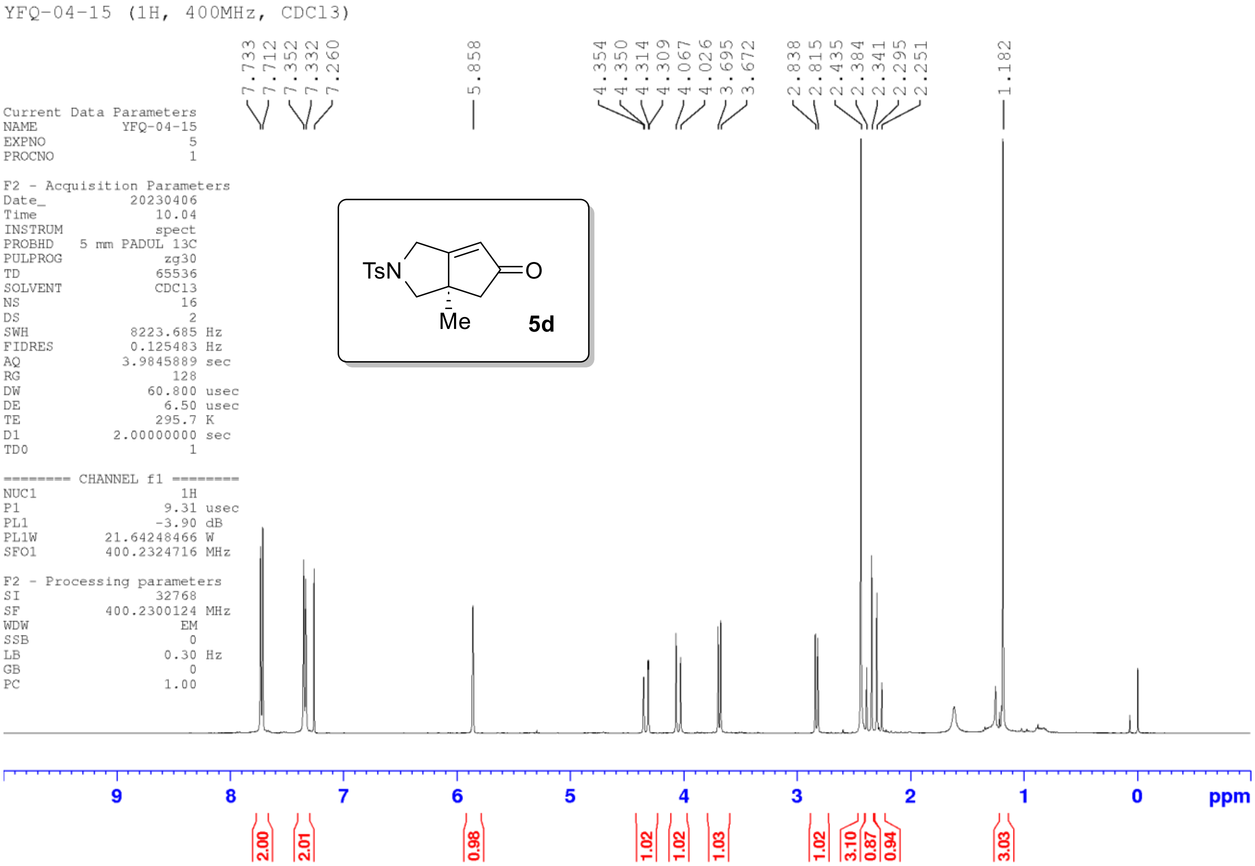
YFQ-04-15 (1H, 400MHz, CDCl3)
7.733
7.712
7.352
7.332
7.260
5.858
4.354
4.350
4.314
4.309
4.067
4.026
3.695
3.672
2.838
2.815
2.435
2.384
2.341
2.295
2.251
1.182
Current Data Parameters
NAME YFQ-04-15
EXPNO 5
PROCNO 1
F2 - Acquisition Parameters
Date_ 20230406
Time 10.04
INSTRUM spect
PROBHD 5 mm PADUL 13C
PULPROG zg30
TD 65536
SOLVENT CDCl3
NS 16
DS 2
SWH 8223.685 Hz
FIDRES 0.125483 Hz
AQ 3.9845889 sec
RG 128
DW 60.800 usec
DE 6.50 usec
TE 295.7 K
D1 2.00000000 sec
TD0 1
======== CHANNEL f1 ========
NUC1 1H
P1 9.31 usec
PL1 -3.90 dB
PL1W 21.64248466 W
SFO1 400.2324716 MHz
F2 - Processing parameters
SI 32768
SF 400.2300124 MHz
WDW EM
SSB 0
LB 0.30 Hz
GB 0
PC 1.00
TsN
O
Me
5d
9
8
7
6
5
4
3
2
1
0
ppm
2.00
2.01
0.98
1.02
1.02
1.03
1.02
3.10
0.87
0.94
3.03

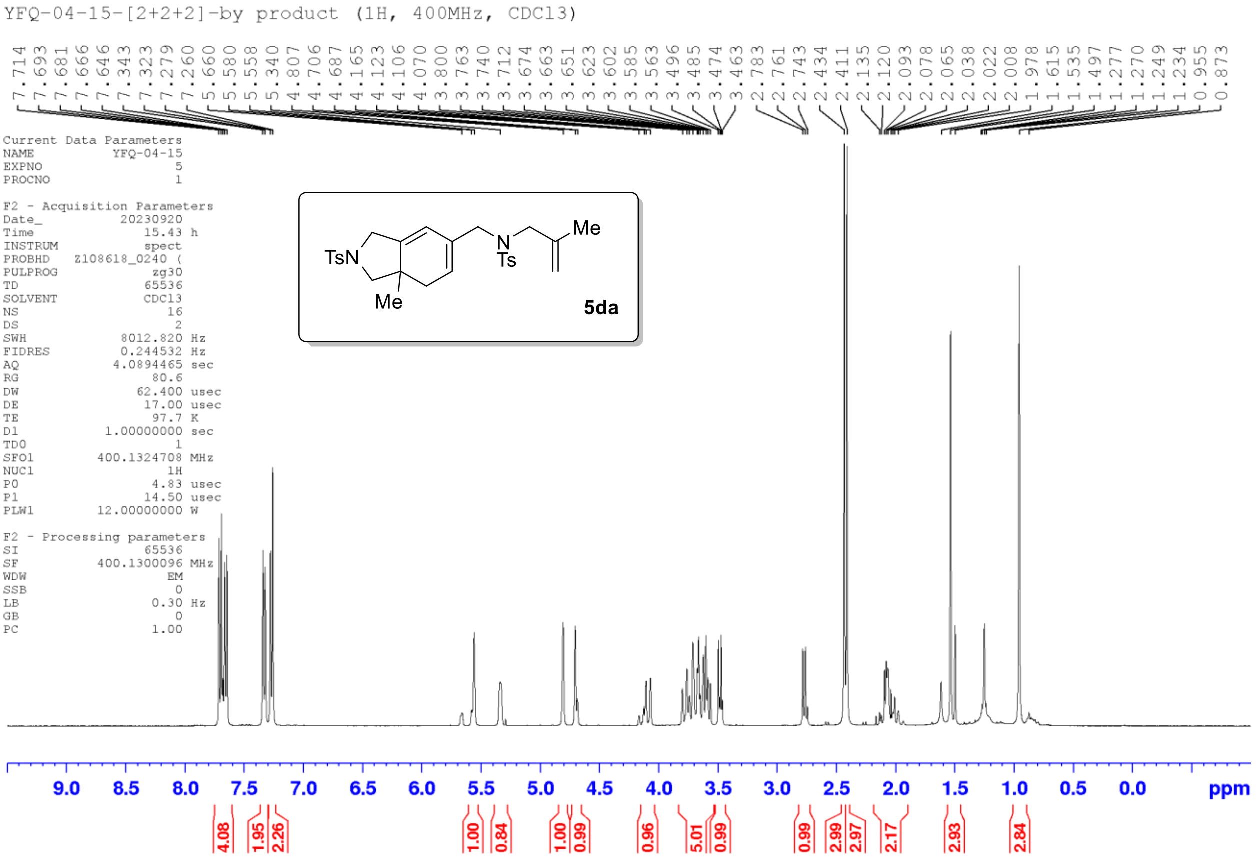
YFQ-04-15-[2+2+2]-by product (1H, 400MHz, CDCl3)
7.714 7.693 7.681 7.666 7.646 7.343 7.323 7.279 7.260 5.660 5.580 5.558 5.340 4.807 4.706 4.687 4.165 4.123 4.106 4.070 3.800 3.763 3.740 3.712 3.674 3.663 3.651 3.623 3.602 3.585 3.563 3.496 3.485 3.474 3.463 2.783 2.761 2.743 2.434 2.411 2.135 2.120 2.093 2.078 2.065 2.038 2.022 2.008 1.978 1.615 1.535 1.497 1.277 1.270 1.249 1.234 0.955 0.873
Current Data Parameters
NAME YFQ-04-15
EXPNO 5
PROCNO 1
F2 - Acquisition Parameters
Date_ 20230920
Time 15.43 h
INSTRUM spect
PROBHD Z108618_0240 (
PULPROG zg30
TD 65536
SOLVENT CDCl3
NS 16
DS 2
SWH 8012.820 Hz
FIDRES 0.244532 Hz
AQ 4.0894465 sec
RG 80.6
DW 62.400 usec
DE 17.00 usec
TE 97.7 K
D1 1.00000000 sec
TD0 1
SFO1 400.1324708 MHz
NUC1 1H
P0 4.83 usec
P1 14.50 usec
PLW1 12.00000000 W
F2 - Processing parameters
SI 65536
SF 400.1300096 MHz
WDW EM
SSB 0
LB 0.30 Hz
GB 0
PC 1.00
TsN
Me
N
Ts
Me
5da
9.0 8.5 8.0 7.5 7.0 6.5 6.0 5.5 5.0 4.5 4.0 3.5 3.0 2.5 2.0 1.5 1.0 0.5 0.0 ppm
4.08 1.95 2.26 1.00 0.84 1.00 0.99 0.96 5.01 0.99 0.99 2.99 2.97 2.17 2.93 2.84

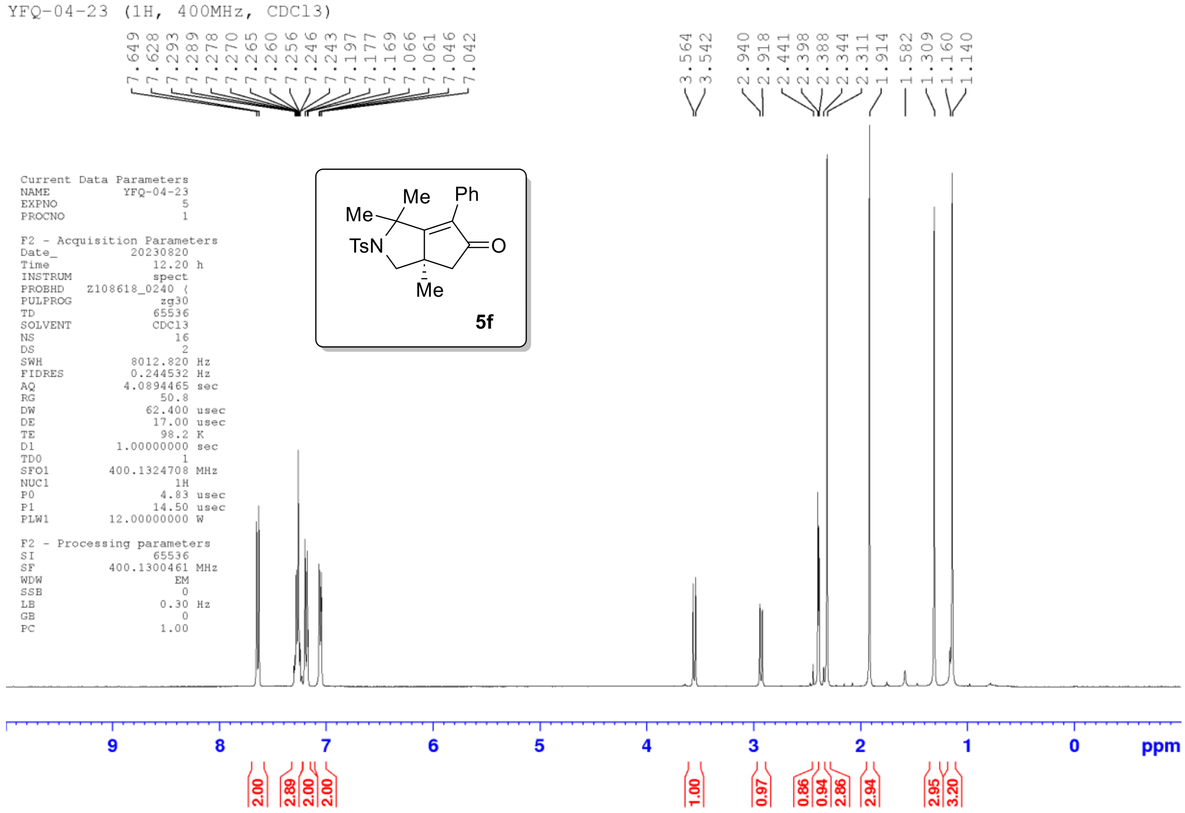
YFQ-04-23 (1H, 400MHz, CDCl3)
7.649 7.628 7.293 7.289 7.278 7.270 7.265 7.260 7.256 7.246 7.243 7.197 7.177 7.169 7.066 7.061 7.046 7.042
3.564 3.542
2.940 2.918 2.441 2.398 2.388 2.344 2.311 1.914
1.582 1.309 1.160 1.140
Me
Ph
Me
TsN
O
Me
5f
2.00 2.89 2.00 2.00 1.00 0.97 0.86 0.94 2.86 2.94 2.95 3.20
ppm

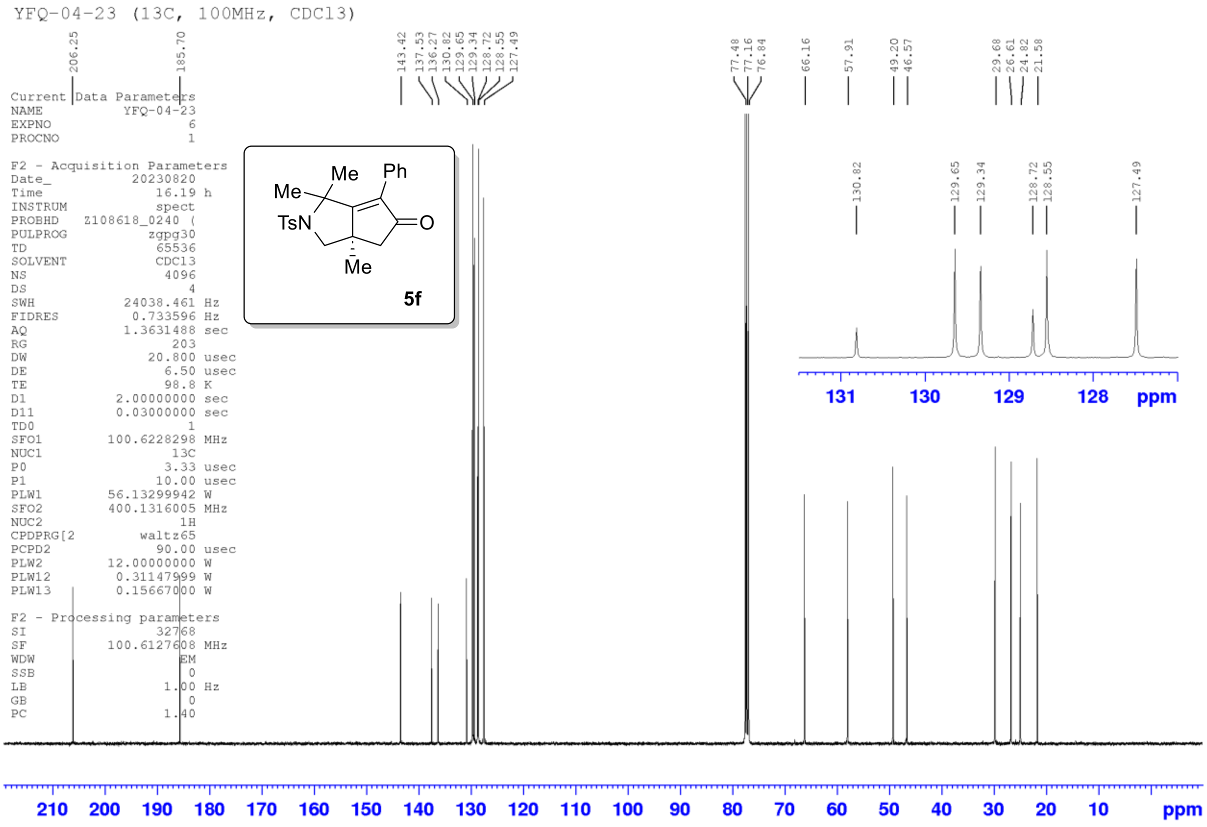
YFQ-04-23 (13C, 100MHz, CDCl3)
206.25 185.70
143.42 137.53 136.27 130.82 129.65 129.34 128.72 128.55 127.49
77.48 77.16 76.84
66.16 57.91 49.20 46.57
29.68 26.61 24.82 21.58
Me
Ph
Me
TsN
O
Me
5f
ppm

YFQ-04-05 (1H, 400MHz, CDCl3)

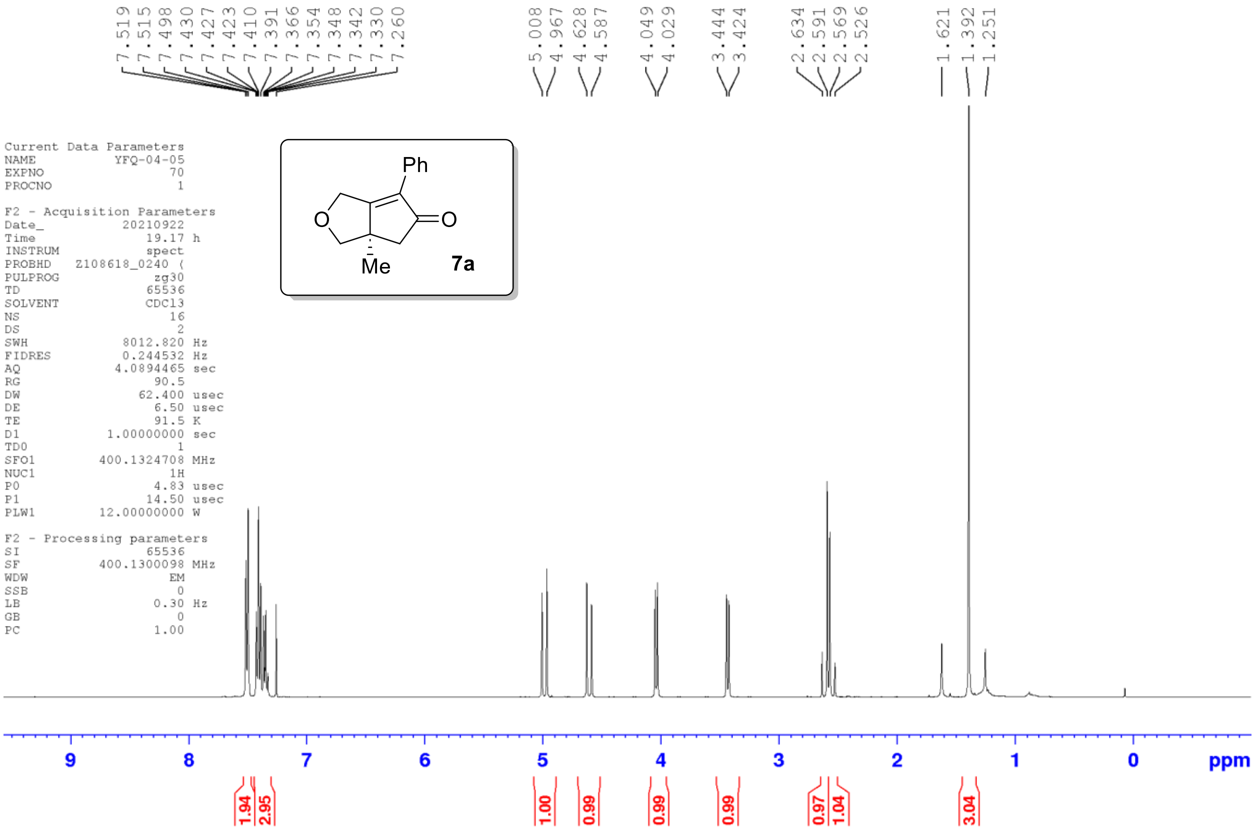


```
Current Data Parameters
NAME          YFQ-04-05
EXPNO                70
PROCNO                1

F2 - Acquisition Parameters
Date_          20210922
Time              19.17 h
INSTRUM           spect
PROBHD   Z108618_0240 (
PULPROG            zg30
TD                65536
SOLVENT           CDCl3
NS                   16
DS                    2
SWH            8012.820 Hz
FIDRES         0.244532 Hz
AQ            4.0894465 sec
RG                 90.5
DW               62.400 usec
DE                 6.50 usec
TE                 91.5 K
D1           1.00000000 sec
TD0                   1
SFO1        400.1324708 MHz
NUC1                 1H
P0                 4.83 usec
P1                14.50 usec
PLW1        12.00000000 W

F2 - Processing parameters
SI                65536
SF          400.1300098 MHz
WDW                  EM
SSB                   0
LB                 0.30 Hz
GB                    0
PC                 1.00
```

7a

YFQ-04-41 (1H, 400MHz, CDCl3)

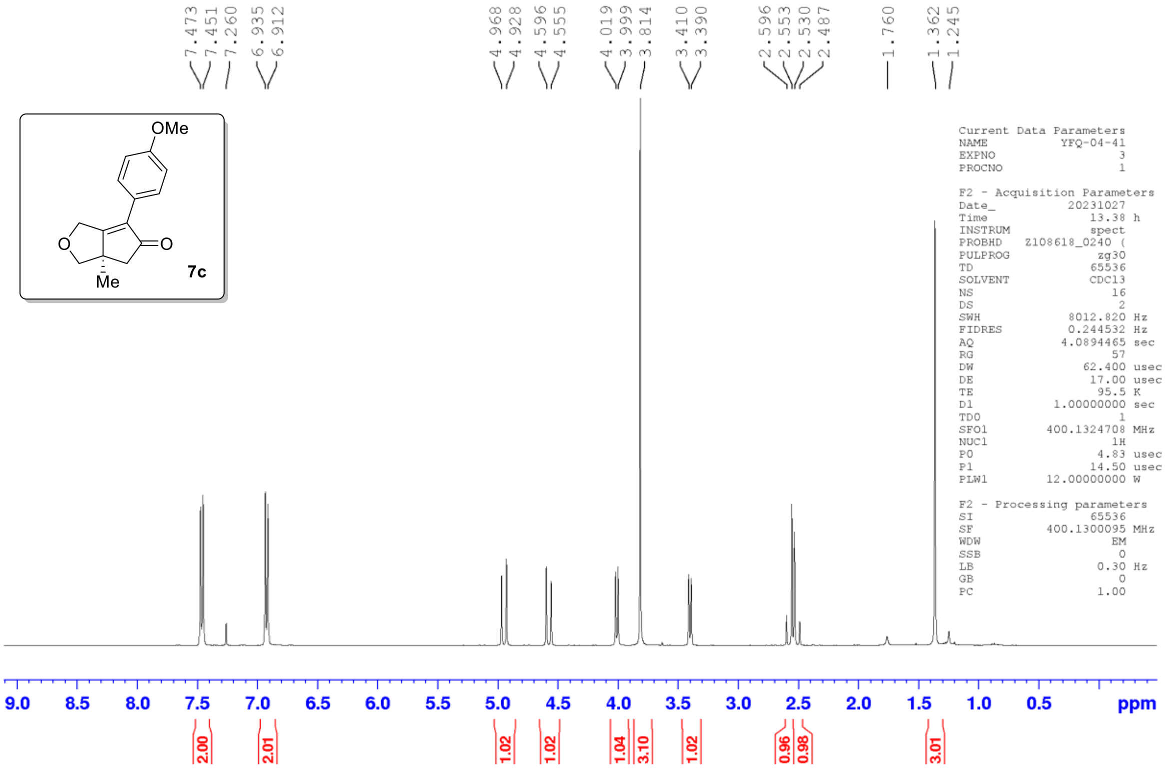

YFQ-04-41 (13C, 400MHz, CDCl3)

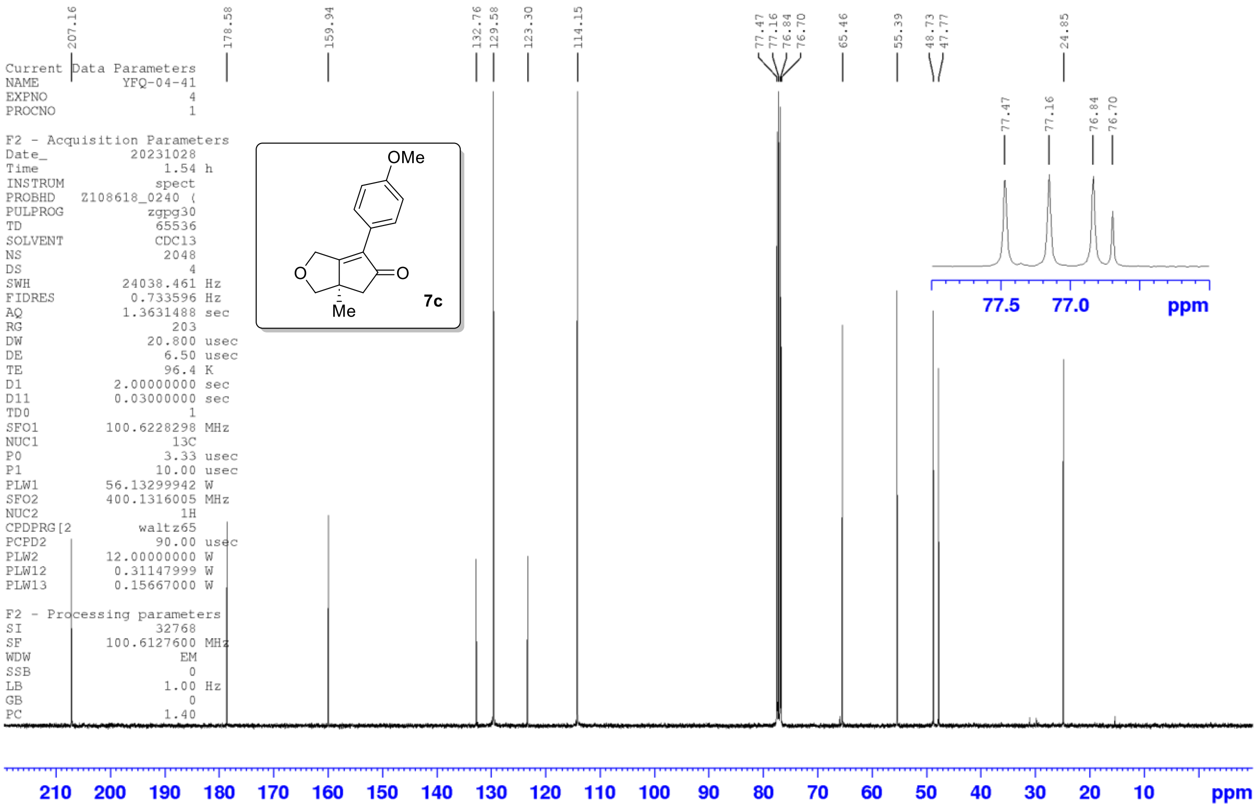

YFQ-04-38 (1H, 400MHz, CDCl3)

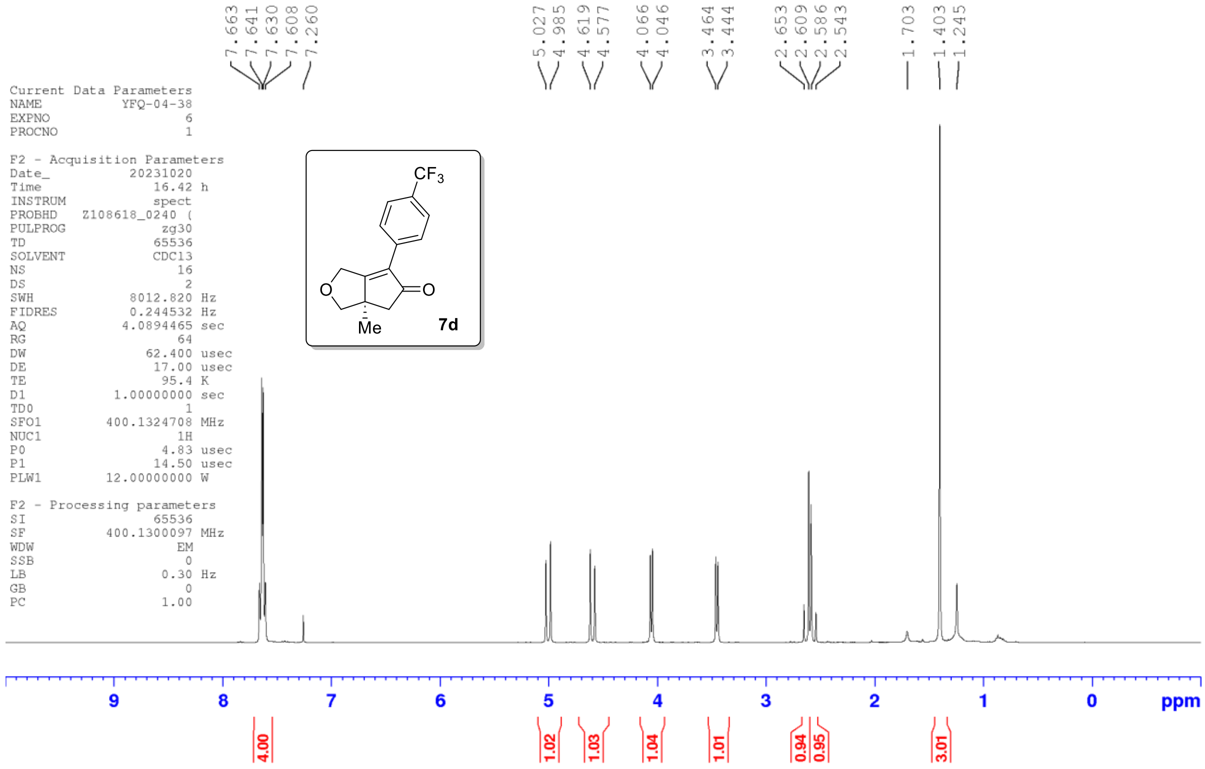

YFQ-04-38 (13C, 100MHz, CDCl3)

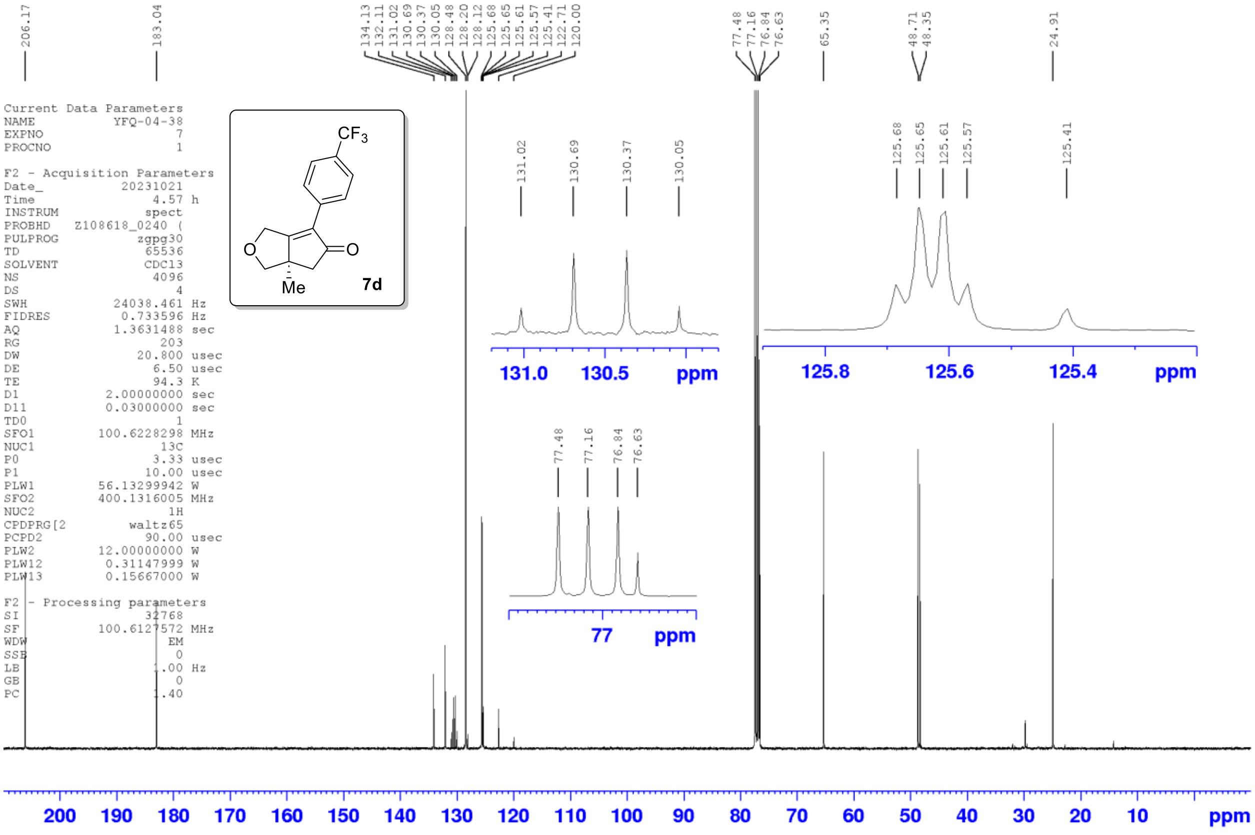

YFQ-04-40 (1H, 400MHz, CDCl3)

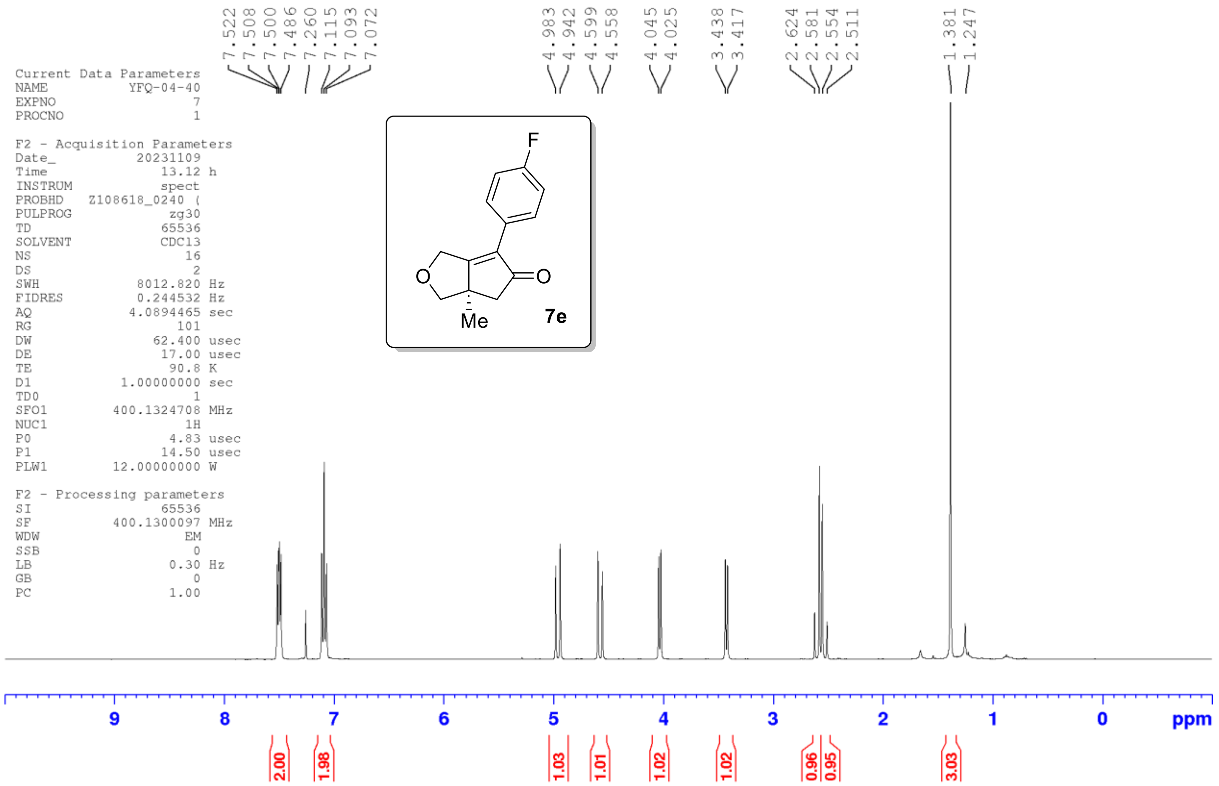

YFQ-04-40 (13C, 100MHz, CDCl3)

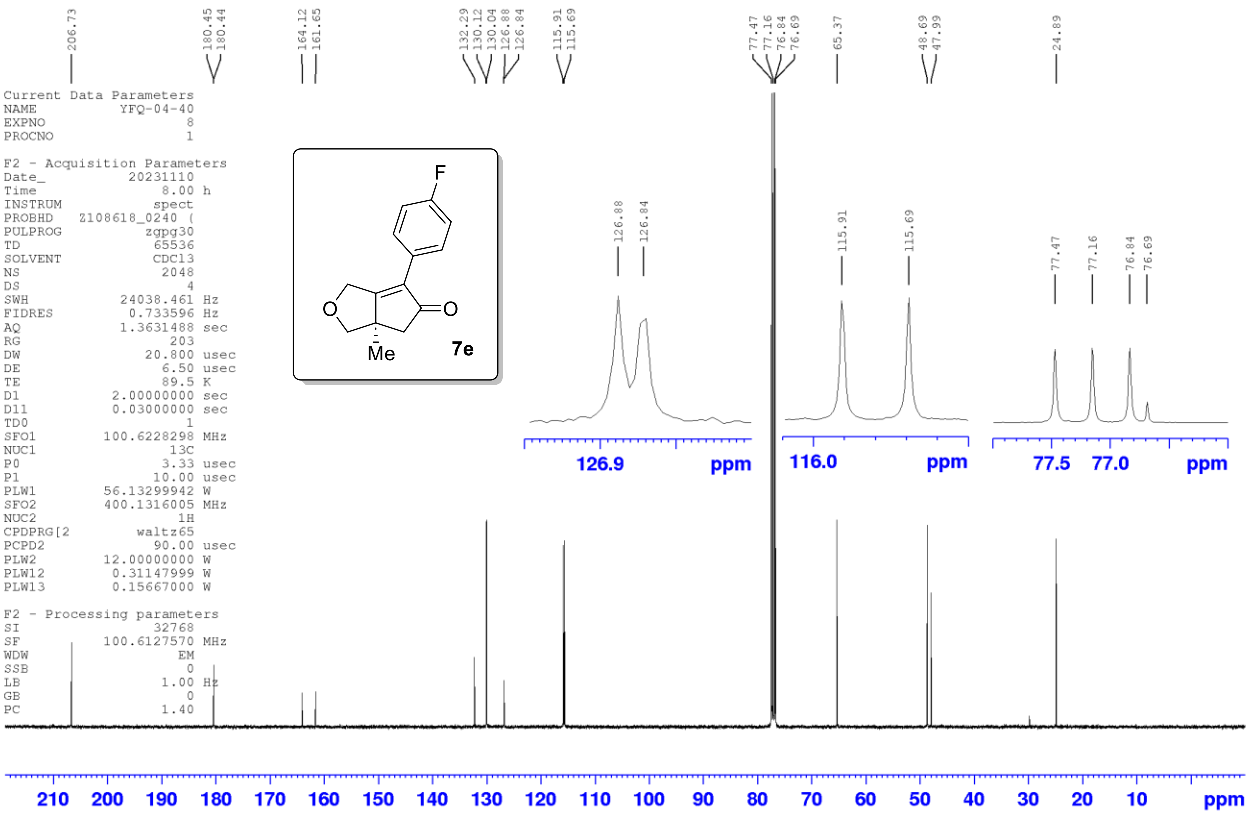

YFQ-04-12 (1H, 400MHz, CDCl3)

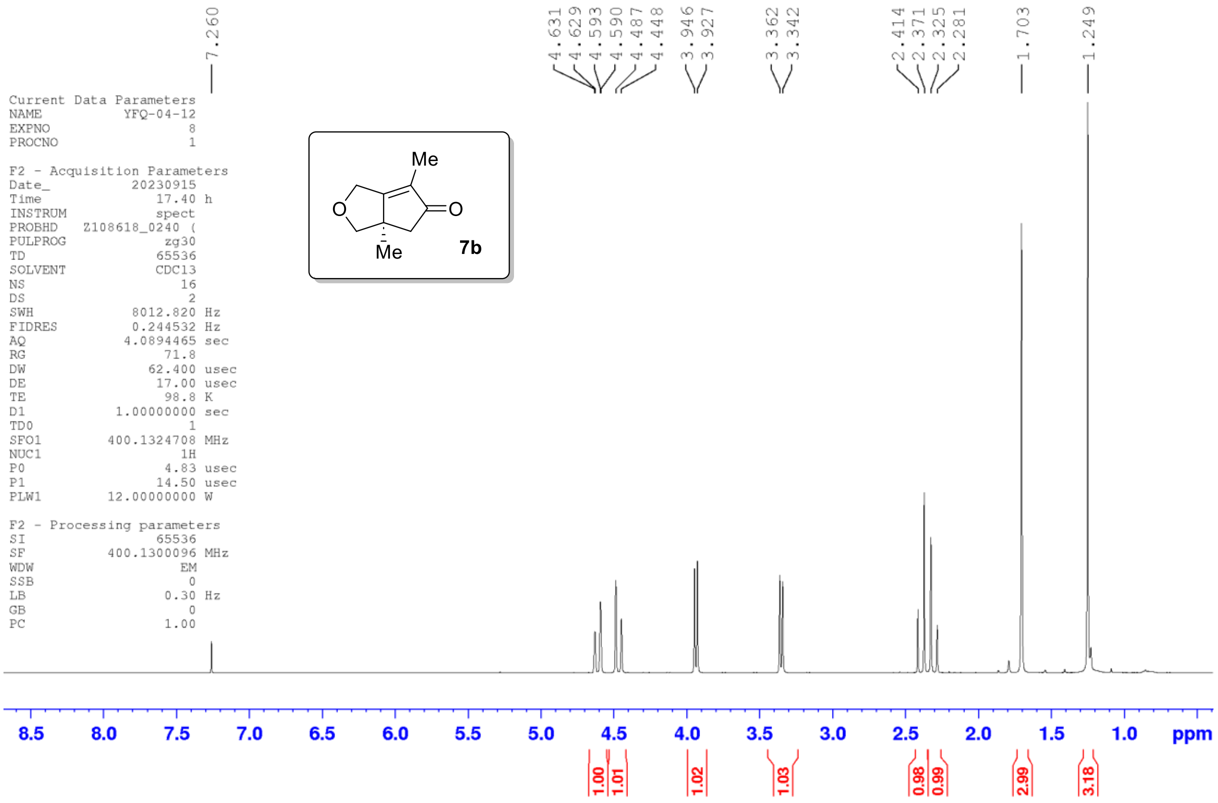

YFQ-04-12 (13C, 100MHz, CDCl3)

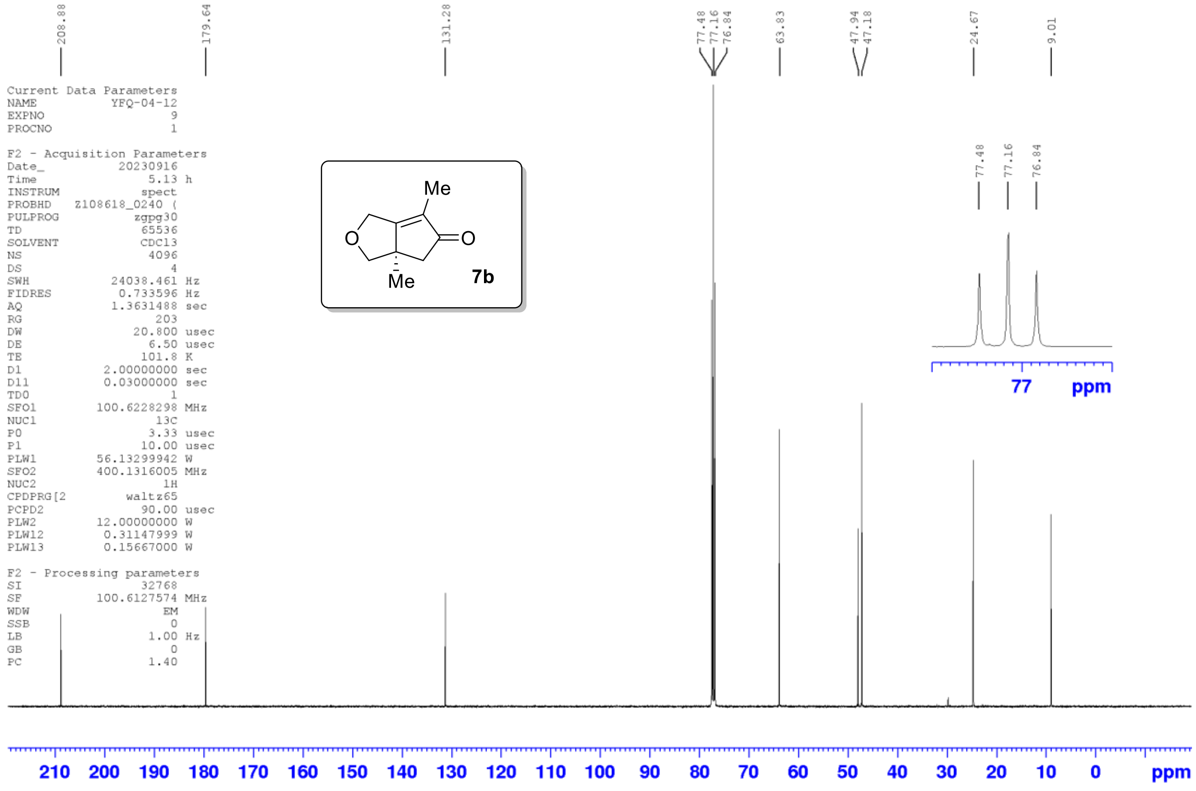

YFQ-03-105 (1H, 400MHz, CDCl3)

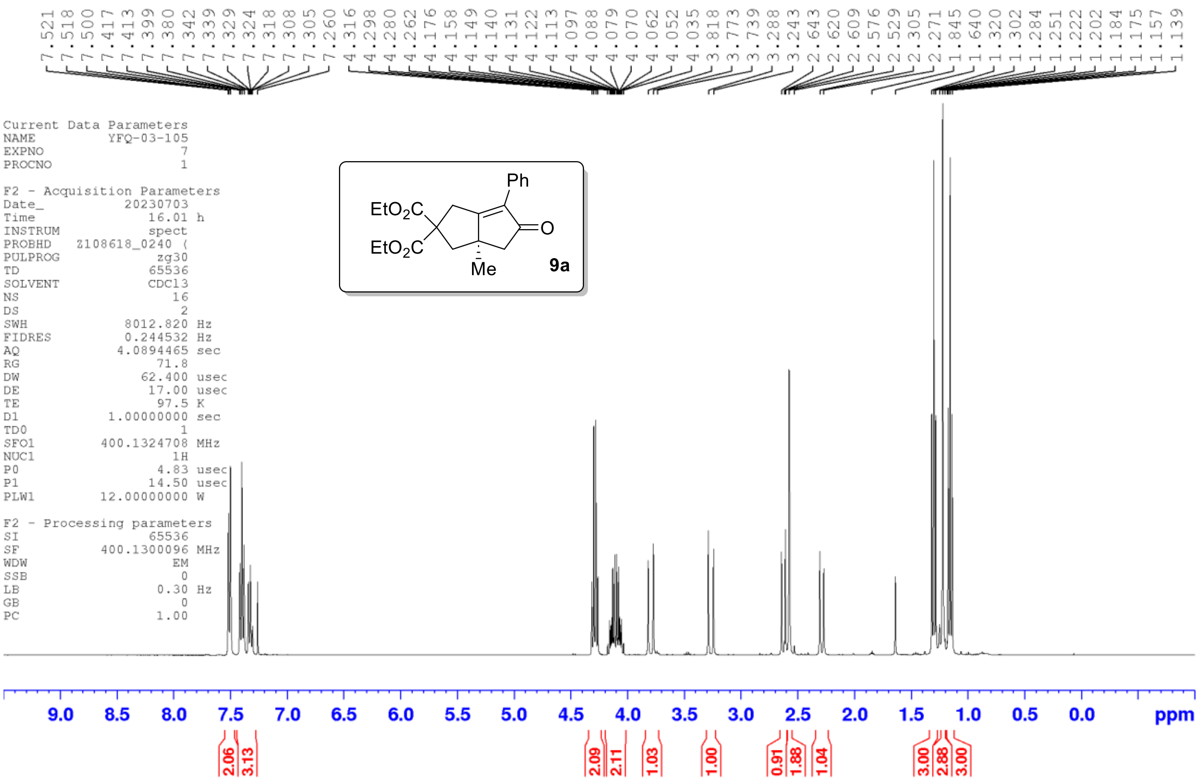

YFQ-03-105 (13C, 100MHz, CDCl3)

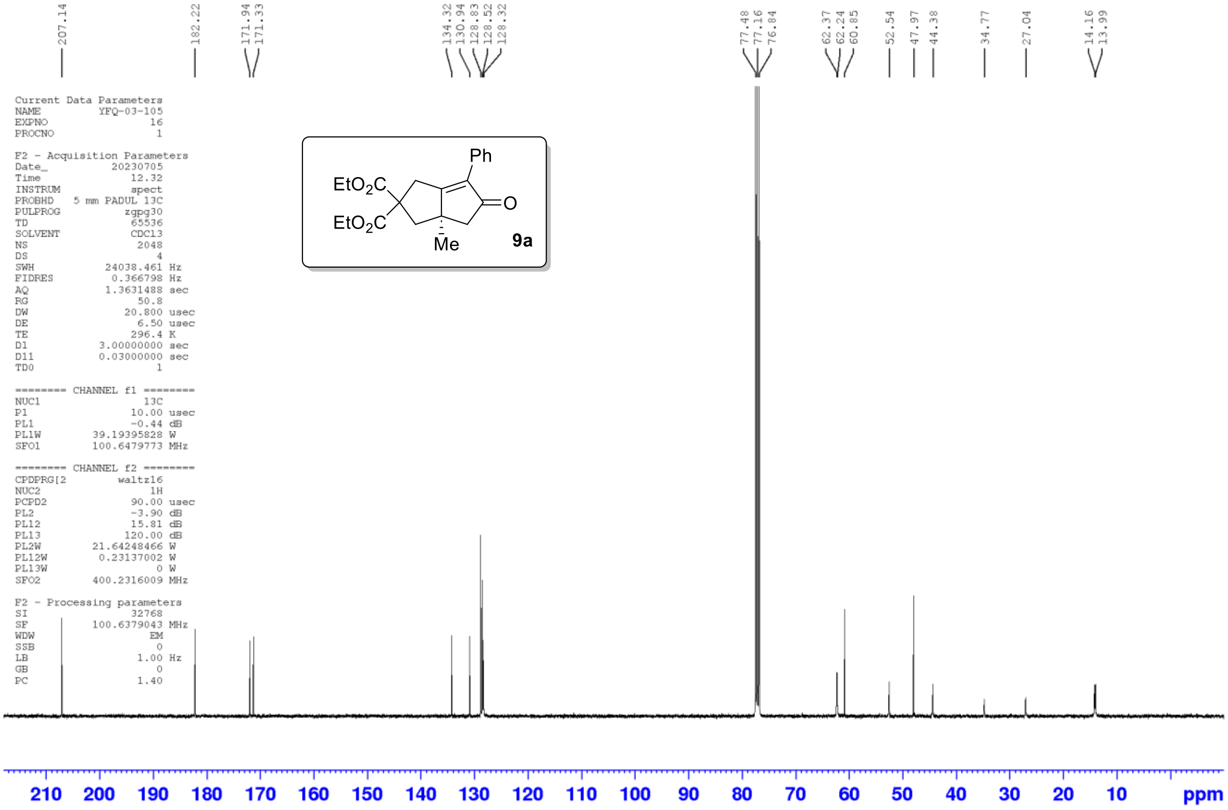

YFQ-03-110 (1H, 400MHz, CDCl3)

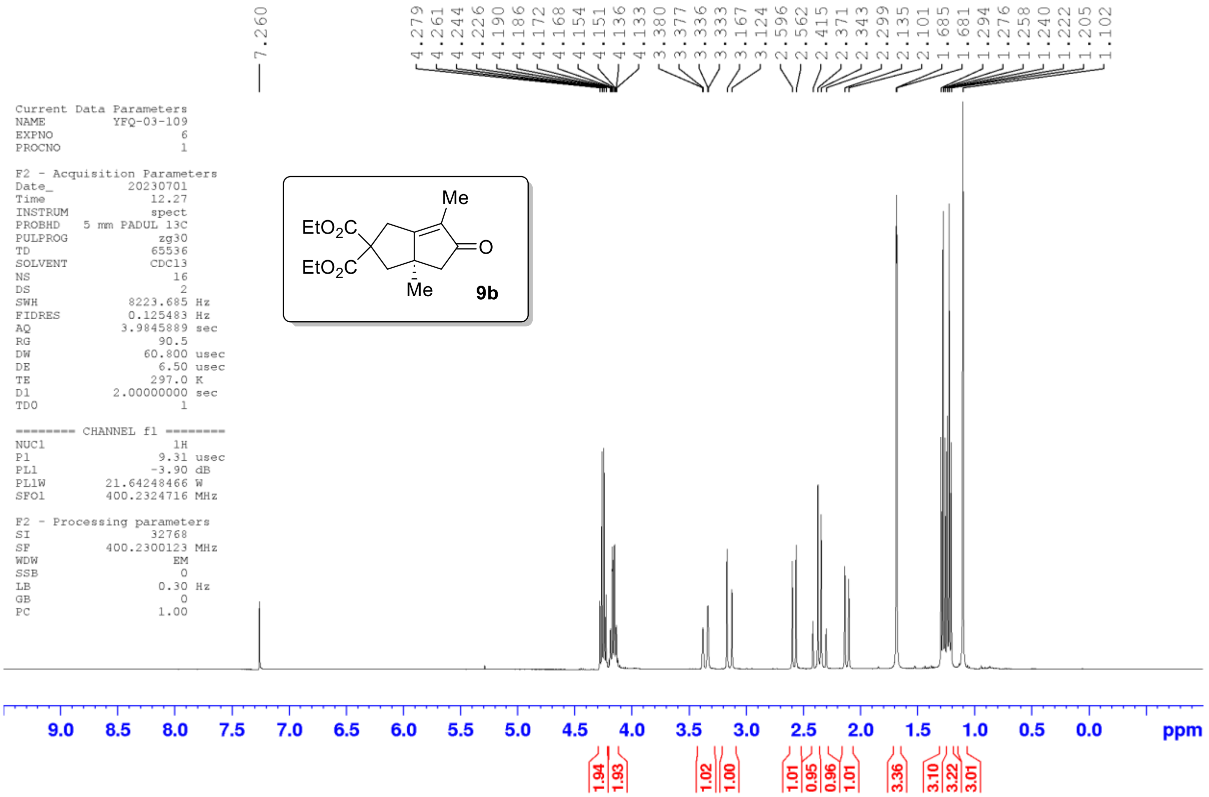

YFQ-03-110 (13C, 400MHz, CDCl3)

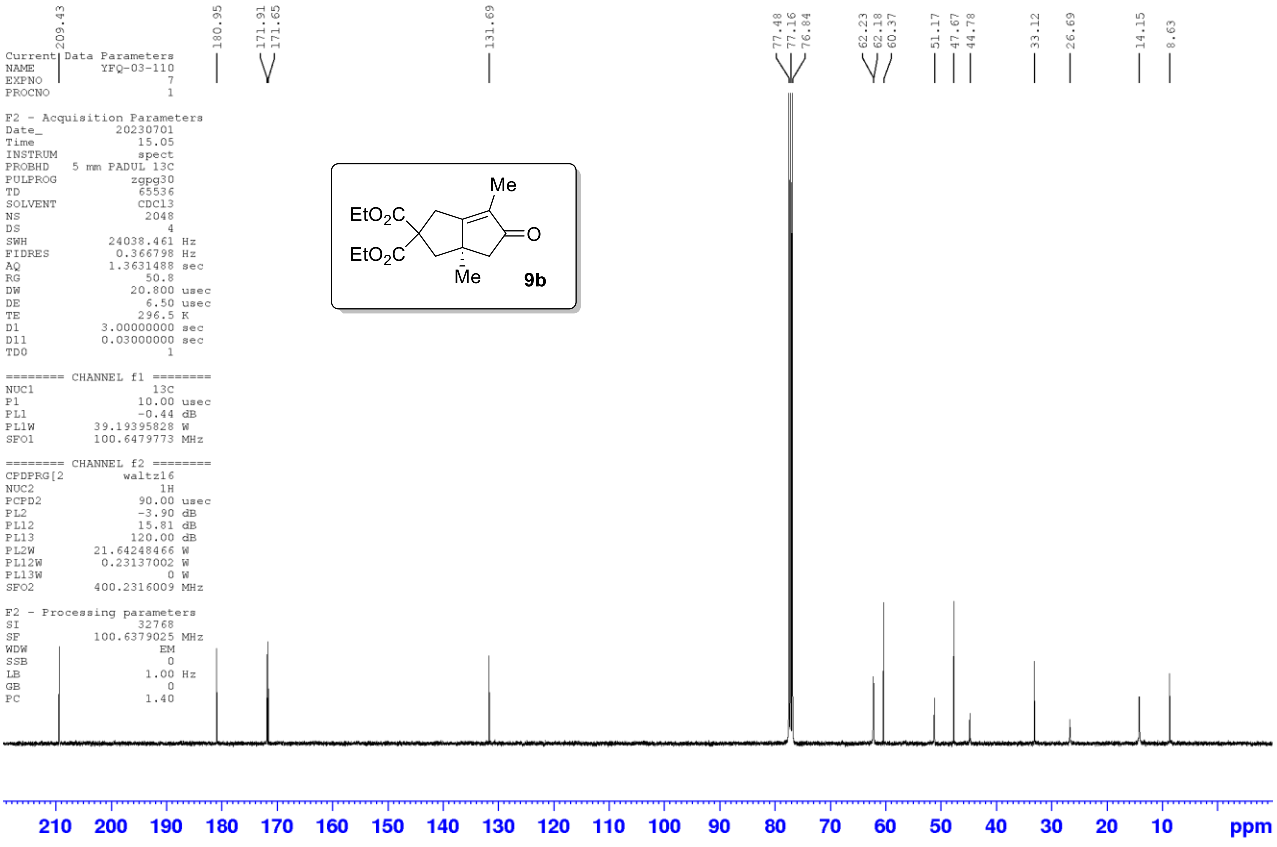

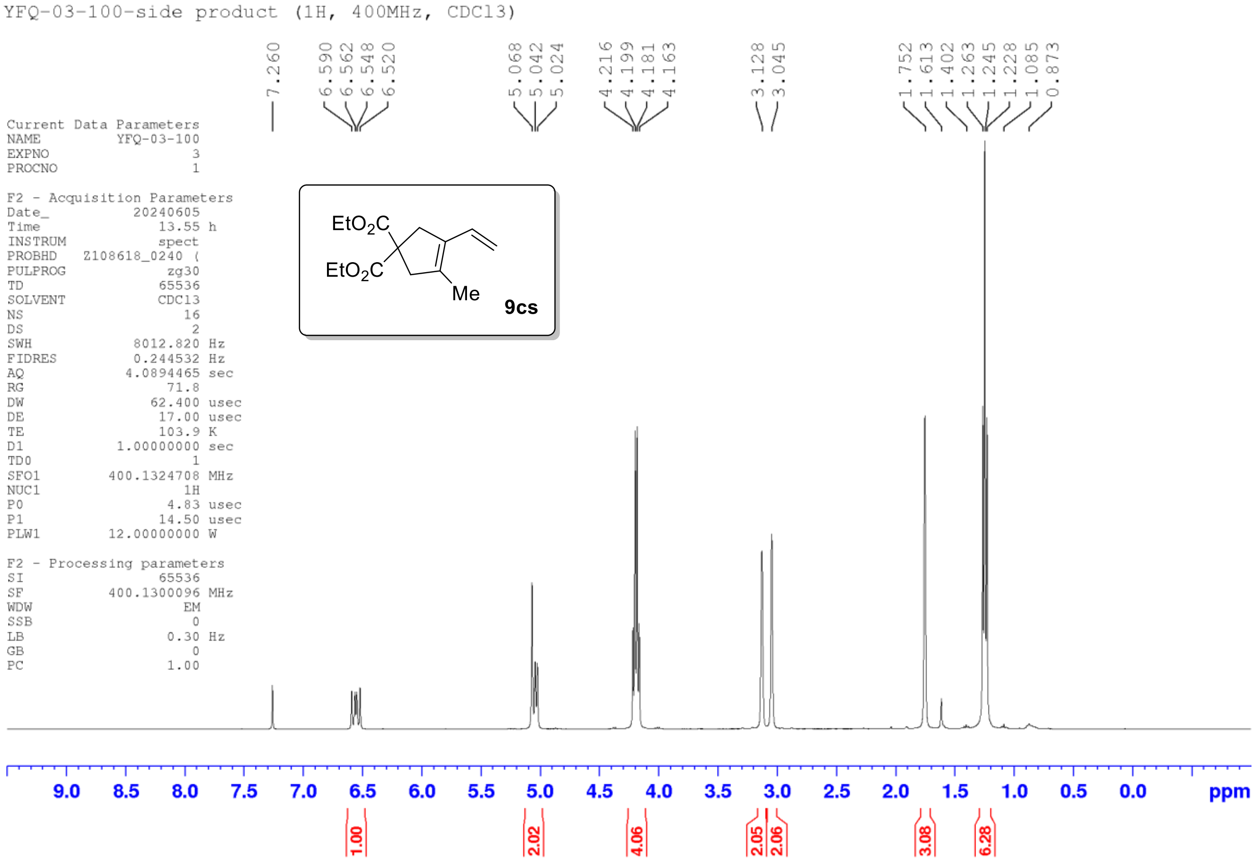
YFQ-03-100-side product (1H, 400MHz, CDCl3)
7.260
6.590
6.562
6.548
6.520
5.068
5.042
5.024
4.216
4.199
4.181
4.163
3.128
3.045
1.752
1.613
1.402
1.263
1.245
1.228
1.085
0.873
Current Data Parameters
NAME YFQ-03-100
EXPNO 3
PROCNO 1
F2 - Acquisition Parameters
Date_ 20240605
Time 13.55 h
INSTRUM spect
PROBHD Z108618_0240 (
PULPROG zg30
TD 65536
SOLVENT CDCl3
NS 16
DS 2
SWH 8012.820 Hz
FIDRES 0.244532 Hz
AQ 4.0894465 sec
RG 71.8
DW 62.400 usec
DE 17.00 usec
TE 103.9 K
D1 1.00000000 sec
TD0 1
SFO1 400.1324708 MHz
NUC1 1H
P0 4.83 usec
P1 14.50 usec
PLW1 12.00000000 W
F2 - Processing parameters
SI 65536
SF 400.1300096 MHz
WDW EM
SSB 0
LB 0.30 Hz
GB 0
PC 1.00
EtO2C
EtO2C
Me
9cs
9.0
8.5
8.0
7.5
7.0
6.5
6.0
5.5
5.0
4.5
4.0
3.5
3.0
2.5
2.0
1.5
1.0
0.5
0.0
ppm
1.00
2.02
4.06
2.05
2.06
3.08
6.28

YFQ-03-121 (1H, 400MHz, CDCl3)

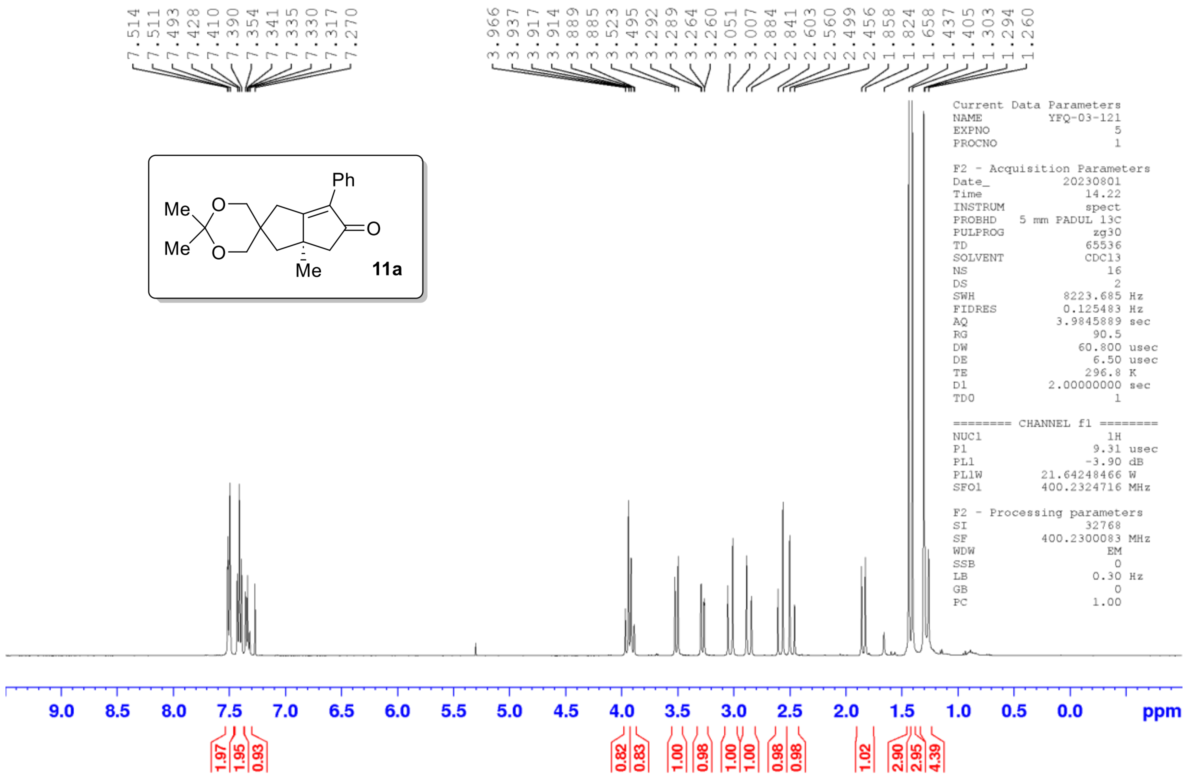

YFQ-03-121 (13C, 100MHz, CDCl3)

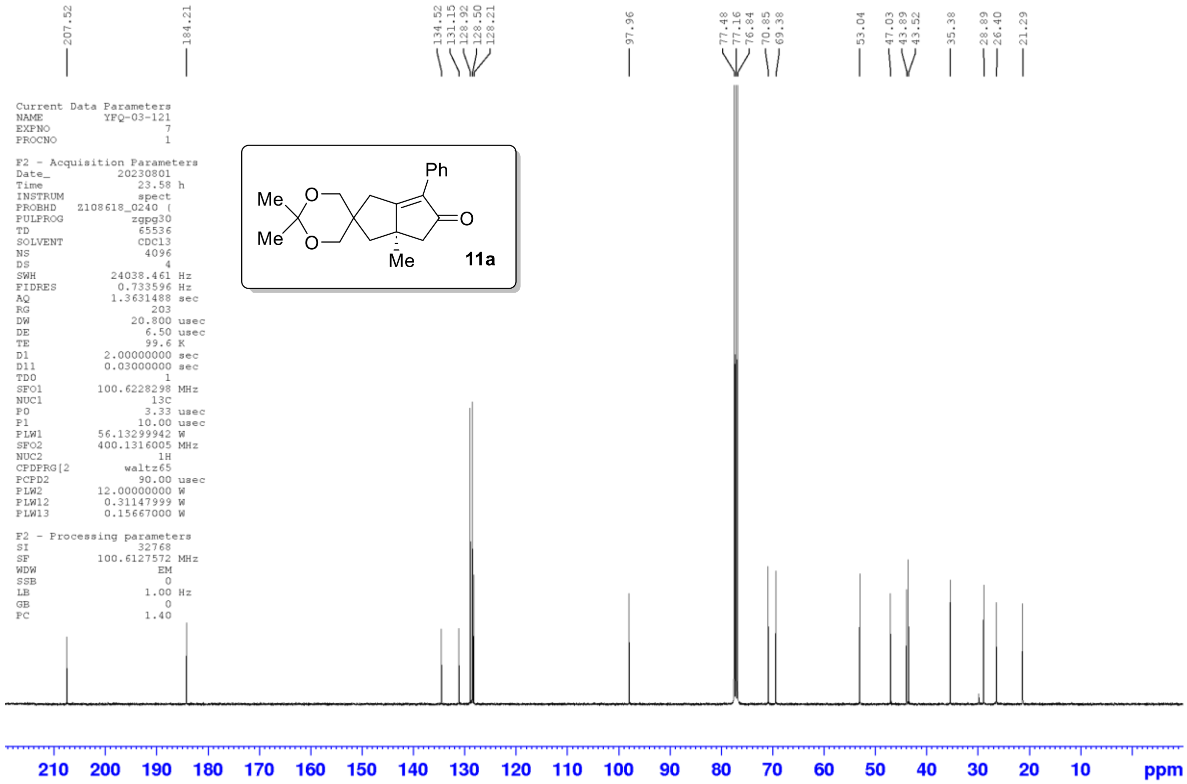

YFQ-01-14 (1H, 400MHz, CDCl3)

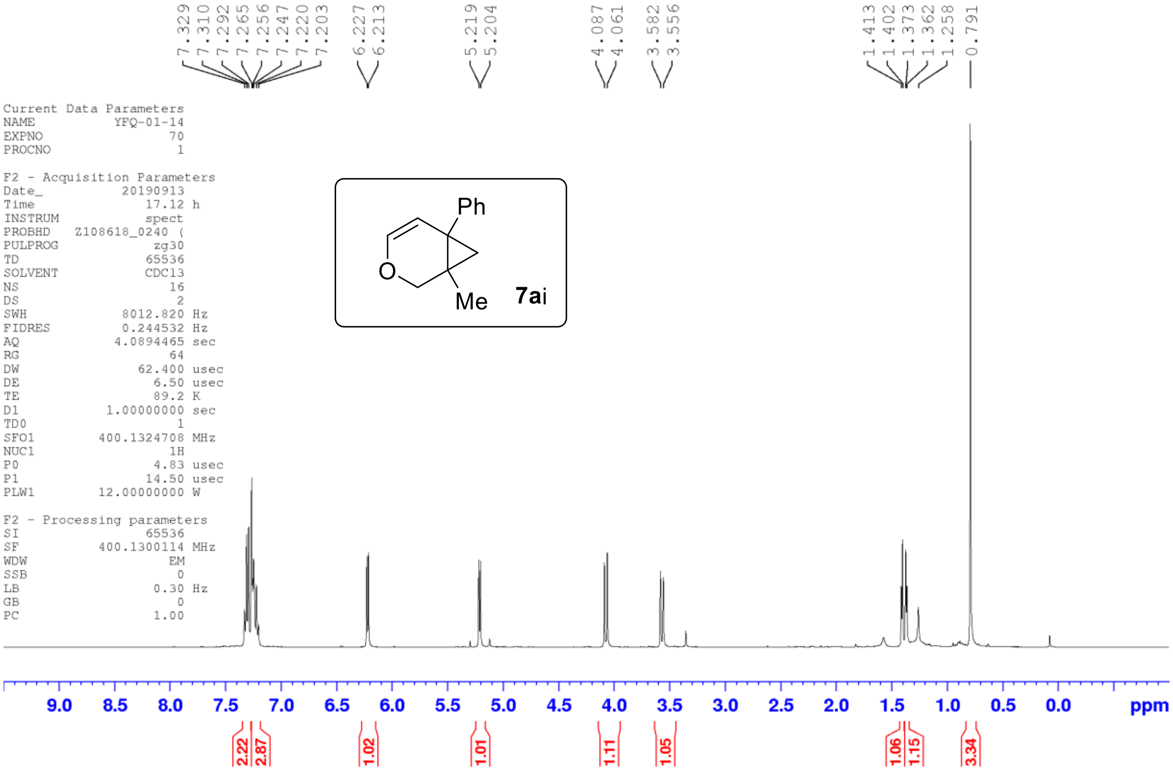

YFQ-01-14 (13C, 100MHz, CDCl3)

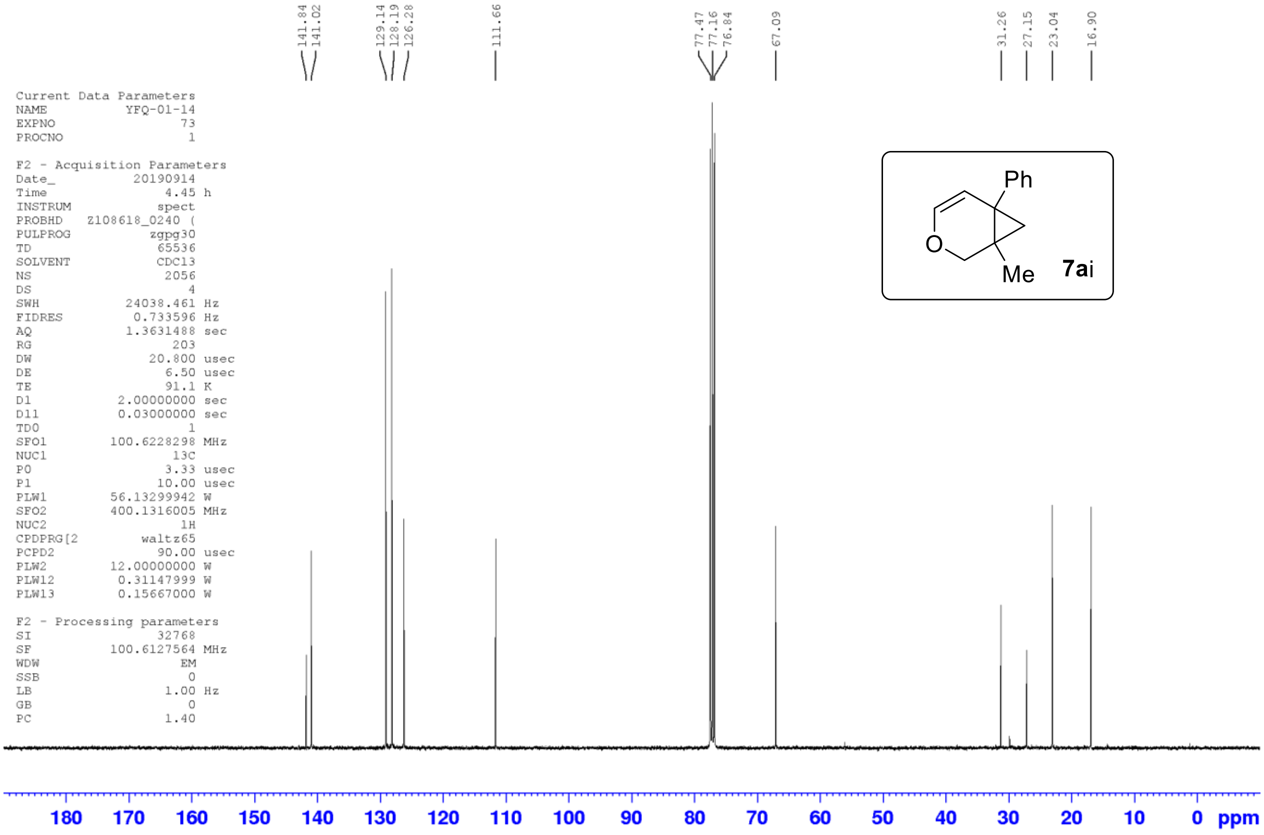

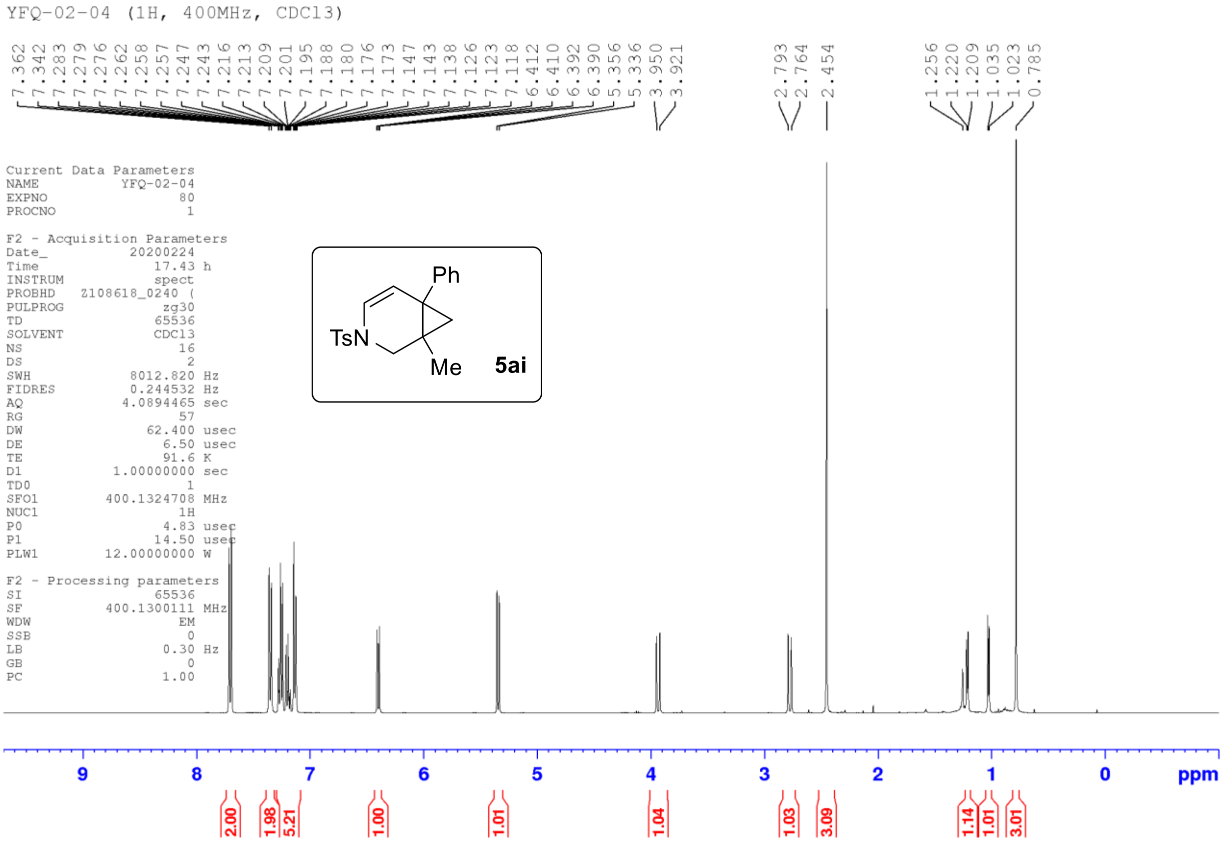
YFQ-02-04 (1H, 400MHz, CDCl3)
7.362 7.342 7.283 7.279 7.276 7.262 7.258 7.257 7.247 7.243 7.216 7.213 7.209 7.201 7.195 7.188 7.180 7.176 7.173 7.147 7.143 7.138 7.126 7.123 7.118 6.412 6.410 6.392 6.390 5.356 5.336 3.950 3.921
2.793 2.764 2.454
1.256 1.220 1.209 1.035 1.023 0.785
Current Data Parameters
NAME YFQ-02-04
EXPNO 80
PROCNO 1
F2 - Acquisition Parameters
Date_ 20200224
Time 17.43 h
INSTRUM spect
PROBHD Z108618_0240 (
PULPROG zg30
TD 65536
SOLVENT CDCl3
NS 16
DS 2
SWH 8012.820 Hz
FIDRES 0.244532 Hz
AQ 4.0894465 sec
RG 57
DW 62.400 usec
DE 6.50 usec
TE 91.6 K
D1 1.00000000 sec
TD0 1
SFO1 400.1324708 MHz
NUC1 1H
P0 4.83 usec
P1 14.50 usec
PLW1 12.00000000 W
F2 - Processing parameters
SI 65536
SF 400.1300111 MHz
WDW EM
SSB 0
LB 0.30 Hz
GB 0
PC 1.00
Ph
TsN
Me
5ai
9 8 7 6 5 4 3 2 1 0 ppm
2.00 1.98 5.21 1.00 1.01 1.04 1.03 3.09 1.14 1.01 3.01

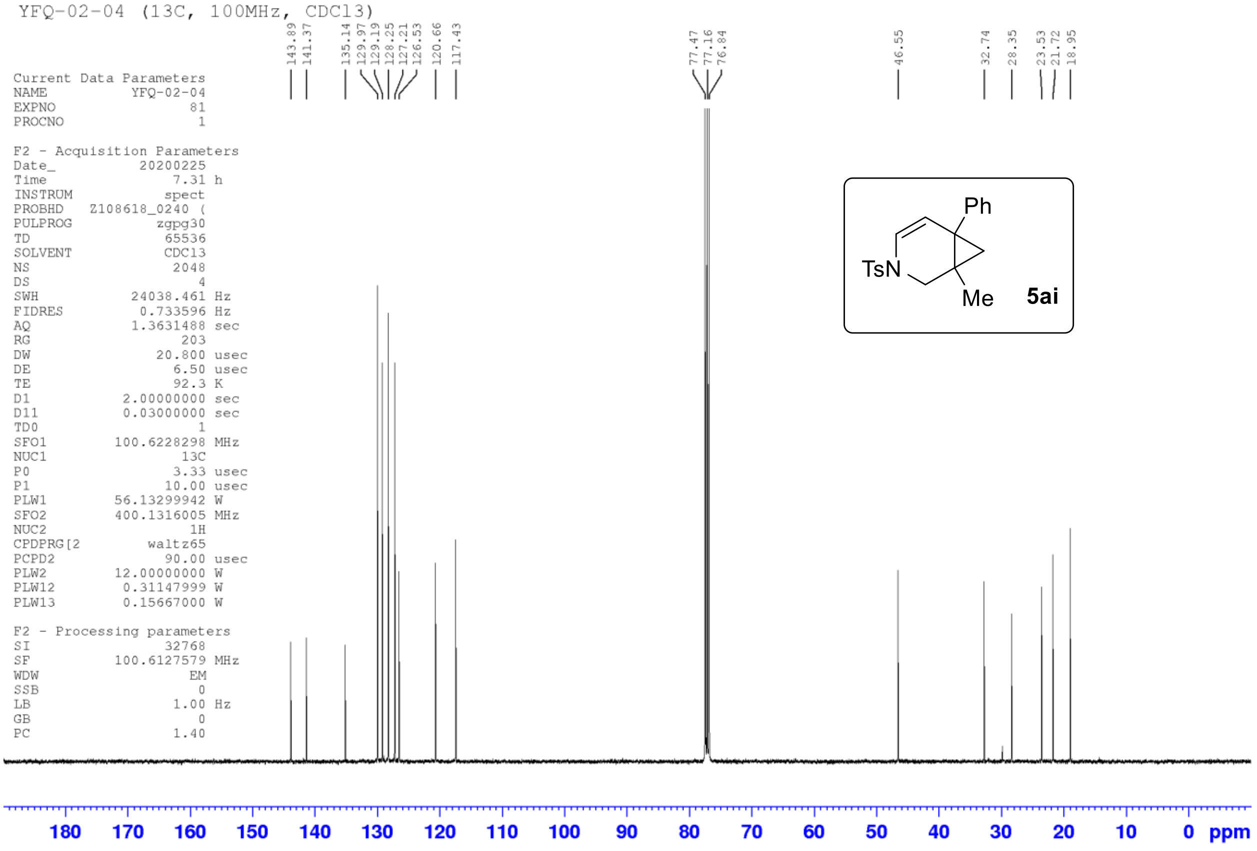
YFQ-02-04 (13C, 100MHz, CDCl3)
143.89 141.37 135.14 129.97 129.19 128.25 127.21 126.53 120.66 117.43
77.47 77.16 76.84
46.55
32.74 28.35 23.53 21.72 18.95
Current Data Parameters
NAME YFQ-02-04
EXPNO 81
PROCNO 1
F2 - Acquisition Parameters
Date_ 20200225
Time 7.31 h
INSTRUM spect
PROBHD Z108618_0240 (
PULPROG zgpg30
TD 65536
SOLVENT CDCl3
NS 2048
DS 4
SWH 24038.461 Hz
FIDRES 0.733596 Hz
AQ 1.3631488 sec
RG 203
DW 20.800 usec
DE 6.50 usec
TE 92.3 K
D1 2.00000000 sec
D11 0.03000000 sec
TD0 1
SFO1 100.6228298 MHz
NUC1 13C
P0 3.33 usec
P1 10.00 usec
PLW1 56.13299942 W
SFO2 400.1316005 MHz
NUC2 1H
CPDPRG[2 waltz65
PCPD2 90.00 usec
PLW2 12.00000000 W
PLW12 0.31147999 W
PLW13 0.15667000 W
F2 - Processing parameters
SI 32768
SF 100.6127579 MHz
WDW EM
SSB 0
LB 1.00 Hz
GB 0
PC 1.40
Ph
TsN
Me
5ai
180 170 160 150 140 130 120 110 100 90 80 70 60 50 40 30 20 10 0 ppm

References

(1) Achard, T.; Lepronier, A.; Gimbert, Y.; Clavier, H.; Giordano, L.; Tenaglia, A.; Buono, G. A Regio- and Diastereoselective Platinum-Catalyzed Tandem [2+1]/[3+2] Cycloaddition Sequence. *Angew. Chem., Int. Ed.* **2011**, *50*, 3552–3556.

(2) Marcyk, P. T.; Jefferies, L. R.; Abusalim, D. I.; Pink, M.; Baik,M.-H.; Cook, S. P. Stereoinversion of Unactivated Alcohols bytethered Sulfonamides. *Angew. Chem., Int. Ed*. **2019**, *58*, 1727−1731.

(3) Gansäuer, A.; Otte, M.; Shi, L. Radical Cyclizations Terminated by Ir-Catalyzed Hydrogen Atom Transfer. *J. Am. Chem. Soc.* **2010**, *133*, 416–417.

(4) Teller, H.; Corbet, M.; Mantilli, L.; Gopakumar, G.; Goddard, R.; Thiel, W.; Fürstner, A. One-Point Binding Ligands for Asymmetric Gold Catalysis: Phosphoramidites with a TADDOL-Related but Acyclic Backbone. *J. Am. Chem. Soc.* **2012**, *134*, 15331–15342.

(5) Ylagan, R. M. P.; Lee, E. J.; Negru, D. E.; Ricci, P.; Park, B.; Ryu, H.; Baik, M.; Evans, P. A. Enantioselective Rhodium-Catalyzed Pauson–Khand Reactions of 1,6-Chloroenynes with 1,1-Disubstituted Olefins. *Angew. Chem., Int. Ed.* **2023**, *62*, No. e202300211.

(6) Ohmura, T.; Sasaki, I.; Suginome, M. Catalytic Generation of Rhodium Silylenoid for Alkene–Alkyne–Silylene [2 + 2 + 1] Cycloaddition. *Org. Lett.* **2019**, *21*, 1649–1653.

(7) Koshikawa, T.; Satoh, M.; Masutomi, K.; Shibata, Y.; Tanaka, K. Gold-Catalyzed Enantioselective Intramolecular Annulation of Ene-Yne-Carbonyls via Benzopyrylium-Type Intermediates. *Eur. J. Org. Chem.* **2019**, *2019*, 1488–1492.

(8) Kitamura, T.; Sato, Y.; Mori, M. Effects of Substituents on the Multiple Bonds on Ring-Closing Metathesis of Enynes. *Adv. Synth. Catal*. **2002**, *344*, 678–693.

(9) Roberts, D. D.; McLaughlin, M. G. Regioselective Synthesis of Multifunctional Allylic Amines; Access to Ambiphilic Aziridine Scaffolds. *Org. Lett.* **2021**, *23*, 4463–4467.

(10) Ni, Z.; Giordano, L.; Tenaglia, A. Cyclobutene Formation in $PtCl_2$-Catalyzed Cycloisomerizations of Heteroatom-Tethered 1,6-Enynes. *Chem.- Eur. J.* **2014**, *20*, 11703–11706.

(11) Manavi, B.; Tejeneki, H. Z.; Rominger, F.; Armaghan, M.; Frank, W.; Bijanzadeh, H. R.; Balalaie, S. Copper(I)-Catalyzed Intramolecular Cyclization of O-Propargyloxy Diketopiperazines to Access Diverse Diazabicyclic and Spiro-Diketopiperazinochromanes. *Adv. Synth. Catal.* **2021**, *363*, 4190–4196.

(12) Chen, C.; Huang, Y.; Zhang, Z.; Dong, X.-Q.; Zhang, X. Cobalt-Catalyzed (*Z*)-Selective Semihydrogenation of Alkynes with Molecular Hydrogen. *Chem. Commun*. **2017**, *53*, 4612–4615.

(13) Morimoto, T.; Fuji, K.; Tsutsumi, K.; Kakiuchi, K. CO-Transfer Carbonylation Reactions. A Catalytic Pauson−Khand-Type Reaction of Enynes with Aldehydes as a Source of Carbon Monoxide. *J. Am. Chem. Soc.* **2002**, *124*, 3806–3807.

(14) Cabrera-Lobera, N.; Quirós, M. T.; Brennessel, W. W.; Neidig, M. L.; Buñuel, E.; Cárdenas, D. J. Atom-Economical Ni-Catalyzed Diborylative Cyclization of Enynes: Preparation of Unsymmetrical Diboronates. *Org. Lett.* **2019**, *21*, 6552–6556.

(15) Diao, Y.; Zuo, Z.; Wang, H.; Liu, J.; Luan, X. Palladium(0)-Catalyzed [2 + 2 + 1] Cyclization of 1,6-enynes with Vinyl Bromides: A Highly Diastereoselective Synthesis of Tetrahydro-1*H*-cyclopenta[*c*]furans Bearing Two Quaternary Carbon Centers. *Org. Biomol. Chem.* **2017**, *15*, 4601–4608.

(16) Dell'Isola, A.; McLachlan, M. M. W.; Neuman, B. W.; Al-Mullah, H. M. N.; Binks, A. W. D.; Elvidge, W.; Shankland, K.; Cobb, A. J. A. Synthesis and Antiviral Properties of Spirocyclic [1,2,3]-Triazolooxazine Nucleosides. *Chem.- Eur. J.* **2014**, *20*, 11685–11689.

(17) Guan, Z.; Zhu, S.; Ye, Y.; Li, X.; Liu, Y.; Wang, P.; Zhang, H.; Huang, Z.; Lei, A. Synthesis of Cyclopentene Derivatives via Electrochemically Induced Intermolecular Selective (3+2) Annulation. *Angew. Chem., Int. Ed.* **2022**, *61*, No. e202207059.

(18) Bahou, K. A.; Braddock, D. C.; Meyer, A. G.; Savage, G. P. Kinetic Benchmarking Reveals the Competence of Prenyl Groups in Ring-Closing Metathesis. *Org. Lett.* **2017**, *19*, 5332–5335.

(19) Kim, R.; Kwon, K.; Lee, H. A Free Radical Cyclization Catalyzed by Ruthenium Hydride Species. *Chem.- Asian. J.* **2021**, *16*, 3909–3913.

(20) Gao, P.; Yan, X.-B.; Tao, T.; Yang, F.; He, T.; Song, X.-R.; Liu,X.-Y.; Liang, Y.-M. Copper-Catalyzed Trifluoromethylation-Cyclization of Enynes: Highly Regioselective Construction of Trifluoromethylated Carbocycles and Heterocycles. *Chem. - Eur. J.* **2013**, *19*, 14420−14424.

(21) Ota, K.; Chatani, N. Rh(II)-Catalyzed Skeletal Reorganization of Enynes Involving Selective Cleavage of C–C Triple Bonds. *Chem. Commun.* **2008**, 2906–2907.

(22) Wang, Y.; Xu, L.; Yu, R.; Chen, J.; Yang, Z. $CoBr_2$–TMTU–zinc Catalysed-Pauson–Khand Reaction. *Chem. Commun.* **2012**, *48*, 8183–8185.

(23) Wang, X.-R.; Lu, F.-H.; Song, Y.; Lu, Z.-L. Intramolecular Pauson–Khand Reaction Catalyzed by Oxime-Derived Palladacycles. *Tetrahedron Lett.* **2012**, *53*, 589–592.

(24) Hamasaki, A.; Muto, A.; Haraguchi, S.; Liu, X.; Sakakibara, T.; Yokoyama, T.; Tokunaga, M. Cobalt Oxide Supported Gold Nanoparticles as a Stable and Readily-Prepared Precursor for the *in situ* Generation of Cobalt Carbonyl Like Species. *Tetrahedron Lett.* **2011**, *52*, 6869–6872.

(25) Mahamulkar, S. G.; Císařová, I.; Jahn, U. New Phosphine Ligand Architectures Lead to Efficient Gold Catalysts for Cycloisomerization Reactions at Very Low Loading. *Adv. Synth. Catal.* **2018**, *360*, 4215 –4224.

(26) Dotson, J. J.; van Dijk, L.; Timmerman, J. C.; Grosslight, S.; Walroth, R. C.; Gosselin, F.; Püntener, K.; Mack, K. A.; Sigman, M. S. Data-Driven Multi-Objective Optimization Tactics for Catalytic Asymmetric Reactions Using Bisphosphine Ligands. *J. Am. Chem. Soc.* **2023**, *145*, 110–121.

(27) Burrows, L. C.; Jesikiewicz, L. T.; Lu, G.; Geib, S. J.; Liu, P.; Brummond, K. M. Computationally Guided Catalyst Design in the Type I Dynamic Kinetic Asymmetric Pauson–Khand Reaction of Allenyl Acetates. *J. Am. Chem. Society*. **2017**, *139*, 15022–15032.

(28) Yang, P.; Zhang, Y.; Chen, M.; Zhao, Q.; Ren, Z.-H.; Guan, Z.-H. Rhodium-Catalyzed Enantioselective and Desymmetrizative Pauson–Khand Reaction: Access to Tricyclo[6.2.1.0^{4,11}]undecenes. *Org. Lett.* **2021**, *23*, 9241–9245.

(29) Burrows, L. C.; Jesikiewicz, L. T.; Liu, P.; Brummond, K. M. Mechanism and Origins of Enantioselectivity in the Rh(I)-Catalyzed Pauson–Khand Reaction: Comparison of Bidentate and Monodentate Chiral Ligands. *ACS Catal.* **2021**, *11*, 323–336.

(30) Jeong, N.; Sung, B. K.; Choi, Y. K. Rhodium(I)-Catalyzed Asymmetric Intramolecular Pauson−Khand-Type Reaction. *J. Am. Chem. Soc.* **2000**, *122*, 6771–6772.

(31) Speight, J. *Lange's Handbook of Chemistry, Seventeenth Edition*; McGraw-Hill Education, **2016**.

(32) Winget, P.; Dolney, D. M.; Giesen, D. J.; Cramer, C. J.; Truhlar,D. G. *Minnesota solvent descriptor database*. https://comp.chem.umn.edu/solvation/mnsddb.pdf (accessed 2023-11-08).

(33) Marcus, Y. The Properties of Organic Liquids That Are Relevant to Their Use as Solvating Solvents. *Chem. Soc. Rev.* **1993**, *22*, 409–416.

(34) Ogawa, A.; Curran, D. P. Benzotrifluoride: A Useful Alternative Solvent for Organic Reactions Currently Conducted in Dichloromethane and Related Solvents. *J. Org. Chem.* **1997**, *62*, 450–451.

(35) Abraham, M. H. Hydrogen bonding. 31. Construction of a Scale of Solute Effective or Summation Hydrogen-bond Basicity. *J. Phys. Org. Chem.* **1993**, *6*, 660–684.

(36) Black, A. W.; Bartlett, P. N. Selection and Characterisation of Weakly Coordinating Solvents for Semiconductor Electrodeposition. *Phys. Chem. Chem. Phys.* **2022**, *24*, 8093–8103.

(37) Tundo, P.; Selva, M. The Chemistry of Dimethyl Carbonate. *Acc. Chem. Res.* **2002**, *35*, 706–716.

(38) Kamlet, M. J.; Abboud, J.-L. M.; Abraham, M. H.; Taft, R.W. Linear Solvation Energy Relationships. 23. A Comprehensive Collection of the Solvatochromic Parameters, π*, α, and β, and Some Methods for Simplifying the Generalized Solvatochromic Equation. *J. Org. Chem.* **1983**, *48*, 2877–2887.

(39) Katritzky, A. R.; Fara, D. C.; Kuanar, M.; Hur, E.; Karelson, M. The Classification of Solvents by Combining Classical QSPR Methodology with Principal Component Analysis. *J. Phys. Chem. A* **2005**, *109*, 10323–10341.

(40) Alvarez, S. Coordinating Ability of Anions, Solvents, Amino Acids, and Gases towards Alkaline and Alkaline-Earth Elements, Transition Metals, and Lanthanides. *Chem.- Eur. J.* **2020**, *26*, 4350–4377.

(41) Kim, D. E.; Ratovelomanana-Vidal, V.; Jeong, N. 2,2′-Bis[bis(3,5-di-tert-butyl-4-methoxyphenyl)phosphino]-6,6′-dimethoxy-1,1′-biphenyl in Intramolecular Rhodium(I)-Catalyzed Asymmetric Pauson–Khand-Type Reactions. *Adv. Synth. Catal.* **2010**, *352*, 2032–2040.

(42) Soriano, E.; Ballesteros, P.; Marco-Contelles, J. A Theoretical Investigation on the Mechanism of the $PtCl_2$-Mediated Cycloisomerization of Heteroatom-Tethered 1,6-Enynes. *J. Org. Chem.* **2004**, *69*, 8018–8023.

(43) Hirsch, J. A. Table of Conformational Energies—1967. *Top. Stereochem.* **2007**, *1*, 199–222.

(44) Hansch, C.; Leo, A.; Taft, R. W. A Survey of Hammett Substituent Constants and Resonance and Field Parameters. *Chem. Rev.* **1991**, *91*, 165–195.
