# Supplementary material for: Systematic Parameter Determination Aimed at a Catalyst-Controlled Asymmetric Rh(I)-Catalyzed Pauson–Khand Reaction
Source: ACS Catal. 2024 Nov 5;14(22):17065–76. doi: 10.1021/acscatal.4c04490 (PMC11574763; doi:10.1021/acscatal.4c04490)
Supplement: Supplementary file 2 — cs4c04490_si_002.pdf [file cs4c04490_si_002.pdf]

Supporting Information for Computational Studies

Systematic Parameter Determination Aimed at a Catalyst-controlled Asymmetric Rh(I)-Catalyzed Pauson-Khand Reaction.

Yifan Qi, Luke T. Jesikiewicz, Grace E. Scofield, Peng Liu*, and Kay M. Brummond*

Department of Chemistry, University of Pittsburgh, Pittsburgh, Pennsylvania 15260, United States

Corresponding authors: kbrummon@pitt.edu; pengliu@pitt.edu

Table of Contents

Computational Details S-2

Temperature and quasi-harmonic approximation effects on enthalpy and Gibbs free energy values S-2

Distortion-Interaction Analysis of Key Transition State Isomers S-3

Summary of Natural Population Analysis (NPA) Charges S-4

Calculations of Sterimol Parameters for PKR Products S-6

References S-6

Cartesian Coordinates S-8

Computed IR information Using Spartan S-23

Xyz Coordinates (IR calculations) S-40

Computational Details

All DFT calculations of the oxidative cyclization pathways were performed using the Gaussian 16 software package.[1] Molecular geometries were optimized using the B3LYP functional[2-3] with Grimme's DFT-D3 dispersion correction[4] and the 6-31G(d) basis set for all non-metal atoms and LANL2DZ for rhodium in the gas phase. Single point energy calculations were performed using the M06 functional[5] and 6-311+G(d,p) basis set for all non-metal atoms and SDD basis set for rhodium with the SMD solvation model[6] in dichloroethane (DCE). The Gibbs free energy values for all optimized transition state and intermediate structures were computed at the experimental temperature (353.15 K). Truhlar's quasi-harmonic approximations[7] were applied for entropy calculations using 100 cm^{-1} as the frequency cutoff. Temperature and quasi-harmonic corrections were applied to the computed Gibbs free energies using GoodVibes (v3.0.1)[8] software package. Natural population analysis and charges were computed using the NBO 7.0 suite[9]. The 3D images of optimized structures were prepared using CYLView.[10]

Temperature and quasi-harmonic approximation effects on enthalpy and Gibbs free energy values

Table S1: Energy values computed at different temperatures and with and without quasi-harmonic (qH) approximation

Structure name	H @ 298.15 K (Hartree)	G @ 298.15 K (Hartree)	H @ 353.15 K (Hartree)	G @ 353.15 K w/ qH correction (Hartree)
**6a-Rh-L1**	-3066.239945	-3066.381158	-3066.012762	-3066.186316
**TS1-R**	-3066.226058	-3066.363176	-3066.005868	-3066.175722
**TS1-S**	-3066.21896	-3066.35731	-3065.999104	-3066.169104
**6a-Rh-L3**	-3380.583164	-3380.758023	-3380.14099	-3380.351363
**TS2-R**	-3380.571089	-3380.743232	-3380.135143	-3380.342236
**TS2-S**	-3380.564565	-3380.736596	-3380.129408	-3380.336551

Distortion-Interaction Analysis of Key Transition State Isomers

Table S2: Distortion-Interaction Analysis of Key Oxidative Cyclization Transition States

	**TS1-R** *(major)*	**TS1-S** *(minor)*	**TS2-R** *(major)*	**TS2-S** *(minor)*
$\Delta G^{‡}$ (kcal/mol)	6.7	10.8	5.7	9.3
$\Delta H^{‡}$ (kcal/mol)	4.3	8.6	3.8	7.2
$\Delta E_{\text{dist (Rh-lig)}}$ (kcal/mol)	-12.0	-14.0	-13.3	-14.8
$\Delta E_{\text{dist (substrate)}}$ (kcal/mol)	50.4	53.0	49.9	52.3
ΔE_{int} (kcal/mol)	-32.6	-29.0	-31.4	-28.8

The distortion energies (ΔE_{dist}) were calculated from the energy difference between the Rh-ligand complex/enyne substrate at their transition state geometry and their geometry in the Rh-substrate π complexes **6a-Rh-L1** and **6a-Rh-L3**. The interaction energies (ΔE_{int}) were calculated from the change of interaction energies between the Rh-ligand complex and the enyne substrate from the Rh-π complexes **6a-Rh-L1** and **6a-Rh-L3** to the transition state structures. The negative values of ΔE_{int} indicate the Rh-ligand complex and the enyne substrate interact more strongly in the TS than in the Rh-substrate π complexes. It was found that transition states leading to the major experimentally observed products (**TS1-R** and **TS2-R**) experience a higher degree of stabilizing interaction (-32.6 and -31.4 kcal/mol for **TS1-R** and **TS2-R** vs. -29.0 and -28.8 kcal/mol for **TS1-S** and **TS2-S**) and less substrate distortion (50.4 and 49.9 kcal/mol vs. 53.0 and 52.3 kcal/mol) than the TS isomers leading to the minor products (**TS1-S** and **TS2-S**). All the calculated transition states had a negative value for $\Delta E_{\text{dist (Rh-lig)}}$, suggesting that there is significant steric strain on the ligand in the square planar π-complexes (**6a-Rh-L1** and **6a-Rh-L3**) that is relieved in the twisted tetrahedral transition state geometry.

Summary of Natural Population Analysis (NPA) Charges

Table S3: Computed NPA partial charges of (*R*)-BINAP (L1)-supported structures

6a-Rh-L1

Atom	Atom ID	NPA Charge
**Rh**	**1**	**0.00094**
P	2	1.26543
P	36	1.06241
C	80	0.02094
C	81	-0.07638
C	82	-0.14265
C	83	-0.07401
C	84	-0.17924
C	85	-0.16821
H	86	0.20166
O	87	-0.61192
C	88	-0.21335
H	89	0.22904
C	90	-0.21488
H	91	0.22296
C	92	-0.06206
C	93	-0.19695
H	94	0.22451
H	95	0.22449
C	96	0.05195
H	97	0.19497
H	98	0.21591
H	99	0.22286
C	100	-0.46547
H	101	0.22604
H	102	0.22428
H	103	0.22016
C	104	-0.63907
H	105	0.23044
H	106	0.2252
H	107	0.23466
**Sum of enyne NPA charge**		**0.12588**
Δenyne NPA charge (with respect to **6a-Rh-L1**)		0.000

TS1-R

Atom	Atom ID	NPA Charge
**Rh**	**1**	**0.05133**
P	2	1.23701
P	36	1.23449
C	80	-0.05669
C	81	-0.08362
C	82	-0.10495
C	83	-0.06489
C	84	-0.19428
C	85	-0.18972
H	86	0.19769
O	87	-0.59892
C	88	-0.20787
H	89	0.22225
C	90	-0.20964
H	91	0.21942
C	92	-0.03822
C	93	-0.20954
H	94	0.22176
H	95	0.22203
C	96	-0.03375
H	97	0.18394
H	98	0.22124
H	99	0.22145
C	100	-0.52994
H	101	0.22471
H	102	0.22676
H	103	0.22606
C	104	-0.61415
H	105	0.22852
H	106	0.22554
H	107	0.22501
**Sum of enyne NPA charge**		**-0.0698**
Δenyne NPA charge (with respect to **6a-Rh-L1**)		-0.19568

TS1-S

Atom	Atom ID	NPA Charge
**Rh**	**8**	**0.06112**
P	26	1.30376
P	60	1.21875
C	1	-0.06757
C	2	-0.13106
C	3	-0.06356
C	4	-0.04137
C	5	-0.05247
C	6	-0.46003
H	7	0.22178
H	9	0.22607
C	10	-0.08629
C	11	-0.21357
C	12	-0.19907
C	13	-0.20457
H	14	0.21765
C	15	-0.20399
H	16	0.22097
C	17	-0.21998
H	18	0.22153
H	19	0.22127
H	20	0.22047
O	21	-0.60175
H	22	0.19125
H	23	0.21969
H	24	0.20416
H	25	0.22035
C	104	-0.60496
H	105	0.22447
H	106	0.22829
H	107	0.22492
**Sum of enyne NPA charge**		**-0.08737**
Δenyne NPA charge (with respect to **6a-Rh-L1**)		-0.21325

Table S4: Computed NPA partial charges of (*R*)-DM-BINAP-supported structures

6a-Rh-L3

Atom	Atom ID	NPA Charge
**Rh**	**1**	**0.02513**
P	2	1.28428
P	32	1.05541
C	72	0.01217
C	73	-0.08275
C	74	-0.1417
C	75	-0.0761
C	76	-0.17862
C	77	-0.17405
H	78	0.1985
O	79	-0.61291
C	80	-0.21251
H	81	0.22924
C	82	-0.21523
H	83	0.22152
C	84	-0.06249
C	85	-0.20254
H	86	0.22398
H	87	0.22386
C	88	0.0449
H	89	0.19488
H	90	0.21609
H	91	0.22237
C	92	-0.46591
H	93	0.22362
H	94	0.22621
H	95	0.22201
C	96	-0.64672
H	97	0.23007
H	98	0.22651
H	99	0.2379
**Sum of enyne NPA charge**		**0.0823**
Δenyne NPA charge (with respect to **6a-Rh-L3**)		0.0

TS2-R

Atom	Atom ID	NPA Charge
**Rh**	**1**	**0.05713**
P	2	1.2799
P	32	1.21976
C	70	-0.06095
C	71	-0.09061
C	72	-0.1023
C	73	-0.06523
C	74	-0.19431
C	75	-0.18844
H	76	0.19641
O	77	-0.59916
C	78	-0.21059
H	79	0.22224
C	80	-0.21171
H	81	0.21732
C	82	-0.03853
C	83	-0.21059
C	84	-0.0364
H	85	0.18274
H	86	0.22039
H	87	0.22117
C	88	-0.52948
H	89	0.22315
H	90	0.22819
H	91	0.22545
C	92	-0.61414
H	93	0.22758
H	94	0.22707
H	95	0.22407
H	112	0.22117
H	113	0.22147
**Sum of enyne NPA charge**		**-0.09402**
Δenyne NPA charge (with respect to **6a-Rh-L3**)		-0.17632

TS2-S

Atom	Atom ID	NPA Charge
**Rh**	**8**	**0.0668**
P	24	1.27747
P	54	1.22674
C	1	-0.06697
C	2	-0.13642
C	3	-0.06583
C	4	-0.04176
C	5	-0.05459
C	6	-0.46213
H	7	0.22264
H	9	0.22506
C	10	-0.08544
C	11	-0.21074
C	12	-0.1999
C	13	-0.20464
H	14	0.21514
C	15	-0.20391
H	16	0.22102
C	17	-0.22265
H	18	0.22009
O	19	-0.60299
H	20	0.19219
H	21	0.21838
H	22	0.20456
H	23	0.21893
C	94	-0.60523
H	95	0.22466
H	96	0.22752
H	97	0.22457
H	122	0.22062
H	123	0.22108
**Sum of enyne NPA charge**		**-0.10674**
Δenyne NPA charge (with respect to **6a-Rh-L3**)		-0.18904

Calculations of Sterimol Parameters for PKR Products

Conformational sampling for **5a-5d** was performed using the Conformer-Rotamer Ensemble Sampling Tool (CREST) program[13] (version 2.12) that uses the semiempirical tight-binding method GFN2-xTB[14] (xTB version 6.6.0) to perform metadynamics sampling with genetic z-matrix crossing (iMTD-GC). In the CREST/GFN2-xTB conformational sampling of each structure, the conformers were filtered using a 0.5 Å root-mean-square-deviation (RMSD) threshold and an energy window of 6 kcal/mol. All conformers were fully optimized using the M06-2X functional[5] and the 6-31G(d) basis set, and duplicate structures were removed using the GoodVibes (v. 3.2)[8] software package. Sterimol L, B_1, and B_5 values[15] were calculated using the Morfeus package.[16] The dummy atom was assigned as hydrogen (H_a, *trans* to Me group, or H_b, *cis* to Me group; see also Figure **7** in the main text) with Bondi radii (r(H) = 1.20 Å). File format conversion was performed using OpenBabel (version 3.1.1).[17]

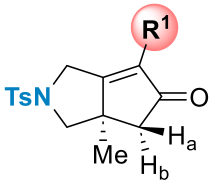

5a-d

Table S5: Sterimol L, B_1, and B_5 values for 5a-5d

	H_a or H_b as the dummy atom	Sterimol L	Sterimol B_1	Sterimol B_5
**5a**	a	5.43	2.08	8.58
	b	10.57	3.53	6.75
**5b**	a	5.15	2.07	8.19
	b	10.81	3.16	6.35
**5c**	a	-	-	-
	b	10.38	3.24	6.48
**5d**	a	5.23	2.08	11.89
	b	11.71	2.06	7.55

References

(1) M. J. Frisch, G. W. Trucks, H. B. Schlegel, G. E. S.; M. A. Robb, J. R. Cheeseman, G. Scalmani, V. B.; G. A. Petersson, H. Nakatsuji, X. Li, M. Caricato, A. V. M.; J. Bloino, B. G. Janesko, R.

Gomperts, B. Mennucci, H. P. H.; J. V. Ortiz, A. F. Izmaylov, J. L. Sonnenberg, D. W.-Y.; F. Ding, F. Lipparini, F. Egidi, J. Goings, B. Peng, A. P.; T. Henderson, D. Ranasinghe, V. G. Zakrzewski, J. Gao, N. R.; G. Zheng, W. Liang, M. Hada, M. Ehara, K. Toyota, R. F.; J. Hasegawa, M. Ishida, T. Nakajima, Y. Honda, O. Kitao, H. N.; T. Vreven, K. Throssell, J. A. Montgomery, Jr., J. E. P.; F. Ogliaro, M. J. Bearpark, J. J. Heyd, E. N. Brothers, K. N. K.; V. N. Staroverov, T. A. Keith, R. Kobayashi, J. N.; K. Raghavachari, A. P. Rendell, J. C. Burant, S. S. I.; J. Tomasi, M. Cossi, J. M. Millam, M. Klene, C. Adamo, R. C.; J. W. Ochterski, R. L. Martin, K. Morokuma, O. F.; J. B. Foresman, D. J. Fox. Gaussian 16. Gaussian, Inc.: Wallingford, CT **2016**.

(2) Becke, A. D. Density-Functional Thermochemistry. III. The Role of Exact Exchange. *J. Chem. Phys.* **1993**, *98* (7), 5648–5652. https://doi.org/10.1063/1.464913.

(3) Lee, C.; Yang, W.; Parr, R. G. Development of the Colle-Salvetti Correlation-Energy Formula into a Functional of the Electron Density. *Phys. Rev. B* **1988**, *37* (2), 785–789. https://doi.org/10.1103/PhysRevB.37.785.

(4) Grimme, S.; Antony, J.; Ehrlich, S.; Krieg, H. A Consistent and Accurate Ab Initio Parametrization of Density Functional Dispersion Correction (DFT-D) for the 94 Elements H-Pu. *J. Chem. Phys.* **2010**, *132* (15). https://doi.org/10.1063/1.3382344.

(5) Zhao, Y.; Truhlar, D. G.; Zhao, Y.; Truhlar, · D G. The M06 Suite of Density Functionals for Main Group Thermochemistry, Thermochemical Kinetics, Noncovalent Interactions, Excited States, and Transition Elements: Two New Functionals and Systematic Testing of Four M06-Class Functionals and 12 Other Functionals and Inorganometallic Chemistry and for Noncovalent Interactions. *Theor. Chem. Acc.* **2008**, *120*, 215–241. https://doi.org/10.1007/s00214-007-0310-x.

(6) Marenich, A.V.; Cramer, C.J.; Truhlar, D G. Universal Solvation Model Based on Solute Electron Density and on a Continuum Model of the Solvent Defined by the Bulk Dielectric Constant and Atomic Surface Tensions. *J. Phys. Chem. B* **2009**, *113* (18), 6378–6396. https://doi.org/10.1021/jp810292n.

(7) Ribeiro, R. F.; Marenich, A. V.; Cramer, C. J.; Truhlar, D. G. Use of Solution-Phase Vibrational Frequencies in Continuum Models for the Free Energy of Solvation. *J. Phys. Chem. B* **2011**, *115* (49), 14556–14562. https://doi.org/10.1021/jp205508z.

(8) Luchini, G.S.; Paton, R. S.; Alegre-requena, J. V; Funes-ardoiz, I. GoodVibes : Automated Thermochemistry for Heterogeneous Computational Chemistry Data [Version 1 ; Peer Review : 2 Approved with Reservations] Guilian Luchini. *F1000Research* **2020**, *9*, 291. https://doi.org/10.12688/f1000research.22758.1

(9) NBO 7.0, E. D. Glendening, J, K. Badenhoop, A. E. Reed, J. E. Carpenter, J. A. Bohmann, C. M. Morales, P. Karafiloglou, C. R. Landis, & F. Weinhold, Theoretical Chemistry Institute, University of Wisconsin, Madison, **2018**.

(10) CYLview, 1.0b; Legault, C. Y., Université de Sherbrooke, **2009** http://www.cylview.org.

(11) Burrows, L.; Jesikiewicz, L.; Liu, P.; Brummond, K. Mechanism and Origins of Enantioselectivity in the Rh(I)-Catalyzed Pauson–Khand Reaction: Comparison of Bidentate and Monodentate Chiral Ligands. *ACS Catal.* **2021**, *11* (1), 323–336. https://doi.org/10.1021/acscatal.0c03774.

(12) Deihl, E.; Jesikiewicz, L.; Newman, L.; Liu, P.; Brummond, K. Rh(I)-Catalyzed Allenic Pauson–Khand Reaction to Access the Thapsigargin Core: Influence of Furan and Allenyl Chloroacetate Groups on Enantioselectivity. *Org. Lett.* **2022**, *24* (4), 995–999. https://doi.org/10.1021/acs.orglett.1c03951.

(13) Pracht, P.; Bohle, F.; Grimme, S. Automated Exploration of the Low-Energy Chemical Space with Fast Quantum Chemical Methods. *Phys. Chem. Chem. Phys.* **2020**, *22* (14), 7169–7192. https://doi.org/10.1039/C9CP06869D.

(14) Bannwarth, C.; Ehlert, S.; Grimme, S. GFN2-xTB—An Accurate and Broadly Parametrized Self-Consistent Tight-Binding Quantum Chemical Method with Multipole Electrostatics and Density-Dependent Dispersion Contributions. *J. Chem. Theory Comput.* **2019**, *15* (3), 1652–1671. https://doi.org/10.1021/acs.jctc.8b01176.

(15) Verloop, A.; Hoogenstraaten, W.; Tipker, J. Development and Application of New Steric Substituent Parameters in Drug Design. In *Drug Design*; Elsevier, 1976; pp 165–207. https://doi.org/10.1016/B978-0-12-060307-7.50010-9.

(16) *Morfeus*; Kjell Jorner, 2021. https://digital-chemistry-laboratory.github.io/morfeus/index.html (accessed 2024-07-20).

(17) O’Boyle, N. M.; Banck, M.; James, C. A.; Morley, C.; Vandermeersch, T.; Hutchison, G. R. Open Babel: An Open Chemical Toolbox. *J. Cheminform.* **2011**, *3* , 33. https://doi.org/10.1186/1758-2946-3-33.

Cartesian Coordinates

6a-Rh-L1

```
B3LYP-D3 SCF energy:            -3067.15801381 a.u.
B3LYP-D3 enthalpy:              -3066.239945 a.u.
B3LYP-D3 Gibbs free energy:     -3066.381158 a.u.
M06 SCF energy in solution:     -3066.95105945 a.u.
M06 enthalpy:                   -3066.032991 a.u.
M06 Gibbs free energy:          -3066.174204 a.u.

Cartesian coordinates
```

ATOM	X	Y	Z
Rh	1.528275	-0.680197	-0.310697
P	0.537512	-0.099095	1.683749
C	-0.463397	1.429939	1.426508
C	-0.534905	-1.437091	2.315919
C	1.706666	0.195868	3.082828
C	-1.550639	1.425771	0.543650
C	0.055480	2.671331	1.886092
C	-0.191949	-2.765647	2.023734
C	-1.638708	-1.169883	3.135901
C	2.695436	1.198901	3.026058
C	1.678964	-0.652245	4.203579
C	-2.453811	0.236900	0.371171
C	-1.970662	2.658991	-0.055844
C	-0.396600	3.866495	1.381073
H	0.818724	2.681766	2.652154
C	-0.962500	-3.814825	2.526638
H	0.678486	-2.975090	1.405443
C	-2.416144	-2.220061	3.624021
H	-1.905662	-0.145738	3.374908
C	3.610279	1.355362	4.065964
H	2.760286	1.862783	2.174317
C	2.605568	-0.497919	5.236968
H	0.937050	-1.437704	4.280374
C	-2.212934	-0.851603	-0.461436
C	-3.670628	0.288762	1.143967
C	-1.359967	3.893039	0.344924
C	-2.978328	2.701135	-1.061113
H	0.013619	4.802530	1.750027
C	-2.081339	-3.541573	3.318245
H	-0.691033	-4.842039	2.299696
H	-3.286915	-2.002373	4.235093
C	3.572864	0.504490	5.173135
H	4.357223	2.141710	4.005074
H	2.564245	-1.164557	6.093409
C	-3.204894	-1.865055	-0.598450
P	-0.624133	-0.989440	-1.399017
C	-4.633515	-0.763402	1.019611
C	-3.944356	1.330771	2.077852
C	-1.750111	5.104392	-0.287614
H	-3.453782	1.778126	-1.369413
C	-3.319612	3.886918	-1.668979
H	-2.687970	-4.357483	3.700301
H	4.291786	0.623573	5.978476
C	-4.376011	-1.819742	0.113355
H	-3.041490	-2.688209	-1.281107
C	-0.735398	-2.638432	-2.211451
C	-0.888496	0.213680	-2.765985
C	-5.816141	-0.736609	1.805743
H	-3.233821	2.138311	2.205578
C	-5.093406	1.322383	2.837438
H	-1.280691	6.032308	0.028985
C	-2.702335	5.102150	-1.280867
H	-4.069661	3.892958	-2.454314
H	-5.117542	-2.604981	-0.009366
C	-0.614426	-2.784694	-3.600651
C	-0.831896	-3.791150	-1.410555

C	-2.139243	0.283455	-3.404455
C	0.134346	1.079838	-3.170282
H	-6.535522	-1.543194	1.689933
C	-6.044194	0.282686	2.700836
H	-5.273333	2.124762	3.547062
H	-2.989393	6.030969	-1.765056
C	-0.581888	-4.058481	-4.176147
H	-0.548814	-1.909200	-4.238695
C	-0.819994	-5.058018	-1.989873
H	-0.928708	-3.697369	-0.333631
C	-2.357622	1.210642	-4.421327
H	-2.942938	-0.381221	-3.100391
C	-0.091049	2.018188	-4.179724
H	1.102129	1.034242	-2.686444
H	-6.948746	0.293903	3.301707
C	-0.685828	-5.195954	-3.375136
H	-0.482785	-4.156848	-5.253312
H	-0.906110	-5.938736	-1.359746
C	-1.336159	2.085573	-4.805174
H	-3.327069	1.256322	-4.909386
H	0.706960	2.696394	-4.467870
H	-0.665693	-6.184086	-3.825243
H	-1.513177	2.815627	-5.589749
C	3.067460	0.797332	-0.110037
C	3.503018	-0.248845	0.431832
C	3.070426	2.160339	-0.579142
C	4.478596	-1.197051	0.999715
C	1.883775	2.843432	-0.896329
C	4.306310	2.825820	-0.723121
H	5.458250	-0.697886	1.083019
O	4.602800	-2.378249	0.208444
C	1.929415	4.157695	-1.356472
H	0.932426	2.341900	-0.776087
C	4.343403	4.140188	-1.179992
H	5.225873	2.302552	-0.477217
C	4.579663	-2.115944	-1.191580
C	3.156487	4.808806	-1.501102
H	1.000531	4.668564	-1.593401
H	5.299426	4.643952	-1.289395
C	3.183819	-2.261224	-1.779332
H	5.012705	-1.135005	-1.424885
H	5.215554	-2.879988	-1.655672
H	3.191977	5.832955	-1.861258
C	2.627485	-1.292303	-2.557068
H	3.156822	-0.363224	-2.754536
H	1.751553	-1.494277	-3.164016
H	4.171448	-1.518892	1.999498
C	2.582178	-3.628085	-1.599130
H	2.410054	-3.833855	-0.536484
H	1.649885	-3.746314	-2.145810
H	3.293104	-4.387667	-1.950075

TS1-R

B3LYP-D3 SCF energy: -3067.14194797 a.u.
B3LYP-D3 enthalpy: -3066.226058 a.u.

```
B3LYP-D3 Gibbs free energy:       -3066.363176 a.u.
M06 SCF energy in solution:       -3066.94175176 a.u.
M06 enthalpy:                     -3066.025862 a.u.
M06 Gibbs free energy:            -3066.162980 a.u.
Imaginary frequency:               -237.4674 cm-1
```

Cartesian coordinates

ATOM	X	Y	Z
Rh	1.241116	-1.032990	-0.223362
P	0.534190	1.055800	-0.860972
C	-1.118082	0.807668	-1.648355
C	0.317983	2.462677	0.283886
C	1.661543	1.646858	-2.193773
C	-2.260488	0.630362	-0.864332
C	-1.189348	0.637781	-3.058896
C	0.738763	2.346741	1.612785
C	-0.304418	3.647694	-0.143622
C	2.206102	0.693486	-3.074632
C	2.069049	2.983235	-2.309654
C	-2.251823	0.850637	0.621746
C	-3.494101	0.241516	-1.486578
C	-2.370277	0.291893	-3.668878
H	-0.306121	0.799435	-3.665300
C	0.561680	3.406171	2.501132
H	1.190031	1.421634	1.947708
C	-0.478434	4.705975	0.745963
H	-0.676802	3.732242	-1.160741
C	3.128059	1.069163	-4.050125
H	1.915186	-0.349362	-2.984830
C	2.993391	3.357310	-3.288184
H	1.697760	3.732975	-1.621902
C	-1.665161	-0.059824	1.502071
C	-2.887928	2.027843	1.137993
C	-3.546674	0.074064	-2.909096
C	-4.676098	-0.014334	-0.735983
H	-2.412142	0.180845	-4.749502
C	-0.044801	4.586867	2.068974
H	0.889630	3.304494	3.531408
H	-0.972875	5.613303	0.413134
C	3.526863	2.405384	-4.157179
H	3.537072	0.320734	-4.722994
H	3.303953	4.395667	-3.359654
C	-1.721870	0.165489	2.903532
P	-0.760618	-1.515323	0.823411
C	-2.896651	2.261137	2.551766
C	-3.495207	2.999348	0.293032
C	-4.765854	-0.315541	-3.523821
H	-4.651267	0.086240	0.342462
C	-5.840009	-0.401841	-1.360134
H	-0.191225	5.409364	2.762803
H	4.250954	2.698998	-4.911553
C	-2.314976	1.296475	3.410058
H	-1.298943	-0.561319	3.586060
C	-0.160513	-2.499874	2.252289
C	-2.099039	-2.483113	0.033225
C	-3.491645	3.445793	3.061544
H	-3.497803	2.842570	-0.779110

C	-4.069972	4.134633	0.818010
H	-4.791579	-0.430456	-4.604425
C	-5.891177	-0.549060	-2.767301
H	-6.728339	-0.599368	-0.767298
H	-2.346286	1.459268	4.484360
C	-0.479517	-3.855849	2.413074
C	0.791788	-1.916031	3.111178
C	-3.202485	-2.899314	0.797946
C	-2.089659	-2.714132	-1.347125
H	-3.487166	3.610214	4.135952
C	-4.066859	4.365252	2.214853
H	-4.528176	4.862633	0.154730
H	-6.819295	-0.848746	-3.245178
C	0.131806	-4.608300	3.419536
H	-1.198240	-4.331434	1.754509
C	1.388936	-2.667302	4.121094
H	1.073665	-0.875610	2.977714
C	-4.264573	-3.564167	0.188669
H	-3.232249	-2.698715	1.865631
C	-3.161618	-3.367946	-1.955778
H	-1.267143	-2.342815	-1.948063
H	-4.522653	5.266534	2.614240
C	1.062154	-4.018272	4.275618
H	-0.122693	-5.658211	3.531589
H	2.116753	-2.202711	4.780110
C	-4.245251	-3.798168	-1.189533
H	-5.112388	-3.887610	0.785545
H	-3.156055	-3.524964	-3.030106
H	1.534312	-4.606555	5.056816
H	-5.080710	-4.303048	-1.665496
C	3.115026	-0.296340	0.203735
C	3.508572	-1.362754	-0.423180
C	3.744437	0.924170	0.694851
C	4.765166	-1.677724	-1.198664
C	4.334498	1.839844	-0.192844
C	3.794836	1.178837	2.076169
H	4.749569	-1.069648	-2.120194
O	4.824320	-3.048907	-1.514514
C	4.934312	3.000358	0.295531
H	4.316319	1.642166	-1.258789
C	4.408628	2.332150	2.558578
H	3.351241	0.462704	2.762949
C	3.509481	-3.458022	-1.809863
C	4.970235	3.252468	1.668513
H	5.379149	3.705253	-0.401165
H	4.447297	2.515499	3.628620
C	2.588160	-3.101608	-0.635649
H	3.139263	-2.992287	-2.739043
H	3.526662	-4.544448	-1.937831
H	5.440193	4.156393	2.044743
C	1.174508	-3.061532	-0.959124
H	0.915188	-3.032300	-2.017261
H	0.526413	-3.706460	-0.370816
H	5.654101	-1.411700	-0.618947
C	2.979778	-3.796645	0.665311
H	2.764640	-4.868676	0.567975
H	2.417323	-3.411008	1.516532

H	4.047620	-3.682577	0.863294

TS1-S

B3LYP-D3 SCF energy: -3067.13493611 a.u.
B3LYP-D3 enthalpy: -3066.218960 a.u.
B3LYP-D3 Gibbs free energy: -3066.357310 a.u.
M06 SCF energy in solution: -3066.93507314 a.u.
M06 enthalpy: -3066.019097 a.u.
M06 Gibbs free energy: -3066.157447 a.u.
Imaginary frequency: -199.0735 cm-1

Cartesian coordinates

ATOM	X	Y	Z
C	-3.634136	-1.063163	0.003090
C	-3.152608	0.139190	-0.105044
C	-4.870287	-1.756108	-0.532083
C	-3.680905	-3.575181	0.204149
C	-2.949491	-2.560902	1.079383
C	-1.509347	-2.771037	1.227142
H	-1.078770	-3.576515	0.633441
Rh	-1.380290	-0.807163	0.425518
H	-1.129093	-2.749701	2.246664
C	-3.629471	1.426198	-0.595203
C	-3.904826	1.596629	-1.963561
C	-3.781706	2.517682	0.274681
C	-4.311831	2.838123	-2.450337
H	-3.784032	0.755632	-2.642046
C	-4.194004	3.754564	-0.217507
H	-3.567593	2.390113	1.330416
C	-4.452867	3.922590	-1.580227
H	-4.521343	2.958195	-3.509728
H	-4.308870	4.591933	0.465123
H	-4.766699	4.889881	-1.961257
O	-4.977736	-3.079162	-0.056964
H	-3.113104	-3.762784	-0.719996
H	-3.806555	-4.516882	0.747418
H	-4.782977	-1.748760	-1.630733
H	-5.781423	-1.216321	-0.254297
P	0.418551	-1.632329	-0.695461
C	1.255344	-0.281307	-1.631027
C	1.786484	-2.561583	0.083574
C	-0.356454	-2.765992	-1.922085
C	2.087255	0.619358	-0.960453
C	0.965410	-0.092631	-3.010026
C	1.817583	-2.758828	1.469212
C	2.857686	-3.014497	-0.704786
C	-1.472121	-2.305636	-2.649539
C	0.023339	-4.110610	-2.042926
C	2.461591	0.435345	0.482925
C	2.626336	1.746408	-1.666667
C	1.483359	0.977290	-3.698334
H	0.346181	-0.808821	-3.535528
C	2.900582	-3.408251	2.060594
H	1.020746	-2.360205	2.085156
C	3.933674	-3.671181	-0.110821

H	2.857106	-2.839965	-1.777391
C	-2.171390	-3.165419	-3.494302
H	-1.802909	-1.277075	-2.539528
C	-0.688933	-4.971757	-2.882737
H	0.866338	-4.495382	-1.479949
C	1.555118	0.676929	1.517769
C	3.800498	0.019007	0.790762
C	2.315432	1.925619	-3.054177
C	3.457201	2.714642	-1.035011
H	1.261854	1.101686	-4.755270
C	3.957380	-3.867116	1.272557
H	2.926288	-3.539650	3.138257
H	4.761726	-4.013008	-0.724416
C	-1.782717	-4.503581	-3.611084
H	-3.024974	-2.794101	-4.054359
H	-0.384611	-6.011198	-2.963842
C	1.968337	0.532239	2.869777
P	-0.214136	1.047430	1.123817
C	4.193313	-0.135624	2.160195
C	4.764028	-0.263839	-0.218538
C	2.847042	3.041580	-3.752894
H	3.691211	2.606441	0.017169
C	3.958374	3.785447	-1.739570
H	4.804912	-4.363855	1.735118
H	-2.333321	-5.176085	-4.262143
C	3.248282	0.139137	3.178857
H	1.273535	0.748451	3.672210
C	-1.014486	1.528935	2.708165
C	-0.109679	2.558042	0.099385
C	5.514931	-0.555340	2.467262
H	4.485243	-0.164563	-1.260789
C	6.038758	-0.665559	0.110824
H	2.603812	3.161583	-4.805439
C	3.654480	3.952773	-3.111989
H	4.589807	4.512392	-1.237048
H	3.550414	0.041520	4.218598
C	-1.488156	2.824806	2.958283
C	-1.252964	0.521638	3.662482
C	0.603024	3.677508	0.560833
C	-0.677335	2.580161	-1.178800
H	5.796840	-0.666328	3.511178
C	6.421961	-0.813625	1.465458
H	6.757400	-0.874808	-0.676336
H	4.059366	4.801838	-3.654889
C	-2.180563	3.105411	4.139427
H	-1.337457	3.613061	2.229713
C	-1.935320	0.807452	4.843265
H	-0.901767	-0.489863	3.476353
C	0.716368	4.811585	-0.241069
H	1.079254	3.656750	1.537550
C	-0.560975	3.715526	-1.978851
H	-1.189371	1.704048	-1.553348
H	7.431863	-1.129942	1.709652
C	-2.404926	2.102463	5.082938
H	-2.545466	4.112777	4.318171
H	-2.105360	0.020553	5.572601
C	0.132280	4.832477	-1.510887

H	1.272474	5.672585	0.117952
H	-1.008486	3.721006	-2.967823
H	-2.943648	2.325638	5.999096
H	0.230065	5.714316	-2.137253
C	-3.679323	-2.235432	2.387578
H	-3.210749	-1.392934	2.901932
H	-3.628725	-3.116490	3.040400
H	-4.732217	-2.001402	2.221133

6a-Rh-L3

B3LYP-D3 SCF energy: -3381.73697890 a.u.
B3LYP-D3 enthalpy: -3380.583164 a.u.
B3LYP-D3 Gibbs free energy: -3380.758023 a.u.
M06 SCF energy in solution: -3381.31944757 a.u.
M06 enthalpy: -3380.165633 a.u.
M06 Gibbs free energy: -3380.340492 a.u.

Cartesian coordinates

ATOM	X	Y	Z
Rh	0.948116	0.015473	-1.390333
P	1.454606	-0.580028	0.760611
C	0.712703	0.599957	1.970169
C	0.844168	-2.268736	1.088394
C	3.266255	-0.700586	1.088065
C	-0.675297	0.736074	2.094948
C	1.565151	1.562437	2.577818
C	0.741511	-3.157700	0.008126
C	0.556614	-2.705599	2.382904
C	4.111043	0.418148	0.961428
C	3.840350	-1.954333	1.341617
C	-1.636821	-0.398192	1.875353
C	-1.213697	1.940901	2.663990
C	1.061136	2.690576	3.175884
H	2.634702	1.405668	2.576205
C	0.334835	-4.478999	0.210578
H	0.992758	-2.819814	-0.995324
C	0.128511	-4.020185	2.610295
H	0.641843	-2.020433	3.221167
C	5.495932	0.297386	1.089928
H	3.701782	1.395559	0.743274
C	5.228770	-2.101047	1.473013
H	3.215131	-2.834980	1.433635
C	-2.109917	-0.829021	0.638333
C	-2.120244	-1.024898	3.082457
C	-0.329977	2.946560	3.176812
C	-2.615906	2.179292	2.747371
H	1.734743	3.411814	3.630521
C	0.023553	-4.885733	1.515883
C	6.039073	-0.970586	1.337795
C	-3.122699	-1.830335	0.587944
P	-1.444977	-0.133959	-0.947738
C	-3.127521	-2.038639	3.001880
C	-1.618370	-0.692939	4.374994
C	-0.866830	4.152997	3.702842
H	-3.302039	1.423271	2.387575

C	-3.106018	3.357772	3.261211
H	-0.304438	-5.909679	1.684088
H	7.118752	-1.077425	1.423417
C	-3.621216	-2.406839	1.727875
H	-3.525414	-2.136333	-0.367889
C	-2.342109	-1.090170	-2.243070
C	-2.202787	1.538324	-1.025554
C	-3.615925	-2.647649	4.189549
H	-0.839354	0.053571	4.471451
C	-2.098836	-1.311554	5.508010
H	-0.180638	4.905070	4.083806
C	-2.226097	4.360565	3.738167
H	-4.179040	3.519226	3.307239
H	-4.404478	-3.157526	1.659679
C	-3.182816	-0.459122	-3.168347
C	-2.111556	-2.470588	-2.351867
C	-3.463643	1.776832	-0.455875
C	-1.544123	2.572831	-1.695919
H	-4.391481	-3.404526	4.103999
C	-3.116843	-2.291065	5.421245
H	-1.694612	-1.041563	6.479281
H	-2.628868	5.284910	4.141756
C	-3.791608	-1.189006	-4.198450
H	-3.373532	0.606789	-3.091189
C	-2.718074	-3.223066	-3.363748
H	-1.464990	-2.969685	-1.633862
C	-4.049495	3.041911	-0.526024
H	-3.991648	0.972753	0.048452
C	-2.113030	3.851227	-1.787251
H	-0.569341	2.395254	-2.136762
H	-3.494752	-2.761351	6.324259
C	-3.547564	-2.564411	-4.279993
C	-3.359344	4.064999	-1.194117
H	-4.017365	-3.138942	-5.075589
H	-3.810130	5.053833	-1.251307
C	2.367544	1.596569	-1.403573
C	2.958810	0.580281	-1.857561
C	2.266687	2.956540	-0.932955
C	4.053584	-0.192835	-2.473364
C	1.197635	3.397750	-0.136302
C	3.296647	3.863342	-1.264286
H	4.849700	0.500578	-2.792761
O	3.620600	-0.972791	-3.585343
C	1.151494	4.717174	0.311112
H	0.412757	2.704065	0.135363
C	3.243592	5.179221	-0.813728
H	4.128204	3.524418	-1.875702
C	2.698250	-0.281213	-4.419330
C	2.169412	5.611585	-0.027045
H	0.319164	5.036812	0.931550
H	4.039395	5.870028	-1.077067
C	1.252658	-0.541500	-4.025903
H	2.915162	0.794906	-4.452543
H	2.843259	-0.682567	-5.430370
H	2.130871	6.639992	0.320491
C	0.358628	0.473124	-3.884278
H	0.675768	1.507112	-3.999578

H	-0.708746	0.286044	-3.845554
H	4.479574	-0.891248	-1.745670
C	0.873397	-1.997758	-4.023738
H	1.560422	-2.565145	-3.386361
H	-0.153419	-2.157631	-3.697641
H	0.982170	-2.402858	-5.039564
C	-4.721700	-0.509590	-5.175794
H	-4.655262	-0.960162	-6.171561
H	-5.765314	-0.598640	-4.847242
H	-4.497680	0.557982	-5.268229
C	-2.446454	-4.704396	-3.482610
H	-3.203031	-5.204610	-4.094551
H	-1.470368	-4.888297	-3.950797
H	-2.428798	-5.187101	-2.499861
C	-1.366625	4.961027	-2.488376
H	-0.472062	5.244042	-1.920029
H	-1.030100	4.646816	-3.483486
H	-1.988629	5.853053	-2.608931
C	-5.379149	3.319975	0.132794
H	-5.235943	3.885822	1.062364
H	-6.031502	3.917624	-0.513226
H	-5.906229	2.394559	0.385814
C	6.387583	1.510372	0.958127
H	7.238063	1.308953	0.296894
H	5.839315	2.368997	0.556369
H	6.799801	1.804587	1.931473
C	5.828214	-3.454325	1.776783
H	5.931669	-3.601197	2.859702
H	5.200979	-4.266615	1.395223
H	6.825628	-3.558558	1.337467
C	-0.243377	-4.468869	4.002353
H	-1.265022	-4.153575	4.245539
H	-0.194337	-5.557737	4.101307
H	0.417794	-4.025970	4.754669
C	0.259303	-5.453844	-0.940805
H	1.107962	-6.149127	-0.923107
H	-0.653117	-6.058705	-0.894140
H	0.275630	-4.938807	-1.906616

TS2-R

B3LYP-D3 SCF energy: -3381.72268127 a.u.
B3LYP-D3 enthalpy: -3380.571089 a.u.
B3LYP-D3 Gibbs free energy: -3380.743232 a.u.
M06 SCF energy in solution: -3381.31114711 a.u.
M06 enthalpy: -3380.159555 a.u.
M06 Gibbs free energy: -3380.331698 a.u.
Imaginary frequency: -232.2000 cm-1

Cartesian coordinates

ATOM	X	Y	Z
Rh	0.764225	-1.393104	-0.322073
P	0.955215	0.854868	-0.722953
C	-0.607142	1.392487	-1.551279
C	1.243517	2.089930	0.588469
C	2.309130	1.061696	-1.952558

C	-1.767012	1.618118	-0.806386
C	-0.655648	1.431349	-2.972049
C	1.421583	1.662755	1.902965
C	1.221131	3.464914	0.297357
C	2.464859	0.065993	-2.935959
C	3.255273	2.085485	-1.865780
C	-1.763640	1.619337	0.695993
C	-3.009154	1.864050	-1.483459
C	-1.833379	1.686089	-3.631327
H	0.251372	1.273549	-3.543897
C	1.595581	2.587740	2.939555
H	1.393393	0.602564	2.124944
C	1.402428	4.406584	1.310565
H	1.033879	3.803870	-0.718275
C	3.542125	0.087693	-3.821993
H	1.747660	-0.748827	-2.992560
C	4.350258	2.129534	-2.743397
H	3.183794	2.835562	-1.086856
C	-1.658111	0.437960	1.431799
C	-1.898599	2.872624	1.380135
C	-3.037581	1.901704	-2.915609
C	-4.234076	2.052409	-0.782696
H	-1.851825	1.724908	-4.717581
C	1.588574	3.947521	2.624079
C	4.479740	1.127535	-3.706304
C	-1.711705	0.476383	2.850707
P	-1.357689	-1.156540	0.553221
C	-1.913998	2.895741	2.812465
C	-1.999855	4.112399	0.687550
C	-4.266368	2.143371	-3.585650
H	-4.237174	2.009593	0.299740
C	-5.410876	2.277329	-1.460977
H	1.711002	4.676904	3.422087
H	5.334164	1.144911	-4.379778
C	-1.832463	1.671161	3.518818
H	-1.663972	-0.445197	3.417493
C	-1.368115	-2.477572	1.824682
C	-2.866684	-1.349414	-0.460990
C	-2.020440	4.139131	3.491387
H	-1.983141	4.116795	-0.395780
C	-2.116362	5.300134	1.373429
H	-4.268837	2.180191	-4.672125
C	-5.431196	2.325996	-2.875610
H	-6.333768	2.418283	-0.905527
H	-1.872002	1.685467	4.605114
C	-2.257676	-3.555336	1.772416
C	-0.326614	-2.485194	2.771557
C	-4.126418	-1.284827	0.152820
C	-2.774669	-1.422781	-1.853816
H	-2.026051	4.140037	4.578331
C	-2.122997	5.318038	2.789528
H	-2.197492	6.233567	0.824053
H	-6.366428	2.509644	-3.396405
C	-2.126078	-4.634166	2.660011
H	-3.054470	-3.570394	1.035400
C	-0.180028	-3.543487	3.670301
H	0.391423	-1.669420	2.790472

C	-5.293298	-1.309536	-0.616130
C	-3.927679	-1.444305	-2.644874
H	-1.800345	-1.406605	-2.328063
H	-2.212275	6.263182	3.317273
C	-1.086363	-4.611511	3.594597
C	-5.172389	-1.394952	-2.008910
H	-0.971111	-5.450474	4.277868
C	2.734209	-1.484187	0.262257
C	2.738662	-2.552970	-0.477552
C	3.766872	-0.661353	0.883813
C	3.844779	-3.242638	-1.239771
C	4.743549	-0.027600	0.096626
C	3.822492	-0.527113	2.281317
H	4.147291	-2.573258	-2.063934
O	3.405261	-4.480289	-1.749062
C	5.735624	0.746694	0.698077
H	4.716318	-0.138060	-0.981838
C	4.827768	0.230700	2.877752
H	3.075840	-1.027379	2.892364
C	2.065968	-4.304989	-2.148761
C	5.779834	0.881405	2.087307
C	1.248155	-3.759462	-0.971227
H	1.988010	-3.623888	-3.013432
H	1.679197	-5.288095	-2.433271
H	6.556893	1.480230	2.553295
C	-0.007094	-3.132228	-1.341773
H	-0.136682	-2.887987	-2.395795
H	-0.903839	-3.532986	-0.875408
H	4.710830	-3.416016	-0.593995
C	1.221513	-4.703682	0.227078
H	0.630057	-5.589834	-0.037714
H	0.764342	-4.235986	1.099769
H	2.229341	-5.034768	0.486811
C	5.375154	3.230396	-2.604451
H	6.213799	3.092251	-3.293191
H	4.931624	4.212449	-2.808872
H	5.775123	3.264209	-1.583633
C	3.700024	-0.976613	-4.882693
H	3.432535	-0.588467	-5.873567
H	4.735556	-1.329218	-4.943908
H	3.059494	-1.842348	-4.682992
C	0.928947	-3.540994	4.695748
H	1.384561	-4.532244	4.792606
H	1.718923	-2.830105	4.430832
H	0.548618	-3.260745	5.686294
C	-3.104091	-5.784448	2.614956
H	-2.643325	-6.716751	2.956622
H	-3.966236	-5.588389	3.265545
H	-3.489518	-5.944552	1.602638
H	6.481211	1.236386	0.077552
H	4.870091	0.314064	3.960210
C	1.366746	5.887238	1.021100
H	0.560082	6.370516	1.584170
H	2.306255	6.372175	1.312030
H	1.198786	6.086666	-0.041812
C	1.775827	2.102854	4.356802
H	2.746984	1.607242	4.477166

H	1.726786	2.925633	5.076418
H	1.000032	1.374021	4.619881
C	-3.837929	-1.478078	-4.151207
H	-4.339158	-2.362076	-4.562750
H	-4.323420	-0.595167	-4.583612
H	-2.797768	-1.488366	-4.491242
C	-6.652006	-1.175797	0.027553
H	-7.028584	-0.151044	-0.090296
H	-7.384789	-1.846058	-0.434446
H	-6.617270	-1.394792	1.099344
H	-4.200906	-1.194986	1.233883
H	-6.075489	-1.396786	-2.616235

TS2-S

B3LYP-D3 SCF energy: -3381.71630333 a.u.
B3LYP-D3 enthalpy: -3380.564565 a.u.
B3LYP-D3 Gibbs free energy: -3380.736596 a.u.
M06 SCF energy in solution: -3381.30555562 a.u.
M06 enthalpy: -3380.153817 a.u.
M06 Gibbs free energy: -3380.325848 a.u.
Imaginary frequency: -197.5722 cm-1

Cartesian coordinates

ATOM	X	Y	Z
C	2.755377	2.547724	0.255429
C	2.872728	1.293848	-0.069607
C	3.507692	3.807122	-0.115988
C	1.636898	4.722307	0.863487
C	1.514720	3.363698	1.546084
C	0.152091	2.850942	1.697701
H	-0.638260	3.447201	1.243501
Rh	0.898138	1.215890	0.566790
H	-0.111388	2.487540	2.689250
C	3.870111	0.487990	-0.763125
C	4.176887	0.757540	-2.108461
C	4.517708	-0.575397	-0.114309
C	5.110768	-0.023392	-2.788436
H	3.673649	1.575830	-2.617868
C	5.450678	-1.351575	-0.798816
H	4.278819	-0.789384	0.921703
C	5.748334	-1.083141	-2.137785
H	6.474176	-1.691843	-2.669033
O	2.997901	4.942259	0.549679
H	0.992164	4.760146	-0.027365
H	1.342322	5.517744	1.555287
H	3.405624	3.923904	-1.206433
H	4.573217	3.720624	0.122342
P	-1.125329	1.311748	-0.465018
C	-1.314285	-0.103486	-1.632288
C	-2.729945	1.389789	0.403226
C	-0.973985	2.850888	-1.460655
C	-1.622178	-1.374959	-1.141774
C	-1.040083	0.080684	-3.014748
C	-2.788990	1.291526	1.796296
C	-3.915522	1.444713	-0.344552

C	0.206140	3.047393	-2.203747
C	-1.897344	3.895910	-1.354768
C	-1.960137	-1.609908	0.303755
C	-1.637816	-2.495210	-2.038819
C	-1.057142	-0.983145	-3.883645
H	-0.830550	1.072746	-3.395349
C	-4.021307	1.247261	2.455182
H	-1.872314	1.191107	2.365526
C	-5.159255	1.416599	0.292499
H	-3.872921	1.493210	-1.429884
C	0.461604	4.262651	-2.840184
H	0.947047	2.255114	-2.260125
C	-1.663824	5.126804	-1.988955
H	-2.802439	3.769231	-0.769911
C	-0.984690	-1.584300	1.303031
C	-3.326338	-1.884190	0.649158
C	-1.343959	-2.293037	-3.426778
C	-1.923167	-3.819075	-1.599801
H	-0.853427	-0.826121	-4.939781
C	-5.189768	1.319139	1.689296
C	-0.484314	5.292024	-2.719705
C	-1.343562	-1.855263	2.650973
P	0.740144	-1.049007	0.898968
C	-3.670515	-2.138917	2.016569
C	-4.370510	-1.899965	-0.318487
C	-1.349968	-3.402515	-4.313250
H	-2.133909	-3.995593	-0.551976
C	-1.932081	-4.873090	-2.485556
H	-6.153689	1.276182	2.192400
H	-0.289546	6.247483	-3.202692
C	-2.645897	-2.126544	2.994614
H	-0.579929	-1.861004	3.419519
C	1.758421	-1.371261	2.393562
C	1.257949	-2.241619	-0.383812
C	-5.023709	-2.397897	2.362416
H	-4.133767	-1.700563	-1.356743
C	-5.671931	-2.163574	0.046268
H	-1.123632	-3.231890	-5.362606
C	-1.639935	-4.667319	-3.855461
H	-2.162137	-5.873220	-2.129575
H	-2.902145	-2.339138	4.029512
C	2.771446	-2.334953	2.420132
C	1.589903	-0.519586	3.500451
C	1.125128	-3.620104	-0.151121
C	1.675378	-1.784012	-1.634541
H	-5.267226	-2.591919	3.404043
C	-6.006653	-2.411126	1.399352
H	-6.450216	-2.180398	-0.711405
H	-1.647695	-5.509209	-4.541608
C	3.616549	-2.455439	3.533706
H	2.932156	-2.982199	1.564728
C	2.412532	-0.624418	4.623689
H	0.813396	0.241211	3.479459
C	1.424483	-4.540826	-1.156896
H	0.761964	-3.977831	0.809323
C	1.972399	-2.686732	-2.661362
H	1.737382	-0.719321	-1.822538

H	-7.037821	-2.615689	1.672204
C	3.426095	-1.594241	4.617902
C	1.845221	-4.053825	-2.402658
H	4.085743	-1.673413	5.479687
H	2.056369	-4.764174	-3.199508
C	2.386343	3.217040	2.798965
H	2.400264	2.181921	3.149000
H	1.965346	3.849651	3.591201
H	3.414090	3.539749	2.622935
C	4.699418	-3.508649	3.556369
H	5.537028	-3.210382	4.194709
H	4.312393	-4.459007	3.946246
H	5.088568	-3.704116	2.551352
C	2.216855	0.279420	5.818234
H	1.718547	-0.253931	6.637674
H	3.175551	0.641872	6.204815
H	1.601773	1.150225	5.568154
C	1.743203	4.484240	-3.608633
H	1.543415	4.835686	-4.627337
H	2.365252	5.245712	-3.121613
H	2.332926	3.564203	-3.680922
C	-2.682732	6.238008	-1.894671
H	-2.215287	7.222031	-2.000535
H	-3.434183	6.145255	-2.689437
H	-3.215739	6.213770	-0.938394
C	-4.098617	1.068638	3.951660
H	-4.779646	1.795227	4.408668
H	-4.476660	0.067988	4.196011
H	-3.117032	1.177503	4.423388
C	-6.441612	1.426743	-0.502489
H	-6.866417	0.415764	-0.546445
H	-7.193911	2.077796	-0.044325
H	-6.278953	1.763984	-1.530955
H	5.946145	-2.171107	-0.285391
H	5.340890	0.196327	-3.827395
C	1.238281	-6.022494	-0.939922
H	0.342525	-6.374390	-1.467528
H	2.089233	-6.595279	-1.324858
H	1.116552	-6.264906	0.120513
C	2.413398	-2.190129	-4.016506
H	3.505411	-2.106342	-4.064196
H	2.091424	-2.869674	-4.812672
H	2.000019	-1.198088	-4.225161

Computed IR information Using Spartan

SPARTAN'20

build 1.1.4 (Jan 13 2022)

Wavefunction Developers:

B.J. Deppmeier, A.J. Driessen, W.J. Hehre, T.S. Hehre,

J.A. Johnson, W.S. Ohlinger, P.E. Klunzinger

Please cite Spartan as:

Spartan'20

Wavefunction Inc.

Irvine CA

Q-Chem 5.1, Q-Chem, Inc., Pleasanton, CA (2020)

Q-Chem Developers:

Yihan Shao, Zhengting Gan, E. Epifanovsky, A. T. B. Gilbert, M. Wormit,

J. Kussmann, A. W. Lange, A. Behn, Jia Deng, Xintian Feng, D. Ghosh,

M. Goldey, P. R. Horn, L. D. Jacobson, I. Kaliman, T. Kus, A. Landau, Jie Liu,

E. I. Proynov, R. M. Richard, R. P. Steele, E. J. Sundstrom,

H. L. Woodcock III, P. M. Zimmerman, D. Zuev, B. Alam, B. Albrecht,

E. Alguire, S. A. Baeppler, D. Barton, Z. Benda, Y. A. Bernard,

E. J. Berquist, K. B. Bravaya, H. Burton, K. Carter-Fenk, D. Casanova,

Chun-Min Chang, Yunqing Chen, A. Chien, K. D. Closser, M. P. Coons,

S. Coriani, S. Dasgupta, A. L. Dempwolff, M. Diedenhofen, Hainam Do,

R. G. Edgar, Po-Tung Fang, S. Faraji, S. Fatehi, Qingguo Feng, J. Fosso-Tande,

J. Gayvert, Qinghui Ge, A. Ghysels, G. Gidofalvi, J. Gomes, J. Gonthier,

A. Gunina, D. Hait, M. W. D. Hanson-Heine, P. H. P. Harbach, A. W. Hauser,

M. F. Herbst, J. E. Herr, E. G. Hohenstein, Z. C. Holden, Kerwin Hui,
B. C. Huynh, T.-C. Jagau, Hyunjun Ji, B. Kaduk, K. Khistyaev, Jaehoon Kim,
P. Klunzinger, K. Koh, D. Kosenkov, L. Koulias, T. Kowalczyk, C. M. Krauter,
A. Kunitsa, Ka Un Lao, A. Laurent, K. V. Lawler, Joonho Lee, D. Lefrancois,
S. Lehtola, D. S. Levine, Yi-Pei Li, You-Sheng Lin, Fenglai Liu, Kuan-Yu Liu,
E. Livshits, M. Loipersberger, A. Luenser, P. Manohar, E. Mansoor,
S. F. Manzer, Shan-Ping Mao, Yuezhi Mao, N. Mardirossian, A. V. Marenich,
T. Markovich, L. A. Martinez-Martinez, S. A. Maurer, N. J. Mayhall,
S. C. McKenzie, J.-M. Mewes, P. Morgante, A. F. Morrison, J. W. Mullinax,
K. Nanda, T. S. Nguyen-Beck, R. Olivares-Amaya, J. A. Parkhill, S. K. Paul,
Zheng Pei, T. M. Perrine, F. Plasser, P. Pokhilko, S. Prager, A. Prociuk,
E. Ramos, B. Rana, D. R. Rehn, F. Rob, M. Scheurer, M. Schneider, N. Sergueev,
S. M. Sharada, S. Sharma, D. W. Small, T. Stauch, C. J. Stein, T. Stein,
Yu-Chuan Su, S. P. Veccham, A. J. W. Thom, A. Tkatchenko, T. Tsuchimochi,
N. M. Tubman, L. Vogt, M. L. Vidal, O. Vydrov, M. A. Watson, J. Wenzel,
M. de Wergifosse, T. A. Wesolowski, A. White, J. Witte, A. Yamada, Jun Yang,
K. Yao, S. Yeganeh, S. R. Yost, Zhi-Qiang You, A. Zech, Igor Ying Zhang,
Xing Zhang, Yan Zhao, Ying Zhu, B. R. Brooks, G. K. L. Chan, C. J. Cramer,
M. S. Gordon, W. J. Hehre, A. Klamt, M. W. Schmidt, C. D. Sherrill,
D. G. Truhlar, A. Aspuru-Guzik, R. Baer, A. T. Bell, N. A. Besley,
Jeng-Da Chai, A. E. DePrince, III, R. A. DiStasio Jr., A. Dreuw,
B. D. Dunietz, T. R. Furlani, Chao-Ping Hsu, Yousung Jung, Jing Kong,
D. S. Lambrecht, WanZhen Liang, C. Ochsenfeld, V. A. Rassolov,
L. V. Slipchenko, J. E. Subotnik, T. Van Voorhis, J. M. Herbert, A. I. Krylov,
P. M. W. Gill, M. Head-Gordon,

Contributors to earlier versions of Q-Chem not listed above:

R. D. Adamson, B. Austin, J. Baker, G. J. O. Beran, K. Brandhorst,

S. T. Brown, E. F. C. Byrd, A. K. Chakraborty, C.-L. Cheng, Siu Hung Chien,

D. M. Chipman, D. L. Crittenden, H. Dachsel, R. J. Doerksen, A. D. Dutoi,

L. Fusti-Molnar, W. A. Goddard III, A. Golubeva-Zadorozhnaya, S. R. Gwaltney,

G. Hawkins, A. Heyden, S. Hirata, G. Kedziora, F. J. Keil, C. Kelley,

Jihan Kim, R. A. King, R. Z. Khaliullin, P. P. Korambath, W. Kurlancheek,

A. M. Lee, M. S. Lee, S. V. Levchenko, Ching Yeh Lin, D. Liotard,

R. C. Lochan, I. Lotan, P. E. Maslen, N. Nair, D. P. O'Neill, D. Neuhauser,

E. Neuscamman, C. M. Oana, R. Olson, B. Peters, R. Peverati, P. A. Pieniazek,

Y. M. Rhee, J. Ritchie, M. A. Rohrdanz, E. Rosta, N. J. Russ,

H. F. Schaefer III, N. E. Schultz, N. Shenvi, A. C. Simmonett, A. Sodt,

D. Stuck, K. S. Thanthiriwatte, V. Vanovschi, Tao Wang, A. Warshel,

C. F. Williams, Q. Wu, X. Xu, W. Zhang,

Please cite Q-Chem as follows :

Y.Shao et al., Mol.Phys. 113, 184 - 215 (2015)

DOI : 10.1080/00268976.2014.952696

Parts of Q-Chem use Armadillo 8.300.2 (tropical Shenanigans).

http://arma.sourceforge.net/

Wavefunction Inc.   Sales: sales@wavefun.com

Irvine CA   Support: support@wavefun.com

Web: www.wavefun.com

Copyright © 1995 - 2021

--

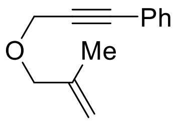

Ph
O
Me

6a

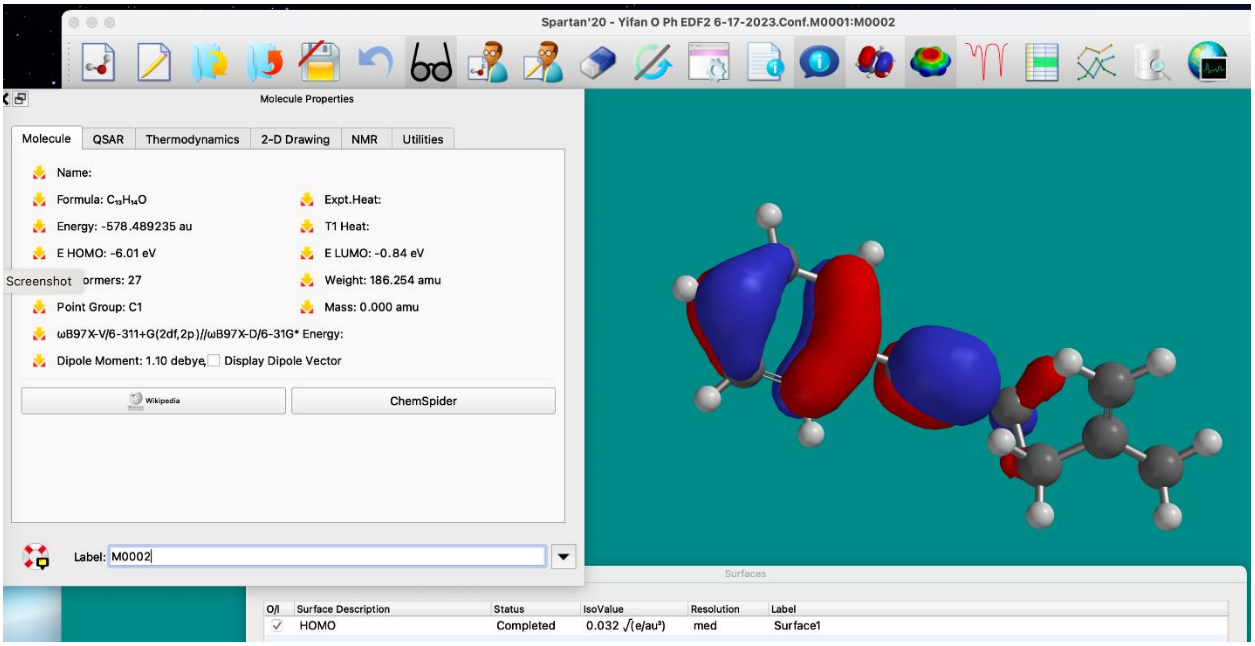

Spartan'20 - Yifan O Ph EDF2 6-17-2023.Conf.M0001:M0002
Molecule Properties
Molecule
QSAR
Thermodynamics
2-D Drawing
NMR
Utilities
Name:
Formula: C13H14O
Expt.Heat:
Energy: -578.489235 au
T1 Heat:
E HOMO: -6.01 eV
E LUMO: -0.84 eV
Screenshot
ormers: 27
Weight: 186.254 amu
Point Group: C1
Mass: 0.000 amu
ωB97X-V/6-311+G(2df,2p)//ωB97X-D/6-31G* Energy:
Dipole Moment: 1.10 debye
Display Dipole Vector
Wikipedia
ChemSpider
Label: M0002
Surfaces
O/I
Surface Description
Status
IsoValue
Resolution
Label
HOMO
Completed
0.032 √(e/au³)
med
Surface1

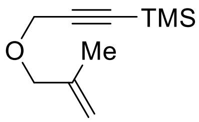
TMS
O
Me

S19

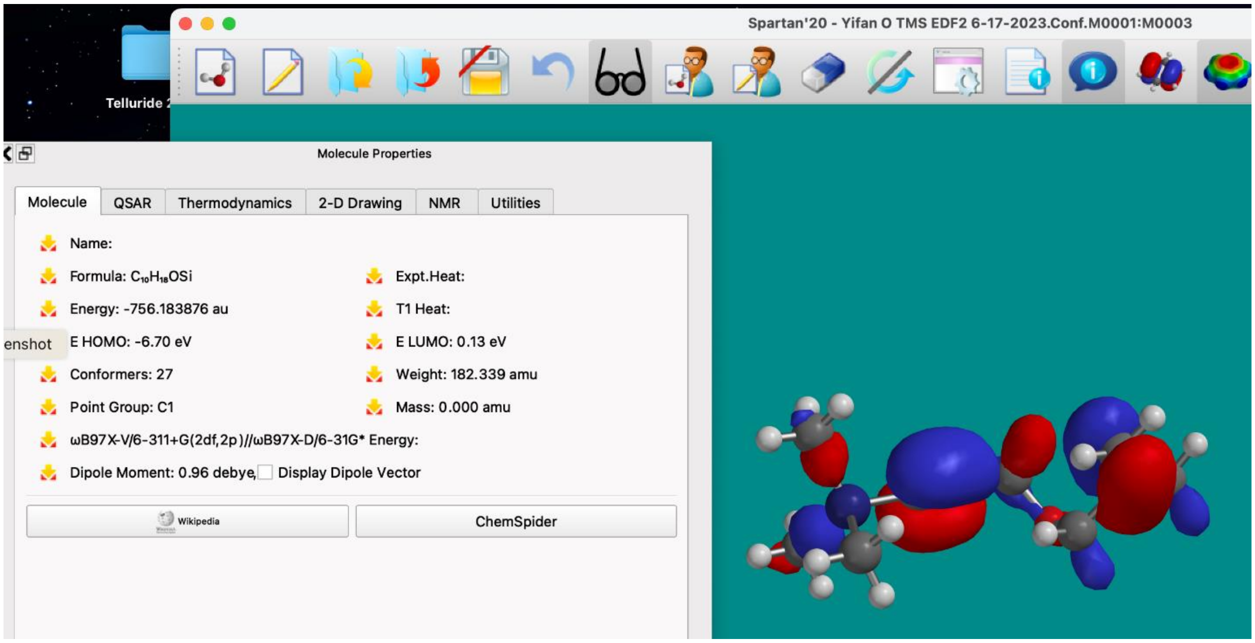
Telluride
Spartan'20 - Yifan O TMS EDF2 6-17-2023.Conf.M0001:M0003
Molecule Properties
Molecule
QSAR
Thermodynamics
2-D Drawing
NMR
Utilities
Name:
Formula: C10H18OSi
Expt.Heat:
Energy: -756.183876 au
T1 Heat:
enshot
E HOMO: -6.70 eV
E LUMO: 0.13 eV
Conformers: 27
Weight: 182.339 amu
Point Group: C1
Mass: 0.000 amu
ωB97X-V/6-311+G(2df,2p)//ωB97X-D/6-31G* Energy:
Dipole Moment: 0.96 debye
Display Dipole Vector
Wikipedia
ChemSpider

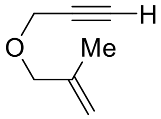
H
O
Me

S18

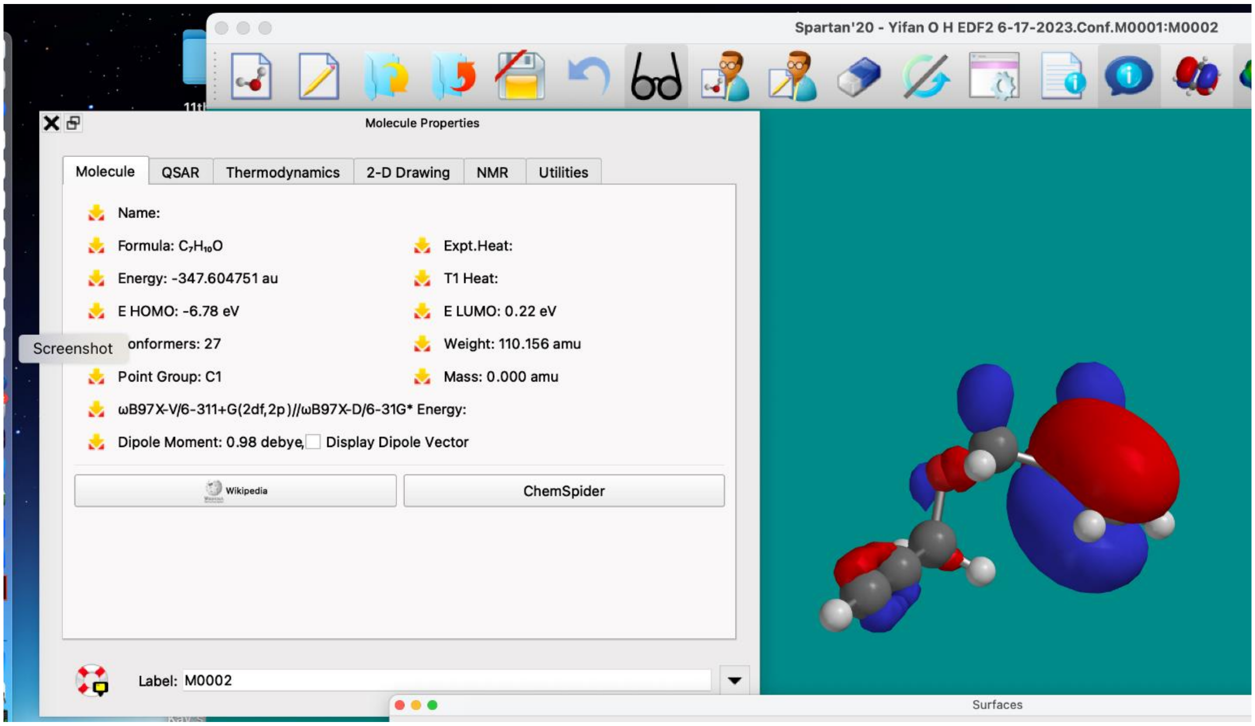
Spartan'20 - Yifan O H EDF2 6-17-2023.Conf.M0001:M0002
Molecule Properties
Molecule
QSAR
Thermodynamics
2-D Drawing
NMR
Utilities
Name:
Formula: C7H10O
Expt.Heat:
Energy: -347.604751 au
T1 Heat:
E HOMO: -6.78 eV
E LUMO: 0.22 eV
Screenshot
onformers: 27
Weight: 110.156 amu
Point Group: C1
Mass: 0.000 amu
ωB97X-V/6-311+G(2df,2p)//ωB97X-D/6-31G* Energy:
Dipole Moment: 0.98 debye
Display Dipole Vector
Wikipedia
ChemSpider
Label: M0002
Surfaces

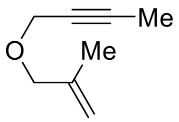

Me
O
Me

6b

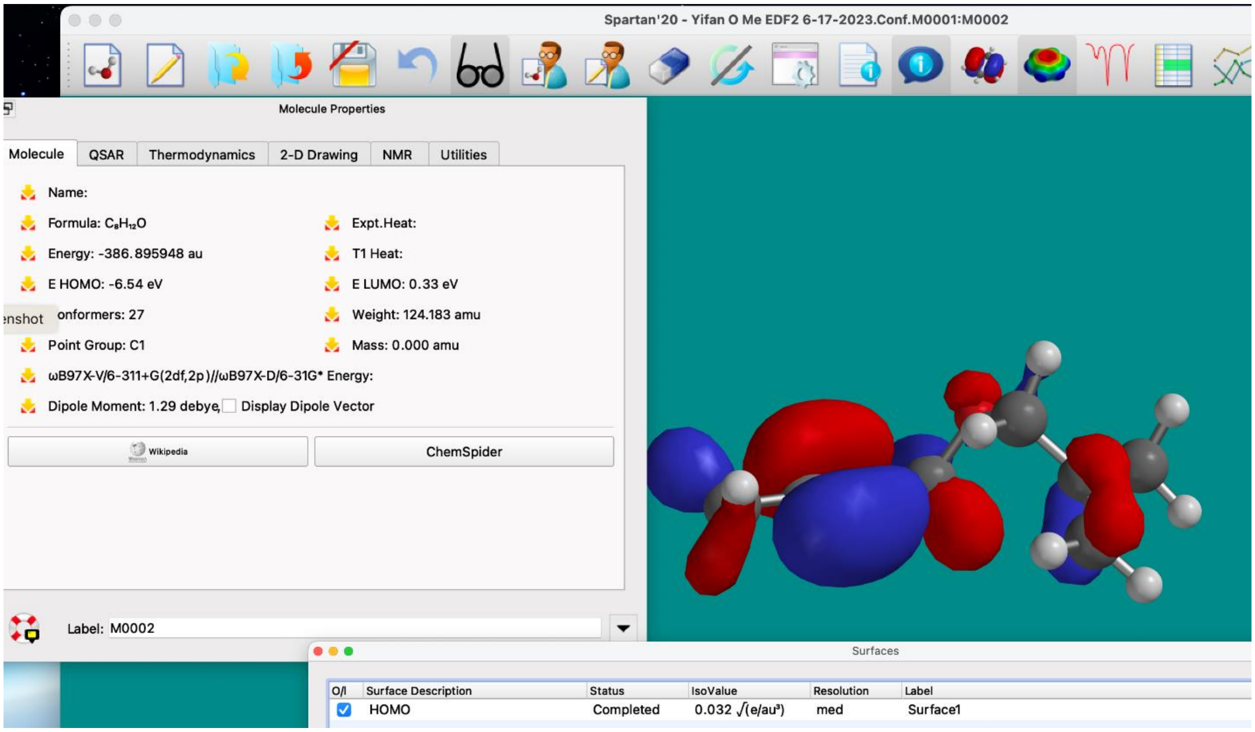

Spartan'20 - Yifan O Me EDF2 6-17-2023.Conf.M0001:M0002
Molecule Properties
Molecule
QSAR
Thermodynamics
2-D Drawing
NMR
Utilities
Name:
Formula: C8H12O
Expt.Heat:
Energy: -386.895948 au
T1 Heat:
E HOMO: -6.54 eV
E LUMO: 0.33 eV
onformers: 27
Weight: 124.183 amu
Point Group: C1
Mass: 0.000 amu
ωB97X-V/6-311+G(2df,2p)//ωB97X-D/6-31G* Energy:
Dipole Moment: 1.29 debye
Display Dipole Vector
Wikipedia
ChemSpider
Label: M0002
Surfaces
O/I
Surface Description
Status
IsoValue
Resolution
Label
HOMO
Completed
0.032 √(e/au³)
med
Surface1

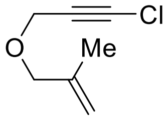
Cl
O
Me

S20

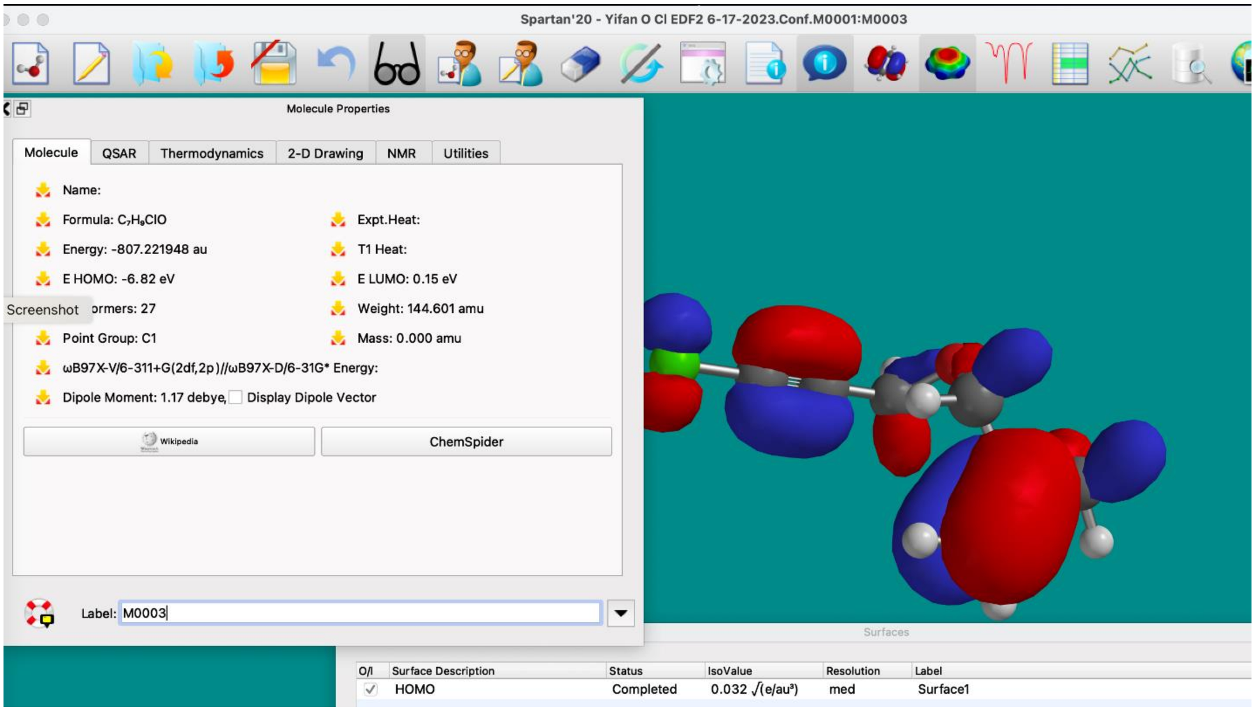
Spartan'20 - Yifan O Cl EDF2 6-17-2023.Conf.M0001:M0003
Molecule Properties
Molecule
QSAR
Thermodynamics
2-D Drawing
NMR
Utilities
Name:
Formula: C7H8ClO
Expt.Heat:
Energy: -807.221948 au
T1 Heat:
E HOMO: -6.82 eV
E LUMO: 0.15 eV
Screenshot
ormers: 27
Weight: 144.601 amu
Point Group: C1
Mass: 0.000 amu
ωB97X-V/6-311+G(2df,2p)//ωB97X-D/6-31G* Energy:
Dipole Moment: 1.17 debye
Display Dipole Vector
Wikipedia
ChemSpider
Label: M0003
Surfaces
O/I
Surface Description
Status
IsoValue
Resolution
Label
HOMO
Completed
0.032 √(e/au³)
med
Surface1

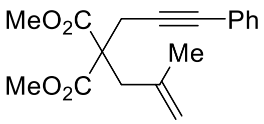
MeO2C
Ph
Me
MeO2C

S21

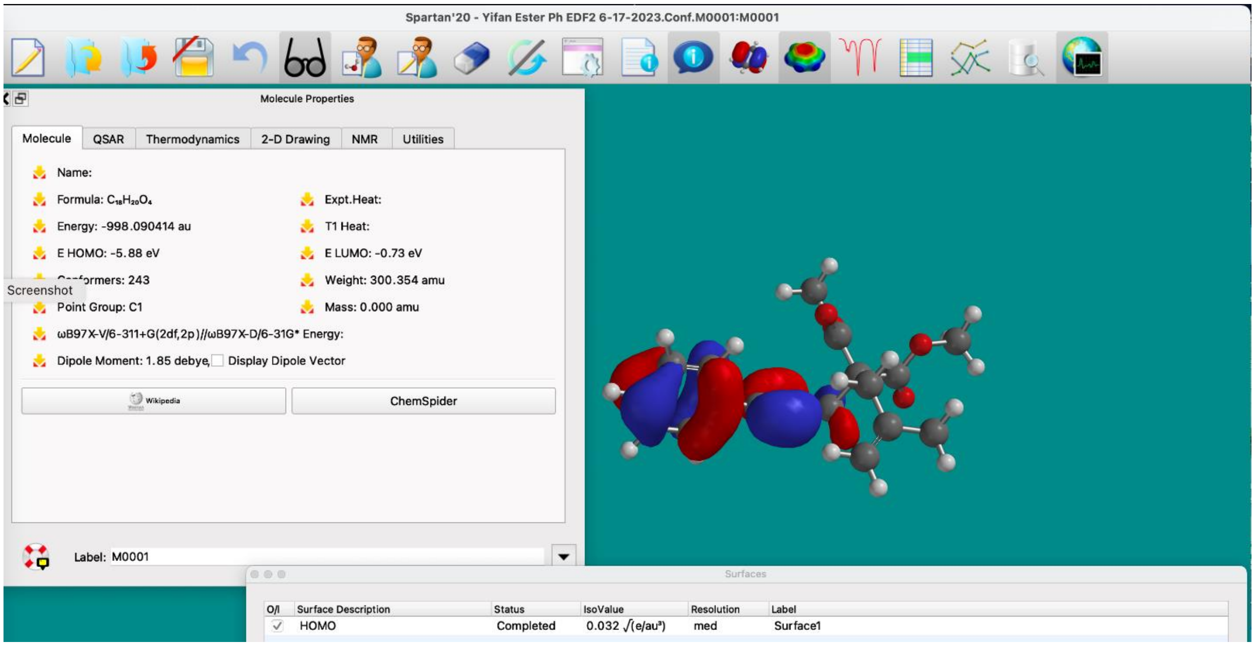
Spartan'20 - Yifan Ester Ph EDF2 6-17-2023.Conf.M0001:M0001
Molecule Properties
Molecule
QSAR
Thermodynamics
2-D Drawing
NMR
Utilities
Name:
Formula: C18H20O4
Expt.Heat:
Energy: -998.090414 au
T1 Heat:
E HOMO: -5.88 eV
E LUMO: -0.73 eV
Conformers: 243
Weight: 300.354 amu
Screenshot
Point Group: C1
Mass: 0.000 amu
ωB97X-V/6-311+G(2df,2p)//ωB97X-D/6-31G* Energy:
Dipole Moment: 1.85 debye,
Display Dipole Vector
ChemSpider
Label: M0001
Surfaces
O/I
Surface Description
Status
IsoValue
Resolution
Label
HOMO
Completed
0.032 √(e/au³)
med
Surface1

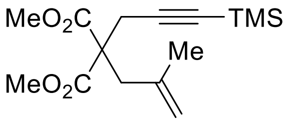
MeO2C
TMS
Me
MeO2C

S24

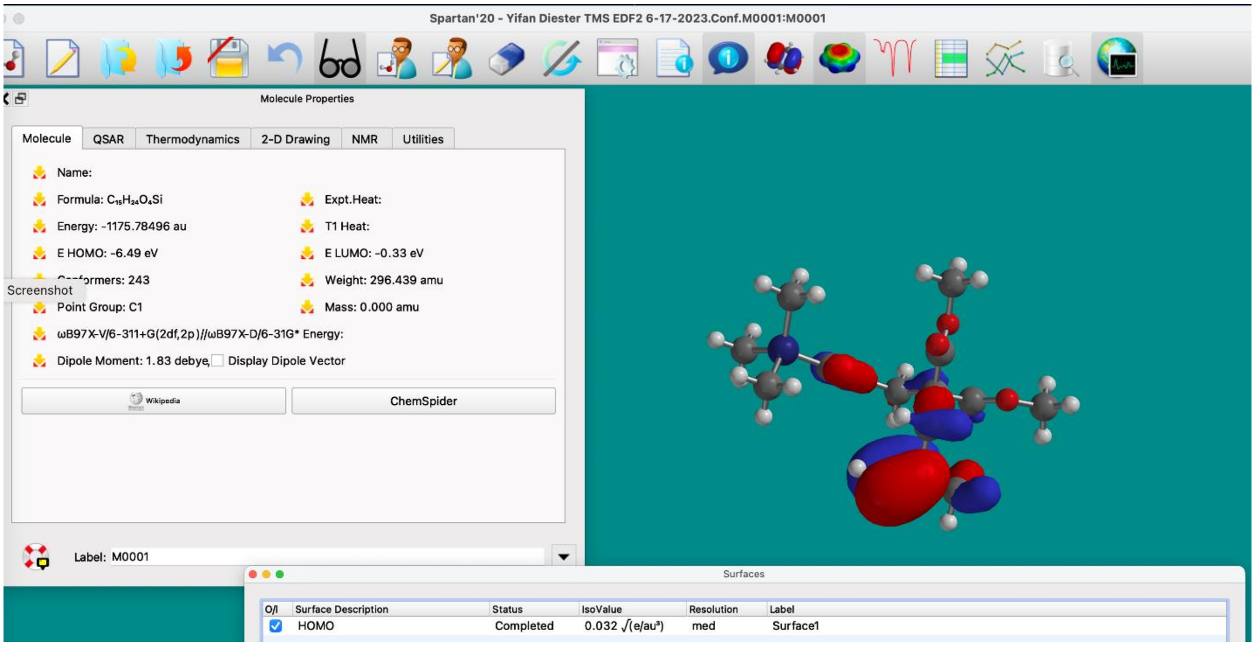
Spartan'20 - Yifan Diester TMS EDF2 6-17-2023.Conf.M0001:M0001
Molecule Properties
Molecule
QSAR
Thermodynamics
2-D Drawing
NMR
Utilities
Name:
Formula: C15H24O4Si
Expt.Heat:
Energy: -1175.78496 au
T1 Heat:
E HOMO: -6.49 eV
E LUMO: -0.33 eV
Weight: 296.439 amu
Point Group: C1
Mass: 0.000 amu
ωB97X-V/6-311+G(2df,2p)//ωB97X-D/6-31G* Energy:
Dipole Moment: 1.83 debye, Display Dipole Vector
Wikipedia
ChemSpider
Label: M0001
Surfaces
O/I
Surface Description
Status
IsoValue
Resolution
Label
HOMO
Completed
0.032 √(e/au³)
med
Surface1

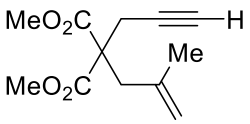
MeO2C
H
Me
MeO2C

S23

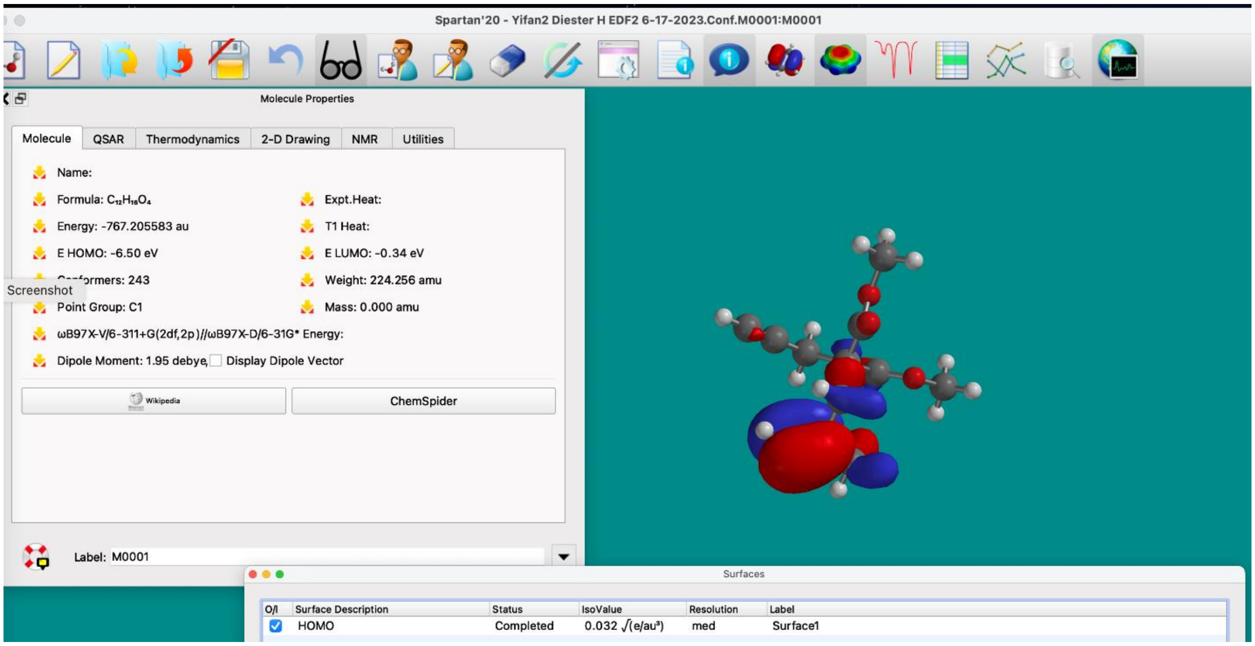
Spartan'20 - Yifan2 Diester H EDF2 6-17-2023.Conf.M0001:M0001
Molecule Properties
Molecule
QSAR
Thermodynamics
2-D Drawing
NMR
Utilities
Name:
Formula: C12H16O4
Expt.Heat:
Energy: -767.205583 au
T1 Heat:
E HOMO: -6.50 eV
E LUMO: -0.34 eV
Conformers: 243
Weight: 224.256 amu
Screenshot
Point Group: C1
Mass: 0.000 amu
ωB97X-V/6-311+G(2df,2p)//ωB97X-D/6-31G* Energy:
Dipole Moment: 1.95 debye
Display Dipole Vector
Wikipedia
ChemSpider
Label: M0001
Surfaces
O/I
Surface Description
Status
IsoValue
Resolution
Label
HOMO
Completed
0.032 √(e/au³)
med
Surface1

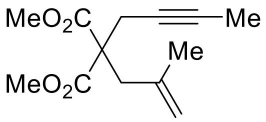
MeO2C
Me
Me
MeO2C

S22

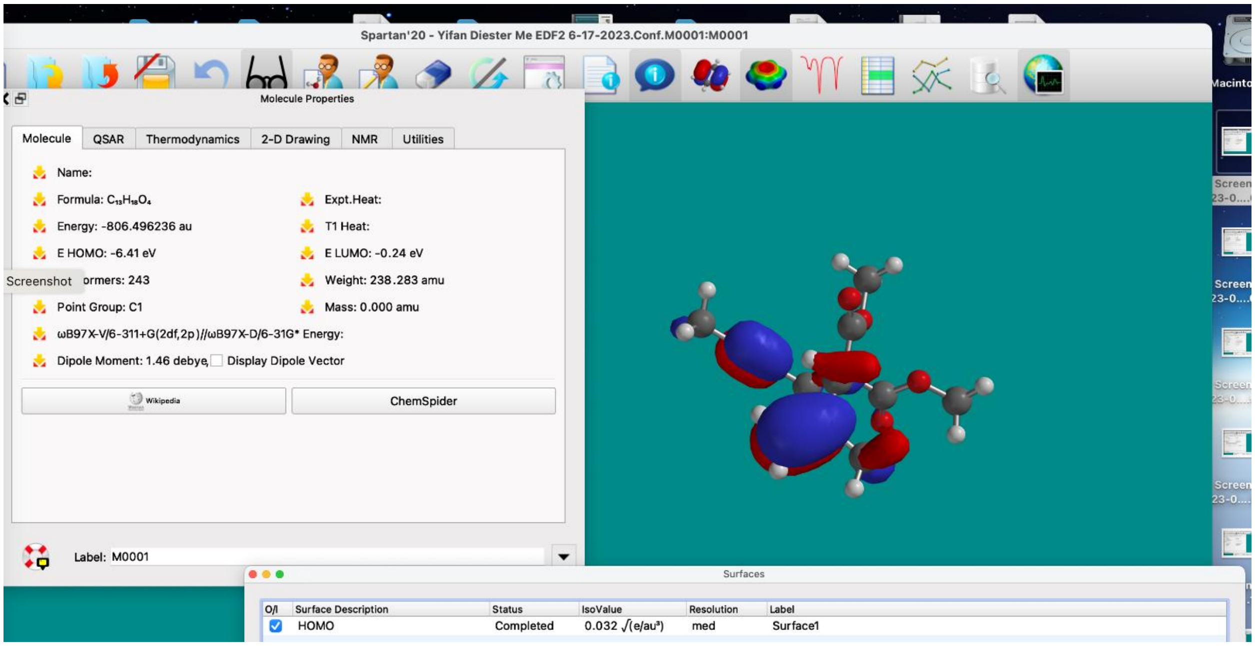
Spartan'20 - Yifan Diester Me EDF2 6-17-2023.Conf.M0001:M0001
Molecule Properties
Molecule
QSAR
Thermodynamics
2-D Drawing
NMR
Utilities
Name:
Formula: C13H18O4
Expt.Heat:
Energy: -806.496236 au
T1 Heat:
E HOMO: -6.41 eV
E LUMO: -0.24 eV
Screenshot
ormers: 243
Weight: 238.283 amu
Point Group: C1
Mass: 0.000 amu
ωB97X-V/6-311+G(2df,2p)//ωB97X-D/6-31G* Energy:
Dipole Moment: 1.46 debye
Display Dipole Vector
Wikipedia
ChemSpider
Label: M0001
Surfaces
O/I
Surface Description
Status
IsoValue
Resolution
Label
HOMO
Completed
0.032 √(e/au³)
med
Surface1

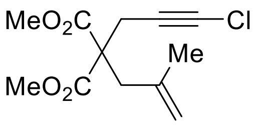
MeO2C
MeO2C
Cl
Me

S25

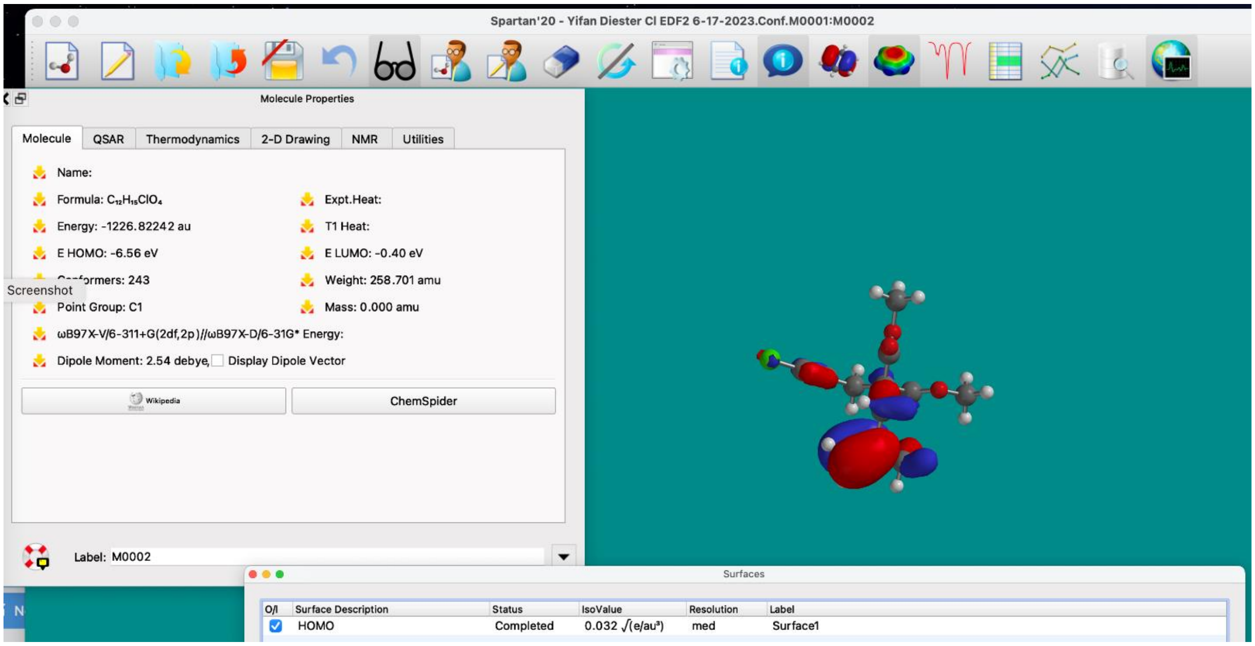
Spartan'20 - Yifan Diester Cl EDF2 6-17-2023.Conf.M0001:M0002
Molecule Properties
Molecule
QSAR
Thermodynamics
2-D Drawing
NMR
Utilities
Name:
Formula: C12H15ClO4
Expt.Heat:
Energy: -1226.82242 au
T1 Heat:
E HOMO: -6.56 eV
E LUMO: -0.40 eV
Conformers: 243
Weight: 258.701 amu
Screenshot
Point Group: C1
Mass: 0.000 amu
ωB97X-V/6-311+G(2df,2p)//ωB97X-D/6-31G* Energy:
Dipole Moment: 2.54 debye,
Display Dipole Vector
Wikipedia
ChemSpider
Label: M0002
Surfaces
O/I
Surface Description
Status
IsoValue
Resolution
Label
HOMO
Completed
0.032 √(e/au³)
med
Surface1

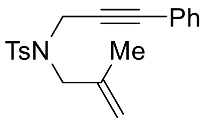
TsN
Me
Ph

4a

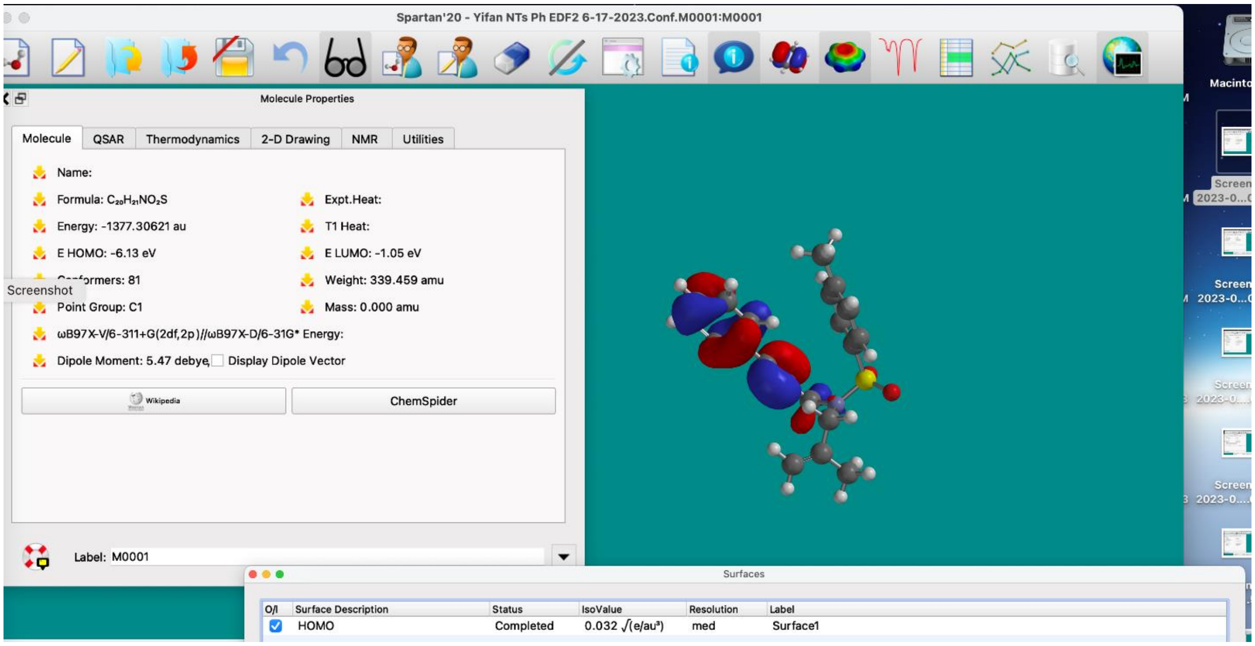
Spartan'20 - Yifan NTs Ph EDF2 6-17-2023.Conf.M0001:M0001
Molecule Properties
Molecule
QSAR
Thermodynamics
2-D Drawing
NMR
Utilities
Name:
Formula: C20H21NO2S
Expt.Heat:
Energy: -1377.30621 au
T1 Heat:
E HOMO: -6.13 eV
E LUMO: -1.05 eV
Conformers: 81
Weight: 339.459 amu
Point Group: C1
Mass: 0.000 amu
ωB97X-V/6-311+G(2df,2p)//ωB97X-D/6-31G* Energy:
Dipole Moment: 5.47 debye
Display Dipole Vector
ChemSpider
Label: M0001
Surfaces
Surface Description
Status
IsoValue
Resolution
Label
HOMO
Completed
0.032 √(e/au³)
med
Surface1

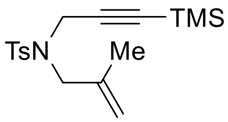

TMS
TsN
Me

4e

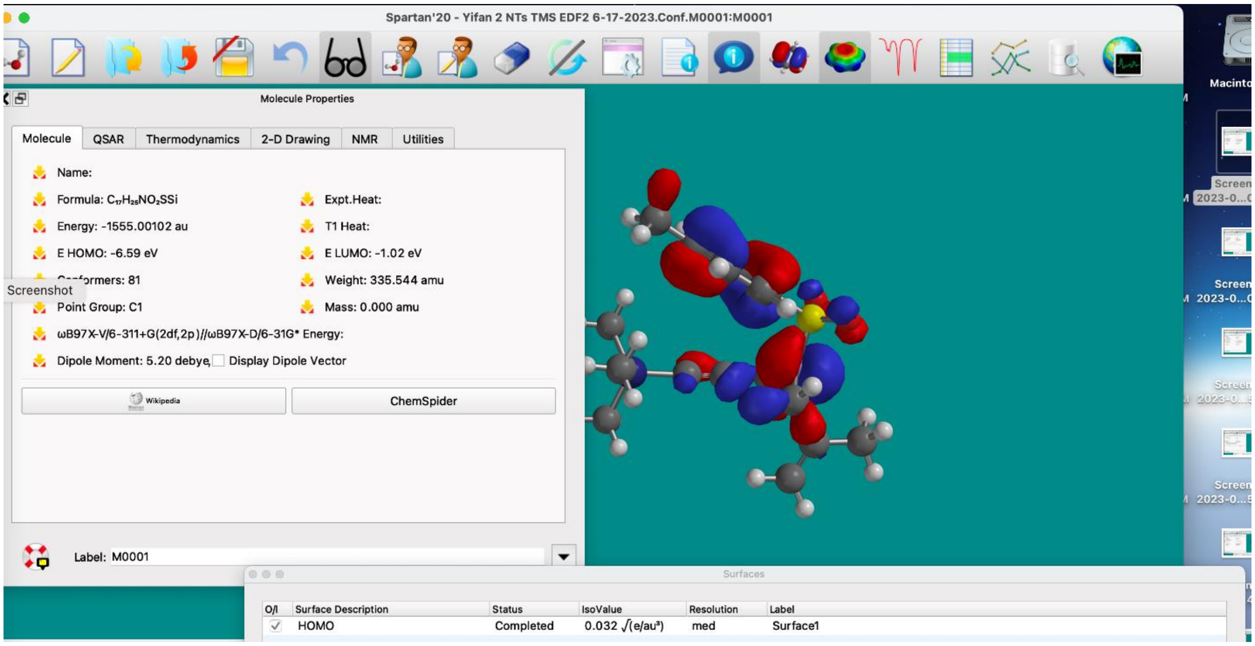

Spartan'20 - Yifan 2 NTs TMS EDF2 6-17-2023.Conf.M0001:M0001
Molecule Properties
Molecule
QSAR
Thermodynamics
2-D Drawing
NMR
Utilities
Name:
Formula: $C_{17}H_{25}NO_2SSi$
Expt.Heat:
Energy: -1555.00102 au
T1 Heat:
E HOMO: -6.59 eV
E LUMO: -1.02 eV
Weight: 335.544 amu
Screenshot
Point Group: C1
Mass: 0.000 amu
ωB97X-V/6-311+G(2df,2p)//ωB97X-D/6-31G* Energy:
Dipole Moment: 5.20 debye
Display Dipole Vector
ChemSpider
Label: M0001
Surfaces
O/I
Surface Description
Status
IsoValue
Resolution
Label
HOMO
Completed
0.032 √(e/au³)
med
Surface1

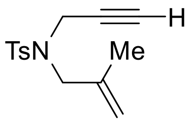
TsN
Me
H

4d

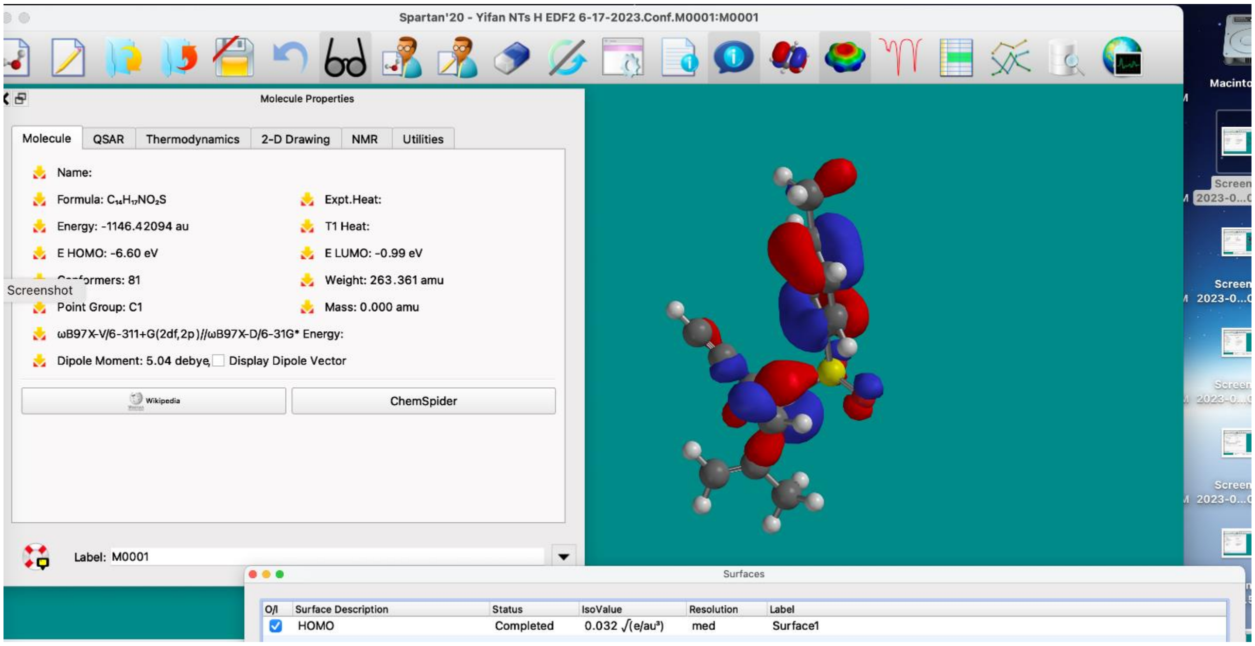
Spartan'20 - Yifan NTs H EDF2 6-17-2023.Conf.M0001:M0001
Molecule Properties
Molecule
QSAR
Thermodynamics
2-D Drawing
NMR
Utilities
Name:
Formula: C14H17NO2S
Expt.Heat:
Energy: -1146.42094 au
T1 Heat:
E HOMO: -6.60 eV
E LUMO: -0.99 eV
Conformers: 81
Weight: 263.361 amu
Point Group: C1
Mass: 0.000 amu
ωB97X-V/6-311+G(2df,2p)//ωB97X-D/6-31G* Energy:
Dipole Moment: 5.04 debye
Display Dipole Vector
Wikipedia
ChemSpider
Label: M0001
Surfaces
O/I
Surface Description
Status
IsoValue
Resolution
Label
HOMO
Completed
0.032 √(e/au³)
med
Surface1

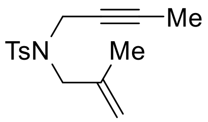
TsN
Me
Me

4b

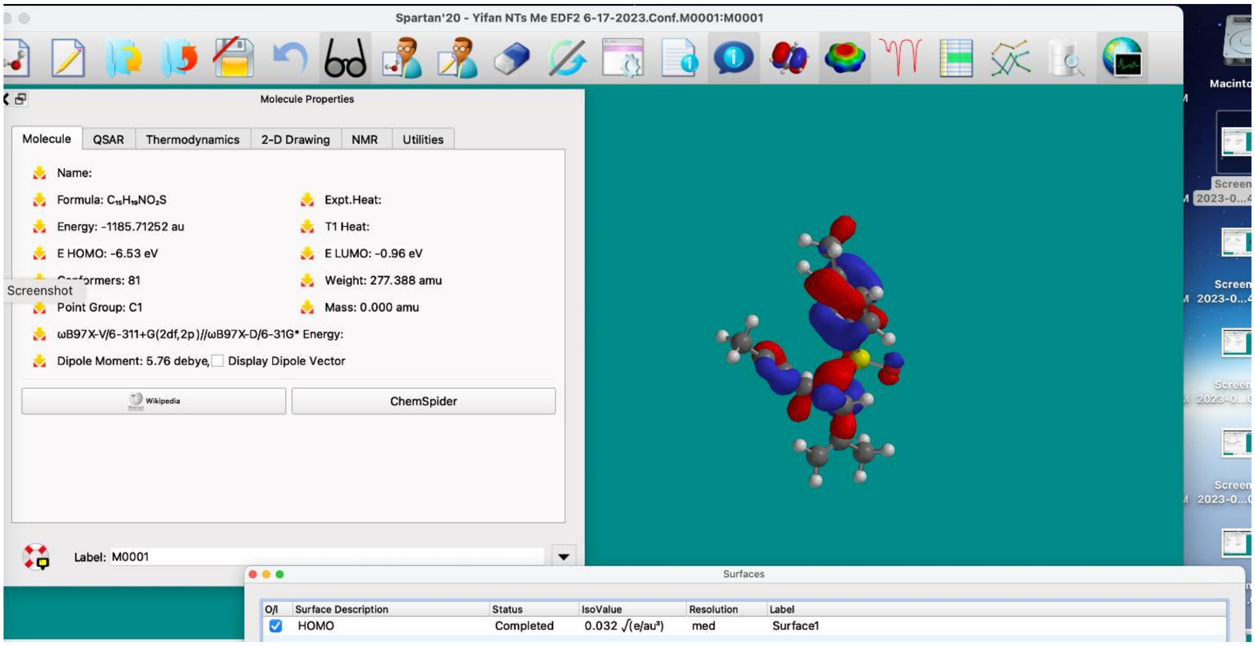
Spartan'20 - Yifan NTs Me EDF2 6-17-2023.Conf.M0001:M0001
Molecule Properties
Molecule
QSAR
Thermodynamics
2-D Drawing
NMR
Utilities
Name:
Formula: C15H19NO2S
Expt.Heat:
Energy: -1185.71252 au
T1 Heat:
E HOMO: -6.53 eV
E LUMO: -0.96 eV
Weight: 277.388 amu
Point Group: C1
Mass: 0.000 amu
ωB97X-V/6-311+G(2df,2p)//ωB97X-D/6-31G* Energy:
Dipole Moment: 5.76 debye
Display Dipole Vector
Screenshot
ChemSpider
Label: M0001
Surfaces
O/I
Surface Description
Status
IsoValue
Resolution
Label
HOMO
Completed
0.032 √(e/au³)
med
Surface1

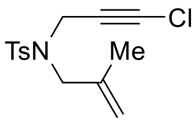

S17

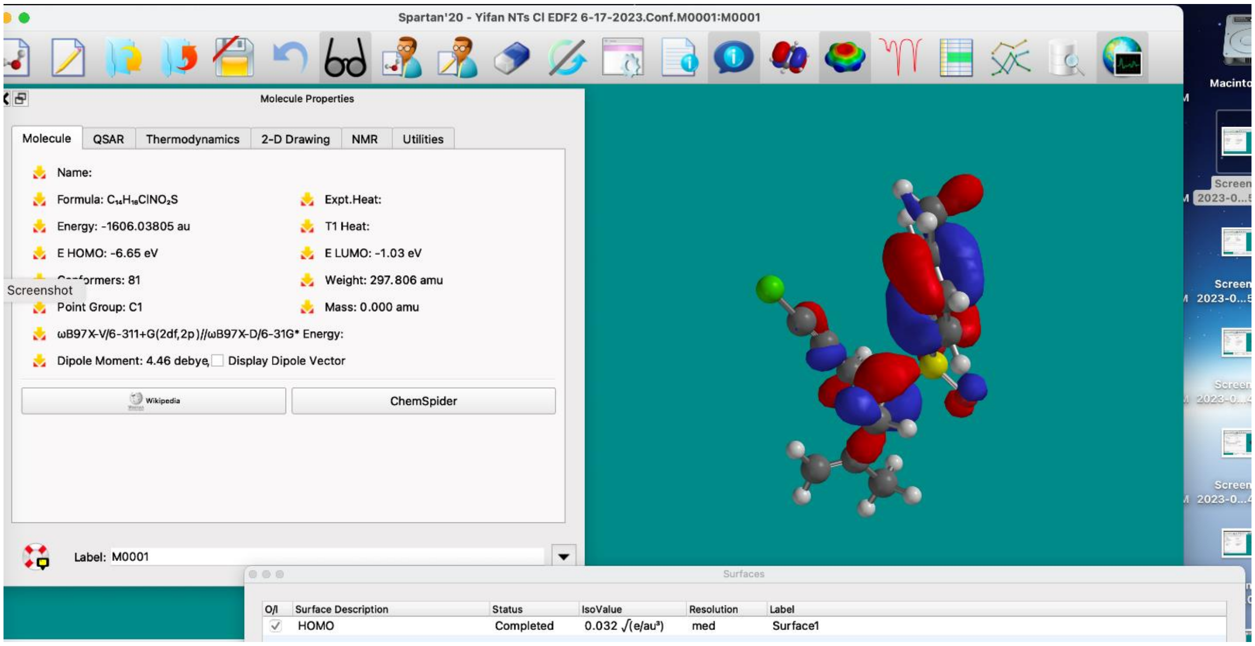

xyz coordinates (IR calculations)

6a

O	-1.057567	-0.515687	-2.347634
C	-0.171284	-1.577570	-2.008803
H	-0.756670	-2.487180	-2.183973
H	0.083489	-1.536107	-0.941800

C	-0.607792	0.757747	-1.919069
H	0.402490	0.964209	-2.303142
H	-1.289507	1.477747	-2.383386
C	-0.617448	0.917302	-0.465367
C	-0.628612	1.019220	0.741921
C	1.072629	-1.584143	-2.859039
C	2.282210	-1.474747	-2.308212
H	3.187996	-1.492760	-2.907945
H	2.411467	-1.374310	-1.234254
C	0.852010	-1.713901	-4.336481
H	0.361420	-2.665834	-4.576325
H	1.790116	-1.663732	-4.894698
H	0.185283	-0.924294	-4.698426
C	-0.653670	1.135718	2.159818
C	-0.701757	1.351155	4.950811
C	-1.572561	0.391899	2.917129
C	0.240252	1.989551	2.824408
C	0.211272	2.095351	4.208122
C	-1.591327	0.500128	4.300598
H	-2.265431	-0.267780	2.406255
H	0.952328	2.564257	2.242215
H	0.906810	2.760616	4.710058
H	-2.306022	-0.081433	4.874439
H	-0.720120	1.434579	6.032779

S19

O	-0.918924	-0.908091	-2.297407
C	0.132653	-1.799164	-1.939453

H	-0.295334	-2.797088	-2.085460
H	0.383056	-1.688334	-0.876303
C	-0.667947	0.439307	-1.940142
H	0.296514	0.779107	-2.347262
H	-1.455307	1.023036	-2.427597
C	-0.694400	0.673439	-0.496396
C	-0.714938	0.856793	0.706319
C	1.356211	-1.623099	-2.800849
C	2.539070	-1.326661	-2.261233
H	3.433696	-1.213700	-2.867409
H	2.658280	-1.196536	-1.189238
C	1.147876	-1.804230	-4.275082
H	0.826314	-2.829224	-4.500070
H	2.058271	-1.598273	-4.842918
H	0.350549	-1.146341	-4.636061
Si	-0.779702	1.142293	2.518155
C	0.224870	2.682003	2.923510
H	1.270287	2.566588	2.621671
H	0.205544	2.881730	4.000266
H	-0.175462	3.562670	2.412075
C	-0.054114	-0.356807	3.394685
H	0.986010	-0.527227	3.100704
H	-0.620026	-1.264478	3.164090
H	-0.076843	-0.214950	4.480633
C	-2.573240	1.384177	3.032985
H	-2.646887	1.556535	4.112291
H	-3.175459	0.503108	2.791337
H	-3.020621	2.243415	2.524159

S18

O	1.073435	0.556548	0.857912
C	-0.313159	0.808996	0.653807

H	-0.455739	1.501354	-0.186016
H	-0.633982	1.313385	1.571777
C	1.749278	0.108143	-0.301243
H	2.746220	-0.189514	0.038245
H	1.261046	-0.784077	-0.721365
C	1.863306	1.128815	-1.344706
C	1.948093	1.969171	-2.205481
C	-1.109764	-0.453424	0.444995
C	-1.830051	-0.631672	-0.663014
H	-2.426562	-1.525600	-0.822881
H	-1.858027	0.118355	-1.448420
C	-1.032810	-1.466094	1.548395
H	0.009256	-1.726318	1.760804
H	-1.579066	-2.379609	1.301487
H	-1.445423	-1.060155	2.480798
H	2.033948	2.711694	-2.965092

6b

O	0.712710	0.066175	1.400814
C	-0.673710	0.257964	1.149230
H	-0.819547	0.962723	0.320066
H	-1.054612	0.725338	2.064378
C	1.455991	-0.303682	0.250874
H	2.450578	-0.566765	0.624647
H	1.028189	-1.205371	-0.212217
C	1.559640	0.756507	-0.752237
C	1.641463	1.632714	-1.580030
C	-1.402898	-1.034314	0.882508
C	-2.084595	-1.214945	-0.249376
H	-2.633881	-2.130997	-0.448378
H	-2.126559	-0.446188	-1.015946

C	-1.309666	-2.073712	1.959411
H	-0.262974	-2.296739	2.190124
H	-1.811529	-3.001515	1.674167
H	-1.760894	-1.711289	2.891735
C	1.751680	2.681310	-2.580676
H	0.823184	3.257411	-2.656216
H	1.959468	2.262272	-3.571244
H	2.557962	3.383105	-2.341634

S20

O	1.081033	0.481622	0.913177
C	-0.303179	0.760561	0.726252
H	-0.440459	1.503294	-0.070584
H	-0.615848	1.214426	1.672858
C	1.747900	0.074286	-0.265041
H	2.743143	-0.246399	0.058152
H	1.248974	-0.795120	-0.718832
C	1.871124	1.133341	-1.269506
C	1.966483	2.007527	-2.096072
C	-1.115553	-0.476787	0.444302
C	-1.837700	-0.578870	-0.672064
H	-2.447118	-1.453268	-0.882521
H	-1.855180	0.216141	-1.412307
C	-1.055048	-1.553017	1.487024
H	-0.017440	-1.839958	1.686614
H	-1.613112	-2.442948	1.186371
H	-1.465192	-1.196561	2.440411
Cl	2.107172	3.191730	-3.228233

S21

C	1.347206	-2.877418	2.514468
H	0.367942	-2.829919	3.003981

H	1.370202	-3.808864	1.935952
C	-0.740697	0.327544	1.305170
H	-1.781661	0.584691	1.521849
H	-0.237952	0.213405	2.270687
C	-0.106313	1.369181	0.514343
C	0.421284	2.184229	-0.209826
C	1.584533	-1.686375	1.627542
C	2.529250	-0.784645	1.903288
H	2.700054	0.075111	1.262763
H	3.161189	-0.874379	2.782111
C	0.744414	-1.554989	0.380411
H	0.684281	-2.516262	-0.139056
H	1.216816	-0.854358	-0.310281
C	1.048658	3.107297	-1.091992
C	2.303619	4.888762	-2.848535
C	1.338694	2.722169	-2.411052
C	1.393630	4.399586	-0.668699
C	2.015895	5.279843	-1.543521
C	1.962133	3.608617	-3.277905
H	1.067888	1.723300	-2.737076
H	1.170354	4.700376	0.349236
H	2.278868	6.276833	-1.203842
H	2.183222	3.297881	-4.294290
H	2.792142	5.579326	-3.528628
H	2.103516	-2.944366	3.300616
C	-0.706207	-1.045247	0.586228
C	-1.355336	-0.896854	-0.794656
C	-1.536759	-2.033287	1.399104
O	-0.789033	-0.995131	-1.854905
O	-2.007272	-1.826286	2.492518
O	-2.661301	-0.605699	-0.670441
O	-1.664364	-3.199532	0.744182

C	-3.360686	-0.393397	-1.899872
H	-2.937856	0.460607	-2.432812
H	-4.394455	-0.197480	-1.619899
H	-3.292079	-1.278754	-2.534889
C	-2.416357	-4.207286	1.424967
H	-3.422693	-3.847867	1.648424
H	-1.923042	-4.483553	2.359369
H	-2.451727	-5.056810	0.744964

S24

C	1.349125	-3.183068	2.242885
H	0.468487	-3.009347	2.871656
H	1.158743	-4.100145	1.673049
C	-0.531702	0.196749	1.316321
H	-1.507984	0.531328	1.677888
H	0.085164	0.015511	2.202713
C	0.077131	1.220165	0.481908
C	0.572367	2.062104	-0.243154
C	1.617816	-2.022451	1.324839
C	2.694623	-1.248461	1.477771
H	2.894016	-0.409607	0.817550
H	3.412558	-1.427159	2.272885
C	0.656008	-1.779101	0.188221
H	0.452471	-2.714924	-0.341636
H	1.109357	-1.100760	-0.536835
H	2.195230	-3.364152	2.910664
C	-0.708156	-1.152404	0.574739
C	-1.508609	-0.919902	-0.711820
C	-1.526337	-2.081922	1.467557
O	-1.107377	-1.094858	-1.834786
O	-1.875607	-1.844567	2.599600
O	-2.740204	-0.466536	-0.419411

O	-1.802906	-3.232098	0.830216
C	-3.564789	-0.165848	-1.547991
H	-3.096249	0.604329	-2.163723
H	-4.507486	0.191441	-1.136605
H	-3.723143	-1.059667	-2.155190
C	-2.561776	-4.182058	1.583083
H	-3.527552	-3.759821	1.868052
H	-2.020628	-4.470684	2.486563
H	-2.695121	-5.039233	0.925188
Si	1.300927	3.325197	-1.352060
C	1.515731	4.928480	-0.389299
H	2.177835	4.786919	0.470071
H	0.555233	5.295913	-0.015616
H	1.950740	5.708792	-1.023270
C	0.140254	3.592262	-2.809509
H	0.549283	4.337758	-3.499924
H	-0.839780	3.946349	-2.475472
H	-0.009554	2.662651	-3.366815
C	2.969150	2.702671	-1.963224
H	3.659237	2.534480	-1.130597
H	3.432029	3.427592	-2.641481
H	2.861444	1.758081	-2.505002

S23

C	1.689569	2.051419	1.492713
H	1.574027	1.164581	2.126201
H	2.610379	1.913895	0.913437
C	-1.644154	0.028321	0.617904
H	-1.970510	-0.957204	0.960151
H	-1.476456	0.630905	1.515935
C	-2.673064	0.641186	-0.208669
C	-3.525404	1.141072	-0.899905

C	0.504492	2.245571	0.587178
C	-0.328580	3.277690	0.737364
H	-1.187290	3.415502	0.087564
H	-0.182496	4.014682	1.521649
C	0.301455	1.251077	-0.529121
H	1.247412	1.061580	-1.045616
H	-0.384361	1.662541	-1.272110
H	1.827737	2.909587	2.155332
C	-0.287658	-0.121850	-0.112906
C	-0.480726	-0.961182	-1.380762
C	0.659731	-0.887792	0.807368
O	-0.286454	-0.589324	-2.510395
O	0.429908	-1.200706	1.951502
O	-0.920656	-2.191780	-1.063683
O	1.815110	-1.161960	0.179388
C	-1.173018	-3.056711	-2.173773
H	-1.928351	-2.620390	-2.830206
H	-1.530846	-3.990684	-1.743352
H	-0.257402	-3.221791	-2.745730
C	2.780865	-1.875849	0.955915
H	2.378606	-2.840556	1.271804
H	3.058590	-1.300610	1.841627
H	3.640535	-2.012186	0.301961
H	-4.280992	1.580968	-1.508765

S22

C	1.862507	-2.117021	1.682088
H	0.976805	-1.990972	2.315109
H	1.715028	-3.036783	1.103645
C	-0.132589	1.234401	0.786591
H	-1.118218	1.563451	1.127138
H	0.465453	1.064466	1.687577

C	0.487650	2.255351	-0.046400
C	0.995251	3.098216	-0.747457
C	2.069224	-0.934487	0.776083
C	3.106946	-0.109360	0.930954
H	3.253546	0.748435	0.281835
H	3.839084	-0.260388	1.718867
C	1.081705	-0.724816	-0.345070
H	0.890361	-1.669569	-0.863413
H	1.500801	-0.039847	-1.084379
H	2.718967	-2.263824	2.345159
C	-0.289335	-0.126949	0.064243
C	-1.122027	0.067240	-1.206652
C	-1.062534	-1.067842	0.984050
O	-0.740431	-0.106609	-2.336705
O	-1.379033	-0.835525	2.126698
O	-2.362005	0.484909	-0.894012
O	-1.339458	-2.225006	0.358394
C	-3.222838	0.735352	-2.007001
H	-2.792262	1.501028	-2.655296
H	-4.164144	1.078038	-1.580001
H	-3.373345	-0.177477	-2.587579
C	-2.060016	-3.184679	1.135643
H	-3.023829	-2.777150	1.447661
H	-1.488869	-3.462254	2.024127
H	-2.198675	-4.046155	0.484380
C	1.602099	4.120776	-1.584405
H	2.679477	3.958911	-1.698131
H	1.462258	5.119339	-1.156130
H	1.162443	4.126801	-2.587612

S25

C	1.679249	2.023367	1.528316

H	1.527805	1.145642	2.166794
H	2.608536	1.856741	0.970577
C	-1.627892	0.025018	0.632133
H	-1.951052	-0.959179	0.981445
H	-1.458145	0.633065	1.526332
C	-2.663256	0.631167	-0.192020
C	-3.525595	1.120407	-0.879477
C	0.520450	2.240648	0.594555
C	-0.284701	3.298380	0.715878
H	-1.120666	3.460082	0.042243
H	-0.134779	4.038572	1.496333
C	0.316890	1.244415	-0.519653
H	1.263509	1.052799	-1.034508
H	-0.365585	1.657087	-1.265151
H	1.825385	2.883817	2.186138
C	-0.273844	-0.127092	-0.103131
C	-0.473399	-0.964909	-1.371304
C	0.672774	-0.899333	0.813222
O	-0.293689	-0.587377	-2.501474
O	0.437485	-1.227744	1.951912
O	-0.901192	-2.199230	-1.053620
O	1.831694	-1.161596	0.187611
C	-1.155930	-3.064309	-2.163770
H	-1.916921	-2.630830	-2.815462
H	-1.506480	-4.000541	-1.732381
H	-0.242520	-3.223782	-2.740747
C	2.796907	-1.881328	0.960096
H	2.396220	-2.850513	1.263875
H	3.069140	-1.314496	1.852866
H	3.659390	-2.007879	0.307912
Cl	-4.709789	1.788932	-1.805540

4a

C	1.301238	-1.375728	-2.089221
H	1.647754	-0.987455	-1.122828
H	1.544932	-0.647104	-2.866357
C	-0.723742	-2.292820	-0.971358
H	-0.257256	-3.284218	-1.007096
C	-0.507808	-1.694245	0.345924
C	-0.305133	-1.169413	1.418704
C	1.970914	-2.688951	-2.409691
C	2.763137	-3.283629	-1.517146
H	3.270511	-4.217598	-1.740545
H	2.940113	-2.854613	-0.535138
C	1.697631	-3.249144	-3.772837
H	0.622360	-3.377301	-3.934349
H	2.191412	-4.212675	-3.919527
H	2.043424	-2.559802	-4.552752
C	-0.077993	-0.528168	2.668128
C	0.362741	0.751090	5.117734
C	-0.242971	-1.225019	3.874685
C	0.313395	0.819998	2.707043
C	0.532548	1.449169	3.924784
C	-0.024677	-0.585933	5.087562
H	-0.543922	-2.266605	3.846704
H	0.439913	1.358393	1.774135
H	0.839775	2.490418	3.943652
H	-0.156604	-1.134852	6.014629
H	0.533316	1.246971	6.067985
H	-1.788242	-2.435240	-1.173663
N	-0.158585	-1.525952	-2.083946
S	-1.104057	-0.318974	-2.773588
O	-0.397891	0.087847	-3.985815
O	-2.461676	-0.857345	-2.805388

C	-1.128517	1.096519	-1.681569
C	-1.160208	3.282873	0.054965
C	-2.160092	1.224742	-0.754030
C	-0.122783	2.053416	-1.764458
C	-0.144541	3.136213	-0.892299
C	-2.169683	2.314473	0.103009
H	-2.951111	0.484961	-0.721895
H	0.648942	1.963632	-2.520040
H	0.636574	3.887790	-0.957441
H	-2.975437	2.416658	0.824299
C	-1.177419	4.448143	1.003388
H	-0.349228	5.135008	0.812547
H	-1.100725	4.108905	2.042542
H	-2.110328	5.015566	0.920562

4e

C	0.599682	-1.316088	-2.353511
H	1.195676	-1.081243	-1.462647
H	0.784547	-0.548913	-3.109646
C	-1.261360	-2.028933	-0.857801
H	-0.990026	-3.074717	-1.045381
C	-0.663934	-1.589629	0.403105
C	-0.141522	-1.214408	1.435501
C	0.975574	-2.667041	-2.910525
C	1.853576	-3.443821	-2.275294
H	2.153011	-4.408490	-2.674432
H	2.311288	-3.140139	-1.338267
C	0.314868	-3.051709	-4.199589
H	-0.775048	-3.032058	-4.098119
H	0.619370	-4.048389	-4.527689
H	0.559129	-2.335225	-4.993209
H	-2.352165	-1.983383	-0.816176

N	-0.829997	-1.265418	-2.030065
S	-1.722780	0.081622	-2.483007
O	-1.250041	0.432240	-3.819800
O	-3.117363	-0.261365	-2.213638
C	-1.295989	1.443342	-1.405580
C	-0.604810	3.542970	0.302051
C	-2.064341	1.688866	-0.272124
C	-0.189961	2.235589	-1.701129
C	0.149923	3.273605	-0.843888
C	-1.713983	2.735800	0.570606
H	-2.934562	1.076146	-0.068044
H	0.380008	2.054892	-2.605341
H	1.010826	3.894399	-1.075566
H	-2.319424	2.934664	1.450633
C	-0.255158	4.696151	1.199582
H	0.824502	4.865866	1.233889
H	-0.606298	4.530424	2.221487
H	-0.717422	5.624543	0.843446
Si	0.649002	-0.586742	2.968171
C	1.964506	0.665722	2.472067
H	2.752246	0.198290	1.873393
H	2.433552	1.109813	3.356624
H	1.527079	1.473975	1.877995
C	-0.661243	0.246023	4.032302
H	-0.213775	0.660022	4.942343
H	-1.441834	-0.459202	4.332700
H	-1.139732	1.066288	3.488949
C	1.427719	-2.024867	3.896602
H	2.182875	-2.528215	3.285681
H	0.677136	-2.768018	4.181655
H	1.916673	-1.673236	4.811683

4d

C	1.372648	-1.245170	-0.848210
H	1.704980	-0.876122	0.130496
H	1.604258	-0.489378	-1.602860
C	-0.644708	-2.249785	0.213149
H	-0.182384	-3.239781	0.125219
C	-0.422789	-1.736482	1.566511
C	-0.219847	-1.330287	2.683588
C	2.073736	-2.533955	-1.199878
C	2.872737	-3.134196	-0.317164
H	3.402088	-4.050895	-0.560148
H	3.032440	-2.727012	0.677022
C	1.820468	-3.064643	-2.578620
H	0.748701	-3.206986	-2.751623
H	2.331612	-4.015835	-2.744959
H	2.159694	-2.350600	-3.338880
H	-1.710573	-2.375953	0.010607
N	-0.083316	-1.425933	-0.856589
S	-1.049367	-0.233124	-1.540510
O	-0.353651	0.185404	-2.754698
O	-2.398927	-0.792072	-1.568405
C	-1.087715	1.178473	-0.445955
C	-1.141733	3.364886	1.289344
C	-2.093503	1.272360	0.511276
C	-0.113670	2.167195	-0.555292
C	-0.146070	3.248396	0.315358
C	-2.111827	2.362257	1.370717
H	-2.858667	0.507043	0.567372
H	0.641273	2.098868	-1.329858
H	0.610816	4.022815	0.230509
H	-2.898159	2.438068	2.116475
C	-1.188502	4.557683	2.201695

H	-0.186968	4.942300	2.413188
H	-1.668449	4.315232	3.153590
H	-1.760720	5.376292	1.748934
H	-0.053907	-0.959062	3.668597

4b

C	1.344786	-1.184991	1.208987
H	1.673986	-0.203617	0.844476
H	1.542907	-1.928776	0.432882
C	-0.623601	-0.081405	2.262619
H	-0.098100	-0.143053	3.222497
C	-0.449518	1.253549	1.687738
C	-0.294964	2.350976	1.206740
C	2.084525	-1.564907	2.467651
C	2.902497	-0.695842	3.062286
H	3.456224	-0.956566	3.959525
H	3.053545	0.305303	2.668931
C	1.841514	-2.952982	2.978102
H	0.771875	-3.128699	3.131397
H	2.366263	-3.135261	3.918921
H	2.171497	-3.700567	2.246436
H	-1.677273	-0.291337	2.458865
N	-0.103909	-1.178281	1.441074
S	-1.106433	-1.848555	0.273103
O	-0.464529	-3.098603	-0.127214
O	-2.452036	-1.811400	0.841092
C	-1.108975	-0.785717	-1.164620
C	-1.093145	0.900099	-3.390743
C	-2.020181	0.264012	-1.238869
C	-0.194151	-1.008322	-2.189524
C	-0.191447	-0.162507	-3.291663
C	-2.004708	1.097862	-2.349044

H	-2.740445	0.411078	-0.443289
H	0.486506	-1.849463	-2.129784
H	0.518238	-0.336147	-4.095349
H	-2.720022	1.913050	-2.413191
C	-1.108856	1.783644	-4.605881
H	-0.116211	1.864871	-5.057534
H	-1.458490	2.791438	-4.365533
H	-1.781294	1.382179	-5.373488
C	-0.132699	3.663676	0.605208
H	0.882485	4.050041	0.744883
H	-0.825784	4.389475	1.044118
H	-0.330078	3.625745	-0.471809

S17

C	1.375064	-1.252087	-0.863175
H	1.712712	-0.881258	0.113326
H	1.602438	-0.496230	-1.619148
C	-0.638019	-2.257120	0.205447
H	-0.152125	-3.237160	0.134449
C	-0.449438	-1.726550	1.558274
C	-0.277005	-1.310318	2.677766
C	2.076342	-2.539863	-1.218248
C	2.890801	-3.132802	-0.344753
H	3.421473	-4.047519	-0.592117
H	3.064604	-2.720503	0.644977
C	1.807326	-3.076796	-2.591558
H	0.734336	-3.226638	-2.750003
H	2.322262	-4.025237	-2.761630
H	2.131471	-2.362810	-3.358354
H	-1.698292	-2.408561	-0.009377
N	-0.080531	-1.433997	-0.865098
S	-1.052041	-0.245799	-1.551486

O	-0.360660	0.168390	-2.769446
O	-2.399847	-0.808999	-1.572176
C	-1.088334	1.168547	-0.461067
C	-1.136783	3.358409	1.269933
C	-2.089125	1.262276	0.502004
C	-0.117579	2.159218	-0.578681
C	-0.147395	3.242330	0.290077
C	-2.104248	2.353402	1.359597
H	-2.852829	0.495840	0.563499
H	0.632926	2.091060	-1.357562
H	0.607105	4.018227	0.199266
H	-2.885924	2.429071	2.110179
C	-1.182075	4.551945	2.181197
H	-0.188916	4.984931	2.327720
H	-1.588592	4.291200	3.162175
H	-1.821568	5.339305	1.764439
Cl	-0.067535	-0.723905	4.199555
