# Supplementary figures and images for: Systematic Parameter Determination Aimed at a Catalyst-Controlled Asymmetric Rh(I)-Catalyzed Pauson–Khand Reaction
Source: ACS Catal. 2024 Nov 5;14(22):17065–76. doi: 10.1021/acscatal.4c04490 (PMC11574763; doi:10.1021/acscatal.4c04490)

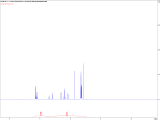

Supplement: Supplementary file 4 — cs4c04490_si_004.zip [file cs4c04490_si_004.zip › FIDs for publication/9b/1H/pdata/1/thumb.png]

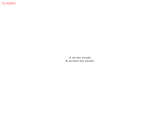

Supplement: Supplementary file 4 — cs4c04490_si_004.zip [file cs4c04490_si_004.zip › FIDs for publication/11a/13C/pdata/1/thumb.png]
